# Sensing Synthon Architectures with Hyperfine-Resolved Rotational Spectroscopy

**DOI:** 10.1021/jacs.6c06316

**Published:** 2026-07-20

**Authors:** Nuno M. Campos, Rita J. C. Roque, Tiddo J. Mooibroek, Melanie Schnell, Sérgio R. Domingos

**Affiliations:** † CFisUC, Department of Physics, 37829 University of Coimbra, Rua Larga, Coimbra 3004-516, Portugal; ‡ European Patent Office, Patentlaan 3-9, Rijswijk 2288 EE, The Netherlands; § 28332 Deutsches Elektronen-Synchrotron DESY, Notkestr. 85, 22607 Hamburg, Germany; ∥ Institut für Physikalische Chemie, Christian-Albrechts-Universität zu Kiel, Max-Eyth-Str.1, 24118 Kiel, Germany

## Abstract

Supramolecular synthons that engage in noncovalent carbon bonding offer promising routes toward programmable molecular architectures, yet experimental strategies capable of unambiguously resolving their docking geometries remain limited. Here, we show that electric field gradients at nitrogen nuclei—accessed through nuclear quadrupole splittings—provide a uniquely sensitive probe of supramolecular topology. Using hyperfine-resolved broadband rotational spectroscopy in a supersonic jet expansion combined with quantum-chemical calculations, we investigate tetracyanocyclopropane (TCCP), a synthetically accessible synthon bearing four cyano groups that act as local sensors of the electronic environment. Complexes formed between TCCP derivatives and tetrahydrofuran are observed in the jet expansion and shown to dock through a tetrel bond between an sp^3^-hybridized carbon center and the oxygen lone pair of tetrahydrofuran. While the rotational constants alone struggle to discriminate between candidate docking geometries, the nitrogen nuclear quadrupole coupling constants encode the orientational fingerprint of the complex through their electric field gradients. By exploiting symmetry relationships among the four nitrogen sites and implementing a tailored spectral fitting strategy to disentangle the dense hyperfine manifold, we extract precise rotational parameters that uniquely define the docking arrangements of two TCCP derivatives with different symmetries. These results demonstrate nitrogen electric field gradients as powerful structural sensors of noncovalent interactions and position hyperfine-resolved rotational spectroscopy as a configuration-sensitive approach for identifying supramolecular synthon motifs and guiding the design of nitrogen-rich supramolecular materials.

## Graphical abstract

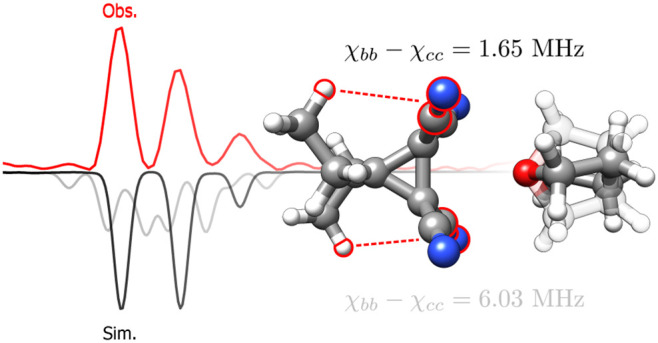

## Introduction

Recent advances in supramolecular chemistry focus on exploring noncovalent interactions to influence several fundamental processes behind molecular self-assembly, protein folding, molecular recognition, among many others.^1–9^ In this context, the design and synthesis of supramolecules arise as a fundamental tool to manipulate localized molecular interactions with the goal of triggering a macroscale effect in biological systems.^10–12^ To custom-build these supramolecules, chemists have at their disposal a diverse portfolio of supramolecular synthons with different structures to form the desired inter- and intramolecular interactions.^13–16^

Tetrel bonds are one category of noncovalent interactions that can be explored in the context of supramolecular chemistry. They are governed by interactions between a group-14 element (i.e., C, Si, Ge, Sn, Pb) with an electrophilic region and a nucleophilic center, such as a lone pair or a π or σ bond.^17–21^ The strength of such tetrel bonds can be deeply influenced by the type of substituents used. For example, theoretical studies on 1,4-diazabicyclo[2.2.2]­octane (DABCO) complexes with various TH_3_X molecules[Fn fn1] identified that the highly electrophilic −NO_2_ substituent can significantly enhance tetrel bond strength, whereas methyl groups weaken it. The authors were also able to pinpoint that charge transfer between the N lone pair in DABCO and the σ*­(T–X) antibonding orbital plays a significant role in mediating these noncovalent interactions.^22^ Tetrel bonds are also currently being explored in new carbon capture technologies through physical desorption.^23,24^ Notable observations using rotational spectroscopy have identified the C···S tetrel bond as the dominant intermolecular interaction between CO_2_ and phenyl isothiocyanate.^25^ A similar study also reported the first spectroscopic evidence of a tetrel bond involving −C≡C– and CO_2_ when studying the carbon capture capabilities of 2-butynyl alcohol.^26^

Tetrel bonding can also be engineered to direct crystal packing and design new molecular materials with specific structural properties.^27^ In this context, tetracyanocyclopropane (TCCP) is a versatile synthon that is easy to synthesize in the lab and has a predisposition to form tetrel bonds with lone pairs or π electrons.^15^ In fact, recent studies on TCCP···tetrahydrofuran (THF) complexes demonstrated that the sp^3^-hybridized carbon centers can be used as anchor points in the design of supramolecular materials.^28^ In this previous work, we have demonstrated experimentally that 3,3-dimethyl-TCCP (2MeTCCP) interacts with THF via sp^3^-C···O bonds both in the crystalline state and in the gas phase, but with distinct binding geometries. Whereas X-ray diffraction measurements reveal that O and C atoms in THF are almost coplanar with the cyclopropane ring plane of 2MeTCCP, the analysis of rotational spectroscopy data led to the assignment of the perpendicular topology. This conclusion was guided with strong support from energetic considerations, since a comparison between fitted and predicted rotational parameters alone struggled to provide a direct assignment on the docking geometry for the 2MeTCCP-THF complex in the gas phase. We note that this previous work does not report the nuclear quadrupole coupling constants (NQCCs) for this synthon complex, mainly because the hyperfine splitting produced by the four ^14^N nuclei challenges the frequency resolution of any chirped-pulse Fourier transform microwave (CP-FTMW)^29^ spectrometer due to the numerous overlapping hyperfine components. However, it is well-known that the information provided by the NQCCs can be essential to reach definitive assignments for some molecular systems.^30–37^ This could also be the case for the 2MeTCCP···THF complex, which holds four ^14^N structural probes.

In this work, we revisit this molecular system and pursue a rigorous deconvolution of the hyperfine-resolved rotational spectra in an attempt to solve the 2MeTCCP···THF docking geometry exclusively from spectroscopic data. Additionally, we further extend our investigation to a second TCCP topology comprising a diethyl back tail, 3,3-diethyl-TCCP (2EtTCCP), which changes the symmetry of the host from C_2*v*_ to C_2_. The complexes of both 2MeTCCP and 2EtTCCP with THF were created and cooled down in a neon-based supersonic jet expansion and probed with a chirp pulse covering the 2–8 GHz frequency range with the COMPACT spectrometer.^38^ Aided by the highly symmetric placements of the four ^14^N nuclei in both TCCP topologies, their respective NQCCs could be determined from the measured rotational spectra. These parameters are deeply linked to the local electric field gradient (EFG) and carry essential information on the interactions that are established close to each ^14^N nuclei. We demonstrate that, although the primary rotational constants alone are insufficient to determine the docking orientation of THF with respect to TCCP, there is vital EFG information enclosed in the NQCCs to confidently identify the preferred binding topology in the gas phase.

## Results and Discussion

Figure 1 shows portions of the hyperfine-resolved rotational spectrum of free 2MeTCCP and 2EtTCCP, as well as their corresponding THF adducts. The depicted spectral regions highlight the rich hyperfine structure of these species. The experimental spectrum (upper traces) is shown in red, while the lower traces (in blue) are simulations based on the fitted spectroscopic parameters (Table 1) obtained by employing a recurring fit using the Watson Hamiltonian^39^ in the I^*r*^ representation as implemented in PGOPHER.^40^

**Figure 1:**
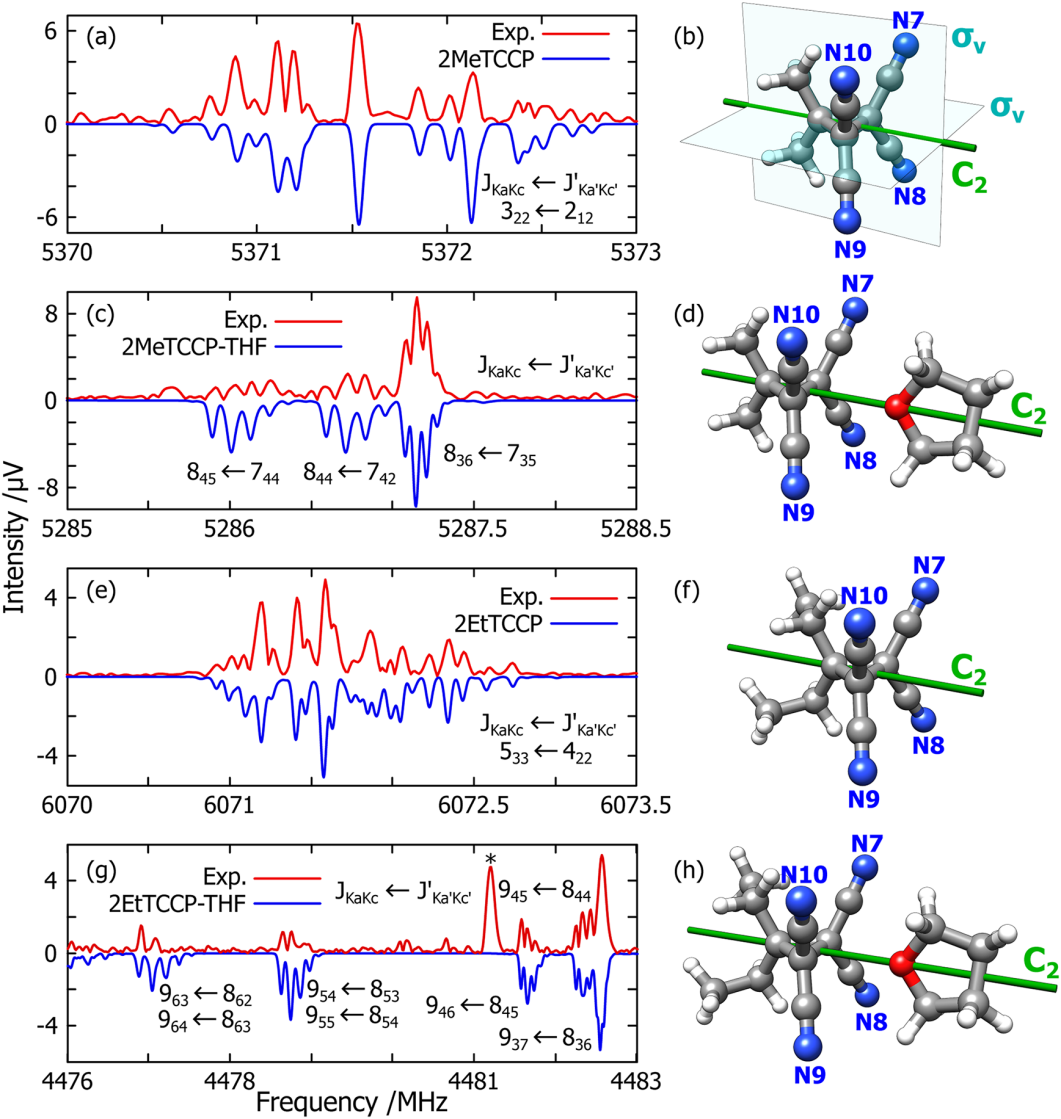
Rotational spectra and molecular structures of free and complexed TCCP. Groups of rotational transitions are identified by the corresponding rotational quantum numbers as $J_{K_{a}K_{c}}\leftarrow J_{K_{a}^{\prime }K_{c}^{\prime }}^{\prime }$ where *J* is the rotational angular momentum quantum number, *K_a_* and *K_c_* are the projections of *J* onto the principal axes at the prolate and oblate symmetric top limits, respectively. The extra angular momentum quantum numbers needed to specify a state, which include the nuclear spins, I­(^14^N) = 1, are omitted as the hyperfine transitions for each group overlap. The corresponding molecular structures are illustrated on the right, determined using B3LYP-D3BJ/def2-TZVP.^41–46^ The top two sets of panels correspond to 2MeTCCP and its THF complex, while the bottom two correspond to 2EtTCCP and its THF complex. The peak just above 4481 MHz and marked with an asterisk does not belong to any of the species addressed in this work.

**Table 1:** Experimental and Predicted Spectroscopic Constants for the Observed TCCP···THF Complexes^a^

	2MeTCCP		2MeTCCP···THF			2EtTCCP		2EtTCCP···THF		
Constants	Theory	Experiment	Perp.	Coplanar	Experiment	Theory	Experiment	Perp.	Coplanar	Experiment
A/MHz	939.56	946.41840(90)	635.01	633.19	632.8304(62)	652.06	652.74599(17)	555.82	554.95	554.8909(69)
B/MHz	853.08	844.12774(34)	340.53	325.75	342.57009(16)	600.92	600.01083(18)	258.57	251.51	258.79462(13)
C/MHz	692.30	691.57073(92)	316.80	322.41	316.78560(16)	563.10	560.16849(19)	238.04	239.46	238.08484(12)
Δ_*J*_ /kHz	–	–	–	–	0.00977(54)	–	0.0180(25)	–	–	0.00501(18)
Δ_*JK*_ /kHz	–	–0.194(66)	–	–	0.1154(38)	–	–	–	–	0.0457(11)
Δ_*K*_ /kHz	–	0.349(56)	–	–	–	–	0.0252(51)	–	–	2.60(45)
δ_*K*_ /kHz	–	–0.189(53)	–	–	–	–	–	–	–	0.00139(16)
χ_*aa*_ /MHz										
N7 & N9	–2.77	–2.4909(41)	1.58	1.60	1.384(17)	–4.41	–3.9028(33)	1.58	1.60	1.4076(90)
N8 & N10			1.58	1.64	1.487(15)	0.85	0.5128(43)	1.55	1.58	1.473(12)
χ_*bb*_-χ_*cc*_ /MHz										
N7 & N9	–0.64	–0.5158(68)	–4.53	5.94	–3.512(40)	–1.11	–0.8858(60)	–6.92	–6.33	–5.830(31)
N8 & N10			–2.98	–6.35	–3.291(32)	4.15	3.4701(62)	1.70	6.03	1.653(29)
μ_*a*_ /D	0.0	no	2.6	2.6	yes	0.0	no	–3.1	–3.1	yes
μ_*b*_ /D	0.0	no	0.0	0.0	no	5.9	yes	0.0	0.0	no
μ_*c*_ /D	–5.4	yes	0.0	0.0	no	0.0	no	0.0	0.0	no
N_*line*_	–	870	–	–	1500	–	1854	–	–	3183
N_*freq*_	–	135	–	–	157	–	436	–	–	391
σ /kHz	–	22.21	–	–	21.94	–	17.49	–	–	17.56
Δ*E*_ZPE_/kJ/mol	–	–	0.0	+4.5	–	–	–	0.0	+4.3	–

a The A, B, C parameters are the rotational constants; Δ*_J_*, Δ*_JK_*, Δ*_K_, δ_K_* are the quartic centrifugal distortion constants; and χ_aa_, χ_bb_-χ_cc_ are the NQCCs associated with the various ^14^N atoms labeled in Figure 1. The theoretical parameters including those calculated for the perpendicular (Perp.) and coplanar docking geometries were obtained at the B3LYP-D3BJ/def2-TZVP^41–46^ level of theory. The predicted dipole moment components, μ_i_, for the a-, b-, and c-type transitions are also shown, as well as the standard error for each fit, σ. Given the large number of overlapped lines, we chose to include both the number of transitions included in the fit, N_*line*_, as well as the number of unique frequencies they were assigned to, N_*freq*_.

A close analysis of the molecular structures presented in Figure 1 reveals that both free TCCP forms and their complexes with THF possess inherent symmetry features. This means that there are nitrogen nuclei that experience the same EFG with respect to the center of mass of the system and, consequently, can be considered equivalent during the fitting process. Free 2MeTCCP presents a C_2*v*_ symmetry, as highlighted by the *C*_*2*_ symmetry axis and the two mirror planes σ_ν_ shown in Figure 1b. This anticipates that all nitrogen nuclei should be equivalent and generate the same NQCCs. In the diethyl variant, 2EtTCCP (Figure 1f), the relative position of the two ethyl groups breaks the mirror symmetry, and the system is reduced to C_2_, meaning that only the diagonal nitrogen pairs (N7–N9 and N8–N10) should be equivalent. Our theoretical calculations predict that upon docking with THF, both forms have *C*_*2*_ symmetry (Figure 1d and h), with two pairs of N-equivalent centers just like in free 2EtTCCP. These symmetry considerations for the free and complexed species were carefully used to link the NQCCs of equivalent N nuclei in PGOPHER.^40^

To efficiently fit the rich hyperfine pattern captured for both free TCCP topologies and their complexes, we employed a spectral fitting strategy using the fringe transitions of each highly convoluted group of lines as initial guidance to resolve the complex spectral patterns. After these outer lines were included in the fit, we continued assigning the rotational transitions from the outside toward the inner, more convoluted part of each line branch. With this methodology, we reached a good agreement for the shape and relative intensities of each group of transitions present in the spectrum. In the spectral segments shown in Figure 1 (panels a, c, e, g), selected groups of lines attest to this agreement. We note that the ultimate frequency of each line belonging to these complex patterns is not unambiguously resolved, but Table 1 demonstrates that the experimental NQCCs of all species are determined to at least three decimal digits, a notably high level of accuracy considering that the frequency resolution of the experimental spectrum is 25 kHz.

A careful comparison between fitted and predicted rotational parameters of both TCCP···THF complexes (Table 1) reveals a striking similarity between primary constants (A, B, C) for the perpendicular and coplanar configurations (shown in Figure 2), which renders the assignment far from trivial. The magnitude of the dipole moments also does not aid in an unambiguous assignment, as highlighted in the former study for the case of 2MeTCCP.^28^

**Figure 2:**
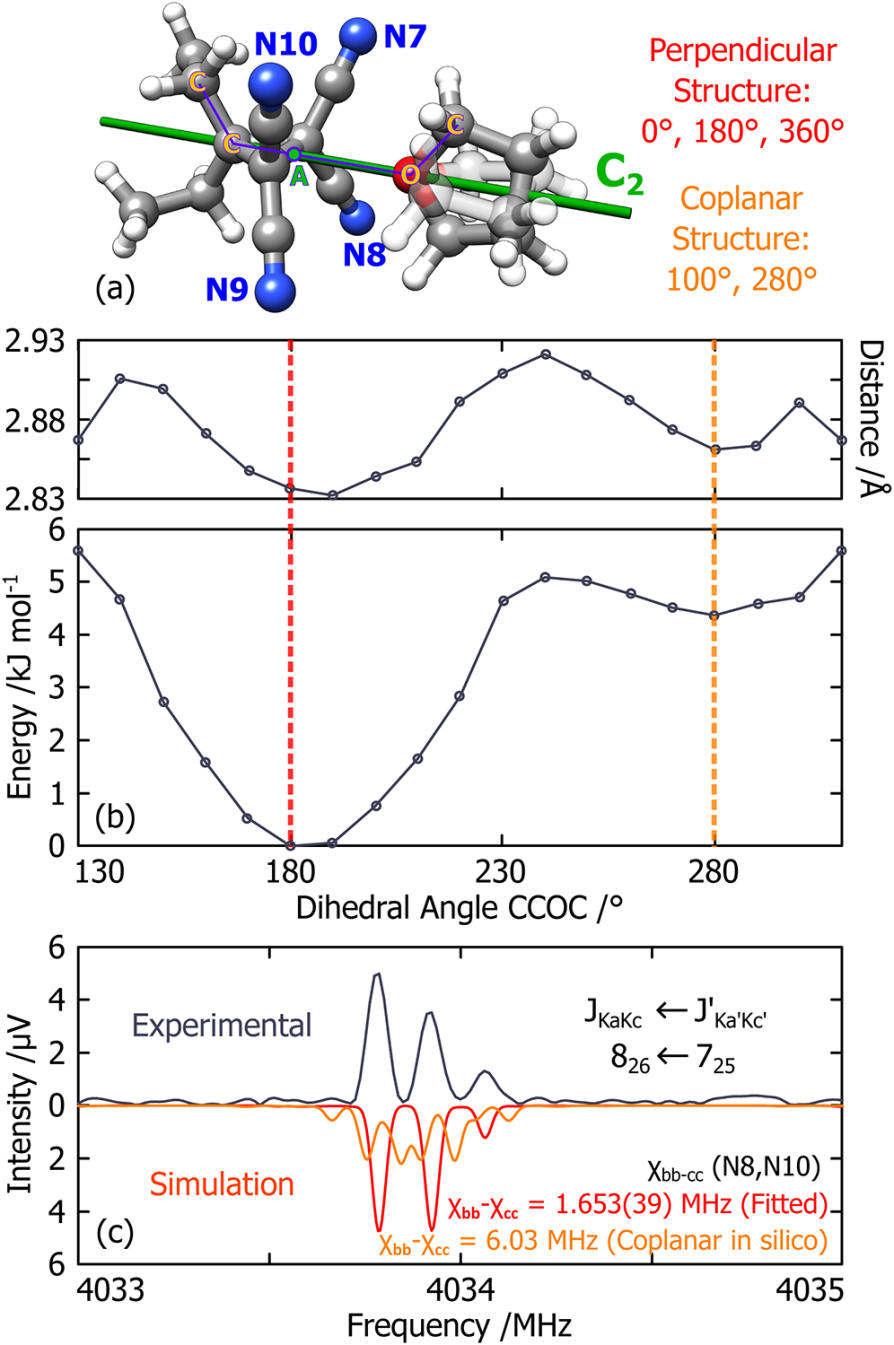
Docking configuration and nuclear quadrupole coupling effects in the 2EtTCCP–THF complex. (a) Molecular structures of perpendicular and coplanar conformations obtained at the B3LYP-D3BJ/def2-TZVP^41–46^ level of theory. The coplanar configuration is shown with semitransparency for clarity, with the *C_2_* axis depicted in coincidence with the inertial axis a. (b) Zero-point corrected energy (*E*_ZPE_) and distance between point A in 2EtTCCP and the O atom in THF as a function of the dihedral angle CCOC, as depicted in panel a. The vertical traces, red and orange lines, mark the perpendicular and coplanar geometries, respectively. (c) Segment of the rotational spectrum showing the experimental (upper trace) and the simulated (lower traces) for the perpendicular (in red) and coplanar (in orange) configurations. The simulated spectral depictions differ exclusively in the χ*_bb_*-χ*_cc_* parameter, which is altered *in silico* for the coplanar geometry to match the predicted values as given in Table 1.

In this work, however, we have the additional information provided by the NQCCs due to an improved and more detailed spectral analysis. We note that for both 2MeTCCP and 2EtTCCP complexes, the NQCCs χ_*aa*_ also barely differ between the perpendicular and coplanar forms, and that the fitted χ_*aa*_ is consistent with both topologies. In fact, we have conducted a relaxed energy scan to evaluate the magnitude of the NQCCs as a function of the THF rotation relative to 2EtTCCP, which is shown in Figure S1 of the Supporting Information. This scan demonstrates that, although the χ_*aa*_ values change for some dihedral angles, they are very similar for the coplanar and perpendicular docking geometries of the 2EtTCCP complex. On the other hand, the magnitude of the predicted and fitted χ_*bb*_-χ_*cc*_ carries key information to allow a more conclusive assignment. In the case of 2MeTCCP···THF, theory predicts a notable difference between the perpendicular and coplanar geometries for the χ_*bb*_-χ_*cc*_ of the two equivalent N pairs, allowing a confident assignment to the perpendicular topology based on the sign and magnitude of the fitted χ_*bb*_-χ_*cc*_ values. For 2EtTCCP···THF, we find that only the χ_*bb*_-χ_*cc*_ for the N8–N10 pair changes between perpendicular and coplanar docking, while χ_*bb*_-χ_*cc*_ for the N7–N9 pair remains unchanged. The agreement between experiment and theory is established for the perpendicular docking configuration based on the information enclosed in the χ_*bb*_-χ_*cc*_ for the N8–N10 pair alone. The χ_*bb*_-χ_*cc*_ values highlighted in Table 1 are thus the only parameters that allow an unambiguous assignment to the perpendicular geometry of both TCCP···THF adducts. It is important to note that the inertial axis *a* coincides with the *C*_*2*_ axis (see Figure 1d and h), and thus it is not surprising that χ_*aa*_ is not sensitive to the different docking configurations. χ_*bb*_-χ_*cc*_, however, seems to be able to probe differences occurring in the electronic environment perpendicular to the *C*_*2*_ axis, consistent with the pronounced effects measured and predicted for inertial axes *b* and *c*.

To further support the assignment of the perpendicular topology, Figure 2 shows the main differences between the perpendicular- and coplanar-docking structures for the case of 2EtTCCP···THF. Figure 2b shows the zero-point corrected energy (*E*_ZPE_) as a function of the dihedral angle CCOC, highlighting that a perpendicular docking is energetically more favorable by ∼4 kJ mol^–1^. The energy scan also reveals a relatively small energy barrier between the two topologies, strongly suggesting that a conformational relaxation to the perpendicular form may occur in the supersonic jet. Furthermore, and to underline that the hyperfine splitting is only compatible with the perpendicular docking topology, we have altered the χ_*bb*_-χ_*cc*_ values of the N8 and N10 pair *in silico* to match those predicted for the coplanar geometry (Table 1, Figure 2c). It is clear that the agreement between this coplanar simulation is not as compatible with the experimental spectrum as the fitted value of χ_*bb*_-χ_*cc*_ = 1.623(29) MHz, which is characteristic of the perpendicular docking topology. These considerations further confirm the assignment reported in the previous work.^28^

Another relevant aspect of this docking interaction emerges when one recalls that the rotational spectrum of free THF has a characteristic splitting pattern that has been previously assigned to a puckering motion involving pseudorotations of the five-carbon ring.^47,48^ Interestingly, we do not observe any line splitting in the rotational spectra of either synthon complex, suggesting that the puckering motion of THF is quenched upon docking with TCCP. The calculated puckering barrier of 9 kJ/mol for THF in docked mode confirms this assignment, as this value is notably higher than the one reported for free THF^47,48^ (about 0.54 kJ/mol).[Fn fn2]

To assess more accurately the intermolecular linkages that participate in both 2MeTCCP···THF and 2EtTCCP···THF complexes, we performed auxiliary noncovalent interaction (NCI)^49–52^ analyses, which are described in detail in the SI. Figure 3 shows the rendering of the NCI surfaces for both monomeric species. For 2MeTCCP (Figure 3a), all the dispersion-driven interactions between the dimethyl moiety and the CN groups are depicted in a symmetric arrangement, as expected. For the 2EtTCCP version, however, the diethyl group engages in additional interactions with the CN groups, as seen in Figure 3b and d. We find that because of the diagonal positioning of the ethyl groups with respect to the carbon ring, the CN groups that contain the N7 and N9 atoms are interacting more strongly with the ethyl groups. Strikingly, these are the same nuclei whose χ_*bb*_-χ_*cc*_ value remains insensitive to the docking orientation of the complex (Table 1). Moreover, the NCI analysis reveals that the CN groups that contain the N8 and N10 atoms do not engage as significantly with the diethyl backbone. For these atoms, the χ_*bb*_-χ_*cc*_ is distinct for the two possible docking geometries (Table 1) and the sole decisive parameter that allows the assignment of the perpendicular topology. These considerations strongly suggest that the diethyl back tail distorts the local CN electronic environments considerably more for those comprising nuclei N7 and N9. This intramolecular interaction seems to produce such a noticeable effect that, when THF docks, those particular CN groups are effectively less sensitive to the relative orientation of THF. This influence on the local EFG is directly expressed in the NQCCs of the diagonal N7 and N9 atoms of 2EtTCCP, minimizing their contribution to χ_*bb*_-χ_*cc*_ as a probe of the docking orientation of THF.

**Figure 3:**
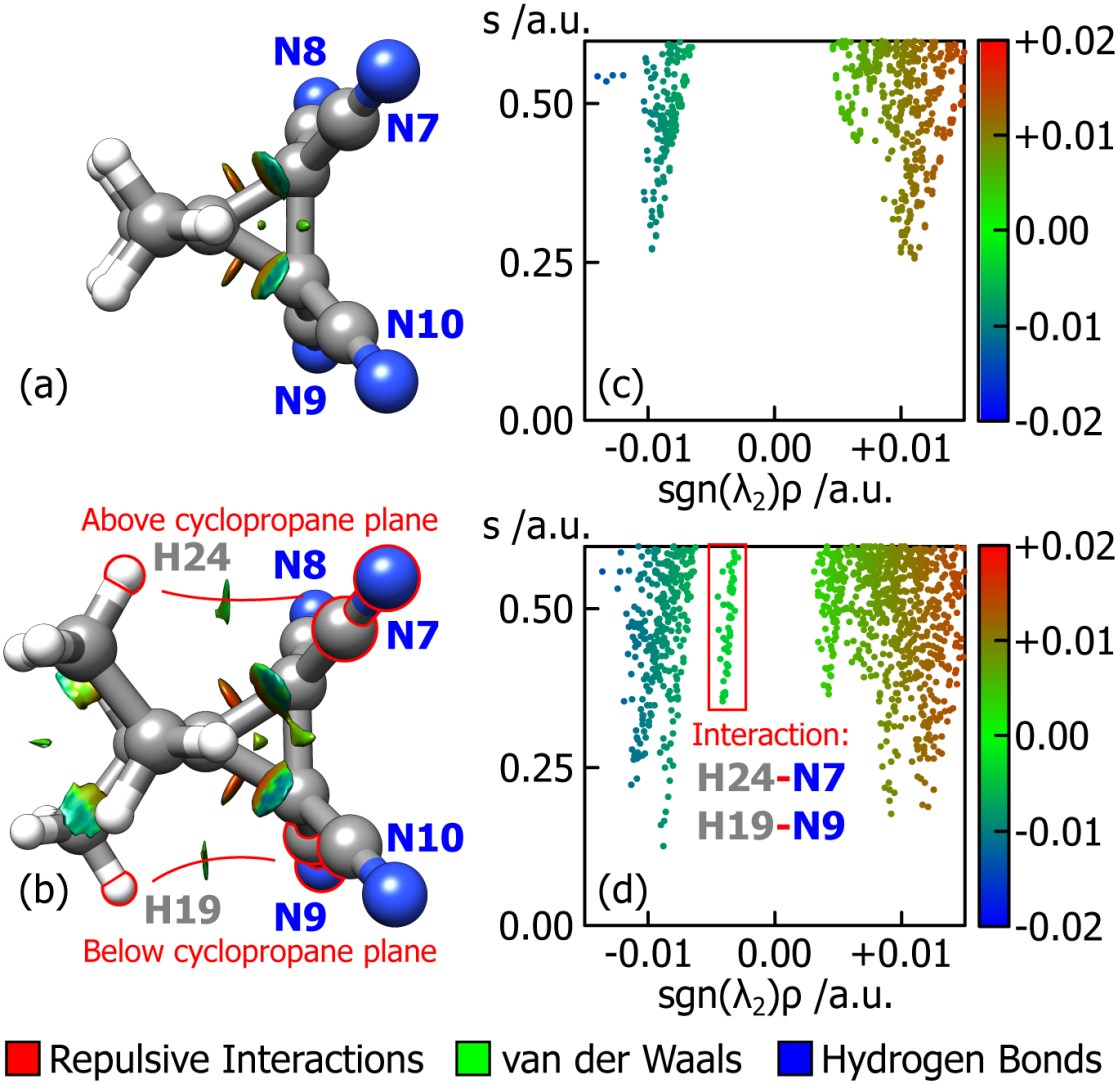
NCI analyses of both TCCP complexes. (a–b) Molecular structures of 2MeTCCP and 2EtTCCP as determined using B3LYP-D3BJ/def2-TZVP.^41–46^ Relevant intramolecular interactions are depicted, and the NCI surfaces are plotted (s = 0.5 a.u.). (c–d) Reduced density gradient as a function of the electronic density multiplied by the sign of the second-largest eigenvalue of the Hessian matrix. For 2EtTCCP (panel d), the valley in the reduced density gradient that corresponds to the interaction between the ethyl backbone and the cyano groups (red highlights in panel b) is indicated with a red box. Details on how this interaction was isolated from the others are provided in the SI.

## Conclusions

In this work we use hyperfine-resolved rotational spectroscopy and quantum chemistry methods to establish a preferred perpendicular docking topology between THF and TCCP, demonstrating that the lone pair of THF interacts with the sp^3^-hybridized carbon centers of the TCCP synthon through a tetrel bond. A definitive assignment is reached after careful symmetry considerations are taken into account, in tandem with a methodical spectral analysis to resolve the complex hyperfine splitting produced by the four N nuclei. The information embedded in the local electric field gradient was crucial to determine the correct docking geometry between the two TCCP···THF synthon complexes, given that an unambiguous assignment could not be obtained using solely the primary rotational constants nor the magnitude of the dipole moment components. For one of the synthon complexes, and a particularly challenging topology, 2EtTCCP-THF, we disclose that the χ_*bb*_-χ_*cc*_ values also express an asymmetric interaction between the two pairs of CN groups and the diethyl back tail.

As a final remark, we reiterate that rotational spectroscopy, being sensitive to the electric field gradients near nitrogen nuclei, offers a uniquely local sensing tool to probe supramolecular synthons while being exploited to probe noncovalent interactions. Because the EFG directly reflects subtle redistributions of electron density, created here as a consequence of tetrel bonding, it encodes structural and electronic information that is often averaged out in bulk observables. Therefore, comparisons between experimentally derived NQCCs and theoretical predictions can expose systematic mispredictions of synthon topologies in the condensed-phase realm. In this context, gas-phase blueprints validated by rotational spectroscopy provide a chemically transparent reference that isolates intrinsic interaction motifs before environmental perturbations intervene. Embedding such experimentally grounded EFG benchmarks into theoretical workflows can, therefore, refine force fields and electronic structure methods, enabling predictive corrections for condensed-phase distortions. Ultimately, leveraging nitrogen EFGs as design parameters may guide the rational engineering of novel nitrogen-based supramolecules whose architectures comprise finely tuned noncovalent interactions.

## Supplementary Material



## Footnotes

1 with T = C, Si, Ge and X= −Me, −H, −OH, −NH_2_, −F, −Cl, −Br, −I, −CN, −NO_2_.

2 It should be noted that the energy values cannot be directly compared since the calculated barrier for docked THF involves a transition through the planar configuration, while for free THF, the puckering occurs through a series of pseudorotations of the ring.

## Notes

Computational and experimental methods, Cartesian coordinates of the molecular structures of all species, auxiliary quantum chemistry calculations (potential energy surface scans of the docking configurations, noncovalent interaction analyses, symmetry adapted perturbation theory calculations) and comprehensive list of the rotational transitions assigned for each species (PDF)
