# Supplementary material for: Sensing Synthon Architectures with Hyperfine-Resolved Rotational Spectroscopy
Source: J Am Chem Soc. 2026 Jul 20;148(30):32026–32. doi: 10.1021/jacs.6c06316 (PMC13449956; doi:10.1021/jacs.6c06316)
Supplement: Supplementary file 1 [file ja6c06316_si_001.pdf]

Supporting Information

Sensing Synthon Architectures with Hyperfine-Resolved Rotational Spectroscopy

Nuno M. Campos[1], Rita J.C. Roque[1], Tiddo J. Mooibroek[2], Melanie Schnell[3,4] and Sérgio R. Domingos[1]

[1]CFisUC, Department of Physics, University of Coimbra, Rua Larga, 3004-516 Coimbra, Portugal
E-mail: sergio.domingos@uc.pt.
[2]European Patent Office Patentlaan 3-9, 2288 EE Rijswijk, The Netherlands.
[3]Deutsches Elektronen-Synchrotron DESY, Notkestr. 85, 22607 Hamburg, Germany E-mail: melanie.schnell@desy.de.
[4]Institut für Physikalische Chemie, Christian-Albrechts Universität zu Kiel, Max-Eyth-Str.1, 24118 Kiel, Germany.

Contents

1 Methods **2**
- 1.1 Computational Methods 2
- 1.2 Experimental Methods 2

2 Molecular Structure **3**
- 2.1 3,3-Dimethyl-1,1,2,2-tetracyanocyclopropane 3
- 2.2 3,3-Diethyl-1,1,2,2-tetracyanocyclopropane 6

3 Additional Calculations and Analysis **9**
- 3.1 Docking configuration and nuclear quadrupole coupling effects in the 3,3-diethyl-1,1,2,2-tetracyanocyclopropane-tetrahydrofuran complex 9
- 3.2 Non-covalent Interaction Analysis 11
- 3.3 Symmetry Adapted Perturbation Theory 13

4 Rotational Transitions **14**
- 4.1 3,3-Dimethyl-1,1,2,2-tetracyanocyclopropane 14
- 4.2 Cluster of 3,3-dimethyl-1,1,2,2-tetracyanocyclopropane with tetrahydrofuran 31
- 4.3 3,3-Diethyl-1,1,2,2-tetracyanocyclopropane 59
- 4.4 Cluster of 3,3-diethyl-1,1,2,2-tetracyanocyclopropane with tetrahydrofuran 95

1. Methods

1.1. Computational Methods

The starting geometries of 3,3-dimethyl-1,1,2,2-tetracyanocylopropane and 3,3-diethyl-1,1,2,2-tetracyanocyclopropane and their 1:1 clusters with tetrahydrofuran were directly optimized through quantum chemistry calculations with ORCA 5.0 [1, 2], using the B3LYP-D3BJ/def2-TZVP [3, 4, 5, 6] level of theory with the RIJCOSX approximation. The optimized geometries were then used as input to a geometry optimization calculation using the Symmetry Module of ORCA in order to detect the point group of the molecule, clean up its coordinates (in order to perfectly obey the symmetry group) and recalculate the molecular properties in these symmetrized geometries.

The recurring fits to the experimental rotational spectra used the Watson's A-reduced semirigid rotor Hamiltonian [7] in the I^r representation and were conducted using PGOPHER [8]. The groups of rotational transitions are identified throughout the paper by the corresponding rotational quantum numbers as $J_{K_aK_c} \leftarrow J'_{K'_aK'_c}$, where J is the rotational angular momentum quantum number and K_a and K_c are the projections of J onto the principal axes at the prolate and oblate symmetric top limits, respectively. The rotational transitions presented later in the Supporting Information use the seven quantum numbers used in PGOPHER to identify the states: J, K_a, K_c, $I_{7,9}$, F_1, $I_{8,10}$ and F_2. These last four quantum numbers are angular momentum quantum numbers obtained using the following coupling schemes between the rotational angular momentum and the nuclear spins of the four nitrogen nuclei (I(^{14}N) = 1): $\mathbf{I_{7,9}} = \mathbf{I_{N7}} + \mathbf{I_{N9}}$, $\mathbf{F_1} = \mathbf{J} + \mathbf{I_{7,9}}$, $\mathbf{I_{8,10}} = \mathbf{I_{N8}} + \mathbf{I_{N10}}$ and $\mathbf{F_2} = \mathbf{F_1} + \mathbf{I_{8,10}}$. The non-diagonal elements of the quadrupole coupling tensors were not determined in our analysis.

1.2. Experimental Methods

The rotational spectra used were obtained using the COMPACT [9] spectrometer in Hamburg, a Chirped-Pulse Fourier Transform (CP-FTMW) spectrometer operating in the 2-8 GHz range. A supersonic expansion is used to introduce the samples into the vaccum chamber where a 4 μs chirp spanning the 2-8 GHz frequency range is used to polarize the molecular emsemble. The molecular beam is generated using a Parker General Valve Series 9 operating at 9 Hz with a constant flow of neon. The chirp is generated with an arbitrary waveform generator and amplified by a 300 W travelling wave tube amplifier before being broadcast into the vaccum chamber through a horn antenna. For each molecular beam, eight of these chirped pulses are emitted into the vacuum chamber to excite the cold molecules, resulting in an effective repetition rate of 72 Hz. The resulting free induction decays (FIDs) of the macroscopic dipole moment of the molecular ensemble are collected by a second horn antenna, recorded by a digital oscilloscope and co-added and averaged to obtain the final spectrum, which is then Fourier-transformed into the frequency domain. To create sufficient vapour pressure the samples of 3,3-dimethyl-1,1,2,2-tetracyanocyclopropane and 3,3-diethyl-1,1,2,2-tetracyanocyclopropane were heated directly at the nozzle. An external reservoir containing tetrahydrofuran was placed before the nozzle to ensure the presence of this species in the molecular beam.

For 3,3-dimethyl-1,1,2,2-tetracyanocyclopropane, the sample was heated to 180 °C, a stagnation pressure of 1.5 bar of neon was used and 500k FIDs were co-added and averaged to obtain the final spectrum. For 3,3-diethyl-1,1,2,2-tetracyanocyclopropane, the sample was heated to 170 °C, a stagnation pressure of 3 bar of neon was used and 2600k FIDs were co-added and averaged to obtain the final spectrum.

All commercially available chemicals were purchased from Sigma Aldrich and used without further purification. 3,3-Dimethyl-1,1,2,2-tetracyanocyclopropane was synthesized according to the procedure described in the Supporting Information of Reference [10].

2. Molecular Structure

2.1. 3,3-Dimethyl-1,1,2,2-tetracyanocyclopropane

Table 1. *Equilibrium geometry of 3,3-Dimethyl-1,1,2,2-tetracyanocyclopropane.*

Atom	*a* /Å	*b* /Å	*c* /Å
C	0.00000000000000	-0.00000000000000	-1.19429040475827
C	0.00000000000000	0.78797597298790	0.12114560942031
C	0.00000000000000	-0.78797597298790	0.12114560942031
C	1.20989289572981	1.44464349342907	0.54120619085161
C	-1.20989289572981	1.44464349342907	0.54120619085161
C	1.20989289572981	-1.44464349342907	0.54120619085161
C	-1.20989289572981	-1.44464349342907	0.54120619085161
N	-2.18235356056408	1.97886594760925	0.84125222918790
N	2.18235356056408	1.97886594760925	0.84125222918790
N	2.18235356056408	-1.97886594760925	0.84125222918790
N	-2.18235356056408	-1.97886594760925	0.84125222918790
C	1.27206462446937	-0.00000000000000	-2.00750491252141
C	-1.27206462446937	-0.00000000000000	-2.00750491252141
H	2.17006348432189	-0.00000000000000	-1.39415642423622
H	1.29135942245549	0.88439875001284	-2.64534363797327
H	1.29135942245549	-0.88439875001284	-2.64534363797327
H	-2.17006348432189	-0.00000000000000	-1.39415642423622
H	-1.29135942245549	-0.88439875001284	-2.64534363797327
H	-1.29135942245549	0.88439875001284	-2.64534363797327

Table 2. *Equilibrium geometry of the complex of 3,3-dimethyl-1,1,2,2-tetracyanocyclopropane and tetrahydrofuran (perpendicular configuration).*

Atom	*a* /Å	*b* /Å	*c* /Å
C	2.4441243032	-0.0000000000	-0.0000000000
C	1.1259915109	0.0475733003	0.7804824573
C	1.1259915109	-0.0475733003	-0.7804824573
C	0.6935699499	-1.1149674095	1.5094302807
C	0.6965702590	1.2930304809	1.3583149365
C	0.6965702590	-1.2930304809	-1.3583149365
C	0.6935699499	1.1149674095	-1.5094302807
N	0.3845162876	2.2975074304	1.8212166256
N	0.3822265777	-2.0499598349	2.1005696744
N	0.3845162876	-2.2975074304	-1.8212166256
N	0.3822265777	2.0499598349	-2.1005696744
C	3.2566463360	-1.2698754130	0.0745726329
C	3.2566463360	1.2698754130	-0.0745726329
H	-4.7157901581	1.3520090023	-0.1723324544
H	-2.1439438296	-1.9078829359	-0.6553350224
C	-3.9542174680	0.7143249130	0.2759249921
H	-2.4778462612	1.6058157036	-1.0586319659
C	-2.5400587823	-1.1799188906	0.0524790470
O	-1.7137274920	0.0000000000	-0.0000000000
C	-2.5400587823	1.1799188906	-0.0524790470
H	-4.1107052335	0.7041577285	1.3569323316
H	-4.1107052335	-0.7041577285	-1.3569323316
C	-3.9542174680	-0.7143249130	-0.2759249921
H	-2.4778462612	-1.6058157036	1.0586319659
H	-2.1439438296	1.9078829359	0.6553350224
H	-4.7157901581	-1.3520090023	0.1723324544
H	2.6406777893	-2.1638078085	0.1402168099
H	3.9044576085	-1.2342873235	0.9513345931
H	3.8842235645	-1.3495952195	-0.8139605112
H	2.6406777893	2.1638078085	-0.1402168099
H	3.9044576085	1.2342873235	-0.9513345931
H	3.8842235645	1.3495952195	0.8139605112

Table 3. *Equilibrium geometry of the complex of 3,3-dimethyl-1,1,2,2-tetracyanocyclopropane and tetrahydrofuran (coplanar configuration).*

Atom	*a* /Å	*b* /Å	*c* /Å
C	2.4533632138	0.0000000000	0.0000000000
C	1.1342841674	-0.5399250044	0.5643594444
C	1.1342841674	0.5399250044	-0.5643594444
C	0.7138987195	-1.8685344786	0.2055908044
C	0.7033345950	-0.1209482679	1.8719545219
C	0.7033345950	0.1209482679	-1.8719545219
C	0.7138987195	1.8685344786	-0.2055908044
N	0.3909839120	0.2077163266	2.9278812895
N	0.4148839364	-2.9437884064	-0.0682818237
N	0.3909839120	-0.2077163266	-2.9278812895
N	0.4148839364	2.9437884064	0.0682818237
C	3.2664520591	-0.9161025593	-0.8819147456
C	3.2664520591	0.9161025593	0.8819147456
H	-4.7479235684	0.8742720674	-1.0457590618
H	-2.1809947223	-1.8489114875	0.8026195415
C	-3.9874115314	0.7117581530	-0.2823734302
H	-2.5100211998	0.4639596094	-1.8660596681
C	-2.5716698164	-0.8312796163	0.8367651761
O	-1.7465446519	0.0000000000	-0.0000000000
C	-2.5716698164	0.8312796163	-0.8367651761
H	-4.1449918114	1.4385163002	0.5175264043
H	-4.1449918114	-1.4385163002	-0.5175264043
C	-3.9874115314	-0.7117581530	0.2823734302
H	-2.5100211998	-0.4639596094	1.8660596681
H	-2.1809947223	1.8489114875	-0.8026195415
H	-4.7479235684	-0.8742720674	1.0457590618
H	2.6516457009	-1.5690545054	-1.4971562189
H	3.9125607561	-1.5376701704	-0.2606985844
H	3.8962269487	-0.3166146541	-1.5405291296
H	2.6516457009	1.5690545054	1.4971562189
H	3.9125607561	1.5376701704	0.2606985844
H	3.8962269487	0.3166146541	1.5405291296

2.2. *3,3-Diethyl-1,1,2,2-tetracyanocyclopropane*

Table 4. *Equilibrium geometry of 3,3-diethyl-1,1,2,2-tetracyanocyclopropane.*

Atom	*a* /Å	*b* /Å	*c* /Å
C	-0.0000000000	0.7879082654	-0.0000000000
C	0.3857208870	-0.5278905027	0.6860672699
C	-0.3857208870	-0.5278905027	-0.6860672699
C	-0.3400871367	-0.9516360453	1.8550040130
C	1.7602034112	-0.9555997703	0.6644077656
C	-1.7602034112	-0.9555997703	-0.6644077656
C	0.3400871367	-0.9516360453	-1.8550040130
N	2.8631354883	-1.2786711721	0.6512212311
N	-0.9155148720	-1.2709804775	2.7973898111
N	-2.8631354883	-1.2786711721	-0.6512212311
N	0.9155148720	-1.2709804775	-2.7973898111
C	-1.1309143578	1.5834211841	0.6271468653
C	1.1309143578	1.5834211841	-0.6271468653
H	-1.8540626720	0.9073413311	1.0823849937
H	-0.7115779944	2.1714077937	1.4455497823
C	-1.8440988613	2.4881525468	-0.3755266835
H	1.8540626720	0.9073413311	-1.0823849937
H	0.7115779944	2.1714077937	-1.4455497823
C	1.8440988613	2.4881525468	0.3755266835
H	-2.2383509619	1.9115287240	-1.2140466406
H	-2.6846554089	2.9854922647	0.1078521171
H	-1.1843716418	3.2601601408	-0.7719674140
H	2.6846554089	2.9854922647	-0.1078521171
H	1.1843716418	3.2601601408	0.7719674140
H	2.2383509619	1.9115287240	1.2140466406

Table 5. *Equilibrium geometry of the complex of 3,3-diethyl-1,1,2,2-tetracyanocyclopropane and tetrahydrofuran (perpendicular configuration).*

Atom	*a* /Å	*b* /Å	*c* /Å
C	-2.0109932412	-0.0000000000	0.0000000000
C	-0.6925725982	0.2970091648	-0.7232278750
C	-0.6925725982	-0.2970091648	0.7232278750
C	-0.2547035123	-0.5632031812	-1.7905126873
C	-0.2619977729	1.6626121242	-0.8672548835
C	-0.2619977729	-1.6626121242	0.8672548835
C	-0.2547035123	0.5632031812	1.7905126873
N	0.0499545004	2.7625530748	-0.9839521299
N	0.0647511414	-1.2487909726	-2.6557931987
N	0.0499545004	-2.7625530748	0.9839521299
N	0.0647511414	1.2487909726	2.6557931987
C	-2.8062310812	-1.1987028574	-0.4827893870
C	-2.8062310812	1.1987028574	0.4827893870
H	5.1459778496	1.2150322770	0.6169309501
H	2.5735007886	-2.0165245840	-0.0229003655
C	4.3838137128	0.7655274790	-0.0191632939
H	2.9064743010	1.1572027501	1.5371118959
C	2.9694070147	-1.0936206708	-0.4464821225
O	2.1440366705	0.0000000000	-0.0000000000
C	2.9694070147	1.0936206708	0.4464821225
H	4.5397087935	1.1198436585	-1.0406517766
H	4.5397087935	-1.1198436585	1.0406517766
C	4.3838137128	-0.7655274790	0.0191632939
H	2.9064743010	-1.1572027501	-1.5371118959
H	2.5735007886	2.0165245840	0.0229003655
H	5.1459778496	-1.2150322770	-0.6169309501
H	-2.1287216384	-1.9710076445	-0.8455521787
H	-3.3957057468	-0.8840757658	-1.3460837588
C	-3.7105901085	-1.7856960329	0.5988780294
H	-2.1287216384	1.9710076445	0.8455521787
H	-3.3957057468	0.8840757658	1.3460837588
C	-3.7105901085	1.7856960329	-0.5988780294
H	-3.1355327616	-2.0709843011	1.4815447763
H	-4.2038852343	-2.6817563210	0.2224713149
H	-4.4866274567	-1.0853363343	0.9090050321
H	-4.2038852343	2.6817563210	-0.2224713149
H	-4.4866274567	1.0853363343	-0.9090050321
H	-3.1355327616	2.0709843011	-1.4815447763

Table 6. *Equilibrium geometry of the complex of 3,3-diethyl-1,1,2,2-tetracyanocyclopropane and tetrahydrofuran (coplanar configuration).*

Atom	*a* /Å	*b* /Å	*c* /Å
C	-2.0192659163	0.0000000000	-0.0000000000
C	-0.6996098999	0.5383693533	-0.5646617912
C	-0.6996098999	-0.5383693533	0.5646617912
C	-0.2680698029	0.1263550578	-1.8744129179
C	-0.2705625076	1.8656012979	-0.2081934471
C	-0.2705625076	-1.8656012979	0.2081934471
C	-0.2680698029	-0.1263550578	1.8744129179
N	0.0384170944	2.9394900667	0.0599788746
N	0.0495571180	-0.1829491096	-2.9345914230
N	0.0384170944	-2.9394900667	-0.0599788746
N	0.0495571180	0.1829491096	2.9345914230
C	-2.8140380080	-0.9418165643	-0.8850520849
C	-2.8140380080	0.9418165643	0.8850520849
H	4.5717128447	1.5068199500	0.2584381015
H	2.9372524921	-0.1297832441	1.9189624391
C	4.4148633761	0.6515820368	-0.4025879312
H	2.6080842583	1.6788596553	-1.1159431491
C	2.9994731273	-0.6709815865	0.9698489581
O	2.1749083062	-0.0000000000	0.0000000000
C	2.9994731273	0.6709815865	-0.9698489581
H	5.1758746106	0.6782597623	-1.1822059776
H	5.1758746106	-0.6782597623	1.1822059776
C	4.4148633761	-0.6515820368	0.4025879312
H	2.6080842583	-1.6788596553	1.1159431491
H	2.9372524921	0.1297832441	-1.9189624391
H	4.5717128447	-1.5068199500	-0.2584381015
H	-2.1358138461	-1.5354396096	-1.4972512511
H	-3.3975927441	-0.3360039994	-1.5808734330
C	-3.7258263243	-1.8735499666	-0.0896840780
H	-2.1358138461	1.5354396096	1.4972512511
H	-3.3975927441	0.3360039994	1.5808734330
C	-3.7258263243	1.8735499666	0.0896840780
H	-3.1565980563	-2.4563041225	0.6366973049
H	-4.2166183759	-2.5753931586	-0.7638384715
H	-4.5038511714	-1.3278689804	0.4449835543
H	-4.2166183759	2.5753931586	0.7638384715
H	-4.5038511714	1.3278689804	-0.4449835543
H	-3.1565980563	2.4563041225	-0.6366973049

3. Additional Calculations and Analysis

3.1. Docking configuration and nuclear quadrupole coupling effects in the 3,3-diethyl-1,1,2,2-tetracyanocyclopropane-tetrahydrofuran complex

In an effort to understand the effects of the docking configuration on the 2EtTCCP-THF complex, we performed a relaxed surface scan by performing multiple constrained geometry optimizations. For each of these calculations, the THF molecule was rotated around the C_2 symmetry axis so that the dihedral angle specified in Figure 2 of the main paper assumed a specific value. This dihedral angle was fixed during each optimization, effectively locking the relative configuration of the 2EtTCCP and THF molecules, and allowing us to determine the effects of the docking configuration on multiple molecular properties. We performed calculations over a full 360° rotation in steps of 10°.

In Figure 2 of the main paper, we present the variation of the zero-point corrected energy as a function of the dihedral angle CCOC. In Figure 1 of the SI, we present the variation of the nuclear quadrupole coupling constants (NQCCs) for the N7-N9 and N8-N10 pairs as a function of the same dihedral angle. As shown in the figure, and as discussed in the main paper, only the difference between the *bb* and *cc* components of the NQCCs for the N8-N10 pair changes significantly between the perpendicular and coplanar configurations.

To get an idea of the shape of the potential energy surface, we ran similar calculations for rotations around two axes perpendicular to the C_2 symmetry axis of the molecular complexes. Like the calculations above, the initial geometries for the constrained geometry optimizations were generated from the minimum perpendicular and coplanar configurations by rotating the THF molecule around an axis passing through the center of mass of the minimum energy configurations of the complexes. As shown in Figure 2 of the SI:

The first axis used for the scan was the one that is simultaneously perpendicular to the C_2 symmetry axis and to the plane formed by the three carbon atoms of the cyclopropane ring. The movement caused by this rotation is illustrated in panels (a) and (b) of Fig. 2 and corresponds to the THF moving along the shorter edge of the rectangle formed by the four nitrogen atoms of the TCCP.

The second axis used for the scan was the one that is simultaneously perpendicular to the C_2 symmetry axis and parallel to the plane formed by the three carbon atoms of the cyclopropane ring. The movement caused by this rotation is illustrated in panels (c) and (d) of Fig. 2 and corresponds to the THF moving along the longer edge of the rectangle formed by the four nitrogen atoms of the TCCP.

As expected, the potential energy surface is different for movement in the two perpendicular directions. Furthermore, it grows faster when THF is moving along the longer edge of the rectangle formed by the four nitrogen atoms (panels (c) and (d)) when compared to when it is moving along the shorter edge (panels (a) and (b)). This is what is intuitively expected since movement along the longer edge forces the THF molecule to pass closer to the nitrogen atoms, since it encounters the shorter edge of the rectangle in its path instead of the longer edge it encounters when moving in the other direction.

To ensure that the rotation applied to THF was maintained during the calculation, we applied a dihedral angle constraint to lock the O atom of THF to the correct plane (defined by the O atom and the axis of rotation). We ran separate calculations for the perpendicular and coplanar configurations of the cluster. Although no constraints were applied to lock the configuration of the cluster, in the majority of the cases the initial configuration remained unchanged.

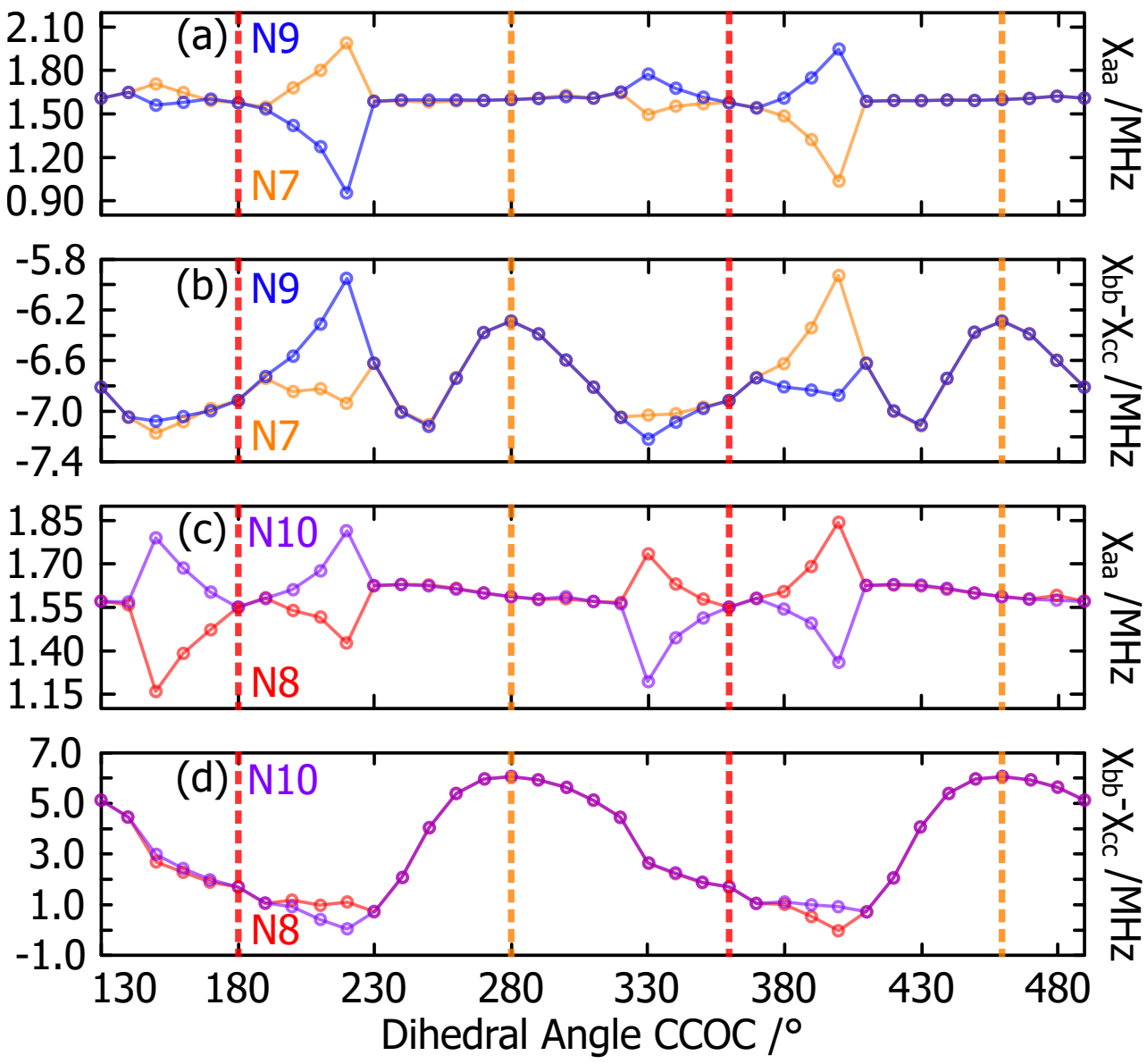

Figure 1. *Docking configuration and nuclear quadrupole coupling effects in the 2EtTCCP-THF complex. The angles corresponding to the perpendicular and coplanar configurations are indicated by vertical red and orange lines, respectively. (a,c) The aa component of the NQCCs as a function of the dihedral angle CCOC for the N7-N9 and N8-N10 pairs of nitrogen atoms. (b,d) The difference between the bb and cc components of the NQCCs as a function of the dihedral angle CCOC for the N7-N9 and N8-N10 pairs of nitrogen atoms.*

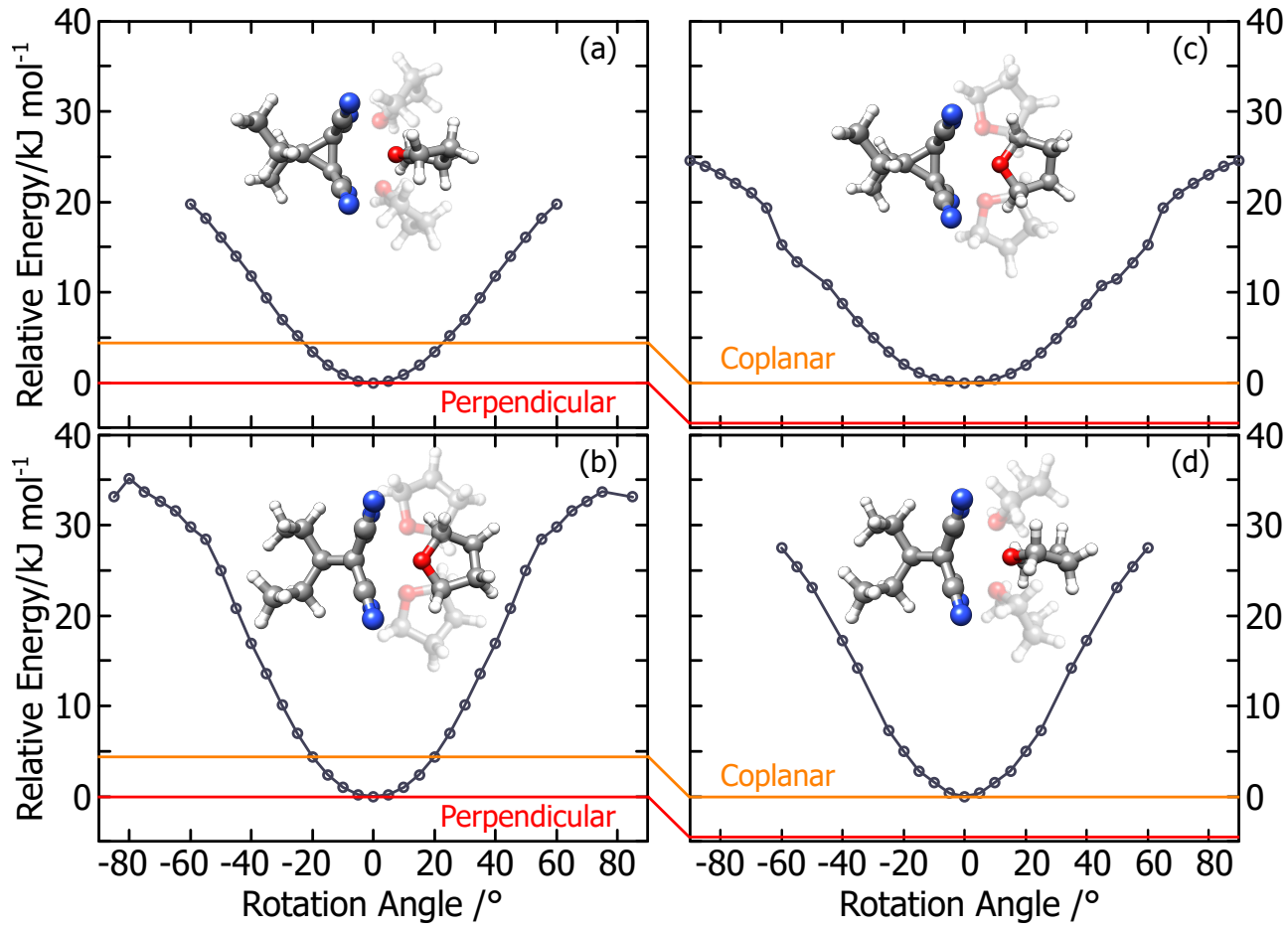

Figure 2. *Potential energy surface of the 2EtTCCP-THF complex for rotations around axes perpendicular to the C_2 symmetry axis of 2EtTCCP-THF (in the minimum energy configurations). (a) Rotation around the axis perpendicular to the plane of the cyclopropane ring for the perpendicular configuration. (b) Rotation around the axis perpendicular to the plane of the cyclopropane ring for the coplanar configuration. (c) Rotation around the axis parallel to the plane of the cyclopropane ring for the perpendicular configuration. (d) Rotation around the axis parallel to the plane of the cyclopropane ring for the coplanar configuration. The red and orange lines mark, respectively, the energy of minimum energy configurations of the perpendicular and coplanar forms of the 2EtTCCP-THF complex.*

3.2. *Non-covalent Interaction Analysis*

We performed a non-covalent interaction (NCI [11, 12]) analysis using NCIPLOT [11, 13, 14] to generate cube files of the reduced density gradient (s) and the electronic density multiplied by the sign of the second-largest eigenvalue of the Hessian matrix ($\mathrm{sgn}(\lambda_2)\rho$). In Figure 3 of the main paper, we present the results of this analysis in two complementary formats. In panels (a) and (b), isosurfaces of the reduced density gradient at $s = 0.5$ a.u. are displayed along with the molecular structures, colored according to the value of $\mathrm{sgn}(\lambda_2)\rho$, and visualized using Chimera [15]. In panels (c) and (d), the same data is shown as scatter plots of the reduced density gradient versus $\mathrm{sgn}(\lambda_2)\rho$ for various points in the system. Only the region relevant to non-covalent interactions is shown in this plot. The reduced density gradient approaches zero in regions where interactions occur, as such, valleys in s correspond to intermolecular or intramolecular interactions. Both of the NCI formats quantify the intramolecular non-covalent interactions in the two TCCP monomers as a color-density map; whereas repulsive interactions are represented in red, at the rightmost side of panels c) and d), attractive interactions are represented in blue, at the leftmost side. Van der Waals interactions are represented in green, nearest to the center of the x-axis. In panel (d), one of these central valleys is identified as arising from two specific interactions between the 2EtTCCP backtail hydrogen atoms and the cyano groups. The identification of these interactions was made by modifying the color scale of the reduced density gradient isosurfaces so that values of $\mathrm{sgn}(\lambda_2)\rho$ lying within a specific range appeared in one color, while values outside the range appeared in another. The initially selected range was centered on the valley of interest and then progressively narrowed until only the desired valley was within the range. This procedure revealed that only the interactions between the ethyl groups and the cyano groups significantly contributed to the highlighted feature. If the features in the scatter plot are not easily separated, one can do a similar procedure to instead link each region of the scatter plot to its corresponding isosurface. When doing so, an initial range is chosen, like before, and then adjusted until the interactions of interest are colored only by the color of the chosen range.

In Figures 3 and 4 of this SI we present, respectively, the scatter plots of s and $\mathrm{sgn}(\lambda_2)\rho$ and the isosurfaces of s together with the molecular structures for all the molecular species studied, complementing what is presented in Figure 3 of the main paper. In each scatter plot in Figure 3, we map each region to the corresponding interaction shown in Figure 4. We used the same color convention as for the ones of the main paper for the two TCCP monomers.

Features in the NCI scatter plots can only be attributed uniquely to a specific interaction when the superposition of the features from multiple interactions is minimal, such as in the case with 2MeTCCP (panel (a)), 2MeTCCP-THF (perpendicular) (panel (b)) and 2EtTCCP (panel (d)). For the remaining panels, the interactions cannot be easily deconvoluted.

1. **Summary of the NCI analysis for 2MeTCCP:** For the 2MeTCCP monomer there is a single feature in the attractive region (negative values of $\mathrm{sgn}(\lambda_2)\rho$) of the scatter plot at about -0.0095 a.u. (Fig. 3a) resulting from the intramolecular interaction between the H atoms in the backtail CH_3 groups and the CN groups (Fig. 4a). When going from the monomer to the complex, this feature remains present and mostly unchanged.
   However, the formation of the complex also gives rise to two new features. One of them is cleanly separated from the rest and is weaker (in pink, at a range of about -0.004 to -0.0015 a.u. for the perpendicular configuration, Fig. 3b, and -0.005 a.u. to -0.002 a.u for the coplanar configuration, 3c). It results from the interactions between the H atoms in the CH_2 groups of THF and the CN groups of 2MeTCCP (Fig. 4b and Fig. 4c). The second feature forms due to the presence of the tetrel bond, between the O atom of THF and the sp^3 carbons of 2MeTCCP. A careful analysis of the isosurface in Fig. 4b and Fig. 4c shows that there are two spatial regions of interest for this interaction: one directly connecting the O atom of THF with the center of the sp^3 carbons, where

the interaction is strongest, and a second, spreading from this central region roughly to the diagonal directions of the CN groups, where the interaction becomes weaker.

- **Perpendicular Configuration:** From the NCI scatter plot we can see that both portions of the tetrel bond interaction are at similar strengths and cleanly separated from the rest of the features, contributing to the points seen in the range of about -0.0075 a.u. to -0.004 a.u. (Fig. 3b, in purple).
- **Coplanar Configuration:** Here the tetrel bond interaction is quite a bit stronger at the center than at the edges, so this interaction contributes to points in a much wider range from about -0.0115 a.u. to -0.005 a.u. (Fig. 3c, in purple). In general, here we see that going from the perpendicular to coplanar configuration the intermolecular interactions move to regions with more negative values of $\mathrm{sgn}(\lambda_2)\rho$, that is, they are more attractive for the coplanar configuration than for the perpendicular configuration. As for the repulsive part of the NCI scatter plot, the valleys in s also move to more positive values, and so, represent stronger repulsive interactions. All of this is in line with the results of the SAPT calculations.

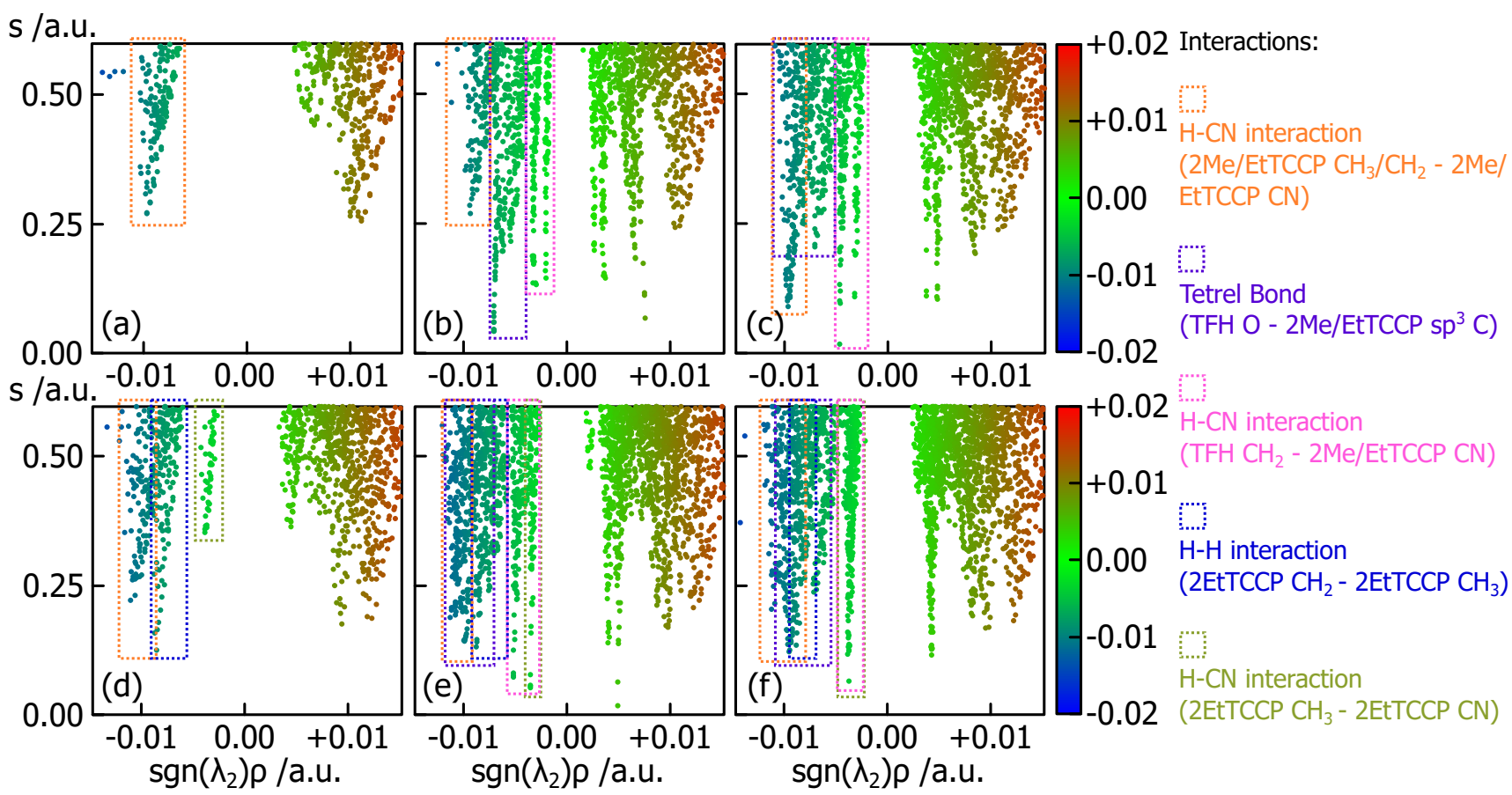

Figure 3. *Scatter plots of the reduced density gradient and of the electronic density multiplied by the sign of the second-largest eigenvalue of the Hessian matrix together with identification of the interactions which result in the features present in the scatter plots. (a) 2MeTCCP, (b) 2MeTCCP-THF (perpendicular), (c) 2MeTCCP-THF (coplanar), (d) 2EtTCCP, (e) 2EtTCCP-THF (perpendicular) and (f) 2EtTCCP-THF (coplanar).*

2. **Summary of the NCI analysis for 2EtTCCP:** For the 2EtTCCP monomer there are two additional features (Fig. 3d) that are not present in the NCI scatter plot for the 2MeTCCP monomer. These correspond to the extra intramocular interactions that are present in this species: one is between the backtails (in blue) and the other interaction is between the H atoms in the backtail and two of the CN groups (in green) that are highlighted in the main paper (Fig. 4d). These interactions remain mostly unchanged when 2EtTCCP complexes with THF.
The new features that appear in the scatter plots of 2EtTCCP-THF in Fig. 3e and 3f reflect the formation of the tetrel bond and the interaction between the H atoms of THF and the CN groups of 2EtTCCP-THF (Fig. 4e and 4f).

- **Perpendicular Configuration:** For this configuration, the interaction between the H atoms of THF and the CN groups contributes to points in the range of -0.006 a.u. to -0.0025 a.u. (in pink,

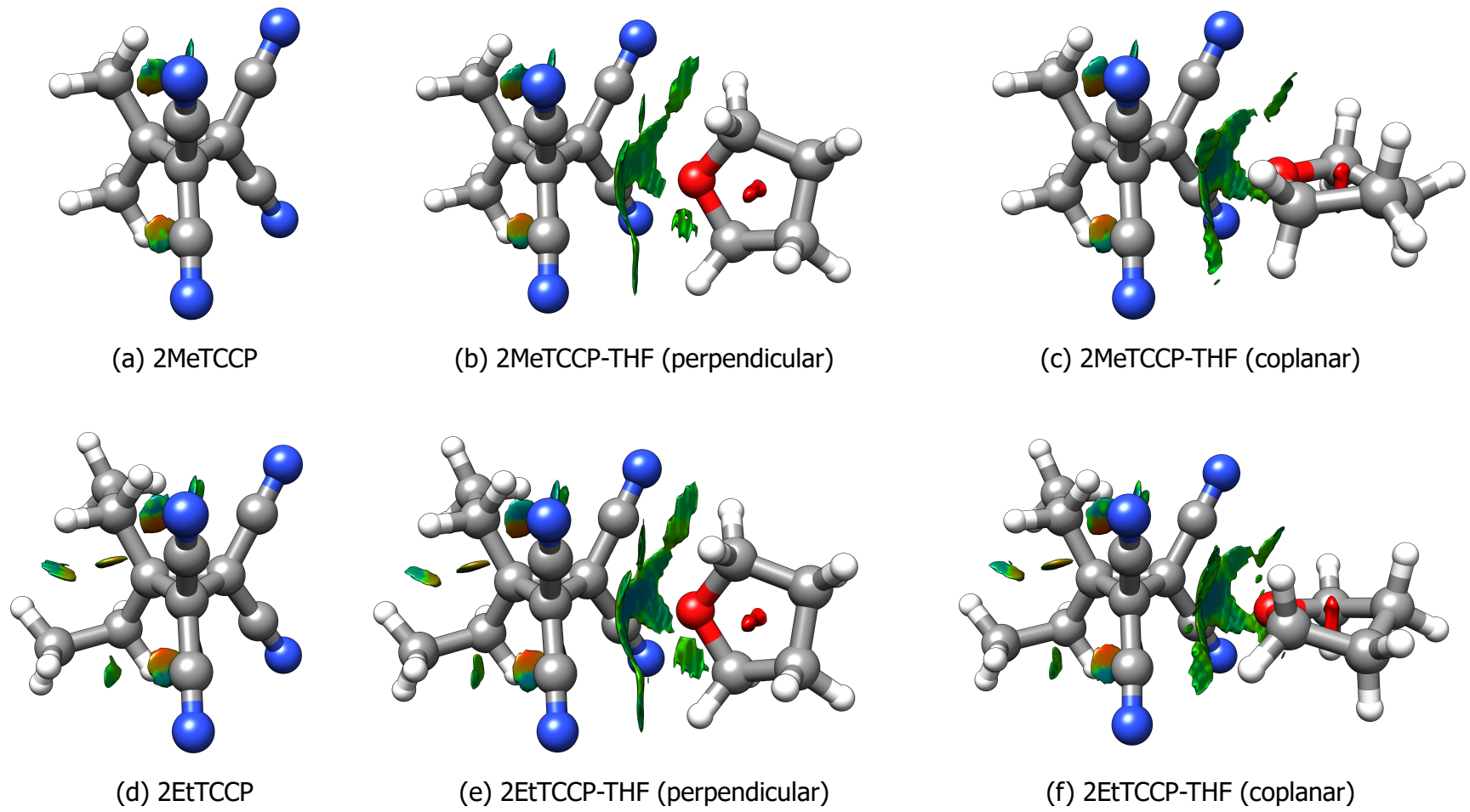

Figure 4. *NCI analyses of all molecular species studied. Molecular structures of 2MeTCCP, 2EtTCCP, 2MeTCCP-THF and 2EtTCCP (both configurations) as determined using B3LYP-D3BJ/def2-TZVP. Relevant intramolecular and intermolecular interactions are depicted and the NCI surfaces plotted (s=0.5 a.u.) and colored based on $sgn(\lambda_2)\rho$ with pure blue corresponding to -0.02 a.u. (or lower) and pure red to +0.02 a.u. (or higher).*

Fig. 3e), while the tetrel bond contributes to points in the -0.012 a.u. to -0.007 a.u. range (in purple, Fig. 3e).
- **Coplanar Configuration:** Here, the interaction between the H atoms of THF and the CN groups contributes to points in the range of -0.004 a.u. to -0.002 a.u. (in pink, Fig. 3f), while the tetrel bond contributes to points in the range of -0.011 a.u. to -0.0055 a.u (in purple, Fig. 3f). In general, we see that when going from the perpendicular to the coplanar configuration, the intermolecular interactions of 2EtTCCP-THF tend to move to regions with less negative values of $\mathrm{sgn}(\lambda_2)\rho$, and so, are weaker in the coplanar configuration than in the perpendicular configuration, in agreement with the SAPT calculations.

3.3. Symmetry Adapted Perturbation Theory

Symmetry adapted perturbation theory (SAPT) allows us to break down the interaction energy of a complex into several components and extract their physical meaning. The overall interaction energy is thus broken up into electrostatic, induction, dispersion, and exchange contributions, with the first three generally being attractive and the last repulsive.

To better understand the importance of these contributions to the interaction energy of the 2MeTCCP and 2EtTCCP complexes with THF, we ran SAPT calculations using Psi4 version 1.9.1 at the SAPT2+3 level and with the jun-cc-pVTZ basis set, a truncated form of the aug-cc-pVTZ basis set. The results are displayed below in Table 7.

For 2MeTCCP, the attractive terms (electrostatic, dispersion, and induction) become more negative going from perpendicular to coplanar docking, which would generally result in a more stable configuration. However, the exchange term is much greater for the coplanar geometry than that of the perpendicular. Consequently, the improvement in the attractive terms is balanced by the considerable increase in the repulsion term, rendering the perpendicular form the most stable one.

For 2EtTCCP, the attractive terms (electrostatic, dispersion, and induction) become less negative going from perpendicular to coplanar, a trend that is opposite to that observed for 2MeTCCP. There is

also a decrease in the repulsive exchange term, but one which is much smaller than the adjustment for 2MeTCCP and which does not compensate the change in the attractive terms. As such, the coplanar configuration remains less stable. The changes in the magnitudes of the relative components result in a final energy which agrees with the energy ordering obtained with DFT.

Table 7. *Results of energy decomposition from SAPT calculations for the perpendicular and coplanar configurations of the 2MeTCCP-THF and 2EtTCCP-THF complexes. The percentages presented are relative to the total attractive component of the interaction (electrostatics + induction + dispersion).*

Energy /kJ/mol	2MeTCCP-THF				2EtTCCP-THF			
	Perpendicular		Coplanar		Perpendicular		Coplanar	
Electrostatics	-32.67	45.22%	-38.36	42.88%	-46.71	45.04%	-37.94	42.63%
Exchange	26.69	36.94%	43.96	49.13%	53.02	51.13%	44.21	49.66%
Induction	-7.64	10.57%	-10.04	11.23%	-11.92	11.50%	-10.00	11.23%
Dispersion	-31.94	44.21%	-41.07	45.90%	-45.06	43.46%	-41.85	46.61%
Total	-45.56		-45.51		-50.67		-45.58	

4. Rotational Transitions

4.1. 3,3-Dimethyl-1,1,2,2-tetracyanocyclopropane

Table 8: Transition frequencies for 3,3-dimethyl-1,1,2,2-tetracyanocyclopropane. J is the rotational angular momentum quantum number, K_a and K_c are the projections of J onto the principal axes at the prolate and oblate symmetric top limits, respectively, and $I_{7,9}$, F_1, $I_{8,10}$ and F_2 are angular momentum quantum numbers obtained using the following coupling schemes between the rotational angular momentum and the nuclear spins of the four nitrogen nuclei (I(^{14}N) = 1): $\mathbf{I_{7,9}} = \mathbf{I_{N7}} + \mathbf{I_{N9}}$, $\mathbf{F_1} = \mathbf{J} + \mathbf{I_{7,9}}$, $\mathbf{I_{8,10}} = \mathbf{I_{N8}} + \mathbf{I_{N10}}$ and $\mathbf{F_2} = \mathbf{F_1} + \mathbf{I_{8,10}}$

Observed Freq. /MHz	Calculated Freq. /MHz	Difference / MHz	J	K_a	K_c	$I_{7,9}$	F_1	$I_{8,10}$	F_2	J'	K'_a	K'_c	$I'_{7,9}$	F'_1	$I'_{8,10}$	F'_2
3478.5942	3478.5876	0.0066	2	1	1	3	4	5	6	1	0	1	2	3	4	5
3478.7431	3478.7496	-0.0065	2	1	1	3	3	4	5	1	0	1	2	2	3	4
3478.7431	3478.7496	-0.0065	2	1	1	3	4	5	5	1	0	1	2	3	4	4
3478.7431	3478.7496	-0.0065	2	1	1	3	4	4	5	1	0	1	2	3	3	4
3478.7431	3478.7496	-0.0065	2	1	1	3	4	4	5	1	0	1	2	3	4	4
3478.7431	3478.7496	-0.0065	2	1	1	3	4	5	5	1	0	1	2	3	3	4
3478.7431	3478.7496	-0.0065	2	1	1	3	3	2	3	1	0	1	1	0	1	2
3478.7431	3478.7496	-0.0065	2	1	1	3	4	4	3	1	0	1	2	3	3	2
3478.7431	3478.7496	-0.0065	2	1	1	3	3	4	3	1	0	1	1	0	1	2
3478.9199	3478.9116	0.0083	2	1	1	2	3	4	4	1	0	1	2	2	3	3
3478.9199	3478.9116	0.0083	2	1	1	3	4	4	4	1	0	1	2	3	3	3
3617.8782	3617.8695	0.0087	2	2	0	3	4	5	5	1	1	0	2	3	4	4
3617.8782	3617.8695	0.0087	2	2	0	3	3	4	5	1	1	0	2	2	3	4
3617.8782	3617.8695	0.0087	2	2	0	3	4	4	5	1	1	0	2	3	3	4
3617.8782	3617.9020	-0.0238	2	2	0	2	3	4	5	1	1	0	1	2	3	4
3617.8782	3617.8694	0.0088	2	2	0	3	4	4	3	1	1	0	2	2	3	2

3617.8782	3617.8694	0.0088	2	0	2	3	3	2	3	1	1	0	2	2	1	2
3617.8782	3617.8694	0.0088	2	2	0	2	3	4	3	1	1	0	2	3	3	2
3617.8782	3617.8537	0.0245	2	2	0	1	2	3	3	1	1	0	0	1	1	2
3617.8782	3617.8537	0.0245	2	2	0	1	2	2	3	1	1	0	2	3	2	2
3682.7650	3682.7644	0.0006	2	2	1	3	4	3	4	1	1	1	2	3	2	3
3682.7650	3682.7644	0.0006	2	2	1	3	2	3	4	1	1	1	2	1	2	3
3682.7650	3682.7644	0.0006	2	2	1	3	4	5	4	1	1	1	2	3	4	3
3682.8731	3682.8684	0.0047	2	2	1	3	4	5	6	1	1	1	2	3	4	5
3683.2431	3683.2338	0.0093	2	2	1	3	2	3	3	1	1	1	0	1	1	2
3683.2431	3683.2539	-0.0108	2	2	1	3	4	4	5	1	1	1	2	3	3	4
3683.2431	3683.2539	-0.0108	2	2	1	2	3	4	5	1	1	1	2	2	3	4
3683.2431	3683.2539	-0.0108	2	2	1	3	4	5	5	1	1	1	2	2	3	4
3683.2431	3683.2539	-0.0108	2	2	1	3	4	5	5	1	1	1	2	3	4	4
3683.2431	3683.2539	-0.0108	2	2	1	2	3	4	5	1	1	1	2	3	4	4
3683.2431	3683.2538	-0.0107	2	2	1	3	3	2	3	1	1	1	2	2	1	2
3683.2431	3683.2538	-0.0107	2	2	1	3	3	4	3	1	1	1	2	2	3	2
3683.2431	3683.2538	-0.0107	2	2	1	3	4	4	3	1	1	1	2	3	3	2
3683.2431	3683.2338	0.0093	2	2	1	1	2	2	3	1	1	1	2	1	1	2
3683.8611	3683.8625	-0.0014	2	2	1	2	3	3	4	1	1	1	1	2	2	3
3683.8611	3683.8625	-0.0014	2	2	1	3	3	3	4	1	1	1	2	2	2	3
3683.8611	3683.8625	-0.0014	2	2	1	2	3	4	4	1	1	1	1	2	3	3
5246.9250	5246.9054	0.0196	3	2	1	2	3	4	5	2	1	1	1	2	3	4
5246.9250	5246.8995	0.0255	3	2	1	2	3	4	3	2	1	1	3	2	3	2
5246.9250	5246.9535	-0.0285	3	2	1	4	3	4	3	2	1	1	1	1	1	2
5246.9250	5246.9535	-0.0285	3	2	1	4	3	2	3	2	1	1	1	2	3	2
5246.9250	5246.8743	0.0507	3	0	3	3	4	5	6	2	1	1	3	4	5	6
5246.9250	5246.8995	0.0255	3	0	3	2	3	2	3	2	1	1	3	2	1	2
5246.9250	5246.9535	-0.0285	3	2	1	4	3	2	3	2	1	1	1	1	1	2
5246.9250	5246.8995	0.0255	3	2	1	4	5	4	3	2	1	1	3	4	3	2
5246.9250	5246.9535	-0.0285	3	2	1	4	3	4	3	2	1	1	1	2	3	2
5247.0029	5247.0320	-0.0291	3	2	1	2	3	4	4	2	1	1	3	4	3	3
5247.0029	5246.9802	0.0227	3	2	1	4	3	3	4	2	1	1	3	2	2	3
5247.0029	5246.9911	0.0118	3	2	1	2	2	3	4	2	1	1	1	1	2	3
5247.0029	5246.9802	0.0227	3	2	1	2	3	3	4	2	1	1	1	2	2	3
5247.0029	5246.9802	0.0227	3	2	1	4	5	4	4	2	1	1	3	2	3	3
5247.0029	5247.0320	-0.0291	3	2	1	4	3	4	4	2	1	1	1	2	3	3
5247.0029	5247.0046	-0.0017	3	2	1	4	5	4	5	2	1	1	3	4	3	4
5247.0029	5247.0046	-0.0017	3	0	3	2	3	4	5	2	1	1	3	4	5	4
5247.0029	5247.0046	-0.0017	3	2	1	4	5	6	5	2	1	1	3	4	5	4
5247.0029	5247.0046	-0.0017	3	2	1	4	5	4	5	2	1	1	3	2	3	4
5247.0045	5247.0585	-0.0540	3	2	1	4	5	6	7	2	1	1	3	4	5	6
5247.1947	5247.1910	0.0037	3	2	1	4	5	6	6	2	1	1	3	4	5	5
5247.1947	5247.2195	-0.0248	3	1	2	2	3	4	3	2	2	0	1	2	3	2
5247.1947	5247.1910	0.0037	3	2	1	4	4	5	6	2	1	1	3	3	4	5
5247.1947	5247.1910	0.0037	3	2	1	3	4	5	6	2	1	1	3	4	4	5
5247.1947	5247.2024	-0.0077	3	3	0	2	3	4	5	2	0	2	3	2	3	4
5247.1947	5247.1909	0.0038	3	2	1	4	4	3	4	2	1	1	3	3	2	3
5247.1947	5247.1909	0.0038	3	2	1	4	5	5	4	2	1	1	3	4	4	3
5247.1947	5247.1909	0.0038	3	2	1	4	4	5	4	2	1	1	3	3	4	3
5247.3875	5247.3485	0.0390	3	1	2	2	3	3	4	2	0	2	1	2	3	3
5247.3875	5247.4029	-0.0154	3	2	1	4	4	4	3	2	1	1	2	3	2	2
5247.3875	5247.4074	-0.0199	3	0	3	3	3	4	5	2	1	1	2	3	4	5
5247.3875	5247.4029	-0.0154	3	2	1	4	4	4	3	2	1	1	3	3	3	2

5247.3875	5247.3485	0.0390	3	3	0	4	3	3	4	2	0	2	3	2	3	3
5247.3875	5247.4029	-0.0154	3	2	1	3	4	4	3	6	1	5	5	4	3	2
5247.3875	5247.3587	0.0288	3	1	2	4	3	3	2	2	0	2	1	0	1	1
5247.3875	5247.3844	0.0031	3	1	2	4	5	6	5	2	0	2	3	4	3	4
5247.3875	5247.3844	0.0031	3	1	2	4	5	4	5	2	0	2	3	4	5	4
5247.3875	5247.3844	0.0031	3	1	2	2	3	4	5	2	0	2	1	2	3	4
5247.4558	5247.4569	-0.0011	3	2	1	3	4	4	5	2	1	1	2	3	4	4
5247.4558	5247.4690	-0.0132	3	2	1	3	4	3	3	2	1	1	2	2	3	2
5247.4558	5247.4904	-0.0346	3	2	1	3	2	3	4	2	1	1	2	1	2	3
5247.4558	5247.4904	-0.0346	3	2	1	3	4	3	4	2	1	1	2	3	2	3
5247.4558	5247.4569	-0.0011	3	2	1	4	4	4	5	2	1	1	2	3	4	4
5247.4558	5247.4569	-0.0011	3	2	1	3	3	4	5	2	1	1	3	4	4	4
5247.4558	5247.4904	-0.0346	3	2	1	3	4	5	4	2	1	1	2	3	4	3
5247.4558	5247.4569	-0.0011	3	2	1	4	4	4	5	2	1	1	3	3	3	4
5247.4558	5247.5086	-0.0528	3	0	3	3	4	5	6	2	1	1	2	3	4	5
5247.4558	5247.4612	-0.0054	3	1	2	4	5	6	7	2	0	2	3	4	5	6
5247.6006	5247.5900	0.0106	3	1	2	4	5	6	6	2	0	2	3	4	5	5
5247.6006	5247.6004	0.0002	3	2	1	3	3	2	2	2	1	1	2	2	1	1
5247.6006	5247.6339	-0.0333	3	0	3	3	3	4	5	2	1	1	2	3	3	4
5247.6006	5247.5900	0.0106	3	1	2	4	5	6	6	2	0	2	3	3	4	5
5247.6006	5247.5900	0.0106	3	1	2	4	5	5	4	2	0	2	3	4	4	3
5247.6006	5247.5900	0.0106	3	3	0	3	4	5	6	2	0	2	3	4	4	5
5247.6006	5247.5990	0.0016	3	2	1	3	3	4	3	2	1	1	2	3	3	2
5247.6006	5247.5900	0.0106	3	1	2	4	5	5	6	2	0	2	3	4	4	5
5247.6006	5247.5990	0.0016	3	2	1	3	3	2	3	2	1	1	2	2	1	2
5247.6006	5247.5990	0.0016	3	2	1	3	2	3	3	2	1	1	2	1	1	2
5247.7203	5247.7528	-0.0325	3	2	1	2	2	3	3	2	1	1	2	2	3	3
5247.7203	5247.7046	0.0157	3	2	1	3	3	3	3	2	1	1	2	2	2	2
5247.7203	5247.6755	0.0448	3	1	2	3	3	2	3	2	0	2	2	1	2	3
5247.7203	5247.7189	0.0014	3	1	2	3	4	4	5	2	0	2	3	4	4	4
5247.7203	5247.7189	0.0014	3	1	2	3	4	5	5	2	2	0	3	3	4	4
5247.7203	5247.6755	0.0448	3	1	2	3	2	2	3	2	0	2	2	3	2	3
5247.7203	5247.7189	0.0014	3	1	2	3	4	4	5	2	2	0	3	3	4	4
5247.7203	5247.7189	0.0014	3	1	2	3	4	5	5	2	0	2	3	4	4	4
5247.9375	5247.9167	0.0208	3	1	2	4	4	4	5	2	0	2	3	3	3	4
5247.9375	5247.9167	0.0208	3	1	2	4	5	5	5	2	0	2	2	3	3	4
5247.9375	5247.9668	-0.0293	3	1	2	3	3	4	4	2	0	2	2	2	3	3
5247.9375	5247.9668	-0.0293	3	1	2	3	4	4	4	2	0	2	2	3	3	3
5247.9375	5247.9167	0.0208	3	1	2	4	4	5	5	2	0	2	3	3	4	4
5247.9375	5247.9668	-0.0293	3	1	2	4	4	4	4	2	0	2	3	3	3	3
5247.9375	5247.9085	0.0290	3	1	2	3	3	3	4	2	0	2	2	2	3	4
5247.9375	5247.9631	-0.0256	3	1	2	4	4	4	3	2	0	2	2	3	2	2
5247.9375	5247.9631	-0.0256	3	1	2	4	4	3	3	2	0	2	2	1	2	2
5247.9750	5247.9890	-0.0140	3	2	1	2	2	2	2	2	1	1	2	2	2	2
5370.7548	5370.7641	-0.0093	3	2	2	2	3	2	2	2	1	2	1	2	1	1
5370.7548	5370.7641	-0.0093	3	2	2	4	3	3	2	2	1	2	1	2	1	1
5370.7548	5370.7639	-0.0091	3	2	2	2	1	2	1	2	1	2	1	2	1	1
5370.7548	5370.7641	-0.0093	3	2	2	4	3	3	2	2	1	2	1	0	1	1
5370.7548	5370.7639	-0.0091	3	2	2	2	3	2	1	2	1	2	1	0	1	1
5370.7548	5370.7641	-0.0093	3	2	2	4	3	2	2	2	1	2	3	2	1	1
5370.7548	5370.7641	-0.0093	3	2	2	2	3	2	2	2	1	2	1	0	1	1
5370.7548	5370.7639	-0.0091	3	2	2	2	3	2	1	2	1	2	1	2	1	1
5370.7548	5370.7639	-0.0091	3	2	2	2	1	2	1	2	1	2	1	0	1	1

5370.7548	5370.7639	-0.0091	3	2	2	4	3	2	1	2	1	2	3	2	1	1
5370.8868	5370.9223	-0.0355	3	2	2	4	3	4	5	2	1	2	1	2	3	4
5370.8868	5370.8907	-0.0039	3	2	2	4	3	2	3	2	1	2	3	2	1	2
5370.8868	5370.8907	-0.0039	3	2	2	2	3	2	3	2	1	2	3	2	3	2
5370.8868	5370.8907	-0.0039	3	2	2	4	5	4	3	2	1	2	3	4	3	2
5370.9864	5371.0145	-0.0281	3	2	2	2	3	4	3	2	1	2	1	1	1	2
5370.9864	5370.9880	-0.0016	3	2	2	2	1	2	3	2	1	2	1	0	1	2
5370.9864	5370.9880	-0.0016	3	2	2	4	3	4	3	2	1	2	1	2	3	2
5370.9864	5371.0152	-0.0288	3	2	2	2	2	3	3	2	1	2	1	1	1	2
5371.1070	5371.0999	0.0071	3	2	2	4	5	4	5	2	1	2	3	2	3	4
5371.1070	5371.1284	-0.0214	3	2	2	2	3	3	4	2	1	2	1	2	3	3
5371.1070	5371.1284	-0.0214	3	2	2	4	3	3	4	2	1	2	3	3	2	3
5371.1070	5371.0999	0.0071	3	2	2	2	3	4	5	2	1	2	3	2	3	4
5371.1070	5371.0999	0.0071	3	2	2	2	3	4	5	2	1	2	3	4	3	4
5371.1070	5371.0999	0.0071	3	2	2	4	5	4	5	2	1	2	3	4	3	4
5371.1070	5371.0999	0.0071	3	2	2	4	5	6	5	2	1	2	3	4	5	4
5371.1070	5371.1284	-0.0214	3	2	2	4	5	4	4	2	1	2	3	4	3	3
5371.1934	5371.2121	-0.0187	3	2	2	3	4	3	4	2	1	2	1	1	2	3
5371.1934	5371.2130	-0.0196	3	2	2	4	5	6	7	2	1	2	3	4	5	6
5371.1934	5371.2129	-0.0195	3	2	2	4	3	4	4	2	1	2	3	2	3	3
5371.1934	5371.2129	-0.0195	3	2	2	2	2	3	4	2	1	2	1	2	2	3
5371.5319	5371.5351	-0.0032	3	2	2	2	3	4	4	2	1	2	3	4	4	3
5371.5319	5371.5351	-0.0032	3	2	2	4	5	6	6	2	1	2	3	3	4	5
5371.5319	5371.5351	-0.0032	3	2	2	4	4	3	4	2	1	2	3	3	4	3
5371.5319	5371.5351	-0.0032	3	2	2	4	5	5	4	2	1	2	2	1	2	3
5371.5319	5371.5351	-0.0032	3	2	2	4	5	5	6	2	1	2	3	4	5	5
5371.5319	5371.5351	-0.0032	3	2	2	4	4	5	6	2	1	2	3	3	4	5
5371.5319	5371.5351	-0.0032	3	2	2	4	5	5	6	2	1	2	3	4	4	5
5371.5319	5371.5351	-0.0032	3	2	2	4	4	3	4	2	1	2	2	1	2	3
5371.5319	5371.5351	-0.0032	3	2	2	4	5	6	6	2	1	2	3	4	4	5
5371.5319	5371.5351	-0.0032	3	2	2	4	4	5	6	2	1	2	3	4	5	5
5371.8502	5371.8572	-0.0070	3	2	2	4	4	4	5	2	1	2	3	3	4	4
5371.8502	5371.8572	-0.0070	3	2	2	4	5	5	5	2	1	2	3	3	4	4
5371.8502	5371.8572	-0.0070	3	2	2	4	4	4	5	2	1	2	3	4	4	4
5371.8502	5371.8572	-0.0070	3	2	2	4	5	5	5	2	1	2	3	4	4	4
5372.0159	5372.0182	-0.0023	3	2	2	3	4	3	3	2	1	2	3	3	2	2
5372.0159	5372.0182	-0.0023	3	2	2	4	4	3	3	2	1	2	2	3	2	2
5372.0159	5372.0180	-0.0021	3	2	2	3	4	3	2	2	1	2	2	3	2	1
5372.0159	5372.0180	-0.0021	3	2	2	3	2	1	2	2	1	2	2	1	2	1
5372.0159	5372.0182	-0.0023	3	2	2	4	4	3	3	2	1	2	3	3	2	2
5372.0159	5372.0182	-0.0023	3	2	2	2	3	3	3	2	1	2	3	3	3	2
5372.0159	5372.0182	-0.0023	3	2	2	3	4	3	3	2	1	2	2	3	2	2
5372.1352	5372.1242	0.0110	3	2	2	3	2	3	4	2	1	2	3	2	2	3
5372.1352	5372.1313	0.0039	3	2	2	3	4	5	5	2	1	2	2	3	4	4
5372.1352	5372.1242	0.0110	3	2	2	3	4	5	4	2	1	2	2	3	2	3
5372.1352	5372.1242	0.0110	3	2	2	4	4	5	4	2	1	2	2	3	4	3
5372.1352	5372.1313	0.0039	3	2	2	3	3	4	5	2	1	2	3	3	3	4
5372.1352	5372.1793	-0.0441	3	2	2	3	4	5	6	2	1	2	2	3	4	5
5372.1352	5372.1313	0.0039	3	2	2	4	4	5	5	2	1	2	2	3	3	4
5372.1352	5372.1356	-0.0004	3	2	2	4	4	4	3	2	1	2	2	1	2	2
5372.1352	5372.1356	-0.0004	3	2	2	3	3	4	3	2	1	2	2	2	3	2
5372.1352	5372.1313	0.0039	3	2	2	3	4	5	5	2	1	2	3	3	3	4
5372.3751	5372.3723	0.0028	3	2	2	4	4	4	4	2	1	2	3	3	3	3

5372.3751	5372.3723	0.0028	3	2	2	4	4	4	4	2	1	2	2	2	3	3
5372.3751	5372.3723	0.0028	3	2	2	3	3	4	4	2	1	2	2	2	3	3
5372.3751	5372.3723	0.0028	3	2	2	4	4	4	4	2	1	2	2	3	3	3
5372.3751	5372.3723	0.0028	3	2	2	3	4	4	4	2	1	2	2	2	3	3
5372.3751	5372.3942	-0.0191	3	2	2	3	2	2	1	2	1	2	2	1	1	0
5372.3751	5372.3723	0.0028	3	2	2	3	3	4	4	2	1	2	2	3	3	3
5372.3751	5372.3942	-0.0191	3	2	2	3	3	2	1	2	1	2	2	2	1	0
5372.3751	5372.3723	0.0028	3	2	2	3	4	4	4	2	1	2	3	3	3	3
5372.4309	5372.4295	0.0014	3	2	2	3	2	2	2	2	1	2	2	2	1	2
5372.4309	5372.4295	0.0014	3	2	2	3	3	2	2	2	1	2	2	1	1	2
5372.4309	5372.4297	0.0012	3	2	2	3	4	4	3	2	1	2	2	3	3	2
5372.4309	5372.4295	0.0014	3	2	2	3	3	3	2	2	1	2	2	3	3	2
5372.4309	5372.4297	0.0012	3	2	2	3	3	2	3	2	1	2	2	2	1	2
5372.4309	5372.4297	0.0012	3	2	2	3	2	2	3	2	1	2	2	1	1	2
5372.5183	5372.4946	0.0237	3	2	2	3	3	2	2	2	1	2	2	2	1	1
5372.5183	5372.4946	0.0237	3	2	2	3	3	3	2	2	1	2	2	2	2	1
5372.5183	5372.5235	-0.0052	3	2	2	3	4	4	5	2	1	2	2	2	3	4
5372.5183	5372.4946	0.0237	3	2	2	3	2	2	2	2	1	2	2	1	1	1
5372.5183	5372.5232	-0.0049	3	2	2	3	3	3	4	2	1	2	2	2	3	4
5495.2789	5495.2926	-0.0137	3	3	0	4	5	4	5	2	2	0	3	4	3	4
5495.2789	5495.2926	-0.0137	3	3	0	4	3	4	5	2	2	0	1	2	3	4
5495.2789	5495.2535	0.0254	3	3	0	2	3	4	3	2	2	0	3	2	3	2
5495.2789	5495.2535	0.0254	3	3	0	4	5	4	3	2	2	0	3	4	3	2
5495.2789	5495.3034	-0.0245	3	3	0	2	3	2	3	2	0	2	1	0	1	2
5495.2789	5495.3034	-0.0245	3	1	2	4	5	4	3	2	0	2	3	2	3	2
5495.2789	5495.2926	-0.0137	3	3	0	4	5	6	5	2	2	0	3	4	5	4
5495.2789	5495.3125	-0.0336	3	3	0	4	5	6	7	2	2	0	3	4	5	6
5495.2789	5495.3034	-0.0245	3	3	0	4	3	2	3	2	2	0	3	2	1	2
5495.5722	5495.5593	0.0129	3	1	2	3	2	3	4	2	2	0	2	3	2	3
5495.5722	5495.5593	0.0129	3	3	0	4	5	5	4	2	2	0	3	4	4	3
5495.5722	5495.5504	0.0218	3	3	0	4	3	4	4	2	2	0	3	2	3	3
5495.5722	5495.5594	0.0128	3	3	0	4	5	5	6	2	2	0	3	3	4	5
5495.5722	5495.5594	0.0128	3	3	0	4	5	5	6	2	2	0	3	4	4	5
5495.5722	5495.5504	0.0218	3	3	0	4	5	4	4	2	2	0	1	2	3	3
5495.5722	5495.5594	0.0128	3	3	0	4	5	6	6	2	2	0	3	4	5	5
5495.5722	5495.5594	0.0128	3	1	2	4	4	5	6	2	2	0	3	4	4	5
5495.5722	5495.5593	0.0129	3	1	2	4	4	5	4	2	2	0	2	3	4	3
5495.5722	5495.5772	-0.0050	3	3	0	4	4	5	6	2	2	0	2	3	4	5
5496.1219	5496.1132	0.0087	3	3	0	3	3	4	4	2	2	0	2	2	2	3
5496.1219	5496.1132	0.0087	3	3	0	3	4	4	4	2	2	0	2	3	3	3
5496.1219	5496.1648	-0.0429	3	3	0	3	3	2	2	2	2	0	2	2	1	1
5496.1219	5496.1132	0.0087	3	3	0	3	4	4	4	2	2	0	2	2	2	3
5496.1219	5496.1132	0.0087	3	3	0	4	4	4	4	2	2	0	3	3	3	3
5496.1219	5496.1077	0.0142	3	3	0	2	2	3	3	2	2	0	1	1	2	3
5496.1219	5496.1132	0.0087	3	3	0	3	3	4	4	2	2	0	2	3	3	3
5496.1224	5496.1648	-0.0424	3	3	0	3	3	3	2	2	2	0	2	2	1	1
5496.1224	5496.1648	-0.0424	3	3	0	3	3	3	2	2	2	0	2	2	2	1
5496.1224	5496.1648	-0.0424	3	3	0	3	3	2	2	2	2	0	2	2	2	1
5545.0534	5545.0492	0.0042	3	3	1	4	4	5	4	2	2	1	3	3	4	3
5545.0534	5545.0492	0.0042	3	3	1	4	5	5	4	2	2	1	1	2	2	3
5545.0534	5545.0492	0.0042	3	3	1	3	2	3	4	2	2	1	3	3	2	3
5545.0534	5545.0492	0.0042	3	1	3	4	4	5	6	2	2	1	3	3	4	5
5545.0534	5545.0492	0.0042	3	3	1	4	5	6	6	2	2	1	3	4	5	5

5545.0534	5545.0758	-0.0224	3	3	1	2	3	4	4	2	2	1	1	2	3	3
5545.0534	5545.0758	-0.0224	3	3	1	4	3	4	4	2	2	1	3	2	3	3
5545.0534	5545.0492	0.0042	3	1	3	4	4	5	6	2	2	1	3	4	5	5
5545.0534	5545.0492	0.0042	3	3	1	4	5	5	6	2	2	1	3	4	4	5
5545.0534	5545.0492	0.0042	3	3	1	4	5	6	6	2	2	1	3	3	4	5
5545.1804	5545.1790	0.0014	3	3	1	4	5	4	4	2	2	1	3	4	3	3
5545.1804	5545.1389	0.0415	3	3	1	4	4	3	2	2	2	1	3	3	2	1
5545.1804	5545.1389	0.0415	3	3	1	2	3	2	2	2	2	1	3	2	2	1
5545.1804	5545.1727	0.0077	3	3	1	2	1	2	3	2	2	1	1	2	1	2
5545.1804	5545.1565	0.0239	3	1	3	3	4	3	2	2	2	1	1	1	0	1
5545.1804	5545.1790	0.0014	3	3	1	2	3	3	4	2	2	1	3	4	4	3
5545.1804	5545.1790	0.0014	3	3	1	4	3	3	4	2	2	1	3	2	2	3
5545.1804	5545.1389	0.0415	3	3	1	2	1	1	2	2	2	1	1	2	2	1
5545.1804	5545.1743	0.0061	3	3	1	2	3	3	3	2	2	1	2	1	2	2
5545.1804	5545.1743	0.0061	3	3	1	3	2	3	3	2	2	1	2	3	2	2
5545.2864	5545.2764	0.0100	3	3	1	4	4	5	5	2	2	1	3	3	4	4
5545.2864	5545.2764	0.0100	3	3	1	4	5	5	5	2	2	1	3	4	4	4
5545.2864	5545.2718	0.0146	3	1	3	3	4	4	5	2	2	1	2	3	3	4
5545.2864	5545.2718	0.0146	3	3	1	3	3	4	5	2	2	1	3	3	3	4
5545.2864	5545.2631	0.0233	3	3	1	3	4	3	3	2	2	1	3	3	2	2
5545.2864	5545.2718	0.0146	3	3	1	3	3	4	5	2	2	1	2	3	4	4
5545.2864	5545.2975	-0.0111	3	3	1	3	2	2	3	2	2	1	2	1	1	2
5545.2864	5545.2718	0.0146	3	3	1	3	4	5	5	2	2	1	2	3	4	4
5545.2864	5545.2631	0.0233	3	3	1	4	4	4	3	2	2	1	3	3	3	2
5545.2864	5545.2631	0.0233	3	1	3	3	4	4	3	2	2	1	2	3	3	2
5545.5556	5545.5461	0.0095	3	1	3	3	4	4	4	2	2	1	2	3	3	3
5545.5556	5545.5348	0.0208	3	3	1	4	3	3	3	2	2	1	1	2	2	2
5545.5556	5545.5875	-0.0319	3	3	1	3	2	2	2	2	2	1	2	2	2	1
5545.5556	5545.5461	0.0095	3	3	1	3	4	4	4	2	2	1	3	3	3	3
5545.5556	5545.5348	0.0208	3	1	3	4	3	3	3	2	2	1	3	2	2	2
5545.5556	5545.5461	0.0095	3	3	1	3	4	4	4	2	2	1	2	2	3	3
5545.5556	5545.5461	0.0095	3	3	1	4	4	4	4	2	2	1	3	3	3	3
5545.5556	5545.5875	-0.0319	3	3	1	3	3	2	2	2	2	1	2	3	2	1
5545.5556	5545.5461	0.0095	3	3	1	4	4	4	4	2	2	1	2	2	3	3
5545.5556	5545.5348	0.0208	3	3	1	2	2	3	3	2	2	1	1	1	2	2
6746.5445	6746.5619	-0.0174	5	3	2	5	6	7	7	4	2	2	5	6	6	6
6746.5445	6746.5619	-0.0174	5	1	4	6	6	7	7	4	4	0	5	5	6	6
6746.5445	6746.5533	-0.0088	5	1	4	4	4	5	5	4	0	4	3	3	3	4
6746.5445	6746.5533	-0.0088	5	3	2	5	5	4	5	4	2	2	3	3	3	4
6746.5445	6746.5752	-0.0307	5	1	4	6	6	5	5	4	2	2	5	5	4	4
6746.5445	6746.5510	-0.0065	5	1	4	6	6	6	5	4	4	0	5	5	5	4
6746.5445	6746.5510	-0.0065	5	1	4	5	6	6	5	4	2	2	4	5	5	4
6746.5445	6746.5398	0.0047	5	1	4	6	7	7	7	4	2	2	5	5	6	6
6746.5445	6746.5510	-0.0065	5	1	4	5	6	5	5	4	4	0	4	5	4	4
6746.5445	6746.5752	-0.0307	5	1	4	6	5	5	5	4	2	2	5	4	4	4
6746.6949	6746.6660	0.0289	5	1	4	4	5	5	6	4	2	2	3	4	4	5
6746.6949	6746.7082	-0.0133	5	1	4	6	5	5	6	4	0	4	3	3	4	5
6746.6949	6746.7006	-0.0057	5	1	4	4	4	5	6	4	2	2	5	4	4	5
6746.6949	6746.6432	0.0517	5	1	4	5	6	7	8	4	2	2	4	5	6	7
6746.6949	6746.6613	0.0336	5	1	4	5	6	7	6	4	2	2	4	5	6	5
6746.6949	6746.7006	-0.0057	5	3	2	6	6	7	6	4	2	2	5	5	6	5
6746.6949	6746.7006	-0.0057	5	1	4	4	5	6	6	4	2	2	5	6	5	5
6746.6949	6746.6865	0.0084	5	1	4	6	7	8	8	4	2	2	5	6	7	7

6746.6949	6746.6865	0.0084	5	3	2	5	6	7	8	4	2	2	5	6	6	7
6746.6949	6746.7082	-0.0133	5	1	4	6	5	6	6	4	2	2	5	4	5	5
6746.8309	6746.8224	0.0085	5	1	4	6	7	6	7	4	2	2	5	6	5	6
6746.8309	6746.8224	0.0085	5	1	4	6	7	8	7	4	2	2	5	4	5	6
6746.8309	6746.8194	0.0115	5	3	2	4	5	6	7	4	2	2	3	4	5	6
6746.8309	6746.8224	0.0085	5	1	4	4	5	6	7	4	2	2	5	6	7	6
6746.8309	6746.8328	-0.0019	5	1	4	6	5	4	5	4	2	2	5	4	3	4
6746.8309	6746.8346	-0.0037	5	1	4	4	5	6	5	4	0	4	5	6	5	4
6746.8309	6746.8346	-0.0037	5	1	4	4	5	4	5	4	2	2	3	4	3	4
6746.8309	6746.8275	0.0034	5	1	4	4	3	4	5	4	4	0	3	2	3	4
6746.8309	6746.8328	-0.0019	5	1	4	6	7	6	5	4	2	2	5	6	5	4
6746.8309	6746.8328	-0.0019	5	3	2	4	5	4	5	4	2	2	5	4	5	4
6958.2258	6958.2526	-0.0268	4	2	2	4	5	6	7	3	1	2	4	5	6	7
6958.2258	6958.2262	-0.0004	4	2	2	4	5	4	5	3	1	2	4	5	4	5
6958.2258	6958.2214	0.0044	4	4	0	4	5	4	3	3	1	2	4	3	2	3
6958.2258	6958.2262	-0.0004	4	2	2	4	5	6	5	3	1	2	2	3	4	5
6958.2258	6958.2262	-0.0004	4	2	2	5	5	4	5	3	1	2	4	5	6	5
6958.2258	6958.2642	-0.0384	4	2	2	3	3	2	3	3	3	0	4	3	4	3
6958.2258	6958.2642	-0.0384	4	0	4	4	3	2	3	3	1	2	2	1	2	3
6958.2258	6958.2642	-0.0384	4	2	2	3	3	4	3	3	1	2	2	3	4	3
6958.2258	6958.2214	0.0044	4	2	2	4	3	4	3	3	1	2	4	3	4	3
6958.4656	6958.4715	-0.0059	4	2	2	3	2	2	2	3	1	2	2	1	1	1
6958.4656	6958.5199	-0.0543	4	4	0	4	5	4	4	3	1	2	2	3	4	4
6958.4656	6958.5199	-0.0543	4	4	0	5	5	5	4	3	3	0	3	4	5	4
6958.4656	6958.4838	-0.0182	4	2	2	3	2	3	3	3	1	2	2	3	2	3
6958.4656	6958.4625	0.0031	4	2	2	4	5	6	6	3	1	2	4	5	6	6
6958.4656	6958.4625	0.0031	4	2	2	5	5	6	6	3	3	0	3	4	5	6
6958.4656	6958.4625	0.0031	4	0	4	5	5	5	6	3	1	2	4	5	5	6
6958.4656	6958.5199	-0.0543	4	2	2	4	5	5	4	3	1	2	4	5	5	4
6958.5641	6958.5851	-0.0210	4	2	2	3	2	3	3	3	1	2	2	1	1	2
6958.5641	6958.5792	-0.0151	4	4	0	3	2	3	2	3	3	0	2	1	0	1
6958.5641	6958.5667	-0.0026	4	2	2	3	2	2	2	3	1	2	2	1	1	2
6958.5641	6958.5550	0.0091	4	2	2	3	2	1	1	3	3	0	2	1	0	1
6959.0304	6959.0412	-0.0108	4	2	2	4	5	6	7	3	1	2	3	4	5	6
6959.0304	6959.0003	0.0301	4	2	2	5	5	6	6	3	1	2	4	4	5	5
6959.0304	6959.0361	-0.0057	4	2	2	4	4	4	5	3	1	2	3	3	4	4
6959.0304	6959.0003	0.0301	4	0	4	5	5	5	6	3	1	2	4	4	4	5
6959.0304	6959.0361	-0.0057	4	2	2	5	5	5	5	3	1	2	4	4	4	4
6959.0304	6959.0361	-0.0057	4	2	2	4	5	5	5	3	1	2	3	4	4	4
6959.0304	6958.9971	0.0333	4	0	4	3	3	3	4	3	1	2	4	4	3	3
6959.0304	6958.9971	0.0333	4	2	2	3	3	3	4	3	1	2	4	4	4	3
6959.0304	6959.0247	0.0057	4	2	2	5	5	4	5	3	1	2	4	3	3	4
6959.0304	6958.9707	0.0597	4	2	2	4	5	5	4	3	3	0	3	4	4	3
6959.1150	6959.0629	0.0521	4	2	2	3	3	3	2	3	1	2	3	2	2	1
6959.1150	6959.1105	0.0045	4	4	0	4	4	5	6	3	1	2	3	3	4	5
6959.1150	6959.0629	0.0521	4	2	2	4	4	3	2	3	1	2	3	3	2	1
6959.1150	6959.1060	0.0090	4	2	2	4	4	4	4	3	1	2	3	3	3	3
6959.1150	6959.1125	0.0025	4	0	4	4	4	4	5	3	1	2	3	3	3	4
6959.1150	6959.0859	0.0291	4	0	4	4	4	3	4	3	1	2	3	3	2	3
6959.1150	6959.0859	0.0291	4	0	4	4	5	5	4	3	1	2	3	4	4	3
6959.1150	6959.0859	0.0291	4	2	2	4	3	4	4	3	1	2	3	2	2	3
6959.1150	6959.0715	0.0435	4	0	4	4	3	3	3	3	1	2	3	2	2	2
6959.1150	6959.0715	0.0435	4	2	2	4	4	4	3	3	1	2	3	3	3	2

7106.8441	7106.8177	0.0264	4	2	3	3	2	2	1	3	1	3	4	3	2	1
7106.8441	7106.8545	-0.0104	4	2	3	3	3	4	4	3	1	3	2	3	4	5
7106.8441	7106.8012	0.0429	4	2	3	4	3	2	2	3	3	1	2	3	3	2
7106.8441	7106.8012	0.0429	4	2	3	3	3	3	2	3	1	3	2	1	2	2
7106.8453	7106.8634	-0.0181	4	2	3	4	3	4	3	3	1	3	4	3	4	3
7106.8453	7106.8727	-0.0274	4	2	3	5	5	4	3	3	1	3	4	3	2	3
7106.8453	7106.8727	-0.0274	4	4	1	4	3	4	3	3	1	3	4	5	4	3
7106.8453	7106.8727	-0.0274	4	2	3	5	5	4	3	3	1	3	2	3	2	3
7106.8453	7106.8727	-0.0274	4	2	3	4	5	4	3	3	1	3	4	3	2	3
7106.8453	7106.8177	0.0276	4	2	3	3	3	2	1	3	1	3	2	3	2	1
7106.8453	7106.8634	-0.0181	4	2	3	4	3	2	3	3	1	3	2	3	4	3
7106.9093	7106.9295	-0.0202	4	2	3	4	5	6	5	3	1	3	4	5	4	5
7106.9093	7106.9295	-0.0202	4	2	3	4	5	4	5	3	1	3	4	5	6	5
7106.9093	7106.9295	-0.0202	4	2	3	4	3	4	5	3	1	3	4	3	4	5
7106.9093	7106.9388	-0.0295	4	2	3	3	2	1	1	3	3	1	2	1	0	1
7106.9875	7106.9599	0.0276	4	2	3	3	2	3	2	3	3	1	2	1	0	1
7106.9875	7107.0090	-0.0215	4	2	3	4	5	6	7	3	1	3	4	5	6	7
7106.9875	7106.9913	-0.0038	4	2	3	3	3	2	2	3	1	3	2	1	1	1
7106.9875	7107.0066	-0.0191	4	2	3	3	4	5	5	3	1	3	2	3	4	5
7106.9875	7106.9980	-0.0105	4	2	3	3	2	2	3	3	1	3	2	1	2	3
7106.9875	7107.0352	-0.0477	4	2	3	3	2	1	0	3	3	1	2	1	0	1
7107.1544	7107.1628	-0.0084	4	4	1	3	4	3	4	3	1	3	2	1	2	3
7107.1544	7107.1522	0.0022	4	2	3	3	2	2	3	3	1	3	2	2	1	2
7107.3010	7107.2800	0.0210	4	4	1	3	4	5	4	3	1	3	4	3	2	3
7107.3010	7107.2800	0.0210	4	2	3	3	4	3	4	3	1	3	4	5	4	3
7107.3010	7107.2512	0.0498	4	2	3	3	4	3	3	3	3	1	2	3	3	2
7107.3010	7107.2800	0.0210	4	2	3	5	6	5	4	3	1	3	2	3	2	3
7107.3010	7107.2512	0.0498	4	2	3	3	2	3	3	3	1	3	2	1	2	2
7107.3010	7107.2512	0.0498	4	2	3	3	4	3	3	3	1	3	4	3	2	2
7107.3010	7107.2800	0.0210	4	4	1	3	4	5	4	3	1	3	4	5	4	3
7107.3010	7107.2512	0.0498	4	4	1	5	4	3	3	3	3	1	2	3	3	2
7107.3010	7107.2800	0.0210	4	2	3	3	4	3	4	3	1	3	4	3	2	3
7107.3010	7107.2512	0.0498	4	4	1	5	4	3	3	3	1	3	4	3	2	2
7107.3014	7107.3332	-0.0318	4	2	3	3	4	5	6	3	1	3	2	3	4	5
7107.3014	7107.3284	-0.0270	4	2	3	3	4	5	4	3	1	3	4	3	4	3
7107.3014	7107.3284	-0.0270	4	2	3	3	2	3	4	3	1	3	2	3	4	3
7107.4002	7107.4177	-0.0175	4	2	3	5	4	5	6	3	1	3	4	3	4	5
7107.4002	7107.4177	-0.0175	4	2	3	5	6	5	6	3	1	3	4	5	4	5
7107.4002	7107.4177	-0.0175	4	2	3	5	6	7	6	3	1	3	4	5	6	5
7107.4002	7107.4177	-0.0175	4	2	3	5	6	5	6	3	1	3	4	5	6	5
7107.4002	7107.4177	-0.0175	4	2	3	5	6	7	6	3	1	3	4	3	4	5
7107.4002	7107.4177	-0.0175	4	2	3	5	4	5	6	3	1	3	4	5	4	5
7107.5691	7107.5484	0.0207	4	4	1	5	4	4	5	3	1	3	2	3	4	4
7107.5691	7107.5484	0.0207	4	4	1	5	4	4	5	3	1	3	4	3	4	4
7107.5691	7107.5484	0.0207	4	4	1	3	4	4	5	3	1	3	2	3	4	4
7107.5691	7107.4999	0.0692	4	2	3	5	6	7	8	3	1	3	4	5	6	7
7107.5691	7107.5226	0.0465	4	2	3	3	4	4	5	3	1	3	2	3	3	4
7107.5691	7107.5226	0.0465	4	2	3	5	4	4	5	3	1	3	4	3	3	4
7107.5691	7107.5226	0.0465	4	2	3	5	6	5	5	3	1	3	4	5	4	4
7107.5691	7107.5484	0.0207	4	4	1	3	4	4	5	3	1	3	4	3	4	4
7107.6543	7107.6360	0.0183	4	2	3	5	4	4	4	3	1	3	2	3	3	3
7107.6543	7107.6360	0.0183	4	4	1	5	5	4	4	3	3	1	4	4	3	3
7107.6543	7107.6360	0.0183	4	2	3	3	4	4	4	3	1	3	2	2	3	3

7107.6543	7107.6073	0.0470	4	2	3	3	3	2	3	3	1	3	4	4	3	2
7107.6543	7107.6073	0.0470	4	2	3	5	4	3	3	3	1	3	2	1	1	2
7107.6543	7107.6073	0.0470	4	2	3	3	4	4	3	3	1	3	4	3	3	2
7107.6543	7107.6226	0.0317	4	2	3	3	3	3	3	3	1	3	2	2	2	2
7107.6543	7107.6073	0.0470	4	2	3	3	3	2	3	3	1	3	2	1	1	2
7107.6543	7107.6073	0.0470	4	2	3	5	4	3	3	3	1	3	4	4	3	2
7107.7599	7107.7683	-0.0084	4	2	3	5	5	6	5	3	1	3	4	4	5	4
7107.7599	7107.7683	-0.0084	4	2	3	5	6	6	5	3	1	3	4	5	5	4
7107.7599	7107.7683	-0.0084	4	2	3	5	6	6	7	3	1	3	4	5	5	6
7107.7599	7107.7683	-0.0084	4	2	3	5	5	6	7	3	1	3	4	5	6	6
7107.7599	7107.7683	-0.0084	4	2	3	5	6	7	7	3	3	1	3	4	5	6
7107.7599	7107.7683	-0.0084	4	2	3	5	5	4	5	3	1	3	4	4	3	4
7107.7599	7107.7683	-0.0084	4	2	3	5	5	6	5	3	1	3	4	5	5	4
7107.7599	7107.7683	-0.0084	4	2	3	5	6	6	5	3	1	3	4	4	5	4
7107.8280	7107.8310	-0.0030	4	2	3	5	5	4	3	3	3	1	2	2	3	2
7107.8280	7107.8310	-0.0030	4	4	1	4	3	4	3	3	3	1	3	2	3	2
7107.8280	7107.8310	-0.0030	4	2	3	4	5	4	3	3	1	3	2	3	3	2
7107.8280	7107.8283	-0.0003	4	2	3	3	2	2	2	3	1	3	2	1	0	1
7107.8280	7107.8525	-0.0245	4	2	3	4	4	5	4	3	1	3	3	4	5	4
7107.8280	7107.8525	-0.0245	4	2	3	4	4	3	4	3	1	3	3	4	3	4
7107.8280	7107.8283	-0.0003	4	2	3	4	3	2	2	3	1	3	3	2	1	1
7107.8280	7107.8283	-0.0003	4	2	3	3	3	3	2	3	1	3	2	2	1	1
7107.8280	7107.8525	-0.0245	4	2	3	4	3	4	4	3	1	3	3	2	3	4
7107.8280	7107.8310	-0.0030	4	2	3	4	5	4	3	3	3	1	3	2	3	2
7108.0345	7108.0367	-0.0022	4	4	1	4	4	5	6	3	3	1	4	4	4	5
7108.0345	7108.0367	-0.0022	4	4	1	5	6	6	6	3	1	3	4	5	5	5
7108.0345	7108.0312	0.0033	4	2	3	4	3	2	3	3	1	3	2	2	3	2
7108.0345	7108.0312	0.0033	4	2	3	4	3	4	3	3	1	3	3	2	3	2
7108.1464	7108.1437	0.0027	4	2	3	4	3	4	5	3	1	3	3	2	3	4
7108.1464	7108.1437	0.0027	4	2	3	4	5	4	5	3	1	3	3	4	3	4
7108.1464	7108.1466	-0.0002	4	2	3	4	3	3	4	3	1	3	4	4	4	3
7108.1464	7108.1437	0.0027	4	2	3	4	5	6	5	3	1	3	3	4	5	4
7108.1464	7108.1466	-0.0002	4	4	1	4	4	5	4	3	1	3	4	4	3	3
7108.1464	7108.1466	-0.0002	4	2	3	4	5	4	4	3	3	1	3	3	4	3
7108.1464	7108.1466	-0.0002	4	2	3	4	5	4	4	3	1	3	4	4	3	3
7108.1477	7108.2289	-0.0812	4	2	3	5	5	6	6	3	1	3	4	4	4	5
7108.1477	7108.2002	-0.0525	4	2	3	5	5	5	4	3	1	3	3	4	3	3
7108.1477	7108.2002	-0.0525	4	2	3	5	5	4	4	3	1	3	2	2	2	3
7108.1477	7108.2081	-0.0604	4	2	3	4	5	6	7	3	1	3	3	4	5	6
7108.1477	7108.2289	-0.0812	4	2	3	5	6	6	6	3	1	3	3	4	5	5
7108.2283	7108.2289	-0.0006	4	2	3	5	5	6	6	3	1	3	4	4	5	5
7108.2283	7108.2289	-0.0006	4	2	3	5	5	5	6	3	1	3	4	4	5	5
7108.2283	7108.2289	-0.0006	4	2	3	5	5	5	6	3	1	3	4	4	4	5
7108.2283	7108.2289	-0.0006	4	2	3	5	5	5	6	3	1	3	3	4	5	5
7108.2283	7108.2289	-0.0006	4	2	3	5	6	6	6	3	1	3	4	4	5	5
7108.3563	7108.3496	0.0067	4	2	3	4	4	4	5	3	1	3	3	3	4	5
7108.3563	7108.4052	-0.0489	4	4	1	5	4	4	5	3	3	1	4	4	4	5
7108.3563	7108.4052	-0.0489	4	4	1	3	4	4	5	3	1	3	4	5	5	5
7108.3563	7108.3585	-0.0022	4	2	3	4	4	3	2	3	1	3	3	3	2	1
7108.3563	7108.3585	-0.0022	4	2	3	4	3	3	2	3	1	3	3	2	2	1
7108.4566	7108.4533	0.0033	4	2	3	4	3	4	4	3	1	3	3	2	3	3
7108.4566	7108.4542	0.0024	4	2	3	5	5	5	5	3	1	3	4	4	4	4
7108.4566	7108.4533	0.0033	4	2	3	4	4	5	4	3	1	3	3	3	4	3

7108.4566	7108.4542	0.0024	4	2	3	4	4	5	5	3	3	1	3	3	3	4
7108.4566	7108.4542	0.0024	4	2	3	5	5	5	5	3	1	3	3	3	4	4
7108.4566	7108.4542	0.0024	4	2	3	4	5	5	5	3	1	3	4	4	4	4
7108.4566	7108.4533	0.0033	4	2	3	4	4	3	4	3	1	3	3	3	2	3
7108.4566	7108.4542	0.0024	4	2	3	4	5	5	5	3	1	3	3	3	4	4
7108.4566	7108.4533	0.0033	4	2	3	4	4	3	4	3	1	3	3	3	4	3
7108.5552	7108.5490	0.0062	4	2	3	4	4	5	6	3	1	3	3	3	4	5
7108.5552	7108.5553	-0.0001	4	2	3	4	3	3	3	3	1	3	3	2	2	2
7108.5552	7108.5553	-0.0001	4	2	3	4	4	4	3	3	1	3	3	3	3	2
7108.5552	7108.5553	-0.0001	4	2	3	4	4	3	3	3	1	3	3	3	2	2
7108.6992	7108.7003	-0.0011	4	2	3	4	4	4	5	3	1	3	3	3	3	4
7108.7889	7108.7876	0.0013	4	2	3	4	4	4	4	3	1	3	3	3	3	3
7108.9836	7108.9814	0.0022	4	2	3	3	4	4	4	3	3	1	3	3	3	4
7108.9836	7109.0244	-0.0408	4	2	3	4	3	2	2	3	1	3	3	2	2	2
7108.9836	7108.9584	0.0252	4	2	3	3	3	4	4	3	1	3	3	3	4	5
7108.9836	7108.9814	0.0022	4	2	3	5	4	4	4	3	1	3	3	3	4	4
7108.9836	7108.9814	0.0022	4	4	1	5	5	4	4	3	1	3	4	4	4	4
7108.9836	7109.0244	-0.0408	4	2	3	3	2	2	2	3	1	3	3	3	2	2
7108.9836	7109.0244	-0.0408	4	2	3	3	3	3	2	3	1	3	3	3	3	2
7108.9836	7108.9814	0.0022	4	2	3	3	4	4	4	3	1	3	4	4	4	4
7108.9836	7108.9814	0.0022	4	4	1	5	5	4	4	3	3	1	3	3	3	4
7191.3580	7191.3574	0.0006	4	3	2	3	2	3	2	3	2	2	2	3	2	1
7191.3580	7191.3574	0.0006	4	3	2	3	4	3	2	3	2	2	4	3	2	1
7191.3580	7191.3574	0.0006	4	3	2	5	4	3	2	3	2	2	2	1	2	1
7191.3580	7191.3149	0.0431	4	1	4	3	2	3	2	3	2	2	2	1	0	1
7191.3580	7191.3144	0.0436	4	1	4	3	2	3	2	3	2	2	2	1	1	2
7191.3580	7191.3572	0.0008	4	3	2	3	4	3	2	3	2	2	4	3	2	2
7191.3580	7191.3330	0.0250	4	3	2	3	2	1	1	3	2	2	2	1	0	1
7191.3580	7191.3827	-0.0247	4	3	2	3	2	3	4	3	2	2	3	4	3	4
7191.3580	7191.3145	0.0435	4	1	4	3	2	3	2	3	2	2	2	1	1	1
7191.4194	7191.3832	0.0362	4	3	2	3	2	3	4	3	2	2	2	3	4	3
7191.4194	7191.4297	-0.0103	4	3	2	5	6	5	4	3	2	2	2	1	2	3
7191.4194	7191.4297	-0.0103	4	3	2	3	4	5	4	3	2	2	4	3	4	3
7191.4194	7191.4151	0.0043	4	3	2	5	4	3	4	3	2	2	4	3	2	3
7191.4194	7191.4151	0.0043	4	1	4	5	6	5	4	3	2	2	2	3	2	3
7191.4194	7191.4151	0.0043	4	3	2	5	4	5	4	3	2	2	2	3	2	3
7191.4194	7191.4151	0.0043	4	1	4	5	6	5	4	3	2	2	4	5	4	3
7191.4194	7191.4151	0.0043	4	3	2	5	4	5	4	3	2	2	4	5	4	3
7191.4703	7191.5199	-0.0496	4	3	2	5	6	7	8	3	2	2	4	5	6	7
7191.4703	7191.4515	0.0188	4	3	2	3	4	5	6	3	2	2	4	3	4	5
7191.4703	7191.4772	-0.0069	4	3	2	5	6	5	6	3	2	2	4	5	4	5
7191.4703	7191.4772	-0.0069	4	3	2	5	6	7	6	3	2	2	4	5	6	5
7191.4703	7191.4772	-0.0069	4	3	2	5	4	5	6	3	2	2	4	5	6	5
7191.4703	7191.5003	-0.0300	4	3	2	3	4	4	3	3	2	2	4	3	2	2
7191.4703	7191.5003	-0.0300	4	1	4	3	3	4	3	3	2	2	4	3	3	2
7191.6306	7191.6619	-0.0313	4	3	2	5	4	4	3	3	2	2	2	1	2	2
7191.6306	7191.6619	-0.0313	4	3	2	5	4	4	3	3	2	2	4	4	3	2
7191.6306	7191.6255	0.0051	4	1	4	5	6	6	5	3	2	2	4	5	4	4
7191.6306	7191.6255	0.0051	4	1	4	5	4	4	5	3	2	2	4	3	3	4
7191.6306	7191.6246	0.0060	4	3	2	3	4	5	5	3	2	2	4	3	4	4
7191.6306	7191.6246	0.0060	4	1	4	3	4	4	5	3	2	2	2	2	3	4
7191.6306	7191.6255	0.0051	4	1	4	5	6	6	5	3	2	2	2	3	3	4
7191.6306	7191.6255	0.0051	4	3	2	5	4	5	5	3	2	2	4	5	4	4

7191.6306	7191.6619	-0.0313	4	3	2	3	4	3	3	3	2	2	2	3	3	2
7191.6306	7191.6619	-0.0313	4	3	2	5	5	4	3	3	2	2	2	3	3	2
7191.7318	7191.7294	0.0024	4	3	2	5	6	6	7	3	2	2	4	5	5	6
7191.7318	7191.7293	0.0025	4	3	2	3	4	4	5	3	2	2	4	4	3	4
7191.7318	7191.7293	0.0025	4	3	2	4	3	4	5	3	2	2	2	3	4	4
7191.7318	7191.7293	0.0025	4	3	2	5	6	6	5	3	2	2	4	5	5	4
7191.7318	7191.7294	0.0024	4	3	2	5	5	6	7	3	2	2	4	5	6	6
7191.7318	7191.7294	0.0024	4	3	2	5	6	7	7	3	2	2	4	4	5	6
7191.7318	7191.7302	0.0016	4	3	2	3	3	4	5	3	2	2	3	4	3	4
7191.7318	7191.7294	0.0024	4	3	2	5	5	6	7	3	2	2	4	4	5	6
7191.7318	7191.7294	0.0024	4	3	2	5	6	7	7	3	2	2	4	5	6	6
7191.7318	7191.7802	-0.0484	4	3	2	4	3	3	4	3	2	2	2	2	2	3
7191.9177	7191.9388	-0.0211	4	3	2	4	5	6	7	3	2	2	3	4	5	6
7191.9177	7191.9389	-0.0212	4	3	2	5	6	6	6	3	2	2	4	5	5	5
7191.9177	7191.9389	-0.0212	4	3	2	5	5	5	6	3	2	2	4	4	4	5
7191.9177	7191.8937	0.0240	4	3	2	5	4	4	5	3	2	2	3	4	5	4
7191.9177	7191.8598	0.0579	4	3	2	3	3	4	4	3	2	2	2	2	3	3
7191.9177	7191.8937	0.0240	4	3	2	4	5	4	5	3	2	2	4	4	5	4
7191.9177	7191.8937	0.0240	4	3	2	4	5	6	5	3	2	2	4	4	5	4
7191.9177	7191.9115	0.0062	4	3	2	4	3	3	3	3	2	2	2	2	2	2
7191.9177	7191.8937	0.0240	4	3	2	5	4	4	5	3	2	2	3	2	3	4
7191.9177	7191.8937	0.0240	4	3	2	4	5	6	5	3	2	2	3	2	3	4
7192.0202	7191.9912	0.0290	4	3	2	5	5	5	4	3	2	2	2	3	3	3
7192.0202	7192.0340	-0.0138	4	3	2	5	5	6	6	3	2	2	3	3	4	5
7192.0202	7191.9912	0.0290	4	1	4	4	5	5	4	3	2	2	3	4	3	3
7192.0202	7192.0340	-0.0138	4	3	2	4	5	6	6	3	2	2	3	4	5	5
7192.0202	7192.0340	-0.0138	4	1	4	4	5	5	6	3	2	2	3	3	4	5
7192.0202	7192.0340	-0.0138	4	3	2	5	5	6	6	3	2	2	4	4	5	5
7192.0202	7191.9912	0.0290	4	3	2	5	5	4	4	3	2	2	4	4	3	3
7192.0202	7192.0065	0.0137	4	3	2	4	5	5	4	3	2	2	4	4	4	3
7192.0202	7192.0065	0.0137	4	3	2	4	5	4	4	3	2	2	3	3	4	3
7192.0202	7192.0340	-0.0138	4	3	2	4	5	6	6	3	2	2	4	4	5	5
7192.1392	7192.1422	-0.0030	4	3	2	4	4	3	4	3	2	2	3	3	2	3
7192.1392	7192.1422	-0.0030	4	3	2	4	4	5	4	3	2	2	3	4	4	3
7192.1392	7192.1422	-0.0030	4	3	2	4	3	4	4	3	2	2	3	2	2	3
7192.2396	7192.2093	0.0303	4	3	2	4	5	5	6	3	2	2	3	4	4	5
7192.2396	7192.2300	0.0096	4	3	2	5	5	5	5	3	2	2	3	3	4	4
7192.2396	7192.2300	0.0096	4	3	2	4	4	4	5	3	2	2	3	4	4	4
7192.2396	7192.2300	0.0096	4	3	2	4	5	5	5	3	2	2	3	4	4	4
7192.2396	7192.2300	0.0096	4	3	2	4	4	4	5	3	2	2	4	4	4	4
7192.2396	7192.2300	0.0096	4	3	2	5	5	5	5	3	2	2	4	4	4	4
7192.2396	7192.2300	0.0096	4	3	2	4	5	5	5	3	2	2	3	3	4	4
7192.2396	7192.2090	0.0306	4	3	2	4	5	5	6	3	2	2	3	4	5	6
7192.2750	7192.2843	-0.0093	4	3	2	4	4	3	3	3	2	2	3	3	2	2
7192.2750	7192.2843	-0.0093	4	3	2	4	4	4	3	3	2	2	3	3	3	2
7192.2750	7192.2843	-0.0093	4	3	2	3	3	3	3	3	2	2	3	2	2	2
7192.3764	7192.3794	-0.0030	4	3	2	4	4	5	5	3	2	2	3	3	3	4
7192.3764	7192.3792	-0.0028	4	3	2	4	4	5	5	3	2	2	3	4	4	5
7192.4837	7192.4910	-0.0073	4	3	2	4	4	4	4	3	2	2	3	3	3	3
7372.5910	7372.5905	0.0005	5	2	3	5	5	5	5	4	3	1	4	4	4	4
7372.8414	7372.8401	0.0013	5	4	1	5	5	5	6	4	3	1	4	4	4	5
7373.0131	7373.0147	-0.0016	5	2	3	5	5	5	4	4	3	1	4	4	4	3
7373.0131	7373.0147	-0.0016	5	2	3	5	5	4	4	4	3	1	4	4	3	3

7373.0131	7373.0147	-0.0016	5	2	3	5	4	4	4	4	3	1	4	3	3	3
7373.0131	7373.0147	-0.0016	5	2	3	5	5	4	4	4	3	1	4	3	3	3
7373.0131	7373.0147	-0.0016	5	2	3	5	4	4	4	4	3	1	4	4	3	3
7373.1119	7373.1081	0.0038	5	0	5	5	5	6	6	4	3	1	4	4	5	5
7373.1119	7373.1081	0.0038	5	2	3	5	6	6	6	4	3	1	4	5	5	5
7373.1119	7373.1081	0.0038	5	2	3	6	6	6	6	4	3	1	5	5	5	5
7373.1119	7373.1081	0.0038	5	2	3	5	6	6	6	4	3	1	5	5	5	5
7373.1119	7373.1081	0.0038	5	2	3	6	6	6	6	4	3	1	4	5	5	5
7373.2038	7373.2015	0.0023	5	2	3	5	5	6	7	4	3	1	4	5	6	6
7373.3268	7373.3255	0.0013	5	4	1	5	5	4	5	4	3	1	4	4	3	4
7373.3268	7373.3255	0.0013	5	0	5	5	6	6	5	4	3	1	4	5	5	4
7373.3268	7373.3255	0.0013	5	0	5	5	4	4	5	4	3	1	4	3	3	4
7373.3268	7373.3225	0.0043	5	2	3	5	5	6	7	4	3	1	4	5	6	7
7373.4500	7373.4506	-0.0006	5	2	3	5	5	4	3	4	3	1	4	4	3	2
7373.4500	7373.4506	-0.0006	5	0	5	5	4	3	3	4	3	1	4	3	3	2
7373.4500	7373.4506	-0.0006	5	0	5	5	4	3	3	4	3	1	4	4	3	2
7373.4500	7373.4506	-0.0006	5	2	3	5	5	4	3	4	3	1	4	3	3	2
7373.4500	7373.4366	0.0134	5	0	5	5	4	4	5	4	3	1	4	3	4	5
7373.4750	7373.5074	-0.0324	5	2	3	5	6	6	7	4	3	1	4	5	5	6
7373.4750	7373.5074	-0.0324	5	4	1	5	5	6	7	4	3	1	4	4	5	6
7373.4750	7373.5074	-0.0324	5	2	3	6	6	6	7	4	1	3	4	5	6	6
7373.4750	7373.5074	-0.0324	5	2	3	6	6	6	7	4	3	1	4	4	5	6
7373.4750	7373.5074	-0.0324	5	4	1	5	5	6	7	4	1	3	4	5	6	6
7373.5186	7373.5814	-0.0628	5	4	1	5	6	6	5	4	1	3	4	5	5	4
7373.5186	7373.5598	-0.0412	5	2	3	5	6	6	5	4	3	1	3	3	4	4
7373.5186	7373.5598	-0.0412	5	2	3	6	6	6	5	4	1	3	4	4	3	4
7373.5186	7373.5814	-0.0628	5	4	1	4	4	5	5	4	3	1	4	3	4	4
7373.5186	7373.5814	-0.0628	5	2	3	5	6	5	5	4	3	1	5	5	5	4
7373.5186	7373.6033	-0.0847	5	2	3	4	4	4	4	4	1	3	4	3	3	3
7373.5186	7373.5814	-0.0628	5	4	1	4	4	5	5	4	3	1	5	5	5	4
7373.5186	7373.5814	-0.0628	5	2	3	5	6	5	5	4	3	1	4	3	4	4
7373.6707	7373.6699	0.0008	5	2	3	5	6	7	7	4	3	1	5	5	5	6
7373.6707	7373.6699	0.0008	5	4	1	5	6	6	7	4	1	3	4	5	5	6
7373.7375	7373.7348	0.0027	5	0	5	5	6	7	8	4	3	1	4	5	6	7
7373.7375	7373.7627	-0.0252	5	2	3	4	4	4	5	4	3	1	3	3	3	4
7373.7375	7373.7444	-0.0069	5	2	3	5	4	3	3	4	3	1	4	3	2	2
7373.7375	7373.7444	-0.0069	5	2	3	4	4	4	3	4	3	1	3	3	3	2
7373.8195	7373.8179	0.0016	5	0	5	5	4	5	6	4	3	1	4	3	4	5
7373.8195	7373.8179	0.0016	5	4	1	5	6	7	6	4	3	1	5	5	6	5
7373.8195	7373.8179	0.0016	5	2	3	5	6	5	6	4	3	1	4	5	4	5
7373.9262	7373.9216	0.0046	5	2	3	5	4	4	5	4	1	3	4	3	3	4
7373.9262	7373.9366	-0.0104	5	2	3	5	4	5	4	4	3	1	4	3	4	3
7373.9262	7373.9366	-0.0104	5	4	1	5	6	5	4	4	3	1	3	2	2	3
7373.9262	7373.9216	0.0046	5	0	5	5	4	5	5	4	3	1	3	4	4	4
7373.9262	7373.9216	0.0046	5	0	5	6	5	5	5	4	3	1	5	4	4	4
7373.9262	7373.9216	0.0046	5	0	5	6	5	5	5	4	3	1	3	4	4	4
7373.9262	7373.9216	0.0046	5	0	5	5	4	5	5	4	3	1	5	4	4	4
7374.0587	7374.0318	0.0269	5	0	5	4	4	5	4	4	3	1	3	3	4	3
7374.0587	7374.0318	0.0269	5	2	3	5	4	3	4	4	1	3	4	3	4	3
7374.0587	7374.0318	0.0269	5	0	5	4	4	3	4	4	3	1	4	5	4	3
7374.0587	7374.0318	0.0269	5	2	3	5	4	3	4	4	3	1	4	5	4	3
7374.0587	7374.0318	0.0269	5	0	5	4	4	3	4	4	1	3	4	3	4	3
7374.0887	7374.0885	0.0002	5	2	3	6	7	8	8	4	3	1	5	6	7	7

7374.0887	7374.0885	0.0002	5	2	3	6	7	7	8	4	1	3	4	5	6	7
7374.0887	7374.0885	0.0002	5	2	3	5	6	7	8	4	3	1	5	6	6	7
7374.0887	7374.0702	0.0185	5	2	3	4	4	5	6	4	3	1	3	3	4	5
7374.0887	7374.0885	0.0002	5	2	3	5	4	5	6	4	3	1	3	4	5	5
7374.0887	7374.0885	0.0002	5	2	3	6	7	7	6	4	3	1	5	6	6	5
7374.0887	7374.0885	0.0002	5	2	3	6	6	5	6	4	3	1	5	5	4	5
7374.2125	7374.1980	0.0145	5	2	3	4	3	3	4	4	3	1	5	4	4	3
7374.2125	7374.1980	0.0145	5	2	3	4	4	3	4	4	3	1	3	2	3	3
7374.2125	7374.1980	0.0145	5	2	3	6	6	5	4	4	3	1	5	5	4	3
7374.2125	7374.1979	0.0146	5	2	3	5	4	3	2	4	3	1	4	3	2	1
7374.2125	7374.1980	0.0145	5	2	3	6	6	5	4	4	3	1	3	2	3	3
7374.2125	7374.1980	0.0145	5	2	3	4	4	3	4	4	3	1	5	5	4	3
7374.2125	7374.1979	0.0146	5	2	3	4	4	3	2	4	3	1	3	3	2	1
7374.2125	7374.1979	0.0146	5	2	3	4	3	3	2	4	3	1	3	2	2	1
7374.2632	7374.2576	0.0056	5	4	1	4	5	5	6	4	3	1	5	4	4	5
7374.2632	7374.2652	-0.0020	5	2	3	6	7	6	6	4	1	3	3	4	5	5
7374.2632	7374.2652	-0.0020	5	2	3	6	5	6	6	4	1	3	5	6	5	5
7374.2632	7374.2576	0.0056	5	0	5	6	5	6	6	4	3	1	5	6	5	5
7374.2632	7374.2652	-0.0020	5	2	3	6	5	6	6	4	1	3	3	4	5	5
7374.2632	7374.2652	-0.0020	5	2	3	6	7	6	6	4	1	3	5	6	5	5
7374.2632	7374.2576	0.0056	5	2	3	6	5	5	6	4	3	1	5	4	4	5
7374.2632	7374.2576	0.0056	5	4	1	4	5	5	6	4	3	1	3	4	4	5
7374.2632	7374.2952	-0.0320	5	2	3	4	3	3	3	4	3	1	3	3	2	2
7374.2632	7374.2576	0.0056	5	2	3	6	5	5	6	4	3	1	3	4	4	5
7374.4910	7374.4654	0.0256	5	2	3	4	5	4	4	4	3	1	5	4	3	3
7374.4910	7374.4654	0.0256	5	2	3	6	5	4	4	4	3	1	3	4	4	3
7374.4910	7374.4654	0.0256	5	2	3	4	4	5	4	4	3	1	3	4	4	3
7374.4910	7374.4654	0.0256	5	2	3	4	4	5	4	4	1	3	5	4	4	3
7374.4910	7374.4654	0.0256	5	2	3	6	5	4	4	4	1	3	5	4	4	3
7374.5878	7374.6173	-0.0295	5	2	3	6	5	6	7	4	3	1	3	4	5	6
7374.5878	7374.5809	0.0069	5	2	3	6	7	6	7	4	3	1	5	6	5	6
7374.5878	7374.5809	0.0069	5	0	5	6	5	6	7	4	3	1	5	4	5	6
7374.5878	7374.5809	0.0069	5	2	3	6	7	8	7	4	3	1	5	6	7	6
7374.5878	7374.5809	0.0069	5	2	3	6	7	8	7	4	3	1	5	4	5	6
7374.5878	7374.5809	0.0069	5	0	5	6	5	6	7	4	3	1	5	6	7	6
7374.7125	7374.6629	0.0496	5	2	3	4	3	4	5	4	3	1	5	6	5	4
7374.7125	7374.6629	0.0496	5	2	3	4	5	4	5	4	3	1	5	4	5	4
7374.7125	7374.7277	-0.0152	5	4	1	4	5	4	5	4	1	3	3	2	3	4
7374.7125	7374.6836	0.0289	5	2	3	4	5	6	5	4	3	1	3	4	5	4
7374.7125	7374.6836	0.0289	5	2	3	6	5	4	5	4	3	1	3	2	3	4
7374.7125	7374.6836	0.0289	5	2	3	6	7	6	5	4	1	3	5	6	5	4
7374.7125	7374.6836	0.0289	5	2	3	6	5	4	5	4	1	3	5	6	5	4
7374.7125	7374.6836	0.0289	5	2	3	6	7	6	5	4	3	1	3	2	3	4
7374.7808	7374.7818	-0.0010	5	2	3	6	5	4	3	4	3	1	5	4	3	2
7374.7808	7374.7818	-0.0010	5	4	1	4	3	4	3	4	3	1	3	4	3	2
7374.7808	7374.7818	-0.0010	5	4	1	4	3	4	3	4	3	1	3	2	3	2
7374.7808	7374.7818	-0.0010	5	0	5	4	3	2	3	4	3	1	3	4	3	2
7374.7808	7374.7818	-0.0010	5	0	5	4	3	2	3	4	3	1	3	2	3	2
7374.7808	7374.7818	-0.0010	5	0	5	4	3	2	3	4	3	1	5	4	3	2
7374.7808	7374.7444	0.0364	5	2	3	4	3	2	3	4	1	3	3	2	2	3
7374.8398	7374.8380	0.0018	5	2	3	4	3	2	3	4	1	3	3	4	3	2
7394.9533	7394.9405	0.0128	4	2	2	4	3	3	3	3	3	0	3	3	3	3
7394.9533	7394.9449	0.0084	4	4	0	3	4	4	5	3	3	0	3	4	4	5

7395.2562	7395.2589	-0.0027	4	4	0	5	4	4	4	3	3	0	3	4	4	4
7395.2562	7395.2589	-0.0027	4	2	2	3	3	4	4	3	3	0	4	4	4	4
7395.2562	7395.2589	-0.0027	4	4	0	3	3	4	4	3	3	0	3	3	4	4
7395.2562	7395.2209	0.0353	4	4	0	3	3	4	5	3	3	0	4	4	5	5
7395.2562	7395.2209	0.0353	4	2	2	5	6	6	5	3	3	0	3	3	4	5
7395.2562	7395.2209	0.0353	4	4	0	5	6	5	5	3	3	0	4	4	4	5
7395.2562	7395.2589	-0.0027	4	2	2	3	3	4	4	3	3	0	3	3	4	4
7395.2562	7395.2589	-0.0027	4	4	0	3	3	4	4	3	3	0	4	4	4	4
7395.4872	7395.4794	0.0078	4	4	0	4	3	2	2	3	3	0	3	3	2	2
7395.4872	7395.4794	0.0078	4	2	2	4	3	3	2	3	3	0	3	2	2	2
7395.4872	7395.5064	-0.0192	4	4	0	3	3	3	4	3	3	0	3	4	4	5
7395.4872	7395.4794	0.0078	4	4	0	3	3	2	2	3	3	0	3	3	3	2
7395.4872	7395.4794	0.0078	4	4	0	3	3	2	2	3	3	0	3	2	2	2
7395.4872	7395.4794	0.0078	4	4	0	4	3	2	2	3	3	0	3	3	3	2
7395.4872	7395.4794	0.0078	4	2	2	4	3	3	2	3	3	0	3	3	3	2
7395.4872	7395.4794	0.0078	4	4	0	3	3	2	2	3	3	0	3	3	2	2
7395.5691	7395.5621	0.0070	4	4	0	3	4	4	3	3	1	2	3	4	3	3
7395.5691	7395.5621	0.0070	4	4	0	3	2	3	3	3	1	2	3	3	4	3
7395.5691	7395.5621	0.0070	4	4	0	5	4	3	3	3	3	0	2	2	2	3
7395.5691	7395.5621	0.0070	4	4	0	5	4	3	3	3	1	2	3	3	4	3
7395.5691	7395.5621	0.0070	4	4	0	3	2	3	3	3	3	0	2	2	2	3
7395.6473	7395.6382	0.0091	4	2	2	3	3	4	5	3	3	0	4	5	5	5
7395.6473	7395.6382	0.0091	4	4	0	5	4	5	5	3	3	0	3	4	5	5
7395.7227	7395.7141	0.0086	4	4	0	5	4	5	6	3	1	2	4	4	5	6
7395.7227	7395.7141	0.0086	4	4	0	5	6	5	6	3	3	0	4	5	5	6
7395.7227	7395.7141	0.0086	4	4	0	5	6	7	6	3	3	0	4	5	6	6
7395.9307	7395.8821	0.0486	4	4	0	3	2	2	2	3	3	0	2	2	2	2
7395.9307	7395.9150	0.0157	4	2	2	5	4	3	3	3	3	0	4	4	3	3
7395.9307	7395.9150	0.0157	4	4	0	3	3	4	3	3	3	0	2	3	3	3
7395.9307	7395.9150	0.0157	4	4	0	3	4	3	3	3	1	2	3	2	3	3
7395.9307	7395.9557	-0.0250	4	4	0	5	4	5	4	3	3	0	4	4	3	4
7395.9307	7395.9150	0.0157	4	4	0	3	3	4	3	3	1	2	3	2	3	3
7395.9307	7395.9557	-0.0250	4	4	0	5	6	5	4	3	1	2	2	2	3	4
7395.9307	7395.8955	0.0352	4	4	0	3	2	2	2	3	3	0	2	2	3	3
7395.9307	7395.9557	-0.0250	4	4	0	5	4	3	4	3	1	2	4	3	4	4
7395.9307	7395.9150	0.0157	4	4	0	3	4	3	3	3	3	0	4	4	3	3
7396.1805	7396.2014	-0.0209	4	2	2	3	4	3	2	3	3	0	2	1	1	2
7396.1805	7396.1784	0.0021	4	4	0	4	3	2	1	3	3	0	3	2	1	1
7396.1805	7396.1784	0.0021	4	4	0	3	2	2	1	3	3	0	2	2	1	1
7396.1805	7396.1748	0.0057	4	4	0	4	3	2	3	3	3	0	3	4	3	3
7396.1805	7396.1748	0.0057	4	4	0	4	3	4	3	3	3	0	3	2	3	3
7396.1805	7396.1784	0.0021	4	4	0	3	3	2	1	3	3	0	2	2	1	1
7396.1805	7396.1784	0.0021	4	4	0	4	3	2	1	3	3	0	2	2	2	1
7396.1805	7396.1784	0.0021	4	4	0	3	2	2	1	3	3	0	2	2	2	1
7396.1805	7396.1784	0.0021	4	4	0	3	3	2	1	3	3	0	3	2	1	1
7396.2942	7396.2867	0.0075	4	4	0	3	2	1	2	3	3	0	2	3	2	2
7396.2942	7396.2867	0.0075	4	4	0	3	4	3	2	3	3	0	2	1	2	2
7396.2942	7396.2867	0.0075	4	4	0	5	4	3	2	3	3	0	4	3	2	2
7396.2942	7396.2793	0.0149	4	4	0	3	2	1	1	3	3	0	2	1	1	2
7396.2942	7396.2867	0.0075	4	4	0	5	4	3	2	3	3	0	2	1	2	2
7396.6159	7396.6128	0.0031	4	4	0	5	6	7	8	3	3	0	4	5	6	7
7396.6159	7396.6600	-0.0441	4	4	0	3	4	5	6	3	1	2	4	3	4	5
7396.6159	7396.6118	0.0041	4	4	0	5	6	5	6	3	3	0	4	5	4	5

7396.6159	7396.6118	0.0041	4	4	0	5	4	5	6	3	3	0	4	3	4	5
7396.6159	7396.6118	0.0041	4	4	0	5	6	7	6	3	3	0	4	3	4	5
7396.6159	7396.6118	0.0041	4	4	0	5	6	7	6	3	3	0	4	5	6	5
7396.6159	7396.6118	0.0041	4	4	0	5	4	5	6	3	3	0	4	5	6	5
7396.6159	7396.6075	0.0084	4	4	0	3	4	5	4	3	3	0	2	3	4	3
7396.6159	7396.6075	0.0084	4	4	0	3	4	3	4	3	3	0	4	5	4	3
7396.6159	7396.6354	-0.0195	4	4	0	5	4	5	4	3	3	0	4	3	2	3
7396.7375	7396.7601	-0.0226	4	4	0	4	5	6	7	3	3	0	4	4	5	6
7396.7375	7396.7279	0.0096	4	4	0	5	5	6	5	3	3	0	4	4	5	4
7396.7375	7396.7279	0.0096	4	4	0	4	5	4	5	3	3	0	3	4	3	4
7396.7375	7396.7279	0.0096	4	4	0	5	4	4	5	3	3	0	3	2	3	4
7396.7375	7396.7121	0.0254	4	2	2	3	4	5	4	3	3	0	2	1	2	3
7396.8130	7396.8054	0.0076	4	4	0	5	6	7	7	3	3	0	4	5	6	6
7396.8130	7396.8054	0.0076	4	4	0	5	6	6	7	3	3	0	4	5	5	6
7396.8130	7396.8054	0.0076	4	4	0	5	5	6	7	3	1	2	4	4	5	6
7396.8130	7396.8281	-0.0151	4	2	2	3	3	4	5	3	3	0	4	5	4	4
7396.8130	7396.8281	-0.0151	4	4	0	5	4	5	5	3	3	0	4	3	4	4
7396.8130	7396.8054	0.0076	4	4	0	5	6	6	5	3	3	0	4	5	5	4
7396.8130	7396.8054	0.0076	4	4	0	5	5	4	5	3	1	2	4	4	5	4
7396.8130	7396.8054	0.0076	4	4	0	3	4	5	5	3	1	2	4	4	5	4
7396.8130	7396.8054	0.0076	4	4	0	5	6	6	5	3	1	2	3	2	3	4
7396.8130	7396.8084	0.0046	4	2	2	4	3	2	3	3	1	2	3	2	1	2
7396.9000	7396.8666	0.0334	4	4	0	5	6	5	5	3	1	2	4	3	4	4
7396.9000	7396.8666	0.0334	4	4	0	3	3	4	5	3	3	0	4	4	3	4
7396.9000	7396.8666	0.0334	4	2	2	5	6	6	5	3	1	2	2	2	3	4
7396.9000	7396.9053	-0.0053	4	4	0	3	3	4	3	3	3	0	2	1	2	2
7396.9000	7396.8471	0.0529	4	4	0	5	4	3	3	3	3	0	4	3	3	2
7396.9000	7396.9053	-0.0053	4	2	2	5	4	3	3	3	3	0	4	3	2	2
7396.9000	7396.9053	-0.0053	4	4	0	3	4	3	3	3	3	0	2	3	2	2
7396.9000	7396.8471	0.0529	4	4	0	3	4	4	3	3	3	0	2	3	3	2
7396.9000	7396.8471	0.0529	4	4	0	3	4	4	3	3	3	0	4	4	3	2
7396.9992	7396.9980	0.0012	4	2	2	5	5	5	6	3	3	0	3	4	5	5
7396.9992	7396.9980	0.0012	4	4	0	5	6	6	6	3	3	0	4	5	5	5
7396.9992	7397.0157	-0.0165	4	4	0	4	5	5	6	3	3	0	3	4	4	5
7396.9992	7396.9877	0.0115	4	4	0	5	5	5	6	3	3	0	4	4	5	5
7396.9992	7396.9536	0.0456	4	4	0	3	4	4	4	3	3	0	3	4	3	3
7396.9992	7396.9536	0.0456	4	4	0	4	3	4	4	3	3	0	3	2	3	3
7396.9992	7396.9867	0.0125	4	4	0	4	5	5	4	3	3	0	2	2	2	3
7396.9992	7396.9877	0.0115	4	4	0	4	5	6	6	3	3	0	3	3	4	5
7396.9992	7396.9679	0.0313	4	4	0	4	3	3	4	3	3	0	3	2	2	3
7396.9992	7396.9877	0.0115	4	2	2	4	5	5	6	3	3	0	4	4	4	5
7397.1358	7397.1272	0.0086	4	4	0	3	3	4	4	3	1	2	3	2	3	3
7397.1358	7397.1272	0.0086	4	2	2	3	3	4	4	3	3	0	4	4	3	3
7397.1358	7397.1272	0.0086	4	4	0	5	4	4	4	3	3	0	2	3	3	3
7397.1358	7397.1071	0.0287	4	2	2	3	2	2	3	3	3	0	2	1	1	2
7397.1358	7397.1405	-0.0047	4	2	2	4	3	3	2	3	3	0	2	2	2	1
7397.1358	7397.1405	-0.0047	4	4	0	3	3	2	2	3	3	0	3	2	1	1
7397.1358	7397.1405	-0.0047	4	4	0	4	3	2	2	3	3	0	2	2	1	1
7397.2129	7397.2033	0.0096	4	4	0	4	4	4	5	3	3	0	3	3	4	4
7397.2129	7397.2033	0.0096	4	4	0	5	5	5	5	3	3	0	4	4	4	4
7397.2129	7397.2033	0.0096	4	4	0	4	5	5	5	3	3	0	3	4	4	4
7397.2129	7397.2033	0.0096	4	4	0	5	5	5	5	3	3	0	3	4	4	4
7397.2129	7397.2092	0.0037	4	4	0	4	4	3	3	3	3	0	3	3	2	2

7397.2129	7397.2092	0.0037	4	4	0	4	4	4	3	3	3	0	3	2	2	2
7397.2129	7397.2033	0.0096	4	4	0	4	5	5	5	3	3	0	4	4	4	4
7397.2129	7397.2092	0.0037	4	4	0	4	3	3	3	3	3	0	3	3	3	2
7397.3335	7397.3676	-0.0341	4	4	0	3	2	2	2	3	3	0	2	1	1	1
7397.3335	7397.3213	0.0122	4	2	2	3	2	2	3	3	3	0	2	1	2	3
7397.3335	7397.3345	-0.0010	4	4	0	3	3	2	2	3	3	0	2	2	3	2
7397.3335	7397.3345	-0.0010	4	2	2	4	3	3	2	3	3	0	2	2	1	2
7397.3335	7397.3345	-0.0010	4	4	0	4	3	2	2	3	1	2	3	2	1	2
7397.4540	7397.4877	-0.0337	4	4	0	3	3	3	4	3	3	0	2	2	3	4
7397.4750	7397.5013	-0.0263	4	2	2	4	3	3	3	3	3	0	2	2	2	2
7397.4750	7397.5147	-0.0397	4	2	2	4	3	3	3	3	3	0	2	2	3	3
7397.5806	7397.5823	-0.0017	4	4	0	4	4	4	3	3	3	0	3	3	4	3
7397.5806	7397.5823	-0.0017	4	4	0	4	4	3	3	3	3	0	3	2	2	3
7397.5806	7397.5823	-0.0017	4	4	0	4	4	3	3	3	3	0	3	3	2	3
7397.5806	7397.5823	-0.0017	4	4	0	4	3	3	3	3	3	0	3	3	2	3
7397.5806	7397.5823	-0.0017	4	4	0	4	3	3	3	3	3	0	3	3	4	3
7397.5806	7397.5823	-0.0017	4	4	0	4	3	3	3	3	3	0	3	2	2	3
7397.5806	7397.5823	-0.0017	4	4	0	4	4	4	3	3	3	0	3	3	2	3
7398.4172	7398.4259	-0.0087	4	4	0	4	5	6	7	3	3	0	4	5	6	7
7398.4172	7398.4147	0.0025	4	4	0	4	5	4	5	3	3	0	4	5	4	5
7398.4172	7398.4147	0.0025	4	4	0	5	5	6	5	3	3	0	4	5	6	5
7398.4172	7398.4147	0.0025	4	4	0	5	4	4	5	3	3	0	4	3	4	5
7398.4172	7398.4147	0.0025	4	4	0	5	4	4	5	3	3	0	4	5	6	5
7419.3658	7419.3701	-0.0043	4	4	1	3	4	4	4	3	3	1	3	4	4	4
7419.3658	7419.3599	0.0059	4	2	3	3	3	4	5	3	3	1	3	3	4	5
7419.3658	7419.3701	-0.0043	4	4	1	5	4	4	4	3	3	1	4	4	4	4
7419.3658	7419.3599	0.0059	4	4	1	5	4	5	5	3	3	1	3	4	5	5
7419.3658	7419.3599	0.0059	4	4	1	4	5	6	5	3	1	3	3	4	4	5
7419.3658	7419.3701	-0.0043	4	4	1	3	3	4	4	3	1	3	3	4	4	4
7419.3658	7419.3701	-0.0043	4	4	1	5	4	4	4	3	1	3	3	4	4	4
7419.3658	7419.3701	-0.0043	4	4	1	3	3	4	4	3	3	1	3	4	4	4
7419.3658	7419.3701	-0.0043	4	4	1	3	3	4	4	3	3	1	4	4	4	4
7419.3658	7419.3278	0.0380	4	4	1	3	3	3	3	3	3	1	3	3	4	4
7420.0449	7420.0355	0.0094	4	4	1	3	2	2	3	3	1	3	3	2	2	3
7420.0449	7420.0212	0.0237	4	4	1	3	2	2	3	3	3	1	2	2	2	2
7420.1272	7420.2123	-0.0851	4	4	1	5	4	5	4	3	3	1	4	3	4	4
7420.1272	7420.2123	-0.0851	4	4	1	5	4	3	4	3	3	1	2	3	4	4
7420.1272	7420.1844	-0.0572	4	4	1	5	6	5	4	3	3	1	2	3	3	4
7420.1272	7420.1844	-0.0572	4	2	3	5	4	3	4	3	3	1	4	3	3	4
7420.1272	7420.1844	-0.0572	4	2	3	5	4	5	4	3	3	1	4	5	4	4
7420.1272	7420.1844	-0.0572	4	2	3	5	4	5	4	3	3	1	4	3	3	4
7420.1272	7420.1844	-0.0572	4	2	3	5	4	3	4	3	3	1	4	5	4	4
7420.1277	7420.0817	0.0460	4	4	1	3	3	2	2	3	3	1	2	2	2	2
7420.1277	7420.1242	0.0035	4	2	3	5	4	4	3	3	3	1	4	3	3	3
7420.1277	7420.1242	0.0035	4	4	1	3	2	3	3	3	3	1	2	2	3	3
7420.1277	7420.1242	0.0035	4	4	1	3	4	4	3	3	1	3	4	3	3	3
7420.1277	7420.1242	0.0035	4	4	1	3	4	4	3	3	3	1	2	2	3	3
7420.1277	7420.0961	0.0316	4	4	1	3	3	2	2	3	1	3	3	2	2	3
7420.1277	7420.1242	0.0035	4	2	3	5	4	4	3	3	1	3	4	3	3	3
7420.1277	7420.1242	0.0035	4	4	1	3	2	3	3	3	1	3	4	3	3	3
7420.1277	7420.1242	0.0035	4	4	1	3	4	4	3	3	3	1	4	3	3	3
7420.5489	7420.5486	0.0003	4	4	1	5	4	3	2	3	1	3	2	3	2	2
7420.5489	7420.5486	0.0003	4	4	1	3	4	3	2	3	3	1	2	1	2	2

7420.5489	7420.5456	0.0033	4	4	1	3	2	1	1	3	3	1	2	2	1	2
7420.5489	7420.5486	0.0003	4	4	1	3	2	1	2	3	3	1	4	3	2	2
7420.5489	7420.5486	0.0003	4	4	1	3	4	3	2	3	3	1	4	3	2	2
7420.5489	7420.5486	0.0003	4	4	1	3	2	1	2	3	1	3	2	3	2	2
7420.5489	7420.5486	0.0003	4	4	1	3	2	1	2	3	3	1	2	1	2	2
7420.8801	7420.8641	0.0160	4	4	1	5	6	7	8	3	3	1	4	5	6	7
7420.8801	7420.9385	-0.0584	4	4	1	4	5	6	7	3	3	1	4	4	5	6
7420.8801	7420.9279	-0.0478	4	4	1	3	4	5	6	3	3	1	2	3	4	5
7420.8801	7420.8715	0.0086	4	4	1	5	6	7	6	3	3	1	4	5	6	5
7420.8801	7420.8715	0.0086	4	4	1	5	6	5	6	3	3	1	4	5	4	5
7420.8801	7420.8715	0.0086	4	4	1	5	4	5	6	3	3	1	4	3	4	5
7420.8801	7420.8763	0.0038	4	4	1	5	4	3	4	3	3	1	2	3	4	3
7420.8801	7420.8763	0.0038	4	4	1	5	4	5	4	3	3	1	2	3	2	3
7420.8801	7420.9131	-0.0330	4	4	1	5	5	4	5	3	3	1	4	4	3	4
7420.8801	7420.9089	-0.0288	4	4	1	5	6	5	4	3	3	1	4	5	4	3
7421.0351	7421.0281	0.0070	4	4	1	5	5	6	7	3	3	1	4	5	5	6
7421.0351	7421.0281	0.0070	4	4	1	4	5	4	5	3	3	1	4	4	5	4
7421.0351	7421.0281	0.0070	4	4	1	5	6	7	7	3	3	1	4	5	6	6
7421.0351	7420.9969	0.0382	4	4	1	3	2	3	4	3	3	1	2	1	2	3
7421.0351	7421.0281	0.0070	4	4	1	5	6	6	5	3	3	1	3	2	3	4
7421.0351	7421.0281	0.0070	4	4	1	5	6	6	7	3	3	1	4	5	6	6
7421.0351	7421.0281	0.0070	4	4	1	5	5	6	7	3	1	3	4	4	5	6
7421.0351	7421.0281	0.0070	4	4	1	3	3	4	5	3	3	1	4	5	5	4
7421.0351	7421.0281	0.0070	4	4	1	5	6	7	7	3	1	3	4	4	5	6
7421.0351	7421.0729	-0.0378	4	2	3	5	4	5	5	3	3	1	4	3	4	4
7421.1328	7421.1624	-0.0296	4	4	1	5	5	6	6	3	1	3	3	4	4	5
7421.1328	7421.1624	-0.0296	4	4	1	5	5	5	6	3	3	1	3	3	4	5
7421.1328	7421.1624	-0.0296	4	4	1	4	5	6	6	3	3	1	3	4	5	5
7421.1328	7421.1580	-0.0252	4	4	1	4	5	5	6	3	3	1	3	4	4	5
7421.1328	7421.1697	-0.0369	4	4	1	4	5	4	4	3	3	1	3	4	3	3
7421.1328	7421.1566	-0.0238	4	4	1	3	4	5	5	3	3	1	2	2	3	4
7421.1328	7421.1697	-0.0369	4	4	1	5	5	5	4	3	3	1	4	4	4	3
7421.1328	7421.1697	-0.0369	4	2	3	4	5	5	4	3	1	3	3	4	4	3
7421.1328	7421.1921	-0.0593	4	2	3	4	5	6	6	3	3	1	4	5	5	5
7421.1328	7421.1921	-0.0593	4	2	3	4	5	5	6	3	3	1	4	4	5	5
7421.3512	7421.3896	-0.0384	4	4	1	4	4	4	5	3	3	1	3	3	4	4
7421.3512	7421.3538	-0.0026	4	4	1	4	5	5	5	3	3	1	3	4	4	4
7421.3512	7421.3609	-0.0097	4	4	1	3	4	4	4	3	3	1	4	3	3	3
7421.3512	7421.3538	-0.0026	4	4	1	5	5	5	5	3	1	3	3	4	4	4
7421.3512	7421.3609	-0.0097	4	4	1	3	3	4	4	3	3	1	2	2	3	3
7421.3512	7421.3609	-0.0097	4	4	1	5	4	4	4	3	1	3	4	3	3	3
7421.3512	7421.3538	-0.0026	4	4	1	4	4	5	5	3	1	3	3	4	4	4
7421.3512	7421.3495	0.0017	4	4	1	4	4	3	3	3	3	1	3	3	2	2
7421.3512	7421.3538	-0.0026	4	4	1	4	4	5	5	3	3	1	4	4	4	4
7421.3512	7421.3538	-0.0026	4	4	1	4	5	5	5	3	3	1	4	4	4	4
7421.5551	7421.5593	-0.0042	4	4	1	4	4	4	4	3	3	1	3	3	3	3
7421.5551	7421.5196	0.0355	4	2	3	3	3	3	4	3	1	3	3	2	2	3
7421.7336	7421.7337	-0.0001	4	4	1	3	3	3	3	3	3	1	2	2	2	2
7421.7336	7421.7294	0.0042	4	2	3	3	3	3	4	3	3	1	2	2	3	4
7421.7336	7421.7469	-0.0133	4	4	1	4	4	4	3	3	3	1	3	4	4	3
7421.7336	7421.7469	-0.0133	4	4	1	4	4	3	3	3	3	1	3	3	2	3
7421.7336	7421.7481	-0.0145	4	4	1	3	3	3	3	3	1	3	3	2	2	3
7421.7336	7421.7469	-0.0133	4	4	1	4	3	3	3	3	3	1	3	2	2	3

7422.6997	7422.7139	-0.0142	4	4	1	4	5	6	7	3	3	1	4	5	6	7
7422.6997	7422.7109	-0.0112	4	4	1	4	3	4	5	3	3	1	4	3	4	5
7422.6997	7422.7109	-0.0112	4	4	1	5	5	4	5	3	3	1	4	5	6	5
7422.6997	7422.7109	-0.0112	4	4	1	5	5	6	5	3	3	1	4	5	4	5
7422.6997	7422.7109	-0.0112	4	4	1	5	5	6	5	3	3	1	4	5	6	5
7422.6997	7422.7109	-0.0112	4	4	1	5	5	4	5	3	3	1	4	5	4	5

4.2. Cluster of 3,3-dimethyl-1,1,2,2-tetracyanocyclopropane with tetrahydrofuran

Table 9: Transition frequencies for the 3,3-dimethyl-1,1,2,2-tetracyanocyclopropane complex with tetrahydrofuran. J is the rotational angular momentum quantum number, K_a and K_c are the projections of J onto the principal axes at the prolate and oblate symmetric top limits, respectively, and $I_{7,9}$, F_1, $I_{8,10}$ and F_2 are angular momentum quantum numbers obtained using the following coupling schemes between the rotational angular momentum and the nuclear spins of the four nitrogen nuclei (I(^{14}N) = 1): $\mathbf{I_{7,9}} = \mathbf{I_{N7}} + \mathbf{I_{N9}}$, $\mathbf{F_1} = \mathbf{J} + \mathbf{I_{7,9}}$, $\mathbf{I_{8,10}} = \mathbf{I_{N8}} + \mathbf{I_{N10}}$ and $\mathbf{F_2} = \mathbf{F_1} + \mathbf{I_{8,10}}$

Observed Freq. /MHz	Calculated Freq. /MHz	Difference / MHz	J	K_a	K_c	$I_{7,9}$	F_1	$I_{8,10}$	F_2	J'	K'_a	K'_c	$I'_{7,9}$	F'_1	$I'_{8,10}$	F'_2
2582.7423	2582.7167	0.0256	4	1	4	0	4	2	6	3	1	3	0	3	2	5
2582.7423	2582.7328	0.0095	4	1	4	1	4	2	6	3	1	3	1	3	2	5
2582.7423	2582.7576	-0.0153	4	1	4	1	5	1	6	3	1	3	1	4	1	5
2582.7423	2582.7466	-0.0043	4	1	4	1	5	2	7	3	1	3	1	4	2	6
2582.7423	2582.7332	0.0091	4	1	4	2	6	0	6	3	1	3	2	5	0	5
2582.7423	2582.7346	0.0077	4	1	4	2	6	1	6	3	1	3	2	5	1	5
2582.7423	2582.7550	-0.0127	4	1	4	2	6	1	7	3	1	3	2	5	1	6
2582.7423	2582.7681	-0.0258	4	1	4	2	6	2	6	3	1	3	2	5	2	5
2582.7423	2582.7545	-0.0122	4	1	4	2	6	2	7	3	1	3	2	5	2	6
2582.7423	2582.7440	-0.0017	4	1	4	2	6	2	8	3	1	3	2	5	2	7
2685.3188	2685.3166	0.0022	4	1	3	0	4	2	5	3	1	2	0	3	2	4
2685.3188	2685.3260	-0.0072	4	1	3	1	3	1	3	3	1	2	1	2	1	2
2685.3188	2685.3172	0.0016	4	1	3	1	4	2	5	3	3	0	1	2	2	4
2685.3188	2685.3259	-0.0071	4	1	3	2	3	2	3	3	1	2	2	2	2	2
2685.3188	2685.3161	0.0027	4	1	3	2	5	1	5	3	1	2	2	4	1	4
2685.3188	2685.3251	-0.0063	4	1	3	2	5	2	7	3	1	2	2	4	2	6
2685.4250	2685.4662	-0.0412	4	1	3	0	4	1	4	3	1	2	0	3	1	3
2685.4250	2685.4653	-0.0403	4	1	3	1	4	0	4	3	1	2	1	3	0	3
2685.4250	2685.4319	-0.0069	4	1	3	1	4	1	4	3	1	2	1	3	1	3
2685.4250	2685.4131	0.0119	4	1	3	1	4	2	6	3	1	2	1	3	2	5
2685.4250	2685.4318	-0.0068	4	1	3	2	3	2	4	3	1	2	2	2	2	3
2685.4250	2685.4352	-0.0102	4	1	3	2	5	2	6	3	1	2	2	4	2	5
2685.4250	2685.4123	0.0127	4	1	3	2	6	0	6	3	1	2	2	5	0	5
2685.4250	2685.4116	0.0134	4	1	3	2	6	1	6	3	1	2	2	5	1	5
2685.4250	2685.4658	-0.0408	4	1	3	2	6	2	4	3	1	2	2	5	2	3
2685.4250	2685.3921	0.0329	4	1	3	2	6	2	8	3	1	2	2	5	2	7
2685.4686	2685.4711	-0.0025	4	1	3	0	4	2	6	3	1	2	0	3	2	5
2685.4686	2685.4711	-0.0025	4	1	3	1	3	2	5	3	1	2	1	2	2	4
2685.4686	2685.4720	-0.0034	4	1	3	1	5	1	5	3	1	2	1	4	1	4
2685.4686	2685.4967	-0.0281	4	1	3	1	5	2	6	3	1	2	1	4	2	5

2685.4686	2685.4703	-0.0017	4	1	3	1	5	2	7	3	1	2	1	4	2	6
2685.4686	2685.4950	-0.0264	4	1	3	2	4	2	6	3	1	2	2	3	2	5
2685.4686	2685.4936	-0.0250	4	1	3	2	5	1	6	3	1	2	2	4	1	5
2685.4686	2685.4729	-0.0043	4	1	3	2	6	1	5	3	1	2	2	5	1	4
2685.4686	2685.4738	-0.0052	4	1	3	2	6	1	7	3	1	2	2	5	1	6
2685.4686	2685.4721	-0.0035	4	1	3	2	6	2	7	3	1	2	2	5	2	6
2685.5614	2685.5835	-0.0221	4	1	3	0	4	1	5	3	1	2	0	3	1	4
2685.5614	2685.5458	0.0156	4	1	3	1	4	1	5	3	1	2	1	3	1	4
2685.5614	2685.5808	-0.0194	4	1	3	1	5	0	5	3	1	2	1	4	0	4
2685.5614	2685.5520	0.0094	4	1	3	1	5	1	6	3	1	2	1	4	1	5
2685.5614	2685.6068	-0.0454	4	1	3	1	5	2	5	3	1	2	1	4	2	4
2685.5614	2685.6010	-0.0396	4	1	3	2	4	1	5	3	1	2	2	3	1	4
2685.5614	2685.5458	0.0156	4	1	3	2	4	2	5	3	1	2	2	3	2	4
2685.5614	2685.6039	-0.0425	4	1	3	2	5	2	5	3	1	2	2	4	2	4
2685.5614	2685.5822	-0.0208	4	1	3	2	6	2	5	3	1	2	2	5	2	4
2685.5614	2685.5522	0.0092	4	1	3	2	6	2	6	3	1	2	2	5	2	5
3225.7766	3225.7890	-0.0124	5	1	5	1	5	2	7	4	1	4	1	4	2	6
3225.7766	3225.7233	0.0533	5	1	5	1	6	1	7	4	1	4	1	5	1	6
3225.7766	3225.7802	-0.0036	5	1	5	1	6	2	8	4	1	4	1	5	2	7
3225.7766	3225.8036	-0.0270	5	1	5	2	6	2	8	4	1	4	2	5	2	7
3225.7766	3225.7869	-0.0103	5	1	5	2	7	0	7	4	1	4	2	6	0	6
3225.7766	3225.7670	0.0096	5	1	5	2	7	1	8	4	1	4	2	6	1	7
3225.7766	3225.8054	-0.0288	5	1	5	2	7	2	8	4	1	4	2	6	2	7
3225.7766	3225.7937	-0.0171	5	1	5	2	7	2	9	4	1	4	2	6	2	8
3265.5963	3265.6176	-0.0213	5	0	5	0	5	2	5	4	0	4	0	4	2	4
3265.5963	3265.5700	0.0263	5	0	5	1	4	2	4	4	2	2	1	3	2	3
3265.5963	3265.6138	-0.0175	5	0	5	1	4	2	5	4	0	4	1	3	2	4
3265.5963	3265.5962	0.0001	5	0	5	1	6	2	6	4	0	4	1	5	2	5
3265.5963	3265.5612	0.0351	5	0	5	2	5	1	4	4	0	4	2	4	1	3
3265.5963	3265.5882	0.0081	5	0	5	2	5	1	6	4	2	2	2	4	1	5
3265.5963	3265.5704	0.0259	5	0	5	2	5	2	4	4	0	4	2	4	2	3
3265.5963	3265.6068	-0.0105	5	0	5	2	6	1	5	4	0	4	2	5	1	4
3265.5963	3265.5890	0.0073	5	0	5	2	6	2	6	4	0	4	2	5	2	5
3265.5963	3265.5842	0.0121	5	0	5	2	6	2	7	4	0	4	2	5	2	6
3265.6993	3265.7074	-0.0081	5	0	5	0	5	2	6	4	0	4	0	4	2	5
3265.6993	3265.6811	0.0182	5	0	5	1	4	1	5	4	0	4	1	3	1	4
3265.6993	3265.7064	-0.0071	5	0	5	1	5	2	6	4	0	4	1	4	2	5
3265.6993	3265.7088	-0.0095	5	0	5	1	6	1	7	4	0	4	1	5	1	6
3265.6993	3265.6613	0.0380	5	0	5	1	6	2	7	4	0	4	1	5	2	6
3265.6993	3265.6944	0.0049	5	0	5	2	5	2	7	4	0	4	2	4	2	6
3265.6993	3265.6621	0.0372	5	0	5	2	6	1	7	4	0	4	2	5	1	6
3265.6993	3265.7061	-0.0068	5	0	5	2	7	1	6	4	0	4	2	6	1	5
3265.6993	3265.6697	0.0296	5	0	5	2	7	2	7	4	0	4	2	6	2	6
3265.6993	3265.6860	0.0133	5	0	5	2	7	2	8	4	0	4	2	5	2	7
3265.8010	3265.7900	0.0110	5	0	5	0	5	1	6	4	0	4	2	5	1	5
3265.8010	3265.7823	0.0187	5	0	5	1	4	2	6	4	0	4	1	3	2	5
3265.8010	3265.7933	0.0077	5	0	5	1	5	1	6	4	0	4	1	4	1	5
3265.8010	3265.8365	-0.0355	5	0	5	1	6	0	6	4	0	4	1	5	0	5
3265.8010	3265.8328	-0.0318	5	0	5	1	6	1	6	4	0	4	1	5	1	5
3265.8010	3265.7826	0.0184	5	0	5	1	6	2	8	4	0	4	1	5	2	7
3265.8010	3265.8349	-0.0339	5	0	5	2	5	2	6	4	0	4	2	4	2	5
3265.8010	3265.8361	-0.0351	5	0	5	2	6	1	6	4	0	4	2	4	1	5
3265.8010	3265.7816	0.0194	5	0	5	2	6	2	8	4	0	4	2	6	2	7

3265.8010	3265.7907	0.0103	5	0	5	2	7	1	8	4	0	4	2	6	1	7
3265.9259	3265.9170	0.0089	5	0	5	0	5	1	5	4	0	4	0	4	1	4
3265.9259	3265.8864	0.0395	5	0	5	0	5	2	7	4	0	4	0	4	2	6
3265.9259	3265.9178	0.0081	5	0	5	1	5	0	5	4	0	4	1	4	0	4
3265.9259	3265.9084	0.0175	5	0	5	1	5	1	5	4	0	4	1	4	1	4
3265.9259	3265.8851	0.0408	5	0	5	1	5	2	7	4	0	4	1	4	2	6
3265.9259	3265.9106	0.0153	5	0	5	2	4	2	5	4	0	4	2	3	2	4
3265.9259	3265.8998	0.0261	5	0	5	2	7	0	7	4	0	4	2	6	0	6
3265.9259	3265.8854	0.0405	5	0	5	2	7	1	7	4	0	4	2	6	1	6
3265.9259	3265.9355	-0.0096	5	0	5	2	7	2	5	4	0	4	2	2	2	4
3265.9259	3265.8646	0.0613	5	0	5	2	7	2	9	4	0	4	2	6	2	8
3293.0138	3293.0032	0.0106	5	2	4	0	5	1	5	4	2	3	0	4	1	4
3293.0138	3293.0113	0.0025	5	2	4	0	5	2	7	4	2	3	0	4	2	6
3293.0138	3293.0034	0.0104	5	2	4	1	5	0	5	4	2	3	1	4	0	4
3293.0138	3293.0028	0.0110	5	2	4	1	5	1	5	4	2	3	1	4	1	4
3293.0138	3293.0117	0.0021	5	2	4	1	5	2	7	4	2	3	1	4	2	6
3293.0138	3293.0047	0.0091	5	2	4	2	3	2	5	4	2	3	2	2	2	4
3293.0138	3293.0033	0.0105	5	2	4	2	4	2	5	4	2	3	2	3	2	4
3293.0138	3293.0116	0.0022	5	2	4	2	7	0	7	4	2	3	2	6	0	6
3293.0138	3293.0107	0.0031	5	2	4	2	7	1	7	4	2	3	2	6	1	6
3293.0138	3293.0189	-0.0051	5	2	4	2	7	2	9	4	2	3	2	6	2	8
3293.0985	3293.0849	0.0136	5	2	4	0	5	1	6	4	2	3	0	4	1	5
3293.0985	3293.0926	0.0059	5	2	4	1	4	2	6	4	2	3	1	3	2	5
3293.0985	3293.0779	0.0206	5	2	4	1	5	1	6	4	2	3	1	4	1	5
3293.0985	3293.0815	0.0170	5	2	4	1	6	0	6	4	2	3	1	5	0	5
3293.0985	3293.0955	0.0030	5	2	4	1	6	1	6	4	2	3	1	5	1	5
3293.0985	3293.0908	0.0077	5	2	4	1	6	2	8	4	2	3	1	5	2	7
3293.0985	3293.1262	-0.0277	5	2	4	2	6	2	8	4	2	3	2	5	2	7
3293.0985	3293.0984	0.0001	5	2	4	2	7	1	6	4	2	3	2	6	1	5
3293.0985	3293.0986	-0.0001	5	2	4	2	7	1	8	4	2	3	2	6	1	7
3293.0985	3293.0942	0.0043	5	2	4	2	7	2	8	4	2	3	2	6	2	7
3293.1857	3293.1778	0.0079	5	2	4	0	5	0	5	4	2	3	0	4	0	4
3293.1857	3293.1781	0.0076	5	2	4	1	4	1	5	4	2	3	1	3	1	4
3293.1857	3293.1807	0.0050	5	2	4	1	5	2	5	4	2	3	1	4	2	4
3293.1857	3293.1706	0.0151	5	2	4	1	6	1	7	4	2	3	1	5	1	6
3293.1857	3293.1870	-0.0013	5	2	4	1	6	2	7	4	2	3	1	5	2	6
3293.1857	3293.1738	0.0119	5	2	4	2	5	1	5	4	2	3	2	4	1	4
3293.1857	3293.1684	0.0173	5	2	4	2	5	2	7	4	2	3	2	4	2	6
3293.1857	3293.1851	0.0006	5	2	4	2	6	1	7	4	2	3	2	5	1	6
3293.1857	3293.2195	-0.0338	5	2	4	2	6	2	7	4	2	3	2	5	2	6
3293.1857	3293.1876	-0.0019	5	2	4	2	7	2	7	4	2	3	2	6	2	6
3293.2716	3293.2758	-0.0042	5	2	4	1	4	2	4	4	2	3	1	3	2	3
3293.2716	3293.2671	0.0045	5	2	4	1	6	2	6	4	2	3	1	5	2	5
3293.2716	3293.2421	0.0295	5	2	4	2	4	0	4	4	2	3	2	3	0	3
3293.2716	3293.2671	0.0045	5	2	4	2	5	1	4	4	2	3	2	4	1	3
3293.2716	3293.2574	0.0142	5	2	4	2	5	1	6	4	2	3	2	4	1	5
3293.2716	3293.2719	-0.0003	5	2	4	2	5	2	4	4	2	3	2	4	2	3
3293.2716	3293.2957	-0.0241	5	2	4	2	5	2	6	4	2	3	2	5	0	5
3293.2716	3293.2619	0.0097	5	2	4	2	6	2	6	4	2	3	2	5	2	5
3353.2135	3353.2098	0.0037	5	1	4	0	5	2	6	4	1	3	0	4	2	5
3353.2135	3353.2369	-0.0234	5	1	4	1	4	1	4	4	1	3	1	3	1	3
3353.2135	3353.2415	-0.0280	5	1	4	1	4	2	5	4	1	3	1	3	2	4
3353.2135	3353.2102	0.0033	5	1	4	1	5	2	6	4	1	3	1	4	2	5

3353.2135	3353.2369	-0.0234	5	1	4	2	4	2	4	4	1	3	2	3	2	3
3353.2135	3353.2391	-0.0256	5	1	4	2	6	1	5	4	1	3	2	5	1	4
3353.2135	3353.2094	0.0041	5	1	4	2	6	1	6	4	1	3	2	5	1	5
3353.2135	3353.2403	-0.0268	5	1	4	2	6	2	5	4	1	3	2	4	0	4
3353.2135	3353.2269	-0.0134	5	1	4	2	6	2	7	4	1	3	2	5	2	6
3353.2135	3353.2002	0.0133	5	1	4	2	6	2	8	4	1	3	2	5	2	7
3353.3473	3353.3437	0.0036	5	1	4	0	5	2	7	4	1	3	0	4	2	6
3353.3473	3353.3129	0.0344	5	1	4	1	5	2	7	4	1	3	1	4	2	6
3353.3473	3353.3594	-0.0121	5	1	4	1	6	1	7	4	1	3	1	5	1	6
3353.3473	3353.3239	0.0234	5	1	4	1	6	2	8	4	1	3	1	5	2	7
3353.3473	3353.3125	0.0348	5	1	4	2	7	0	7	4	1	3	2	6	0	6
3353.3473	3353.3120	0.0353	5	1	4	2	7	1	7	4	1	3	2	6	1	6
3353.3473	3353.3271	0.0202	5	1	4	2	7	1	8	4	1	3	2	6	1	7
3353.3473	3353.3594	-0.0121	5	1	4	2	7	2	7	4	1	3	2	6	2	6
3353.3473	3353.3255	0.0218	5	1	4	2	7	2	8	4	1	3	2	6	2	7
3353.3473	3353.2916	0.0557	5	1	4	2	7	2	9	4	1	3	2	6	2	8
3353.4908	3353.4856	0.0052	5	1	4	0	5	2	4	4	1	3	0	4	2	3
3353.4908	3353.4920	-0.0012	5	1	4	0	5	2	5	4	1	3	0	4	2	4
3353.4908	3353.4849	0.0059	5	1	4	1	5	2	4	4	1	3	1	4	2	3
3353.4908	3353.4924	-0.0016	5	1	4	1	6	2	5	4	1	3	1	5	2	4
3353.4908	3353.4502	0.0406	5	1	4	2	3	0	3	4	1	3	2	2	0	2
3353.4908	3353.5264	-0.0356	5	1	4	2	3	2	4	4	1	3	2	2	2	3
3353.4908	3353.4863	0.0045	5	1	4	2	4	1	4	4	1	3	2	2	1	3
3353.4908	3353.4916	-0.0008	5	1	4	2	4	1	5	4	1	3	2	3	1	4
3353.4908	3353.5029	-0.0121	5	1	4	2	5	2	5	4	1	3	2	4	2	4
3867.3415	3867.3752	-0.0337	6	1	6	1	6	2	8	5	1	5	1	5	2	7
3867.3415	3867.3380	0.0035	6	1	6	1	7	1	8	5	1	5	1	6	1	7
3867.3415	3867.3551	-0.0136	6	1	6	1	7	2	9	5	1	5	1	6	2	8
3867.3415	3867.3653	-0.0238	6	1	6	2	7	2	9	5	1	5	2	6	2	8
3867.3415	3867.3760	-0.0345	6	1	6	2	8	0	8	5	1	5	2	7	0	7
3867.3415	3867.3765	-0.0350	6	1	6	2	8	1	8	5	1	5	2	7	1	7
3867.3415	3867.3602	-0.0187	6	1	6	2	8	1	9	5	1	5	2	7	1	8
3867.3415	3867.3427	-0.0012	6	1	6	2	8	2	8	5	1	5	2	7	2	7
3867.3415	3867.3593	-0.0178	6	1	6	2	8	2	9	5	1	5	2	7	2	8
3867.3415	3867.3772	-0.0357	6	1	6	2	8	2	10	5	1	5	2	7	2	9
3904.4765	3904.4852	-0.0087	6	4	2	2	5	2	6	5	2	3	2	5	2	5
3904.5500	3904.5796	-0.0296	6	0	6	1	5	2	5	5	0	5	1	4	2	4
3904.5500	3904.6000	-0.0500	6	0	6	1	7	2	7	5	0	5	1	6	2	6
3904.5500	3904.5711	-0.0211	6	0	6	2	6	1	5	5	0	5	2	5	1	4
3904.5500	3904.5920	-0.0420	6	0	6	2	6	1	7	5	0	5	2	5	1	6
3904.5500	3904.5891	-0.0391	6	0	6	2	7	2	8	5	0	5	2	6	2	7
3904.5500	3904.5856	-0.0356	6	2	4	0	6	2	7	5	0	5	2	6	2	6
3904.5500	3904.5923	-0.0423	6	2	4	2	5	0	5	5	0	5	2	5	2	4
3904.5500	3904.5484	0.0016	6	2	4	2	7	0	7	5	0	5	2	6	0	6
3904.6500	3904.6703	-0.0203	6	0	6	0	6	2	6	5	0	5	0	5	2	5
3904.6500	3904.6969	-0.0469	6	0	6	1	5	1	6	5	0	5	1	4	1	5
3904.6500	3904.6946	-0.0446	6	0	6	1	7	1	6	5	0	5	1	6	1	5
3904.6500	3904.7177	-0.0677	6	0	6	1	7	1	8	5	0	5	1	6	1	7
3904.6500	3904.6815	-0.0315	6	0	6	1	7	2	8	5	0	5	1	6	2	7
3904.6500	3904.7446	-0.0946	6	0	6	2	6	0	6	5	0	5	2	5	0	5
3904.6500	3904.6821	-0.0321	6	0	6	2	7	1	8	5	0	5	2	6	1	7
3904.6500	3904.6773	-0.0273	6	0	6	2	7	2	8	5	0	5	2	7	2	7
3904.6500	3904.7440	-0.0940	6	0	6	2	8	2	9	5	0	5	2	7	2	8

3904.6500	3904.6945	-0.0445	6	2	4	0	6	2	8	5	0	5	2	5	2	7
3904.8179	3904.7981	0.0198	6	0	6	1	5	2	7	5	0	5	1	4	2	6
3904.8179	3904.8213	-0.0034	6	0	6	1	6	1	7	5	0	5	1	5	1	6
3904.8179	3904.7621	0.0558	6	0	6	1	6	2	7	5	0	5	1	5	2	6
3904.8179	3904.8373	-0.0194	6	0	6	1	7	0	7	5	0	5	1	6	0	6
3904.8179	3904.8341	-0.0162	6	0	6	1	7	1	7	5	0	5	1	6	1	6
3904.8179	3904.8040	0.0139	6	0	6	1	7	2	9	5	0	5	1	6	2	8
3904.8179	3904.8379	-0.0200	6	0	6	2	5	2	7	5	0	5	2	5	2	6
3904.8179	3904.7933	0.0246	6	0	6	2	7	2	9	5	0	5	2	6	2	8
3904.8179	3904.7637	0.0542	6	0	6	2	8	1	7	5	0	5	2	7	1	6
3904.8179	3904.8121	0.0058	6	0	6	2	8	1	9	5	0	5	2	7	1	8
3904.9499	3904.9373	0.0126	6	0	6	0	6	1	6	5	0	5	0	5	1	5
3904.9499	3904.9165	0.0334	6	0	6	0	6	2	8	5	0	5	0	5	2	7
3904.9499	3904.9378	0.0121	6	0	6	1	6	0	6	5	0	5	1	5	0	5
3904.9499	3904.9330	0.0169	6	0	6	1	6	1	6	5	0	5	1	5	1	5
3904.9499	3904.9146	0.0353	6	0	6	1	6	2	8	5	0	5	1	5	2	7
3904.9499	3904.9350	0.0149	6	0	6	2	5	2	6	5	0	5	2	4	2	5
3904.9499	3904.9210	0.0289	6	0	6	2	8	0	8	5	0	5	2	7	0	7
3904.9499	3904.9148	0.0351	6	0	6	2	8	1	8	5	0	5	2	7	1	7
3904.9499	3904.9424	0.0075	6	0	6	2	8	2	6	5	0	5	2	7	2	5
3904.9499	3904.8984	0.0515	6	0	6	2	8	2	10	5	0	5	2	7	2	9
3948.7521	3948.7353	0.0168	6	2	5	1	5	2	7	5	2	4	1	4	2	6
3948.7521	3948.7745	-0.0224	6	2	5	1	7	1	8	5	2	4	1	6	1	7
3948.7521	3948.7736	-0.0215	6	2	5	1	7	2	8	5	2	4	1	6	2	7
3948.7521	3948.7341	0.0180	6	2	5	1	7	2	9	5	2	4	1	6	2	8
3948.7521	3948.7720	-0.0199	6	2	5	2	6	2	8	5	2	4	2	5	2	7
3948.7521	3948.7730	-0.0209	6	2	5	2	7	1	8	5	2	4	2	6	1	7
3948.7521	3948.7331	0.0190	6	2	5	2	7	2	9	5	2	4	2	6	2	8
3948.7521	3948.7398	0.0123	6	2	5	2	8	1	9	5	2	4	2	7	1	8
3948.7521	3948.7751	-0.0230	6	2	5	2	8	2	8	5	2	4	2	7	2	7
3948.7521	3948.7364	0.0157	6	2	5	2	8	2	9	5	2	4	2	7	2	8
3999.8196	3999.8129	0.0067	6	2	4	0	6	1	6	5	2	3	0	5	1	5
3999.8196	3999.8129	0.0067	6	2	4	1	6	0	6	5	2	3	1	5	0	5
3999.8196	3999.8103	0.0093	6	2	4	1	6	1	6	5	2	3	1	5	1	5
3999.8196	3999.8193	0.0003	6	2	4	1	6	2	8	5	2	3	1	5	2	7
3999.8196	3999.8129	0.0067	6	2	4	2	4	2	6	5	4	1	2	3	2	5
3999.8196	3999.8190	0.0006	6	2	4	2	8	1	8	5	2	3	2	7	1	7
3999.8196	3999.8103	0.0093	6	2	4	2	8	2	6	5	2	3	2	7	2	5
3999.8196	3999.8277	-0.0081	6	2	4	2	8	2	10	5	2	3	2	7	2	9
3999.8196	3999.8233	-0.0037	6	4	2	0	6	2	8	5	2	3	0	5	2	7
3999.8196	3999.8192	0.0004	6	4	2	2	8	0	8	5	2	3	2	7	0	7
3999.9930	3999.9640	0.0290	6	0	6	2	6	2	7	5	0	5	2	5	2	6
3999.9930	3999.9569	0.0361	6	2	4	0	6	1	7	5	2	3	0	5	1	6
3999.9930	3999.9569	0.0361	6	2	4	1	6	1	7	5	2	3	1	5	1	6
3999.9930	3999.9451	0.0479	6	2	4	1	7	0	7	5	2	3	1	6	0	6
3999.9930	3999.9710	0.0220	6	2	4	1	7	1	7	5	2	3	1	6	1	6
3999.9930	3999.9710	0.0220	6	2	4	1	7	2	9	5	2	3	1	6	2	8
3999.9930	4000.0012	-0.0082	6	2	4	2	7	2	9	5	2	3	2	6	2	8
3999.9930	3999.9718	0.0212	6	2	4	2	8	1	7	5	2	3	2	7	1	6
3999.9930	3999.9721	0.0209	6	2	4	2	8	1	9	5	2	3	2	7	1	8
3999.9930	3999.9716	0.0214	6	2	4	2	8	2	9	5	2	3	2	7	2	8
4000.1192	4000.1310	-0.0118	6	0	6	2	6	2	8	5	2	3	2	5	2	7
4000.1192	4000.1220	-0.0028	6	2	4	1	5	1	6	5	2	3	1	4	1	5

4000.1192	4000.1228	-0.0036	6	2	4	1	7	1	6	5	2	3	1	6	1	5
4000.1192	4000.1154	0.0038	6	2	4	1	7	1	8	5	2	3	1	6	1	7
4000.1192	4000.1235	-0.0043	6	2	4	1	7	2	6	5	2	3	1	6	2	5
4000.1192	4000.1316	-0.0124	6	2	4	1	7	2	8	5	2	3	1	6	2	7
4000.1192	4000.1200	-0.0008	6	2	4	2	7	1	6	5	2	3	2	6	1	5
4000.1192	4000.1313	-0.0121	6	2	4	2	7	1	8	5	2	3	2	6	1	7
4000.1192	4000.1200	-0.0008	6	2	4	2	7	2	6	5	2	3	2	6	2	5
4000.1192	4000.1673	-0.0481	6	2	4	2	7	2	8	5	2	3	2	6	2	7
4000.2867	4000.2858	0.0009	6	0	6	2	6	2	5	5	2	3	2	5	2	4
4000.2867	4000.2772	0.0095	6	0	6	2	7	2	7	5	0	5	2	6	2	6
4000.2867	4000.2864	0.0003	6	2	4	1	5	2	5	5	2	3	1	4	2	4
4000.2867	4000.2780	0.0087	6	2	4	1	7	2	7	5	2	3	1	6	2	6
4000.2867	4000.2852	0.0015	6	2	4	2	6	1	5	5	2	3	2	5	1	4
4000.2867	4000.2764	0.0103	6	2	4	2	6	1	7	5	2	3	2	5	1	6
4000.2867	4000.3170	-0.0303	6	2	4	2	6	2	7	5	2	3	2	5	2	6
4000.2867	4000.2840	0.0027	6	4	2	2	5	2	5	5	2	3	2	4	0	4
4018.4733	4018.4880	-0.0147	6	1	5	0	6	2	8	5	1	4	0	5	2	7
4018.4733	4018.4692	0.0041	6	1	5	1	6	2	8	5	1	4	1	5	2	7
4018.4733	4018.4549	0.0184	6	1	5	1	7	1	8	5	1	4	1	6	1	7
4018.4733	4018.4529	0.0204	6	1	5	1	7	2	9	5	1	4	1	6	2	8
4018.4733	4018.4692	0.0041	6	1	5	2	8	0	8	5	1	4	2	7	0	7
4018.4733	4018.4693	0.0040	6	1	5	2	8	1	8	5	1	4	2	7	1	7
4018.4733	4018.4520	0.0213	6	1	5	2	8	1	9	5	1	4	2	7	1	8
4018.4733	4018.4548	0.0185	6	1	5	2	8	2	8	5	1	4	2	7	2	7
4018.4733	4018.4524	0.0209	6	1	5	2	8	2	9	5	1	4	2	7	2	8
4018.4733	4018.4500	0.0233	6	1	5	2	8	2	10	5	1	4	2	7	2	9
4507.4715	4507.4844	-0.0129	7	1	7	1	7	2	9	6	1	6	1	6	2	8
4507.4715	4507.4358	0.0357	7	1	7	1	8	1	9	6	1	6	1	7	1	8
4507.4715	4507.4584	0.0131	7	1	7	1	8	2	10	6	1	6	1	7	2	9
4507.4715	4507.4631	0.0084	7	1	7	2	8	2	10	6	1	6	2	7	2	9
4507.4715	4507.4851	-0.0136	7	1	7	2	9	0	9	6	1	6	2	8	0	8
4507.4715	4507.4854	-0.0139	7	1	7	2	9	1	9	6	1	6	2	8	1	8
4507.4715	4507.4627	0.0088	7	1	7	2	9	1	10	6	1	6	2	8	1	9
4507.4715	4507.4393	0.0322	7	1	7	2	9	2	9	6	1	6	2	8	2	8
4507.4715	4507.4618	0.0097	7	1	7	2	9	2	10	6	1	6	2	8	2	9
4507.4715	4507.4853	-0.0138	7	1	7	2	9	2	11	6	1	6	2	8	2	10
4539.5329	4539.5793	-0.0464	7	0	7	0	7	1	8	6	0	6	0	6	1	7
4539.5329	4539.5412	-0.0083	7	0	7	1	6	2	8	6	0	6	1	5	2	7
4539.5329	4539.5773	-0.0444	7	0	7	1	7	1	8	6	0	6	1	6	1	7
4539.5329	4539.5734	-0.0405	7	0	7	1	8	0	8	6	0	6	1	7	0	7
4539.5329	4539.5709	-0.0380	7	0	7	1	8	1	8	6	0	6	1	7	1	7
4539.5329	4539.5563	-0.0234	7	0	7	1	8	2	10	6	0	6	1	7	2	9
4539.5329	4539.5302	0.0027	7	0	7	2	8	2	10	6	0	6	2	7	2	9
4539.5329	4539.5459	-0.0130	7	0	7	2	9	1	8	6	0	6	2	8	1	7
4539.5329	4539.5639	-0.0310	7	0	7	2	9	1	10	6	0	6	2	8	1	9
4539.5329	4539.5339	-0.0010	7	0	7	2	9	2	10	6	0	6	2	8	2	9
4539.6654	4539.6817	-0.0163	7	0	7	0	7	0	7	6	0	6	0	6	0	6
4539.6654	4539.6792	-0.0138	7	0	7	0	7	1	7	6	0	6	0	6	1	6
4539.6654	4539.6652	0.0002	7	0	7	0	7	2	9	6	0	6	0	6	2	8
4539.6654	4539.6795	-0.0141	7	0	7	1	7	0	7	6	0	6	1	6	0	6
4539.6654	4539.6770	-0.0116	7	0	7	1	7	1	7	6	0	6	1	6	1	6
4539.6654	4539.6632	0.0022	7	0	7	1	7	2	9	6	0	6	1	6	2	8
4539.6654	4539.6799	-0.0145	7	0	7	2	5	2	7	6	0	6	2	5	2	6

4539.6654	4539.6659	-0.0005	7	0	7	2	9	0	9	6	0	6	2	8	0	8
4539.6654	4539.6634	0.0020	7	0	7	2	9	1	9	6	0	6	2	8	1	8
4539.6654	4539.6502	0.0152	7	0	7	2	9	2	11	6	0	6	2	8	2	10
4602.7758	4602.7674	0.0084	7	2	6	0	7	2	9	6	2	5	0	6	2	8
4602.7758	4602.7673	0.0085	7	2	6	1	7	2	9	6	2	5	1	6	2	8
4602.7758	4602.7924	-0.0166	7	2	6	1	8	1	9	6	2	5	1	7	1	8
4602.7758	4602.7760	-0.0002	7	2	6	1	8	2	10	6	2	5	1	7	2	9
4602.7758	4602.7895	-0.0137	7	2	6	2	7	2	9	6	2	5	2	6	2	8
4602.7758	4602.7776	-0.0018	7	2	6	2	8	2	10	6	2	5	2	8	2	9
4602.7758	4602.7672	0.0086	7	2	6	2	9	1	9	6	2	5	2	8	1	8
4602.7758	4602.7807	-0.0049	7	2	6	2	9	1	10	6	2	5	2	8	1	9
4602.7758	4602.7621	0.0137	7	2	6	2	9	2	10	6	2	5	2	7	2	9
4602.7758	4602.7643	0.0115	7	2	6	2	9	2	11	6	2	5	2	8	2	10
4678.3197	4678.3150	0.0047	7	2	5	0	7	0	7	6	2	4	2	4	2	6
4678.3197	4678.3150	0.0047	7	2	5	0	7	1	7	6	2	4	0	6	1	6
4678.3197	4678.3192	0.0005	7	2	5	0	7	2	9	6	4	2	0	6	2	8
4678.3197	4678.3150	0.0047	7	2	5	1	7	0	7	6	2	4	1	6	0	6
4678.3197	4678.3123	0.0074	7	2	5	1	7	1	7	6	2	4	1	6	1	6
4678.3197	4678.3148	0.0049	7	2	5	1	7	2	9	6	2	4	1	6	2	8
4678.3197	4678.3147	0.0050	7	2	5	2	9	0	9	6	4	2	2	8	0	8
4678.3197	4678.3146	0.0051	7	2	5	2	9	1	9	6	2	4	2	8	1	8
4678.3197	4678.3123	0.0074	7	2	5	2	9	2	7	6	2	4	2	8	2	6
4678.3197	4678.3175	0.0022	7	2	5	2	9	2	11	6	2	4	2	8	2	10
4678.4361	4678.4306	0.0055	7	2	5	0	7	1	8	6	2	4	0	6	1	7
4678.4361	4678.4351	0.0010	7	2	5	0	7	2	8	6	0	6	2	6	2	7
4678.4361	4678.4306	0.0055	7	2	5	1	7	1	8	6	2	4	1	6	1	7
4678.4361	4678.4329	0.0032	7	2	5	1	8	0	8	6	2	4	1	7	0	7
4678.4361	4678.4330	0.0031	7	2	5	1	8	1	8	6	2	4	1	7	1	7
4678.4361	4678.4327	0.0034	7	2	5	1	8	2	10	6	2	4	1	7	2	9
4678.4361	4678.4377	-0.0016	7	2	5	2	8	2	10	6	2	4	2	7	2	9
4678.4361	4678.4332	0.0029	7	2	5	2	9	1	8	6	2	4	2	8	1	7
4678.4361	4678.4334	0.0027	7	2	5	2	9	1	10	6	2	4	2	8	1	9
4678.4361	4678.4331	0.0030	7	2	5	2	9	2	10	6	2	4	2	8	2	9
4678.5555	4678.5492	0.0063	7	2	5	1	6	1	7	6	2	4	1	5	1	6
4678.5555	4678.5496	0.0059	7	2	5	1	8	1	7	6	2	4	1	7	1	6
4678.5555	4678.5485	0.0070	7	2	5	1	8	1	9	6	2	4	1	7	1	8
4678.5555	4678.5500	0.0055	7	2	5	1	8	2	7	6	2	4	1	7	2	6
4678.5555	4678.5524	0.0031	7	2	5	1	8	2	9	6	2	4	1	7	2	8
4678.5555	4678.5636	-0.0081	7	2	5	2	7	2	9	6	2	4	2	7	2	8
4678.5555	4678.5467	0.0088	7	2	5	2	8	1	7	6	2	4	2	7	1	6
4678.5555	4678.5523	0.0032	7	2	5	2	8	1	9	6	2	4	2	7	1	8
4678.5555	4678.5522	0.0033	7	2	5	2	8	2	9	6	0	6	2	6	2	8
4678.5555	4678.5485	0.0070	7	2	5	2	9	2	9	6	0	6	2	8	2	8
4678.6726	4678.6717	0.0009	7	2	5	1	6	2	6	6	2	4	1	5	2	5
4678.6726	4678.6705	0.0021	7	2	5	1	8	2	8	6	2	4	1	7	2	7
4678.6726	4678.6712	0.0014	7	2	5	2	7	1	6	6	2	4	2	6	1	5
4678.6726	4678.6698	0.0028	7	2	5	2	7	1	8	6	2	4	2	6	1	7
4678.6726	4678.6714	0.0012	7	2	5	2	7	2	6	6	0	6	2	6	2	5
4678.6726	4678.6861	-0.0135	7	2	5	2	7	2	8	6	2	4	2	6	2	7
4678.6726	4678.6969	-0.0243	7	4	3	2	6	0	6	6	4	2	2	5	2	5
4678.6726	4678.6701	0.0025	7	4	3	2	8	0	8	6	0	6	2	7	2	7
4679.9005	4679.9550	-0.0545	7	1	6	1	8	1	9	6	1	5	1	7	1	8
4679.9005	4679.9046	-0.0041	7	1	6	1	8	2	8	6	1	5	1	7	2	7

4679.9005	4679.9166	-0.0161	7	1	6	1	8	2	9	6	1	5	1	7	2	8
4679.9005	4679.8937	0.0068	7	1	6	2	7	1	8	6	1	5	2	6	1	7
4679.9005	4679.9549	-0.0544	7	1	6	2	7	2	9	6	1	5	2	6	2	8
4679.9005	4679.9052	-0.0047	7	1	6	2	8	1	8	6	1	5	2	7	1	7
4679.9005	4679.9140	-0.0135	7	1	6	2	8	1	9	6	1	5	2	7	1	8
4679.9005	4679.8991	0.0014	7	1	6	2	8	2	8	6	1	5	2	7	2	7
4679.9005	4679.8888	0.0117	7	1	6	2	8	2	10	6	1	5	2	7	2	9
4679.9005	4679.9154	-0.0149	7	1	6	2	9	2	9	6	1	5	2	8	2	8
4680.0136	4680.0179	-0.0043	7	1	6	0	7	2	9	6	1	5	0	6	2	8
4680.0136	4679.9756	0.0380	7	1	6	1	6	2	8	6	1	5	1	5	2	7
4680.0136	4680.0185	-0.0049	7	1	6	1	7	2	9	6	1	5	1	6	2	8
4680.0136	4679.9735	0.0401	7	1	6	1	8	2	10	6	1	5	1	7	2	9
4680.0136	4680.0301	-0.0165	7	1	6	2	9	0	9	6	1	5	2	8	0	8
4680.0136	4679.9798	0.0338	7	1	6	2	9	1	8	6	1	5	2	8	1	7
4680.0136	4680.0173	-0.0037	7	1	6	2	9	1	9	6	1	5	2	8	1	8
4680.0136	4679.9820	0.0316	7	1	6	2	9	1	10	6	1	5	2	8	1	9
4680.0136	4679.9777	0.0359	7	1	6	2	9	2	10	6	1	5	2	8	2	9
4680.0136	4680.0004	0.0132	7	1	6	2	9	2	11	6	1	5	2	8	2	10
5146.1783	5146.2015	-0.0232	8	1	8	1	8	2	10	7	1	7	1	7	2	9
5146.1783	5146.1473	0.0310	8	1	8	1	9	1	10	7	1	7	1	8	1	9
5146.1783	5146.1727	0.0056	8	1	8	1	9	2	11	7	1	7	1	8	2	10
5146.1783	5146.1752	0.0031	8	1	8	2	9	2	11	7	1	7	2	8	2	10
5146.1783	5146.2021	-0.0238	8	1	8	2	10	0	10	7	1	7	2	9	0	9
5146.1783	5146.2023	-0.0240	8	1	8	2	10	1	10	7	1	7	2	9	1	9
5146.1783	5146.1765	0.0018	8	1	8	2	10	1	11	7	1	7	2	9	1	10
5146.1783	5146.1501	0.0282	8	1	8	2	10	2	10	7	1	7	2	9	2	9
5146.1783	5146.1755	0.0028	8	1	8	2	10	2	11	7	1	7	2	9	2	10
5146.1783	5146.2019	-0.0236	8	1	8	2	10	2	12	7	1	7	2	9	2	11
5171.5191	5171.5128	0.0063	8	0	8	0	8	2	7	7	0	7	0	7	2	6
5171.5191	5171.4859	0.0332	8	0	8	0	8	2	9	7	0	7	0	7	2	8
5171.5191	5171.5062	0.0129	8	0	8	1	7	0	7	7	0	7	1	6	2	6
5171.5191	5171.5157	0.0034	8	0	8	1	9	2	9	7	0	7	1	8	2	8
5171.5191	5171.5599	-0.0408	8	0	8	2	7	0	7	7	0	7	2	6	0	6
5171.5191	5171.4994	0.0197	8	0	8	2	8	1	7	7	0	7	2	7	1	6
5171.5191	5171.5095	0.0096	8	0	8	2	8	1	9	7	0	7	2	7	1	8
5171.5191	5171.5080	0.0111	8	0	8	2	9	0	9	7	0	7	2	8	0	8
5171.6006	5171.6004	0.0002	8	0	8	1	7	1	8	7	0	7	1	6	1	7
5171.6006	5171.5777	0.0229	8	0	8	1	7	2	8	7	0	7	1	6	2	7
5171.6006	5171.6000	0.0006	8	0	8	1	9	1	8	7	0	7	1	8	1	7
5171.6006	5171.6100	-0.0094	8	0	8	1	9	1	10	7	0	7	1	8	1	9
5171.6006	5171.5922	0.0084	8	0	8	1	9	2	10	7	0	7	1	8	2	9
5171.6006	5171.6275	-0.0269	8	0	8	2	7	1	8	7	0	7	2	6	1	7
5171.6006	5171.5739	0.0267	8	0	8	2	8	2	10	7	0	7	2	7	2	9
5171.6006	5171.5927	0.0079	8	0	8	2	9	1	10	7	0	7	2	8	1	9
5171.6006	5171.5810	0.0196	8	0	8	2	9	2	10	7	0	7	2	8	2	9
5171.6006	5171.6051	-0.0045	8	0	8	2	10	2	10	7	0	7	2	9	2	9
5171.6972	5171.7070	-0.0098	8	0	8	0	8	1	9	7	0	7	0	7	1	8
5171.6972	5171.7061	-0.0089	8	0	8	1	8	1	9	7	0	7	1	7	1	8
5171.6972	5171.6952	0.0020	8	0	8	1	9	0	9	7	0	7	1	8	0	8
5171.6972	5171.6949	0.0023	8	0	8	1	9	1	9	7	0	7	1	8	1	8
5171.6972	5171.6877	0.0095	8	0	8	1	9	2	11	7	0	7	1	8	2	10
5171.6972	5171.6611	0.0361	8	0	8	2	9	2	11	7	0	7	2	8	2	10
5171.6972	5171.6884	0.0088	8	0	8	2	10	1	9	7	0	7	2	9	1	8

5171.6972	5171.6944	0.0028	8	0	8	2	10	1	11	7	0	7	2	9	1	10
5171.6972	5171.7082	-0.0110	8	0	8	2	10	2	9	7	0	7	2	9	2	8
5171.6972	5171.6835	0.0137	8	0	8	2	10	2	11	7	0	7	2	9	2	10
5171.7907	5171.7907	0.0000	8	0	8	0	8	1	8	7	0	7	0	7	1	7
5171.7907	5171.7815	0.0092	8	0	8	0	8	2	10	7	0	7	0	7	2	9
5171.7907	5171.7909	-0.0002	8	0	8	1	8	0	8	7	0	7	1	7	0	7
5171.7907	5171.7897	0.0010	8	0	8	1	8	1	8	7	0	7	1	7	1	7
5171.7907	5171.7804	0.0103	8	0	8	1	8	2	10	7	0	7	1	7	2	9
5171.7907	5171.7903	0.0004	8	0	8	2	6	2	8	7	0	7	2	5	2	7
5171.7907	5171.7815	0.0092	8	0	8	2	10	0	10	7	0	7	2	9	0	9
5171.7907	5171.7807	0.0100	8	0	8	2	10	1	10	7	0	7	2	9	1	9
5171.7907	5171.7907	0.0000	8	0	8	2	10	2	8	7	0	7	2	9	2	7
5171.7907	5171.7723	0.0184	8	0	8	2	10	2	12	7	0	7	2	9	2	11
5255.0000	5255.0133	-0.0133	8	2	7	0	8	2	10	7	2	6	0	7	2	9
5255.0000	5255.0132	-0.0132	8	2	7	1	8	2	10	7	2	6	1	7	2	9
5255.0000	5255.0058	-0.0058	8	2	7	1	9	1	10	7	2	6	1	8	1	9
5255.0000	5255.0054	-0.0054	8	2	7	1	9	2	11	7	2	6	1	8	2	10
5255.0000	5255.0024	-0.0024	8	2	7	2	8	2	10	7	2	6	2	7	2	9
5255.0000	5255.0066	-0.0066	8	2	7	2	9	2	11	7	2	6	2	8	2	10
5255.0000	5255.0132	-0.0132	8	2	7	2	10	1	10	7	2	6	2	9	1	9
5255.0000	5255.0096	-0.0096	8	2	7	2	10	1	11	7	2	6	2	9	1	10
5255.0000	5254.9879	0.0121	8	2	7	2	10	2	11	7	2	6	2	9	2	10
5255.0000	5255.0092	-0.0092	8	2	7	2	10	2	12	7	2	6	2	9	2	11
5285.9140	5285.8904	0.0236	8	4	5	0	8	1	8	7	4	4	0	7	1	7
5285.9140	5285.9000	0.0140	8	4	5	0	8	2	10	7	4	4	0	7	2	9
5285.9140	5285.8905	0.0235	8	4	5	1	8	0	8	7	4	4	1	7	0	7
5285.9140	5285.8915	0.0225	8	4	5	1	8	1	8	7	4	4	1	7	1	7
5285.9140	5285.9018	0.0122	8	4	5	1	8	2	10	7	4	4	1	7	2	9
5285.9140	5285.8882	0.0258	8	4	5	2	6	2	8	7	4	4	2	5	2	7
5285.9140	5285.9022	0.0118	8	4	5	2	10	0	10	7	4	4	2	9	0	9
5285.9140	5285.9025	0.0115	8	4	5	2	10	1	10	7	4	4	2	9	1	9
5285.9140	5285.8917	0.0223	8	4	5	2	10	2	8	7	4	4	2	9	2	7
5285.9140	5285.9120	0.0020	8	4	5	2	10	2	12	7	4	4	2	9	2	11
5286.0333	5285.9929	0.0404	8	4	5	0	8	1	9	7	2	6	0	7	1	8
5286.0333	5285.9962	0.0371	8	4	5	0	8	2	9	7	4	4	0	7	2	8
5286.0333	5285.9929	0.0404	8	4	5	1	8	1	9	7	4	4	1	7	1	8
5286.0333	5286.0159	0.0174	8	4	5	1	9	0	9	7	4	4	1	8	0	8
5286.0333	5286.0148	0.0185	8	4	5	1	9	1	9	7	4	4	1	8	1	8
5286.0333	5286.0174	0.0159	8	4	5	1	9	2	11	7	4	4	1	8	2	10
5286.0333	5286.0131	0.0202	8	4	5	2	10	1	9	7	4	4	2	9	1	8
5286.0333	5286.0118	0.0215	8	4	5	2	10	1	11	7	4	4	2	9	1	10
5286.0333	5285.9944	0.0389	8	4	5	2	10	2	9	7	4	4	2	9	2	8
5286.0333	5286.0145	0.0188	8	4	5	2	10	2	11	7	4	4	2	9	2	10
5286.1334	5286.1311	0.0023	8	4	5	1	7	1	8	7	4	4	1	6	1	7
5286.1334	5286.1279	0.0055	8	4	5	1	9	1	8	7	4	4	1	8	1	7
5286.1334	5286.1173	0.0161	8	4	5	1	9	1	10	7	4	4	1	8	1	9
5286.1334	5286.1247	0.0087	8	4	5	1	9	2	8	7	4	4	1	8	2	7
5286.1334	5286.1371	-0.0037	8	4	5	1	9	2	10	7	4	4	1	8	2	9
5286.1334	5286.1385	-0.0051	8	4	5	2	8	2	10	7	4	4	2	7	2	9
5286.1334	5286.1293	0.0041	8	4	5	2	9	1	8	7	4	4	2	8	1	7
5286.1334	5286.1383	-0.0049	8	4	5	2	9	1	10	7	4	4	2	8	1	9
5286.1334	5286.1291	0.0043	8	4	5	2	9	2	8	7	4	4	2	8	2	7
5286.1334	5286.1166	0.0168	8	4	5	2	10	2	10	7	4	4	2	9	2	9

5286.2508	5286.2468	0.0040	8	2	7	2	8	2	9	7	4	4	2	7	2	8
5286.2508	5286.2467	0.0041	8	4	5	1	7	2	7	7	4	4	1	6	0	6
5286.2508	5286.2361	0.0147	8	4	5	1	9	2	9	7	4	4	1	8	2	8
5286.2508	5286.2042	0.0466	8	4	5	2	8	1	7	7	4	4	2	7	1	6
5286.2508	5286.1897	0.0611	8	4	5	2	8	1	9	7	6	2	2	7	1	8
5286.2508	5286.2504	0.0004	8	4	5	2	8	2	7	7	4	4	0	7	2	6
5286.2508	5286.2394	0.0114	8	4	5	2	9	2	9	7	6	2	2	8	0	8
5286.5881	5286.6041	-0.0160	8	4	4	0	8	1	8	7	4	3	0	7	1	7
5286.5881	5286.5947	-0.0066	8	4	4	1	8	0	8	7	4	3	1	7	0	7
5286.5881	5286.5895	-0.0014	8	4	4	1	8	1	8	7	4	3	1	7	1	7
5286.5881	5286.6057	-0.0176	8	4	4	1	8	2	10	7	4	3	1	7	2	9
5286.5881	5286.5833	0.0048	8	4	4	2	6	2	8	7	2	5	2	5	2	7
5286.5881	5286.6019	-0.0138	8	4	4	2	10	1	10	7	4	3	2	9	1	9
5286.5881	5286.5931	-0.0050	8	4	4	2	10	2	8	7	4	3	2	9	2	7
5286.5881	5286.6116	-0.0235	8	4	4	2	10	2	12	7	4	3	2	9	2	11
5286.5881	5286.6013	-0.0132	8	6	2	0	8	2	10	7	4	3	0	7	2	9
5286.5881	5286.6016	-0.0135	8	6	2	2	10	0	10	7	4	3	2	9	0	9
5286.7236	5286.6985	0.0251	8	2	6	2	8	2	9	7	4	3	0	7	2	8
5286.7236	5286.7480	-0.0244	8	4	4	0	8	1	9	7	4	3	0	7	1	8
5286.7236	5286.6948	0.0288	8	4	4	1	8	1	9	7	4	3	1	7	1	8
5286.7236	5286.7184	0.0052	8	4	4	1	9	0	9	7	4	3	1	8	0	8
5286.7236	5286.7173	0.0063	8	4	4	1	9	1	9	7	4	3	1	8	1	8
5286.7236	5286.7198	0.0038	8	4	4	1	9	2	11	7	4	3	1	8	2	10
5286.7236	5286.7460	-0.0224	8	4	4	2	10	1	9	7	4	3	2	9	1	8
5286.7236	5286.7145	0.0091	8	4	4	2	10	1	11	7	4	3	2	9	1	10
5286.7236	5286.6968	0.0268	8	4	4	2	10	2	9	7	4	3	2	9	2	8
5286.7236	5286.7170	0.0066	8	4	4	2	10	2	11	7	4	3	2	9	2	10
5286.8293	5286.8445	-0.0152	8	2	6	2	8	2	10	7	4	3	2	7	2	9
5286.8293	5286.8222	0.0071	8	2	6	2	10	2	10	7	4	3	2	9	2	9
5286.8293	5286.8369	-0.0076	8	4	4	1	7	1	8	7	4	3	1	6	1	7
5286.8293	5286.8337	-0.0044	8	4	4	1	9	1	8	7	4	3	1	8	1	7
5286.8293	5286.8227	0.0066	8	4	4	1	9	1	10	7	4	3	1	8	1	9
5286.8293	5286.8306	-0.0013	8	4	4	1	9	2	8	7	4	3	1	8	2	7
5286.8293	5286.8432	-0.0139	8	4	4	1	9	2	10	7	4	3	1	8	2	9
5286.8293	5286.8349	-0.0056	8	4	4	2	9	1	8	7	4	3	2	8	1	7
5286.8293	5286.8443	-0.0150	8	4	4	2	9	1	10	7	4	3	2	8	1	9
5286.8293	5286.8349	-0.0056	8	4	4	2	9	2	8	7	4	3	2	8	2	7
5286.9500	5286.9483	0.0017	8	0	8	2	9	2	9	7	2	5	2	8	2	8
5286.9500	5286.9603	-0.0103	8	2	6	2	8	2	7	7	4	3	2	7	2	6
5286.9500	5286.9573	-0.0073	8	4	4	1	7	2	7	7	4	3	1	6	2	6
5286.9500	5286.9451	0.0049	8	4	4	1	9	2	9	7	4	3	1	8	2	8
5286.9500	5286.9517	-0.0017	8	4	4	2	8	1	9	7	4	3	2	7	1	8
5286.9500	5286.9881	-0.0381	8	4	4	2	8	2	9	7	4	3	2	7	2	8
5286.9500	5286.9631	-0.0131	8	6	2	2	8	1	7	7	4	3	2	7	1	6
5287.0824	5287.0777	0.0047	8	3	6	0	8	1	8	7	3	5	0	7	1	7
5287.0824	5287.0806	0.0018	8	3	6	0	8	2	10	7	3	5	0	7	2	9
5287.0824	5287.0777	0.0047	8	3	6	1	8	0	8	7	3	5	1	7	0	7
5287.0824	5287.0771	0.0053	8	3	6	1	8	1	8	7	3	5	1	7	1	7
5287.0824	5287.0796	0.0028	8	3	6	1	8	2	10	7	3	5	1	7	2	9
5287.0824	5287.0776	0.0048	8	3	6	2	7	2	8	7	3	5	2	6	2	7
5287.0824	5287.0797	0.0027	8	3	6	2	10	0	10	7	5	3	2	9	0	9
5287.0824	5287.0798	0.0026	8	3	6	2	10	1	10	7	3	5	2	9	1	9
5287.0824	5287.0773	0.0051	8	3	6	2	10	2	8	7	3	5	2	9	2	7

5287.0824	5287.0822	0.0002	8	3	6	2	10	2	12	7	3	5	2	9	2	11
5287.1494	5287.1410	0.0084	8	1	8	2	8	2	9	7	1	7	2	7	2	8
5287.1494	5287.1398	0.0096	8	3	6	0	8	1	9	7	3	5	0	7	1	8
5287.1494	5287.1443	0.0051	8	3	6	1	7	2	9	7	3	5	1	6	2	8
5287.1494	5287.1387	0.0107	8	3	6	1	8	1	9	7	3	5	1	7	1	8
5287.1494	5287.1438	0.0056	8	3	6	1	9	1	9	7	3	5	1	8	1	8
5287.1494	5287.1452	0.0042	8	3	6	1	9	2	11	7	3	5	1	8	2	10
5287.1494	5287.1534	-0.0040	8	3	6	2	9	2	11	7	3	5	2	8	2	10
5287.1494	5287.1426	0.0068	8	3	6	2	10	1	9	7	3	5	2	9	1	8
5287.1494	5287.1418	0.0076	8	3	6	2	10	1	11	7	3	5	2	9	1	10
5287.1494	5287.1433	0.0061	8	3	6	2	10	2	11	7	3	5	2	9	2	10
5287.2122	5287.2067	0.0055	8	3	6	1	7	1	8	7	3	5	1	6	1	7
5287.2122	5287.2053	0.0069	8	3	6	1	8	2	8	7	3	5	1	7	2	7
5287.2122	5287.2071	0.0051	8	3	6	1	9	1	8	7	3	5	1	8	1	7
5287.2122	5287.2048	0.0074	8	3	6	1	9	1	10	7	3	5	1	8	1	9
5287.2122	5287.2097	0.0025	8	3	6	1	9	2	10	7	3	5	1	8	2	9
5287.2122	5287.2090	0.0032	8	3	6	2	8	1	8	7	3	5	2	7	1	7
5287.2122	5287.2101	0.0021	8	3	6	2	8	2	10	7	3	5	2	7	2	9
5287.2122	5287.2104	0.0018	8	3	6	2	9	1	10	7	3	5	2	8	1	9
5287.2122	5287.2209	-0.0087	8	3	6	2	9	2	10	7	3	5	2	8	2	9
5287.2122	5287.2040	0.0082	8	3	6	2	10	2	10	7	1	7	2	9	2	9
5287.2750	5287.2742	0.0008	8	1	8	2	8	2	7	7	1	7	2	7	2	6
5287.2750	5287.2714	0.0036	8	1	8	2	9	2	9	7	1	7	2	8	2	8
5287.2750	5287.2722	0.0028	8	3	6	1	7	2	7	7	3	5	1	6	2	6
5287.2750	5287.2696	0.0054	8	3	6	1	9	2	9	7	3	5	1	8	2	8
5287.2750	5287.2715	0.0035	8	3	6	2	7	2	7	7	3	5	2	6	2	6
5287.2750	5287.2759	-0.0009	8	3	6	2	8	1	7	7	3	5	2	7	1	6
5287.2750	5287.2735	0.0015	8	3	6	2	8	1	9	7	3	5	2	7	1	8
5287.2750	5287.2834	-0.0084	8	3	6	2	8	2	9	7	3	5	2	7	2	8
5302.5247	5302.5230	0.0017	8	3	5	0	8	1	8	7	3	4	0	7	1	7
5302.5247	5302.5296	-0.0049	8	3	5	0	8	2	10	7	3	4	0	7	2	9
5302.5247	5302.5229	0.0018	8	3	5	1	8	0	8	7	3	4	1	7	0	7
5302.5247	5302.5227	0.0020	8	3	5	1	8	1	8	7	3	4	1	7	1	7
5302.5247	5302.5293	-0.0046	8	3	5	1	8	2	10	7	3	4	1	7	2	9
5302.5247	5302.5235	0.0012	8	3	5	2	6	2	8	7	5	2	2	5	2	7
5302.5247	5302.5293	-0.0046	8	3	5	2	10	0	10	7	3	4	2	9	0	9
5302.5247	5302.5293	-0.0046	8	3	5	2	10	1	10	7	3	4	2	9	1	9
5302.5247	5302.5227	0.0020	8	3	5	2	10	2	8	7	3	4	2	9	2	7
5302.5247	5302.5356	-0.0109	8	3	5	2	10	2	12	7	3	4	2	9	2	11
5302.6306	5302.6252	0.0054	8	3	5	0	8	1	9	7	3	4	0	7	1	8
5302.6306	5302.6252	0.0054	8	3	5	1	8	1	9	7	3	4	1	7	1	8
5302.6306	5302.6380	-0.0074	8	3	5	1	9	0	9	7	3	4	1	8	0	8
5302.6306	5302.6378	-0.0072	8	3	5	1	9	1	9	7	3	4	1	8	1	8
5302.6306	5302.6383	-0.0077	8	3	5	1	9	2	11	7	3	4	1	8	2	10
5302.6306	5302.6283	0.0023	8	3	5	2	8	2	9	7	1	6	2	7	2	8
5302.6306	5302.6638	-0.0332	8	3	5	2	9	2	11	7	3	4	2	8	2	10
5302.6306	5302.6376	-0.0070	8	3	5	2	10	1	9	7	3	4	2	9	1	8
5302.6306	5302.6374	-0.0068	8	3	5	2	10	1	11	7	3	4	2	9	1	10
5302.6306	5302.6378	-0.0072	8	3	5	2	10	2	11	7	3	4	2	9	2	10
5302.7454	5302.7470	-0.0016	8	3	5	1	7	1	8	7	3	4	1	6	1	7
5302.7454	5302.7466	-0.0012	8	3	5	1	9	1	8	7	3	4	1	8	1	7
5302.7454	5302.7401	0.0053	8	3	5	1	9	1	10	7	3	4	1	8	1	9
5302.7454	5302.7462	-0.0008	8	3	5	1	9	2	8	7	3	4	1	8	2	7

5302.7454	5302.7531	-0.0077	8	3	5	1	9	2	10	7	3	4	1	8	2	9
5302.7454	5302.7530	-0.0076	8	3	5	2	8	2	10	7	3	4	2	7	2	9
5302.7454	5302.7464	-0.0010	8	3	5	2	9	1	8	7	3	4	2	8	1	7
5302.7454	5302.7530	-0.0076	8	3	5	2	9	1	10	7	3	4	2	8	1	9
5302.7454	5302.7794	-0.0340	8	3	5	2	9	2	10	7	3	4	2	8	2	9
5302.7454	5302.7401	0.0053	8	3	5	2	10	2	10	7	3	4	2	9	2	9
5302.8594	5302.8618	-0.0024	8	3	5	1	7	2	7	7	1	6	1	6	2	6
5302.8594	5302.8550	0.0044	8	3	5	1	9	2	9	7	3	4	1	8	2	8
5302.8594	5302.8392	0.0202	8	3	5	2	7	2	7	7	5	2	2	6	0	6
5302.8594	5302.8627	-0.0033	8	3	5	2	8	1	7	7	5	2	2	7	1	6
5302.8594	5302.8558	0.0036	8	3	5	2	8	1	9	7	3	4	2	7	1	8
5302.8594	5302.8622	-0.0028	8	3	5	2	8	2	7	7	1	6	2	7	2	6
5302.8594	5302.8554	0.0040	8	3	5	2	9	2	9	7	1	6	2	8	2	8
5302.8594	5302.8819	-0.0225	8	5	3	2	8	2	9	7	3	4	2	7	2	8
5336.7941	5336.8150	-0.0209	8	1	7	1	7	2	8	7	1	6	1	6	2	7
5336.7941	5336.8167	-0.0226	8	1	7	1	9	2	9	7	1	6	1	8	2	8
5336.7941	5336.8545	-0.0604	8	1	7	1	9	2	10	7	1	6	1	8	2	9
5336.7941	5336.8109	-0.0168	8	1	7	2	8	1	9	7	1	6	2	7	1	8
5336.7941	5336.8119	-0.0178	8	1	7	2	9	1	8	7	1	6	2	8	1	7
5336.7941	5336.8535	-0.0594	8	1	7	2	9	1	10	7	1	6	2	8	1	9
5336.7941	5336.8138	-0.0197	8	1	7	2	9	2	9	7	1	6	2	8	2	8
5336.7941	5336.7873	0.0068	8	1	7	2	9	2	10	7	1	6	2	8	2	9
5336.7941	5336.8565	-0.0624	8	1	7	2	9	2	11	7	1	6	2	8	2	10
5336.7941	5336.8543	-0.0602	8	1	7	2	10	2	10	7	1	6	2	9	2	9
5336.8562	5336.8741	-0.0179	8	1	7	0	8	2	9	7	1	6	0	7	2	8
5336.8562	5336.8728	-0.0166	8	1	7	1	8	2	8	7	1	6	1	7	2	7
5336.8562	5336.8745	-0.0183	8	1	7	1	8	2	9	7	1	6	1	7	2	8
5336.8562	5336.8894	-0.0332	8	1	7	1	9	1	10	7	1	6	1	8	1	9
5336.8562	5336.8890	-0.0328	8	1	7	2	8	2	10	7	1	6	2	7	2	9
5336.8562	5336.8737	-0.0175	8	1	7	2	9	1	9	7	1	6	2	8	1	8
5336.9500	5336.9730	-0.0230	8	1	7	0	8	1	9	7	1	6	0	7	1	8
5336.9500	5336.9329	0.0171	8	1	7	1	7	2	9	7	1	6	1	6	2	8
5336.9500	5336.9729	-0.0229	8	1	7	1	8	1	9	7	1	6	1	7	1	8
5336.9500	5336.9703	-0.0203	8	1	7	1	9	0	9	7	1	6	1	8	0	8
5336.9500	5336.9343	0.0157	8	1	7	1	9	1	9	7	1	6	1	8	1	8
5336.9500	5336.9318	0.0182	8	1	7	1	9	2	11	7	1	6	1	8	2	10
5336.9500	5336.9354	0.0146	8	1	7	2	10	1	9	7	1	6	2	9	1	8
5336.9500	5336.9366	0.0134	8	1	7	2	10	1	11	7	1	6	2	9	1	10
5336.9500	5336.9341	0.0159	8	1	7	2	10	2	11	7	1	6	2	9	2	10
5336.9500	5336.9789	-0.0289	8	1	7	2	10	2	12	7	1	6	2	9	2	11
5337.0111	5336.9949	0.0162	8	1	7	0	8	2	10	7	1	6	0	7	2	9
5337.0111	5336.9951	0.0160	8	1	7	1	8	2	10	7	1	6	1	7	2	9
5337.0111	5336.9729	0.0382	8	1	7	2	7	2	9	7	1	6	2	6	2	8
5337.0111	5337.0033	0.0078	8	1	7	2	10	0	10	7	1	6	2	9	0	9
5337.0111	5336.9946	0.0165	8	1	7	2	10	1	10	7	1	6	2	9	1	9
5337.0111	5336.9718	0.0393	8	1	7	2	10	2	9	7	1	6	2	9	2	8
5357.3644	5357.3545	0.0099	8	2	6	0	8	0	8	7	2	5	0	7	0	7
5357.3644	5357.3545	0.0099	8	2	6	0	8	1	8	7	2	5	0	7	1	7
5357.3644	5357.3546	0.0098	8	2	6	1	8	0	8	7	2	5	1	7	0	7
5357.3644	5357.3520	0.0124	8	2	6	1	8	1	8	7	2	5	1	7	1	7
5357.3644	5357.3501	0.0143	8	2	6	1	8	2	10	7	2	5	1	7	2	9
5357.3644	5357.3503	0.0141	8	2	6	2	10	1	10	7	2	5	2	9	1	9
5357.3644	5357.3520	0.0124	8	2	6	2	10	2	8	7	2	5	2	9	2	7

5357.3644	5357.3492	0.0152	8	2	6	2	10	2	12	7	2	5	2	9	2	11
5357.3644	5357.3546	0.0098	8	4	4	0	8	2	10	7	2	5	0	7	2	9
5357.3644	5357.3502	0.0142	8	4	4	2	10	0	10	7	2	5	2	9	0	9
5357.4519	5357.4413	0.0106	8	2	6	0	8	1	9	7	2	5	0	7	1	8
5357.4519	5357.4413	0.0106	8	2	6	1	8	1	9	7	2	5	1	7	1	8
5357.4519	5357.4363	0.0156	8	2	6	1	9	0	9	7	2	5	1	8	0	8
5357.4519	5357.4359	0.0160	8	2	6	1	9	1	9	7	2	5	1	8	1	8
5357.4519	5357.4367	0.0152	8	2	6	1	9	2	11	7	2	5	1	8	2	10
5357.4519	5357.4250	0.0269	8	2	6	2	9	2	11	7	2	5	2	8	2	10
5357.4519	5357.4355	0.0164	8	2	6	2	10	1	9	7	2	5	2	9	1	8
5357.4519	5357.4351	0.0168	8	2	6	2	10	1	11	7	2	5	2	9	1	10
5357.4519	5357.4359	0.0160	8	2	6	2	10	2	11	7	2	5	2	9	2	10
5357.4519	5357.4438	0.0081	8	4	4	0	8	2	9	7	2	5	0	7	2	8
5357.5253	5357.5136	0.0117	8	0	8	2	8	2	10	7	2	5	2	7	2	9
5357.5253	5357.5224	0.0029	8	0	8	2	10	2	10	7	2	5	2	9	2	9
5357.5253	5357.5206	0.0047	8	2	6	1	7	1	8	7	2	5	1	6	1	7
5357.5253	5357.5197	0.0056	8	2	6	1	9	1	8	7	2	5	1	8	1	7
5357.5253	5357.5224	0.0029	8	2	6	1	9	1	10	7	2	5	1	8	1	9
5357.5253	5357.5187	0.0066	8	2	6	1	9	2	8	7	2	5	1	8	2	7
5357.5253	5357.5184	0.0069	8	2	6	1	9	2	10	7	2	5	1	8	2	9
5357.5253	5357.5170	0.0083	8	2	6	2	9	1	8	7	2	5	2	8	1	7
5357.5253	5357.5186	0.0067	8	2	6	2	9	1	10	7	2	5	2	8	1	9
5357.5253	5357.5188	0.0065	8	2	6	2	9	2	10	7	2	5	2	8	2	9
5357.6183	5357.6043	0.0140	8	0	8	2	8	2	7	7	2	5	2	7	2	6
5357.6183	5357.6070	0.0113	8	0	8	2	8	2	9	7	2	5	2	7	2	8
5357.6183	5357.6034	0.0149	8	2	6	1	7	2	7	7	2	5	1	6	2	6
5357.6183	5357.6064	0.0119	8	2	6	1	9	2	9	7	2	5	1	8	2	8
5357.6183	5357.6051	0.0132	8	2	6	2	8	1	7	7	2	5	2	7	1	6
5357.6183	5357.6084	0.0099	8	2	6	2	8	1	9	7	2	5	2	7	1	8
5357.6183	5357.6440	-0.0257	8	6	2	2	7	0	7	7	4	3	2	6	0	6
5357.6183	5357.6074	0.0109	8	6	2	2	9	0	9	7	4	3	2	8	0	8
5783.6502	5783.6820	-0.0318	9	1	9	1	9	2	11	8	1	8	1	8	2	10
5783.6502	5783.6261	0.0241	9	1	9	1	10	1	11	8	1	8	1	9	1	10
5783.6502	5783.6524	-0.0022	9	1	9	1	10	2	12	8	1	8	1	9	2	11
5783.6502	5783.6542	-0.0040	9	1	9	2	10	2	12	8	1	8	2	9	2	11
5783.6502	5783.6826	-0.0324	9	1	9	2	11	0	11	8	1	8	2	10	0	10
5783.6502	5783.6827	-0.0325	9	1	9	2	11	1	11	8	1	8	2	10	1	10
5783.6502	5783.6559	-0.0057	9	1	9	2	11	1	12	8	1	8	2	10	1	11
5783.6502	5783.6286	0.0216	9	1	9	2	11	2	11	8	1	8	2	10	2	10
5783.6502	5783.6550	-0.0048	9	1	9	2	11	2	12	8	1	8	2	10	2	11
5783.6502	5783.6823	-0.0321	9	1	9	2	11	2	13	8	1	8	2	10	2	12
5802.5850	5802.5817	0.0033	9	0	9	0	9	2	8	8	0	8	0	8	2	7
5802.5850	5802.5651	0.0199	9	0	9	0	9	2	10	8	0	8	0	8	2	9
5802.5850	5802.5819	0.0031	9	0	9	1	8	2	8	8	0	8	1	7	0	7
5802.5850	5802.5879	-0.0029	9	0	9	1	10	2	10	8	0	8	1	9	2	9
5802.5850	5802.6055	-0.0205	9	0	9	2	8	0	8	8	0	8	2	7	0	7
5802.5850	5802.5763	0.0087	9	0	9	2	9	1	8	8	0	8	2	8	1	7
5802.5850	5802.5827	0.0023	9	0	9	2	9	1	10	8	0	8	2	8	1	9
5802.5850	5802.5841	0.0009	9	0	9	2	10	0	10	8	0	8	2	9	0	9
5802.6660	5802.6586	0.0074	9	0	9	1	8	1	9	8	0	8	1	7	1	8
5802.6660	5802.6582	0.0078	9	0	9	1	10	1	9	8	0	8	1	9	1	8
5802.6660	5802.6646	0.0014	9	0	9	1	10	1	11	8	0	8	1	9	1	10
5802.6660	5802.6651	0.0009	9	0	9	1	10	2	9	8	0	8	1	9	2	8

5802.6660	5802.6528	0.0132	9	0	9	1	10	2	11	8	0	8	1	9	2	10
5802.6660	5802.6356	0.0304	9	0	9	2	9	2	11	8	0	8	2	8	2	10
5802.6660	5802.6532	0.0128	9	0	9	2	10	1	9	8	0	8	2	9	1	8
5802.6660	5802.6536	0.0124	9	0	9	2	10	1	11	8	0	8	2	9	1	10
5802.6660	5802.6490	0.0170	9	0	9	2	10	2	11	8	0	8	2	9	2	10
5802.6660	5802.6637	0.0023	9	0	9	2	11	2	11	8	0	8	2	10	2	10
5802.7404	5802.7438	-0.0034	9	0	9	0	9	1	10	8	0	8	0	8	1	9
5802.7404	5802.7191	0.0213	9	0	9	1	8	2	10	8	0	8	1	7	2	9
5802.7404	5802.7438	-0.0034	9	0	9	1	9	1	10	8	0	8	1	8	1	9
5802.7404	5802.7337	0.0067	9	0	9	1	10	0	10	8	0	8	1	9	0	9
5802.7404	5802.7341	0.0063	9	0	9	1	10	1	10	8	0	8	1	9	1	9
5802.7404	5802.7302	0.0102	9	0	9	1	10	2	12	8	0	8	1	9	2	11
5802.7404	5802.7117	0.0287	9	0	9	2	10	2	12	8	0	8	2	9	2	11
5802.7404	5802.7331	0.0073	9	0	9	2	11	1	10	8	0	8	2	10	1	9
5802.7404	5802.7359	0.0045	9	0	9	2	11	1	12	8	0	8	2	10	1	11
5802.7404	5802.7307	0.0097	9	0	9	2	11	2	12	8	0	8	2	10	2	11
5802.8160	5802.8133	0.0027	9	0	9	0	9	1	9	8	0	8	0	8	1	8
5802.8160	5802.8074	0.0086	9	0	9	0	9	2	11	8	0	8	0	8	2	10
5802.8160	5802.8135	0.0025	9	0	9	1	9	0	9	8	0	8	1	8	0	8
5802.8160	5802.8129	0.0031	9	0	9	1	9	1	9	8	0	8	1	8	1	8
5802.8160	5802.8069	0.0091	9	0	9	1	9	2	11	8	0	8	1	8	2	10
5802.8160	5802.8131	0.0029	9	0	9	2	7	2	9	8	0	8	2	6	2	8
5802.8160	5802.8076	0.0084	9	0	9	2	11	0	11	8	0	8	2	10	0	10
5802.8160	5802.8072	0.0088	9	0	9	2	11	1	11	8	0	8	2	10	1	10
5802.8160	5802.8136	0.0024	9	0	9	2	11	2	9	8	0	8	2	10	2	8
5802.8160	5802.8016	0.0144	9	0	9	2	11	2	13	8	0	8	2	10	2	12
5905.2935	5905.2725	0.0210	9	2	8	1	9	2	11	8	2	7	1	8	2	10
5905.2935	5905.2425	0.0510	9	2	8	1	10	1	11	8	2	7	1	9	1	10
5905.2935	5905.2534	0.0401	9	2	8	1	10	2	12	8	2	7	1	9	2	11
5905.2935	5905.2384	0.0551	9	2	8	2	9	2	11	8	2	7	2	8	2	10
5905.2935	5905.2330	0.0605	9	2	8	2	10	1	11	8	2	7	2	9	1	10
5905.2935	5905.2541	0.0394	9	2	8	2	10	2	12	8	2	7	2	9	2	11
5905.2935	5905.2726	0.0209	9	2	8	2	11	1	11	8	2	7	2	10	1	10
5905.2935	5905.2573	0.0362	9	2	8	2	11	1	12	8	2	7	2	10	1	11
5905.2935	5905.2357	0.0578	9	2	8	2	11	2	12	8	2	7	2	10	2	11
5905.2935	5905.2682	0.0253	9	2	8	2	11	2	13	8	2	7	2	10	2	12
5948.5947	5948.6246	-0.0299	9	3	7	1	10	1	11	8	3	6	1	9	1	10
5948.5947	5948.6246	-0.0299	9	3	7	1	10	2	11	8	3	6	1	9	2	10
5948.5947	5948.5803	0.0144	9	3	7	1	10	2	12	8	3	6	1	9	2	11
5948.5947	5948.6238	-0.0291	9	3	7	2	9	2	11	8	3	6	2	8	2	10
5948.5947	5948.6245	-0.0298	9	3	7	2	10	1	11	8	3	6	2	9	1	10
5948.5947	5948.6255	-0.0308	9	3	7	2	10	2	11	8	3	6	2	9	2	10
5948.5947	5948.5808	0.0139	9	3	7	2	10	2	12	8	3	6	2	9	2	11
5948.5947	5948.5830	0.0117	9	3	7	2	11	1	12	8	3	6	2	10	1	11
5948.5947	5948.6251	-0.0304	9	3	7	2	11	2	11	8	3	6	2	10	2	10
5948.5947	5948.5815	0.0132	9	3	7	2	11	2	12	8	3	6	2	10	2	11
5949.1127	5949.1078	0.0049	9	2	8	0	9	0	9	8	2	7	0	8	0	8
5949.1127	5949.1084	0.0043	9	4	6	0	9	1	9	8	4	5	0	8	1	8
5949.1127	5949.1146	-0.0019	9	4	6	0	9	2	11	8	4	5	0	8	2	10
5949.1127	5949.1084	0.0043	9	4	6	1	9	0	9	8	4	5	1	8	0	8
5949.1127	5949.1087	0.0040	9	4	6	1	9	1	9	8	4	5	1	8	1	8
5949.1127	5949.1147	-0.0020	9	4	6	1	9	2	11	8	4	5	1	8	2	10
5949.1127	5949.1088	0.0039	9	4	6	2	7	2	9	8	4	5	2	6	2	8

5949.1127	5949.1139	-0.0012	9	4	6	2	11	0	11	8	4	5	2	10	0	10
5949.1127	5949.1144	-0.0017	9	4	6	2	11	1	11	8	4	5	2	10	1	10
5949.1127	5949.1201	-0.0074	9	4	6	2	11	2	13	8	4	5	2	10	2	12
5949.2057	5949.1905	0.0152	9	4	6	0	9	1	10	8	2	7	0	8	1	9
5949.2057	5949.1983	0.0074	9	4	6	1	8	2	10	8	4	5	1	7	2	9
5949.2057	5949.1995	0.0062	9	4	6	1	9	1	10	8	4	5	1	8	1	9
5949.2057	5949.1884	0.0173	9	4	6	1	10	0	10	8	4	5	1	9	0	9
5949.2057	5949.1873	0.0184	9	4	6	1	10	1	10	8	4	5	1	9	1	9
5949.2057	5949.1974	0.0083	9	4	6	1	10	2	12	8	4	5	1	9	2	11
5949.2057	5949.2218	-0.0161	9	4	6	2	10	2	12	8	4	5	2	9	2	11
5949.2057	5949.2002	0.0055	9	4	6	2	11	1	10	8	4	5	2	10	1	9
5949.2057	5949.2012	0.0045	9	4	6	2	11	1	12	8	4	5	2	10	1	11
5949.2057	5949.1992	0.0065	9	4	6	2	11	2	12	8	4	5	2	10	2	11
5949.3011	5949.2841	0.0170	9	4	6	0	9	0	9	8	4	5	0	8	0	8
5949.3011	5949.2840	0.0171	9	4	6	1	8	1	9	8	4	5	1	7	1	8
5949.3011	5949.2864	0.0147	9	4	6	1	9	2	9	8	4	5	1	8	2	8
5949.3011	5949.2785	0.0226	9	4	6	1	10	1	11	8	4	5	1	9	1	10
5949.3011	5949.2898	0.0113	9	4	6	1	10	2	11	8	4	5	1	9	2	10
5949.3011	5949.2820	0.0191	9	4	6	2	9	1	9	8	4	5	2	8	1	8
5949.3011	5949.2778	0.0233	9	4	6	2	9	2	11	8	4	5	2	8	2	10
5949.3011	5949.2891	0.0120	9	4	6	2	10	1	11	8	4	5	2	9	1	10
5949.3011	5949.3117	-0.0106	9	4	6	2	10	2	11	8	4	5	2	9	2	10
5949.3011	5949.2900	0.0111	9	4	6	2	11	2	11	8	4	5	2	10	2	10
5950.7678	5950.7691	-0.0013	9	4	5	0	9	1	9	8	4	4	0	8	1	8
5950.7678	5950.7759	-0.0081	9	4	5	0	9	2	11	8	4	4	0	8	2	10
5950.7678	5950.7691	-0.0013	9	4	5	1	9	0	9	8	4	4	1	8	0	8
5950.7678	5950.7695	-0.0017	9	4	5	1	9	1	9	8	4	4	1	8	1	8
5950.7678	5950.7760	-0.0082	9	4	5	1	9	2	11	8	4	4	1	8	2	10
5950.7678	5950.7696	-0.0018	9	4	5	2	7	2	9	8	4	4	2	6	2	8
5950.7678	5950.7751	-0.0073	9	4	5	2	11	0	11	8	4	4	2	10	0	10
5950.7678	5950.7757	-0.0079	9	4	5	2	11	1	11	8	4	4	2	10	1	10
5950.7678	5950.7819	-0.0141	9	4	5	2	11	2	13	8	4	4	2	10	2	12
5950.7678	5950.7684	-0.0006	9	6	3	0	9	0	9	8	6	2	0	8	0	8
5950.8646	5950.8569	0.0077	9	4	5	0	9	1	10	8	4	4	0	8	1	9
5950.8646	5950.8657	-0.0011	9	4	5	1	8	2	10	8	4	4	1	7	2	9
5950.8646	5950.8668	-0.0022	9	4	5	1	9	1	10	8	4	4	1	8	1	9
5950.8646	5950.8549	0.0097	9	4	5	1	10	0	10	8	4	4	1	9	0	9
5950.8646	5950.8536	0.0110	9	4	5	1	10	1	10	8	4	4	1	9	1	9
5950.8646	5950.8649	-0.0003	9	4	5	1	10	2	12	8	4	4	1	9	2	11
5950.8646	5950.8912	-0.0266	9	4	5	2	10	2	12	8	4	4	2	9	2	11
5950.8646	5950.8674	-0.0028	9	4	5	2	11	1	10	8	4	4	2	10	1	9
5950.8646	5950.8683	-0.0037	9	4	5	2	11	1	12	8	4	4	2	10	1	11
5950.8646	5950.8665	-0.0019	9	4	5	2	11	2	12	8	4	4	2	10	2	11
5950.9711	5950.9574	0.0137	9	4	5	1	8	1	9	8	4	4	1	7	1	8
5950.9711	5950.9594	0.0117	9	4	5	1	9	2	9	8	4	4	1	8	2	8
5950.9711	5950.9513	0.0198	9	4	5	1	10	1	11	8	4	4	1	9	1	10
5950.9711	5950.9636	0.0075	9	4	5	1	10	2	11	8	4	4	1	9	2	10
5950.9711	5950.9555	0.0156	9	4	5	2	9	1	9	8	4	4	2	8	1	8
5950.9711	5950.9508	0.0203	9	4	5	2	9	2	11	8	4	4	2	8	2	10
5950.9711	5950.9630	0.0081	9	4	5	2	10	1	11	8	4	4	2	9	1	10
5950.9711	5950.9875	-0.0164	9	4	5	2	10	2	11	8	4	4	2	9	2	10
5950.9711	5950.9637	0.0074	9	4	5	2	11	2	11	8	4	4	2	10	2	10
5950.9711	5950.9577	0.0134	9	8	1	0	9	0	9	8	8	0	0	8	0	8

5975.7317	5975.7316	0.0001	9	3	6	0	9	1	9	8	3	5	0	8	1	8
5975.7317	5975.7365	-0.0048	9	3	6	0	9	2	11	8	3	5	0	8	2	10
5975.7317	5975.7316	0.0001	9	3	6	1	9	0	9	8	3	5	1	8	0	8
5975.7317	5975.7313	0.0004	9	3	6	1	9	1	9	8	3	5	1	8	1	8
5975.7317	5975.7361	-0.0044	9	3	6	1	9	2	11	8	3	5	1	8	2	10
5975.7317	5975.7324	-0.0007	9	3	6	2	7	2	9	8	3	5	2	6	2	8
5975.7317	5975.7360	-0.0043	9	3	6	2	11	0	11	8	3	5	2	10	0	10
5975.7317	5975.7359	-0.0042	9	3	6	2	11	1	11	8	3	5	2	10	1	10
5975.7317	5975.7312	0.0005	9	3	6	2	11	2	9	8	3	5	2	10	2	8
5975.7317	5975.7407	-0.0090	9	3	6	2	11	2	13	8	3	5	2	10	2	12
5975.8369	5975.8297	0.0072	9	3	6	0	9	1	10	8	3	5	0	8	1	9
5975.8369	5975.8297	0.0072	9	3	6	1	9	1	10	8	3	5	1	8	1	9
5975.8369	5975.8385	-0.0016	9	3	6	1	10	0	10	8	3	5	1	9	0	9
5975.8369	5975.8386	-0.0017	9	3	6	1	10	1	10	8	3	5	1	9	1	9
5975.8369	5975.8383	-0.0014	9	3	6	1	10	2	12	8	3	5	1	9	2	11
5975.8369	5975.8565	-0.0196	9	3	6	2	10	2	12	8	3	5	2	9	2	11
5975.8369	5975.8388	-0.0019	9	3	6	2	11	1	10	8	3	5	2	10	1	9
5975.8369	5975.8390	-0.0021	9	3	6	2	11	1	12	8	3	5	2	10	1	11
5975.8369	5975.8324	0.0045	9	3	6	2	11	2	10	8	3	5	2	10	2	9
5975.8369	5975.8387	-0.0018	9	3	6	2	11	2	12	8	3	5	2	10	2	11
5975.9388	5975.9406	-0.0018	9	3	6	1	8	1	9	8	3	5	1	7	1	8
5975.9388	5975.9412	-0.0024	9	3	6	1	10	1	9	8	3	5	1	9	1	8
5975.9388	5975.9367	0.0021	9	3	6	1	10	1	11	8	3	5	1	9	1	10
5975.9388	5975.9417	-0.0029	9	3	6	1	10	2	9	8	3	5	1	9	2	8
5975.9388	5975.9460	-0.0072	9	3	6	1	10	2	11	8	3	5	1	9	2	10
5975.9388	5975.9456	-0.0068	9	3	6	2	9	2	11	8	3	5	2	8	2	10
5975.9388	5975.9407	-0.0019	9	3	6	2	10	1	9	8	3	5	2	9	1	8
5975.9388	5975.9457	-0.0069	9	3	6	2	10	1	11	8	3	5	2	9	1	10
5975.9388	5975.9646	-0.0258	9	3	6	2	10	2	11	8	3	5	2	9	2	10
5975.9388	5975.9368	0.0020	9	3	6	2	11	2	11	8	3	5	2	10	2	10
5976.0435	5976.0492	-0.0057	9	3	6	1	8	2	8	8	3	5	1	7	2	7
5976.0435	5976.0447	-0.0012	9	3	6	1	10	2	10	8	3	5	1	9	2	9
5976.0435	5976.0346	0.0089	9	3	6	2	8	2	8	8	3	5	2	7	2	7
5976.0435	5976.0483	-0.0048	9	3	6	2	9	1	8	8	3	5	2	8	1	7
5976.0435	5976.0436	-0.0001	9	3	6	2	9	1	10	8	3	5	2	8	1	9
5976.0435	5976.0488	-0.0053	9	3	6	2	9	2	8	8	3	5	2	8	2	7
5976.0435	5976.0636	-0.0201	9	3	6	2	9	2	10	8	5	3	2	8	2	9
5976.0435	5976.0442	-0.0007	9	5	4	2	10	0	10	8	3	5	2	9	2	9
5988.2391	5988.2741	-0.0350	9	1	8	0	9	2	9	8	1	7	0	8	2	8
5988.2391	5988.2754	-0.0363	9	1	8	1	8	2	9	8	1	7	1	7	2	8
5988.2391	5988.2515	-0.0124	9	1	8	1	10	2	10	8	1	7	1	9	2	9
5988.2391	5988.2265	0.0126	9	1	8	2	9	1	8	8	1	7	2	8	1	7
5988.2391	5988.2450	-0.0059	9	1	8	2	9	1	10	8	1	7	2	8	1	9
5988.2391	5988.2297	0.0094	9	1	8	2	9	2	8	8	1	7	2	8	2	7
5988.2391	5988.2720	-0.0329	9	1	8	2	10	1	9	8	1	7	2	9	1	8
5988.2391	5988.2481	-0.0090	9	1	8	2	10	2	10	8	1	7	2	9	2	9
5988.2391	5988.2480	-0.0089	9	1	8	2	10	2	11	8	1	7	2	9	2	10
5988.2391	5988.2325	0.0066	9	3	6	1	8	0	8	8	1	7	1	7	2	7
5988.3076	5988.3538	-0.0462	9	1	8	0	9	2	10	8	1	7	0	8	2	9
5988.3076	5988.3261	-0.0185	9	1	8	1	9	2	9	8	1	7	1	8	2	8
5988.3076	5988.3541	-0.0465	9	1	8	1	9	2	10	8	1	7	1	8	2	9
5988.3076	5988.3411	-0.0335	9	1	8	1	10	1	11	8	1	7	1	9	1	10
5988.3076	5988.3096	-0.0020	9	1	8	1	10	2	11	8	1	7	1	9	2	10

5988.3076	5988.3401	-0.0325	9	1	8	2	9	2	11	8	1	7	2	8	2	10
5988.3076	5988.3534	-0.0458	9	1	8	2	10	1	10	8	1	7	2	9	1	9
5988.3076	5988.3087	-0.0011	9	1	8	2	10	1	11	8	1	7	2	9	1	10
5988.3076	5988.3365	-0.0289	9	1	8	2	10	2	12	8	1	7	2	9	2	11
5988.3076	5988.3099	-0.0023	9	1	8	2	11	2	11	8	1	7	2	10	2	10
5988.3888	5988.4389	-0.0501	9	1	8	0	9	1	10	8	1	7	0	8	1	9
5988.3888	5988.4039	-0.0151	9	1	8	1	8	2	10	8	1	7	1	7	2	9
5988.3888	5988.4057	-0.0169	9	1	8	1	9	1	10	8	1	7	1	9	1	9
5988.3888	5988.4357	-0.0469	9	1	8	1	10	0	10	8	1	7	1	9	0	9
5988.3888	5988.4396	-0.0508	9	1	8	1	10	1	10	8	1	7	1	8	1	9
5988.3888	5988.4026	-0.0138	9	1	8	1	10	2	12	8	1	7	1	9	2	11
5988.3888	5988.4067	-0.0179	9	1	8	2	11	1	10	8	1	7	2	10	1	9
5988.3888	5988.4082	-0.0194	9	1	8	2	11	1	12	8	1	7	2	10	1	11
5988.3888	5988.4376	-0.0488	9	1	8	2	11	2	10	8	1	7	2	10	2	9
5988.3888	5988.4052	-0.0164	9	1	8	2	11	2	12	8	1	7	2	10	2	11
5988.4627	5988.5042	-0.0415	9	1	8	0	9	1	9	8	1	7	0	8	1	8
5988.4627	5988.4843	-0.0216	9	1	8	0	9	2	11	8	1	7	0	8	2	10
5988.4627	5988.5041	-0.0414	9	1	8	1	9	0	9	8	1	7	1	8	0	8
5988.4627	5988.5007	-0.0380	9	1	8	1	9	1	9	8	1	7	1	8	1	8
5988.4627	5988.4845	-0.0218	9	1	8	1	9	2	11	8	1	7	1	8	2	10
5988.4627	5988.5112	-0.0485	9	1	8	2	7	2	9	8	1	7	0	8	0	8
5988.4627	5988.5009	-0.0382	9	1	8	2	8	2	9	8	1	7	2	6	2	8
5988.4627	5988.4904	-0.0277	9	1	8	2	11	0	11	8	1	7	2	10	0	10
5988.4627	5988.4841	-0.0214	9	1	8	2	11	1	11	8	1	7	2	10	1	10
5988.4627	5988.4696	-0.0069	9	1	8	2	11	2	13	8	1	7	2	10	2	12
6035.0200	6035.0826	-0.0626	9	2	7	1	10	1	11	8	2	6	1	9	1	10
6035.0200	6035.0736	-0.0536	9	2	7	1	10	2	11	8	2	6	1	9	2	10
6035.0200	6035.0248	-0.0048	9	2	7	1	10	2	12	8	2	6	1	9	2	11
6035.0200	6035.0826	-0.0626	9	2	7	2	9	2	11	8	2	6	2	8	2	10
6035.0200	6035.0737	-0.0537	9	2	7	2	10	1	11	8	2	6	2	9	1	10
6035.0200	6035.0578	-0.0378	9	2	7	2	10	2	11	8	2	6	2	9	2	10
6035.0200	6035.0034	0.0166	9	2	7	2	10	2	12	8	2	6	2	9	2	11
6035.0200	6035.0239	-0.0039	9	2	7	2	11	1	12	8	2	6	2	10	1	11
6035.0200	6035.0737	-0.0537	9	2	7	2	11	2	11	8	2	6	2	10	2	10
6035.0200	6035.0244	-0.0044	9	2	7	2	11	2	12	8	2	6	2	10	2	11
6420.0872	6420.1226	-0.0354	10	1	10	1	10	2	12	9	1	9	1	9	2	11
6420.0872	6420.0674	0.0198	10	1	10	1	11	1	12	9	1	9	1	10	1	11
6420.0872	6420.0936	-0.0064	10	1	10	1	11	2	13	9	1	9	1	10	2	12
6420.0872	6420.0952	-0.0080	10	1	10	2	11	2	13	9	1	9	2	10	2	12
6420.0872	6420.1230	-0.0358	10	1	10	2	12	0	12	9	1	9	2	11	0	11
6420.0872	6420.1231	-0.0359	10	1	10	2	12	1	12	9	1	9	2	11	1	11
6420.0872	6420.0967	-0.0095	10	1	10	2	12	1	13	9	1	9	2	11	1	12
6420.0872	6420.0697	0.0175	10	1	10	2	12	2	12	9	1	9	2	11	2	11
6420.0872	6420.0959	-0.0087	10	1	10	2	12	2	13	9	1	9	2	11	2	12
6420.0872	6420.1228	-0.0356	10	1	10	2	12	2	14	9	1	9	2	11	2	13
6433.5852	6433.5641	0.0211	10	0	10	0	10	2	11	9	0	9	0	9	2	10
6433.5852	6433.5760	0.0092	10	0	10	1	9	0	9	9	0	9	1	8	2	8
6433.5852	6433.6275	-0.0423	10	0	10	1	9	2	10	9	0	9	1	8	2	9
6433.5852	6433.5794	0.0058	10	0	10	1	11	2	11	9	0	9	1	10	2	10
6433.5852	6433.6270	-0.0418	10	0	10	2	10	0	10	9	0	9	2	9	0	9
6433.5852	6433.5715	0.0137	10	0	10	2	10	1	9	9	0	9	2	9	1	8
6433.5852	6433.6263	-0.0411	10	0	10	2	10	1	10	9	0	9	2	9	1	9
6433.5852	6433.5753	0.0099	10	0	10	2	10	1	11	9	0	9	2	9	1	10

6433.5852	6433.5773	0.0079	10	0	10	2	11	0	11	9	0	9	2	10	0	10
6433.6436	6433.6366	0.0070	10	0	10	1	9	1	10	9	0	9	1	8	1	9
6433.6436	6433.6361	0.0075	10	0	10	1	11	1	10	9	0	9	1	10	1	9
6433.6436	6433.6399	0.0037	10	0	10	1	11	1	12	9	0	9	1	10	1	11
6433.6436	6433.6397	0.0039	10	0	10	1	11	2	10	9	0	9	1	10	2	9
6433.6436	6433.6327	0.0109	10	0	10	1	11	2	12	9	0	9	1	10	2	11
6433.6436	6433.6207	0.0229	10	0	10	2	10	2	12	9	0	9	2	9	2	11
6433.6436	6433.6335	0.0101	10	0	10	2	11	1	12	9	0	9	2	10	1	11
6433.6436	6433.6310	0.0126	10	0	10	2	11	2	12	9	0	9	2	10	2	11
6433.6436	6433.6407	0.0029	10	0	10	2	12	2	12	9	0	9	2	11	2	11
6433.6991	6433.7026	-0.0035	10	0	10	0	10	1	11	9	0	9	0	9	1	10
6433.6991	6433.6871	0.0120	10	0	10	1	9	2	11	9	0	9	1	8	2	10
6433.6991	6433.7029	-0.0038	10	0	10	1	10	1	11	9	0	9	1	9	1	10
6433.6991	6433.6954	0.0037	10	0	10	1	11	0	11	9	0	9	1	10	0	10
6433.6991	6433.6957	0.0034	10	0	10	1	11	1	11	9	0	9	1	10	1	10
6433.6991	6433.6935	0.0056	10	0	10	1	11	2	13	9	0	9	1	10	2	12
6433.6991	6433.6825	0.0166	10	0	10	2	11	2	13	9	0	9	2	10	2	12
6433.6991	6433.6966	0.0025	10	0	10	2	12	1	11	9	0	9	2	11	1	10
6433.6991	6433.6982	0.0009	10	0	10	2	12	1	13	9	0	9	2	11	1	12
6433.6991	6433.6952	0.0039	10	0	10	2	12	2	13	9	0	9	2	11	2	12
6433.7562	6433.7588	-0.0026	10	0	10	0	10	0	10	9	0	9	0	9	0	9
6433.7562	6433.7587	-0.0025	10	0	10	0	10	1	10	9	0	9	0	9	1	9
6433.7562	6433.7551	0.0011	10	0	10	0	10	2	12	9	0	9	0	9	2	11
6433.7562	6433.7589	-0.0027	10	0	10	1	10	0	10	9	0	9	1	9	0	9
6433.7562	6433.7587	-0.0025	10	0	10	1	10	1	10	9	0	9	1	9	1	9
6433.7562	6433.7550	0.0012	10	0	10	1	10	2	12	9	0	9	1	9	2	11
6433.7562	6433.7555	0.0007	10	0	10	2	12	0	12	9	0	9	2	11	0	11
6433.7562	6433.7554	0.0008	10	0	10	2	12	1	12	9	0	9	2	11	1	11
6433.7562	6433.7592	-0.0030	10	0	10	2	12	2	10	9	0	9	2	11	2	9
6433.7562	6433.7519	0.0043	10	0	10	2	12	2	14	9	0	9	2	11	2	13
6553.4765	6553.4363	0.0402	10	2	9	0	10	2	12	9	2	8	0	9	2	11
6553.4765	6553.4361	0.0404	10	2	9	1	10	2	12	9	2	8	1	9	2	11
6553.4765	6553.3898	0.0867	10	2	9	1	11	1	12	9	2	8	1	10	1	11
6553.4765	6553.4089	0.0676	10	2	9	1	11	2	13	9	2	8	1	10	2	12
6553.4765	6553.3849	0.0916	10	2	9	2	10	2	12	9	2	8	2	9	2	11
6553.4765	6553.4091	0.0674	10	2	9	2	11	2	13	9	2	8	2	10	2	12
6553.4765	6553.4362	0.0403	10	2	9	2	12	1	12	9	2	8	2	11	1	11
6553.4765	6553.4126	0.0639	10	2	9	2	12	1	13	9	2	8	2	11	1	12
6553.4765	6553.3925	0.0840	10	2	9	2	12	2	13	9	2	8	2	11	2	12
6553.4765	6553.4318	0.0447	10	2	9	2	12	2	14	9	2	8	2	11	2	13
6603.8600	6603.8545	0.0055	10	6	4	0	10	1	10	9	6	3	0	9	1	9
6603.8600	6603.8554	0.0046	10	6	4	1	10	1	10	9	6	3	1	9	1	9
6603.8600	6603.8673	-0.0073	10	6	4	1	10	2	12	9	6	3	1	9	2	11
6603.8600	6603.8665	-0.0065	10	6	4	2	12	1	12	9	6	3	2	11	1	11
6603.8600	6603.8775	-0.0175	10	6	4	2	12	2	14	9	6	3	2	11	2	13
6603.8600	6603.8517	0.0083	10	6	5	0	10	1	10	9	6	4	0	9	1	9
6603.8600	6603.8529	0.0071	10	6	5	1	10	1	10	9	6	4	1	9	1	9
6603.8600	6603.8648	-0.0048	10	6	5	1	10	2	12	9	6	4	1	9	2	11
6603.8600	6603.8660	-0.0060	10	6	5	2	12	1	12	9	6	4	2	11	1	11
6603.8600	6603.8751	-0.0151	10	6	5	2	12	2	14	9	6	4	2	11	2	13
6603.9922	6603.9786	0.0136	10	6	4	1	10	1	11	9	6	3	1	9	1	10
6603.9922	6603.9756	0.0166	10	6	4	1	11	0	11	9	6	3	1	10	0	10
6603.9922	6603.9790	0.0132	10	6	4	1	11	1	11	9	6	3	1	10	1	10

6603.9922	6603.9852	0.0070	10	6	4	1	11	2	13	9	6	3	1	10	2	12
6603.9922	6603.9899	0.0023	10	6	4	2	12	1	13	9	6	3	2	11	1	12
6603.9922	6603.9772	0.0150	10	6	5	1	10	1	11	9	6	4	1	9	1	10
6603.9922	6603.9746	0.0176	10	6	5	1	11	0	11	9	6	4	1	10	0	10
6603.9922	6603.9759	0.0163	10	6	5	1	11	1	11	9	6	4	1	10	1	10
6603.9922	6603.9847	0.0075	10	6	5	1	11	2	13	9	6	4	1	10	2	12
6603.9922	6603.9896	0.0026	10	6	5	2	12	1	13	9	6	4	2	11	1	12
6604.1324	6604.1111	0.0213	10	6	4	1	9	1	10	9	6	3	1	8	1	9
6604.1324	6604.1106	0.0218	10	6	4	1	11	1	10	9	6	3	1	10	1	9
6604.1324	6604.0998	0.0326	10	6	4	1	11	1	12	9	6	3	1	10	1	11
6604.1324	6604.1201	0.0123	10	6	4	1	11	2	12	9	6	3	1	10	2	11
6604.1324	6604.1208	0.0116	10	6	4	2	11	1	12	9	6	3	2	10	1	11
6604.1324	6604.1093	0.0231	10	6	5	1	9	1	10	9	6	4	1	8	1	9
6604.1324	6604.1107	0.0217	10	6	5	1	11	1	10	9	6	4	1	10	1	9
6604.1324	6604.1002	0.0322	10	6	5	1	11	1	12	9	6	4	1	10	1	11
6604.1324	6604.1204	0.0120	10	6	5	1	11	2	12	9	6	4	1	10	2	11
6604.1324	6604.1190	0.0134	10	6	5	2	11	1	12	9	6	4	2	10	1	11
6604.2400	6604.2463	-0.0063	10	6	4	1	9	2	9	9	6	3	1	8	2	8
6604.2400	6604.2343	0.0057	10	6	4	1	11	2	11	9	6	3	1	10	2	10
6604.2400	6604.2408	-0.0008	10	6	4	2	10	1	9	9	4	5	0	9	1	8
6604.2400	6604.2279	0.0121	10	6	4	2	10	1	11	9	6	3	2	9	1	10
6604.2400	6604.2457	-0.0057	10	6	5	1	9	2	9	9	6	4	1	8	2	8
6604.2400	6604.2337	0.0063	10	6	5	1	11	2	11	9	6	4	1	10	2	10
6604.2400	6604.2403	-0.0003	10	6	5	2	10	1	9	9	4	6	0	9	1	8
6604.2400	6604.2271	0.0129	10	6	5	2	10	1	11	9	6	4	2	9	1	10
6604.2400	6604.2296	0.0104	10	10	0	2	11	0	11	9	6	3	2	9	2	10
6604.2400	6604.2301	0.0099	10	10	1	2	11	0	11	9	4	6	0	9	2	10
6607.4766	6607.4867	-0.0101	10	5	6	0	10	1	10	9	5	5	0	9	1	9
6607.4766	6607.4931	-0.0165	10	5	6	0	10	2	12	9	5	5	0	9	2	11
6607.4766	6607.4867	-0.0101	10	5	6	1	10	0	10	9	5	5	1	9	0	9
6607.4766	6607.4873	-0.0107	10	5	6	1	10	1	10	9	5	5	1	9	1	9
6607.4766	6607.4940	-0.0174	10	5	6	1	10	2	12	9	5	5	1	9	2	11
6607.4766	6607.4856	-0.0090	10	5	6	2	8	2	10	9	5	5	2	7	2	9
6607.4766	6607.4942	-0.0176	10	5	6	2	12	0	12	9	5	5	2	11	0	11
6607.4766	6607.4944	-0.0178	10	5	6	2	12	1	12	9	5	5	2	11	1	11
6607.4766	6607.4874	-0.0108	10	5	6	2	12	2	10	9	5	5	2	11	2	9
6607.4766	6607.5007	-0.0241	10	5	6	2	12	2	14	9	5	5	2	11	2	13
6607.6038	6607.6201	-0.0163	10	5	5	0	10	2	12	9	5	4	0	9	2	11
6607.6038	6607.6210	-0.0172	10	5	5	1	10	2	12	9	5	4	1	9	2	11
6607.6038	6607.6212	-0.0174	10	5	5	2	12	0	12	9	5	4	2	11	0	11
6607.6038	6607.6214	-0.0176	10	5	5	2	12	1	12	9	5	4	2	11	1	11
6607.6038	6607.6277	-0.0239	10	5	5	2	12	2	14	9	5	4	2	11	2	13
6607.6038	6607.5878	0.0160	10	5	6	1	11	2	13	9	5	5	1	10	2	12
6607.6038	6607.6096	-0.0058	10	5	6	2	11	2	13	9	5	5	2	10	2	12
6607.6038	6607.5844	0.0194	10	5	6	2	12	1	11	9	5	5	2	11	1	10
6607.6038	6607.5833	0.0205	10	5	6	2	12	1	13	9	5	5	2	11	1	12
6607.6038	6607.5854	0.0184	10	5	6	2	12	2	13	9	5	5	2	11	2	12
6607.7027	6607.7153	-0.0126	10	5	5	1	11	2	13	9	5	4	1	10	2	12
6607.7027	6607.7412	-0.0385	10	5	5	2	11	2	13	9	5	4	2	10	2	12
6607.7027	6607.7138	-0.0111	10	5	5	2	12	1	13	9	5	4	2	11	1	12
6607.7027	6607.7138	-0.0111	10	5	5	2	12	2	13	9	5	4	2	11	2	12
6607.7027	6607.6704	0.0323	10	5	6	1	11	1	12	9	5	5	1	10	1	11
6607.7027	6607.6834	0.0193	10	5	6	1	11	2	12	9	5	5	1	10	2	11

6607.7027	6607.6842	0.0185	10	5	6	2	10	2	12	9	5	5	2	9	2	11
6607.7027	6607.6843	0.0184	10	5	6	2	11	1	12	9	5	5	2	10	1	11
6607.7027	6607.7024	0.0003	10	5	6	2	11	2	12	9	5	5	2	10	2	11
6607.7027	6607.6697	0.0330	10	5	6	2	12	2	12	9	5	5	2	11	2	11
6607.7761	6607.7680	0.0081	10	3	8	2	11	2	11	9	9	1	2	10	0	10
6607.7761	6607.7983	-0.0222	10	5	5	1	11	1	12	9	5	4	1	10	1	11
6607.7761	6607.8194	-0.0433	10	5	5	1	11	2	12	9	5	4	1	10	2	11
6607.7761	6607.8123	-0.0362	10	5	5	2	10	2	12	9	5	4	2	9	2	11
6607.7761	6607.8127	-0.0366	10	5	5	2	11	1	12	9	5	4	2	10	1	11
6607.7761	6607.8389	-0.0628	10	5	5	2	11	2	12	9	5	4	2	10	2	11
6607.7761	6607.7977	-0.0216	10	5	5	2	12	2	12	9	5	4	2	11	2	11
6607.7761	6607.7655	0.0106	10	5	6	1	11	2	11	9	5	5	1	10	2	10
6607.7761	6607.7708	0.0053	10	5	6	2	10	1	11	9	5	5	2	9	1	10
6607.7761	6607.7820	-0.0059	10	5	6	2	10	2	11	9	5	5	2	9	2	10
6612.9250	6612.9156	0.0094	10	2	9	0	10	0	10	9	2	8	0	9	0	9
6612.9250	6612.9152	0.0098	10	4	7	0	10	1	10	9	4	6	0	9	1	9
6612.9250	6612.9181	0.0069	10	4	7	0	10	2	12	9	4	6	0	9	2	11
6612.9250	6612.9152	0.0098	10	4	7	1	10	0	10	9	4	6	1	9	0	9
6612.9250	6612.9151	0.0099	10	4	7	1	10	1	10	9	4	6	1	9	1	9
6612.9250	6612.9181	0.0069	10	4	7	1	10	2	12	9	4	6	1	9	2	11
6612.9250	6612.9152	0.0098	10	4	7	2	8	2	10	9	4	6	2	7	2	9
6612.9250	6612.9183	0.0067	10	4	7	2	12	0	12	9	4	6	2	11	0	11
6612.9250	6612.9180	0.0070	10	4	7	2	12	1	12	9	4	6	2	11	1	11
6612.9250	6612.9209	0.0041	10	4	7	2	12	2	14	9	4	6	2	11	2	13
6612.9940	6612.9835	0.0105	10	4	7	1	9	2	11	9	4	6	1	8	2	10
6612.9940	6612.9843	0.0097	10	4	7	1	10	1	11	9	4	6	1	9	1	10
6612.9940	6612.9792	0.0148	10	4	7	1	11	0	11	9	4	6	1	10	0	10
6612.9940	6612.9783	0.0157	10	4	7	1	11	1	11	9	4	6	1	10	1	10
6612.9940	6612.9828	0.0112	10	4	7	1	11	2	13	9	4	6	1	10	2	12
6612.9940	6612.9970	-0.0030	10	4	7	2	11	2	13	9	4	6	2	10	2	12
6612.9940	6612.9848	0.0092	10	4	7	2	12	1	11	9	4	6	2	11	1	10
6612.9940	6612.9855	0.0085	10	4	7	2	12	1	13	9	4	6	2	11	1	12
6612.9940	6612.9801	0.0139	10	4	7	2	12	2	11	9	4	6	2	11	2	10
6612.9940	6612.9840	0.0100	10	4	7	2	12	2	13	9	4	6	2	11	2	12
6613.0500	6613.0505	-0.0005	10	4	7	1	9	1	10	9	4	6	1	8	1	9
6613.0500	6613.0519	-0.0019	10	4	7	1	10	2	10	9	4	6	1	9	2	9
6613.0500	6613.0505	-0.0005	10	4	7	1	11	1	10	9	4	6	1	10	1	9
6613.0500	6613.0474	0.0026	10	4	7	1	11	1	12	9	4	6	1	10	1	11
6613.0500	6613.0538	-0.0038	10	4	7	1	11	2	12	9	4	6	1	10	2	11
6613.0500	6613.0491	0.0009	10	4	7	2	10	1	10	9	4	6	2	9	1	9
6613.0500	6613.0468	0.0032	10	4	7	2	10	2	12	9	4	6	2	9	2	11
6613.0500	6613.0535	-0.0035	10	4	7	2	11	1	12	9	4	6	2	10	1	11
6613.0500	6613.0666	-0.0166	10	4	7	2	11	2	12	9	4	6	2	10	2	11
6613.0500	6613.0541	-0.0041	10	4	7	2	12	2	12	9	4	6	2	11	2	11
6616.4593	6616.4585	0.0008	10	4	6	0	10	1	10	9	4	5	0	9	1	9
6616.4593	6616.4624	-0.0031	10	4	6	0	10	2	12	9	4	5	0	9	2	11
6616.4593	6616.4585	0.0008	10	4	6	1	10	0	10	9	4	5	1	9	0	9
6616.4593	6616.4585	0.0008	10	4	6	1	10	1	10	9	4	5	1	9	1	9
6616.4593	6616.4622	-0.0029	10	4	6	1	10	2	12	9	4	5	1	9	2	11
6616.4593	6616.4587	0.0006	10	4	6	2	8	2	10	9	4	5	2	7	2	9
6616.4593	6616.4622	-0.0029	10	4	6	2	12	0	12	9	4	5	2	11	0	11
6616.4593	6616.4623	-0.0030	10	4	6	2	12	1	12	9	4	5	2	11	1	11
6616.4593	6616.4585	0.0008	10	4	6	2	12	2	10	9	4	5	2	11	2	9

6616.4593	6616.4659	-0.0066	10	4	6	2	12	2	14	9	4	5	2	11	2	13
6616.5451	6616.5331	0.0120	10	2	8	2	10	2	11	9	2	7	2	9	2	10
6616.5451	6616.5312	0.0139	10	4	6	0	10	1	11	9	4	5	0	9	1	10
6616.5451	6616.5311	0.0140	10	4	6	1	10	1	11	9	4	5	1	9	1	10
6616.5451	6616.5391	0.0060	10	4	6	1	11	0	11	9	4	5	1	10	0	10
6616.5451	6616.5387	0.0064	10	4	6	1	11	1	11	9	4	5	1	10	1	10
6616.5451	6616.5396	0.0055	10	4	6	1	11	2	13	9	4	5	1	10	2	12
6616.5451	6616.5547	-0.0096	10	4	6	2	11	2	13	9	4	5	2	10	2	12
6616.5451	6616.5380	0.0071	10	4	6	2	12	1	11	9	4	5	2	11	1	10
6616.5451	6616.5376	0.0075	10	4	6	2	12	1	13	9	4	5	2	11	1	12
6616.5451	6616.5385	0.0066	10	4	6	2	12	2	13	9	4	5	2	11	2	12
6616.6549	6616.6153	0.0396	10	4	6	1	9	1	10	9	4	5	1	8	1	9
6616.6549	6616.6153	0.0396	10	4	6	1	11	1	10	9	4	5	1	10	1	9
6616.6549	6616.6113	0.0436	10	4	6	1	11	1	12	9	4	5	1	10	1	11
6616.6549	6616.6142	0.0407	10	4	6	1	11	2	10	9	4	5	1	10	2	9
6616.6549	6616.6191	0.0358	10	4	6	1	11	2	12	9	4	5	1	10	2	11
6616.6549	6616.6193	0.0356	10	4	6	2	10	2	12	9	4	5	2	9	2	11
6616.6549	6616.6164	0.0385	10	4	6	2	11	1	10	9	4	5	2	10	1	9
6616.6549	6616.6195	0.0354	10	4	6	2	11	1	12	9	4	5	2	10	1	11
6616.6549	6616.6352	0.0197	10	4	6	2	11	2	12	9	4	5	2	10	2	11
6616.6549	6616.6110	0.0439	10	4	6	2	12	2	12	9	4	5	2	11	2	11
6616.7478	6616.6960	0.0518	10	2	8	2	10	2	9	9	2	7	2	9	2	8
6616.7478	6616.6918	0.0560	10	2	8	2	11	2	11	9	2	7	2	10	2	10
6616.7478	6616.6949	0.0529	10	4	6	1	9	2	9	9	4	5	1	8	2	8
6616.7478	6616.6908	0.0570	10	4	6	1	11	2	11	9	4	5	1	10	2	10
6616.7478	6616.6821	0.0657	10	4	6	2	9	2	9	9	4	5	2	8	2	8
6616.7478	6616.6970	0.0508	10	4	6	2	10	1	9	9	6	3	2	9	1	8
6616.7478	6616.6930	0.0548	10	4	6	2	10	1	11	9	4	5	2	9	1	10
6616.7478	6616.7077	0.0401	10	4	6	2	10	2	11	9	4	5	2	9	2	10
6633.5994	6633.6512	-0.0518	10	1	9	0	10	2	10	9	1	8	0	9	2	9
6633.5994	6633.5905	0.0089	10	1	9	1	9	2	9	9	3	6	1	8	0	8
6633.5994	6633.6074	-0.0080	10	1	9	1	11	2	11	9	1	8	1	10	2	10
6633.5994	6633.5838	0.0156	10	1	9	2	10	1	9	9	1	8	2	9	1	8
6633.5994	6633.6002	-0.0008	10	1	9	2	10	1	11	9	1	8	2	9	1	10
6633.5994	6633.5876	0.0118	10	1	9	2	10	2	9	9	1	8	2	9	2	8
6633.5994	6633.6485	-0.0491	10	1	9	2	11	1	10	9	1	8	2	10	1	9
6633.5994	6633.6034	-0.0040	10	1	9	2	11	2	11	9	1	8	2	10	2	10
6633.5994	6633.6259	-0.0265	10	1	9	2	11	2	12	9	1	8	2	10	2	11
6633.5994	6633.5530	0.0464	10	3	7	2	10	2	11	9	1	8	2	9	2	10
6633.6862	6633.7458	-0.0596	10	1	9	0	10	2	11	9	1	8	0	9	2	10
6633.6862	6633.6992	-0.0130	10	1	9	1	10	2	10	9	1	8	1	9	2	9
6633.6862	6633.7461	-0.0599	10	1	9	1	10	2	11	9	1	8	1	9	2	10
6633.6862	6633.7103	-0.0241	10	1	9	1	11	1	12	9	1	8	1	10	1	11
6633.6862	6633.6819	0.0043	10	1	9	1	11	2	12	9	1	8	1	10	2	11
6633.6862	6633.7082	-0.0220	10	1	9	2	10	2	12	9	1	8	2	9	2	11
6633.6862	6633.7453	-0.0591	10	1	9	2	11	1	11	9	1	8	2	10	1	10
6633.6862	6633.6810	0.0052	10	1	9	2	11	1	12	9	1	8	2	10	1	11
6633.6862	6633.7291	-0.0429	10	1	9	2	11	2	13	9	1	8	2	10	2	12
6633.6862	6633.6830	0.0032	10	1	9	2	12	2	12	9	1	8	2	11	2	11
6633.7744	6633.8195	-0.0451	10	1	9	0	10	1	11	9	1	8	0	9	1	10
6633.7744	6633.7886	-0.0142	10	1	9	1	9	2	11	9	1	8	1	8	2	10
6633.7744	6633.7911	-0.0167	10	1	9	1	10	1	11	9	1	8	1	9	1	10
6633.7744	6633.8160	-0.0416	10	1	9	1	11	0	11	9	1	8	1	10	0	10

6633.7744	6633.8204	-0.0460	10	1	9	1	11	1	11	9	1	8	1	10	1	10
6633.7744	6633.7872	-0.0128	10	1	9	1	11	2	13	9	1	8	1	10	2	12
6633.7744	6633.7919	-0.0175	10	1	9	2	12	1	11	9	1	8	2	11	1	10
6633.7744	6633.7935	-0.0191	10	1	9	2	12	1	13	9	1	8	2	11	1	12
6633.7744	6633.8184	-0.0440	10	1	9	2	12	2	11	9	1	8	2	11	2	10
6633.7744	6633.7899	-0.0155	10	1	9	2	12	2	13	9	1	8	2	11	2	12
6633.9051	6633.9013	0.0038	10	1	9	0	10	1	10	9	1	8	0	9	1	9
6633.9051	6633.8840	0.0211	10	1	9	0	10	2	12	9	1	8	0	9	2	11
6633.9051	6633.9012	0.0039	10	1	9	1	10	0	10	9	1	8	1	9	0	9
6633.9051	6633.8987	0.0064	10	1	9	1	10	1	10	9	1	8	1	9	1	9
6633.9051	6633.8841	0.0210	10	1	9	1	10	2	12	9	1	8	1	9	2	11
6633.9051	6633.9063	-0.0012	10	1	9	2	8	2	10	9	1	8	2	7	2	9
6633.9051	6633.8991	0.0060	10	1	9	2	9	2	10	9	1	8	2	8	2	9
6633.9051	6633.8884	0.0167	10	1	9	2	12	0	12	9	1	8	2	11	0	11
6633.9051	6633.8837	0.0214	10	1	9	2	12	1	12	9	1	8	2	11	1	11
6633.9051	6633.8704	0.0347	10	1	9	2	12	2	14	9	1	8	2	11	2	13
6653.1198	6653.1240	-0.0042	10	3	7	0	10	1	10	9	3	6	0	9	1	9
6653.1198	6653.1278	-0.0080	10	3	7	0	10	2	12	9	3	6	0	9	2	11
6653.1198	6653.1239	-0.0041	10	3	7	1	10	0	10	9	3	6	1	9	0	9
6653.1198	6653.1235	-0.0037	10	3	7	1	10	1	10	9	3	6	1	9	1	9
6653.1198	6653.1273	-0.0075	10	3	7	1	10	2	12	9	3	6	1	9	2	11
6653.1198	6653.1248	-0.0050	10	3	7	2	8	2	10	9	3	6	2	7	2	9
6653.1198	6653.1272	-0.0074	10	3	7	2	12	0	12	9	3	6	2	11	0	11
6653.1198	6653.1270	-0.0072	10	3	7	2	12	1	12	9	3	6	2	11	1	11
6653.1198	6653.1235	-0.0037	10	3	7	2	12	2	10	9	3	6	2	11	2	9
6653.1198	6653.1308	-0.0110	10	3	7	2	12	2	14	9	3	6	2	11	2	13
6653.2189	6653.2267	-0.0078	10	1	9	1	11	0	11	9	3	6	1	10	0	10
6653.2189	6653.2204	-0.0015	10	3	7	0	10	1	11	9	3	6	0	9	1	10
6653.2189	6653.2204	-0.0015	10	3	7	1	10	1	11	9	3	6	1	9	1	10
6653.2189	6653.2271	-0.0082	10	3	7	1	11	1	11	9	3	6	1	10	1	10
6653.2189	6653.2262	-0.0073	10	3	7	1	11	2	13	9	3	6	1	10	2	12
6653.2189	6653.2408	-0.0219	10	3	7	2	11	2	13	9	3	6	2	10	2	12
6653.2189	6653.2277	-0.0088	10	3	7	2	12	1	11	9	3	6	2	11	1	10
6653.2189	6653.2282	-0.0093	10	3	7	2	12	1	13	9	3	6	2	11	1	12
6653.2189	6653.2230	-0.0041	10	3	7	2	12	2	11	9	3	6	2	11	2	10
6653.2189	6653.2272	-0.0083	10	3	7	2	12	2	13	9	3	6	2	11	2	12
6653.3209	6653.3258	-0.0049	10	3	7	1	9	1	10	9	3	6	1	8	1	9
6653.3209	6653.3270	-0.0061	10	3	7	1	11	1	10	9	3	6	1	10	1	9
6653.3209	6653.3236	-0.0027	10	3	7	1	11	1	12	9	3	6	1	10	1	11
6653.3209	6653.2760	0.0449	10	3	7	1	11	2	10	9	3	6	1	10	2	9
6653.3209	6653.3309	-0.0100	10	3	7	1	11	2	12	9	3	6	1	10	2	11
6653.3209	6653.3452	-0.0243	10	3	7	2	10	2	12	9	3	6	2	10	2	11
6653.3209	6653.3265	-0.0056	10	3	7	2	11	1	10	9	3	6	2	10	1	9
6653.3209	6653.3305	-0.0096	10	3	7	2	11	1	12	9	3	6	2	10	1	11
6653.3209	6653.3303	-0.0094	10	3	7	2	11	2	12	9	3	6	2	9	2	11
6653.3209	6653.3238	-0.0029	10	3	7	2	12	2	12	9	3	6	2	11	2	11
6653.4309	6653.4427	-0.0118	10	3	7	0	10	2	11	9	3	6	2	9	2	10
6653.4309	6653.4319	-0.0010	10	3	7	1	9	2	9	9	3	6	1	8	2	8
6653.4309	6653.4216	0.0093	10	3	7	2	9	0	9	9	3	6	2	8	2	8
6653.4309	6653.4261	0.0048	10	3	7	2	10	1	11	9	3	6	2	9	1	10
6653.4309	6653.4308	0.0001	10	5	5	0	10	2	9	9	3	6	2	9	2	8
6653.4309	6653.4286	0.0023	10	5	5	1	11	2	11	9	3	6	1	10	2	10
6653.4309	6653.4297	0.0012	10	5	5	2	10	1	9	9	3	6	2	9	1	8

6653.4309	6653.4274	0.0035	10	5	5	2	11	0	11	9	5	4	2	10	0	10
6709.6875	6709.6468	0.0407	10	2	8	1	10	2	12	9	2	7	1	9	2	11
6709.6875	6709.7058	-0.0183	10	2	8	1	11	1	12	9	2	7	1	10	1	11
6709.6875	6709.6736	0.0139	10	2	8	1	11	2	13	9	2	7	1	10	2	12
6709.6875	6709.7058	-0.0183	10	2	8	2	10	2	12	9	2	7	2	9	2	11
6709.6875	6709.6464	0.0411	10	2	8	2	11	2	13	9	2	7	2	10	2	12
6709.6875	6709.6468	0.0407	10	2	8	2	12	0	12	9	2	7	2	11	0	11
6709.6875	6709.6468	0.0407	10	2	8	2	12	1	12	9	2	7	2	11	1	11
6709.6875	6709.6737	0.0138	10	2	8	2	12	1	13	9	2	7	2	11	1	12
6709.6875	6709.6736	0.0139	10	2	8	2	12	2	13	9	2	7	2	11	2	12
6709.6875	6709.6414	0.0461	10	2	8	2	12	2	14	9	2	7	2	11	2	13
7055.7101	7055.7308	-0.0207	11	1	11	1	11	2	13	10	1	10	1	10	2	12
7055.7101	7055.6781	0.0320	11	1	11	1	12	1	13	10	1	10	1	11	1	12
7055.7101	7055.7032	0.0069	11	1	11	1	12	2	14	10	1	10	1	11	2	13
7055.7101	7055.7050	0.0051	11	1	11	2	12	2	14	10	1	10	2	11	2	13
7055.7101	7055.7311	-0.0210	11	1	11	2	13	0	13	10	1	10	2	12	0	12
7055.7101	7055.7313	-0.0212	11	1	11	2	13	1	13	10	1	10	2	12	1	12
7055.7101	7055.7060	0.0041	11	1	11	2	13	1	14	10	1	10	2	12	1	13
7055.7101	7055.6803	0.0298	11	1	11	2	13	2	13	10	1	10	2	12	2	12
7055.7101	7055.7054	0.0047	11	1	11	2	13	2	14	10	1	10	2	12	2	13
7055.7101	7055.7311	-0.0210	11	1	11	2	13	2	15	10	1	10	2	12	2	14
7065.0436	7065.1006	-0.0570	11	0	11	0	11	2	13	10	0	10	0	10	2	12
7065.0436	7065.1007	-0.0571	11	0	11	1	11	2	13	10	0	10	1	10	2	12
7065.0436	7065.0517	-0.0081	11	0	11	1	12	2	14	10	0	10	1	11	2	13
7065.0436	7065.0461	-0.0025	11	0	11	2	12	2	14	10	0	10	2	11	2	13
7065.0436	7065.1011	-0.0575	11	0	11	2	13	0	13	10	0	10	2	12	0	12
7065.0436	7065.1011	-0.0575	11	0	11	2	13	1	13	10	0	10	2	12	1	12
7065.0436	7065.0556	-0.0120	11	0	11	2	13	1	14	10	0	10	2	12	1	13
7065.0436	7065.0099	0.0337	11	0	11	2	13	2	13	10	0	10	2	12	2	12
7065.0436	7065.0538	-0.0102	11	0	11	2	13	2	14	10	0	10	2	12	2	13
7065.0436	7065.0990	-0.0554	11	0	11	2	13	2	15	10	0	10	2	12	2	14
7199.4563	7199.4649	-0.0086	11	2	10	0	11	2	13	10	2	9	0	10	2	12
7199.4563	7199.4647	-0.0084	11	2	10	1	11	2	13	10	2	9	1	10	2	12
7199.4563	7199.4066	0.0497	11	2	10	1	12	1	13	10	2	9	1	11	1	12
7199.4563	7199.4319	0.0244	11	2	10	1	12	2	14	10	2	9	1	11	2	13
7199.4563	7199.4011	0.0552	11	2	10	2	11	2	13	10	2	9	2	10	2	12
7199.4563	7199.4314	0.0249	11	2	10	2	12	2	14	10	2	9	2	11	2	13
7199.4563	7199.4648	-0.0085	11	2	10	2	13	1	13	10	2	9	2	12	1	12
7199.4563	7199.4355	0.0208	11	2	10	2	13	1	14	10	2	9	2	12	1	13
7199.4563	7199.4173	0.0390	11	2	10	2	13	2	14	10	2	9	2	12	2	13
7199.4563	7199.4607	-0.0044	11	2	10	2	13	2	15	10	2	9	2	12	2	14
7268.6243	7268.6174	0.0069	11	3	9	1	11	2	13	10	3	8	1	10	2	12
7268.6243	7268.6490	-0.0247	11	3	9	1	12	1	13	10	3	8	1	11	1	12
7268.6243	7268.6333	-0.0090	11	3	9	1	12	2	14	10	3	8	1	11	2	13
7268.6243	7268.6229	0.0014	11	3	9	2	12	2	14	10	3	8	2	11	2	13
7268.6243	7268.6174	0.0069	11	3	9	2	13	0	13	10	3	8	2	12	0	12
7268.6243	7268.6174	0.0069	11	3	9	2	13	1	13	10	3	8	2	12	1	12
7268.6243	7268.6312	-0.0069	11	3	9	2	13	1	14	10	3	8	2	12	1	13
7268.6243	7268.6480	-0.0237	11	3	9	2	13	2	13	10	3	8	2	12	2	12
7268.6243	7268.6320	-0.0077	11	3	9	2	13	2	14	10	3	8	2	12	2	13
7268.6243	7268.6155	0.0088	11	3	9	2	13	2	15	10	3	8	2	12	2	14
7270.8700	7270.8679	0.0021	11	5	7	0	11	1	11	10	5	6	0	10	1	10
7270.8700	7270.8715	-0.0015	11	5	7	0	11	2	13	10	5	6	0	10	2	12

7270.8700	7270.8679	0.0021	11	5	7	1	11	0	11	10	5	6	1	10	0	10
7270.8700	7270.8678	0.0022	11	5	7	1	11	1	11	10	5	6	1	10	1	10
7270.8700	7270.8716	-0.0016	11	5	7	1	11	2	13	10	5	6	1	10	2	12
7270.8700	7270.8679	0.0021	11	5	7	2	9	2	11	10	5	6	2	8	2	10
7270.8700	7270.8716	-0.0016	11	5	7	2	13	0	13	10	5	6	2	12	0	12
7270.8700	7270.8713	-0.0013	11	5	7	2	13	1	13	10	5	6	2	12	1	12
7270.8700	7270.8749	-0.0049	11	5	7	2	13	2	15	10	5	6	2	12	2	14
7270.8700	7270.8681	0.0019	11	7	5	0	11	0	11	10	7	4	0	10	0	10
7270.9396	7270.9397	-0.0001	11	5	7	0	11	1	12	10	5	6	0	10	1	11
7270.9396	7270.9430	-0.0034	11	5	7	1	10	2	12	10	5	6	1	9	2	11
7270.9396	7270.9441	-0.0045	11	5	7	1	11	1	12	10	5	6	1	10	1	11
7270.9396	7270.9376	0.0020	11	5	7	1	12	0	12	10	5	6	1	11	0	11
7270.9396	7270.9422	-0.0026	11	5	7	1	12	2	14	10	5	6	1	11	2	13
7270.9396	7270.9648	-0.0252	11	5	7	2	12	2	14	10	5	6	2	11	2	13
7270.9396	7270.9446	-0.0050	11	5	7	2	13	1	12	10	5	6	2	12	1	11
7270.9396	7270.9454	-0.0058	11	5	7	2	13	1	14	10	5	6	2	12	1	13
7270.9396	7270.9388	0.0008	11	5	7	2	13	2	12	10	5	6	2	12	2	11
7270.9396	7270.9437	-0.0041	11	5	7	2	13	2	14	10	5	6	2	12	2	13
7270.9929	7271.0178	-0.0249	11	5	7	1	10	1	11	10	5	6	1	9	1	10
7270.9929	7271.0195	-0.0266	11	5	7	1	11	2	11	10	5	6	1	10	2	10
7270.9929	7271.0183	-0.0254	11	5	7	1	12	1	11	10	5	6	1	11	1	10
7270.9929	7271.0128	-0.0199	11	5	7	1	12	1	13	10	5	6	1	11	1	12
7270.9929	7271.0228	-0.0299	11	5	7	1	12	2	13	10	5	6	1	11	2	12
7270.9929	7271.0161	-0.0232	11	5	7	2	11	1	11	10	5	6	2	10	1	10
7270.9929	7271.0122	-0.0193	11	5	7	2	11	2	13	10	5	6	2	10	2	12
7270.9929	7271.0225	-0.0296	11	5	7	2	12	1	13	10	5	6	2	11	1	12
7270.9929	7271.0422	-0.0493	11	5	7	2	12	2	13	10	5	6	2	11	2	12
7270.9929	7271.0231	-0.0302	11	5	7	2	13	2	13	10	5	6	2	12	2	12
7271.0875	7271.0962	-0.0087	11	3	9	2	11	2	10	10	3	8	2	10	2	9
7271.0875	7271.0909	-0.0034	11	3	9	2	12	2	12	10	3	8	2	11	2	11
7271.0875	7271.0928	-0.0053	11	5	7	1	12	2	12	10	5	6	1	11	2	11
7271.0875	7271.0943	-0.0068	11	5	7	2	11	1	10	10	5	6	2	10	1	9
7271.0875	7271.0892	-0.0017	11	5	7	2	11	1	12	10	5	6	2	10	1	11
7271.0875	7271.0978	-0.0103	11	7	5	1	10	0	10	10	3	8	1	9	2	9
7271.0875	7271.0814	0.0061	11	9	3	2	10	2	10	10	5	6	2	9	2	9
7271.0875	7271.1094	-0.0219	11	9	3	2	11	2	12	10	7	4	2	10	2	11
7271.1779	7271.1826	-0.0047	11	5	6	0	11	1	11	10	5	5	0	10	1	10
7271.1779	7271.1863	-0.0084	11	5	6	0	11	2	13	10	5	5	0	10	2	12
7271.1779	7271.1826	-0.0047	11	5	6	1	11	0	11	10	5	5	1	10	0	10
7271.1779	7271.1825	-0.0046	11	5	6	1	11	1	11	10	5	5	1	10	1	10
7271.1779	7271.1864	-0.0085	11	5	6	1	11	2	13	10	5	5	1	10	2	12
7271.1779	7271.1864	-0.0085	11	5	6	2	13	0	13	10	5	5	2	12	0	12
7271.1779	7271.1861	-0.0082	11	5	6	2	13	1	13	10	5	5	2	12	1	12
7271.1779	7271.1898	-0.0119	11	5	6	2	13	2	15	10	5	5	2	12	2	14
7271.1779	7271.1682	0.0097	11	5	7	2	11	2	11	10	5	6	2	10	2	10
7271.1779	7271.1828	-0.0049	11	7	4	0	11	0	11	10	7	3	0	10	0	10
7271.2612	7271.2553	0.0059	11	5	6	0	11	1	12	10	5	5	0	10	1	11
7271.2612	7271.2588	0.0024	11	5	6	1	10	2	12	10	5	5	1	9	2	11
7271.2612	7271.2599	0.0013	11	5	6	1	11	1	12	10	5	5	1	10	1	11
7271.2612	7271.2533	0.0079	11	5	6	1	12	0	12	10	5	5	1	11	0	11
7271.2612	7271.2581	0.0031	11	5	6	1	12	2	14	10	5	5	1	11	2	13
7271.2612	7271.2809	-0.0197	11	5	6	2	12	2	14	10	5	5	2	11	2	13
7271.2612	7271.2605	0.0007	11	5	6	2	13	1	12	10	5	5	2	12	1	11

7271.2612	7271.2613	-0.0001	11	5	6	2	13	1	14	10	5	5	2	12	1	13
7271.2612	7271.2545	0.0067	11	5	6	2	13	2	12	10	5	5	2	12	2	11
7271.2612	7271.2596	0.0016	11	5	6	2	13	2	14	10	5	5	2	12	2	13
7271.3422	7271.3346	0.0076	11	5	6	1	10	1	11	10	5	5	1	9	1	10
7271.3422	7271.3364	0.0058	11	5	6	1	11	2	11	10	5	5	1	10	2	10
7271.3422	7271.3352	0.0070	11	5	6	1	12	1	11	10	5	5	1	11	1	10
7271.3422	7271.3296	0.0126	11	5	6	1	12	1	13	10	5	5	1	11	1	12
7271.3422	7271.3397	0.0025	11	5	6	1	12	2	13	10	5	5	1	11	2	12
7271.3422	7271.3330	0.0092	11	5	6	2	11	1	11	10	5	5	2	10	1	10
7271.3422	7271.3290	0.0132	11	5	6	2	11	2	13	10	5	5	2	10	2	12
7271.3422	7271.3394	0.0028	11	5	6	2	12	1	13	10	5	5	2	11	1	12
7271.3422	7271.3400	0.0022	11	5	6	2	13	2	13	10	5	5	2	12	2	12
7271.4216	7271.4106	0.0110	11	5	6	1	12	2	12	10	5	5	1	11	2	11
7271.4216	7271.4123	0.0093	11	5	6	2	11	1	10	10	5	5	2	10	1	9
7271.4216	7271.4071	0.0145	11	5	6	2	11	1	12	10	5	5	2	10	1	11
7271.4216	7271.4142	0.0074	11	5	6	2	11	2	10	10	5	5	2	10	2	9
7271.4216	7271.4088	0.0128	11	5	6	2	12	2	12	10	5	5	2	11	2	11
7271.4216	7271.3594	0.0622	11	5	6	2	12	2	13	10	5	5	2	11	2	12
7271.4216	7271.4157	0.0059	11	7	4	1	10	0	10	10	3	7	1	9	2	9
7271.4216	7271.3996	0.0220	11	7	4	2	10	2	10	10	5	5	2	9	2	9
7271.4216	7271.4275	-0.0059	11	7	4	2	11	2	12	10	7	3	2	10	2	11
7284.0738	7284.1262	-0.0524	11	4	7	2	13	2	15	10	4	6	2	12	2	14
7284.0738	7284.1235	-0.0497	11	4	7	0	11	2	13	10	4	6	0	10	2	12
7284.0738	7284.1237	-0.0499	11	4	7	2	13	0	13	10	4	6	2	12	0	12
7284.0738	7284.1209	-0.0471	11	2	9	2	10	2	11	10	2	8	2	9	2	10
7284.0738	7284.1209	-0.0471	11	4	7	1	11	1	11	10	4	6	1	10	1	10
7284.0738	7284.1210	-0.0472	11	4	7	2	13	2	11	10	4	6	2	12	2	10
7284.0738	7284.1213	-0.0475	11	4	7	2	9	2	11	10	6	4	0	10	0	10
7284.0738	7284.1235	-0.0497	11	4	7	1	11	2	13	10	4	6	1	10	2	12
7284.0738	7284.1235	-0.0497	11	4	7	2	13	1	13	10	4	6	2	12	1	12
7284.0738	7284.1184	-0.0446	11	4	7	2	9	0	9	10	4	6	2	8	0	8
7284.1564	7284.1929	-0.0365	11	4	7	2	13	2	14	10	4	6	2	12	2	13
7284.1564	7284.2032	-0.0468	11	4	7	2	12	2	14	10	4	6	2	11	2	13
7284.1564	7284.1930	-0.0366	11	4	7	1	11	1	12	10	4	6	1	10	1	11
7284.1564	7284.1891	-0.0327	11	4	7	2	13	2	12	10	4	6	2	12	2	11
7284.1564	7284.1877	-0.0313	11	4	7	1	12	1	12	10	4	6	1	11	1	11
7284.1564	7284.1876	-0.0312	11	4	7	2	11	2	12	10	2	8	2	10	2	11
7284.1564	7284.1992	-0.0428	11	4	7	0	11	2	12	10	6	4	0	10	2	11
7284.1564	7284.1959	-0.0395	11	6	5	2	12	0	12	10	4	6	2	11	0	11
7284.1564	7284.1925	-0.0361	11	4	7	1	12	2	14	10	4	6	1	11	2	13
7284.1564	7284.1934	-0.0370	11	4	7	2	13	1	14	10	4	6	2	12	1	13
7284.2394	7284.2597	-0.0203	11	4	7	1	12	1	13	10	4	6	1	11	1	12
7284.2394	7284.2596	-0.0202	11	4	7	2	11	2	13	10	4	6	2	10	2	12
7284.2394	7284.2649	-0.0255	11	4	7	2	13	2	13	10	4	6	2	12	2	12
7284.2394	7284.2755	-0.0361	11	4	7	2	12	2	13	10	4	6	2	11	2	12
7284.2394	7284.2622	-0.0228	11	4	7	1	10	1	11	10	4	6	1	9	1	10
7284.2394	7284.2621	-0.0227	11	4	7	1	12	1	11	10	4	6	1	11	1	10
7284.2394	7284.2621	-0.0227	11	6	5	0	11	0	11	10	8	2	0	10	0	10
7284.2394	7284.2553	-0.0159	11	4	7	2	11	0	11	10	4	6	2	11	2	10
7284.2394	7284.2696	-0.0302	11	4	7	0	11	2	11	10	4	6	0	10	2	10
7284.2394	7284.2633	-0.0239	11	4	7	2	12	2	11	10	6	4	2	11	2	10
7284.3142	7284.3317	-0.0175	11	2	9	2	12	2	12	10	2	8	2	11	2	11
7284.3142	7284.3427	-0.0285	11	6	5	2	11	2	12	10	4	6	2	10	2	11

7284.3142	7284.3344	-0.0202	11	2	9	2	11	2	10	10	2	8	2	10	2	9
7284.3142	7284.3254	-0.0112	11	4	7	2	10	2	10	10	2	8	2	9	2	9
7284.3142	7284.3313	-0.0171	11	2	9	2	11	1	12	10	4	6	2	10	1	11
7284.3142	7284.3322	-0.0180	11	4	7	1	12	2	12	10	4	6	1	11	2	11
7284.3142	7284.3348	-0.0206	11	0	11	1	10	2	10	10	8	2	1	9	0	9
7284.3142	7284.3339	-0.0197	11	4	7	2	11	1	10	10	4	6	2	10	1	9
7334.4213	7334.4183	0.0030	11	3	8	0	11	1	11	10	3	7	0	10	1	10
7334.4213	7334.4212	0.0001	11	3	8	0	11	2	13	10	3	7	0	10	2	12
7334.4213	7334.4182	0.0031	11	3	8	1	11	0	11	10	3	7	1	10	0	10
7334.4213	7334.4178	0.0035	11	3	8	1	11	1	11	10	3	7	1	10	1	10
7334.4213	7334.4207	0.0006	11	3	8	1	11	2	13	10	3	7	1	10	2	12
7334.4213	7334.4205	0.0008	11	3	8	2	13	0	13	10	3	7	2	12	0	12
7334.4213	7334.4204	0.0009	11	3	8	2	13	1	13	10	3	7	2	12	1	12
7334.4213	7334.4178	0.0035	11	3	8	2	13	2	11	10	3	7	2	12	2	10
7334.4213	7334.4233	-0.0020	11	3	8	2	13	2	15	10	3	7	2	12	2	14
7334.4213	7334.4191	0.0022	11	5	6	2	9	2	11	10	3	7	2	8	2	10
7334.5166	7334.5114	0.0052	11	3	8	0	11	1	12	10	3	7	0	10	1	11
7334.5166	7334.5114	0.0052	11	3	8	1	11	1	12	10	3	7	1	10	1	11
7334.5166	7334.5157	0.0009	11	3	8	1	12	0	12	10	1	9	1	11	0	11
7334.5166	7334.5162	0.0004	11	3	8	1	12	1	12	10	3	7	1	11	1	11
7334.5166	7334.5150	0.0016	11	3	8	1	12	2	14	10	3	7	1	11	2	13
7334.5166	7334.5263	-0.0097	11	3	8	2	12	2	14	10	3	7	2	11	2	13
7334.5166	7334.5170	-0.0004	11	3	8	2	13	1	12	10	3	7	2	12	1	11
7334.5166	7334.5177	-0.0011	11	3	8	2	13	1	14	10	3	7	2	12	1	13
7334.5166	7334.5138	0.0028	11	3	8	2	13	2	12	10	3	7	2	12	2	11
7334.5166	7334.5164	0.0002	11	3	8	2	13	2	14	10	3	7	2	12	2	13
7334.6082	7334.6103	-0.0021	11	3	8	1	10	1	11	10	3	7	1	9	1	10
7334.6082	7334.6118	-0.0036	11	3	8	1	12	1	11	10	3	7	1	11	1	10
7334.6082	7334.6093	-0.0011	11	3	8	1	12	1	13	10	3	7	1	11	1	12
7334.6082	7334.6154	-0.0072	11	3	8	1	12	2	11	10	3	7	1	11	2	10
7334.6082	7334.6149	-0.0067	11	3	8	1	12	2	13	10	3	7	1	11	2	12
7334.6082	7334.6255	-0.0173	11	3	8	2	11	2	13	10	3	7	2	10	2	12
7334.6082	7334.6114	-0.0032	11	3	8	2	12	1	11	10	3	7	2	11	1	10
7334.6082	7334.6144	-0.0062	11	3	8	2	12	1	13	10	3	7	2	11	1	12
7334.6082	7334.6143	-0.0061	11	3	8	2	12	2	13	10	3	7	2	11	2	12
7334.6082	7334.6095	-0.0013	11	3	8	2	13	2	13	10	3	7	2	12	2	12
7334.7046	7334.7119	-0.0073	11	3	8	1	10	2	10	10	3	7	1	9	2	9
7334.7046	7334.7096	-0.0050	11	3	8	1	12	2	12	10	5	5	1	11	2	11
7334.7046	7334.7052	-0.0006	11	3	8	2	10	0	10	10	3	7	2	9	0	9
7334.7046	7334.7090	-0.0044	11	3	8	2	11	1	10	10	5	5	2	10	1	9
7334.7046	7334.7064	-0.0018	11	3	8	2	11	1	12	10	3	7	2	10	1	11
7334.7046	7334.7104	-0.0058	11	3	8	2	11	2	10	10	5	5	0	10	2	9
7334.7046	7334.7197	-0.0151	11	5	6	2	11	2	12	10	3	7	0	10	2	11
7334.7046	7334.7080	-0.0034	11	5	6	2	12	0	12	10	5	5	2	11	0	11
7380.2885	7380.2788	0.0097	11	2	9	1	11	2	13	10	2	8	1	10	2	12
7380.2885	7380.2896	-0.0011	11	2	9	1	12	1	13	10	2	8	1	11	1	12
7380.2885	7380.2804	0.0081	11	2	9	1	12	2	14	10	2	8	1	11	2	13
7380.2885	7380.2501	0.0384	11	2	9	2	12	2	14	10	2	8	2	11	2	13
7380.2885	7380.2787	0.0098	11	2	9	2	13	0	13	10	2	8	2	12	0	12
7380.2885	7380.2786	0.0099	11	2	9	2	13	1	13	10	2	8	2	12	1	12
7380.2885	7380.2815	0.0070	11	2	9	2	13	1	14	10	2	8	2	12	1	13
7380.2885	7380.2896	-0.0011	11	2	9	2	13	2	13	10	2	8	2	12	2	12
7380.2885	7380.2810	0.0075	11	2	9	2	13	2	14	10	2	8	2	12	2	13

7380.2885	7380.2723	0.0162	11	2	9	2	13	2	15	10	2	8	2	12	2	14
7696.9190	7696.9658	-0.0468	12	0	12	0	12	2	14	11	0	11	0	11	2	13
7696.9190	7696.9660	-0.0470	12	0	12	1	12	2	14	11	0	11	1	11	2	13
7696.9190	7696.8914	0.0276	12	0	12	1	13	1	14	11	0	11	1	12	1	13
7696.9190	7696.9267	-0.0077	12	0	12	1	13	2	15	11	0	11	1	12	2	14
7696.9190	7696.9244	-0.0054	12	0	12	2	13	2	15	11	0	11	2	12	2	14
7696.9190	7696.9663	-0.0473	12	0	12	2	14	0	14	11	0	11	2	13	0	13
7696.9190	7696.9663	-0.0473	12	0	12	2	14	1	14	11	0	11	2	13	1	13
7696.9190	7696.9299	-0.0109	12	0	12	2	14	1	15	11	0	11	2	13	1	14
7696.9190	7696.8932	0.0258	12	0	12	2	14	2	14	11	0	11	2	13	2	13
7696.9190	7696.9287	-0.0097	12	0	12	2	14	2	15	11	0	11	2	13	2	14
7907.1033	7907.0941	0.0092	12	1	11	1	13	2	13	11	1	10	1	12	2	12
7907.1033	7907.0743	0.0290	12	1	11	2	12	1	11	11	1	10	2	11	1	10
7907.1033	7907.0794	0.0239	12	1	11	2	12	2	11	11	1	10	2	11	2	10
7907.1033	7907.0890	0.0143	12	1	11	2	12	2	13	11	1	10	2	12	2	12
7907.1033	7907.0815	0.0218	12	3	9	1	11	2	11	11	3	8	1	10	2	10
7907.1033	7907.0865	0.0168	12	3	9	2	12	1	13	11	1	10	2	11	1	12
7907.1033	7907.0510	0.0523	12	3	9	2	13	0	13	11	3	8	2	11	2	12
7907.2046	7907.1928	0.0118	12	1	11	1	11	1	12	11	1	10	1	10	1	11
7907.2046	7907.1963	0.0083	12	1	11	1	12	2	12	11	1	10	1	11	2	11
7907.2046	7907.2047	-0.0001	12	1	11	1	13	1	14	11	1	10	1	12	1	13
7907.2046	7907.1830	0.0216	12	1	11	1	13	2	14	11	1	10	1	12	2	13
7907.2046	7907.1901	0.0145	12	1	11	2	12	1	12	11	1	10	2	11	1	11
7907.2046	7907.1984	0.0062	12	1	11	2	12	2	14	11	1	10	2	11	2	13
7907.2046	7907.1823	0.0223	12	1	11	2	13	1	14	11	1	10	2	12	1	13
7907.2046	7907.1414	0.0632	12	1	11	2	13	2	14	11	1	10	2	12	2	13
7907.2046	7907.1869	0.0177	12	1	11	2	14	2	14	11	1	10	2	13	2	13
7907.2046	7907.1921	0.0125	12	3	9	2	11	2	12	11	1	10	0	11	0	11
7907.3227	7907.2961	0.0266	12	1	11	1	11	2	13	11	1	10	1	10	2	12
7907.3227	7907.3010	0.0217	12	1	11	1	12	1	13	11	1	10	1	11	1	12
7907.3227	7907.3155	0.0072	12	1	11	1	13	0	13	11	1	10	1	12	0	12
7907.3227	7907.3190	0.0037	12	1	11	1	13	1	13	11	1	10	1	12	1	12
7907.3227	7907.2950	0.0277	12	1	11	1	13	2	15	11	1	10	1	12	2	14
7907.3227	7907.3002	0.0225	12	1	11	2	12	1	13	11	1	10	2	12	1	12
7907.3227	7907.3184	0.0043	12	1	11	2	13	1	13	11	1	10	0	11	1	12
7907.3227	7907.2968	0.0259	12	1	11	2	13	2	15	11	1	10	2	12	2	14
7907.3227	7907.3019	0.0208	12	1	11	2	14	1	15	11	1	10	2	13	1	14
7907.3227	7907.2537	0.0690	12	1	11	2	14	2	15	11	1	10	2	13	2	14
7907.4127	7907.4154	-0.0027	12	1	11	0	12	1	12	11	1	10	0	11	1	11
7907.4127	7907.4029	0.0098	12	1	11	0	12	2	14	11	1	10	0	11	2	13
7907.4127	7907.4154	-0.0027	12	1	11	1	12	0	12	11	1	10	1	11	0	11
7907.4127	7907.4140	-0.0013	12	1	11	1	12	1	12	11	1	10	1	11	1	11
7907.4127	7907.4029	0.0098	12	1	11	1	12	2	14	11	1	10	1	11	2	13
7907.4127	7907.4179	-0.0052	12	1	11	2	10	2	12	11	1	10	2	9	2	11
7907.4127	7907.4146	-0.0019	12	1	11	2	11	2	12	11	3	8	2	9	2	11
7907.4127	7907.4050	0.0077	12	1	11	2	14	0	14	11	1	10	2	13	0	13
7907.4127	7907.4025	0.0102	12	1	11	2	14	1	14	11	1	10	2	13	1	13
7907.4127	7907.4151	-0.0024	12	1	11	2	14	2	12	11	1	10	2	13	2	11
7926.4773	7926.4744	0.0029	12	3	10	0	12	2	14	11	3	9	0	11	2	13
7926.4773	7926.4734	0.0039	12	3	10	1	12	2	14	11	3	9	1	11	2	13
7926.4773	7926.4843	-0.0070	12	3	10	1	13	1	14	11	3	9	1	12	1	13
7926.4773	7926.4787	-0.0014	12	3	10	1	13	2	15	11	3	9	1	12	2	14
7926.4773	7926.4665	0.0108	12	3	10	2	13	2	15	11	3	9	2	12	2	14

7926.4773	7926.4734	0.0039	12	3	10	2	14	0	14	11	3	9	2	13	0	13
7926.4773	7926.4734	0.0039	12	3	10	2	14	1	14	11	3	9	2	13	1	13
7926.4773	7926.4766	0.0007	12	3	10	2	14	1	15	11	3	9	2	13	1	14
7926.4773	7926.4831	-0.0058	12	3	10	2	14	2	14	11	3	9	2	13	2	13
7926.4773	7926.4774	-0.0001	12	3	10	2	14	2	15	11	3	9	2	13	2	14
7928.8607	7928.8932	-0.0325	12	6	6	0	12	2	14	11	6	5	0	11	2	13
7928.8607	7928.8883	-0.0276	12	6	6	1	12	1	12	11	6	5	1	11	1	11
7928.8607	7928.8933	-0.0326	12	6	6	1	12	2	14	11	6	5	1	11	2	13
7928.8607	7928.8925	-0.0318	12	6	6	2	14	0	14	11	6	5	2	13	0	13
7928.8607	7928.8930	-0.0323	12	6	6	2	14	1	14	11	6	5	2	13	1	13
7928.8607	7928.8712	-0.0105	12	6	7	0	12	2	14	11	6	6	0	11	2	13
7928.8607	7928.8663	-0.0056	12	6	7	1	12	1	12	11	6	6	1	11	1	11
7928.8607	7928.8713	-0.0106	12	6	7	1	12	2	14	11	6	6	1	11	2	13
7928.8607	7928.8705	-0.0098	12	6	7	2	14	0	14	11	6	6	2	13	0	13
7928.8607	7928.8710	-0.0103	12	6	7	2	14	1	14	11	6	6	2	13	1	13
7928.9577	7928.9688	-0.0111	12	6	6	1	11	2	13	11	6	5	1	10	2	12
7928.9577	7928.9679	-0.0102	12	6	6	1	13	2	15	11	6	5	1	12	2	14
7928.9577	7928.9894	-0.0317	12	6	6	2	13	2	15	11	6	5	2	12	2	14
7928.9577	7928.9717	-0.0140	12	6	6	2	14	1	15	11	6	5	2	13	1	14
7928.9577	7928.9696	-0.0119	12	6	6	2	14	2	15	11	6	5	2	13	2	14
7928.9577	7928.9467	0.0110	12	6	7	1	11	2	13	11	6	6	1	10	2	12
7928.9577	7928.9458	0.0119	12	6	7	1	13	2	15	11	6	6	1	12	2	14
7928.9577	7928.9673	-0.0096	12	6	7	2	13	2	15	11	6	6	2	12	2	14
7928.9577	7928.9496	0.0081	12	6	7	2	14	1	15	11	6	6	2	13	1	14
7928.9577	7928.9475	0.0102	12	6	7	2	14	2	15	11	6	6	2	13	2	14
7929.0375	7929.0418	-0.0043	12	6	6	1	13	1	14	11	6	5	1	12	1	13
7929.0375	7929.0515	-0.0140	12	6	6	1	13	2	14	11	6	5	1	12	2	13
7929.0375	7929.0411	-0.0036	12	6	6	2	12	2	14	11	6	5	2	11	2	13
7929.0375	7929.0509	-0.0134	12	6	6	2	13	1	14	11	6	5	2	12	1	13
7929.0375	7929.0517	-0.0142	12	6	6	2	14	2	14	11	6	5	2	13	2	13
7929.0375	7929.0196	0.0179	12	6	7	1	13	1	14	11	6	6	1	12	1	13
7929.0375	7929.0294	0.0081	12	6	7	1	13	2	14	11	6	6	1	12	2	13
7929.0375	7929.0189	0.0186	12	6	7	2	12	2	14	11	6	6	2	11	2	13
7929.0375	7929.0288	0.0087	12	6	7	2	13	1	14	11	6	6	2	12	1	13
7929.1000	7929.1301	-0.0301	12	4	8	1	11	2	11	11	2	9	1	10	2	10
7929.1000	7929.1228	-0.0228	12	4	8	2	13	2	13	11	4	7	2	12	2	12
7929.1000	7929.1006	-0.0006	12	4	9	2	13	2	13	11	4	8	2	12	2	12
7929.1000	7929.1251	-0.0251	12	6	6	1	13	2	13	11	6	5	1	12	2	12
7929.1000	7929.1208	-0.0208	12	6	6	2	12	1	13	11	4	7	2	11	1	12
7929.1000	7929.0702	0.0298	12	6	6	2	13	2	14	11	6	5	2	12	2	13
7929.1000	7929.1029	-0.0029	12	6	7	1	13	2	13	11	6	6	1	12	2	12
7929.1000	7929.0985	0.0015	12	6	7	2	12	1	13	11	4	8	2	11	1	12
7929.1000	7929.1413	-0.0413	12	10	2	2	12	2	13	11	8	3	2	11	2	12
7929.1000	7929.1190	-0.0190	12	10	3	2	12	2	13	11	8	4	2	11	2	12
7941.8073	7941.7281	0.0792	12	4	9	0	12	2	14	11	4	8	0	11	2	13
7941.8073	7941.7277	0.0796	12	4	9	1	12	2	14	11	4	8	1	11	2	13
7941.8073	7941.8127	-0.0054	12	4	9	1	13	1	14	11	4	8	1	12	1	13
7941.8073	7941.7712	0.0361	12	4	9	1	13	2	15	11	4	8	1	12	2	14
7941.8073	7941.7720	0.0353	12	4	9	2	13	2	15	11	4	8	2	12	2	14
7941.8073	7941.7277	0.0796	12	4	9	2	14	0	14	11	4	8	2	13	0	13
7941.8073	7941.7277	0.0796	12	4	9	2	14	1	14	11	4	8	2	13	1	13
7941.8073	7941.7698	0.0375	12	4	9	2	14	1	15	11	4	8	2	13	1	14
7941.8073	7941.8123	-0.0050	12	4	9	2	14	2	14	11	4	8	2	13	2	13

7941.8073	7941.7704	0.0369	12	4	9	2	14	2	15	11	4	8	2	13	2	14
7954.3592	7954.3352	0.0240	12	4	8	0	12	1	12	11	4	7	0	11	1	11
7954.3592	7954.3371	0.0221	12	4	8	0	12	2	14	11	4	7	0	11	2	13
7954.3592	7954.3352	0.0240	12	4	8	1	12	0	12	11	4	7	1	11	0	11
7954.3592	7954.3350	0.0242	12	4	8	1	12	1	12	11	4	7	1	11	1	11
7954.3592	7954.3371	0.0221	12	4	8	1	12	2	14	11	4	7	1	11	2	13
7954.3592	7954.3350	0.0242	12	4	8	2	10	2	12	11	2	9	2	10	2	11
7954.3592	7954.3374	0.0218	12	4	8	2	14	0	14	11	4	7	2	13	0	13
7954.3592	7954.3372	0.0220	12	4	8	2	14	1	14	11	4	7	2	13	1	13
7954.3592	7954.3352	0.0240	12	4	8	2	14	2	12	11	4	7	2	13	2	11
7954.3592	7954.3355	0.0237	12	6	6	0	12	0	12	11	4	7	2	9	2	11
7954.4076	7954.4014	0.0062	12	2	10	2	14	2	13	11	4	7	2	13	2	12
7954.4076	7954.4014	0.0062	12	4	8	0	12	1	13	11	6	5	0	11	1	12
7954.4076	7954.4043	0.0033	12	4	8	1	11	2	13	11	4	7	1	10	2	12
7954.4076	7954.4043	0.0033	12	4	8	1	12	1	13	11	4	7	1	11	1	12
7954.4076	7954.4014	0.0062	12	4	8	1	13	0	13	11	4	7	1	12	0	12
7954.4076	7954.4043	0.0033	12	4	8	1	13	2	15	11	4	7	1	12	2	14
7954.4076	7954.4123	-0.0047	12	4	8	2	13	2	15	11	4	7	2	12	2	14
7954.4076	7954.4043	0.0033	12	4	8	2	14	1	13	11	4	7	2	13	1	12
7954.4076	7954.4043	0.0033	12	4	8	2	14	1	15	11	4	7	2	13	1	14
7954.4076	7954.4043	0.0033	12	4	8	2	14	2	15	11	4	7	2	13	2	14
7954.4712	7954.4714	-0.0002	12	4	8	1	11	1	12	11	4	7	1	10	1	11
7954.4712	7954.4713	-0.0001	12	4	8	1	12	2	12	11	4	7	1	11	2	11
7954.4712	7954.4712	-0.0000	12	4	8	1	13	1	12	11	4	7	1	12	1	11
7954.4712	7954.4694	0.0018	12	4	8	1	13	1	14	11	4	7	1	12	1	13
7954.4712	7954.4734	-0.0022	12	4	8	1	13	2	14	11	4	7	1	12	2	13
7954.4712	7954.4715	-0.0003	12	4	8	2	12	1	12	11	4	7	2	11	1	11
7954.4712	7954.4695	0.0017	12	4	8	2	12	2	14	11	4	7	2	11	2	13
7954.4712	7954.4736	-0.0024	12	4	8	2	13	1	14	11	4	7	2	12	1	13
7954.4712	7954.4819	-0.0107	12	4	8	2	13	2	14	11	4	7	2	12	2	13
7954.4712	7954.4734	-0.0022	12	4	8	2	14	2	14	11	4	7	2	13	2	13
7954.5312	7954.5407	-0.0095	12	2	10	1	11	2	11	11	8	3	1	10	0	10
7954.5312	7954.5407	-0.0095	12	2	10	2	12	2	11	11	2	9	2	11	2	10
7954.5312	7954.5388	-0.0076	12	2	10	2	13	2	13	11	2	9	2	12	2	12
7954.5312	7954.5387	-0.0075	12	4	8	1	13	2	13	11	4	7	1	12	2	12
7954.5312	7954.5345	-0.0033	12	4	8	2	11	2	11	11	4	7	2	10	2	10
7954.5312	7954.5408	-0.0096	12	4	8	2	12	1	11	11	4	7	2	11	1	10
7954.5312	7954.5389	-0.0077	12	4	8	2	12	1	13	11	2	9	2	11	1	12
7954.5312	7954.5475	-0.0163	12	6	6	2	12	2	13	11	6	5	2	11	2	12

4.3. 3,3-Diethyl-1,1,2,2-tetracyanocyclopropane

Table 10: Transition frequencies for 3,3-diethyl-1,1,2,2-tetracyanocyclopropane. J is the rotational angular momentum quantum number, K_a and K_c are the projections of J onto the principal axes at the prolate and oblate symmetric top limits, respectively, and $I_{7,9}$, F_1, $I_{8,10}$ and F_2 are angular momentum quantum numbers obtained using the following coupling schemes between the rotational angular momentum and the nuclear spins of the four nitrogen nuclei ($I(^{14}N)$ = 1): $\mathbf{I_{7,9}} = \mathbf{I_{N7}} + \mathbf{I_{N9}}$, $\mathbf{F_1} = \mathbf{J} + \mathbf{I_{7,9}}$, $\mathbf{I_{8,10}} = \mathbf{I_{N8}} + \mathbf{I_{N10}}$ and $\mathbf{F_2} = \mathbf{F_1} + \mathbf{I_{8,10}}$

Observed	Calculated	Difference

Freq. /MHz	Freq. /MHz	/ MHz	J	K_a	K_c	$I_{7,9}$	F_1	$I_{8,10}$	F_2	J'	K'_a	K'_c	$I'_{7,9}$	F'_1	$I'_{8,10}$	F'_2
2330.7650	2330.7296	0.0354	2	1	2	2	1	2	1	1	0	1	2	1	2	2
2330.7650	2330.7358	0.0292	2	1	2	2	1	2	1	1	0	1	2	1	0	1
2330.7650	2330.7417	0.0233	2	1	2	2	4	2	4	1	0	1	2	1	2	3
2330.7650	2330.7371	0.0279	2	1	2	2	1	2	2	1	0	1	2	1	0	1
2330.7650	2330.7309	0.0341	2	1	2	2	1	2	2	1	0	1	2	1	2	2
2330.8375	2330.8131	0.0244	2	1	2	2	1	2	2	1	0	1	2	1	2	3
2330.8375	2330.8451	-0.0076	2	1	2	2	1	1	0	1	0	1	2	1	1	1
2330.8375	2330.8136	0.0239	2	1	2	2	4	2	2	1	0	1	2	1	2	1
2332.7409	2332.7126	0.0283	2	1	2	1	2	2	2	1	0	1	1	1	2	1
2332.7409	2332.7099	0.0310	2	1	2	1	1	2	3	1	0	1	1	2	0	2
2332.7409	2332.7190	0.0219	2	1	2	1	3	2	2	1	0	1	1	2	2	2
2332.7409	2332.7033	0.0376	2	1	2	1	3	2	1	1	0	1	1	2	2	0
2517.9606	2517.9619	-0.0013	2	2	1	2	4	2	6	1	1	0	2	3	2	5
2517.9613	2517.9701	-0.0088	2	2	1	2	4	1	4	1	1	0	2	3	1	3
2517.9613	2517.9819	-0.0206	2	2	1	2	4	0	4	1	1	0	2	3	2	3
2518.0282	2518.0206	0.0076	2	2	1	1	2	1	3	1	1	0	1	1	1	2
2518.0282	2518.0340	-0.0058	2	2	1	1	1	1	2	1	1	0	1	0	1	1
2518.0282	2518.0319	-0.0037	2	2	1	1	2	2	2	1	1	0	1	2	0	2
2518.1533	2518.1506	0.0027	2	2	1	2	4	1	5	1	1	0	2	3	1	4
2518.2571	2518.2660	-0.0089	2	2	1	1	3	1	3	1	1	0	1	2	1	2
2518.2571	2518.2429	0.0142	2	2	1	1	3	0	3	1	1	0	1	1	2	2
2518.2571	2518.2600	-0.0029	2	2	1	1	2	2	2	1	1	0	1	1	0	1
2518.3500	2518.3337	0.0163	2	2	1	2	4	1	3	1	1	0	2	3	1	2
2518.3500	2518.3655	-0.0155	2	2	1	2	4	2	3	1	1	0	2	3	2	2
2518.3500	2518.3467	0.0033	2	2	1	2	2	0	2	1	1	0	0	1	2	1
2518.3500	2518.3680	-0.0180	2	2	1	2	3	2	1	1	1	0	2	2	2	1
2518.4446	2518.4388	0.0058	2	2	1	1	3	2	5	1	1	0	1	2	2	4
2518.4446	2518.4392	0.0054	2	2	1	2	4	2	5	1	1	0	2	3	2	4
2518.4446	2518.4746	-0.0300	2	2	1	2	4	2	4	1	1	0	2	3	0	3
2518.4446	2518.4207	0.0239	2	2	1	1	2	2	4	1	1	0	1	1	2	3
2518.5875	2518.5976	-0.0101	2	2	1	1	3	2	4	1	1	0	1	2	2	3
2518.5875	2518.5770	0.0105	2	2	1	1	2	1	1	1	1	0	1	1	1	1
2518.6309	2518.6277	0.0032	2	2	1	1	3	1	4	1	1	0	1	2	1	3
2518.7549	2518.7560	-0.0011	2	2	1	2	3	2	5	1	1	0	2	2	2	4
2518.7549	2518.7605	-0.0056	2	2	1	1	2	0	2	1	1	0	1	1	2	1
2518.8927	2518.9075	-0.0148	2	2	1	0	2	2	4	1	1	0	2	1	2	3
2518.8927	2518.9049	-0.0122	2	2	1	2	3	1	3	1	1	0	2	2	1	2
2518.8927	2518.9209	-0.0282	2	2	1	2	3	0	3	1	1	0	2	2	2	2
2518.8927	2518.9018	-0.0091	2	2	1	0	2	1	3	1	1	0	2	1	1	2
2518.9742	2518.9751	-0.0009	2	2	1	1	3	2	3	1	1	0	1	2	2	2
2518.9742	2519.0073	-0.0331	2	2	1	0	2	2	3	1	1	0	0	1	2	2
2518.9742	2518.9702	0.0040	2	2	1	0	2	0	2	1	1	0	2	1	0	1
2519.2301	2519.2305	-0.0004	2	2	1	2	2	2	4	1	1	0	0	1	2	3
2519.2301	2519.2375	-0.0074	2	2	1	2	1	1	2	1	1	0	2	1	1	2
2519.2301	2519.2399	-0.0098	2	2	1	1	3	2	2	1	1	0	1	2	2	2
2520.1712	2520.1846	-0.0134	2	2	1	2	3	2	4	1	1	0	2	3	2	4
2520.2944	2520.3050	-0.0106	2	2	1	2	3	2	3	1	1	0	2	3	0	3
2520.2944	2520.2641	0.0303	2	2	1	1	1	2	2	1	1	0	1	1	2	3
2573.2561	2573.2479	0.0082	2	2	0	2	4	1	5	1	1	1	2	3	1	4
2573.3323	2573.3404	-0.0081	2	2	0	1	3	2	4	1	1	1	1	2	2	3
2573.3323	2573.3366	-0.0043	2	2	0	1	2	2	4	1	1	1	1	1	2	3

2573.3323	2573.3300	0.0023	2	2	0	1	2	1	3	1	1	1	1	1	1	2
2573.3323	2573.2985	0.0338	2	2	0	2	4	2	5	1	1	1	2	3	2	5
2573.7518	2573.7550	-0.0032	2	2	0	2	4	2	6	1	1	1	2	3	2	5
2573.8362	2573.8256	0.0106	2	2	0	1	3	1	4	1	1	1	1	2	1	3
2573.8362	2573.8586	-0.0224	2	2	0	0	2	1	2	1	1	1	2	1	1	1
2573.9506	2573.9313	0.0193	2	2	0	2	4	1	4	1	1	1	2	3	1	3
2574.0304	2574.0235	0.0069	2	2	0	2	3	2	4	1	1	1	2	2	2	4
2574.0304	2574.0137	0.0167	2	2	0	2	3	2	2	1	1	1	0	1	0	1
2574.0304	2573.9894	0.0410	2	2	0	0	2	2	4	1	1	1	2	1	2	3
2574.3412	2574.3309	0.0103	2	2	0	1	3	2	5	1	1	1	1	2	2	4
2574.3412	2574.3642	-0.0230	2	2	0	2	3	1	4	1	1	1	2	2	1	3
2574.4538	2574.4389	0.0149	2	2	0	1	3	1	3	1	1	1	1	2	1	2
2574.4538	2574.4200	0.0338	2	2	0	2	3	1	3	1	1	1	0	1	1	2
2574.4538	2574.4655	-0.0117	2	2	0	2	2	2	3	1	1	1	2	2	0	2
2574.9053	2574.9236	-0.0183	2	2	0	1	1	2	3	1	1	1	1	2	2	2
2574.9053	2574.9162	-0.0109	2	2	0	2	2	1	3	1	1	1	0	1	1	2
2574.9053	2574.9428	-0.0375	2	2	0	2	3	1	4	1	1	1	2	3	1	4
2574.9053	2574.8832	0.0221	2	2	0	2	4	0	4	1	1	1	2	1	2	3
2574.9053	2574.9369	-0.0316	2	2	0	2	2	1	3	1	1	1	2	2	1	3
2574.9053	2574.8877	0.0176	2	2	0	1	3	1	3	1	1	1	1	1	1	2
2574.9053	2574.9002	0.0051	2	2	0	2	2	2	3	1	1	1	2	3	0	3
2574.9053	2574.9192	-0.0139	2	2	0	2	2	2	3	1	1	1	2	2	2	2
2575.2442	2575.2494	-0.0052	2	2	0	2	3	2	5	1	1	1	2	3	2	5
2575.2442	2575.2154	0.0288	2	2	0	2	2	1	2	1	1	1	2	2	1	1
2575.2442	2575.2686	-0.0244	2	2	0	2	3	2	4	1	1	1	0	1	2	3
2575.2442	2575.2464	-0.0022	2	2	0	2	2	1	1	1	1	1	0	1	1	1
2575.2442	2575.2754	-0.0312	2	2	0	1	1	2	3	1	1	1	1	1	2	3
2575.2442	2575.2511	-0.0069	2	2	0	1	1	1	2	1	1	1	1	1	1	2
2575.4510	2575.4672	-0.0162	2	2	0	1	3	0	3	1	1	1	1	1	2	2
2575.4510	2575.4235	0.0275	2	2	0	1	3	2	2	1	1	1	1	1	0	1
3400.5779	3400.5723	0.0056	3	0	3	2	4	2	4	2	1	2	2	2	2	3
3400.5779	3400.5625	0.0154	3	0	3	2	4	1	4	2	1	2	2	3	1	3
3400.5779	3400.5599	0.0180	3	0	3	2	3	2	4	2	1	2	2	3	0	3
3400.5779	3400.5648	0.0131	3	0	3	2	2	2	4	2	1	2	2	1	2	3
3400.6524	3400.6615	-0.0091	3	0	3	2	4	2	6	2	1	2	2	3	2	5
3400.6524	3400.6549	-0.0025	3	0	3	2	4	2	2	2	1	2	2	3	2	1
3401.1790	3401.2121	-0.0331	3	0	3	2	5	2	7	2	1	2	2	4	2	6
3401.1790	3401.1732	0.0058	3	0	3	2	5	1	5	2	1	2	2	4	1	4
3401.1790	3401.1672	0.0118	3	0	3	2	5	0	5	2	1	2	2	4	2	4
3401.1790	3401.1788	0.0002	3	0	3	2	5	2	4	2	1	2	2	4	2	3
3440.5892	3440.5570	0.0322	3	1	3	1	4	1	4	2	0	2	1	3	1	3
3440.5892	3440.5686	0.0206	3	1	3	1	4	0	4	2	0	2	1	3	0	3
3440.5892	3440.5761	0.0131	3	1	3	1	3	2	3	2	0	2	1	2	0	2
3440.5892	3440.5694	0.0198	3	1	3	1	3	1	3	2	0	2	1	2	1	2
3440.5892	3440.6026	-0.0134	3	1	3	0	3	0	3	2	0	2	0	2	0	2
3440.6352	3440.6474	-0.0122	3	1	3	2	5	2	7	2	0	2	2	4	2	6
3440.6352	3440.6157	0.0195	3	1	3	1	4	2	6	2	0	2	1	3	2	5
3440.6352	3440.6485	-0.0133	3	1	3	2	5	1	5	2	0	2	2	4	1	4
3440.6352	3440.6622	-0.0270	3	1	3	2	5	0	5	2	0	2	2	4	0	4
3440.6352	3440.6439	-0.0087	3	1	3	1	3	2	5	2	0	2	1	2	2	4
3440.6352	3440.6595	-0.0243	3	1	3	0	3	1	4	2	0	2	0	2	1	3
3440.6352	3440.6597	-0.0245	3	1	3	2	5	2	3	2	0	2	2	4	2	2
3440.6352	3440.6196	0.0156	3	1	3	0	3	1	3	2	0	2	2	2	1	2

3440.8451	3440.8477	-0.0026	3	1	3	2	4	2	6	2	0	2	2	3	2	5
3440.8451	3440.8291	0.0160	3	1	3	1	3	1	4	2	0	2	1	2	1	3
3440.8451	3440.8602	-0.0151	3	1	3	1	2	2	4	2	0	2	1	1	2	3
3440.8451	3440.8754	-0.0303	3	1	3	2	3	1	4	2	0	2	2	2	1	3
3440.8451	3440.8621	-0.0170	3	1	3	0	3	2	4	2	0	2	0	2	2	3
3440.8451	3440.8660	-0.0209	3	1	3	2	4	0	4	2	0	2	2	2	2	3
3440.8451	3440.8096	0.0355	3	1	3	1	4	2	3	2	0	2	1	3	2	2
3440.8988	3440.9143	-0.0155	3	1	3	1	4	1	5	2	0	2	1	3	1	4
3440.8988	3440.8999	-0.0011	3	1	3	1	4	2	5	2	0	2	1	3	2	4
3440.8988	3440.9050	-0.0062	3	1	3	2	3	2	4	2	0	2	2	3	0	3
3440.8988	3440.8958	0.0030	3	1	3	1	4	1	3	2	0	2	1	3	1	2
3441.0297	3441.0657	-0.0360	3	1	3	2	5	2	6	2	0	2	2	4	2	5
3441.0297	3441.0087	0.0210	3	1	3	2	5	1	4	2	0	2	2	4	1	3
3441.0297	3441.0429	-0.0132	3	1	3	2	5	2	4	2	0	2	2	4	2	3
3441.0297	3441.0359	-0.0062	3	1	3	1	3	2	4	2	0	2	1	2	2	3
3441.1741	3441.1970	-0.0229	3	1	3	2	4	1	3	2	0	2	2	3	1	2
3441.1741	3441.1958	-0.0217	3	1	3	2	3	2	3	2	0	2	2	3	2	2
3441.2768	3441.2751	0.0017	3	1	3	1	4	2	4	2	0	2	1	3	2	3
3441.2768	3441.2809	-0.0041	3	1	3	1	3	0	3	2	0	2	1	2	2	2
3441.2768	3441.2792	-0.0024	3	1	3	1	2	2	3	2	0	2	1	1	2	2
3441.3504	3441.3500	0.0004	3	1	3	2	5	2	5	2	0	2	2	4	2	4
3441.3504	3441.3344	0.0160	3	1	3	2	1	2	3	2	0	2	2	1	2	3
3637.6745	3637.6620	0.0125	3	2	2	0	3	0	3	2	1	1	2	2	2	2
3637.6745	3637.6630	0.0115	3	2	2	2	2	2	2	2	1	1	2	2	2	2
3637.6745	3637.6625	0.0120	3	2	2	2	1	2	1	2	1	1	2	2	2	2
3637.6745	3637.6619	0.0126	3	2	2	2	1	2	3	2	1	1	2	2	2	2
3637.6745	3637.6611	0.0134	3	2	2	2	5	2	3	2	1	1	2	2	2	2
3637.6745	3637.6620	0.0125	3	2	2	0	3	2	2	2	1	1	2	2	2	2
3637.6745	3637.6630	0.0115	3	2	2	2	2	2	1	2	1	1	2	2	2	2
3637.8781	3637.8656	0.0125	3	2	2	2	1	2	1	2	1	1	2	1	0	1
3637.8781	3637.8641	0.0140	3	2	2	2	1	1	2	2	1	1	2	0	1	1
3637.8781	3637.8727	0.0054	3	2	2	1	3	2	1	2	1	1	1	2	2	1
3637.8781	3637.8643	0.0138	3	2	2	2	1	1	1	2	1	1	2	0	1	1
3637.8781	3637.8660	0.0121	3	2	2	2	2	0	2	2	1	1	2	1	0	1
3637.8781	3637.8640	0.0141	3	2	2	0	3	1	2	2	1	1	2	0	1	1
3637.8781	3637.8723	0.0058	3	2	2	1	4	2	2	2	1	1	1	2	2	1
3637.8781	3637.8725	0.0056	3	2	2	1	3	2	2	2	1	1	1	2	2	1
3637.8781	3637.8662	0.0119	3	2	2	2	2	2	1	2	1	1	2	1	0	1
3637.8781	3637.8652	0.0129	3	2	2	0	3	2	2	2	1	1	2	1	0	1
3637.9944	3638.0254	-0.0310	3	2	2	2	5	0	5	2	1	1	2	4	0	4
3637.9944	3637.9922	0.0022	3	2	2	0	3	1	3	2	1	1	0	2	1	2
3637.9944	3637.9954	-0.0010	3	2	2	1	3	2	3	2	1	1	1	2	0	2
3637.9944	3637.9945	-0.0001	3	2	2	2	1	2	3	2	1	1	2	4	2	2
3637.9944	3637.9952	-0.0008	3	2	2	1	4	2	3	2	1	1	1	2	0	2
3637.9944	3637.9937	0.0007	3	2	2	2	5	2	3	2	1	1	2	4	2	2
3638.0250	3638.0262	-0.0012	3	2	2	0	3	2	5	2	1	1	2	4	0	4
3638.0951	3638.0910	0.0041	3	2	2	1	3	1	3	2	1	1	1	2	1	2
3638.0951	3638.0908	0.0043	3	2	2	1	4	1	3	2	1	1	1	2	1	2
3638.0951	3638.1185	-0.0234	3	2	2	0	3	0	3	2	1	1	2	1	2	2
3638.0951	3638.0897	0.0054	3	2	2	2	5	2	3	2	1	1	2	0	2	2
3638.0951	3638.0914	0.0037	3	2	2	2	2	2	3	2	1	1	2	0	2	2
3638.0951	3638.0906	0.0045	3	2	2	0	3	0	3	2	1	1	2	0	2	2
3638.0951	3638.0905	0.0046	3	2	2	2	1	2	3	2	1	1	2	0	2	2

3638.2001	3638.2038	-0.0037	3	2	2	2	5	1	5	2	1	1	2	4	1	4
3638.2001	3638.2007	-0.0006	3	2	2	1	3	2	5	2	1	1	1	2	2	4
3638.2001	3638.2336	-0.0335	3	2	2	1	3	2	4	2	1	1	1	2	2	3
3638.2001	3638.2274	-0.0273	3	2	2	0	3	1	4	2	1	1	2	2	1	3
3638.2001	3638.2021	-0.0020	3	2	2	0	3	2	5	2	1	1	0	2	2	4
3638.2001	3638.2013	-0.0012	3	2	2	2	5	0	5	2	1	1	0	2	2	4
3638.2001	3638.2005	-0.0004	3	2	2	1	4	2	5	2	1	1	1	2	2	4
3638.2001	3638.2291	-0.0290	3	2	2	2	5	2	4	2	1	1	2	4	2	3
3638.6313	3638.6313	-0.0000	3	2	2	2	5	1	6	2	1	1	2	4	1	5
3638.6313	3638.6377	-0.0064	3	2	2	1	4	2	6	2	1	1	1	3	2	5
3638.6313	3638.6343	-0.0030	3	2	2	2	5	2	6	2	1	1	2	4	2	5
3638.6313	3638.6344	-0.0031	3	2	2	1	4	1	4	2	1	1	1	3	1	3
3638.6313	3638.6324	-0.0011	3	2	2	2	5	1	4	2	1	1	2	4	1	3
3638.6313	3638.6355	-0.0042	3	2	2	1	4	2	4	2	1	1	1	3	2	3
3638.6313	3638.6361	-0.0048	3	2	2	1	2	2	4	2	1	1	1	3	2	3
3638.6313	3638.6346	-0.0033	3	2	2	2	4	2	6	2	1	1	2	4	2	5
3638.9577	3638.9538	0.0039	3	2	2	2	3	2	5	2	1	1	2	4	2	4
3638.9577	3638.9539	0.0038	3	2	2	2	5	2	5	2	1	1	2	4	2	4
3638.9577	3638.9546	0.0031	3	2	2	1	4	1	5	2	1	1	1	3	1	4
3639.1135	3639.1085	0.0050	3	2	2	1	3	0	3	2	1	1	1	3	2	2
3639.1135	3639.1192	-0.0057	3	2	2	2	3	1	3	2	1	1	2	3	1	2
3639.1135	3639.1144	-0.0009	3	2	2	1	2	1	3	2	1	1	1	3	1	2
3639.1135	3639.1147	-0.0012	3	2	2	1	2	1	2	2	1	1	1	1	1	1
3639.1135	3639.1138	-0.0003	3	2	2	1	4	1	3	2	1	1	1	3	1	2
3639.1135	3639.1201	-0.0066	3	2	2	2	4	1	3	2	1	1	2	3	1	2
3639.1135	3639.1139	-0.0004	3	2	2	1	3	1	3	2	1	1	1	3	1	2
3639.1135	3639.1150	-0.0015	3	2	2	1	2	1	1	2	1	1	1	1	1	1
3639.1135	3639.1096	0.0039	3	2	2	1	2	2	3	2	1	1	1	3	2	2
3639.2250	3639.2299	-0.0049	3	2	2	2	4	1	5	2	1	1	2	3	1	4
3639.2250	3639.2235	0.0015	3	2	2	1	4	2	5	2	1	1	1	3	2	4
3639.2250	3639.2268	-0.0018	3	2	2	2	5	2	5	2	1	1	2	2	2	4
3639.2250	3639.2209	0.0041	3	2	2	2	4	1	4	2	1	1	2	3	1	3
3639.2250	3639.2194	0.0056	3	2	2	1	2	2	4	2	1	1	1	1	2	3
3639.2250	3639.2196	0.0054	3	2	2	2	4	0	4	2	1	1	2	3	0	3
3639.2250	3639.2312	-0.0062	3	2	2	2	4	2	3	2	1	1	0	2	0	2
3639.2250	3639.2315	-0.0065	3	2	2	1	2	1	3	2	1	1	1	1	1	2
3639.2250	3639.2268	-0.0018	3	2	2	2	3	2	5	2	1	1	2	2	2	4
3639.2250	3639.2308	-0.0058	3	2	2	1	4	1	3	2	1	1	1	1	1	2
3639.2644	3639.2748	-0.0104	3	2	2	2	4	2	6	2	1	1	2	3	2	5
3639.2644	3639.2744	-0.0100	3	2	2	2	5	2	6	2	1	1	2	3	2	5
3639.2644	3639.2750	-0.0106	3	2	2	2	4	2	5	2	1	1	2	3	2	5
3639.4625	3639.4610	0.0015	3	2	2	1	4	0	4	2	1	1	1	3	0	3
3639.4625	3639.4716	-0.0091	3	2	2	2	3	1	4	2	1	1	0	2	1	3
3639.4625	3639.4656	-0.0031	3	2	2	2	5	2	4	2	1	1	2	3	2	3
3639.4625	3639.4663	-0.0038	3	2	2	2	4	2	4	2	1	1	2	3	2	3
3639.5089	3639.5252	-0.0163	3	2	2	2	4	1	3	2	1	1	2	2	1	2
3639.5089	3639.5206	-0.0117	3	2	2	1	2	2	3	2	1	1	1	1	2	2
3639.5089	3639.5228	-0.0139	3	2	2	2	3	0	3	2	1	1	2	2	0	2
3639.5089	3639.5207	-0.0118	3	2	2	1	2	2	2	2	1	1	1	1	2	2
3639.5089	3639.5229	-0.0140	3	2	2	2	3	2	2	2	1	1	2	2	0	2
3639.5089	3639.5243	-0.0154	3	2	2	2	3	1	3	2	1	1	2	2	1	2
3639.5089	3639.5247	-0.0158	3	2	2	2	3	1	2	2	1	1	2	2	1	2
3639.5089	3639.5201	-0.0112	3	2	2	1	3	2	3	2	1	1	1	1	2	2

3639.5089	3639.5234	-0.0145	3	2	2	2	4	2	3	2	1	1	2	2	0	2
3639.5833	3639.5871	-0.0038	3	2	2	2	3	2	2	2	1	1	2	2	2	1
3639.5833	3639.5838	-0.0005	3	2	2	1	3	2	2	2	1	1	1	1	0	1
3639.5833	3639.5845	-0.0012	3	2	2	1	2	2	2	2	1	1	1	1	0	1
3639.5833	3639.5843	-0.0010	3	2	2	1	2	0	2	2	1	1	1	1	0	1
3639.6125	3639.6166	-0.0041	3	2	2	2	4	2	5	2	1	1	2	3	2	4
3639.6125	3639.5909	0.0216	3	2	2	2	3	1	2	2	1	1	0	2	1	1
3639.6125	3639.6157	-0.0032	3	2	2	2	3	2	5	2	1	1	2	3	2	4
3639.6125	3639.6160	-0.0035	3	2	2	2	3	2	4	2	1	1	2	3	2	4
3639.6125	3639.6161	-0.0036	3	2	2	2	5	0	5	2	1	1	2	3	2	4
3639.6125	3639.5877	0.0248	3	2	2	2	4	2	2	2	1	1	2	2	2	1
3639.7480	3639.7544	-0.0064	3	2	2	2	3	2	4	2	1	1	0	2	2	3
3639.7480	3639.7549	-0.0069	3	2	2	2	4	0	4	2	1	1	0	2	2	3
3639.7480	3639.7545	-0.0065	3	2	2	0	3	2	3	2	1	1	0	2	2	3
3639.8565	3639.8552	0.0013	3	2	2	0	3	2	3	2	1	1	2	3	2	2
3855.8935	3855.8912	0.0023	3	3	0	2	5	2	5	2	2	1	2	4	2	4
3855.8935	3855.8978	-0.0043	3	3	0	1	3	2	3	2	2	1	1	2	2	2
3856.0145	3856.0290	-0.0145	3	3	0	2	5	2	6	2	2	1	2	4	2	5
3856.0145	3856.0225	-0.0080	3	3	0	1	3	2	4	2	2	1	1	2	2	3
3856.0145	3856.0333	-0.0188	3	3	0	0	3	2	3	2	2	1	0	2	0	2
3856.0845	3856.0808	0.0037	3	3	0	2	5	1	6	2	2	1	2	4	1	5
3856.0845	3856.0982	-0.0137	3	3	0	2	5	2	4	2	2	1	2	4	2	3
3856.2231	3856.2247	-0.0016	3	3	0	2	5	2	7	2	2	1	2	4	2	6
3856.2231	3856.2445	-0.0214	3	3	0	2	5	1	5	2	2	1	2	4	1	4
3856.2231	3856.2461	-0.0230	3	3	0	2	5	0	5	2	2	1	2	4	0	4
3856.2231	3856.2323	-0.0092	3	3	0	1	3	2	5	2	2	1	1	2	2	4
3856.2231	3856.2495	-0.0264	3	3	0	1	4	2	4	2	2	1	1	3	2	3
3856.2231	3856.2403	-0.0172	3	3	0	0	3	1	4	2	2	1	0	2	1	3
3856.2231	3856.2426	-0.0195	3	3	0	2	4	2	4	2	2	1	2	3	2	3
3856.2940	3856.3056	-0.0116	3	3	0	1	2	1	3	2	2	1	1	1	1	2
3856.4408	3856.4392	0.0016	3	3	0	1	4	1	5	2	2	1	1	3	1	4
3856.4408	3856.4097	0.0311	3	3	0	2	4	1	5	2	2	1	2	3	1	4
3856.4408	3856.4425	-0.0017	3	3	0	1	2	2	4	2	2	1	1	1	2	3
3856.4408	3856.4131	0.0277	3	3	0	1	4	1	3	2	2	1	1	3	1	2
3856.4408	3856.4063	0.0345	3	3	0	0	3	1	3	2	2	1	0	2	1	2
3856.4408	3856.4104	0.0304	3	3	0	0	3	0	3	2	2	1	0	2	2	2
3856.4408	3856.4379	0.0029	3	3	0	2	4	2	3	2	2	1	2	3	2	2
3856.4408	3856.4265	0.0143	3	3	0	2	1	2	3	2	2	1	2	0	2	2
3856.6137	3856.5829	0.0308	3	3	0	1	4	2	6	2	2	1	1	3	2	5
3856.6137	3856.6113	0.0024	3	3	0	1	4	1	4	2	2	1	1	3	1	3
3856.6137	3856.6146	-0.0009	3	3	0	1	4	0	4	2	2	1	1	3	0	3
3856.6137	3856.5937	0.0200	3	3	0	2	4	2	2	2	2	1	2	3	2	1
3856.8357	3856.8311	0.0046	3	3	0	2	3	2	4	2	2	1	2	2	2	3
3856.8357	3856.8388	-0.0031	3	3	0	2	2	1	3	2	2	1	2	1	1	2
3856.8357	3856.8367	-0.0010	3	3	0	2	3	1	2	2	2	1	2	2	1	1
3856.8886	3856.9169	-0.0283	3	3	0	2	2	2	4	2	2	1	2	1	2	3
3856.9873	3856.9900	-0.0027	3	3	0	2	3	2	5	2	2	1	2	2	2	4
3856.9873	3857.0217	-0.0344	3	3	0	2	3	1	3	2	2	1	2	2	1	2
3858.1000	3858.1120	-0.0120	3	3	0	2	4	1	5	2	2	1	2	4	1	5
3858.1000	3858.1000	-0.0000	3	3	0	2	4	2	5	2	2	1	2	4	2	5
3858.1000	3858.0730	0.0270	3	3	0	2	4	2	4	2	2	1	2	4	2	4
3858.1000	3858.0924	0.0076	3	3	0	2	4	1	3	2	2	1	2	4	1	3
3858.1000	3858.1049	-0.0049	3	3	0	2	4	2	3	2	2	1	2	4	2	3

3858.1000	3858.1101	-0.0101	3	3	0	1	2	2	2	2	2	1	1	2	2	2
3858.1500	3858.1586	-0.0086	3	3	0	2	4	2	6	2	2	1	2	4	2	6
3858.1500	3858.1610	-0.0110	3	3	0	2	4	1	4	2	2	1	2	4	1	4
3858.1500	3858.1574	-0.0074	3	3	0	2	4	0	4	2	2	1	2	4	0	4
3858.1500	3858.1719	-0.0219	3	3	0	1	2	2	3	2	2	1	1	2	2	3
3858.1500	3858.1722	-0.0222	3	3	0	2	4	2	2	2	2	1	2	4	2	2
4534.5023	4534.5037	-0.0014	4	0	4	0	4	2	6	3	1	3	2	3	2	5
4534.5023	4534.4985	0.0038	4	0	4	2	3	2	5	3	1	3	2	2	2	4
4534.5023	4534.4972	0.0051	4	0	4	2	4	1	4	3	1	3	0	3	1	3
4534.5023	4534.5138	-0.0115	4	0	4	2	4	0	4	3	1	3	0	3	0	3
4534.5023	4534.5378	-0.0355	4	0	4	2	3	0	3	3	1	3	2	2	0	2
4534.5023	4534.4978	0.0045	4	0	4	0	4	2	2	3	1	3	0	3	2	1
4534.7375	4534.7178	0.0197	4	0	4	1	5	2	7	3	1	3	1	4	2	6
4534.7375	4534.7312	0.0063	4	0	4	2	5	2	7	3	1	3	2	4	2	6
4534.7375	4534.7317	0.0058	4	0	4	1	5	1	5	3	1	3	1	4	1	4
4534.7375	4534.7381	-0.0006	4	0	4	1	5	0	5	3	1	3	1	4	0	4
4534.7375	4534.7666	-0.0291	4	0	4	1	3	2	5	3	1	3	1	2	2	4
4534.7375	4534.7255	0.0120	4	0	4	2	5	0	5	3	1	3	2	4	0	4
4534.7375	4534.7239	0.0136	4	0	4	2	3	1	4	3	1	3	2	2	1	3
4534.8414	4534.8237	0.0177	4	0	4	1	5	1	6	3	1	3	1	4	1	5
4534.8414	4534.8501	-0.0087	4	0	4	1	5	2	6	3	1	3	1	4	2	5
4534.8414	4534.8017	0.0397	4	2	2	0	4	2	6	3	1	3	0	3	2	5
4534.8414	4534.8305	0.0109	4	0	4	1	4	2	6	3	1	3	1	3	2	5
4534.8414	4534.8392	0.0022	4	0	4	1	5	1	4	3	1	3	1	4	1	3
4534.8414	4534.8106	0.0308	4	0	4	2	2	2	4	3	1	3	2	2	2	3
4534.8414	4534.8027	0.0387	4	0	4	0	4	1	4	3	1	3	2	3	1	3
4534.8414	4534.8204	0.0210	4	0	4	1	4	0	4	3	1	3	1	3	2	3
4534.8414	4534.8211	0.0203	4	0	4	1	4	1	4	3	1	3	1	3	1	3
4534.8414	4534.8594	-0.0180	4	2	2	2	5	2	4	3	1	3	2	3	2	3
4534.9142	4534.9199	-0.0057	4	0	4	2	6	2	8	3	1	3	2	5	2	7
4534.9142	4534.9100	0.0042	4	0	4	2	6	1	6	3	1	3	2	5	1	5
4534.9142	4534.9078	0.0064	4	0	4	2	6	0	6	3	1	3	2	5	0	5
4534.9142	4534.8934	0.0208	4	0	4	2	5	1	6	3	1	3	2	4	1	5
4534.9142	4534.9427	-0.0285	4	0	4	2	5	2	6	3	1	3	2	4	2	5
4534.9142	4534.9273	-0.0131	4	0	4	1	5	2	5	3	1	3	1	4	2	4
4534.9142	4534.9503	-0.0361	4	0	4	0	4	2	5	3	1	3	0	3	2	4
4534.9142	4534.8978	0.0164	4	0	4	2	6	2	4	3	1	3	2	5	2	3
4534.9142	4534.9154	-0.0012	4	0	4	1	3	1	4	3	1	3	1	2	1	3
4534.9142	4534.9087	0.0055	4	0	4	1	5	2	4	3	1	3	1	4	2	3
4549.6092	4549.6178	-0.0086	4	1	4	2	3	2	5	3	0	3	2	2	2	4
4549.6092	4549.6253	-0.0161	4	1	4	2	2	1	2	3	0	3	2	1	1	1
4549.7375	4549.7351	0.0024	4	1	4	2	3	2	4	3	2	1	2	1	2	3
4549.7375	4549.7379	-0.0004	4	1	4	0	4	2	2	3	0	3	0	3	2	1
4549.7375	4549.7513	-0.0138	4	1	4	2	3	2	2	3	0	3	2	2	2	1
4549.8000	4549.8296	-0.0296	4	1	4	1	5	2	7	3	0	3	1	4	2	6
4549.8000	4549.7956	0.0044	4	1	4	2	4	2	6	3	0	3	2	3	2	5
4549.8000	4549.8121	-0.0121	4	1	4	0	4	2	6	3	0	3	0	3	2	5
4549.8000	4549.8172	-0.0172	4	1	4	1	5	1	5	3	0	3	1	4	1	4
4549.8000	4549.8279	-0.0279	4	1	4	1	5	0	5	3	0	3	1	4	0	4
4549.8000	4549.8130	-0.0130	4	1	4	2	2	2	4	3	0	3	2	1	2	3
4549.8000	4549.8019	-0.0019	4	1	4	0	4	1	4	3	0	3	0	3	1	3
4549.8000	4549.8139	-0.0139	4	1	4	2	3	1	4	3	0	3	2	2	1	3
4549.8000	4549.8156	-0.0156	4	1	4	1	5	2	3	3	0	3	1	4	2	2

4549.8000	4549.8043	-0.0043	4	1	4	2	4	2	2	3	0	3	2	3	2	1
4549.8375	4549.8650	-0.0275	4	1	4	1	4	0	4	3	0	3	1	3	0	3
4549.8375	4549.8572	-0.0197	4	1	4	1	4	1	4	3	0	3	1	3	1	3
4549.8375	4549.8689	-0.0314	4	1	4	2	3	1	2	3	0	3	2	2	1	1
4549.9159	4549.9139	0.0020	4	1	4	2	6	2	8	3	0	3	2	5	2	7
4549.9159	4549.9217	-0.0058	4	1	4	2	5	2	7	3	0	3	2	4	2	6
4549.9159	4549.9184	-0.0025	4	1	4	2	6	1	6	3	0	3	2	5	1	5
4549.9159	4549.9240	-0.0081	4	1	4	2	6	0	6	3	0	3	2	5	0	5
4549.9159	4549.8856	0.0303	4	1	4	1	4	2	6	3	0	3	1	3	2	5
4549.9159	4549.9428	-0.0269	4	1	4	2	4	2	5	3	0	3	2	4	2	4
4549.9159	4549.9176	-0.0017	4	1	4	1	3	2	5	3	0	3	1	2	2	4
4549.9159	4549.9386	-0.0227	4	1	4	2	5	1	5	3	0	3	2	4	1	4
4549.9159	4549.9305	-0.0146	4	1	4	2	6	2	4	3	0	3	2	5	2	3
4550.0408	4550.0235	0.0173	4	1	4	1	5	1	6	3	0	3	1	4	1	5
4550.0408	4550.0070	0.0338	4	1	4	1	5	2	6	3	0	3	1	4	2	5
4550.0408	4550.0583	-0.0175	4	1	4	1	4	1	5	3	0	3	1	3	1	4
4550.0408	4550.0043	0.0365	4	1	4	0	4	1	5	3	0	3	0	3	1	4
4550.0408	4550.0561	-0.0153	4	1	4	0	4	2	5	3	0	3	0	3	2	4
4550.0408	4550.0099	0.0309	4	1	4	1	5	1	4	3	0	3	1	4	1	3
4550.0408	4550.0278	0.0130	4	1	4	1	5	2	4	3	0	3	1	4	2	3
4550.0408	4550.0652	-0.0244	4	1	4	1	4	1	3	3	0	3	1	3	1	2
4550.1579	4550.1391	0.0188	4	1	4	2	6	2	7	3	0	3	2	5	2	6
4550.1579	4550.1262	0.0317	4	1	4	2	5	1	6	3	0	3	2	4	1	5
4550.1579	4550.1483	0.0096	4	1	4	2	5	2	6	3	0	3	2	4	2	5
4550.1579	4550.1193	0.0386	4	1	4	2	6	1	5	3	0	3	2	5	1	4
4550.1579	4550.1449	0.0130	4	1	4	2	6	2	5	3	0	3	2	5	2	4
4550.1579	4550.1275	0.0304	4	1	4	1	3	1	4	3	0	3	1	2	1	3
4550.1579	4550.1563	0.0016	4	1	4	2	5	1	4	3	0	3	2	4	1	3
4550.1579	4550.1580	-0.0001	4	1	4	2	4	2	4	3	2	1	2	4	2	3
4550.2563	4550.2297	0.0266	4	1	4	1	5	2	5	3	0	3	1	4	2	4
4550.2563	4550.2691	-0.0128	4	1	4	0	4	2	4	3	0	3	0	3	2	3
4550.2563	4550.2651	-0.0088	4	3	2	2	2	2	2	3	0	3	2	1	0	1
4550.3593	4550.3384	0.0209	4	1	4	2	6	2	6	3	0	3	2	5	2	5
4550.3593	4550.3732	-0.0139	4	1	4	2	5	2	5	3	2	1	2	4	2	4
4563.6341	4563.6380	-0.0039	4	1	3	2	4	2	4	3	2	2	0	3	2	3
4563.6703	4563.6936	-0.0233	4	1	3	2	4	2	5	3	2	2	2	3	2	4
4563.6703	4563.7071	-0.0368	4	1	3	2	4	1	3	3	2	2	2	3	1	2
4563.7914	4563.7830	0.0084	4	1	3	2	4	2	6	3	2	2	2	3	2	5
4563.7914	4563.7977	-0.0063	4	1	3	2	4	1	4	3	2	2	2	3	1	3
4563.7914	4563.8019	-0.0105	4	1	3	2	4	0	4	3	2	2	2	3	0	3
4563.7914	4563.8211	-0.0297	4	1	3	2	4	2	2	3	2	2	2	3	2	1
4564.3028	4564.3053	-0.0025	4	1	3	2	5	2	5	3	2	2	2	4	2	4
4564.3028	4564.3059	-0.0031	4	1	3	2	5	2	5	3	2	2	2	5	2	4
4564.3663	4564.3886	-0.0223	4	1	3	2	5	1	6	3	2	2	2	4	1	5
4564.3663	4564.3648	0.0015	4	1	3	2	5	2	6	3	2	2	2	4	2	5
4564.3663	4564.3753	-0.0090	4	1	3	2	5	1	4	3	2	2	2	4	1	3
4564.4618	4564.4477	0.0141	4	1	3	2	5	2	7	3	2	2	2	4	2	6
4564.4618	4564.4605	0.0013	4	1	3	2	5	1	5	3	2	2	2	4	1	4
4564.4618	4564.4636	-0.0018	4	1	3	2	5	0	5	3	2	2	2	4	0	4
4564.4618	4564.4689	-0.0071	4	1	3	1	3	2	4	3	2	2	1	2	2	3
4564.4618	4564.4790	-0.0172	4	1	3	2	5	2	3	3	2	2	2	4	2	2
4564.4618	4564.4836	-0.0218	4	1	3	1	3	1	2	3	2	2	1	2	1	1
4565.3502	4565.3479	0.0023	4	1	3	2	6	2	6	3	2	2	2	5	2	5

4738.6088	4738.6084	0.0004	4	2	3	1	4	0	4	3	1	2	1	3	0	3
4738.6088	4738.5938	0.0150	4	2	3	0	4	1	4	3	1	2	0	3	1	3
4738.6088	4738.6105	-0.0017	4	2	3	2	6	2	4	3	1	2	2	5	2	3
4738.6088	4738.6069	0.0019	4	2	3	2	2	2	4	3	1	2	2	1	2	3
4738.6088	4738.6068	0.0020	4	2	3	2	3	2	5	3	1	2	0	3	2	4
4739.0270	4739.0070	0.0200	4	2	3	1	4	1	5	3	1	2	1	3	1	4
4739.0270	4738.9972	0.0298	4	2	3	2	6	2	5	3	1	2	2	5	2	4
4739.0270	4738.9958	0.0312	4	2	3	1	3	0	3	3	1	2	1	2	0	2
4739.0833	4739.0804	0.0029	4	2	3	0	4	2	5	3	1	2	0	3	2	4
4739.0833	4739.0844	-0.0011	4	2	3	1	3	1	3	3	1	2	1	2	1	2
4739.0833	4739.0551	0.0282	4	2	3	0	4	1	3	3	1	2	0	3	1	2
4739.0833	4739.0746	0.0087	4	2	3	2	3	2	4	3	1	2	2	2	2	3
4739.0833	4739.0863	-0.0030	4	2	3	2	3	1	2	3	1	2	2	2	1	1
4739.1374	4739.1389	-0.0015	4	2	3	2	6	1	7	3	1	2	2	5	1	6
4739.1374	4739.1341	0.0033	4	2	3	1	4	2	5	3	1	2	1	3	2	4
4739.1374	4739.1106	0.0268	4	2	3	1	4	1	3	3	1	2	1	3	1	2
4739.1374	4739.1044	0.0330	4	2	3	2	5	2	3	3	1	2	2	4	2	2
4739.2125	4739.2031	0.0094	4	2	3	2	6	1	5	3	1	2	2	5	1	4
4739.2125	4739.2030	0.0095	4	2	3	1	3	2	5	3	1	2	1	2	2	4
4739.2125	4739.2058	0.0067	4	2	3	2	5	1	5	3	1	2	2	4	1	4
4739.2125	4739.1905	0.0220	4	2	3	2	5	0	5	3	1	2	2	4	0	4
4739.2295	4739.2475	-0.0180	4	2	3	2	6	2	7	3	1	2	2	5	2	6
4739.2295	4739.2662	-0.0367	4	2	3	2	5	2	7	3	1	2	2	4	2	6
4739.2295	4739.2367	-0.0072	4	2	3	1	5	2	4	3	1	2	1	4	2	3
4739.3325	4739.3212	0.0113	4	2	3	1	5	1	6	3	1	2	1	4	1	5
4739.3325	4739.3296	0.0029	4	2	3	0	4	2	4	3	1	2	0	3	2	3
4739.3325	4739.3364	-0.0039	4	2	3	2	4	0	4	3	1	2	2	3	0	3
4739.3325	4739.3654	-0.0329	4	2	3	2	4	1	4	3	1	2	2	3	1	3
4739.3325	4739.3644	-0.0319	4	2	3	1	4	2	4	3	1	2	1	3	2	3
4739.3625	4739.3834	-0.0209	4	2	3	2	5	2	4	3	1	2	2	4	2	3
4739.3625	4739.3939	-0.0314	4	2	3	2	2	2	4	3	1	2	2	2	2	4
4739.3625	4739.3744	-0.0119	4	2	3	0	4	2	5	3	1	2	2	2	2	4
4739.3625	4739.3823	-0.0198	4	2	3	2	5	2	6	3	1	2	2	4	2	6
4739.4547	4739.4751	-0.0204	4	2	3	2	5	1	6	3	1	2	2	4	1	5
4739.4547	4739.4395	0.0152	4	2	3	2	4	2	6	3	1	2	2	3	2	5
4739.4547	4739.4325	0.0222	4	2	3	1	5	1	4	3	1	2	1	4	1	3
4739.4547	4739.4404	0.0143	4	2	3	1	3	1	4	3	1	2	1	2	1	3
4739.4889	4739.4966	-0.0077	4	2	3	1	5	2	6	3	1	2	1	4	2	5
4739.4889	4739.5050	-0.0161	4	2	3	2	5	1	4	3	1	2	2	4	1	3
4739.5968	4739.6023	-0.0055	4	2	3	2	6	2	6	3	1	2	2	5	2	5
4739.5968	4739.6095	-0.0127	4	2	3	2	4	1	5	3	1	2	2	3	1	4
4739.5968	4739.6161	-0.0193	4	2	3	1	3	1	2	3	1	2	1	2	1	1
4967.3566	4967.3214	0.0352	4	3	2	2	2	1	2	3	2	1	2	1	1	1
4967.3566	4967.3628	-0.0062	4	3	2	2	2	1	2	3	2	1	2	2	1	2
4967.3566	4967.3908	-0.0342	4	3	2	1	4	2	2	3	2	1	1	4	2	2
4967.3566	4967.3705	-0.0139	4	3	2	0	4	2	5	3	2	1	0	3	2	5
4967.3566	4967.3757	-0.0191	4	3	2	0	4	2	2	3	2	1	0	3	2	1
4967.3566	4967.3636	-0.0070	4	3	2	2	2	0	2	3	2	1	2	2	0	2
4967.3566	4967.3238	0.0328	4	3	2	2	2	0	2	3	2	1	2	1	0	1
4967.3566	4967.3646	-0.0080	4	3	2	1	4	2	2	3	2	1	1	3	2	1
4967.4375	4967.4531	-0.0156	4	3	2	2	6	2	4	3	2	1	2	5	2	3
4967.4375	4967.4437	-0.0062	4	1	4	2	4	0	4	3	2	1	0	3	0	3
4967.5094	4967.4912	0.0182	4	3	2	0	4	1	4	3	2	1	0	3	1	3

4967.5125	4967.5221	-0.0096	4	3	2	1	4	1	4	3	2	1	1	3	1	3
4967.5125	4967.4764	0.0361	4	3	2	1	4	0	4	3	2	1	1	3	0	3
4967.5815	4967.6123	-0.0308	4	3	2	1	4	2	6	3	2	1	1	3	2	5
4967.5815	4967.5860	-0.0045	4	3	2	0	4	2	6	3	2	1	0	3	2	5
4967.5815	4967.5901	-0.0086	4	3	2	2	6	0	6	3	2	1	2	5	0	5
4967.6375	4967.6428	-0.0053	4	3	2	2	6	1	6	3	2	1	2	5	1	5
4967.7487	4967.7190	0.0297	4	3	2	2	2	1	3	3	2	1	2	1	1	2
4967.7487	4967.7446	0.0041	4	3	2	2	6	2	8	3	2	1	2	5	2	7
4967.8750	4967.8402	0.0348	4	3	2	0	4	1	5	3	2	1	0	3	1	4
4967.8750	4967.8617	0.0133	4	3	2	1	5	2	3	3	2	1	1	4	2	2
4967.8750	4967.8739	0.0011	4	3	2	1	5	2	6	3	2	1	1	3	2	5
4967.8750	4967.8703	0.0047	4	3	2	1	4	1	5	3	2	1	1	3	1	4
4967.8750	4967.8877	-0.0127	4	3	2	2	2	2	3	3	2	1	2	2	2	2
4967.9625	4967.9722	-0.0097	4	3	2	0	4	1	3	3	2	1	0	3	1	2
4967.9625	4967.9658	-0.0033	4	3	2	2	6	1	7	3	2	1	2	5	1	6
4967.9796	4968.0072	-0.0276	4	3	2	1	5	0	5	3	2	1	1	4	0	4
4967.9796	4968.0096	-0.0300	4	3	2	1	4	1	3	3	2	1	1	3	1	2
4968.0438	4968.0535	-0.0097	4	3	2	1	5	1	5	3	2	1	1	4	1	4
4968.0438	4968.0613	-0.0175	4	3	2	1	4	2	5	3	2	1	1	3	2	4
4968.1375	4968.1208	0.0167	4	3	2	2	6	1	5	3	2	1	2	5	1	4
4968.1375	4968.1428	-0.0053	4	3	2	1	3	0	3	3	2	1	1	2	0	2
4968.1375	4968.1512	-0.0137	4	3	2	2	5	0	5	3	2	1	2	4	0	4
4968.1375	4968.1455	-0.0080	4	3	2	1	5	2	4	3	2	1	1	4	2	3
4968.1375	4968.1515	-0.0140	4	3	2	1	5	2	7	3	2	1	1	4	2	6
4968.2072	4968.2166	-0.0094	4	3	2	2	3	1	4	3	2	1	2	2	1	3
4968.2125	4968.1748	0.0377	4	3	2	2	6	2	7	3	2	1	2	5	2	6
4968.2125	4968.1792	0.0333	4	3	2	1	3	1	3	3	2	1	1	2	1	2
4968.2125	4968.2094	0.0031	4	3	2	2	5	1	5	3	2	1	2	4	1	4
4968.2625	4968.2651	-0.0026	4	3	2	1	3	2	5	3	2	1	1	2	2	4
4968.2625	4968.2707	-0.0082	4	3	2	1	4	2	4	3	2	1	1	3	2	3
4968.2625	4968.2424	0.0201	4	3	2	0	4	2	4	3	2	1	0	3	2	3
4968.3465	4968.3289	0.0176	4	3	2	2	5	2	7	3	2	1	2	4	2	6
4968.3625	4968.3724	-0.0099	4	3	2	1	5	1	6	3	2	1	1	4	1	5
4968.3625	4968.3562	0.0063	4	3	2	2	6	2	6	3	2	1	2	5	2	5
4968.5190	4968.5203	-0.0013	4	3	2	1	3	1	4	3	2	1	1	2	1	3
4968.5190	4968.5180	0.0010	4	3	2	1	5	1	4	3	2	1	1	4	1	3
4968.5190	4968.5164	0.0026	4	3	2	2	4	2	4	3	2	1	2	3	2	3
4968.5190	4968.5510	-0.0320	4	3	2	2	4	0	4	3	2	1	2	3	0	3
4968.6068	4968.5990	0.0078	4	3	2	2	4	1	4	3	2	1	2	3	1	3
4968.6068	4968.5736	0.0332	4	3	2	1	5	2	6	3	2	1	1	4	2	5
4968.6068	4968.5979	0.0089	4	3	2	2	5	1	6	3	2	1	2	4	1	5
4968.6375	4968.6362	0.0013	4	3	2	2	6	2	5	3	0	3	2	4	0	4
4968.6375	4968.6409	-0.0034	4	3	2	1	3	2	4	3	2	1	1	3	2	4
4968.6375	4968.6621	-0.0246	4	3	2	2	4	2	6	3	2	1	2	4	2	6
4968.6375	4968.6369	0.0006	4	3	2	1	3	1	2	3	2	1	1	2	1	1
4968.7284	4968.7102	0.0182	4	3	2	2	4	2	6	3	2	1	2	3	2	5
4968.7310	4968.7423	-0.0113	4	3	2	1	5	2	5	3	2	1	1	4	2	4
4968.7310	4968.7460	-0.0150	4	3	2	2	5	1	4	3	2	1	2	4	1	3
4968.7902	4968.8097	-0.0195	4	3	2	2	4	2	2	3	2	1	2	3	2	1
4968.7902	4968.7983	-0.0081	4	3	2	2	5	2	6	3	2	1	2	4	2	5
4968.7902	4968.7792	0.0110	4	3	2	2	5	1	5	3	2	1	2	3	1	4
4968.7902	4968.8016	-0.0114	4	3	2	2	5	2	4	3	0	3	2	3	2	3
4968.9125	4968.9243	-0.0118	4	3	2	1	3	2	3	3	2	1	1	2	2	2

4968.9125	4968.9385	-0.0260	4	3	2	2	4	1	5	3	2	1	2	4	1	5
4968.9125	4968.9221	-0.0096	4	3	2	2	5	2	7	3	2	1	2	5	2	6
4968.9854	4968.9836	0.0018	4	3	2	2	4	1	5	3	2	1	2	3	1	4
4968.9854	4968.9999	-0.0145	4	3	2	2	5	0	5	3	2	1	2	2	2	4
4968.9854	4968.9873	-0.0019	4	3	2	2	5	2	5	3	2	1	2	5	2	5
4969.0385	4969.0346	0.0039	4	3	2	2	5	2	5	3	0	3	2	4	0	4
4969.0385	4969.0421	-0.0036	4	3	2	2	4	1	4	3	2	1	2	2	1	3
4969.1065	4969.1077	-0.0012	4	3	2	2	4	1	3	3	2	1	2	3	1	2
5060.0453	5060.0539	-0.0086	4	1	3	2	2	2	2	3	2	2	2	1	0	1
5060.0453	5060.0538	-0.0085	4	1	3	2	2	2	2	3	2	2	2	1	2	2
5060.1974	5060.1991	-0.0017	4	3	1	2	6	2	6	3	2	2	2	5	2	5
5060.1974	5060.1921	0.0053	4	3	1	2	2	2	3	3	2	2	2	1	2	2
5060.3585	5060.3502	0.0083	4	3	1	0	4	2	5	3	2	2	0	3	2	4
5060.3585	5060.3579	0.0006	4	3	1	1	4	2	5	3	2	2	1	4	0	4
5060.3585	5060.3578	0.0007	4	3	1	1	4	2	5	3	2	2	1	4	2	4
5060.4683	5060.4567	0.0116	4	1	3	1	3	2	3	3	2	2	1	3	2	2
5060.4683	5060.4368	0.0315	4	3	1	0	4	1	3	3	2	2	2	1	1	2
5060.4683	5060.4666	0.0017	4	3	1	2	3	2	3	3	2	2	0	3	2	2
5060.4683	5060.4551	0.0132	4	3	1	2	3	2	4	3	2	2	2	1	2	3
5060.4683	5060.4542	0.0141	4	3	1	2	3	2	4	3	2	2	2	2	2	4
5060.4683	5060.4369	0.0314	4	3	1	0	4	1	3	3	2	2	0	3	1	2
5060.4683	5060.4550	0.0133	4	3	1	2	3	2	4	3	2	2	0	3	0	3
5060.5000	5060.5024	-0.0024	4	3	1	2	6	2	7	3	2	2	2	5	2	6
5060.5000	5060.4736	0.0264	4	3	1	1	4	1	3	3	2	2	1	3	1	2
5060.5000	5060.4795	0.0205	4	3	1	2	2	1	3	3	2	2	0	3	1	2
5060.5625	5060.5699	-0.0074	4	3	1	1	4	1	5	3	2	2	1	3	1	4
5060.5625	5060.5547	0.0078	4	3	1	2	6	2	5	3	2	2	2	5	2	4
5060.5625	5060.5363	0.0262	4	1	3	2	4	1	5	3	2	2	0	3	1	4
5060.5625	5060.5667	-0.0042	4	3	1	2	6	1	5	3	2	2	2	5	1	4
5060.5625	5060.5489	0.0136	4	3	1	1	5	2	5	3	2	2	1	4	0	4
5060.5625	5060.5371	0.0254	4	1	3	2	4	1	5	3	2	2	2	5	1	4
5060.5625	5060.5659	-0.0034	4	3	1	2	6	1	5	3	2	2	0	3	1	4
5060.5625	5060.5488	0.0137	4	3	1	1	5	2	5	3	2	2	1	4	2	4
5060.6350	5060.6373	-0.0023	4	3	1	2	6	1	7	3	2	2	2	5	1	6
5060.6978	5060.7082	-0.0104	4	3	1	1	5	2	6	3	2	2	1	3	2	5
5060.6978	5060.7318	-0.0340	4	3	1	2	5	2	6	3	2	2	0	3	2	5
5060.6978	5060.7083	-0.0105	4	3	1	1	5	2	6	3	2	2	1	4	2	5
5060.7671	5060.7747	-0.0076	4	3	1	1	5	1	4	3	2	2	1	3	1	3
5060.7671	5060.7724	-0.0053	4	3	1	1	4	2	4	3	2	2	1	3	2	3
5060.7671	5060.7806	-0.0135	4	3	1	2	4	1	4	3	2	2	0	3	1	3
5060.7671	5060.7763	-0.0092	4	3	1	2	2	2	4	3	2	2	2	1	2	3
5060.7671	5060.7798	-0.0127	4	3	1	2	4	1	4	3	2	2	2	2	1	3
5060.7671	5060.7948	-0.0277	4	3	1	0	4	2	3	3	2	2	2	2	0	2
5060.7671	5060.7719	-0.0048	4	3	1	1	4	2	4	3	2	2	1	2	2	3
5060.7671	5060.7653	0.0018	4	3	1	2	2	2	2	3	2	2	2	1	2	1
5060.7671	5060.7773	-0.0102	4	3	1	2	2	2	4	3	2	2	0	3	2	3
5060.7671	5060.7749	-0.0078	4	3	1	1	5	1	4	3	2	2	1	4	1	3
5060.8625	5060.8656	-0.0031	4	3	1	1	4	2	3	3	2	2	1	2	2	2
5060.8625	5060.8733	-0.0108	4	3	1	2	3	1	4	3	2	2	2	2	1	3
5060.8625	5060.8742	-0.0117	4	3	1	2	3	1	4	3	2	2	0	3	1	3
5060.8625	5060.8745	-0.0120	4	3	1	2	3	1	4	3	2	2	2	4	1	3
5060.9570	5060.9612	-0.0042	4	3	1	2	6	2	8	3	2	2	2	5	2	7
5060.9570	5060.9382	0.0188	4	3	1	1	5	1	6	3	2	2	1	4	1	5

5060.9570	5060.9528	0.0042	4	3	1	2	6	1	6	3	2	2	2	5	1	5
5060.9570	5060.9545	0.0025	4	3	1	1	4	2	6	3	2	2	1	4	2	5
5060.9570	5060.9570	-0.0000	4	3	1	2	4	2	6	3	2	2	2	5	0	5
5060.9570	5060.9542	0.0028	4	3	1	2	5	2	5	3	2	2	2	4	2	4
5060.9570	5060.9460	0.0110	4	3	1	1	4	1	4	3	2	2	1	4	1	3
5061.0250	5061.0189	0.0061	4	3	1	1	3	2	4	3	2	2	1	2	2	3
5061.0250	5061.0233	0.0017	4	3	1	2	3	1	3	3	2	2	2	2	1	2
5061.0250	5061.0194	0.0056	4	3	1	1	3	2	4	3	2	2	1	3	2	3
5061.0250	5061.0256	-0.0006	4	3	1	2	3	1	3	3	2	2	2	3	1	3
5061.0250	5061.0197	0.0053	4	3	1	1	3	2	4	3	2	2	1	3	2	4
5061.1090	5061.1209	-0.0119	4	3	1	1	5	2	4	3	2	2	1	4	2	3
5061.1090	5061.1135	-0.0045	4	3	1	2	5	1	4	3	2	2	2	4	1	3
5061.1090	5061.1192	-0.0102	4	3	1	2	6	2	4	3	2	2	2	5	2	3
5061.1090	5061.1153	-0.0063	4	3	1	1	3	1	4	3	2	2	1	2	1	3
5061.1090	5061.1102	-0.0012	4	3	1	2	3	0	3	3	2	2	2	2	2	2
5061.1500	5061.1781	-0.0281	4	3	1	2	5	1	6	3	2	2	2	4	1	5
5061.1500	5061.1678	-0.0178	4	3	1	2	6	0	6	3	2	2	2	4	2	5
5061.1500	5061.1683	-0.0183	4	3	1	2	6	0	6	3	2	2	2	5	0	5
5061.2730	5061.2617	0.0113	4	3	1	1	5	2	7	3	2	2	1	4	2	6
5061.3125	5061.3348	-0.0223	4	3	1	1	5	1	5	3	2	2	1	4	1	4
5061.3125	5061.3301	-0.0176	4	3	1	1	3	2	5	3	2	2	1	2	2	4
5061.3125	5061.3125	-0.0000	4	3	1	2	4	1	5	3	2	2	2	3	1	4
5061.3125	5061.3120	0.0005	4	3	1	2	4	1	5	3	2	2	2	4	1	4
5061.3125	5061.3202	-0.0077	4	3	1	2	4	2	5	3	2	2	2	3	2	4
5061.3125	5061.3306	-0.0181	4	3	1	1	3	2	5	3	2	2	1	3	2	4
5061.3125	5061.3197	-0.0072	4	3	1	2	4	2	5	3	2	2	2	4	0	4
5061.3979	5061.3853	0.0126	4	3	1	2	5	2	7	3	2	2	2	4	2	6
5061.3979	5061.3648	0.0331	4	3	1	1	5	0	5	3	2	2	1	4	2	4
5061.3979	5061.3647	0.0332	4	3	1	1	5	0	5	3	2	2	1	3	2	4
5061.3979	5061.3642	0.0337	4	3	1	1	5	0	5	3	2	2	1	2	2	4
5061.4278	5061.4235	0.0043	4	3	1	1	3	1	3	3	2	2	1	2	1	2
5061.4278	5061.4081	0.0197	4	3	1	2	4	1	3	3	2	2	2	3	1	2
5061.4278	5061.4168	0.0110	4	3	1	2	5	2	3	3	2	2	2	4	2	2
5061.4278	5061.4293	-0.0015	4	3	1	1	5	2	3	3	2	2	1	4	2	2
5061.4278	5061.4321	-0.0043	4	3	1	2	5	2	4	3	2	2	2	4	2	4
5061.4278	5061.4324	-0.0046	4	3	1	2	5	2	4	3	2	2	2	4	2	3
5061.4278	5061.4286	-0.0008	4	3	1	1	5	2	3	3	2	2	1	2	0	2
5061.4278	5061.4329	-0.0051	4	3	1	2	5	2	4	3	2	2	0	3	2	3
5061.4278	5061.4322	-0.0044	4	3	1	2	5	2	4	3	2	2	2	3	2	3
5061.5241	5061.5341	-0.0100	4	3	1	2	5	0	5	3	2	2	2	4	0	4
5061.5241	5061.5206	0.0035	4	3	1	2	5	1	5	3	2	2	2	3	1	4
5061.5241	5061.5201	0.0040	4	3	1	2	5	1	5	3	2	2	2	4	1	4
5061.5241	5061.5346	-0.0105	4	3	1	2	5	0	5	3	2	2	2	3	2	4
5061.5241	5061.5200	0.0041	4	3	1	2	5	1	5	3	2	2	2	4	1	5
5061.5241	5061.5340	-0.0099	4	3	1	2	5	0	5	3	2	2	2	4	2	5
5061.6677	5061.6689	-0.0012	4	3	1	0	4	2	6	3	2	2	2	3	2	5
5061.6677	5061.6929	-0.0252	4	3	1	2	4	2	3	3	2	2	2	3	2	2
5061.7250	5061.7537	-0.0287	4	3	1	2	4	0	4	3	2	2	2	3	0	3
5061.7250	5061.7333	-0.0083	4	3	1	0	4	1	4	3	2	2	2	3	1	3
5061.8250	5061.8264	-0.0014	4	3	1	0	4	2	2	3	2	2	2	3	2	1
5061.8250	5061.8263	-0.0013	4	3	1	0	4	2	2	3	2	2	2	3	2	2
5133.6305	5133.6482	-0.0177	4	2	2	2	6	1	7	3	1	3	2	5	1	6
5133.6305	5133.6115	0.0190	4	2	2	1	4	1	5	3	1	3	1	3	1	4

5135.0486	5135.0544	-0.0058	4	2	2	1	5	2	7	3	1	3	1	4	2	6
5135.0486	5135.0858	-0.0372	4	2	2	2	5	2	7	3	1	3	2	4	2	6
5135.8495	5135.8386	0.0109	4	2	2	2	4	1	4	3	1	3	0	3	1	3
5135.8495	5135.8285	0.0210	4	2	2	2	6	2	6	3	1	3	2	3	2	5
5135.8495	5135.8711	-0.0216	4	0	4	0	4	0	4	3	1	3	0	3	0	3
5135.8495	5135.8437	0.0058	4	2	2	1	5	2	3	3	1	3	1	2	0	2
5135.8495	5135.8681	-0.0186	4	0	4	2	4	2	2	3	1	3	0	3	2	1
5135.8495	5135.8265	0.0230	4	2	2	0	4	2	4	3	1	3	2	4	2	3
5146.8409	5146.8489	-0.0080	4	4	1	2	3	2	5	3	3	0	2	3	2	5
5146.8409	5146.8476	-0.0067	4	4	1	2	3	1	3	3	3	0	2	3	1	3
5146.8409	5146.8504	-0.0095	4	4	1	2	3	0	3	3	3	0	2	3	0	3
5146.8409	5146.8791	-0.0382	4	4	1	2	3	2	4	3	3	0	2	3	2	4
5146.8409	5146.8643	-0.0234	4	4	1	2	3	2	3	3	3	0	2	3	2	3
5151.9066	5151.9113	-0.0047	4	4	0	2	3	2	5	3	3	1	2	3	2	5
5151.9066	5151.9145	-0.0079	4	4	0	2	3	1	4	3	3	1	2	3	1	4
5151.9066	5151.9201	-0.0135	4	4	0	2	3	1	3	3	3	1	2	3	1	3
5151.9066	5151.9276	-0.0210	4	4	0	2	3	0	3	3	3	1	2	3	0	3
5151.9066	5151.9064	0.0002	4	4	0	2	3	2	4	3	3	1	2	3	2	4
5151.9066	5151.9276	-0.0210	4	4	0	2	3	1	2	3	3	1	2	3	1	2
5151.9066	5151.8777	0.0289	4	2	2	2	3	0	3	3	1	3	2	3	0	3
5152.6289	5152.6280	0.0009	4	4	0	1	4	2	6	3	3	1	1	4	2	6
5152.6289	5152.6243	0.0046	4	4	0	1	4	1	5	3	3	1	1	4	1	5
5152.6289	5152.6265	0.0024	4	4	0	1	4	1	4	3	3	1	1	4	1	4
5152.6289	5152.6247	0.0042	4	4	0	1	4	0	4	3	3	1	1	4	0	4
5152.6289	5152.6029	0.0260	4	4	0	1	4	2	5	3	3	1	1	4	2	5
5152.6289	5152.6140	0.0149	4	4	0	1	4	1	3	3	3	1	1	4	1	3
5152.6289	5152.6281	0.0008	4	4	0	1	4	2	2	3	3	1	1	4	2	2
5152.6289	5152.6010	0.0279	4	4	0	1	4	2	4	3	3	1	1	4	2	4
5152.6289	5152.6628	-0.0339	4	2	2	2	2	0	2	3	3	1	2	2	2	2
5152.6289	5152.5930	0.0359	4	4	0	1	4	2	3	3	3	1	1	4	2	3
5153.8854	5153.8882	-0.0028	4	4	0	2	6	2	7	3	3	1	2	5	2	7
5153.8854	5153.8873	-0.0019	4	4	0	2	6	1	5	3	3	1	2	5	1	5
5153.9480	5153.9512	-0.0032	4	4	0	1	4	2	5	3	3	1	1	3	2	5
5153.9480	5153.9443	0.0037	4	4	0	2	6	2	6	3	3	1	2	5	2	6
5153.9480	5153.9462	0.0018	4	4	0	1	4	1	3	3	3	1	1	3	1	3
5154.0185	5154.0129	0.0056	4	2	2	0	4	2	5	3	3	1	0	3	2	5
5154.0185	5154.0184	0.0001	4	4	0	1	4	2	4	3	3	1	1	3	2	4
5154.1625	5154.1436	0.0189	4	4	0	2	6	2	6	3	3	1	2	5	2	5
5154.2095	5154.1929	0.0166	4	4	0	2	6	1	7	3	3	1	2	5	1	6
5154.2095	5154.1930	0.0165	4	4	0	2	6	2	7	3	3	1	2	5	2	6
5154.2095	5154.1949	0.0146	4	4	0	2	6	1	5	3	3	1	2	5	1	4
5154.2095	5154.1938	0.0157	4	4	0	2	6	2	5	3	3	1	2	5	2	4
5154.2625	5154.2396	0.0229	4	4	0	2	6	2	8	3	3	1	2	5	2	7
5154.2625	5154.2409	0.0216	4	4	0	2	6	1	6	3	3	1	2	5	1	5
5154.2625	5154.2399	0.0226	4	4	0	2	6	0	6	3	3	1	2	5	0	5
5154.2625	5154.2389	0.0236	4	4	0	2	6	2	4	3	3	1	2	5	2	3
5154.2916	5154.2862	0.0054	4	4	0	1	4	2	6	3	3	1	1	3	2	5
5154.2916	5154.2841	0.0075	4	4	0	0	4	1	5	3	3	1	0	3	1	4
5154.2916	5154.2824	0.0092	4	2	2	0	4	2	5	3	3	1	0	3	2	4
5154.2916	5154.2841	0.0075	4	4	0	1	4	0	4	3	3	1	1	3	0	3
5154.2916	5154.2877	0.0039	4	4	0	0	4	1	3	3	3	1	0	3	1	2
5154.2916	5154.2827	0.0089	4	2	2	0	4	2	3	3	3	1	0	3	2	2
5154.2916	5154.2775	0.0141	4	4	0	1	4	2	2	3	3	1	1	3	2	1

5154.4641	5154.4564	0.0077	4	4	0	1	5	1	6	3	3	1	1	4	1	5
5154.4641	5154.4568	0.0073	4	4	0	1	5	2	6	3	3	1	1	4	2	5
5154.4641	5154.4532	0.0109	4	4	0	2	5	1	5	3	3	1	2	4	1	4
5154.4641	5154.4547	0.0094	4	4	0	2	5	0	5	3	3	1	2	4	0	4
5154.4641	5154.4594	0.0047	4	4	0	1	5	1	4	3	3	1	1	4	1	3
5154.4641	5154.4592	0.0049	4	4	0	2	2	2	4	3	3	1	2	1	2	3
5154.4641	5154.4587	0.0054	4	4	0	2	5	2	3	3	3	1	2	4	2	2
5154.4934	5154.5031	-0.0097	4	4	0	1	5	2	7	3	3	1	1	4	2	6
5154.4934	5154.5011	-0.0077	4	4	0	1	5	1	5	3	3	1	1	4	1	4
5154.4934	5154.4966	-0.0032	4	4	0	1	5	0	5	3	3	1	1	4	0	4
5154.4934	5154.5029	-0.0095	4	2	2	2	3	0	3	3	3	1	2	2	2	2
5154.6051	5154.5991	0.0060	4	4	0	2	4	2	4	3	1	3	2	3	0	3
5154.6051	5154.5923	0.0128	4	4	0	2	3	2	4	3	3	1	2	2	2	3
5154.6051	5154.6075	-0.0024	4	4	0	2	3	1	2	3	3	1	2	2	1	1
5154.6882	5154.6940	-0.0058	4	4	0	2	4	1	5	3	3	1	2	3	1	4
5154.6882	5154.6771	0.0111	4	4	0	0	4	2	5	3	3	1	2	3	2	4
5154.6882	5154.6904	-0.0022	4	4	0	2	4	1	3	3	3	1	2	3	1	2
5154.6882	5154.6887	-0.0005	4	4	0	0	4	2	3	3	3	1	2	3	2	2
5154.7657	5154.7587	0.0070	4	4	0	2	4	2	6	3	3	1	2	3	2	5
5154.7657	5154.7359	0.0298	4	4	0	2	3	2	5	3	3	1	2	2	2	4
5154.7657	5154.7646	0.0011	4	4	0	2	4	1	4	3	3	1	2	3	1	3
5154.7657	5154.7610	0.0047	4	4	0	2	4	0	4	3	3	1	2	3	0	3
5154.7657	5154.7572	0.0085	4	4	0	2	3	1	3	3	3	1	2	2	1	2
5154.7657	5154.7511	0.0146	4	4	0	2	3	0	3	3	3	1	2	2	0	2
5154.7657	5154.7662	-0.0005	4	4	0	2	4	2	2	3	3	1	2	3	2	1
5390.0572	5390.0547	0.0025	4	3	2	2	6	2	6	3	0	3	2	5	2	5
5390.0572	5390.0619	-0.0047	4	3	2	0	4	2	5	3	0	3	0	3	2	4
5390.0572	5390.0262	0.0310	4	3	2	2	2	2	4	3	2	1	2	1	2	3
5391.6821	5391.6819	0.0002	4	3	2	2	6	2	8	3	0	3	2	5	2	7
5391.6821	5391.7132	-0.0311	4	3	2	2	3	2	5	3	0	3	2	2	2	4
5391.6821	5391.7080	-0.0259	4	3	2	2	3	2	4	3	0	3	2	2	2	3
5391.7324	5391.7645	-0.0321	4	3	2	2	6	1	6	3	0	3	2	5	1	5
5391.7324	5391.7648	-0.0324	4	3	2	2	6	0	6	3	0	3	2	5	0	5
5392.7346	5392.7407	-0.0061	4	3	2	2	5	2	5	3	2	1	2	4	2	4
5392.7346	5392.6930	0.0416	4	3	2	1	5	2	4	3	0	3	1	4	2	3
5394.3945	5394.4095	-0.0150	4	3	2	2	4	1	5	3	0	3	2	3	1	4
5394.3945	5394.3634	0.0311	4	3	2	2	4	2	5	3	0	3	2	3	2	4
5649.6759	5649.6640	0.0119	5	2	3	2	6	1	7	4	3	2	2	5	1	6
5649.6759	5649.6555	0.0204	5	2	3	2	6	2	7	4	3	2	2	5	2	6
5649.7125	5649.7124	0.0001	5	2	3	1	4	2	5	4	3	2	1	3	2	4
5649.7125	5649.6974	0.0151	5	2	3	2	6	1	5	4	3	2	2	5	1	4
5649.7125	5649.7080	0.0045	5	2	3	2	6	0	6	4	3	2	2	4	2	5
5650.3097	5650.3160	-0.0063	5	2	3	1	6	2	8	4	3	2	1	5	2	7
5650.3097	5650.3266	-0.0169	5	2	3	1	6	1	6	4	3	2	1	5	1	5
5650.3097	5650.3185	-0.0088	5	2	3	1	6	0	6	4	3	2	1	5	0	5
5650.8341	5650.8403	-0.0062	5	2	3	2	7	2	7	4	3	2	2	6	2	6
5651.0746	5651.0764	-0.0018	5	2	3	2	7	1	8	4	3	2	2	6	1	7
5651.0746	5651.0771	-0.0025	5	2	3	2	7	2	8	4	3	2	2	6	2	7
5651.0746	5651.0785	-0.0039	5	2	3	2	7	1	6	4	3	2	2	6	1	5
5659.9938	5659.9906	0.0032	5	0	5	2	4	2	6	4	1	4	2	3	2	5
5659.9938	5659.9836	0.0102	5	0	5	2	4	1	4	4	1	4	2	3	1	3
5660.0442	5660.0375	0.0067	5	0	5	2	5	2	7	4	1	4	2	4	2	6
5660.0442	5660.0385	0.0057	5	0	5	2	5	1	5	4	1	4	2	4	1	4

5660.0442	5660.0491	-0.0049	5	0	5	2	5	0	5	4	1	4	0	4	0	4
5660.0442	5660.0216	0.0226	5	0	5	2	4	0	4	4	1	4	2	3	0	3
5660.0442	5660.0495	-0.0053	5	0	5	2	5	2	3	4	1	4	0	4	2	2
5660.0442	5660.0727	-0.0285	5	0	5	2	3	1	3	4	1	4	2	2	1	2
5660.1500	5660.1513	-0.0013	5	0	5	1	6	1	6	4	1	4	1	5	1	5
5660.1500	5660.1571	-0.0071	5	0	5	1	6	0	6	4	1	4	1	5	0	5
5660.1500	5660.1564	-0.0064	5	0	5	2	5	2	6	4	1	4	2	4	2	5
5660.1500	5660.1597	-0.0097	5	0	5	2	6	1	6	4	1	4	2	5	1	5
5660.1500	5660.1405	0.0095	5	0	5	1	6	2	8	4	1	4	1	5	2	7
5660.1875	5660.1765	0.0110	5	0	5	2	6	2	8	4	1	4	2	5	2	7
5660.1875	5660.1806	0.0069	5	0	5	0	5	2	7	4	1	4	0	4	2	6
5660.1875	5660.1808	0.0067	5	0	5	1	4	2	6	4	1	4	1	3	2	5
5660.1875	5660.1752	0.0123	5	0	5	2	5	1	6	4	1	4	2	4	1	5
5660.1875	5660.1839	0.0036	5	0	5	0	5	1	5	4	1	4	0	4	1	4
5660.1875	5660.1705	0.0170	5	0	5	2	4	1	5	4	1	4	2	3	1	4
5660.1875	5660.1756	0.0119	5	0	5	1	6	2	4	4	1	4	1	5	2	3
5660.1875	5660.1776	0.0099	5	0	5	2	6	2	4	4	1	4	2	5	2	3
5660.1875	5660.1767	0.0108	5	0	5	2	3	0	3	4	1	4	2	2	2	2
5660.1875	5660.1983	-0.0108	5	0	5	1	5	2	7	4	1	4	1	4	2	6
5660.1875	5660.1995	-0.0120	5	0	5	2	6	0	6	4	1	4	2	5	0	5
5660.1875	5660.2021	-0.0146	5	0	5	1	5	0	5	4	1	4	1	4	0	4
5660.1875	5660.1992	-0.0117	5	0	5	1	5	1	5	4	1	4	1	4	1	4
5660.1875	5660.1905	-0.0030	5	0	5	1	4	1	4	4	1	4	1	3	1	3
5660.2584	5660.2617	-0.0033	5	0	5	2	7	2	9	4	1	4	2	6	2	8
5660.2584	5660.2373	0.0211	5	0	5	1	6	1	7	4	1	4	1	5	1	6
5660.2584	5660.2591	-0.0007	5	0	5	2	7	0	7	4	1	4	2	6	0	6
5660.2584	5660.2597	-0.0013	5	0	5	2	7	1	7	4	1	4	2	6	1	6
5660.2584	5660.2422	0.0162	5	0	5	1	6	2	7	4	1	4	1	5	2	6
5660.2584	5660.2896	-0.0312	5	0	5	0	5	2	6	4	1	4	0	4	2	5
5660.2584	5660.2394	0.0190	5	0	5	1	6	1	5	4	1	4	1	5	1	4
5660.2584	5660.2598	-0.0014	5	0	5	2	7	2	5	4	1	4	2	6	2	4
5660.2584	5660.2876	-0.0292	5	0	5	1	6	2	5	4	1	4	1	5	2	4
5660.2584	5660.2841	-0.0257	5	0	5	2	5	2	5	4	1	4	2	4	2	4
5660.2794	5660.3080	-0.0286	5	0	5	2	6	1	7	4	1	4	2	5	1	6
5660.2794	5660.3126	-0.0332	5	0	5	0	5	1	6	4	1	4	0	4	1	5
5660.2794	5660.3117	-0.0323	5	0	5	1	5	2	6	4	1	4	1	4	2	5
5660.4674	5660.4494	0.0180	5	0	5	2	7	2	7	4	1	4	2	6	2	6
5660.4674	5660.4735	-0.0061	5	0	5	2	6	2	6	4	1	4	2	5	2	5
5660.4674	5660.4403	0.0271	5	0	5	1	5	2	5	4	1	4	1	4	2	4
5664.9148	5664.9291	-0.0143	5	1	5	2	5	2	7	4	0	4	0	4	2	6
5664.9148	5664.9243	-0.0095	5	1	5	2	5	1	5	4	0	4	2	4	1	4
5664.9148	5664.9331	-0.0183	5	1	5	2	5	0	5	4	0	4	2	4	0	4
5664.9148	5664.9215	-0.0067	5	3	3	2	4	2	5	4	2	2	2	3	2	4
5664.9148	5664.9273	-0.0125	5	1	5	0	5	2	3	4	0	4	0	4	2	2
5664.9148	5664.8974	0.0174	5	1	5	2	3	1	3	4	0	4	2	2	1	2
5737.6711	5737.6821	-0.0110	5	1	4	2	5	2	7	4	2	3	2	4	2	6
5737.6711	5737.6819	-0.0108	5	1	4	2	5	1	5	4	2	3	2	4	1	4
5737.6711	5737.6833	-0.0122	5	1	4	2	5	0	5	4	2	3	2	4	0	4
5737.6711	5737.6834	-0.0123	5	1	4	2	5	2	3	4	2	3	2	4	2	2
5737.7296	5737.7396	-0.0100	5	1	4	2	5	2	5	4	2	3	2	4	2	4
5738.0284	5738.0368	-0.0084	5	1	4	2	6	2	8	4	2	3	2	5	2	7
5738.0284	5738.0335	-0.0051	5	1	4	2	6	1	6	4	2	3	2	5	1	5
5738.0284	5738.0322	-0.0038	5	1	4	2	6	0	6	4	2	3	2	5	0	5

5738.0284	5738.0491	-0.0207	5	1	4	2	6	2	5	4	2	3	2	5	2	4
5738.0284	5738.0319	-0.0035	5	1	4	2	6	2	4	4	2	3	2	5	2	3
5738.0918	5738.0580	0.0338	5	1	4	2	6	1	7	4	2	3	2	5	1	6
5738.0918	5738.0609	0.0309	5	1	4	2	6	2	7	4	2	3	2	5	2	6
5738.0918	5738.0838	0.0080	5	1	4	1	4	2	6	4	2	3	1	3	2	5
5738.0918	5738.0853	0.0065	5	1	4	2	6	2	6	4	2	3	2	5	2	5
5738.0918	5738.1042	-0.0124	5	1	4	1	4	1	5	4	2	3	1	3	1	4
5738.0918	5738.0568	0.0350	5	1	4	2	6	1	5	4	2	3	2	5	1	4
5738.0918	5738.1016	-0.0098	5	1	4	1	4	2	5	4	2	3	1	3	2	4
5738.0918	5738.0855	0.0063	5	1	4	1	4	1	4	4	2	3	1	3	1	3
5738.0918	5738.0896	0.0022	5	1	4	1	4	0	4	4	2	3	1	3	0	3
5738.0918	5738.1291	-0.0373	5	1	4	1	4	2	4	4	2	3	1	3	2	3
5738.1667	5738.1419	0.0248	5	1	4	1	6	2	8	4	2	3	1	5	2	7
5738.1667	5738.1577	0.0090	5	1	4	1	6	1	7	4	2	3	1	5	1	6
5738.1667	5738.1581	0.0086	5	1	4	1	6	2	7	4	2	3	1	5	2	6
5738.1667	5738.1435	0.0232	5	1	4	1	6	1	6	4	2	3	1	5	1	5
5738.1667	5738.1448	0.0219	5	1	4	1	6	0	6	4	2	3	1	5	0	5
5738.1667	5738.1727	-0.0060	5	1	4	1	6	2	6	4	2	3	1	5	2	5
5738.1667	5738.1573	0.0094	5	1	4	1	6	1	5	4	2	3	1	5	1	4
5738.1667	5738.1655	0.0012	5	1	4	1	6	2	5	4	2	3	1	5	2	4
5738.1667	5738.1473	0.0194	5	1	4	1	6	2	4	4	2	3	1	5	2	3
5738.2375	5738.2279	0.0096	5	1	4	2	4	1	5	4	2	3	2	3	1	4
5738.2375	5738.2410	-0.0035	5	1	4	2	4	2	5	4	2	3	2	3	2	4
5738.2375	5738.2008	0.0367	5	1	4	2	4	0	4	4	2	3	2	3	0	3
5738.2375	5738.2701	-0.0326	5	1	4	2	4	2	4	4	2	3	2	3	2	3
5738.2375	5738.2364	0.0011	5	1	4	2	4	1	3	4	2	3	2	3	1	2
5738.2375	5738.2427	-0.0052	5	1	4	2	4	2	3	4	2	3	2	3	2	2
5738.2375	5738.1992	0.0383	5	1	4	2	4	2	2	4	2	3	2	3	2	1
5738.5250	5738.5022	0.0228	5	1	4	2	7	2	9	4	2	3	2	6	2	8
5738.5250	5738.5183	0.0067	5	1	4	2	7	1	8	4	2	3	2	6	1	7
5738.5250	5738.5157	0.0093	5	1	4	2	7	2	8	4	2	3	2	6	2	7
5738.5250	5738.5012	0.0238	5	1	4	2	7	1	7	4	2	3	2	6	1	6
5738.5250	5738.5010	0.0240	5	1	4	2	7	0	7	4	2	3	2	6	0	6
5738.5250	5738.5162	0.0088	5	1	4	2	7	1	6	4	2	3	2	6	1	5
5738.5250	5738.5141	0.0109	5	1	4	2	7	2	6	4	2	3	2	6	2	5
5738.5410	5738.5327	0.0083	5	1	4	2	7	2	7	4	2	3	2	6	2	6
5738.5410	5738.5481	-0.0071	5	1	4	1	5	2	7	4	2	3	1	4	2	6
5738.5410	5738.5529	-0.0119	5	1	4	0	5	2	7	4	2	3	0	4	2	6
5738.5410	5738.5703	-0.0293	5	1	4	1	5	1	6	4	2	3	1	4	1	5
5738.5410	5738.5748	-0.0338	5	1	4	0	5	1	6	4	2	3	0	4	1	5
5738.5410	5738.5658	-0.0248	5	1	4	1	5	2	6	4	2	3	1	4	2	5
5738.5410	5738.5706	-0.0296	5	1	4	0	5	2	6	4	2	3	0	4	2	5
5738.5410	5738.5484	-0.0074	5	1	4	1	5	1	5	4	2	3	1	4	1	4
5738.5410	5738.5530	-0.0120	5	1	4	0	5	1	5	4	2	3	0	4	1	4
5738.5410	5738.5495	-0.0085	5	1	4	1	5	0	5	4	2	3	1	4	0	4
5739.0112	5739.0081	0.0031	5	1	4	2	4	2	6	4	2	3	2	4	2	6
5739.0112	5739.0089	0.0023	5	1	4	2	4	1	5	4	2	3	2	4	1	5
5739.0112	5739.0035	0.0077	5	1	4	2	4	0	4	4	2	3	2	4	0	4
5739.0112	5739.0051	0.0061	5	1	4	2	4	1	4	4	2	3	2	4	1	4
5739.0112	5739.0174	-0.0062	5	1	4	2	4	2	5	4	2	3	2	4	2	5
5739.0112	5739.0057	0.0055	5	1	4	2	4	1	3	4	2	3	2	4	1	3
5739.0112	5739.0299	-0.0187	5	1	4	2	4	2	4	4	2	3	2	4	2	4
5739.0112	5739.0111	0.0001	5	1	4	2	4	2	3	4	2	3	2	4	2	3

5739.0112	5738.9997	0.0115	5	1	4	2	4	2	2	4	2	3	2	4	2	2
5739.0112	5739.0148	-0.0036	5	1	4	2	3	2	5	4	2	3	2	3	2	4
5739.0940	5739.0902	0.0038	5	1	4	2	3	2	5	4	2	3	2	3	2	5
5739.0940	5739.1271	-0.0331	5	1	4	2	3	1	4	4	2	3	2	3	1	4
5739.0940	5739.0788	0.0152	5	1	4	2	3	0	3	4	2	3	2	3	0	3
5739.0940	5739.0810	0.0130	5	1	4	2	3	1	3	4	2	3	2	3	1	3
5739.0940	5739.0814	0.0126	5	1	4	1	5	2	7	4	2	3	1	5	2	7
5739.0940	5739.0835	0.0105	5	1	4	1	5	1	6	4	2	3	1	5	1	6
5739.0940	5739.0824	0.0116	5	1	4	1	5	0	5	4	2	3	1	5	0	5
5739.0940	5739.0820	0.0120	5	1	4	1	5	1	5	4	2	3	1	5	1	5
5739.0940	5739.0609	0.0331	5	1	4	2	3	2	1	4	2	3	2	3	2	1
5739.0940	5739.1258	-0.0318	5	1	4	2	3	2	2	4	2	3	2	3	2	2
5829.4953	5829.5045	-0.0092	5	2	4	2	3	0	3	4	3	1	2	2	0	2
5829.4953	5829.5262	-0.0309	5	2	4	1	5	2	3	4	1	3	1	4	2	2
5829.4953	5829.5040	-0.0087	5	2	4	0	5	2	3	4	1	3	0	4	2	2
5829.5816	5829.5910	-0.0094	5	2	4	2	7	2	5	4	1	3	2	6	2	4
5829.5816	5829.5783	0.0033	5	2	4	0	5	0	5	4	1	3	0	4	0	4
5829.5816	5829.6001	-0.0185	5	2	4	1	5	0	5	4	1	3	1	4	0	4
5829.5816	5829.6193	-0.0377	5	2	4	1	6	2	4	4	1	3	1	5	2	3
5829.5816	5829.6135	-0.0319	5	2	4	1	5	1	5	4	1	3	1	4	1	4
5829.5816	5829.5695	0.0121	5	2	4	2	4	2	2	4	1	3	2	3	2	1
5829.5816	5829.5907	-0.0091	5	2	4	0	5	1	5	4	1	3	0	4	1	4
5829.6448	5829.6443	0.0005	5	2	4	2	4	0	4	4	1	3	2	3	0	3
5829.6448	5829.6465	-0.0017	5	2	4	2	7	1	7	4	1	3	2	6	1	6
5829.6448	5829.6473	-0.0025	5	2	4	0	5	2	7	4	1	3	0	4	2	6
5829.6448	5829.6369	0.0079	5	2	4	2	3	2	5	4	1	3	2	2	2	4
5829.6448	5829.6653	-0.0205	5	2	4	2	4	1	4	4	1	3	2	3	1	3
5829.6448	5829.6589	-0.0141	5	2	4	1	4	2	2	4	1	3	1	3	2	1
5829.6448	5829.6410	0.0038	5	2	4	2	7	0	7	4	1	3	2	6	0	6
5829.6448	5829.6710	-0.0262	5	2	4	1	5	2	7	4	1	3	1	4	2	6
5829.6644	5829.6877	-0.0233	5	2	4	2	7	2	9	4	1	3	2	6	2	8
5829.6644	5829.6960	-0.0316	5	2	4	1	6	1	6	4	1	3	1	5	1	5
5829.6644	5829.6852	-0.0208	5	2	4	1	6	0	6	4	1	3	1	5	0	5
5829.7500	5829.7513	-0.0013	5	2	4	1	4	0	4	4	3	1	1	3	0	3
5829.7500	5829.7515	-0.0015	5	2	4	1	6	2	8	4	1	3	1	5	2	7
5829.7500	5829.7237	0.0263	5	2	4	2	4	2	6	4	1	3	2	3	2	5
5829.7500	5829.7749	-0.0249	5	2	4	1	4	1	4	4	1	3	1	3	1	3
5829.8426	5829.8390	0.0036	5	2	4	0	5	2	4	4	1	3	0	4	2	3
5829.8426	5829.8454	-0.0028	5	2	4	1	4	2	6	4	1	3	1	3	2	5
5829.8426	5829.8629	-0.0203	5	2	4	2	5	0	5	4	1	3	2	4	0	4
5829.8426	5829.8274	0.0152	5	2	4	2	3	1	4	4	1	3	2	2	1	3
5829.8426	5829.8580	-0.0154	5	2	4	1	5	2	4	4	1	3	1	4	2	3
5829.8426	5829.8758	-0.0332	5	2	4	2	5	1	5	4	1	3	2	4	1	4
5829.8426	5829.8477	-0.0051	5	2	4	2	6	1	6	4	1	3	2	5	1	5
5829.8426	5829.8437	-0.0011	5	2	4	2	6	0	6	4	1	3	2	5	0	5
5830.0122	5829.9960	0.0162	5	2	4	2	7	1	8	4	1	3	2	6	1	7
5830.0122	5830.0106	0.0016	5	2	4	1	5	1	4	4	1	3	1	4	1	3
5830.0122	5829.9867	0.0255	5	2	4	0	5	1	4	4	1	3	0	4	1	3
5830.0122	5829.9856	0.0266	5	2	4	2	4	2	5	4	1	3	2	3	2	4
5830.0122	5829.9939	0.0183	5	2	4	2	3	2	4	4	1	3	2	2	2	3
5830.0538	5830.0932	-0.0394	5	2	4	2	7	2	8	4	1	3	2	6	2	7
5830.0538	5830.0602	-0.0064	5	2	4	1	6	1	7	4	1	3	1	5	1	6
5830.0538	5830.0524	0.0014	5	2	4	2	7	1	6	4	1	3	2	6	1	5

5830.0538	5830.0623	-0.0085	5	2	4	1	5	2	6	4	1	3	1	4	2	5
5830.0538	5830.0385	0.0153	5	2	4	0	5	2	6	4	1	3	0	4	2	5
5830.0538	5830.0892	-0.0354	5	2	4	2	6	2	5	4	1	3	2	5	2	4
5830.0538	5830.0607	-0.0069	5	2	4	2	5	2	4	4	1	3	2	4	2	3
5830.1606	5830.1601	0.0005	5	2	4	1	6	2	7	4	1	3	1	5	2	6
5830.1606	5830.1328	0.0278	5	2	4	2	6	1	7	4	1	3	2	5	1	6
5830.1606	5830.1708	-0.0102	5	2	4	2	5	1	6	4	1	3	0	4	1	5
5830.1606	5830.1889	-0.0283	5	2	4	2	6	1	5	4	1	3	2	5	1	4
5830.1606	5830.1636	-0.0030	5	2	4	2	4	2	4	4	1	3	2	3	2	3
5830.1606	5830.1700	-0.0094	5	2	4	2	3	2	3	4	1	3	2	2	0	2
5830.2438	5830.2141	0.0297	5	2	4	2	6	2	7	4	1	3	2	5	2	6
5830.2438	5830.2538	-0.0100	5	2	4	2	5	2	6	4	1	3	2	4	2	5
5830.2438	5830.2524	-0.0086	5	2	4	1	4	2	5	4	1	3	1	3	2	4
5830.2438	5830.2354	0.0084	5	2	4	2	5	1	4	4	1	3	2	4	1	3
5830.2438	5830.2124	0.0314	5	2	4	1	4	1	3	4	1	3	1	3	1	2
5830.3271	5830.3201	0.0070	5	2	4	1	5	2	5	4	1	3	1	4	2	4
5830.3271	5830.2960	0.0311	5	2	4	0	5	2	5	4	1	3	0	4	2	4
5830.3987	5830.3930	0.0057	5	2	4	2	7	2	7	4	1	3	2	6	2	6
5830.4667	5830.4504	0.0163	5	2	4	2	6	2	6	4	1	3	2	5	2	5
5830.4667	5830.4725	-0.0058	5	2	4	2	5	2	5	4	1	3	2	4	2	4
5830.4667	5830.4634	0.0033	5	2	4	1	6	2	6	4	1	3	1	5	2	5
5830.4667	5830.4907	-0.0240	5	2	4	1	4	2	4	4	1	3	1	3	0	3
6070.9202	6070.9176	0.0026	5	3	3	2	3	2	2	4	2	2	2	2	2	1
6070.9202	6070.9194	0.0008	5	5	1	2	3	2	3	4	4	0	2	2	2	2
6071.0127	6070.9943	0.0184	5	3	3	0	5	2	3	4	2	2	2	2	2	2
6071.0127	6070.9929	0.0198	5	3	3	2	3	1	3	4	2	2	2	2	1	2
6071.0127	6070.9959	0.0168	5	3	3	1	5	2	3	4	2	2	1	4	2	2
6071.0127	6070.9846	0.0281	5	3	3	2	3	2	1	4	2	2	2	2	2	1
6071.0875	6071.1134	-0.0259	5	3	3	1	5	1	5	4	2	2	1	4	1	4
6071.0875	6071.0885	-0.0010	5	3	3	1	5	0	5	4	2	2	1	4	0	4
6071.0875	6071.0878	-0.0003	5	3	3	0	5	1	5	4	2	2	0	4	1	4
6071.0875	6071.0882	-0.0007	5	3	3	2	3	2	5	4	2	2	2	2	2	4
6071.0875	6071.1134	-0.0259	5	3	3	2	7	2	5	4	2	2	2	6	2	4
6071.1978	6071.1910	0.0068	5	3	3	2	7	1	7	4	2	2	2	6	1	6
6071.1978	6071.1891	0.0087	5	3	3	1	5	2	7	4	2	2	1	4	2	6
6071.1978	6071.1900	0.0078	5	3	3	2	7	0	7	4	2	2	2	6	0	6
6071.1978	6071.1988	-0.0010	5	3	3	2	3	1	4	4	2	2	2	2	1	3
6071.1978	6071.2003	-0.0025	5	5	1	2	5	2	4	4	4	0	2	4	2	3
6071.1978	6071.2020	-0.0042	5	3	3	1	5	2	4	4	2	2	1	4	2	3
6071.1978	6071.2235	-0.0257	5	3	3	2	4	2	3	4	2	2	2	3	2	2
6071.2675	6071.2589	0.0086	5	3	3	2	7	2	9	4	2	2	2	6	2	8
6071.2675	6071.2311	0.0364	5	3	3	2	3	2	4	4	2	2	2	2	2	3
6071.4171	6071.4062	0.0109	5	3	3	2	7	2	6	4	4	0	2	4	2	5
6071.4171	6071.4080	0.0091	5	3	3	1	6	0	6	4	2	2	1	5	0	5
6071.4171	6071.4057	0.0114	5	3	3	2	4	2	6	4	2	2	2	6	2	5
6071.4171	6071.4034	0.0137	5	3	3	0	5	1	6	4	2	2	0	4	1	5
6071.4171	6071.4061	0.0110	5	3	3	1	5	1	6	4	2	2	1	4	1	5
6071.4750	6071.4655	0.0095	5	3	3	1	5	1	4	4	2	2	1	4	1	3
6071.4750	6071.4585	0.0165	5	3	3	2	3	1	2	4	2	2	2	2	1	1
6071.4750	6071.4728	0.0022	5	3	3	1	4	2	2	4	2	2	1	3	2	1
6071.4750	6071.4692	0.0058	5	3	3	1	6	2	4	4	2	2	1	5	2	3
6071.4750	6071.4620	0.0130	5	3	3	2	4	1	4	4	2	2	2	3	1	3
6071.4750	6071.4655	0.0095	5	3	3	2	4	2	2	4	2	2	2	3	2	1

6071.5892	6071.5734	0.0158	5	3	3	2	7	1	8	4	2	2	2	6	1	7
6071.5892	6071.5803	0.0089	5	3	3	1	6	2	8	4	2	2	1	5	2	7
6071.5892	6071.5767	0.0125	5	3	3	2	7	2	8	4	2	2	2	6	2	7
6071.5892	6071.5750	0.0142	5	3	3	2	7	1	6	4	2	2	2	6	1	5
6071.5892	6071.5786	0.0106	5	3	3	1	4	2	6	4	2	2	1	4	2	5
6071.5892	6071.5769	0.0123	5	3	3	1	6	1	6	4	2	2	1	5	1	5
6071.5892	6071.5729	0.0163	5	5	1	2	5	2	6	4	2	2	2	3	2	5
6071.6427	6071.6321	0.0106	5	3	3	2	4	1	5	4	2	2	2	3	1	4
6071.6427	6071.6312	0.0115	5	3	3	0	5	2	5	4	2	2	0	4	2	4
6071.6427	6071.6305	0.0122	5	3	3	1	6	2	5	4	2	2	1	5	2	4
6071.6427	6071.6354	0.0073	5	3	3	1	4	0	4	4	2	2	1	3	0	3
6071.6427	6071.6310	0.0117	5	5	1	2	4	2	4	4	4	0	2	3	2	3
6071.6427	6071.6270	0.0157	5	3	3	0	5	1	4	4	2	2	0	4	1	3
6071.7625	6071.7528	0.0097	5	3	3	2	5	2	5	4	2	2	2	4	2	4
6071.7625	6071.7397	0.0228	5	3	3	1	4	1	4	4	2	2	1	3	1	3
6071.7625	6071.7398	0.0227	5	3	3	2	6	2	4	4	4	0	2	3	2	3
6071.7625	6071.7712	-0.0087	5	3	3	2	6	2	7	4	2	2	2	5	2	7
6071.8375	6071.7996	0.0379	5	3	3	2	5	2	3	4	2	2	2	4	2	2
6071.8375	6071.8030	0.0345	5	3	3	1	4	2	3	4	2	2	1	3	2	2
6071.8625	6071.8463	0.0162	5	3	3	1	5	2	6	4	2	2	1	4	2	5
6071.8625	6071.8471	0.0154	5	5	1	2	6	2	6	4	4	0	2	5	2	5
6071.8625	6071.8481	0.0144	5	3	3	2	6	1	6	4	2	2	2	5	1	5
6071.8875	6071.9180	-0.0305	5	3	3	2	6	2	8	4	2	2	2	5	2	7
6071.8875	6071.8947	-0.0072	5	3	3	1	6	1	7	4	2	2	1	5	1	6
6071.8875	6071.8943	-0.0068	5	3	3	2	7	2	7	4	2	2	2	6	2	6
6071.9875	6072.0065	-0.0190	5	1	5	0	5	0	5	4	0	4	0	4	0	4
6071.9875	6071.9750	0.0125	5	3	3	1	5	2	5	4	2	2	1	4	2	4
6071.9875	6071.9848	0.0027	5	3	3	2	5	1	5	4	2	2	2	4	1	4
6071.9875	6072.0066	-0.0191	5	3	3	1	4	1	5	4	2	2	1	3	1	4
6071.9875	6071.9799	0.0076	5	3	3	1	6	1	5	4	2	2	1	5	1	4
6072.0583	6072.0507	0.0076	5	3	3	2	6	1	7	4	2	2	2	5	1	6
6072.0583	6072.0490	0.0093	5	3	3	2	5	2	7	4	0	4	2	4	2	6
6072.0583	6072.0469	0.0114	5	3	3	1	6	2	7	4	2	2	1	5	2	6
6072.2271	6072.2226	0.0045	5	3	3	1	4	2	5	4	2	2	1	3	2	4
6072.2271	6072.2253	0.0018	5	3	3	2	5	0	5	4	2	2	2	4	0	4
6072.2271	6072.2278	-0.0007	5	3	3	2	6	1	5	4	2	2	2	5	1	4
6072.3447	6072.3304	0.0143	5	3	3	2	6	2	7	4	2	2	2	5	2	6
6072.3447	6072.3382	0.0065	5	3	3	1	6	2	6	4	2	2	1	5	2	5
6072.3447	6072.3432	0.0015	5	5	1	2	6	2	6	4	2	2	2	5	2	5
6072.3447	6072.3483	-0.0036	5	3	3	2	5	1	6	4	2	2	2	4	1	5
6072.4398	6072.4349	0.0049	5	3	3	2	5	1	4	4	2	2	2	4	1	3
6072.4398	6072.4310	0.0088	5	3	3	2	5	2	4	4	2	2	2	4	2	3
6072.4398	6072.4270	0.0128	5	3	3	1	4	2	4	4	2	2	1	3	2	3
6072.5977	6072.5806	0.0171	5	3	3	2	5	2	6	4	2	2	2	4	2	5
6072.7351	6072.7403	-0.0052	5	1	5	2	5	2	5	4	0	4	2	4	2	4
6315.8008	6315.8148	-0.0140	5	3	2	0	5	2	5	4	2	3	0	4	2	4
6315.8008	6315.8345	-0.0337	5	3	2	1	5	2	5	4	2	3	1	4	2	4
6315.8842	6315.8872	-0.0030	5	3	2	2	6	2	7	4	2	3	2	6	2	6
6316.1541	6316.1587	-0.0046	5	3	2	1	6	2	6	4	2	3	1	5	2	5
6316.3090	6316.3238	-0.0148	5	3	2	0	5	2	6	4	2	3	0	4	2	5
6316.3090	6316.3474	-0.0384	5	3	2	1	5	2	6	4	2	3	1	4	2	5
6316.3090	6316.3246	-0.0156	5	3	2	2	6	2	6	4	2	3	2	5	2	5
6316.3090	6316.2889	0.0201	5	3	2	2	3	2	5	4	2	3	2	5	2	4

6316.4896	6316.5162	-0.0266	5	3	2	2	7	1	6	4	2	3	2	6	1	5
6316.4896	6316.4951	-0.0055	5	3	2	2	3	1	4	4	2	3	2	2	1	3
6316.5541	6316.5790	-0.0249	5	3	2	0	5	1	6	4	2	3	0	4	1	5
6316.5541	6316.5677	-0.0136	5	3	2	2	3	2	5	4	2	3	2	4	2	4
6316.5541	6316.5653	-0.0112	5	3	2	2	5	2	5	4	2	3	2	3	2	4
6316.6771	6316.6771	0.0000	5	3	2	2	7	1	8	4	2	3	2	6	1	7
6316.6771	6316.6768	0.0003	5	3	2	1	6	2	7	4	2	3	1	5	2	6
6316.6771	6316.6751	0.0020	5	3	2	2	4	1	3	4	2	3	2	3	1	2
6316.8712	6316.8794	-0.0082	5	3	2	1	6	1	7	4	2	3	1	5	1	6
6316.8712	6316.8738	-0.0026	5	3	2	2	6	1	5	4	2	3	2	5	1	4
6316.8712	6316.8641	0.0071	5	3	2	2	7	2	7	4	2	3	2	5	2	6
6316.9800	6316.9767	0.0033	5	3	2	1	4	1	5	4	2	3	1	3	1	4
6316.9800	6316.9818	-0.0018	5	3	2	2	4	2	5	4	2	3	2	2	2	4
6317.0984	6317.1067	-0.0083	5	3	2	0	5	2	7	4	2	3	0	4	2	6
6317.0984	6317.1318	-0.0334	5	3	2	1	5	2	7	4	2	3	1	4	2	6
6317.0984	6317.0956	0.0028	5	3	2	1	6	2	5	4	2	3	1	5	2	4
6317.0984	6317.0870	0.0114	5	3	2	2	5	1	4	4	2	3	2	4	1	3
6317.0984	6317.0694	0.0290	5	3	2	2	5	2	6	4	2	3	2	4	2	5
6317.0984	6317.1102	-0.0118	5	3	2	2	3	1	3	4	2	3	2	2	1	2
6317.2033	6317.2173	-0.0140	5	3	2	2	7	2	9	4	2	3	2	6	2	8
6317.2033	6317.2076	-0.0043	5	3	2	2	5	1	6	4	2	3	2	4	1	5
6317.2033	6317.2370	-0.0337	5	3	2	1	5	1	5	4	2	3	1	4	1	4
6317.2033	6317.2156	-0.0123	5	3	2	0	5	1	5	4	2	3	0	4	1	4
6317.2033	6317.2390	-0.0357	5	3	2	0	5	0	5	4	2	3	0	4	0	4
6317.2750	6317.2484	0.0266	5	3	2	2	4	2	6	4	2	3	2	3	2	5
6317.2750	6317.2622	0.0128	5	3	2	1	5	0	5	4	2	3	1	4	0	4
6317.2750	6317.2697	0.0053	5	3	2	2	7	1	6	4	2	3	2	5	1	5
6317.2750	6317.2763	-0.0013	5	3	2	2	4	2	3	4	2	3	2	3	2	2
6317.2750	6317.2549	0.0201	5	3	2	1	5	1	6	4	2	3	1	5	1	5
6317.2750	6317.2550	0.0200	5	3	2	2	3	2	1	4	2	3	2	2	2	1
6317.2750	6317.2703	0.0047	5	3	2	1	5	2	3	4	2	3	1	4	2	3
6317.3607	6317.3362	0.0245	5	3	2	2	3	2	1	4	2	3	2	2	2	0
6317.3612	6317.3327	0.0285	5	3	2	2	7	0	7	4	2	3	2	6	0	6
6317.4116	6317.3809	0.0307	5	3	2	0	5	2	3	4	2	3	0	4	2	2
6317.4125	6317.4447	-0.0322	5	3	2	2	7	2	5	4	2	3	2	6	2	4
6317.4125	6317.3921	0.0204	5	3	2	1	5	2	3	4	2	3	1	4	2	2
6317.4125	6317.4223	-0.0098	5	3	2	2	4	0	4	4	2	3	2	3	0	3
6317.4125	6317.3925	0.0200	5	3	2	2	4	1	4	4	2	3	2	3	1	3
6317.4125	6317.3994	0.0131	5	3	2	2	4	2	5	4	2	3	2	3	2	4
6317.4126	6317.4193	-0.0067	5	3	2	1	6	2	8	4	2	3	1	5	2	7
6317.5375	6317.5179	0.0196	5	3	2	1	4	2	6	4	2	3	1	3	2	5
6317.5375	6317.5161	0.0214	5	3	2	2	4	2	4	4	2	3	2	4	2	3
6317.5793	6317.5686	0.0107	5	3	2	2	6	2	8	4	2	3	2	5	2	7
6317.5793	6317.5657	0.0136	5	3	2	1	6	0	6	4	2	3	1	5	0	5
6317.5793	6317.5417	0.0376	5	3	2	1	6	1	6	4	2	3	1	5	1	5
6317.6250	6317.6279	-0.0029	5	3	2	1	4	1	4	4	2	3	1	3	1	3
6317.6250	6317.6140	0.0110	5	3	2	2	4	1	5	4	2	3	2	4	1	4
6317.7269	6317.7399	-0.0130	5	3	2	2	5	2	7	4	2	3	2	4	2	6
6317.7269	6317.7226	0.0043	5	3	2	1	6	2	4	4	2	3	1	5	2	3
6332.5750	6332.5860	-0.0110	5	4	1	1	5	2	5	4	3	2	1	4	2	4
6332.5750	6332.5831	-0.0081	5	4	1	0	5	2	5	4	3	2	0	4	2	4
6332.5919	6332.6228	-0.0309	5	4	1	2	7	2	7	4	3	2	2	6	2	6
6332.6828	6332.6886	-0.0058	5	4	1	1	5	2	6	4	3	2	1	4	2	5

6332.6828	6332.6863	-0.0035	5	4	1	0	5	2	6	4	3	2	0	4	2	5
6332.6828	6332.7002	-0.0174	5	4	1	0	5	1	4	4	3	2	0	4	1	3
6332.6828	6332.6778	0.0050	5	4	1	2	3	1	4	4	3	2	2	2	1	3
6332.6828	6332.6626	0.0202	5	4	1	2	3	1	2	4	3	2	2	2	1	1
6332.7125	6332.7294	-0.0169	5	4	1	2	7	2	8	4	3	2	2	6	2	7
6332.7125	6332.7402	-0.0277	5	4	1	2	7	1	6	4	3	2	2	6	1	5
6332.7125	6332.7175	-0.0050	5	4	1	1	5	1	6	4	3	2	1	4	1	5
6332.7125	6332.7154	-0.0029	5	4	1	0	5	1	6	4	3	2	0	4	1	5
6332.7125	6332.7032	0.0093	5	4	1	1	5	1	4	4	3	2	1	4	1	3
6332.7125	6332.7367	-0.0242	5	4	1	1	5	2	4	4	3	2	1	4	2	3
6332.7125	6332.7344	-0.0219	5	4	1	0	5	2	4	4	3	2	0	4	2	3
6332.8256	6332.8605	-0.0349	5	4	1	2	7	2	9	4	3	2	2	6	2	8
6332.8256	6332.8202	0.0054	5	4	1	0	5	2	7	4	3	2	0	4	2	6
6332.8256	6332.8228	0.0028	5	4	1	1	5	2	7	4	3	2	1	4	2	6
6332.8256	6332.8343	-0.0087	5	4	1	0	5	1	5	4	3	2	0	4	1	4
6332.8256	6332.8386	-0.0130	5	4	1	1	5	0	5	4	3	2	1	4	0	4
6332.8256	6332.8371	-0.0115	5	4	1	1	5	1	5	4	3	2	1	4	1	4
6332.8256	6332.8361	-0.0105	5	4	1	0	5	0	5	4	1	4	2	4	0	4
6332.8256	6332.8496	-0.0240	5	4	1	2	4	2	4	4	3	2	2	3	2	3
6332.8256	6332.8510	-0.0254	5	4	1	1	5	2	3	4	3	2	1	4	2	2
6332.8256	6332.8472	-0.0216	5	4	1	0	5	2	3	4	3	2	0	4	2	2
6332.8569	6332.8710	-0.0141	5	4	1	2	7	1	7	4	3	2	2	6	1	6
6332.8569	6332.8721	-0.0152	5	4	1	2	7	0	7	4	3	2	2	6	0	6
6332.8569	6332.8926	-0.0357	5	4	1	1	6	2	6	4	3	2	1	5	2	5
6332.8569	6332.8835	-0.0266	5	4	1	2	7	2	5	4	3	2	2	6	2	4
6333.4636	6333.4655	-0.0019	5	4	1	2	5	2	7	4	3	2	2	4	2	6
6333.4636	6333.4795	-0.0159	5	4	1	2	5	1	5	4	3	2	2	4	1	4
6333.4636	6333.4804	-0.0168	5	4	1	2	5	0	5	4	3	2	2	4	0	4
6333.4636	6333.4965	-0.0329	5	4	1	2	5	2	3	4	3	2	2	4	2	2
6456.7500	6456.7704	-0.0204	5	5	1	2	7	2	9	4	4	0	2	6	2	8
6456.7500	6456.7580	-0.0080	5	5	1	2	7	1	8	4	4	0	2	6	1	7
6456.7500	6456.7687	-0.0187	5	5	1	2	7	2	8	4	4	0	2	6	2	7
6456.7500	6456.7670	-0.0170	5	5	1	2	7	1	7	4	4	0	2	6	1	6
6456.7500	6456.7658	-0.0158	5	5	1	2	7	0	7	4	4	0	2	6	0	6
6456.7500	6456.7533	-0.0033	5	5	1	2	7	2	7	4	4	0	2	6	2	6
6456.7500	6456.7655	-0.0155	5	5	1	2	7	1	6	4	4	0	2	6	1	5
6456.7500	6456.7879	-0.0379	5	5	1	1	5	1	6	4	4	0	1	4	1	5
6456.7500	6456.7536	-0.0036	5	5	1	2	7	2	6	4	4	0	2	6	2	5
6456.7500	6456.7600	-0.0100	5	5	1	2	7	2	5	4	4	0	2	6	2	4
6456.7750	6456.8136	-0.0386	5	5	1	1	5	2	7	4	4	0	1	4	2	6
6456.7750	6456.8034	-0.0284	5	5	1	1	5	2	6	4	4	0	1	4	2	5
6456.7750	6456.8078	-0.0328	5	5	1	1	5	1	5	4	4	0	1	4	1	4
6456.7750	6456.8033	-0.0283	5	5	1	1	5	0	5	4	4	0	1	4	0	4
6456.7750	6456.8057	-0.0307	5	5	1	0	5	2	5	4	4	0	0	4	2	4
6456.7750	6456.7998	-0.0248	5	5	1	1	5	1	4	4	4	0	1	4	1	3
6456.8625	6456.8459	0.0166	5	5	1	0	5	2	7	4	4	0	0	4	2	6
6456.8625	6456.8542	0.0083	5	3	3	2	6	0	6	4	2	2	2	5	0	5
6456.8625	6456.8402	0.0223	5	5	1	0	5	1	5	4	4	0	0	4	1	4
6456.8625	6456.8314	0.0311	5	5	1	0	5	1	4	4	4	0	0	4	1	3
6456.8625	6456.8876	-0.0251	5	5	1	2	6	1	7	4	4	0	2	5	1	6
6456.8625	6456.8897	-0.0272	5	5	1	2	6	2	7	4	4	0	2	5	2	6
6456.8625	6456.8888	-0.0263	5	5	1	2	6	2	5	4	4	0	2	5	2	4
6456.8625	6456.8958	-0.0333	5	5	1	1	4	0	4	4	4	0	1	3	0	3

6456.8625	6456.8756	-0.0131	5	5	1	2	3	1	4	4	4	0	2	2	1	3
6456.9375	6456.9175	0.0200	5	5	1	2	6	2	8	4	4	0	2	5	2	7
6456.9375	6456.9175	0.0200	5	5	1	1	4	2	6	4	4	0	1	3	2	5
6456.9375	6456.9180	0.0195	5	5	1	2	6	1	6	4	4	0	2	5	1	5
6456.9375	6456.9177	0.0198	5	5	1	2	6	0	6	4	4	0	2	5	0	5
6456.9375	6456.9101	0.0274	5	5	1	1	4	2	5	4	4	0	1	3	2	4
6456.9375	6456.9312	0.0063	5	5	1	2	3	2	5	4	4	0	2	2	2	4
6456.9375	6456.9072	0.0303	5	5	1	1	4	1	4	4	4	0	1	3	1	3
6456.9645	6456.9734	-0.0089	5	5	1	1	6	2	8	4	4	0	1	5	2	7
6456.9645	6456.9609	0.0036	5	5	1	1	6	1	7	4	4	0	1	5	1	6
6456.9645	6456.9717	-0.0072	5	5	1	1	6	2	7	4	4	0	1	5	2	6
6456.9645	6456.9662	-0.0017	5	5	1	1	6	1	6	4	4	0	1	5	1	5
6456.9645	6456.9624	0.0021	5	5	1	1	6	0	6	4	4	0	1	5	0	5
6456.9645	6456.9568	0.0077	5	5	1	1	6	2	6	4	4	0	1	5	2	5
6456.9645	6456.9691	-0.0046	5	5	1	1	6	1	5	4	4	0	1	5	1	4
6456.9645	6456.9403	0.0242	5	5	1	1	6	2	5	4	4	0	1	5	2	4
6456.9645	6456.9523	0.0122	5	5	1	1	6	2	4	4	4	0	1	5	2	3
6457.0625	6457.0613	0.0012	5	5	1	2	4	2	5	4	4	0	2	3	2	4
6457.0625	6457.0676	-0.0051	5	5	1	2	4	1	3	4	4	0	2	3	1	2
6457.0625	6457.0732	-0.0107	5	5	1	2	4	1	5	4	4	0	2	3	1	4
6457.0625	6457.0771	-0.0146	5	5	1	2	5	2	5	4	4	0	2	4	2	4
6457.0625	6457.0453	0.0172	5	5	1	2	4	2	3	4	4	0	2	3	2	2
6457.0625	6457.0973	-0.0348	5	5	1	0	5	2	4	4	4	0	0	4	2	3
6457.1250	6457.1422	-0.0172	5	5	1	2	5	0	5	4	4	0	2	4	0	4
6457.1250	6457.1248	0.0002	5	5	1	2	5	1	4	4	4	0	2	4	1	3
6457.1250	6457.1263	-0.0013	5	5	1	2	4	1	4	4	4	0	2	3	1	3
6457.1250	6457.1184	0.0066	5	5	1	2	5	1	6	4	4	0	2	4	1	5
6457.1250	6457.1463	-0.0213	5	5	1	2	5	1	5	4	4	0	2	4	1	4
6457.1250	6457.1175	0.0075	5	5	1	2	4	0	4	4	4	0	2	3	0	3
6457.1250	6457.1495	-0.0245	5	5	1	2	5	2	7	4	4	0	2	4	2	6
6457.1250	6457.1186	0.0064	5	5	1	0	5	2	6	4	4	0	0	4	2	5
6457.1250	6457.1272	-0.0022	5	5	1	2	4	2	6	4	4	0	2	3	2	5
6457.1250	6457.1368	-0.0118	5	5	1	2	5	2	3	4	4	0	2	4	2	2
6457.8764	6457.8495	0.0269	5	5	0	2	7	1	8	4	4	1	2	6	1	7
6457.8764	6457.8567	0.0197	5	5	0	2	7	2	8	4	4	1	2	6	2	7
6457.8764	6457.8808	-0.0044	5	5	0	1	5	1	6	4	4	1	1	4	1	5
6457.8764	6457.8462	0.0302	5	5	0	2	7	2	6	4	4	1	2	6	2	5
6457.8764	6457.8638	0.0126	5	5	0	2	7	2	5	4	4	1	2	6	2	4
6457.8764	6457.8549	0.0215	5	5	0	2	7	1	6	4	4	1	2	6	1	5
6457.8764	6457.8680	0.0084	5	5	0	2	7	0	7	4	4	1	2	6	0	6
6457.8764	6457.8691	0.0073	5	5	0	2	7	1	7	4	4	1	2	6	1	6
6457.8764	6457.8712	0.0052	5	5	0	2	7	2	9	4	4	1	2	6	2	8
6457.9726	6457.9468	0.0258	5	5	0	0	5	2	7	4	4	1	0	4	2	6
6457.9726	6457.9430	0.0296	5	5	0	0	5	1	5	4	4	1	0	4	1	4
6457.9726	6457.9391	0.0335	5	5	0	0	5	0	5	4	4	1	0	4	0	4
6458.0063	6457.9733	0.0330	5	5	0	2	6	1	7	4	4	1	2	5	1	6
6458.0063	6457.9729	0.0334	5	5	0	2	6	2	7	4	4	1	2	5	2	6
6458.0063	6457.9707	0.0356	5	5	0	2	3	1	4	4	4	1	2	2	1	3
6458.0063	6457.9762	0.0301	5	5	0	2	6	1	5	4	4	1	2	5	1	4
6458.0063	6457.9849	0.0214	5	5	0	2	3	2	4	4	4	1	2	2	2	3
6458.0063	6457.9753	0.0310	5	5	0	2	6	2	5	4	4	1	2	5	2	4
6458.0063	6457.9771	0.0292	5	5	0	1	4	1	5	4	4	1	1	3	1	4
6458.0375	6458.0115	0.0260	5	5	0	2	6	0	6	4	4	1	2	5	0	5

6458.0375	6458.0115	0.0260	5	5	0	1	4	2	6	4	4	1	1	3	2	5
6458.0375	6458.0103	0.0272	5	5	0	2	6	2	8	4	4	1	2	5	2	7
6458.0375	6458.0036	0.0339	5	5	0	1	4	1	4	4	4	1	1	3	1	3
6458.0375	6458.0118	0.0257	5	5	0	2	6	1	6	4	4	1	2	5	1	5
6458.0665	6458.0696	-0.0031	5	5	0	1	6	2	8	4	4	1	1	5	2	7
6458.0665	6458.0643	0.0022	5	5	0	1	6	1	6	4	4	1	1	5	1	5
6458.0665	6458.0609	0.0056	5	5	0	1	6	0	6	4	4	1	1	5	0	5
6458.0665	6458.0321	0.0344	5	5	0	1	6	2	6	4	4	1	1	5	2	5
6458.0665	6458.0541	0.0124	5	5	0	1	6	1	5	4	4	1	1	5	1	4
6458.0665	6458.0478	0.0187	5	5	0	1	6	1	7	4	4	1	1	5	1	6
6458.0665	6458.0555	0.0110	5	5	0	1	6	2	7	4	4	1	1	5	2	6
6458.0665	6458.0532	0.0133	5	5	0	1	6	2	4	4	4	1	1	5	2	3
6458.0665	6458.0309	0.0356	5	5	0	1	6	2	5	4	4	1	1	5	2	4
6458.1510	6458.1570	-0.0060	5	5	0	2	4	1	3	4	4	1	2	3	1	2
6458.1510	6458.1836	-0.0326	5	5	0	2	5	2	4	4	4	1	2	4	2	3
6458.1510	6458.1415	0.0095	5	5	0	2	4	2	3	4	4	1	2	3	2	2
6458.1510	6458.1513	-0.0003	5	5	0	2	5	2	5	4	4	1	2	4	2	4
6458.1510	6458.1636	-0.0126	5	5	0	2	4	1	5	4	4	1	2	3	1	4
6458.1510	6458.1499	0.0011	5	5	0	2	4	2	5	4	4	1	2	3	2	4
6458.2367	6458.1982	0.0385	5	5	0	2	5	2	6	4	4	1	2	4	2	5
6458.2367	6458.2214	0.0153	5	5	0	2	4	2	6	4	4	1	2	3	2	5
6458.2367	6458.2296	0.0071	5	5	0	2	5	2	3	4	4	1	2	4	2	2
6458.2367	6458.2381	-0.0014	5	5	0	2	5	2	7	4	4	1	2	4	2	6
6458.2367	6458.2001	0.0366	5	5	0	2	5	1	6	4	4	1	2	4	1	5
6458.2367	6458.2156	0.0211	5	5	0	2	4	0	4	4	4	1	2	3	0	3
6458.2367	6458.2366	0.0001	5	5	0	2	5	1	5	4	4	1	2	4	1	4
6458.2367	6458.2331	0.0036	5	5	0	2	5	0	5	4	4	1	2	4	0	4
6458.2367	6458.2044	0.0323	5	5	0	2	5	1	4	4	4	1	2	4	1	3
6458.2367	6458.2232	0.0135	5	5	0	2	4	1	4	4	4	1	2	3	1	3
6782.0078	6782.0059	0.0019	6	0	6	2	5	2	7	5	1	5	2	4	2	6
6782.0078	6782.0111	-0.0033	6	0	6	2	5	1	5	5	1	5	2	4	1	4
6782.0678	6782.0607	0.0071	6	2	4	2	5	2	6	5	3	3	2	4	2	5
6782.0678	6782.0340	0.0338	6	0	6	2	5	0	5	5	1	5	2	4	0	4
6782.0678	6782.0706	-0.0028	6	0	6	2	6	2	4	5	1	5	0	5	2	3
6782.0678	6782.0690	-0.0012	6	0	6	2	4	1	4	5	1	5	2	3	1	3
6782.0678	6782.0510	0.0168	6	0	6	2	6	2	8	5	1	5	2	5	2	7
6782.0678	6782.0561	0.0117	6	0	6	2	6	1	6	5	1	5	2	5	1	5
6782.0678	6782.0633	0.0045	6	0	6	2	6	0	6	5	1	5	2	5	0	5
6782.0678	6782.0457	0.0221	6	0	6	2	5	2	3	5	1	5	2	4	2	2
6782.1500	6782.1375	0.0125	6	0	6	2	7	1	7	5	1	5	2	6	1	6
6782.1500	6782.1430	0.0070	6	0	6	1	5	2	7	5	1	5	1	4	2	6
6782.1500	6782.1344	0.0156	6	0	6	2	7	2	7	5	1	5	2	5	2	6
6782.1500	6782.1472	0.0028	6	0	6	2	7	2	9	5	1	5	2	6	2	8
6782.1500	6782.1334	0.0166	6	0	6	0	6	2	8	5	1	5	0	5	2	7
6782.1500	6782.1515	-0.0015	6	0	6	1	6	2	8	5	1	5	1	5	2	7
6782.1500	6782.1222	0.0278	6	0	6	1	7	1	7	5	1	5	1	6	1	6
6782.1500	6782.1265	0.0235	6	0	6	1	7	0	7	5	1	5	1	6	0	6
6782.1500	6782.1456	0.0044	6	0	6	2	5	2	6	5	1	5	2	3	2	5
6782.1844	6782.1747	0.0097	6	0	6	2	6	2	7	5	1	5	2	6	0	6
6782.1844	6782.1625	0.0219	6	0	6	2	6	1	7	5	1	5	2	5	1	6
6782.1844	6782.1907	-0.0063	6	0	6	1	7	1	6	5	1	5	1	6	1	5
6782.1844	6782.2002	-0.0158	6	0	6	2	8	2	6	5	1	5	2	7	2	5
6782.1844	6782.1968	-0.0124	6	0	6	2	8	2	10	5	1	5	2	7	2	9

6782.1844	6782.1922	-0.0078	6	0	6	1	7	1	8	5	1	5	1	6	1	7
6782.1844	6782.1969	-0.0125	6	0	6	2	8	0	8	5	1	5	2	7	0	7
6782.1844	6782.1973	-0.0129	6	0	6	2	8	1	8	5	1	5	2	7	1	7
6782.1844	6782.1909	-0.0065	6	0	6	1	7	2	8	5	1	5	1	6	2	7
6782.2125	6782.2422	-0.0297	6	0	6	0	6	1	7	5	1	5	0	5	1	6
6782.2125	6782.2177	-0.0052	6	0	6	0	6	2	7	5	1	5	0	5	2	6
6782.2125	6782.2308	-0.0183	6	0	6	1	6	2	7	5	1	5	1	5	2	6
6782.2125	6782.2523	-0.0398	6	0	6	2	7	1	8	5	1	5	2	6	1	7
6782.2853	6782.3055	-0.0202	6	0	6	2	4	2	4	5	1	5	2	3	2	3
6782.2853	6782.3033	-0.0180	6	0	6	1	5	2	4	5	1	5	1	4	2	3
6782.2853	6782.3222	-0.0369	6	0	6	0	6	2	6	5	1	5	0	5	2	5
6782.2853	6782.3120	-0.0267	6	0	6	2	7	2	6	5	1	5	2	6	2	5
6782.2853	6782.3150	-0.0297	6	0	6	0	6	2	5	5	1	5	0	5	2	4
6782.2853	6782.2928	-0.0075	6	0	6	2	4	2	3	5	1	5	2	3	2	2
6783.4236	6783.4162	0.0074	6	1	6	2	5	2	7	5	0	5	2	4	2	6
6783.4236	6783.4185	0.0051	6	1	6	2	5	1	5	5	0	5	2	4	1	4
6783.4236	6783.4393	-0.0157	6	1	6	2	5	0	5	5	0	5	2	4	0	4
6783.4236	6783.4474	-0.0238	6	1	6	2	5	2	3	5	0	5	2	4	2	2
6783.4815	6783.4729	0.0086	6	1	6	2	6	1	6	5	0	5	2	5	1	5
6783.4815	6783.4692	0.0123	6	1	6	2	6	2	8	5	0	5	2	5	2	7
6783.4815	6783.4799	0.0016	6	1	6	2	6	0	6	5	0	5	2	5	0	5
6783.4815	6783.4717	0.0098	6	1	6	2	4	1	4	5	0	5	2	3	1	3
6783.4815	6783.4671	0.0144	6	1	6	2	5	2	6	5	0	5	2	4	2	5
6783.4815	6783.4857	-0.0042	6	1	6	2	6	2	4	5	0	5	2	5	2	3
6783.5603	6783.5553	0.0050	6	1	6	1	6	2	8	5	0	5	1	5	2	7
6783.5603	6783.5518	0.0085	6	1	6	1	5	2	7	5	0	5	1	4	2	6
6783.5603	6783.5500	0.0103	6	1	6	2	7	2	7	5	0	5	2	5	2	6
6783.5603	6783.5524	0.0079	6	1	6	2	7	1	7	5	0	5	2	6	1	6
6783.5603	6783.5578	0.0025	6	1	6	2	7	2	9	5	0	5	2	6	2	8
6783.5603	6783.5355	0.0248	6	1	6	0	6	2	8	5	0	5	0	5	2	7
6783.5603	6783.5443	0.0160	6	1	6	0	6	1	6	5	0	5	0	5	1	5
6783.5603	6783.5282	0.0321	6	1	6	1	7	1	7	5	0	5	1	6	1	6
6783.5603	6783.5327	0.0276	6	1	6	1	7	0	7	5	0	5	1	6	0	6
6783.5603	6783.5475	0.0128	6	1	6	2	4	2	6	5	0	5	2	3	2	5
6783.5937	6783.5824	0.0113	6	1	6	2	6	1	7	5	0	5	2	5	1	6
6783.5937	6783.6023	-0.0086	6	1	6	1	7	2	8	5	0	5	1	6	2	7
6783.5937	6783.5979	-0.0042	6	1	6	2	8	1	8	5	0	5	2	7	1	7
6783.5937	6783.5962	-0.0025	6	1	6	2	8	2	10	5	0	5	2	7	2	9
6783.5937	6783.5845	0.0092	6	1	6	0	6	0	6	5	0	5	0	5	0	5
6783.5937	6783.6071	-0.0134	6	1	6	1	7	1	8	5	0	5	1	6	1	7
6783.5937	6783.5980	-0.0043	6	1	6	2	8	0	8	5	0	5	2	7	0	7
6783.5937	6783.5914	0.0023	6	1	6	2	6	2	7	5	0	5	2	6	0	6
6783.5937	6783.6035	-0.0098	6	1	6	1	7	1	6	5	0	5	1	6	1	5
6783.5937	6783.6026	-0.0089	6	1	6	2	8	2	6	5	0	5	2	7	2	5
6783.6268	6783.6624	-0.0356	6	1	6	1	6	1	7	5	0	5	1	5	1	6
6783.6268	6783.6474	-0.0206	6	1	6	0	6	1	7	5	0	5	0	5	1	6
6783.6268	6783.6413	-0.0145	6	1	6	1	6	2	7	5	0	5	1	5	2	6
6783.7020	6783.7244	-0.0224	6	1	6	0	6	2	5	5	0	5	0	5	2	4
6783.7020	6783.7187	-0.0167	6	1	6	2	4	2	4	5	0	5	2	3	2	3
6783.7020	6783.7141	-0.0121	6	1	6	1	5	2	4	5	0	5	1	4	2	3
6783.7020	6783.7371	-0.0351	6	1	6	0	6	2	6	5	0	5	0	5	2	5
6783.7020	6783.7301	-0.0281	6	1	6	2	7	2	6	5	0	5	2	6	2	5
6783.7375	6783.7647	-0.0272	6	1	6	2	8	2	8	5	0	5	2	7	2	7

6783.7375	6783.7765	-0.0390	6	1	6	1	5	2	5	5	0	5	1	4	2	4
6873.4658	6873.4606	0.0052	6	0	6	2	6	2	6	5	1	5	2	5	2	5
6873.5976	6873.6242	-0.0266	6	2	4	2	6	1	7	5	3	3	2	5	1	6
6873.5976	6873.6018	-0.0042	6	2	4	2	6	2	7	5	3	3	2	5	2	6
6873.5976	6873.6036	-0.0060	6	2	4	2	6	1	5	5	3	3	2	5	1	4
6873.6750	6873.6772	-0.0022	6	2	4	2	6	2	5	5	3	3	2	5	2	4
6873.8029	6873.8000	0.0029	6	2	4	2	6	2	8	5	3	3	2	6	2	7
6873.8029	6873.8134	-0.0105	6	2	4	2	6	0	6	5	3	3	2	5	0	5
6873.8029	6873.8069	-0.0040	6	2	4	2	6	1	6	5	3	3	2	6	1	5
6873.8029	6873.8273	-0.0244	6	2	4	2	6	2	4	5	1	5	2	5	2	3
6874.0142	6874.0133	0.0009	6	4	2	2	7	2	7	5	3	3	2	6	2	6
6874.0955	6874.0865	0.0090	6	2	4	1	5	2	5	5	3	3	1	4	2	4
6874.0955	6874.0874	0.0081	6	2	4	2	7	2	8	5	3	3	2	7	2	7
6874.0955	6874.0850	0.0105	6	4	2	2	7	2	7	5	3	3	2	5	2	6
6874.1852	6874.1876	-0.0024	6	2	4	2	7	1	8	5	3	3	2	6	1	7
6874.1852	6874.1911	-0.0059	6	2	4	1	7	2	7	5	3	3	1	6	2	6
6874.1852	6874.2112	-0.0260	6	2	4	2	7	2	8	5	3	3	2	6	2	7
6874.1852	6874.2225	-0.0373	6	2	4	2	7	2	6	5	1	5	0	5	0	5
6874.1852	6874.2098	-0.0246	6	2	4	2	7	1	6	5	3	3	2	6	1	5
6874.3253	6874.3332	-0.0079	6	2	4	1	7	2	8	5	3	3	1	6	2	7
6874.3253	6874.3390	-0.0137	6	2	4	2	7	2	9	5	3	3	2	6	2	8
6874.3253	6874.3540	-0.0287	6	2	4	2	7	1	7	5	3	3	2	6	1	6
6874.3253	6874.3595	-0.0342	6	0	6	2	7	0	7	5	5	1	2	6	2	6
6874.3253	6874.3467	-0.0214	6	2	4	1	7	1	6	5	3	3	1	6	1	5
6874.3253	6874.3356	-0.0103	6	2	4	2	5	2	5	5	3	3	2	4	2	4
6874.3253	6874.3520	-0.0267	6	2	4	1	7	1	6	5	3	3	1	4	1	5
6874.3253	6874.3421	-0.0168	6	2	4	1	7	2	6	5	3	3	1	6	2	5
6874.4241	6874.4208	0.0033	6	2	4	1	5	2	7	5	3	3	1	5	2	6
6874.4241	6874.4302	-0.0061	6	2	4	1	5	0	5	5	3	3	1	4	0	4
6874.4241	6874.4401	-0.0160	6	2	4	1	5	1	5	5	3	3	1	4	1	4
6874.4241	6874.4286	-0.0045	6	2	4	1	7	2	6	5	3	3	1	5	2	5
6874.4241	6874.4490	-0.0249	6	2	4	1	5	2	3	5	3	3	1	4	2	2
6874.5107	6874.5121	-0.0014	6	2	4	1	7	2	9	5	3	3	1	6	2	8
6874.5107	6874.5080	0.0027	6	2	4	2	5	1	6	5	3	3	2	4	1	5
6874.5107	6874.4945	0.0162	6	2	4	2	5	1	4	5	3	3	2	4	1	3
6874.5107	6874.5051	0.0056	6	2	4	1	7	0	7	5	3	3	1	6	0	6
6874.5250	6874.5442	-0.0192	6	2	4	1	7	1	7	5	3	3	1	6	1	6
6874.5250	6874.5616	-0.0366	6	2	4	1	7	2	5	5	3	3	1	6	2	4
6874.5250	6874.5487	-0.0237	6	2	4	1	7	0	7	5	3	3	1	4	2	6
6874.9000	6874.8955	0.0045	6	2	4	2	8	2	9	5	3	3	2	7	2	8
6874.9000	6874.9182	-0.0182	6	2	4	2	8	1	7	5	3	3	2	7	1	6
6874.9000	6874.9142	-0.0142	6	2	4	1	6	2	6	5	3	3	1	5	2	5
6874.9585	6874.9479	0.0106	6	2	4	2	8	1	9	5	3	3	2	7	1	8
6874.9585	6874.9627	-0.0042	6	2	4	2	8	2	7	5	3	3	2	7	2	6
6874.9585	6874.9647	-0.0062	6	2	4	1	6	2	7	5	3	3	1	6	0	6
6874.9585	6874.9602	-0.0017	6	2	4	2	8	2	7	5	5	1	2	5	2	6
6875.0031	6875.0011	0.0020	6	2	4	1	6	1	5	5	3	3	1	5	1	4
6875.0031	6875.0220	-0.0189	6	2	4	0	6	1	5	5	3	3	2	3	1	4
6875.0031	6874.9881	0.0150	6	2	4	2	8	2	9	5	3	3	2	6	2	8
6875.0031	6875.0082	-0.0051	6	2	4	1	6	2	7	5	3	3	1	4	2	6
6875.0031	6874.9894	0.0137	6	2	4	2	8	1	7	5	3	3	2	6	1	6
6875.0031	6874.9863	0.0168	6	2	4	0	6	2	7	5	3	3	2	7	2	6
6875.1187	6875.0912	0.0275	6	2	4	2	8	2	10	5	3	3	2	7	2	9

6875.1187	6875.1067	0.0120	6	2	4	2	8	1	8	5	3	3	2	7	1	7
6875.1187	6875.1109	0.0078	6	2	4	2	8	0	8	5	3	3	2	7	0	7
6875.1187	6875.1280	-0.0093	6	2	4	2	8	2	6	5	3	3	2	7	2	5
6875.1187	6875.1020	0.0167	6	2	4	2	8	0	8	5	3	3	0	5	2	7
6875.2103	6875.1786	0.0317	6	2	4	1	6	2	8	5	3	3	1	5	2	7
6875.2103	6875.1975	0.0128	6	2	4	1	6	1	6	5	3	3	1	5	1	5
6875.2103	6875.2001	0.0102	6	2	4	1	6	0	6	5	3	3	1	5	0	5
6875.2103	6875.2135	-0.0032	6	2	4	0	6	1	6	5	3	3	0	5	1	5
6875.2103	6875.1917	0.0186	6	2	4	0	6	2	8	5	3	3	0	5	2	7
6875.2103	6875.2216	-0.0113	6	2	4	1	6	2	4	5	3	3	1	5	2	3
6875.2103	6875.2100	0.0003	6	2	4	0	6	0	6	5	3	3	0	5	0	5
6875.2103	6875.2007	0.0096	6	2	4	0	6	2	8	5	3	3	2	7	0	7
6875.3308	6875.3191	0.0117	6	2	4	2	4	1	4	5	3	3	2	3	1	3
6875.3308	6875.2978	0.0330	6	2	4	2	4	2	6	5	3	3	2	3	2	5
6875.3308	6875.3201	0.0107	6	2	4	2	4	0	4	5	5	1	2	3	2	3
6875.3308	6875.3334	-0.0026	6	2	4	2	4	0	4	5	3	3	2	3	2	3
6886.3822	6886.3769	0.0053	6	1	5	2	6	2	8	5	2	4	2	5	2	7
6886.3822	6886.3702	0.0120	6	1	5	2	6	1	6	5	2	4	2	5	1	5
6886.3822	6886.3707	0.0115	6	1	5	2	6	0	6	5	2	4	2	5	0	5
6886.3822	6886.3638	0.0184	6	1	5	2	6	2	4	5	2	4	2	5	2	3
6886.4681	6886.4539	0.0142	6	1	5	2	6	1	7	5	2	4	2	5	1	6
6886.4681	6886.4669	0.0012	6	1	5	2	6	2	7	5	2	4	2	5	2	6
6886.4681	6886.4603	0.0078	6	1	5	2	6	1	5	5	2	4	2	5	1	4
6886.4681	6886.4477	0.0204	6	1	5	2	6	2	5	5	2	4	2	5	2	4
6886.6004	6886.5940	0.0064	6	1	5	1	7	2	9	5	2	4	1	6	2	8
6886.6004	6886.5722	0.0282	6	1	5	2	7	2	9	5	2	4	2	6	2	8
6886.6004	6886.5885	0.0119	6	1	5	1	7	1	7	5	2	4	1	6	1	6
6886.6004	6886.5859	0.0145	6	1	5	1	5	2	7	5	2	4	1	4	2	6
6886.6004	6886.5652	0.0352	6	1	5	2	7	0	7	5	2	4	2	6	0	6
6886.6004	6886.5660	0.0344	6	1	5	2	7	1	7	5	2	4	2	6	1	6
6886.6004	6886.5923	0.0081	6	1	5	2	5	2	7	5	2	4	2	4	2	6
6886.6004	6886.5835	0.0169	6	1	5	1	7	2	5	5	2	4	1	6	2	4
6886.6004	6886.5803	0.0201	6	1	5	1	5	1	5	5	2	4	1	4	1	4
6886.6004	6886.5889	0.0115	6	1	5	1	7	0	7	5	2	4	1	6	0	6
6886.6822	6886.6493	0.0329	6	1	5	2	7	1	8	5	2	4	2	6	1	7
6886.6822	6886.6618	0.0204	6	1	5	2	7	2	8	5	2	4	2	6	2	7
6886.6822	6886.6757	0.0065	6	1	5	1	7	1	6	5	2	4	1	6	1	5
6886.6822	6886.6752	0.0070	6	1	5	1	5	2	6	5	2	4	1	4	2	5
6886.6822	6886.6811	0.0011	6	1	5	1	7	2	8	5	2	4	1	6	2	7
6886.6822	6886.6719	0.0103	6	1	5	2	5	1	6	5	2	4	2	4	1	5
6886.6822	6886.6560	0.0262	6	1	5	2	7	1	6	5	2	4	2	6	1	5
6886.6822	6886.6702	0.0120	6	1	5	1	7	1	8	5	2	4	1	6	1	7
6886.6822	6886.6636	0.0186	6	1	5	1	5	1	6	5	2	4	1	4	1	5
6886.6822	6886.6661	0.0161	6	1	5	1	7	2	6	5	2	4	1	6	2	5
6886.7970	6886.7863	0.0107	6	1	5	2	8	2	10	5	2	4	2	7	2	9
6886.7970	6886.7810	0.0160	6	1	5	2	8	0	8	5	2	4	2	7	0	7
6886.7970	6886.7812	0.0158	6	1	5	2	8	1	8	5	2	4	2	7	1	7
6886.7970	6886.7924	0.0046	6	1	5	0	6	2	8	5	2	4	0	5	2	7
6886.7970	6886.7863	0.0107	6	1	5	0	6	0	6	5	2	4	0	5	0	5
6886.7970	6886.7759	0.0211	6	1	5	2	8	2	6	5	2	4	2	7	2	5
6886.7970	6886.7570	0.0400	6	1	5	1	7	2	7	5	2	4	1	6	2	6
6886.7970	6886.7900	0.0070	6	1	5	1	6	0	6	5	2	4	1	5	0	5
6886.7970	6886.7862	0.0108	6	1	5	0	6	1	6	5	2	4	0	5	1	5

6886.7970	6886.7960	0.0010	6	1	5	1	6	2	8	5	2	4	1	5	2	7
6886.8842	6886.8682	0.0160	6	1	5	2	8	1	7	5	2	4	2	7	1	6
6886.8842	6886.8735	0.0107	6	1	5	1	6	1	7	5	2	4	1	5	1	6
6886.8842	6886.8699	0.0143	6	1	5	0	6	1	7	5	2	4	0	5	1	6
6886.8842	6886.8786	0.0056	6	1	5	1	6	1	5	5	2	4	1	5	1	4
6886.8842	6886.8753	0.0089	6	1	5	0	6	1	5	5	2	4	0	5	1	4
6886.8842	6886.8820	0.0022	6	1	5	0	6	2	7	5	2	4	0	5	2	6
6886.8842	6886.8855	-0.0013	6	1	5	1	6	2	7	5	2	4	1	5	2	6
6886.8842	6886.8572	0.0270	6	1	5	2	8	2	7	5	2	4	2	7	2	6
6886.8842	6886.8628	0.0214	6	1	5	2	8	1	9	5	2	4	2	7	1	8
6886.8842	6886.8737	0.0105	6	1	5	2	8	2	9	5	2	4	2	7	2	8
6886.9731	6886.9501	0.0230	6	1	5	2	8	2	8	5	2	4	2	7	2	7
6886.9731	6886.9628	0.0103	6	1	5	1	6	2	6	5	2	4	1	5	2	5
6886.9731	6886.9596	0.0135	6	1	5	0	6	2	6	5	2	4	0	5	2	5
6886.9731	6886.9728	0.0003	6	3	3	2	4	0	4	5	2	4	2	3	2	3
6926.2675	6926.2940	-0.0265	6	2	5	2	8	2	8	5	1	4	2	7	2	7
6926.2675	6926.2669	0.0006	6	2	5	1	7	2	7	5	1	4	1	6	2	6
6926.2675	6926.2608	0.0067	6	2	5	2	7	2	7	5	1	4	2	6	2	6
6926.2675	6926.2699	-0.0024	6	2	5	1	6	2	6	5	1	4	1	5	2	5
6926.2675	6926.2590	0.0085	6	2	5	0	6	2	6	5	1	4	0	5	2	5
6926.2675	6926.2782	-0.0107	6	2	5	1	5	2	5	5	1	4	1	4	2	4
7153.0250	7153.0237	0.0013	6	3	4	2	4	2	2	5	2	3	2	3	2	1
7153.1197	7153.1394	-0.0197	6	3	4	1	6	2	4	5	2	3	1	5	2	3
7153.1197	7153.1535	-0.0338	6	3	4	2	4	1	4	5	2	3	2	3	1	3
7153.1197	7153.1300	-0.0103	6	3	4	0	6	2	4	5	2	3	0	5	2	3
7153.1197	7153.1206	-0.0009	6	3	4	2	4	0	4	5	2	3	2	3	0	3
7153.1197	7153.1526	-0.0329	6	3	4	0	6	0	6	5	2	3	2	3	2	5
7153.2221	7153.2316	-0.0095	6	3	4	1	6	1	6	5	2	3	1	5	1	5
7153.2221	7153.2164	0.0057	6	3	4	0	6	1	6	5	2	3	0	5	1	5
7153.2221	7153.2163	0.0058	6	3	4	1	6	0	6	5	2	3	1	5	0	5
7153.2221	7153.2112	0.0109	6	3	4	2	8	2	6	5	2	3	2	7	2	5
7153.2221	7153.2207	0.0014	6	3	4	2	4	2	6	5	2	3	2	3	2	5
7153.2221	7153.2061	0.0160	6	3	4	0	6	0	6	5	2	3	0	5	0	5
7153.2814	7153.2835	-0.0021	6	3	4	2	8	1	8	5	2	3	2	7	1	7
7153.2814	7153.2961	-0.0147	6	3	4	1	6	2	8	5	2	3	1	5	2	7
7153.2814	7153.2758	0.0056	6	3	4	2	8	0	8	5	2	3	2	7	0	7
7153.2814	7153.2775	0.0039	6	3	4	0	6	2	8	5	2	3	0	5	2	7
7153.3326	7153.3403	-0.0077	6	3	4	2	8	2	10	5	2	3	2	7	2	9
7153.4625	7153.4545	0.0080	6	3	4	1	7	2	5	5	2	3	1	6	2	4
7153.4625	7153.4584	0.0041	6	3	4	2	4	1	5	5	2	3	2	3	1	4
7153.4625	7153.4438	0.0187	6	3	4	1	6	2	5	5	2	3	1	5	2	4
7153.4625	7153.4339	0.0286	6	3	4	0	6	2	5	5	2	3	0	5	2	4
7153.4625	7153.4625	-0.0000	6	3	4	1	5	2	3	5	2	3	1	4	2	2
7153.4625	7153.4541	0.0084	6	3	4	2	5	2	3	5	2	3	2	4	2	2
7153.5375	7153.5202	0.0173	6	3	4	1	7	1	7	5	2	3	1	6	1	6
7153.5375	7153.5211	0.0164	6	3	4	1	7	0	7	5	2	3	1	6	0	6
7153.5625	7153.5605	0.0020	6	3	4	1	7	2	9	5	2	3	1	6	2	8
7153.5625	7153.5825	-0.0200	6	3	4	0	6	1	7	5	2	3	0	5	1	6
7153.5625	7153.5947	-0.0322	6	3	4	1	6	1	7	5	2	3	1	5	1	6
7153.5625	7153.5677	-0.0052	6	3	4	2	8	2	7	5	2	3	2	7	2	6
7153.5625	7153.5619	0.0006	6	3	4	2	5	2	7	5	2	3	2	4	2	6
7153.5625	7153.5754	-0.0129	6	3	4	1	5	0	5	5	2	3	1	4	0	4
7153.5625	7153.5404	0.0221	6	3	4	2	5	1	5	5	2	3	2	4	1	4

7153.5625	7153.5580	0.0045	6	3	4	2	5	0	5	5	2	3	2	4	0	4
7153.5625	7153.5531	0.0094	6	3	4	2	4	2	5	5	2	3	2	3	2	4
7153.6250	7153.6398	-0.0148	6	3	4	0	6	2	7	5	2	3	0	5	2	6
7153.6250	7153.6127	0.0123	6	3	4	1	5	1	5	5	2	3	1	4	1	4
7153.6250	7153.6082	0.0168	6	3	4	2	7	2	5	5	2	3	2	6	2	4
7153.6250	7153.6397	-0.0147	6	3	4	0	6	1	5	5	2	3	0	5	1	4
7153.6250	7153.6210	0.0040	6	3	4	2	4	1	3	5	2	3	2	3	1	2
7153.7105	7153.6813	0.0292	6	3	4	2	8	1	9	5	2	3	2	7	1	8
7153.7105	7153.7109	-0.0004	6	3	4	2	8	1	7	5	2	3	2	7	1	6
7153.7105	7153.7026	0.0079	6	3	4	1	5	2	7	5	2	3	1	4	2	6
7153.7105	7153.6995	0.0110	6	3	4	2	7	1	7	5	2	3	2	6	1	6
7153.7105	7153.6896	0.0209	6	3	4	2	7	0	7	5	2	3	2	6	0	6
7153.8419	7153.8547	-0.0128	6	3	4	2	5	1	4	5	2	3	2	4	1	3
7153.8419	7153.8302	0.0117	6	3	4	1	5	2	4	5	2	3	1	4	2	3
7153.8419	7153.8386	0.0033	6	3	4	2	6	2	4	5	2	3	2	5	2	3
7153.8419	7153.8329	0.0090	6	3	4	2	5	2	4	5	2	3	2	4	2	3
7153.9122	7153.9017	0.0105	6	3	4	1	7	1	8	5	2	3	1	6	1	7
7153.9122	7153.8920	0.0202	6	3	4	1	7	2	6	5	2	3	1	6	2	5
7153.9122	7153.8754	0.0368	6	3	4	2	5	2	6	5	2	3	2	4	2	5
7153.9229	7153.9301	-0.0072	6	3	4	2	6	0	6	5	2	3	2	5	0	5
7153.9229	7153.9378	-0.0149	6	3	4	0	6	2	6	5	2	3	0	5	2	5
7153.9229	7153.9332	-0.0103	6	3	4	2	7	2	6	5	2	3	2	6	2	5
7153.9229	7153.9361	-0.0132	6	3	4	1	6	2	6	5	2	3	1	5	2	5
7153.9229	7153.9487	-0.0258	6	3	4	2	6	1	6	5	2	3	2	5	1	5
7153.9391	7153.9759	-0.0368	6	3	4	1	7	1	6	5	2	3	1	6	1	5
7153.9391	7153.9674	-0.0283	6	3	4	1	5	1	6	5	2	3	1	4	1	5
7154.1000	7154.0861	0.0139	6	3	4	2	8	2	8	5	2	3	2	7	2	7
7154.1000	7154.0600	0.0400	6	3	4	1	5	1	4	5	2	3	1	4	1	3
7154.1375	7154.1404	-0.0029	6	3	4	1	5	2	6	5	2	3	1	4	2	5
7154.2095	7154.2095	-0.0000	6	3	4	2	6	1	7	5	2	3	2	5	1	6
7154.2095	7154.2165	-0.0070	6	3	4	2	6	2	5	5	2	3	2	5	2	4
7154.2800	7154.2820	-0.0020	6	3	4	2	6	1	5	5	2	3	2	5	1	4
7154.3562	7154.3550	0.0012	6	3	4	1	7	2	7	5	2	3	1	6	2	6
7154.3562	7154.3593	-0.0031	6	1	6	2	7	0	7	5	2	3	2	6	2	6
7154.3562	7154.3645	-0.0083	6	3	4	2	7	2	8	5	2	3	2	5	2	7
7154.4089	7154.3830	0.0259	6	3	4	2	6	2	7	5	2	3	2	5	2	6
7154.4089	7154.4134	-0.0045	6	3	4	1	5	2	5	5	2	3	1	4	2	4
7154.6186	7154.6231	-0.0045	6	3	4	2	6	2	6	5	2	3	2	5	2	5
7154.6186	7154.6474	-0.0288	6	3	4	2	5	2	7	5	2	3	2	5	2	7
7539.3375	7539.3422	-0.0047	6	4	2	1	6	2	6	5	3	3	1	5	2	5
7539.3375	7539.3316	0.0059	6	4	2	2	8	2	8	5	3	3	2	7	2	7
7539.3375	7539.3472	-0.0097	6	4	2	0	6	2	6	5	1	5	0	5	0	5
7539.3375	7539.3627	-0.0252	6	4	2	2	4	2	4	5	1	5	2	5	2	3
7539.4000	7539.3747	0.0253	6	4	2	2	8	2	8	5	3	3	2	5	2	7
7539.4000	7539.4090	-0.0090	6	4	2	0	6	2	6	5	3	3	2	5	0	5
7539.4000	7539.4098	-0.0098	6	4	2	1	6	2	6	5	3	3	1	4	2	5
7539.4000	7539.4056	-0.0056	6	4	2	2	5	2	5	5	3	3	2	4	2	4
7539.5557	7539.5509	0.0048	6	4	2	1	7	2	7	5	3	3	1	6	2	6
7539.5557	7539.5888	-0.0331	6	4	2	1	5	2	5	5	3	3	1	4	2	4
7539.5557	7539.5540	0.0017	6	4	2	2	8	2	9	5	3	3	2	7	2	8
7539.5557	7539.5354	0.0203	6	4	2	2	4	1	3	5	3	3	2	3	1	2
7539.5557	7539.5715	-0.0158	6	6	0	2	7	2	7	5	3	3	2	6	2	6
7539.5557	7539.5563	-0.0006	6	4	2	2	5	2	5	5	3	3	2	5	2	4

7539.5557	7539.5574	-0.0017	6	4	2	1	6	1	5	5	3	3	1	5	1	4
7539.5557	7539.5493	0.0064	6	4	2	2	4	2	5	5	3	3	2	3	2	4
7539.5557	7539.5509	0.0048	6	4	2	0	6	2	7	5	3	3	2	4	2	6
7539.5557	7539.5406	0.0151	6	4	2	2	4	1	5	5	3	3	2	4	1	4
7539.6250	7539.6074	0.0176	6	4	2	0	6	1	7	5	3	3	0	5	1	6
7539.6250	7539.6155	0.0095	6	4	2	1	6	2	7	5	3	3	1	5	2	6
7539.6250	7539.5989	0.0261	6	4	2	2	8	1	7	5	3	3	2	7	1	6
7539.6250	7539.5962	0.0288	6	4	2	0	6	1	5	5	3	3	0	5	1	4
7539.6250	7539.6074	0.0176	6	4	2	1	6	1	7	5	3	3	1	5	1	6
7539.6250	7539.6150	0.0100	6	4	2	0	6	2	7	5	5	1	2	6	2	6
7539.6639	7539.6638	0.0001	6	4	2	2	8	1	9	5	3	3	2	7	1	8
7539.6639	7539.6466	0.0173	6	4	2	2	8	2	9	5	3	3	2	6	2	8
7539.6639	7539.6539	0.0100	6	4	2	1	6	1	7	5	3	3	1	6	1	6
7539.6639	7539.6432	0.0207	6	6	0	2	7	2	7	5	3	3	2	5	2	6
7539.6639	7539.6701	-0.0062	6	4	2	2	8	1	7	5	3	3	2	6	1	6
7539.6639	7539.6244	0.0395	6	4	2	1	6	1	5	5	3	3	1	4	1	4
7539.7968	7539.7797	0.0171	6	4	2	1	7	2	8	5	3	3	1	6	2	7
7539.7968	7539.7781	0.0187	6	2	4	2	6	2	6	5	1	5	2	5	2	5
7539.7968	7539.7759	0.0209	6	4	2	2	5	1	6	5	3	3	2	4	1	5
7539.7968	7539.8093	-0.0125	6	4	2	1	7	1	6	5	3	3	1	6	1	5
7539.8500	7539.8577	-0.0077	6	4	2	1	7	1	8	5	3	3	1	6	1	7
7539.8500	7539.8606	-0.0106	6	4	2	1	5	1	4	5	3	3	1	4	1	3
7539.8838	7539.8984	-0.0146	6	4	2	2	8	2	10	5	3	3	2	7	2	9
7539.8838	7539.8697	0.0141	6	4	2	1	6	2	8	5	3	3	1	5	2	7
7539.8838	7539.9217	-0.0379	6	4	2	2	7	1	8	5	3	3	2	6	1	7
7539.8838	7539.9084	-0.0246	6	4	2	1	6	1	6	5	3	3	1	5	1	5
7539.8838	7539.9011	-0.0173	6	4	2	0	6	1	6	5	3	3	0	5	1	5
7539.8838	7539.9141	-0.0303	6	4	2	1	6	0	6	5	3	3	1	5	0	5
7539.8838	7539.8601	0.0237	6	4	2	0	6	2	8	5	3	3	0	5	2	7
7539.8838	7539.8801	0.0037	6	4	2	2	7	2	8	5	3	3	2	6	2	7
7539.8838	7539.8764	0.0074	6	4	2	1	7	2	6	5	3	3	1	6	2	5
7539.8838	7539.9050	-0.0212	6	4	2	2	7	1	6	5	3	3	2	6	1	5
7539.9250	7539.9421	-0.0171	6	4	2	2	8	1	8	5	3	3	2	7	1	7
7539.9250	7539.9523	-0.0273	6	4	2	2	8	0	8	5	3	3	2	7	0	7
7539.9250	7539.9013	0.0237	6	4	2	0	6	0	6	5	3	3	0	5	0	5
7540.0125	7540.0367	-0.0242	6	4	2	2	5	2	7	5	3	3	2	7	2	6
7540.0125	7540.0076	0.0049	6	4	2	2	8	2	6	5	3	3	2	7	2	5
7540.0125	7540.0335	-0.0210	6	4	2	2	5	1	5	5	3	3	2	4	1	4
7540.0125	7540.0469	-0.0344	6	4	2	2	6	2	7	5	3	3	2	5	2	6
7540.0125	7540.0341	-0.0216	6	4	2	2	5	2	7	5	5	1	2	5	2	6
7540.0125	7540.0463	-0.0338	6	4	2	2	6	2	5	5	3	3	2	4	2	4
7540.0125	7540.0450	-0.0325	6	4	2	2	5	0	5	5	5	1	2	5	2	4
7540.0125	7540.0345	-0.0220	6	4	2	2	5	2	3	5	3	3	2	3	2	2
7540.1176	7540.0920	0.0256	6	4	2	1	7	2	9	5	3	3	1	6	2	8
7540.1176	7540.1099	0.0077	6	4	2	2	6	1	7	5	3	3	2	5	1	6
7540.1176	7540.1111	0.0065	6	4	2	2	7	2	9	5	3	3	2	7	2	8
7540.1176	7540.1151	0.0025	6	4	2	1	7	0	7	5	3	3	1	6	0	6
7540.1176	7540.1009	0.0167	6	4	2	1	7	1	7	5	3	3	1	5	1	6
7540.1176	7540.1029	0.0147	6	4	2	1	5	2	7	5	3	3	1	4	2	6
7540.1176	7540.1333	-0.0157	6	4	2	1	5	1	5	5	3	3	1	5	1	4
7540.2125	7540.2037	0.0088	6	4	2	2	7	2	9	5	3	3	2	6	2	8
7540.2125	7540.1883	0.0242	6	4	2	1	5	0	5	5	3	3	1	4	0	4
7540.2125	7540.2172	-0.0047	6	4	2	1	5	2	3	5	3	3	1	4	2	2

7540.2125	7540.1971	0.0154	6	4	2	1	7	2	5	5	3	3	1	6	2	4
7540.2125	7540.2003	0.0122	6	4	2	1	5	1	5	5	3	3	1	4	1	4
7540.2125	7540.1895	0.0230	6	2	4	2	7	0	7	5	3	3	2	4	2	6
7540.2125	7540.1969	0.0156	6	4	2	2	6	2	5	5	3	3	2	5	2	4
7551.9024	7551.8996	0.0028	7	4	4	2	8	2	10	6	5	1	2	7	2	9
7551.9024	7551.9020	0.0004	7	4	4	2	8	1	8	6	5	1	2	7	1	7
7551.9024	7551.9000	0.0024	7	6	2	2	8	2	8	6	5	1	2	7	0	7
7551.9024	7551.9275	-0.0251	7	4	4	2	5	2	6	6	5	1	2	7	2	5
7551.9024	7551.9046	-0.0022	7	4	4	2	8	2	6	6	5	1	2	7	2	5
7552.0250	7551.9998	0.0252	7	4	4	2	8	1	9	6	5	1	2	7	1	8
7552.0250	7551.9869	0.0381	7	4	4	2	8	1	7	6	5	1	2	7	1	6
7552.0250	7552.0074	0.0176	7	4	4	2	9	2	10	6	5	1	2	7	2	9
7552.0879	7552.0637	0.0242	7	4	4	2	7	1	7	6	5	1	2	7	1	6
7552.0886	7552.1055	-0.0169	7	4	4	1	6	2	5	6	5	1	1	5	2	4
7552.0886	7552.1000	-0.0114	7	4	4	2	8	2	8	6	3	3	2	7	0	7
7552.0886	7552.1204	-0.0318	7	4	4	1	6	2	8	6	5	1	1	5	2	7
7552.0886	7552.1136	-0.0250	7	4	4	2	9	2	9	6	5	1	2	7	2	8
7552.0886	7552.1058	-0.0172	7	4	4	1	7	1	6	6	5	1	1	5	1	5
7552.0886	7552.0934	-0.0048	7	4	4	1	8	2	6	6	5	1	1	5	0	5
7552.1625	7552.1656	-0.0031	7	4	4	1	6	2	7	6	5	1	1	5	2	6
7552.1625	7552.1762	-0.0137	7	4	4	1	6	1	5	6	5	1	1	5	1	4
7552.1625	7552.1452	0.0173	7	4	4	1	6	1	7	6	5	1	1	5	1	6
7552.1625	7552.1494	0.0131	7	4	4	1	8	1	7	6	5	1	1	5	1	6
7552.2290	7552.2351	-0.0061	7	4	4	1	6	2	6	6	5	1	1	5	2	5
7552.2290	7552.2460	-0.0170	7	4	4	1	8	1	8	6	5	1	1	7	1	7
7552.2290	7552.2246	0.0044	7	4	4	1	8	2	6	6	5	1	1	7	2	5
7552.2290	7552.2390	-0.0100	7	4	4	1	6	2	8	6	5	1	1	7	0	7
7552.2290	7552.2447	-0.0157	7	4	4	1	7	2	7	6	5	1	1	5	2	6
7552.2683	7552.2830	-0.0147	7	4	4	1	8	2	10	6	5	1	1	7	2	9
7552.2683	7552.2869	-0.0186	7	4	4	1	8	0	8	6	5	1	1	7	0	7
7552.2683	7552.3001	-0.0318	7	4	4	1	7	1	8	6	5	1	1	7	1	7
7552.3728	7552.3607	0.0121	7	4	4	1	8	1	9	6	5	1	1	7	1	8
7552.3728	7552.3739	-0.0011	7	4	4	1	8	1	7	6	5	1	1	7	1	6
7552.3728	7552.3801	-0.0073	7	4	4	1	8	2	9	6	5	1	1	7	2	8
7552.3728	7552.3672	0.0056	7	4	4	1	8	2	7	6	5	1	1	7	2	6
7552.3728	7552.3696	0.0032	7	4	4	1	6	1	7	6	5	1	1	7	1	6
7552.4520	7552.4585	-0.0065	7	4	4	1	8	2	8	6	5	1	1	7	2	7
7552.4520	7552.4784	-0.0264	7	4	4	2	6	2	4	6	5	1	2	5	2	3
7552.5500	7552.5635	-0.0135	7	4	4	2	7	2	6	6	3	3	2	5	0	5
7552.5500	7552.5473	0.0027	7	4	4	2	5	1	6	6	5	1	2	5	1	5
7552.5500	7552.5377	0.0123	7	4	4	2	7	1	7	6	5	1	2	5	1	6
7552.5500	7552.5473	0.0027	7	6	2	2	7	2	8	6	5	1	2	5	2	7
7552.5500	7552.5282	0.0218	7	4	4	2	8	2	7	6	5	1	2	5	2	6
7552.5500	7552.5138	0.0362	7	4	4	2	9	0	9	6	5	1	2	7	2	8
7552.6311	7552.6093	0.0218	7	4	4	2	6	2	8	6	5	1	2	5	2	7
7552.6311	7552.6398	-0.0087	7	4	4	2	6	2	5	6	5	1	2	5	2	4
7552.6311	7552.6407	-0.0096	7	4	4	2	7	2	7	6	5	1	2	5	2	6
7552.6311	7552.6240	0.0071	7	4	4	2	6	1	6	6	5	1	2	5	1	5
7552.6311	7552.6111	0.0200	7	6	2	2	7	2	6	6	5	1	2	5	0	5
7552.6311	7552.6342	-0.0031	7	4	4	2	6	1	7	6	5	1	2	5	1	6
7552.6311	7552.6199	0.0112	7	4	4	2	6	1	5	6	5	1	2	5	1	4
7552.6311	7552.6150	0.0161	7	4	4	2	9	2	8	6	5	1	2	5	2	7
7560.3696	7560.3707	-0.0011	7	3	5	2	7	2	5	6	4	2	2	6	2	4

7560.4456	7560.4525	-0.0069	7	3	5	2	7	1	7	6	4	2	2	6	1	6
7560.4456	7560.4411	0.0045	7	1	7	2	7	0	7	6	4	2	2	6	0	6
7560.5131	7560.5137	-0.0006	7	3	5	2	7	2	9	6	4	2	2	6	2	8
7560.8250	7560.8282	-0.0032	7	3	5	2	7	2	6	6	4	2	2	6	2	5
7560.8988	7560.9016	-0.0028	7	3	5	2	7	1	8	6	4	2	2	6	1	7
7561.0338	7560.9977	0.0361	7	3	5	2	8	2	6	6	4	2	2	7	2	5
7561.0762	7561.0888	-0.0126	7	3	5	2	8	1	8	6	4	2	2	7	1	7
7561.0762	7561.0775	-0.0013	7	1	7	2	8	0	8	6	4	2	2	7	0	7
7561.0762	7561.0454	0.0308	7	3	5	2	7	2	8	6	4	2	2	6	2	7
7561.0762	7561.0756	0.0006	7	3	5	1	6	2	4	6	4	2	1	5	2	3
7561.1558	7561.1550	0.0008	7	3	5	1	6	0	6	6	4	2	1	5	0	5
7561.1558	7561.1555	0.0003	7	3	5	2	8	2	10	6	4	2	2	7	2	9
7561.1558	7561.1669	-0.0111	7	3	5	1	6	1	6	6	4	2	1	5	1	5
7561.1558	7561.1920	-0.0362	7	3	5	1	8	2	6	6	4	2	1	7	2	5
7561.2375	7561.2648	-0.0273	7	3	5	1	8	0	8	6	4	2	1	7	0	7
7561.2375	7561.2333	0.0042	7	3	5	1	6	2	8	6	4	2	1	5	2	7
7561.2679	7561.2763	-0.0084	7	3	5	1	8	1	8	6	4	2	1	7	1	7
7561.3409	7561.3387	0.0022	7	3	5	1	8	2	10	6	4	2	1	7	2	9
7561.3409	7561.3437	-0.0028	7	3	5	2	6	2	4	6	4	2	2	5	2	3
7561.4140	7561.4173	-0.0033	7	3	5	2	6	1	6	6	4	2	2	5	1	5
7561.4140	7561.4051	0.0089	7	3	5	2	6	0	6	6	4	2	2	5	0	5
7561.4875	7561.4755	0.0120	7	3	5	2	6	2	8	6	4	2	2	5	2	7
7561.4875	7561.4541	0.0334	7	3	5	2	8	2	7	6	4	2	2	7	2	6
7561.5375	7561.5446	-0.0071	7	3	5	2	8	1	9	6	4	2	2	7	1	8
7561.5375	7561.5293	0.0082	7	3	5	1	6	2	5	6	4	2	1	5	2	4
7561.6059	7561.6240	-0.0181	7	3	5	1	6	1	7	6	4	2	1	5	1	6
7561.6059	7561.6152	-0.0093	7	3	5	2	8	1	7	6	4	2	2	7	1	6
7561.6059	7561.6406	-0.0347	7	3	5	1	8	2	7	6	4	2	1	7	2	6
7561.6625	7561.6769	-0.0144	7	3	5	2	8	2	9	6	4	2	2	7	2	8
7561.6625	7561.6979	-0.0354	7	3	5	1	6	1	5	6	4	2	1	5	1	4
7561.7056	7561.7325	-0.0269	7	3	5	1	8	1	9	6	4	2	1	7	1	8
7561.7958	7561.8101	-0.0143	7	3	5	1	8	1	7	6	4	2	1	7	1	6
7561.7958	7561.8328	-0.0370	7	3	5	2	9	2	7	6	4	2	2	8	2	6
7561.8600	7561.8637	-0.0037	7	3	5	1	8	2	9	6	4	2	1	7	2	8
7561.8600	7561.8684	-0.0084	7	3	5	2	6	1	7	6	4	2	2	5	1	6
7561.9125	7561.9266	-0.0141	7	3	5	2	9	1	9	6	4	2	2	8	1	8
7561.9125	7561.9153	-0.0028	7	3	5	2	9	0	9	6	4	2	2	8	0	8
7561.9125	7561.9308	-0.0183	7	3	5	1	7	2	5	6	4	2	1	6	2	4
7561.9125	7561.9490	-0.0365	7	3	5	0	7	2	5	6	4	2	0	6	2	4
7561.9758	7561.9946	-0.0188	7	3	5	2	9	2	11	6	4	2	2	8	2	10
7561.9758	7562.0100	-0.0342	7	3	5	1	7	0	7	6	4	2	1	6	0	6
7561.9758	7561.9943	-0.0185	7	3	5	2	6	2	7	6	4	2	2	5	2	6
7562.0844	7562.0879	-0.0035	7	3	5	1	7	2	9	6	4	2	1	6	2	8
7562.0844	7562.1017	-0.0173	7	3	5	0	7	2	9	6	4	2	0	6	2	8
7562.0844	7562.0552	0.0292	7	3	5	2	8	0	8	6	4	2	2	7	2	7
7562.1500	7562.1364	0.0136	7	3	5	1	6	2	6	6	4	2	1	5	2	5
7562.1500	7562.1469	0.0031	7	3	5	2	5	1	5	6	4	2	2	4	1	4
7562.1500	7562.1343	0.0157	7	3	5	2	5	0	5	6	4	2	2	4	0	4
7562.2545	7562.2465	0.0080	7	3	5	1	8	2	8	6	4	2	1	7	2	7
7562.2545	7562.2792	-0.0247	7	3	5	2	9	2	8	6	4	2	2	8	2	7
7562.3875	7562.3888	-0.0013	7	3	5	2	9	1	10	6	4	2	2	8	1	9
7562.3875	7562.3850	0.0025	7	3	5	0	7	2	6	6	4	2	0	6	2	5
7562.3875	7562.3624	0.0251	7	3	5	1	7	2	6	6	4	2	1	6	2	5

7562.3875	7562.3786	0.0089	7	3	5	2	6	2	6	6	4	2	2	5	2	5
7562.5208	7562.5100	0.0108	7	3	5	2	9	2	10	6	4	2	2	8	2	9
7562.5208	7562.4962	0.0246	7	3	5	0	7	1	8	6	4	2	0	6	1	7
7562.5208	7562.4936	0.0272	7	3	5	2	5	2	4	6	4	2	2	4	2	3
7562.6035	7562.6031	0.0004	7	3	5	1	7	2	8	6	4	2	1	6	2	7
7562.6035	7562.6164	-0.0129	7	3	5	0	7	2	8	6	4	2	0	6	2	7
7562.6035	7562.6038	-0.0003	7	3	5	2	5	1	6	6	4	2	2	4	1	5
7562.6875	7562.6861	0.0014	7	3	5	2	5	1	4	6	4	2	2	4	1	3
7562.7151	7562.7242	-0.0091	7	3	5	2	5	2	6	6	4	2	2	4	2	5
7562.8870	7562.8923	-0.0053	7	3	5	2	9	2	9	6	4	2	2	8	2	8
7562.9885	7562.9849	0.0036	7	3	5	1	7	2	7	6	4	2	1	6	2	6
7562.9885	7563.0017	-0.0132	7	3	5	0	7	2	7	6	4	2	0	6	2	6
7563.0949	7563.1059	-0.0110	7	3	5	2	5	2	5	6	4	2	2	4	2	4
7617.1565	7617.1629	-0.0064	6	5	2	2	8	2	10	5	4	1	2	7	2	9
7617.1565	7617.1837	-0.0272	6	5	2	2	8	1	9	5	4	1	2	7	1	8
7617.1565	7617.1604	-0.0039	6	5	2	2	8	1	8	5	4	1	2	7	1	7
7617.1565	7617.1612	-0.0047	6	5	2	2	8	0	8	5	4	1	2	7	0	7
7617.1565	7617.1554	0.0011	6	5	2	0	6	2	8	5	4	1	0	5	2	7
7617.1565	7617.1564	0.0001	6	5	2	1	6	2	8	5	4	1	1	5	2	7
7617.1565	7617.1955	-0.0390	6	5	2	2	8	1	7	5	4	1	2	7	1	6
7617.1565	7617.1588	-0.0023	6	5	2	1	6	1	7	5	4	1	1	5	1	6
7617.1565	7617.1601	-0.0036	6	5	2	0	6	1	7	5	4	1	0	5	1	6
7617.1565	7617.1919	-0.0354	6	5	2	2	8	2	7	5	4	1	2	7	2	6
7617.4074	7617.4269	-0.0195	6	5	2	1	7	2	9	5	4	1	1	6	2	8
7617.4074	7617.4160	-0.0086	6	5	2	1	7	1	7	5	4	1	1	6	1	6
7617.4074	7617.4130	-0.0056	6	5	2	1	7	0	7	5	4	1	1	6	0	6
7617.4074	7617.4364	-0.0290	6	5	2	2	5	2	7	5	4	1	2	4	2	6
7617.4074	7617.4210	-0.0136	6	5	2	1	7	2	6	5	4	1	1	6	2	5
7617.4792	7617.4786	0.0006	6	5	2	2	7	2	9	5	4	1	2	6	2	8
7617.4792	7617.4475	0.0317	6	5	2	1	7	1	8	5	4	1	1	6	1	7
7617.4792	7617.4559	0.0233	6	5	2	1	7	2	8	5	4	1	1	6	2	7
7617.4792	7617.4834	-0.0042	6	5	2	2	7	1	8	5	4	1	2	6	1	7
7617.4792	7617.4882	-0.0090	6	5	2	2	7	2	8	5	4	1	2	6	2	7
7617.4792	7617.4523	0.0269	6	5	2	1	5	2	7	5	4	1	1	4	2	6
7617.4792	7617.4797	-0.0005	6	5	2	1	7	2	7	5	4	1	1	6	2	6
7617.4792	7617.4824	-0.0032	6	5	2	2	7	1	7	5	4	1	2	6	1	6
7617.4792	7617.4865	-0.0073	6	5	2	2	7	0	7	5	4	1	2	6	0	6
7617.4792	7617.4847	-0.0055	6	5	2	2	7	2	7	5	4	1	2	6	2	6
7617.7420	7617.7425	-0.0005	6	5	2	2	6	2	8	5	4	1	2	5	2	7
7617.7420	7617.7427	-0.0007	6	5	2	2	6	1	7	5	4	1	2	5	1	6
7617.7420	7617.7361	0.0059	6	5	2	2	6	2	7	5	4	1	2	5	2	6
7617.7420	7617.7339	0.0081	6	5	2	2	6	1	6	5	4	1	2	5	1	5
7617.7420	7617.7302	0.0118	6	5	2	2	6	0	6	5	4	1	2	5	0	5
7617.7420	7617.7162	0.0258	6	5	2	2	6	2	6	5	4	1	2	5	2	5
7617.7420	7617.7552	-0.0132	6	5	2	2	6	1	5	5	4	1	2	5	1	4
7617.7420	7617.7048	0.0372	6	5	2	2	6	2	5	5	4	1	2	5	2	4
7617.7420	7617.7198	0.0222	6	5	2	2	6	2	4	5	4	1	2	5	2	3
7626.6750	7626.6699	0.0051	6	5	1	2	8	2	8	5	4	2	2	7	2	7
7626.6750	7626.7105	-0.0355	6	5	1	2	8	2	7	5	4	2	2	7	2	6
7626.6750	7626.6902	-0.0152	6	5	1	1	6	2	7	5	4	2	1	5	2	6
7626.6750	7626.6920	-0.0170	6	3	3	0	6	2	7	5	4	2	0	5	2	6
7626.6750	7626.6491	0.0259	6	5	1	1	6	2	6	5	4	2	1	5	2	5
7626.7150	7626.7400	-0.0250	6	5	1	2	8	2	10	5	4	2	2	7	2	9

7626.7150	7626.7051	0.0099	6	5	1	2	8	1	9	5	4	2	2	7	1	8
7626.7150	7626.7062	0.0088	6	5	1	2	8	2	9	5	4	2	2	7	2	8
7626.7150	7626.7418	-0.0268	6	5	1	2	8	1	8	5	4	2	2	7	1	7
7626.7150	7626.7421	-0.0271	6	5	1	2	8	0	8	5	4	2	2	7	0	7
7626.7150	7626.7297	-0.0147	6	5	1	0	6	2	8	5	4	2	0	5	2	7
7626.7150	7626.7281	-0.0131	6	5	1	1	6	2	8	5	4	2	1	5	2	7
7626.7150	7626.7064	0.0086	6	5	1	2	8	1	7	5	4	2	2	7	1	6
7626.7150	7626.6863	0.0287	6	5	1	1	6	1	7	5	4	2	1	5	1	6
7626.7150	7626.6885	0.0265	6	5	1	0	6	1	7	5	4	2	0	5	1	6
7626.9603	7626.9769	-0.0166	6	5	1	1	7	2	9	5	4	2	1	6	2	8
7626.9603	7626.9420	0.0183	6	5	1	1	7	1	8	5	4	2	1	6	1	7
7626.9603	7626.9395	0.0208	6	5	1	1	7	2	8	5	4	2	1	6	2	7
7626.9603	7626.9741	-0.0138	6	5	1	2	7	1	8	5	4	2	2	6	1	7
7626.9603	7626.9693	-0.0090	6	5	1	2	7	2	8	5	4	2	2	6	2	7
7626.9603	7626.9759	-0.0156	6	5	1	1	7	1	7	5	4	2	1	6	1	6
7626.9603	7626.9746	-0.0143	6	5	1	1	7	0	7	5	4	2	1	6	0	6
7626.9603	7626.9945	-0.0342	6	5	1	1	5	2	7	5	4	2	1	4	2	6
7626.9603	7626.9768	-0.0165	6	5	1	2	5	2	7	5	4	2	2	4	2	6
7626.9603	7626.9414	0.0189	6	5	1	1	7	1	6	5	4	2	1	6	1	5
7627.1975	7627.2112	-0.0137	6	5	1	2	6	1	7	5	4	2	2	5	1	6
7627.1975	7627.2022	-0.0047	6	5	1	0	6	2	7	5	4	2	2	5	2	6
7627.1975	7627.2007	-0.0032	6	5	1	0	6	2	5	5	4	2	2	5	2	4
7627.2295	7627.2537	-0.0242	6	5	1	2	6	2	8	5	4	2	2	5	2	7
7627.2295	7627.2542	-0.0247	6	5	1	2	6	1	6	5	4	2	2	5	1	5
7627.2295	7627.2526	-0.0231	6	5	1	2	6	0	6	5	4	2	2	5	0	5
7627.2295	7627.2120	0.0175	6	5	1	2	6	1	5	5	4	2	2	5	1	4
7627.2295	7627.2526	-0.0231	6	5	1	2	6	2	4	5	4	2	2	5	2	3
7633.3905	7633.4078	-0.0173	6	3	3	1	7	2	8	5	2	4	1	6	2	7
7633.3905	7633.3965	-0.0060	6	3	3	2	8	2	9	5	2	4	2	7	2	8
7633.3905	7633.3983	-0.0078	6	3	3	2	7	2	8	5	2	4	2	6	2	7
7633.3905	7633.3875	0.0030	6	3	3	1	5	2	6	5	2	4	1	4	2	5
7633.3905	7633.3894	0.0011	6	3	3	1	6	1	5	5	2	4	1	5	1	4
7633.4537	7633.4741	-0.0204	6	3	3	2	8	1	9	5	2	4	2	7	1	8
7633.4537	7633.4682	-0.0145	6	3	3	0	6	1	7	5	2	4	0	5	1	6
7633.4537	7633.4589	-0.0052	6	3	3	2	5	1	4	5	2	4	2	4	1	3
7633.4537	7633.4892	-0.0355	6	3	3	1	5	1	6	5	2	4	1	6	1	5
7649.8559	7649.8628	-0.0069	7	2	5	2	7	2	7	6	5	2	2	6	2	6
7649.8559	7649.8811	-0.0252	7	2	5	2	7	2	6	6	5	2	2	6	2	5
7650.1336	7650.1438	-0.0102	7	4	3	2	7	1	8	6	5	2	2	6	1	7
7650.1336	7650.1169	0.0167	7	4	3	2	7	1	6	6	5	2	2	6	1	5
7650.1336	7650.1298	0.0038	7	4	3	2	7	2	8	6	5	2	2	6	2	7
7650.2616	7650.2578	0.0038	7	4	3	2	7	2	6	6	5	2	2	6	2	5
7650.2616	7650.3014	-0.0398	7	4	3	2	7	2	5	6	5	2	2	6	2	4
7650.2616	7650.2715	-0.0099	7	4	3	2	6	1	7	6	5	2	2	6	1	6
7650.4048	7650.4015	0.0033	7	4	3	2	7	2	9	6	5	2	2	6	2	8
7650.4617	7650.4635	-0.0018	7	4	3	2	7	0	7	6	5	2	2	6	0	6
7650.4617	7650.4673	-0.0056	7	4	3	2	7	1	7	6	5	2	2	6	1	6
7650.9802	7650.9810	-0.0008	7	4	3	2	8	2	8	6	5	2	2	7	2	7
7651.0977	7651.0941	0.0036	7	4	3	2	8	2	9	6	5	2	2	7	2	8
7651.2105	7651.2455	-0.0350	7	4	3	2	8	1	7	6	5	2	2	7	1	6
7651.2105	7651.2056	0.0049	7	4	3	1	6	2	6	6	5	2	1	5	2	5
7651.2105	7651.2120	-0.0015	7	4	3	2	7	2	8	6	5	2	2	7	2	7
7651.2950	7651.3010	-0.0060	7	4	3	2	8	1	9	6	5	2	2	7	1	8

7651.2950	7651.2806	0.0144	7	4	3	2	9	2	9	6	5	2	2	7	2	8
7651.4355	7651.4445	-0.0090	7	4	3	1	8	2	8	6	5	2	1	7	2	7
7651.4355	7651.4611	-0.0256	7	4	3	1	6	1	7	6	5	2	1	5	1	6
7651.4355	7651.4417	-0.0062	7	4	3	1	6	1	5	6	5	2	1	5	1	4
7651.4355	7651.4640	-0.0285	7	4	3	1	6	2	7	6	5	2	1	5	2	6
7651.4355	7651.4541	-0.0186	7	4	3	0	7	2	6	6	5	2	2	7	2	5
7651.5875	7651.5673	0.0202	7	4	3	2	8	2	10	6	5	2	2	7	2	9
7651.5875	7651.5917	-0.0042	7	2	5	2	7	2	6	6	5	2	2	5	2	5
7651.7179	7651.6903	0.0276	7	4	3	1	8	1	9	6	5	2	1	7	1	8
7651.7179	7651.7003	0.0176	7	4	3	1	6	2	8	6	5	2	1	5	2	7
7651.7179	7651.7136	0.0043	7	4	3	1	6	1	6	6	5	2	1	5	1	5
7651.7179	7651.7162	0.0017	7	4	3	1	6	0	6	6	5	2	1	5	0	5
7651.7179	7651.7116	0.0063	7	4	3	1	6	2	4	6	5	2	1	5	2	3
7651.8875	7651.8874	0.0001	7	4	3	1	8	2	10	6	5	2	1	7	2	9
7651.8875	7651.8860	0.0015	7	4	3	2	7	2	7	6	5	2	2	5	2	6
7651.9327	7651.9308	0.0019	7	4	3	1	8	0	8	6	5	2	1	7	0	7
7651.9327	7651.9285	0.0042	7	4	3	1	8	1	8	6	5	2	1	7	1	7
7651.9327	7651.9142	0.0185	7	4	3	2	6	1	7	6	5	2	2	5	1	6
7651.9327	7651.9343	-0.0016	7	4	3	2	6	1	5	6	5	2	2	5	1	4
7651.9660	7651.9860	-0.0200	7	4	3	1	8	2	6	6	5	2	1	7	2	5
7651.9660	7651.9683	-0.0023	7	4	3	2	7	2	6	6	5	2	2	5	2	5
7652.2353	7652.2271	0.0082	7	4	3	2	6	2	8	6	5	2	2	5	2	7
7652.2717	7652.2729	-0.0012	7	4	3	2	6	0	6	6	5	2	2	5	0	5
7652.2717	7652.2770	-0.0053	7	4	3	2	6	1	6	6	5	2	2	5	1	5
7652.5483	7652.5511	-0.0028	7	4	3	2	8	2	9	6	5	2	2	8	2	8
7652.7692	7652.7720	-0.0028	7	4	3	2	9	2	10	6	5	2	2	8	2	9
7652.7692	7652.7761	-0.0069	7	4	3	0	7	2	7	6	5	2	0	6	2	6
7652.7692	7652.7405	0.0287	7	4	3	1	7	2	7	6	5	2	1	6	2	6
7652.7692	7652.7376	0.0316	7	4	3	2	9	2	9	6	5	2	2	8	2	8
7652.8861	7652.8778	0.0083	7	4	3	2	9	1	10	6	5	2	2	8	1	9
7652.8861	7652.8897	-0.0036	7	4	3	2	9	2	8	6	5	2	2	8	2	7
7653.0326	7653.0072	0.0254	7	4	3	0	7	2	8	6	5	2	0	6	2	7
7653.0326	7653.0158	0.0168	7	4	3	1	7	1	6	6	5	2	1	6	1	5
7653.0326	7652.9918	0.0408	7	4	3	2	5	2	5	6	5	2	2	4	2	4
7653.0977	7653.0749	0.0228	7	4	3	2	9	2	11	6	5	2	2	8	2	10
7653.0977	7653.1048	-0.0071	7	4	3	2	9	1	9	6	5	2	2	8	1	8
7653.0977	7653.0769	0.0208	7	4	3	0	7	1	8	6	5	2	0	6	1	7
7653.0977	7653.0568	0.0409	7	4	3	0	7	1	6	6	5	2	0	6	1	5
7653.0977	7653.0649	0.0328	7	4	3	0	7	2	6	6	5	2	0	6	2	5
7653.2460	7653.2773	-0.0313	7	4	3	1	7	2	9	6	5	2	1	6	2	8
7653.2460	7653.2524	-0.0064	7	4	3	2	5	2	6	6	5	2	2	4	2	5
7653.2460	7653.2429	0.0031	7	4	3	2	5	2	4	6	5	2	2	4	2	3
7653.2995	7653.3080	-0.0085	7	4	3	0	7	2	9	6	5	2	0	6	2	8
7653.2995	7653.3010	-0.0015	7	4	3	1	7	1	7	6	5	2	1	6	1	6
7653.2995	7653.3040	-0.0045	7	4	3	1	7	0	7	6	5	2	1	6	0	6
7653.2995	7653.2852	0.0143	7	4	3	2	5	1	6	6	5	2	2	4	1	5
7653.2995	7653.3285	-0.0290	7	4	3	1	7	2	5	6	5	2	1	6	2	4
7653.2995	7653.3021	-0.0026	7	4	3	2	5	1	4	6	5	2	2	4	1	3
7653.5652	7653.5544	0.0108	7	4	3	2	5	2	7	6	5	2	2	4	2	6
7653.5652	7653.5689	-0.0037	7	4	3	2	5	1	5	6	5	2	2	4	1	4
7653.5652	7653.5577	0.0075	7	4	3	2	5	0	5	6	5	2	2	4	0	4
7653.5652	7653.5724	-0.0072	7	4	3	2	5	2	3	6	5	2	2	4	2	2
7762.7628	7762.7729	-0.0101	6	6	1	2	8	2	10	5	5	0	2	7	2	9

7762.7628	7762.7604	0.0024	6	6	1	2	8	1	9	5	5	0	2	7	1	8
7762.7628	7762.7680	-0.0052	6	6	1	2	8	2	9	5	5	0	2	7	2	8
7762.7628	7762.7702	-0.0074	6	6	1	2	8	1	8	5	5	0	2	7	1	7
7762.7628	7762.7693	-0.0065	6	6	1	2	8	0	8	5	5	0	2	7	0	7
7762.7628	7762.7537	0.0091	6	6	1	2	8	2	8	5	5	0	2	7	2	7
7762.7628	7762.7656	-0.0028	6	6	1	2	8	1	7	5	5	0	2	7	1	6
7762.7628	7762.7888	-0.0260	6	6	1	1	6	1	7	5	5	0	1	5	1	6
7762.7628	7762.7563	0.0065	6	6	1	2	8	2	7	5	5	0	2	7	2	6
7762.7628	7762.8003	-0.0375	6	6	1	1	6	2	7	5	5	0	1	5	2	6
7762.7628	7762.8015	-0.0387	6	6	1	1	6	0	6	5	5	0	1	5	0	5
7762.8927	7762.8717	0.0210	6	6	1	2	7	2	9	5	5	0	2	6	2	8
7762.8927	7762.8813	0.0114	6	6	1	1	5	2	7	5	5	0	1	4	2	6
7762.8927	7762.8708	0.0219	6	6	1	2	7	1	7	5	5	0	2	6	1	6
7762.8927	7762.8701	0.0226	6	6	1	2	7	0	7	5	5	0	2	6	0	6
7762.8927	7762.8606	0.0321	6	6	1	1	5	1	6	5	5	0	1	4	1	5
7762.8927	7762.8901	0.0026	6	6	1	2	4	2	6	5	5	0	2	3	2	5
7762.8927	7762.8751	0.0176	6	6	1	1	5	2	6	5	5	0	1	4	2	5
7762.8943	7762.9261	-0.0318	6	6	1	1	7	2	9	5	5	0	1	6	2	8
7762.8943	7762.9135	-0.0192	6	6	1	1	7	1	8	5	5	0	1	6	1	7
7762.8943	7762.9217	-0.0274	6	6	1	1	7	2	8	5	5	0	1	6	2	7
7762.8943	7762.9210	-0.0267	6	6	1	1	7	1	7	5	5	0	1	6	1	6
7762.8943	7762.9188	-0.0245	6	6	1	1	7	0	7	5	5	0	1	6	0	6
7762.8943	7762.9078	-0.0135	6	6	1	1	7	2	7	5	5	0	1	6	2	6
7762.8943	7762.9194	-0.0251	6	6	1	1	7	1	6	5	5	0	1	6	1	5
7762.8943	7762.9007	-0.0064	6	6	1	1	7	2	6	5	5	0	1	6	2	5
7762.8943	7762.9119	-0.0176	6	6	1	1	7	2	5	5	5	0	1	6	2	4
7763.0057	7763.0239	-0.0182	6	6	0	1	6	2	8	5	5	1	1	5	2	7
7763.0057	7763.0436	-0.0379	6	6	0	0	6	2	8	5	5	1	0	5	2	7
7763.0057	7763.0435	-0.0378	6	6	1	2	6	2	8	5	5	0	2	5	2	7
7763.0057	7763.0218	-0.0161	6	6	0	0	6	1	7	5	5	1	0	5	1	6
7763.0057	7763.0131	-0.0074	6	6	0	1	6	2	7	5	5	1	1	5	2	6
7763.0057	7763.0326	-0.0269	6	6	0	0	6	2	7	5	3	3	0	5	2	6
7763.0057	7763.0362	-0.0305	6	4	2	2	7	0	7	5	3	3	2	6	0	6
7763.0057	7763.0225	-0.0168	6	6	1	2	6	2	7	5	5	0	2	5	2	6
7763.0058	7762.9877	0.0181	6	6	0	2	8	2	10	5	5	1	2	7	2	9
7763.0058	7762.9736	0.0322	6	6	0	2	8	1	9	5	5	1	2	7	1	8
7763.0058	7762.9807	0.0251	6	6	0	2	8	2	9	5	5	1	2	7	2	8
7763.0058	7762.9852	0.0206	6	6	0	2	8	1	8	5	5	1	2	7	1	7
7763.0058	7762.9843	0.0215	6	6	0	2	8	0	8	5	5	1	2	7	0	7
7763.0058	7762.9785	0.0273	6	6	0	2	8	1	7	5	5	1	2	7	1	6
7763.0058	7763.0022	0.0036	6	6	0	1	6	1	7	5	5	1	1	5	1	6
7763.0058	7762.9697	0.0361	6	6	0	2	8	2	7	5	5	1	2	7	2	6
7763.0058	7763.0192	-0.0134	6	6	1	2	6	1	7	5	5	0	2	5	1	6
7763.1081	7763.0852	0.0229	6	6	0	2	7	2	9	5	5	1	2	6	2	8
7763.1081	7763.0949	0.0132	6	6	0	1	5	2	7	5	5	1	1	4	2	6
7763.1081	7763.0844	0.0237	6	6	0	2	7	1	7	5	5	1	2	6	1	6
7763.1081	7763.0837	0.0244	6	6	0	2	7	0	7	5	5	1	2	6	0	6
7763.1081	7763.0728	0.0353	6	6	0	1	5	1	6	5	5	1	1	4	1	5
7763.1081	7763.1048	0.0033	6	6	0	2	4	2	6	5	5	1	2	3	2	5
7763.1081	7763.0866	0.0215	6	6	0	1	5	2	6	5	5	1	1	4	2	5
7763.1125	7763.1400	-0.0275	6	6	0	1	7	2	9	5	5	1	1	6	2	8
7763.1125	7763.1258	-0.0133	6	6	0	1	7	1	8	5	5	1	1	6	1	7
7763.1125	7763.1335	-0.0210	6	6	0	1	7	2	8	5	5	1	1	6	2	7

7763.1125	7763.1352	-0.0227	6	6	0	1	7	1	7	5	5	1	1	6	1	6
7763.1125	7763.1330	-0.0205	6	6	0	1	7	0	7	5	5	1	1	6	0	6
7763.1125	7763.1187	-0.0062	6	6	0	1	7	2	7	5	5	1	1	6	2	6
7763.1125	7763.1314	-0.0189	6	6	0	1	7	1	6	5	5	1	1	6	1	5
7763.1125	7763.1135	-0.0010	6	6	0	1	7	2	6	5	5	1	1	6	2	5
7763.1125	7763.1264	-0.0139	6	6	0	1	7	2	5	5	5	1	1	6	2	4
7763.2250	7763.2305	-0.0055	6	6	0	2	6	1	7	5	5	1	2	5	1	6
7763.2250	7763.2335	-0.0085	6	6	0	2	6	2	7	5	5	1	0	5	2	6
7763.2250	7763.2060	0.0190	6	6	0	2	5	1	6	5	5	1	2	4	1	5
7763.2250	7763.2028	0.0222	6	6	0	2	6	2	6	5	5	1	2	5	2	5
7763.2250	7763.2055	0.0195	6	6	0	2	5	2	6	5	5	1	2	4	2	5
7763.2250	7763.2357	-0.0107	6	6	0	2	6	1	5	5	5	1	2	5	1	4
7763.2250	7763.2364	-0.0114	6	6	0	2	5	0	5	5	5	1	2	4	0	4
7763.2250	7763.2166	0.0084	6	6	0	2	6	2	5	5	5	1	0	5	2	4
7763.2250	7763.2074	0.0176	6	6	0	2	5	1	4	5	5	1	2	4	1	3
7763.2250	7763.1872	0.0378	6	6	0	2	5	2	4	5	5	1	2	4	2	3
7763.2500	7763.2561	-0.0061	6	6	0	2	6	2	8	5	5	1	2	5	2	7
7763.2500	7763.2434	0.0066	6	6	0	2	5	2	7	5	5	1	2	4	2	6
7763.2500	7763.2530	-0.0030	6	6	0	2	6	1	6	5	5	1	2	5	1	5
7763.2500	7763.2506	-0.0006	6	6	0	2	6	0	6	5	5	1	2	5	0	5
7763.2500	7763.2412	0.0088	6	6	0	2	5	1	5	5	5	1	2	4	1	4
7927.9570	7927.9475	0.0095	7	3	4	2	8	2	9	6	4	3	2	6	2	8
7927.9570	7927.9569	0.0001	7	3	4	2	8	1	7	6	4	3	2	6	1	6
7928.8029	7928.8005	0.0024	7	3	4	1	8	2	10	6	4	3	1	7	2	9
7928.8029	7928.8066	-0.0037	7	3	4	1	8	1	8	6	4	3	1	7	1	7
7928.8029	7928.7973	0.0056	7	3	4	1	8	0	8	6	4	3	1	7	0	7
7928.8029	7928.7674	0.0355	7	3	4	2	6	1	7	6	4	3	2	5	1	6
7928.8029	7928.8227	-0.0198	7	3	4	1	8	2	6	6	4	3	1	7	2	5
7928.9899	7928.9880	0.0019	7	3	4	1	8	2	7	6	4	3	1	6	2	6
7928.9899	7928.9537	0.0362	7	3	4	2	8	2	10	6	4	3	2	8	2	9
7928.9899	7929.0056	-0.0157	7	3	4	1	7	2	8	6	4	3	1	5	2	7
7928.9899	7928.9870	0.0029	7	3	4	1	7	2	8	6	4	3	1	7	0	7
7928.9899	7928.9951	-0.0052	7	3	4	2	8	1	8	6	4	3	2	8	1	7
7928.9899	7928.9718	0.0181	7	3	4	1	7	1	6	6	4	3	1	5	1	5
7928.9899	7929.0103	-0.0204	7	3	4	0	7	2	7	6	4	3	2	5	2	6
7928.9899	7928.9870	0.0029	7	3	4	2	6	1	6	6	4	3	2	6	1	5
7928.9899	7928.9917	-0.0018	7	3	4	2	9	2	8	6	4	3	2	7	0	7
7928.9899	7929.0254	-0.0355	7	3	4	0	7	1	6	6	4	3	2	5	1	5
7929.0728	7929.0720	0.0008	7	3	4	2	9	2	9	6	4	3	2	8	2	8
7929.0728	7929.0479	0.0249	7	3	4	0	7	2	8	6	4	3	2	5	2	7
7929.2449	7929.2587	-0.0138	7	3	4	0	7	2	7	6	4	3	0	6	0	6
7929.2449	7929.2547	-0.0098	7	3	4	1	7	2	7	6	4	3	1	6	2	6
7929.2449	7929.2624	-0.0175	7	3	4	2	6	2	8	6	4	3	0	6	2	7
7929.2449	7929.2759	-0.0310	7	3	4	2	6	1	6	6	4	3	2	4	1	5
7929.3424	7929.3292	0.0132	7	3	4	2	9	1	10	6	4	3	2	8	1	9
7929.3424	7929.3399	0.0025	7	3	4	2	9	2	10	6	4	3	2	8	2	9
7929.3424	7929.3381	0.0043	7	3	4	2	9	1	8	6	4	3	2	8	1	7
7929.3424	7929.3452	-0.0028	7	3	4	2	9	2	8	6	4	3	2	8	2	7
7929.4872	7929.4809	0.0063	7	3	4	0	7	1	8	6	4	3	0	6	1	7
7929.4872	7929.4681	0.0191	7	3	4	1	7	1	8	6	4	3	1	6	1	7
7929.4872	7929.4695	0.0177	7	3	4	0	7	1	6	6	4	3	0	6	1	5
7929.4872	7929.4477	0.0395	7	3	4	1	7	2	8	6	4	3	1	6	2	7
7929.4872	7929.4636	0.0236	7	3	4	0	7	2	8	6	4	3	0	6	2	7

7929.5817	7929.5792	0.0025	7	3	4	2	9	2	11	6	4	3	2	8	2	10
7929.5817	7929.5834	-0.0017	7	3	4	2	9	0	9	6	4	3	2	8	0	8
7929.5817	7929.5815	0.0002	7	3	4	2	9	1	9	6	4	3	2	8	1	8
7929.5817	7929.5726	0.0091	7	3	4	2	9	2	7	6	4	3	2	8	2	6
7929.5817	7929.5603	0.0214	7	3	4	2	5	1	4	6	4	3	2	4	1	3
7929.6979	7929.6725	0.0254	7	3	4	1	7	2	9	6	4	3	1	6	2	8
7929.6979	7929.6887	0.0092	7	3	4	0	7	2	9	6	4	3	0	6	2	8
7929.6979	7929.6960	0.0019	7	3	4	1	7	0	7	6	4	3	1	6	0	6
7929.6979	7929.7088	-0.0109	7	3	4	0	7	1	7	6	4	3	0	6	1	6
7929.6979	7929.6919	0.0060	7	3	4	1	7	1	7	6	4	3	1	6	1	6
7929.6979	7929.7083	-0.0104	7	3	4	0	7	0	7	6	4	3	2	4	2	6
7929.6979	7929.7127	-0.0148	7	3	4	1	7	2	5	6	4	3	1	6	2	4
7929.6979	7929.7266	-0.0287	7	3	4	2	9	2	7	6	4	3	2	7	2	6
7929.8106	7929.7994	0.0112	7	3	4	2	5	2	7	6	6	1	2	6	2	6
7929.8598	7929.8397	0.0201	7	3	4	2	5	1	5	6	4	3	2	4	1	4
7929.8598	7929.8513	0.0085	7	3	4	2	5	0	5	6	4	3	2	4	0	4
7929.8598	7929.8362	0.0236	7	3	4	1	7	2	5	6	4	3	1	5	2	4
7929.9184	7929.9131	0.0053	7	3	4	2	5	2	3	6	4	3	2	4	2	2
7929.9184	7929.9125	0.0059	7	3	4	0	7	2	5	6	4	3	2	5	2	4

4.4. Cluster of 3,3-diethyl-1,1,2,2-tetracyanocyclopropane with tetrahydrofuran

Table 11: Transition frequencies for the 3,3-diethyl-1,1,2,2-tetracyanocyclopropane complex with tetrahydrofuran. J is the rotational angular momentum quantum number, K_a and K_c are the projections of J onto the principal axes at the prolate and oblate symmetric top limits, respectively, and $I_{7,9}$, F_1, $I_{8,10}$ and F_2 are angular momentum quantum numbers obtained using the following coupling schemes between the rotational angular momentum and the nuclear spins of the four nitrogen nuclei ($I(^{14}N) = 1$): $\mathbf{I_{7,9}} = \mathbf{I_{N7}} + \mathbf{I_{N9}}$, $\mathbf{F_1} = \mathbf{J} + \mathbf{I_{7,9}}$, $\mathbf{I_{8,10}} = \mathbf{I_{N8}} + \mathbf{I_{N10}}$ and $\mathbf{F_2} = \mathbf{F_1} + \mathbf{I_{8,10}}$

Observed Freq. /MHz	Calculated Freq. /MHz	Difference / MHz	J	K_a	K_c	$I_{7,9}$	F_1	$I_{8,10}$	F_2	J'	K'_a	K'_c	$I'_{7,9}$	F'_1	$I'_{8,10}$	F'_2
2026.6872	2026.6934	-0.0062	4	1	3	2	6	1	5	3	1	2	2	5	1	4
2026.6872	2026.7247	-0.0375	4	1	3	1	5	1	5	3	1	2	1	4	1	4
2026.6872	2026.7243	-0.0371	4	1	3	1	5	0	5	3	1	2	1	4	0	4
2026.6872	2026.6765	0.0107	4	1	3	2	6	2	5	3	1	2	2	5	2	4
2026.6872	2026.6758	0.0114	4	1	3	1	3	1	4	3	1	2	1	2	1	3
2026.6872	2026.6878	-0.0006	4	1	3	2	5	2	6	3	1	2	2	4	2	5
2026.6872	2026.6720	0.0152	4	1	3	2	5	1	6	3	1	2	2	4	1	5
2026.6872	2026.6869	0.0003	4	1	3	2	6	1	7	3	1	2	2	5	1	6
2026.6872	2026.6990	-0.0118	4	1	3	2	6	2	7	3	1	2	2	5	2	6
2026.6872	2026.6893	-0.0021	4	1	3	1	4	2	6	3	1	2	1	3	2	5
2026.7655	2026.7461	0.0194	4	1	3	0	4	2	6	3	1	2	0	3	2	5
2026.7655	2026.7387	0.0268	4	1	3	2	5	2	5	3	3	0	2	4	0	4
2026.7655	2026.7716	-0.0061	4	1	3	2	4	2	6	3	1	2	2	3	2	5
2026.7655	2026.7428	0.0227	4	1	3	1	4	1	5	3	1	2	1	3	1	4
2026.7655	2026.7590	0.0065	4	1	3	1	4	2	5	3	1	2	1	3	2	4
2026.7655	2026.7530	0.0125	4	1	3	2	6	2	6	3	1	2	2	5	2	5
2026.7655	2026.7317	0.0338	4	1	3	1	5	2	7	3	1	2	1	4	2	6

2026.7655	2026.7370	0.0285	4	1	3	0	4	1	4	3	1	2	0	3	1	3
2428.3000	2428.3059	-0.0059	5	1	5	0	5	2	3	4	1	4	0	4	2	2
2428.3000	2428.2918	0.0082	5	1	5	0	5	1	5	4	1	4	0	4	1	4
2428.3000	2428.3021	-0.0021	5	1	5	2	5	0	5	4	1	4	2	4	0	4
2428.3000	2428.3056	-0.0056	5	1	5	2	6	2	7	4	1	4	2	5	2	6
2428.3000	2428.2866	0.0134	5	1	5	2	5	2	7	4	1	4	0	4	2	6
2428.3250	2428.3421	-0.0171	5	1	5	1	6	0	6	4	1	4	1	5	0	5
2428.3250	2428.3260	-0.0010	5	1	5	1	4	2	6	4	1	4	1	3	2	5
2428.3250	2428.3205	0.0045	5	1	5	1	6	2	7	4	1	4	1	5	2	6
2428.3250	2428.3126	0.0124	5	1	5	1	6	2	8	4	1	4	1	5	2	7
2428.3250	2428.3350	-0.0100	5	1	5	2	6	2	8	4	1	4	2	5	2	7
2428.3250	2428.3233	0.0017	5	1	5	1	6	1	5	4	1	4	1	5	1	4
2428.3250	2428.3218	0.0032	5	1	5	2	5	1	5	4	1	4	2	4	1	4
2428.3250	2428.3252	-0.0002	5	1	5	1	6	1	6	4	1	4	1	5	1	5
2428.3250	2428.3333	-0.0083	5	1	5	2	5	2	6	4	1	4	2	4	2	5
2428.3250	2428.3443	-0.0193	5	1	5	2	5	1	6	4	1	4	2	4	1	5
2428.3750	2428.3620	0.0130	5	1	5	2	6	1	6	4	1	4	2	5	1	5
2428.3750	2428.3733	0.0017	5	1	5	2	6	0	6	4	1	4	2	5	0	5
2428.3750	2428.3658	0.0092	5	1	5	0	5	2	6	4	1	4	0	4	2	5
2428.3750	2428.3643	0.0107	5	1	5	2	7	1	7	4	1	4	2	6	1	6
2428.3750	2428.3526	0.0224	5	1	5	2	7	2	9	4	1	4	2	6	2	8
2428.3750	2428.3756	-0.0006	5	1	5	1	6	1	7	4	1	4	1	5	1	6
2428.3750	2428.3568	0.0182	5	1	5	1	5	2	7	4	1	4	1	4	2	6
2428.3750	2428.3562	0.0188	5	1	5	0	5	2	7	4	1	4	2	4	2	6
2428.4129	2428.4113	0.0016	5	1	5	1	6	2	5	4	1	4	1	5	2	4
2428.4129	2428.4014	0.0115	5	1	5	1	5	1	6	4	1	4	1	4	1	5
2428.4129	2428.4126	0.0003	5	1	5	2	6	2	6	4	1	4	2	6	2	5
2428.4129	2428.4008	0.0121	5	1	5	0	5	1	6	4	3	2	2	4	1	5
2428.4129	2428.4096	0.0033	5	1	5	2	6	1	5	4	1	4	2	5	1	4
2428.4129	2428.4189	-0.0060	5	1	5	2	7	0	7	4	1	4	2	6	0	6
2428.4129	2428.4152	-0.0023	5	1	5	1	6	2	6	4	1	4	1	5	2	5
2428.4129	2428.4156	-0.0027	5	1	5	2	7	1	8	4	1	4	2	6	1	7
2428.4129	2428.3948	0.0181	5	1	5	2	7	2	8	4	1	4	2	6	2	7
2428.4129	2428.4146	-0.0017	5	1	5	2	6	1	7	4	1	4	2	5	1	6
2531.1736	2531.1666	0.0070	5	1	4	2	7	2	9	4	1	3	2	6	2	8
2531.1736	2531.1775	-0.0039	5	1	4	1	4	1	5	4	1	3	1	3	1	4
2531.1736	2531.1614	0.0122	5	1	4	2	7	2	5	4	1	3	2	6	2	4
2531.1736	2531.1632	0.0104	5	1	4	2	6	1	5	4	1	3	2	5	1	4
2531.1736	2531.1807	-0.0071	5	1	4	2	5	1	5	4	1	3	2	4	1	4
2531.1736	2531.1638	0.0098	5	1	4	2	7	1	7	4	1	3	2	6	1	6
2531.1736	2531.1641	0.0095	5	1	4	2	7	0	7	4	1	3	2	6	0	6
2531.1736	2531.1597	0.0139	5	1	4	2	6	1	7	4	1	3	2	5	1	6
2531.1736	2531.1848	-0.0112	5	1	4	2	5	2	7	4	1	3	2	4	2	6
2531.1736	2531.1675	0.0061	5	1	4	2	6	2	7	4	1	3	2	5	2	6
2531.2050	2531.2070	-0.0020	5	1	4	1	6	0	6	4	1	3	1	5	0	5
2531.2050	2531.2035	0.0015	5	1	4	2	7	2	6	4	1	3	2	6	2	5
2531.2050	2531.2272	-0.0222	5	1	4	2	5	1	6	4	1	3	2	4	1	5
2531.2050	2531.2059	-0.0009	5	1	4	2	7	1	8	4	1	3	2	6	1	7
2531.2050	2531.2108	-0.0058	5	1	4	1	5	2	7	4	1	3	1	4	2	6
2531.2050	2531.2081	-0.0031	5	1	4	2	7	1	6	4	1	3	2	6	1	5
2531.2050	2531.2086	-0.0036	5	1	4	1	6	2	8	4	1	3	1	5	2	7
2531.2050	2531.2107	-0.0057	5	1	4	2	7	2	8	4	1	3	2	6	2	7
2531.2050	2531.2064	-0.0014	5	1	4	1	6	1	6	4	1	3	1	5	1	5

2531.2050	2531.2099	-0.0049	5	3	2	2	6	0	6	4	1	3	2	5	2	5
2531.2407	2531.2528	-0.0121	5	1	4	1	6	2	7	4	1	3	1	5	2	6
2531.2407	2531.2524	-0.0117	5	1	4	1	5	1	6	4	1	3	1	4	1	5
2531.2407	2531.2396	0.0011	5	1	4	0	5	2	7	4	1	3	0	4	2	6
2531.2407	2531.2497	-0.0090	5	1	4	1	6	1	5	4	1	3	1	5	1	4
2531.2407	2531.2366	0.0041	5	1	4	0	5	1	5	4	1	3	0	4	1	4
2531.2407	2531.2579	-0.0172	5	1	4	1	5	2	6	4	1	3	1	4	2	5
2531.2407	2531.2360	0.0047	5	3	2	0	5	2	6	4	1	3	2	4	2	5
2531.2407	2531.2480	-0.0073	5	1	4	1	6	1	7	4	1	3	1	5	1	6
2531.2407	2531.2377	0.0030	5	1	4	0	5	0	5	4	1	3	0	4	0	4
2531.2407	2531.2502	-0.0095	5	1	4	2	7	2	7	4	1	3	2	6	2	6
2976.4200	2976.4940	-0.0740	6	2	5	2	8	2	10	5	2	4	2	7	2	9
2976.4200	2976.4951	-0.0751	6	2	5	1	6	2	8	5	2	4	1	5	2	7
2976.4200	2976.4903	-0.0703	6	2	5	2	8	1	8	5	2	4	2	7	1	7
2976.4200	2976.4948	-0.0748	6	2	5	0	6	2	8	5	2	4	0	5	2	7
2976.4200	2976.4934	-0.0734	6	2	5	2	8	0	8	5	2	4	2	7	0	7
2976.4200	2976.4916	-0.0716	6	2	5	1	6	1	6	5	2	4	1	5	1	5
2976.4200	2976.4935	-0.0735	6	2	5	0	6	1	6	5	2	4	0	5	1	5
2976.4200	2976.4931	-0.0731	6	2	5	1	6	0	6	5	2	4	1	5	0	5
2976.4200	2976.4967	-0.0767	6	2	5	2	4	2	6	5	2	4	2	4	2	5
2976.5007	2976.5626	-0.0619	6	2	5	2	8	1	9	5	2	4	2	7	1	8
2976.5007	2976.5223	-0.0216	6	2	5	1	7	2	9	5	2	4	1	6	2	8
2976.5007	2976.5310	-0.0303	6	2	5	2	7	2	9	5	2	4	2	6	2	8
2976.5007	2976.5546	-0.0539	6	2	5	2	8	2	9	5	2	4	2	7	2	8
2976.5007	2976.5575	-0.0568	6	2	5	2	6	2	8	5	2	4	2	5	2	7
2976.5007	2976.5272	-0.0265	6	2	5	1	5	2	7	5	2	4	1	4	2	6
2976.5007	2976.5576	-0.0569	6	2	5	1	6	1	7	5	2	4	1	5	1	6
2976.5007	2976.5556	-0.0549	6	2	5	0	6	1	7	5	2	4	2	5	1	6
2976.5007	2976.5261	-0.0254	6	2	5	1	7	0	7	5	2	4	1	6	0	6
2976.5007	2976.5268	-0.0261	6	2	5	1	7	1	7	5	2	4	1	6	1	6
2976.5761	2976.5909	-0.0148	6	2	5	1	7	1	8	5	2	4	1	6	1	7
2976.5761	2976.5874	-0.0113	6	2	5	2	7	1	8	5	2	4	2	6	1	7
2976.5761	2976.5972	-0.0211	6	2	5	1	7	2	8	5	2	4	1	6	2	7
2976.5761	2976.6110	-0.0349	6	2	5	2	8	2	8	5	2	4	2	7	2	7
2976.5761	2976.6124	-0.0363	6	2	5	2	7	2	8	5	2	4	2	6	2	7
2976.5761	2976.6177	-0.0416	6	2	5	2	6	1	7	5	2	4	0	5	1	6
2976.5761	2976.5883	-0.0122	6	2	5	1	5	1	6	5	2	4	1	4	1	5
2976.5761	2976.5919	-0.0158	6	2	5	1	7	1	6	5	2	4	1	6	1	5
2976.5761	2976.5748	0.0013	6	2	5	2	6	1	6	5	2	4	2	5	1	5
2976.5761	2976.6104	-0.0343	6	2	5	1	6	2	6	5	2	4	1	5	2	5
3010.4375	3010.4421	-0.0046	6	2	4	1	6	2	4	5	2	3	1	5	2	3
3010.4375	3010.4433	-0.0058	6	2	4	0	6	0	6	5	2	3	0	5	0	5
3010.4375	3010.4420	-0.0045	6	2	4	0	6	2	8	5	2	3	0	5	2	7
3010.4375	3010.4395	-0.0020	6	2	4	1	6	1	6	5	2	3	1	5	1	5
3010.4375	3010.4422	-0.0047	6	2	4	0	6	1	6	5	2	3	0	5	1	5
3010.4375	3010.4444	-0.0069	6	2	4	0	6	2	4	5	2	3	0	5	2	3
3010.4375	3010.4350	0.0025	6	2	4	2	4	0	4	5	2	3	2	3	0	3
3010.4375	3010.4409	-0.0034	6	2	4	1	6	0	6	5	2	3	1	5	0	5
3010.4375	3010.4392	-0.0017	6	2	4	1	6	2	8	5	2	3	1	5	2	7
3010.4648	3010.4528	0.0120	6	2	4	2	8	0	8	5	2	3	2	7	0	7
3010.4648	3010.4522	0.0126	6	2	4	2	8	2	10	5	2	3	2	7	2	9
3010.4648	3010.4523	0.0125	6	2	4	2	8	1	8	5	2	3	2	7	1	7
3010.4648	3010.4564	0.0084	6	2	4	2	4	1	3	5	2	3	2	3	1	2

3010.4648	3010.4533	0.0115	6	2	4	2	8	2	6	5	2	3	2	7	2	5
3010.4878	3010.4640	0.0238	6	2	4	2	4	1	5	5	2	3	2	3	1	4
3010.4878	3010.4657	0.0221	6	2	4	2	4	2	5	5	2	3	2	3	2	4
3010.4878	3010.4673	0.0205	6	2	4	1	6	1	7	5	2	3	1	5	1	6
3010.4878	3010.4700	0.0178	6	2	4	0	6	1	7	5	2	3	0	5	1	6
3010.4878	3010.4692	0.0186	6	2	4	1	6	2	7	5	2	3	1	5	2	6
3010.4878	3010.4722	0.0156	6	2	4	0	6	2	7	5	2	3	0	5	2	6
3010.4878	3010.4664	0.0214	6	2	4	1	6	1	5	5	2	3	1	5	1	4
3010.4878	3010.4694	0.0184	6	2	4	0	6	1	5	5	2	3	0	5	1	4
3010.6375	3010.6400	-0.0025	6	2	4	1	7	0	7	5	2	3	1	6	0	6
3010.6375	3010.6408	-0.0033	6	2	4	1	7	2	5	5	2	3	1	6	2	4
3010.6375	3010.6400	-0.0025	6	2	4	1	7	2	9	5	2	3	1	6	2	8
3010.6375	3010.6396	-0.0021	6	2	4	1	7	1	7	5	2	3	1	6	1	6
3010.6612	3010.6631	-0.0019	6	2	4	2	5	1	4	5	2	3	2	4	1	3
3010.6612	3010.6651	-0.0039	6	2	4	2	5	1	6	5	2	3	2	4	1	5
3010.6612	3010.6669	-0.0057	6	4	2	2	5	2	6	5	4	1	2	4	2	5
3010.6612	3010.6643	-0.0031	6	2	4	1	7	2	8	5	2	3	1	6	2	7
3010.6612	3010.6639	-0.0027	6	2	4	1	7	1	6	5	2	3	1	6	1	5
3010.6612	3010.6646	-0.0034	6	2	4	1	7	2	6	5	2	3	1	6	2	5
3010.6612	3010.6654	-0.0042	6	2	4	1	7	1	8	5	2	3	1	6	1	7
3010.6875	3010.6908	-0.0033	6	2	4	1	7	2	7	5	2	3	1	6	2	6
3010.6875	3010.6921	-0.0046	6	2	4	2	7	1	6	5	2	3	2	6	1	5
3010.6875	3010.6904	-0.0029	6	2	4	2	7	2	6	5	2	3	2	6	2	5
3010.6875	3010.6895	-0.0020	6	2	4	2	7	1	8	5	2	3	2	6	1	7
3010.6875	3010.6932	-0.0057	6	2	4	2	7	2	8	5	2	3	2	6	2	7
3010.8499	3010.8464	0.0035	6	2	4	2	6	1	6	5	2	3	2	5	1	5
3010.8499	3010.8485	0.0014	6	2	4	2	6	2	4	5	2	3	2	5	2	3
3010.8499	3010.8468	0.0031	6	2	4	2	6	2	8	5	2	3	2	5	2	7
3010.8499	3010.8475	0.0024	6	2	4	2	6	0	6	5	2	3	2	5	0	5
3010.9000	3010.8784	0.0216	6	2	4	2	6	2	7	5	2	3	2	5	2	6
3010.9000	3010.8791	0.0209	6	2	4	2	6	1	5	5	2	3	2	5	1	4
3010.9000	3010.8796	0.0204	6	2	4	2	6	1	7	5	2	3	2	5	1	6
3010.9000	3010.8770	0.0230	6	4	2	0	6	2	5	5	0	5	2	5	2	4
3034.0249	3034.0153	0.0096	6	1	5	1	5	1	6	5	1	4	1	4	1	5
3034.0249	3034.0130	0.0119	6	1	5	2	6	1	7	5	3	2	0	5	1	6
3034.0249	3034.0169	0.0080	6	1	5	2	6	2	7	5	1	4	2	5	2	6
3034.0249	3034.0308	-0.0059	6	1	5	2	8	2	10	5	1	4	2	7	2	9
3034.0249	3034.0336	-0.0087	6	1	5	1	7	2	9	5	1	4	1	6	2	8
3034.0249	3034.0294	-0.0045	6	1	5	2	8	1	8	5	1	4	2	7	1	7
3034.0249	3034.0297	-0.0048	6	1	5	2	8	0	8	5	1	4	2	7	0	7
3034.0249	3034.0328	-0.0079	6	1	5	1	7	1	7	5	1	4	1	6	1	6
3034.0249	3034.0334	-0.0085	6	1	5	1	7	0	7	5	1	4	1	6	0	6
3034.0249	3034.0354	-0.0105	6	3	3	2	7	0	7	5	3	2	2	6	0	6
3034.0625	3034.0644	-0.0019	6	1	5	2	8	1	9	5	1	4	2	7	1	8
3034.0625	3034.0690	-0.0065	6	1	5	1	7	2	8	5	1	4	1	6	2	7
3034.0625	3034.0652	-0.0027	6	1	5	2	8	1	7	5	1	4	2	7	1	6
3034.0625	3034.0639	-0.0014	6	1	5	2	8	2	7	5	1	4	2	7	2	6
3034.0625	3034.0675	-0.0050	6	1	5	1	7	1	6	5	1	4	1	6	1	5
3034.0625	3034.0646	-0.0021	6	1	5	1	6	1	6	5	1	4	1	5	1	5
3034.0625	3034.0666	-0.0041	6	1	5	2	8	2	9	5	1	4	2	7	2	8
3034.0625	3034.0814	-0.0189	6	1	5	0	6	2	8	5	1	4	0	5	2	7
3034.0625	3034.0657	-0.0032	6	1	5	1	6	2	8	5	1	4	1	5	2	7
3034.0625	3034.0673	-0.0048	6	1	5	1	7	1	8	5	1	4	1	6	1	7

3034.0875	3034.1176	-0.0301	6	1	5	0	6	1	7	5	1	4	0	5	1	6
3034.0875	3034.1106	-0.0231	6	1	5	2	5	2	7	5	1	4	2	4	2	6
3034.0875	3034.1006	-0.0131	6	1	5	2	8	2	8	5	1	4	2	7	2	7
3034.0875	3034.1036	-0.0161	6	1	5	1	6	2	7	5	1	4	1	5	2	6
3034.0875	3034.1195	-0.0320	6	1	5	0	6	2	7	5	1	4	0	5	2	6
3034.0875	3034.1035	-0.0160	6	1	5	1	7	2	7	5	1	4	1	6	2	6
3034.0875	3034.1017	-0.0142	6	1	5	1	6	1	7	5	1	4	1	5	1	6
3034.0875	3034.1017	-0.0142	6	1	5	1	6	1	5	5	1	4	1	5	1	4
3034.0875	3034.1176	-0.0301	6	1	5	0	6	1	5	5	1	4	0	5	1	4
3034.0875	3034.1298	-0.0423	6	1	5	2	4	2	6	5	1	4	2	3	2	5
3393.7959	3393.7748	0.0211	7	1	7	2	6	1	6	6	1	6	2	5	1	5
3393.7959	3393.7746	0.0213	7	1	7	2	7	2	9	6	1	6	2	6	2	8
3393.7959	3393.7648	0.0311	7	3	5	2	7	2	8	6	3	4	2	6	2	7
3393.7959	3393.7819	0.0140	7	1	7	2	7	1	7	6	1	6	2	6	1	6
3393.7959	3393.7864	0.0095	7	1	7	2	7	0	7	6	1	6	2	6	0	6
3393.7959	3393.7956	0.0003	7	3	5	2	6	2	7	6	3	4	2	5	2	6
3393.7959	3393.7938	0.0021	7	1	7	2	6	0	6	6	1	6	2	5	0	5
3393.7959	3393.7958	0.0001	7	1	7	2	7	2	5	6	1	6	2	6	2	4
3393.7959	3393.8088	-0.0129	7	1	7	2	6	1	5	6	1	6	2	5	1	4
3393.8375	3393.8274	0.0101	7	1	7	1	8	1	8	6	1	6	1	7	1	7
3393.8375	3393.8193	0.0182	7	1	7	2	7	2	8	6	1	6	2	6	2	7
3393.8375	3393.8305	0.0070	7	1	7	1	8	2	9	6	1	6	1	7	2	8
3393.8375	3393.8315	0.0060	7	1	7	2	8	2	9	6	1	6	2	7	2	8
3393.8375	3393.8241	0.0134	7	1	7	1	6	2	8	6	1	6	1	5	2	7
3393.8375	3393.8147	0.0228	7	1	7	2	7	1	8	6	1	6	2	6	1	7
3393.8375	3393.8181	0.0194	7	1	7	1	8	2	10	6	1	6	1	7	2	9
3393.8375	3393.8271	0.0104	7	1	7	2	8	2	10	6	1	6	2	7	2	9
3393.8750	3393.8993	-0.0243	7	1	7	0	7	1	8	6	1	6	0	6	1	7
3393.8750	3393.8903	-0.0153	7	1	7	2	9	2	10	6	1	6	2	8	2	9
3393.8750	3393.8920	-0.0170	7	1	7	2	9	0	9	6	1	6	2	8	0	8
3393.8750	3393.8875	-0.0125	7	1	7	2	8	1	9	6	1	6	2	7	1	8
3393.8750	3393.9011	-0.0261	7	1	7	1	7	1	8	6	1	6	1	6	1	7
3393.8750	3393.8623	0.0127	7	1	7	2	9	1	9	6	1	6	2	8	1	8
3393.8750	3393.8656	0.0094	7	1	7	0	7	2	9	6	1	6	0	6	2	8
3393.8750	3393.8655	0.0095	7	1	7	1	7	2	9	6	1	6	1	6	2	8
3393.8750	3393.8677	0.0073	7	1	7	2	9	2	11	6	1	6	2	8	2	10
3393.8750	3393.8573	0.0177	7	1	7	1	8	1	9	6	1	6	1	7	1	8
3393.9250	3393.9234	0.0016	7	1	7	2	8	2	7	6	1	6	2	7	2	6
3393.9250	3393.9069	0.0181	7	1	7	2	9	1	10	6	1	6	2	8	1	9
3393.9250	3393.9500	-0.0250	7	1	7	2	9	2	8	6	1	6	2	8	2	7
3393.9250	3393.9298	-0.0048	7	1	7	1	7	2	7	6	1	6	1	6	2	6
3393.9250	3393.9262	-0.0012	7	3	5	0	7	0	7	6	1	6	0	6	2	6
3393.9250	3393.9175	0.0075	7	1	7	1	6	2	5	6	1	6	1	5	2	4
3393.9250	3393.9455	-0.0205	7	1	7	1	7	2	6	6	1	6	1	6	2	5
3393.9250	3393.9356	-0.0106	7	1	7	0	7	2	6	6	1	6	0	6	2	5
3393.9250	3393.9207	0.0043	7	3	5	2	5	2	5	6	1	6	2	4	0	4
3393.9250	3393.9338	-0.0088	7	1	7	2	9	2	9	6	1	6	2	8	2	8
3469.9875	3469.9917	-0.0042	7	2	6	1	7	2	9	6	2	5	1	6	2	8
3469.9875	3469.9919	-0.0044	7	2	6	0	7	2	9	6	2	5	0	6	2	8
3469.9875	3469.9898	-0.0023	7	2	6	2	9	0	9	6	2	5	2	8	0	8
3469.9875	3469.9880	-0.0005	7	2	6	2	9	2	11	6	2	5	2	8	2	10
3469.9875	3469.9914	-0.0039	7	2	6	1	8	2	10	6	2	5	1	7	2	9
3469.9875	3469.9957	-0.0082	7	2	6	2	8	2	10	6	2	5	2	7	2	9

3469.9875	3469.9982	-0.0107	7	2	6	1	8	0	8	6	2	5	1	7	0	7
3469.9875	3469.9873	0.0002	7	2	6	2	9	1	9	6	2	5	2	8	1	8
3469.9875	3469.9960	-0.0085	7	2	6	2	7	2	9	6	2	5	2	6	2	8
3469.9875	3469.9936	-0.0061	7	2	6	1	6	2	8	6	2	5	1	5	2	7
3470.0125	3470.0359	-0.0234	7	2	6	0	7	1	8	6	2	5	0	6	1	7
3470.0125	3470.0549	-0.0424	7	2	6	2	8	2	9	6	2	5	2	8	2	8
3470.0125	3470.0430	-0.0305	7	2	6	1	8	1	9	6	2	5	1	7	1	8
3470.0125	3470.0334	-0.0209	7	2	6	2	8	1	9	6	2	5	2	7	1	8
3470.0125	3470.0388	-0.0263	7	2	6	2	7	1	8	6	2	5	2	6	1	7
3470.0125	3470.0430	-0.0305	7	2	6	1	8	2	9	6	2	5	1	7	2	8
3470.0125	3470.0382	-0.0257	7	2	6	1	7	1	8	6	2	5	1	6	1	7
3470.0125	3470.0395	-0.0270	7	2	6	2	9	1	10	6	2	5	2	8	1	9
3470.0125	3470.0236	-0.0111	7	2	6	2	9	1	8	6	2	5	2	8	1	7
3470.0125	3470.0234	-0.0109	7	2	6	2	9	2	10	6	2	5	2	8	2	9
3470.1000	3470.0834	0.0166	7	2	6	2	5	2	5	6	2	5	2	4	2	4
3470.1000	3470.0903	0.0097	7	2	6	1	6	2	6	6	2	5	1	5	2	5
3470.1000	3470.0831	0.0169	7	2	6	2	6	2	6	6	2	5	2	6	2	5
3470.1000	3470.0777	0.0223	7	2	6	2	8	2	8	6	4	3	2	7	2	7
3470.1000	3470.0610	0.0390	7	2	6	2	9	2	9	6	2	5	2	7	2	8
3470.1000	3470.0958	0.0042	7	2	6	2	7	2	7	6	2	5	2	6	2	6
3470.1000	3470.0726	0.0274	7	2	6	0	7	2	7	6	2	5	0	6	2	6
3470.1000	3470.0760	0.0240	7	2	6	2	7	2	6	6	4	3	0	6	2	5
3470.1000	3470.0961	0.0039	7	2	6	1	8	2	8	6	2	5	1	7	2	7
3470.1000	3470.0699	0.0301	7	2	6	1	7	2	7	6	2	5	1	6	2	6
3484.7752	3484.7704	0.0048	7	3	5	2	9	2	7	6	3	4	2	8	2	6
3484.7752	3484.7702	0.0050	7	3	5	0	7	1	7	6	3	4	0	6	1	6
3484.7752	3484.7698	0.0054	7	3	5	1	7	0	7	6	3	4	1	6	0	6
3484.7752	3484.7703	0.0049	7	3	5	1	7	1	7	6	3	4	1	6	1	6
3484.7752	3484.7777	-0.0025	7	3	5	2	9	0	9	6	3	4	2	8	0	8
3484.7752	3484.7776	-0.0024	7	3	5	1	7	2	9	6	3	4	1	6	2	8
3484.7752	3484.7773	-0.0021	7	3	5	0	7	2	9	6	3	4	0	6	2	8
3484.7752	3484.7848	-0.0096	7	3	5	2	9	2	11	6	3	4	2	8	2	10
3484.7752	3484.7781	-0.0029	7	3	5	2	9	1	9	6	3	4	2	8	1	8
3484.7752	3484.7699	0.0053	7	3	5	0	7	0	7	6	5	2	0	6	0	6
3488.8499	3488.8493	0.0006	7	3	4	0	7	1	7	6	3	3	0	6	1	6
3488.8499	3488.8555	-0.0056	7	3	4	0	7	2	9	6	3	3	0	6	2	8
3488.8499	3488.8545	-0.0046	7	3	4	2	9	2	7	6	3	3	2	8	2	6
3488.8499	3488.8494	0.0005	7	3	4	1	7	0	7	6	3	3	1	6	0	6
3488.8499	3488.8488	0.0011	7	3	4	0	7	0	7	6	3	3	0	6	0	6
3488.8499	3488.8498	0.0001	7	3	4	1	7	1	7	6	3	3	1	6	1	6
3488.8499	3488.8561	-0.0062	7	3	4	1	7	2	9	6	3	3	1	6	2	8
3488.8499	3488.8611	-0.0112	7	3	4	2	9	1	9	6	3	3	2	8	1	8
3488.8499	3488.8607	-0.0108	7	3	4	2	9	0	9	6	3	3	2	8	0	8
3488.8499	3488.8668	-0.0169	7	3	4	2	9	2	11	6	3	3	2	8	2	10
3521.3408	3521.3746	-0.0338	7	2	5	2	9	1	9	6	2	4	2	8	1	8
3521.3408	3521.3725	-0.0317	7	2	5	2	9	2	11	6	2	4	2	8	2	10
3521.3408	3521.3797	-0.0389	7	2	5	2	9	1	10	6	2	4	2	8	1	9
3521.3408	3521.3774	-0.0366	7	2	5	2	9	2	10	6	2	4	2	8	2	9
3521.3408	3521.3781	-0.0373	7	2	5	2	9	1	8	6	2	4	2	8	1	7
3521.3408	3521.3753	-0.0345	7	2	5	1	7	1	8	6	2	4	1	6	1	7
3521.3408	3521.3652	-0.0244	7	2	5	1	7	2	9	6	2	4	1	6	2	8
3521.3408	3521.3678	-0.0270	7	2	5	0	7	2	9	6	2	4	0	6	2	8
3521.3408	3521.3846	-0.0438	7	2	5	2	9	2	9	6	2	4	2	8	2	8

3521.3408	3521.3752	-0.0344	7	2	5	2	9	0	9	6	2	4	2	8	0	8
3521.5192	3521.5541	-0.0349	7	2	5	2	8	2	9	6	2	4	2	7	2	8
3521.5192	3521.5403	-0.0211	7	2	5	1	8	1	8	6	2	4	1	7	1	7
3521.5192	3521.5409	-0.0217	7	2	5	1	8	0	8	6	2	4	1	7	0	7
3521.5192	3521.5384	-0.0192	7	2	5	1	8	2	10	6	2	4	1	7	2	9
3521.5192	3521.5429	-0.0237	7	2	5	2	8	2	10	6	2	4	2	7	2	9
3521.5192	3521.5486	-0.0294	7	2	5	1	8	2	8	6	2	4	1	7	2	7
3521.5192	3521.5560	-0.0368	7	2	5	2	8	1	9	6	2	4	2	7	1	8
3521.5192	3521.5457	-0.0265	7	2	5	1	8	1	9	6	2	4	1	7	1	8
3521.5192	3521.5363	-0.0171	7	2	5	1	6	2	8	6	2	4	1	5	2	7
3521.5192	3521.5402	-0.0210	7	2	5	1	8	2	9	6	2	4	1	7	2	8
3874.9625	3874.9578	0.0047	8	3	6	2	8	2	9	7	3	5	2	7	2	8
3874.9625	3874.9658	-0.0033	8	1	8	2	8	0	8	7	1	7	2	7	0	7
3874.9625	3874.9679	-0.0054	8	1	8	2	7	1	7	7	1	7	2	6	1	6
3874.9625	3874.9561	0.0064	8	1	8	2	8	2	10	7	1	7	2	7	2	9
3874.9625	3874.9627	-0.0002	8	1	8	2	8	1	8	7	1	7	2	7	1	7
3874.9625	3874.9743	-0.0118	8	1	8	2	8	2	6	7	1	7	2	7	2	5
3875.0000	3875.0000	-0.0000	8	1	8	2	8	2	9	7	1	7	2	7	2	8
3875.0000	3875.0209	-0.0209	8	1	8	2	9	2	10	7	1	7	2	8	2	9
3875.0000	3875.0028	-0.0028	8	1	8	1	9	2	11	7	1	7	1	8	2	10
3875.0000	3875.0094	-0.0094	8	1	8	2	9	2	11	7	1	7	2	8	2	10
3875.0000	3875.0228	-0.0228	8	1	8	2	9	0	9	7	1	7	2	8	0	8
3875.0000	3875.0362	-0.0362	8	1	8	1	9	1	10	7	1	7	1	8	1	9
3875.0000	3875.0170	-0.0170	8	1	8	1	9	2	10	7	1	7	1	8	2	9
3875.0000	3875.0142	-0.0142	8	1	8	1	9	0	9	7	1	7	1	8	0	8
3875.0000	3875.0106	-0.0106	8	1	8	1	9	1	9	7	1	7	1	8	1	8
3875.0000	3875.0073	-0.0073	8	1	8	1	7	2	9	7	1	7	1	6	2	8
3875.0728	3875.0642	0.0086	8	1	8	2	9	1	10	7	1	7	2	8	1	9
3875.0728	3875.0734	-0.0006	8	1	8	2	10	2	11	7	1	7	2	9	2	10
3875.0728	3875.0507	0.0221	8	1	8	0	8	2	10	7	1	7	0	7	2	9
3875.0728	3875.0772	-0.0044	8	1	8	2	10	1	9	7	1	7	2	9	1	8
3875.0728	3875.0451	0.0277	8	1	8	2	10	1	10	7	1	7	2	9	1	9
3875.0728	3875.0503	0.0225	8	1	8	1	8	2	10	7	1	7	1	7	2	9
3875.0728	3875.0541	0.0187	8	1	8	2	10	2	12	7	1	7	2	9	2	11
3875.0728	3875.0689	0.0039	8	1	8	1	9	2	9	7	1	7	1	8	2	8
3875.0728	3875.0693	0.0035	8	1	8	2	10	0	10	7	1	7	2	9	0	9
3875.0728	3875.0712	0.0016	8	1	8	2	7	2	9	7	1	7	2	8	2	8
3875.1157	3875.0895	0.0262	8	1	8	2	9	2	8	7	1	7	2	8	2	7
3875.1157	3875.1067	0.0090	8	1	8	1	8	0	8	7	1	7	1	7	2	7
3875.1157	3875.1043	0.0114	8	1	8	0	8	2	8	7	3	5	0	7	0	7
3875.1157	3875.1073	0.0084	8	1	8	0	8	2	7	7	1	7	0	7	2	6
3875.1157	3875.1098	0.0059	8	1	8	2	10	2	10	7	1	7	2	9	2	9
3875.1157	3875.1125	0.0032	8	1	8	1	8	2	7	7	1	7	1	7	2	6
3875.1157	3875.1159	-0.0002	8	1	8	2	10	2	9	7	1	7	2	9	2	8
3875.1157	3875.1009	0.0148	8	1	8	2	6	2	5	7	1	7	2	5	2	4
3875.1157	3875.0998	0.0159	8	5	4	2	6	2	6	7	3	5	2	5	2	5
3983.6769	3983.6780	-0.0011	8	3	6	1	8	1	8	7	3	5	1	7	1	7
3983.6769	3983.6787	-0.0018	8	3	6	0	8	0	8	7	3	5	0	7	0	7
3983.6769	3983.6786	-0.0017	8	3	6	0	8	1	8	7	3	5	0	7	1	7
3983.6769	3983.6780	-0.0011	8	3	6	2	10	2	8	7	3	5	2	9	2	7
3983.6769	3983.6837	-0.0068	8	3	6	2	10	2	12	7	3	5	2	9	2	11
3983.6769	3983.6815	-0.0046	8	3	6	0	8	2	10	7	3	5	0	7	2	9
3983.6769	3983.6809	-0.0040	8	3	6	1	8	2	10	7	3	5	1	7	2	9

3983.6769	3983.6782	-0.0013	8	3	6	1	8	0	8	7	3	5	1	7	0	7
3983.6769	3983.6809	-0.0040	8	3	6	2	10	1	10	7	3	5	2	9	1	9
3983.6769	3983.6809	-0.0040	8	3	6	2	10	0	10	7	3	5	2	9	0	9
3983.7625	3983.7368	0.0257	8	3	6	2	10	1	9	7	3	5	2	9	1	8
3983.7625	3983.7506	0.0119	8	3	6	1	9	2	11	7	3	5	1	8	2	10
3983.7625	3983.7348	0.0277	8	3	6	2	10	1	11	7	3	5	2	9	1	10
3983.7625	3983.7386	0.0239	8	3	6	2	10	2	11	7	3	5	2	9	2	10
3983.7625	3983.7483	0.0142	8	3	6	1	9	0	9	7	3	5	1	8	0	8
3983.7625	3983.7483	0.0142	8	3	6	1	9	1	9	7	3	5	1	8	1	8
3983.7625	3983.7325	0.0300	8	3	6	0	8	1	9	7	3	5	0	7	1	8
3983.7625	3983.7319	0.0306	8	3	6	1	8	1	9	7	3	5	1	7	1	8
3983.7625	3983.7371	0.0254	8	3	6	0	8	2	9	7	3	5	0	7	2	8
3983.7625	3983.7573	0.0052	8	3	6	2	9	2	11	7	3	5	2	8	2	10
3983.8058	3983.8044	0.0014	8	3	6	1	9	1	8	7	3	5	1	8	1	7
3983.8058	3983.7901	0.0157	8	3	6	2	10	2	10	7	3	5	2	9	2	9
3983.8058	3983.8073	-0.0015	8	3	6	1	9	2	10	7	3	5	1	8	2	9
3983.8058	3983.7999	0.0059	8	3	6	1	9	2	8	7	3	5	1	8	2	7
3983.8058	3983.8118	-0.0060	8	3	6	2	9	2	10	7	3	5	2	8	2	9
3983.8058	3983.8038	0.0020	8	3	6	1	7	1	8	7	3	5	1	6	1	7
3983.8058	3983.8089	-0.0031	8	3	6	2	9	1	8	7	3	5	2	8	1	7
3983.8058	3983.8018	0.0040	8	3	6	1	9	1	10	7	3	5	1	8	1	9
3983.8058	3983.8070	-0.0012	8	3	6	2	9	1	10	7	3	5	2	8	1	9
3983.8058	3983.8256	-0.0198	8	3	6	2	8	2	10	7	3	5	2	7	2	9
3991.7250	3991.7092	0.0158	8	3	5	1	8	0	8	7	3	4	1	7	0	7
3991.7250	3991.7090	0.0160	8	3	5	0	8	1	8	7	3	4	0	7	1	7
3991.7250	3991.7163	0.0087	8	3	5	2	10	1	10	7	3	4	2	9	1	9
3991.7250	3991.7089	0.0161	8	3	5	1	8	1	8	7	3	4	1	7	1	7
3991.7250	3991.7148	0.0102	8	3	5	2	10	2	8	7	3	4	2	9	2	7
3991.7250	3991.7092	0.0158	8	3	5	0	8	0	8	7	3	4	0	7	0	7
3991.7250	3991.7164	0.0086	8	3	5	2	10	0	10	7	3	4	2	9	0	9
3991.7250	3991.7105	0.0145	8	3	5	1	8	2	10	7	3	4	1	7	2	9
3991.7250	3991.7108	0.0142	8	3	5	0	8	2	10	7	3	4	0	7	2	9
3991.7250	3991.7180	0.0070	8	3	5	2	10	2	12	7	3	4	2	9	2	11
3991.7532	3991.7594	-0.0062	8	3	5	2	10	1	9	7	3	4	2	9	1	8
3991.7532	3991.7570	-0.0038	8	3	5	2	10	1	11	7	3	4	2	9	1	10
3991.7532	3991.7562	-0.0030	8	3	5	0	8	2	9	7	3	4	0	7	2	8
3991.7532	3991.7618	-0.0086	8	3	5	2	10	2	11	7	3	4	2	9	2	10
3991.7532	3991.7526	0.0006	8	3	5	0	8	1	7	7	3	4	0	7	1	6
3991.7532	3991.7505	0.0027	8	3	5	0	8	1	9	7	3	4	0	7	1	8
3991.7532	3991.7558	-0.0026	8	3	5	1	8	2	9	7	3	4	1	7	2	8
3991.7532	3991.7522	0.0010	8	3	5	1	8	1	7	7	3	4	1	7	1	6
3991.7532	3991.7504	0.0028	8	3	5	1	8	1	9	7	3	4	1	7	1	8
3991.7532	3991.7564	-0.0032	8	3	5	2	10	2	9	7	3	4	2	9	2	8
4032.5000	4032.5081	-0.0081	8	1	7	2	8	2	10	7	1	6	2	7	2	9
4032.5000	4032.5438	-0.0438	8	1	7	2	8	1	9	7	1	6	2	7	1	8
4032.5000	4032.5462	-0.0462	8	1	7	2	8	2	9	7	1	6	2	7	2	8
4032.5000	4032.5076	-0.0076	8	1	7	2	8	0	8	7	1	6	2	7	0	7
4032.5000	4032.5071	-0.0071	8	1	7	2	8	1	8	7	1	6	2	7	1	7
4032.5000	4032.5441	-0.0441	8	1	7	2	8	1	7	7	1	6	2	7	1	6
4032.5000	4032.5453	-0.0453	8	3	5	0	8	2	7	7	3	4	0	7	2	6
4032.5000	4032.5072	-0.0072	8	1	7	2	8	2	6	7	1	6	2	7	2	5
4032.6125	4032.6121	0.0004	8	1	7	1	9	2	11	7	1	6	1	8	2	10
4032.6125	4032.5937	0.0188	8	1	7	2	9	1	10	7	1	6	2	8	1	9

4032.6125	4032.5956	0.0169	8	1	7	2	9	2	10	7	1	6	2	8	2	9
4032.6125	4032.6117	0.0008	8	1	7	1	9	1	9	7	1	6	1	8	1	8
4032.6125	4032.6121	0.0004	8	1	7	1	9	0	9	7	1	6	1	8	0	8
4032.6125	4032.5779	0.0346	8	1	7	1	7	2	9	7	1	6	1	6	2	8
4032.6125	4032.6317	-0.0192	8	3	5	2	9	0	9	7	3	4	2	8	0	8
4032.6125	4032.6133	-0.0008	8	1	7	1	7	1	8	7	1	6	1	6	1	7
4032.6125	4032.5940	0.0185	8	1	7	2	9	1	8	7	1	6	2	8	1	7
4032.6125	4032.6133	-0.0008	8	1	7	1	7	2	8	7	1	6	1	6	2	7
4032.6750	4032.6460	0.0290	8	1	7	1	9	1	10	7	1	6	1	8	1	9
4032.6750	4032.6472	0.0278	8	1	7	1	9	2	10	7	1	6	1	8	2	9
4032.6750	4032.6816	-0.0066	8	1	7	1	9	2	9	7	1	6	1	8	2	8
4032.6750	4032.6558	0.0192	8	1	7	2	7	2	9	7	1	6	2	6	2	8
4032.6750	4032.6462	0.0288	8	1	7	1	9	1	8	7	1	6	1	8	1	7
4032.6987	4032.6686	0.0301	8	1	7	2	10	2	12	7	1	6	2	9	2	11
4032.6987	4032.7026	-0.0039	8	1	7	2	10	1	11	7	1	6	2	9	1	10
4032.6987	4032.7036	-0.0049	8	1	7	2	10	2	11	7	1	6	2	9	2	10
4032.6987	4032.6677	0.0310	8	1	7	2	10	1	10	7	1	6	2	9	1	9
4032.6987	4032.6680	0.0307	8	1	7	2	10	0	10	7	1	6	2	9	0	9
4032.6987	4032.6942	0.0045	8	1	7	1	8	2	10	7	1	6	1	7	2	9
4032.6987	4032.7010	-0.0023	8	1	7	0	8	2	10	7	1	6	0	7	2	9
4032.6987	4032.7379	-0.0392	8	1	7	2	10	2	10	7	1	6	2	9	2	9
4032.6987	4032.7028	-0.0041	8	1	7	2	10	1	9	7	1	6	2	9	1	8
4032.6987	4032.7294	-0.0307	8	1	7	1	8	1	9	7	1	6	1	7	1	8
4032.7250	4032.7363	-0.0113	8	1	7	0	8	1	9	7	1	6	0	7	1	8
4032.7250	4032.7021	0.0229	8	1	7	2	10	2	9	7	1	6	2	9	2	8
4032.7250	4032.7368	-0.0118	8	1	7	0	8	2	9	7	1	6	0	7	2	8
4033.7821	4033.7839	-0.0018	8	2	6	2	10	1	11	7	2	5	2	9	1	10
4033.7821	4033.7842	-0.0021	8	2	6	2	10	2	12	7	2	5	2	9	2	11
4033.7821	4033.7806	0.0015	8	2	6	2	10	2	11	7	2	5	2	9	2	10
4033.7821	4033.7803	0.0018	8	2	6	2	10	2	10	7	2	5	2	9	2	9
4033.7821	4033.7873	-0.0052	8	2	6	2	10	0	10	7	2	5	2	9	0	9
4033.7821	4033.7833	-0.0012	8	2	6	1	8	2	10	7	2	5	1	7	2	9
4033.7821	4033.7866	-0.0045	8	2	6	0	8	2	10	7	2	5	0	7	2	9
4033.7821	4033.7818	0.0003	8	2	6	2	10	1	9	7	2	5	2	9	1	8
4033.7821	4033.7867	-0.0046	8	2	6	2	10	1	10	7	2	5	2	9	1	9
4033.7821	4033.7851	-0.0030	8	2	6	1	8	1	9	7	2	5	1	7	1	8
4033.9203	4033.9256	-0.0053	8	2	6	1	9	1	9	7	2	5	1	8	1	8
4033.9203	4033.9262	-0.0059	8	2	6	1	9	0	9	7	2	5	1	8	0	8
4033.9203	4033.9231	-0.0028	8	2	6	1	9	2	11	7	2	5	1	8	2	10
4033.9203	4033.9220	-0.0017	8	2	6	2	9	2	11	7	2	5	2	8	2	10
4033.9203	4033.9193	0.0010	8	2	6	1	7	2	9	7	2	5	1	6	2	8
4033.9203	4033.9177	0.0026	8	2	6	1	9	2	9	7	2	5	1	8	2	8
4033.9203	4033.9229	-0.0026	8	2	6	1	9	1	10	7	2	5	1	8	1	9
4033.9203	4033.9172	0.0031	8	2	6	1	9	2	10	7	2	5	1	8	2	9
4033.9203	4033.9267	-0.0064	8	2	6	2	9	1	10	7	2	5	2	8	1	9
4033.9203	4033.9233	-0.0030	8	2	6	2	9	2	10	7	2	5	2	8	2	9
4034.0654	4034.0700	-0.0046	8	4	4	0	8	2	7	7	4	3	0	7	2	6
4034.0654	4034.0639	0.0015	8	2	6	2	8	1	7	7	2	5	2	7	1	6
4034.0654	4034.0644	0.0010	8	2	6	2	8	1	8	7	2	5	2	7	1	7
4034.0654	4034.0673	-0.0019	8	2	6	2	8	1	9	7	2	5	2	7	1	8
4034.0654	4034.0653	0.0001	8	0	8	2	8	0	8	7	2	5	2	7	0	7
4034.0654	4034.0661	-0.0007	8	2	6	2	8	2	8	7	0	7	2	7	2	7
4034.0654	4034.0616	0.0038	8	2	6	2	8	2	10	7	2	5	2	7	2	9

4034.0654	4034.0606	0.0048	8	4	4	2	8	2	9	7	4	3	2	7	2	8
4034.0654	4034.0690	-0.0036	8	2	6	2	8	2	6	7	2	5	2	7	2	5
4355.1746	4355.1705	0.0041	9	3	7	2	9	2	10	8	3	6	2	8	2	9
4355.1746	4355.1772	-0.0026	9	1	9	2	9	2	7	8	1	8	2	8	2	6
4355.1746	4355.1698	0.0048	9	1	9	2	9	0	9	8	1	8	2	8	0	8
4355.1746	4355.1617	0.0129	9	1	9	2	9	2	11	8	1	8	2	8	2	10
4355.1746	4355.1676	0.0070	9	1	9	0	9	1	9	8	1	8	2	8	1	8
4355.1746	4355.1802	-0.0056	9	1	9	2	8	1	8	8	1	8	2	7	1	7
4355.2122	4355.2251	-0.0129	9	1	9	1	10	2	11	8	1	8	1	9	2	10
4355.2122	4355.2244	-0.0122	9	1	9	2	10	0	10	8	1	8	2	9	0	9
4355.2122	4355.2131	-0.0009	9	1	9	1	8	2	10	8	1	8	1	7	2	9
4355.2122	4355.2095	0.0027	9	1	9	1	10	2	12	8	1	8	1	9	2	11
4355.2122	4355.2184	-0.0062	9	1	9	1	10	0	10	8	1	8	1	9	0	9
4355.2122	4355.2147	-0.0025	9	1	9	2	10	2	12	8	1	8	2	9	2	11
4355.2122	4355.2387	-0.0265	9	1	9	1	10	1	11	8	1	8	1	9	1	10
4355.2122	4355.2309	-0.0187	9	1	9	2	10	2	11	8	1	8	2	9	2	10
4355.2122	4355.2160	-0.0038	9	1	9	1	10	1	10	8	1	8	1	9	1	9
4355.2122	4355.2040	0.0082	9	1	9	2	9	2	10	8	1	8	2	8	2	9
4355.2500	4355.2823	-0.0323	9	1	9	2	11	1	10	8	1	8	2	10	1	9
4355.2500	4355.2507	-0.0007	9	1	9	2	11	1	11	8	1	8	2	10	1	10
4355.2500	4355.2735	-0.0235	9	1	9	2	10	2	10	8	1	8	2	7	2	9
4355.2500	4355.2567	-0.0067	9	1	9	0	9	2	11	8	1	8	0	8	2	10
4355.2500	4355.2610	-0.0110	9	1	9	2	11	2	13	8	1	8	2	10	2	12
4355.2500	4355.2701	-0.0201	9	1	9	1	10	2	10	8	1	8	1	9	2	9
4355.2500	4355.2558	-0.0058	9	1	9	1	9	2	11	8	1	8	1	8	2	10
4355.2500	4355.2699	-0.0199	9	1	9	2	11	0	11	8	1	8	2	10	0	10
4355.2500	4355.2643	-0.0143	9	1	9	2	10	1	11	8	1	8	2	9	1	10
4355.2500	4355.2782	-0.0282	9	1	9	2	11	2	12	8	1	8	2	10	2	11
4355.3000	4355.2997	0.0003	9	1	9	2	7	2	6	8	1	8	2	6	2	5
4355.3000	4355.3088	-0.0088	9	1	9	2	11	2	10	8	1	8	2	10	2	9
4355.3000	4355.3015	-0.0015	9	1	9	2	7	0	7	8	5	4	2	6	2	6
4355.3000	4355.3064	-0.0064	9	1	9	1	9	2	8	8	1	8	1	8	2	7
4355.3000	4355.2902	0.0098	9	1	9	2	11	1	12	8	1	8	2	10	1	11
4355.3000	4355.3097	-0.0097	9	1	9	2	11	2	11	8	1	8	2	10	2	10
4355.3000	4355.3070	-0.0070	9	1	9	1	9	0	9	8	1	8	1	8	0	8
4355.3000	4355.3053	-0.0053	9	1	9	0	9	2	9	8	1	8	0	8	2	8
4355.3000	4355.3039	-0.0039	9	1	9	0	9	2	8	8	1	8	0	8	2	7
4453.2773	4453.3022	-0.0249	9	2	8	2	11	2	12	8	2	7	2	10	2	11
4453.2773	4453.2763	0.0010	9	2	8	2	10	1	11	8	2	7	2	9	1	10
4453.2773	4453.2789	-0.0016	9	2	8	2	11	2	13	8	2	7	2	10	2	12
4453.2773	4453.2844	-0.0071	9	2	8	0	9	2	11	8	2	7	0	8	2	10
4453.2773	4453.2880	-0.0107	9	2	8	1	10	1	11	8	2	7	1	9	1	10
4453.2773	4453.2839	-0.0066	9	2	8	1	9	2	11	8	2	7	1	8	2	10
4453.2773	4453.2799	-0.0026	9	2	8	2	11	1	11	8	2	7	2	10	1	10
4453.2773	4453.2811	-0.0038	9	2	8	2	11	0	11	8	2	7	2	10	0	10
4453.2773	4453.2817	-0.0044	9	2	8	2	10	2	11	8	2	7	2	9	2	10
4453.2773	4453.2850	-0.0077	9	2	8	1	10	2	11	8	2	7	1	9	2	10
4476.9033	4476.8725	0.0308	9	6	4	1	9	1	9	8	6	3	1	8	1	8
4476.9033	4476.8891	0.0142	9	6	3	0	9	2	11	8	6	2	0	8	2	10
4476.9033	4476.8889	0.0144	9	6	4	0	9	2	11	8	6	3	0	8	2	10
4476.9033	4476.8908	0.0125	9	6	3	2	11	1	11	8	6	2	2	10	1	10
4476.9033	4476.8906	0.0127	9	6	4	2	11	1	11	8	6	3	2	10	1	10
4476.9033	4476.8902	0.0131	9	6	4	1	9	2	11	8	6	3	1	8	2	10

4476.9033	4476.8874	0.0159	9	6	3	2	11	0	11	8	6	2	2	10	0	10
4476.9033	4476.8873	0.0160	9	6	4	2	11	0	11	8	6	3	2	10	0	10
4476.9033	4476.8904	0.0129	9	6	3	1	9	2	11	8	6	2	1	8	2	10
4476.9033	4476.8727	0.0306	9	6	3	1	9	1	9	8	6	2	1	8	1	8
4477.0774	4477.0648	0.0126	9	6	4	2	10	2	12	8	6	3	2	9	2	11
4477.0774	4477.0243	0.0531	9	6	3	1	10	0	10	8	6	2	1	9	0	9
4477.0774	4477.0519	0.0255	9	6	3	1	10	2	12	8	6	2	1	9	2	11
4477.0774	4477.0517	0.0257	9	6	4	1	10	2	12	8	6	3	1	9	2	11
4477.0774	4477.0411	0.0363	9	6	3	2	11	1	12	8	6	2	2	10	1	11
4477.0774	4477.0409	0.0365	9	6	4	2	11	1	12	8	6	3	2	10	1	11
4477.0774	4477.0241	0.0533	9	6	4	1	10	0	10	8	6	3	1	9	0	9
4477.0774	4477.0650	0.0124	9	6	3	2	10	2	12	8	6	2	2	9	2	11
4477.0774	4477.0312	0.0462	9	6	4	1	9	1	10	8	6	3	1	8	1	9
4477.0774	4477.0314	0.0460	9	6	3	1	9	1	10	8	6	2	1	8	1	9
4478.6422	4478.6397	0.0025	9	5	4	2	11	0	11	8	5	3	2	10	0	10
4478.6422	4478.6332	0.0090	9	5	5	2	11	2	13	8	5	4	2	10	2	12
4478.6422	4478.6399	0.0023	9	5	4	2	11	1	11	8	5	3	2	10	1	10
4478.6422	4478.6260	0.0162	9	5	5	2	11	1	11	8	5	4	2	10	1	10
4478.6422	4478.6258	0.0164	9	5	5	0	9	2	11	8	5	4	0	8	2	10
4478.6422	4478.6281	0.0141	9	5	5	1	9	2	11	8	5	4	1	8	2	10
4478.6422	4478.6305	0.0117	9	5	4	0	9	1	9	8	5	3	0	8	1	8
4478.6422	4478.6160	0.0262	9	5	5	0	9	1	9	8	3	6	0	8	1	8
4478.6422	4478.6306	0.0116	9	5	4	1	9	0	9	8	5	3	1	8	0	8
4478.6422	4478.6257	0.0165	9	5	5	2	11	0	11	8	5	4	2	10	0	10
4478.7483	4478.7486	-0.0003	9	5	4	1	10	1	10	8	5	3	1	9	1	9
4478.7483	4478.7341	0.0142	9	5	5	1	10	1	10	8	5	4	1	9	1	9
4478.7483	4478.7280	0.0203	9	5	4	0	9	1	10	8	5	3	0	8	1	9
4478.7483	4478.7409	0.0074	9	5	4	2	11	1	12	8	5	3	2	10	1	11
4478.7483	4478.7440	0.0043	9	5	5	2	11	2	12	8	5	4	2	10	2	11
4478.7483	4478.7288	0.0195	9	5	4	1	9	1	10	8	5	3	1	8	1	9
4478.7483	4478.7269	0.0214	9	5	5	2	11	1	12	8	5	4	2	10	1	11
4478.7483	4478.7583	-0.0100	9	5	4	1	10	2	12	8	5	3	1	9	2	11
4478.7483	4478.7703	-0.0220	9	5	5	2	10	2	12	8	5	4	2	9	2	11
4478.7483	4478.7438	0.0045	9	5	5	1	10	2	12	8	5	4	1	9	2	11
4481.5761	4481.5678	0.0083	9	4	6	1	9	0	9	8	4	5	1	8	0	8
4481.5761	4481.5679	0.0082	9	4	6	0	9	1	9	8	4	5	0	8	1	8
4481.5761	4481.5676	0.0085	9	4	6	0	9	0	9	8	4	5	0	8	0	8
4481.5761	4481.5681	0.0080	9	4	6	1	9	1	9	8	4	5	1	8	1	8
4481.5761	4481.5742	0.0019	9	4	6	2	11	1	11	8	4	5	2	10	1	10
4481.5761	4481.5739	0.0022	9	4	6	2	11	0	11	8	4	5	2	10	0	10
4481.5761	4481.5727	0.0034	9	4	6	0	9	2	11	8	4	5	0	8	2	10
4481.5761	4481.5690	0.0071	9	4	6	2	11	2	9	8	4	5	2	10	2	8
4481.5761	4481.5730	0.0031	9	4	6	1	9	2	11	8	4	5	1	8	2	10
4481.5761	4481.5787	-0.0026	9	4	6	2	11	2	13	8	4	5	2	10	2	12
4481.6491	4481.6389	0.0102	9	4	6	2	11	1	12	8	4	5	2	10	1	11
4481.6491	4481.6318	0.0173	9	4	6	1	9	1	10	8	4	5	1	8	1	9
4481.6491	4481.6420	0.0071	9	4	6	2	11	1	10	8	4	5	2	10	1	9
4481.6491	4481.6711	-0.0220	9	4	6	1	8	2	10	8	4	5	1	7	2	9
4481.6491	4481.6625	-0.0134	9	4	6	1	10	2	12	8	4	5	1	9	2	11
4481.6491	4481.6448	0.0043	9	4	6	2	11	2	12	8	4	5	2	10	2	11
4481.6491	4481.6587	-0.0096	9	4	6	1	10	1	10	8	4	5	1	9	1	9
4481.6491	4481.6316	0.0175	9	4	6	0	9	1	10	8	4	5	0	8	1	9
4481.6491	4481.6587	-0.0096	9	4	6	1	10	0	10	8	4	5	1	9	0	9

4481.6491	4481.6783	-0.0292	9	4	6	2	10	2	12	8	4	5	2	9	2	11
4481.7375	4481.7277	0.0098	9	4	6	1	10	1	9	8	4	5	1	9	1	8
4481.7375	4481.7200	0.0175	9	4	6	1	10	2	9	8	4	5	1	9	2	8
4481.7375	4481.7640	-0.0265	9	4	6	2	9	2	11	8	4	5	2	8	2	10
4481.7375	4481.7343	0.0032	9	4	6	2	10	1	11	8	4	5	2	9	1	10
4481.7375	4481.7409	-0.0034	9	4	6	2	10	2	11	8	4	5	2	9	2	10
4481.7375	4481.7227	0.0148	9	4	6	1	10	1	11	8	4	5	1	9	1	10
4481.7375	4481.7366	0.0009	9	4	6	2	10	1	9	8	4	5	2	9	1	8
4481.7375	4481.7055	0.0320	9	4	6	2	11	2	11	8	4	5	2	10	2	10
4481.7375	4481.7321	0.0054	9	4	6	1	10	2	11	8	4	5	1	9	2	10
4481.7375	4481.7286	0.0089	9	4	6	1	8	1	9	8	4	5	1	7	1	8
4482.2443	4482.2469	-0.0026	9	4	5	2	11	2	9	8	4	4	2	10	2	8
4482.2443	4482.2448	-0.0005	9	4	5	0	9	0	9	8	6	2	0	8	0	8
4482.2443	4482.2517	-0.0074	9	4	5	2	11	0	11	8	4	4	2	10	0	10
4482.2443	4482.2502	-0.0059	9	4	5	1	9	2	11	8	4	4	1	8	2	10
4482.2443	4482.2499	-0.0056	9	4	5	0	9	2	11	8	4	4	0	8	2	10
4482.2443	4482.2451	-0.0008	9	4	5	1	9	0	9	8	4	4	1	8	0	8
4482.2443	4482.2519	-0.0076	9	4	5	2	11	1	11	8	4	4	2	10	1	10
4482.2443	4482.2455	-0.0012	9	4	5	1	9	1	9	8	4	4	1	8	1	8
4482.2443	4482.2451	-0.0008	9	4	5	0	9	1	9	8	4	4	0	8	1	8
4482.2443	4482.2564	-0.0121	9	4	5	2	11	2	13	8	4	4	2	10	2	12
4482.3230	4482.3077	0.0153	9	4	5	0	9	1	10	8	4	4	0	8	1	9
4482.3230	4482.3186	0.0044	9	4	5	2	11	1	10	8	4	4	2	10	1	9
4482.3230	4482.3410	-0.0180	9	4	5	1	10	0	10	8	4	4	1	9	0	9
4482.3230	4482.3411	-0.0181	9	4	5	1	10	1	10	8	4	4	1	9	1	9
4482.3230	4482.3079	0.0151	9	4	5	1	9	1	10	8	4	4	1	8	1	9
4482.3230	4482.3215	0.0015	9	4	5	2	11	2	12	8	4	4	2	10	2	11
4482.3230	4482.3536	-0.0306	9	4	5	1	8	2	10	8	4	4	1	7	2	9
4482.3230	4482.3122	0.0108	9	4	5	2	11	2	10	8	4	4	2	10	2	9
4482.3230	4482.3153	0.0077	9	4	5	2	11	1	12	8	4	4	2	10	1	11
4482.3230	4482.3449	-0.0219	9	4	5	1	10	2	12	8	4	4	1	9	2	11
4482.4227	4482.4112	0.0115	9	4	5	2	10	2	9	8	4	4	2	9	2	8
4482.4227	4482.3947	0.0280	9	4	5	2	8	1	9	8	4	4	2	7	1	8
4482.4227	4482.4007	0.0220	9	4	5	1	10	2	9	8	4	4	1	9	2	8
4482.4227	4482.4130	0.0097	9	4	5	1	10	2	11	8	4	4	1	9	2	10
4482.4227	4482.4164	0.0063	9	4	5	2	10	1	11	8	4	4	2	9	1	10
4482.4227	4482.4190	0.0037	9	4	5	2	10	1	9	8	4	4	2	9	1	8
4482.4227	4482.4038	0.0189	9	4	5	1	10	1	11	8	4	4	1	9	1	10
4482.4227	4482.4103	0.0124	9	4	5	1	8	1	9	8	4	4	1	7	1	8
4482.4227	4482.4233	-0.0006	9	4	5	2	10	2	11	8	4	4	2	9	2	10
4482.4227	4482.4087	0.0140	9	4	5	1	10	1	9	8	4	4	1	9	1	8
4482.5336	4482.4974	0.0362	9	3	7	2	11	1	11	8	3	6	2	10	1	10
4482.5336	4482.4984	0.0352	9	3	7	2	11	2	13	8	3	6	2	10	2	12
4482.5336	4482.4976	0.0360	9	3	7	2	11	0	11	8	3	6	2	10	0	10
4482.5336	4482.4979	0.0357	9	3	7	1	9	2	11	8	3	6	1	8	2	10
4482.5336	4482.4987	0.0349	9	3	7	0	9	2	11	8	3	6	0	8	2	10
4482.5634	4482.5460	0.0174	9	3	7	1	10	0	10	8	3	6	1	9	0	9
4482.5634	4482.5760	-0.0126	9	3	7	2	11	2	11	8	3	6	2	10	2	10
4482.5634	4482.5372	0.0262	9	1	9	0	9	1	10	8	3	6	0	8	1	9
4482.5634	4482.5366	0.0268	9	3	7	1	9	1	10	8	3	6	1	8	1	9
4482.5634	4482.5362	0.0272	9	3	7	2	11	1	12	8	3	6	2	10	1	11
4482.5634	4482.5471	0.0163	9	3	7	1	10	2	12	8	3	6	1	9	2	11
4482.5634	4482.5382	0.0252	9	3	7	2	11	2	12	8	3	6	2	10	2	11

4482.5634	4482.5461	0.0173	9	3	7	2	10	2	12	8	3	6	2	9	2	11
4482.5634	4482.5461	0.0173	9	3	7	1	10	1	10	8	3	6	1	9	1	9
4482.5634	4482.5372	0.0262	9	5	5	2	11	1	10	8	3	6	2	10	1	9
4482.5953	4482.5883	0.0070	9	3	7	2	10	2	11	8	3	6	2	9	2	10
4482.5953	4482.5865	0.0088	9	3	7	1	10	2	11	8	3	6	1	9	2	10
4482.5953	4482.5850	0.0103	9	3	7	1	10	1	11	8	3	6	1	9	1	10
4482.5953	4482.5946	0.0007	9	3	7	2	9	2	11	8	3	6	2	8	2	10
4482.5953	4482.5851	0.0102	9	3	7	2	10	1	11	8	3	6	2	9	1	10
4482.6159	4482.6243	-0.0084	9	3	7	1	8	0	8	8	3	6	1	7	2	7
4482.6159	4482.5939	0.0220	9	3	7	2	9	0	9	8	3	6	2	8	0	8
4482.6159	4482.5901	0.0258	9	5	5	2	8	2	9	8	3	6	2	7	2	8
4482.6159	4482.5935	0.0224	9	1	9	2	9	1	9	8	3	6	2	8	1	8
4482.6159	4482.6352	-0.0193	9	3	7	2	9	1	8	8	3	6	2	8	1	7
4482.6159	4482.6346	-0.0187	9	3	7	2	9	1	10	8	1	8	2	8	1	9
4482.6159	4482.6248	-0.0089	9	3	7	1	10	2	10	8	3	6	1	9	2	9
4482.6159	4482.6366	-0.0207	9	5	5	2	9	2	10	8	5	4	2	8	2	9
4482.6159	4482.6260	-0.0101	9	5	5	2	10	0	10	8	1	8	2	9	2	9
4496.8713	4496.8692	0.0021	9	3	6	2	11	0	11	8	3	5	2	10	0	10
4496.8713	4496.8848	-0.0135	9	3	6	1	9	1	10	8	3	5	1	8	1	9
4496.8713	4496.8912	-0.0199	9	3	6	2	11	2	12	8	3	5	2	10	2	11
4496.8713	4496.8624	0.0089	9	3	6	1	9	2	11	8	3	5	1	8	2	10
4496.8713	4496.8902	-0.0189	9	3	6	2	11	1	10	8	3	5	2	10	1	9
4496.8713	4496.8894	-0.0181	9	3	6	2	11	1	12	8	3	5	2	10	1	11
4496.8713	4496.8685	0.0028	9	3	6	2	11	2	13	8	3	5	2	10	2	12
4496.8713	4496.8627	0.0086	9	3	6	0	9	2	11	8	3	5	0	8	2	10
4496.8713	4496.8850	-0.0137	9	3	6	0	9	1	10	8	3	5	0	8	1	9
4496.8713	4496.8689	0.0024	9	3	6	2	11	1	11	8	3	5	2	10	1	10
4496.9866	4497.0221	-0.0355	9	3	6	1	10	2	10	8	3	5	1	9	2	9
4496.9866	4496.9834	0.0032	9	3	6	1	8	2	10	8	3	5	1	7	2	9
4496.9866	4496.9808	0.0058	9	3	6	1	10	1	10	8	3	5	1	9	1	9
4496.9866	4496.9808	0.0058	9	3	6	1	10	0	10	8	3	5	1	9	0	9
4496.9866	4496.9811	0.0055	9	3	6	1	10	2	12	8	3	5	1	9	2	11
4496.9866	4496.9885	-0.0019	9	3	6	2	10	2	12	8	3	5	2	9	2	11
4496.9866	4497.0020	-0.0154	9	3	6	1	10	1	11	8	3	5	1	9	1	10
4496.9866	4497.0133	-0.0267	9	3	6	2	10	1	11	8	3	5	2	9	1	10
4496.9866	4497.0156	-0.0290	9	3	6	2	10	2	11	8	3	5	2	9	2	10
4496.9866	4497.0006	-0.0140	9	3	6	1	10	2	11	8	3	5	1	9	2	10
4497.1052	4497.0993	0.0059	9	3	6	2	9	2	11	8	3	5	2	8	2	10
4497.1052	4497.1233	-0.0181	9	1	8	2	9	2	8	8	1	7	2	8	2	7
4497.1052	4497.1029	0.0023	9	3	6	2	9	2	7	8	3	5	2	8	2	6
4497.1052	4497.1011	0.0041	9	3	6	2	9	0	9	8	3	5	2	8	0	8
4497.1052	4497.1264	-0.0212	9	3	6	2	9	1	10	8	3	5	2	8	1	9
4497.1052	4497.1254	-0.0202	9	3	6	2	9	1	8	8	3	5	2	8	1	7
4497.1052	4497.1005	0.0047	9	3	6	2	9	1	9	8	3	5	2	8	1	8
4497.1052	4497.1228	-0.0176	9	3	6	2	9	2	10	8	3	5	2	8	2	9
4527.1750	4527.1484	0.0266	9	1	8	2	10	1	11	8	1	7	2	9	1	10
4527.1750	4527.1500	0.0250	9	1	8	2	10	2	11	8	1	7	2	9	2	10
4527.1750	4527.1581	0.0169	9	1	8	1	10	2	12	8	1	7	1	9	2	11
4527.1750	4527.1678	0.0072	9	1	8	1	8	2	9	8	1	7	1	7	2	8
4527.1750	4527.1670	0.0080	9	1	8	1	8	1	9	8	1	7	1	7	1	8
4527.1750	4527.1484	0.0266	9	1	8	2	10	1	9	8	1	7	2	9	1	8
4527.1750	4527.1580	0.0170	9	1	8	1	10	0	10	8	1	7	1	9	0	9
4527.1750	4527.1481	0.0269	9	1	8	2	10	2	9	8	1	7	2	9	2	8

4527.1750	4527.1577	0.0173	9	1	8	1	10	2	8	8	1	7	1	9	2	7
4527.1750	4527.1576	0.0174	9	1	8	1	10	1	10	8	1	7	1	9	1	9
4527.2000	4527.1958	0.0042	9	1	8	1	10	2	9	8	1	7	1	9	2	8
4527.2000	4527.1936	0.0064	9	1	8	2	8	1	8	8	1	7	2	7	1	7
4527.2000	4527.1941	0.0059	9	1	8	2	8	2	6	8	1	7	2	7	2	5
4527.2000	4527.1884	0.0116	9	3	6	2	10	0	10	8	3	5	2	9	0	9
4527.2000	4527.1942	0.0058	9	1	8	2	8	0	8	8	1	7	2	7	0	7
4527.2000	4527.1941	0.0059	9	1	8	2	8	2	10	8	1	7	2	7	2	9
4527.2000	4527.1953	0.0047	9	1	8	1	10	1	9	8	1	7	1	9	1	8
4527.2000	4527.2061	-0.0061	9	1	8	1	8	2	8	8	1	7	1	7	2	7
4527.2000	4527.1964	0.0036	9	1	8	1	10	2	11	8	1	7	1	9	2	10
4527.2000	4527.1949	0.0051	9	1	8	1	10	1	11	8	1	7	1	9	1	10
4527.2500	4527.2356	0.0144	9	1	8	2	8	2	7	8	1	7	2	7	2	6
4527.2500	4527.2389	0.0111	9	1	8	2	11	2	9	8	1	7	2	10	2	8
4527.2500	4527.2335	0.0165	9	1	8	2	8	1	9	8	1	7	2	7	1	8
4527.2500	4527.2398	0.0102	9	1	8	2	11	0	11	8	1	7	2	10	0	10
4527.2500	4527.2396	0.0104	9	1	8	2	11	1	11	8	1	7	2	10	1	10
4527.2500	4527.2357	0.0143	9	1	8	2	8	2	9	8	1	7	2	7	2	8
4527.2500	4527.2407	0.0093	9	1	8	2	11	2	13	8	1	7	2	10	2	12
4527.2500	4527.2335	0.0165	9	1	8	1	10	2	10	8	1	7	1	9	2	9
4527.2500	4527.2344	0.0156	9	1	8	2	8	1	7	8	1	7	2	7	1	6
4527.2750	4527.2632	0.0118	9	1	8	1	9	0	9	8	1	7	1	8	0	8
4527.2750	4527.2764	-0.0014	9	1	8	2	11	2	10	8	1	7	2	10	2	9
4527.2750	4527.2628	0.0122	9	1	8	1	9	1	9	8	1	7	1	8	1	8
4527.2750	4527.2681	0.0069	9	1	8	0	9	0	9	8	1	7	0	8	0	8
4527.2750	4527.2777	-0.0027	9	1	8	2	11	1	10	8	1	7	2	10	1	9
4527.2750	4527.2783	-0.0033	9	1	8	2	11	2	12	8	1	7	2	10	2	11
4527.2750	4527.2775	-0.0025	9	1	8	2	11	1	12	8	1	7	2	10	1	11
4527.2750	4527.2676	0.0074	9	1	8	0	9	1	9	8	1	7	0	8	1	8
4527.2750	4527.2635	0.0115	9	1	8	1	9	2	11	8	1	7	1	8	2	10
4527.2750	4527.2683	0.0067	9	1	8	0	9	2	11	8	1	7	0	8	2	10
4527.3125	4527.2952	0.0173	9	1	8	2	7	2	9	8	1	7	2	6	2	8
4527.3125	4527.3013	0.0112	9	1	8	1	9	1	8	8	1	7	1	8	1	7
4527.3125	4527.3061	0.0064	9	1	8	0	9	1	8	8	1	7	0	8	1	7
4527.3125	4527.3016	0.0109	9	1	8	1	9	2	10	8	1	7	1	8	2	9
4527.3125	4527.3041	0.0084	9	1	8	0	9	2	8	8	1	7	0	8	2	7
4527.3125	4527.3011	0.0114	9	1	8	1	9	2	8	8	1	7	1	8	2	7
4527.3125	4527.3064	0.0061	9	1	8	0	9	2	10	8	1	7	0	8	2	9
4527.3125	4527.3011	0.0114	9	1	8	1	9	1	10	8	1	7	1	8	1	9
4527.3125	4527.3060	0.0065	9	1	8	0	9	1	10	8	1	7	0	8	1	9
4527.3375	4527.3360	0.0015	9	1	8	2	7	2	8	8	1	7	2	6	2	7
4527.3375	4527.3372	0.0003	9	1	8	2	7	2	6	8	1	7	2	6	2	5
4527.3375	4527.3341	0.0034	9	1	8	2	7	1	6	8	1	7	2	6	1	5
4527.3375	4527.3345	0.0030	9	1	8	2	7	1	8	8	1	7	2	6	1	7
4527.3375	4527.3396	-0.0021	9	1	8	1	9	2	9	8	1	7	1	8	2	8
4527.3375	4527.3155	0.0220	9	1	8	2	11	2	11	8	1	7	2	10	2	10
4527.3375	4527.3445	-0.0070	9	1	8	0	9	2	9	8	1	7	0	8	2	8
4546.3937	4546.3755	0.0182	9	2	7	2	11	0	11	8	2	6	2	10	0	10
4546.3937	4546.3670	0.0267	9	2	7	2	11	2	11	8	2	6	2	10	2	10
4546.3937	4546.3751	0.0186	9	2	7	2	11	1	11	8	2	6	2	10	1	10
4546.3937	4546.3716	0.0221	9	2	7	2	11	1	12	8	2	6	2	10	1	11
4546.3937	4546.3683	0.0254	9	2	7	2	11	2	12	8	2	6	2	10	2	11
4546.3937	4546.3696	0.0241	9	2	7	2	11	1	10	8	2	6	2	10	1	9

4546.3937	4546.3753	0.0184	9	2	7	1	9	1	10	8	2	6	1	8	1	9
4546.3937	4546.3730	0.0207	9	2	7	2	11	2	13	8	2	6	2	10	2	12
4546.3937	4546.3752	0.0185	9	2	7	1	9	2	11	8	2	6	1	8	2	10
4546.3937	4546.3776	0.0161	9	2	7	0	9	2	11	8	2	6	0	8	2	10
4546.4884	4546.4697	0.0187	9	2	7	2	10	2	11	8	2	6	2	9	2	10
4546.4884	4546.4732	0.0152	9	2	7	2	10	1	11	8	2	6	2	9	1	10
4546.4884	4546.4802	0.0082	9	2	7	1	10	2	12	8	2	6	1	9	2	11
4546.4884	4546.4713	0.0171	9	2	7	2	10	2	12	8	2	6	2	9	2	11
4546.4884	4546.4732	0.0152	9	2	7	1	10	2	10	8	2	6	1	9	2	9
4546.4884	4546.4727	0.0157	9	2	7	1	8	2	10	8	2	6	1	7	2	9
4546.4884	4546.4789	0.0095	9	2	7	1	10	1	11	8	2	6	1	9	1	10
4546.4884	4546.4740	0.0144	9	2	7	1	10	2	11	8	2	6	1	9	2	10
4546.4884	4546.4828	0.0056	9	2	7	1	10	0	10	8	2	6	1	9	0	9
4546.4884	4546.4823	0.0061	9	2	7	1	10	1	10	8	2	6	1	9	1	9
4546.5937	4546.5801	0.0136	9	2	7	2	9	2	11	8	2	6	2	8	2	10
4546.5937	4546.5797	0.0140	9	2	7	2	9	1	8	8	2	6	2	8	1	7
4546.5937	4546.5851	0.0086	9	0	9	2	9	2	8	8	4	4	0	8	2	7
4546.5937	4546.5860	0.0077	9	2	7	2	9	2	7	8	2	6	2	8	2	6
4546.5937	4546.5797	0.0140	9	2	7	2	9	2	9	8	2	6	2	8	2	8
4546.5937	4546.5826	0.0111	9	2	7	2	9	1	10	8	2	6	2	8	1	9
4546.5937	4546.5770	0.0167	9	2	7	2	9	2	10	8	4	4	2	8	2	9
4546.5937	4546.5830	0.0107	9	0	9	2	9	0	9	8	0	8	2	8	0	8
4546.5937	4546.5824	0.0113	9	2	7	2	9	1	9	8	2	6	2	8	1	8
4834.5125	4834.5333	-0.0208	10	1	10	2	10	1	9	9	1	9	2	9	1	8
4834.5125	4834.5326	-0.0201	10	1	10	2	10	2	11	9	1	9	2	9	2	10
4834.5125	4834.5362	-0.0237	10	3	8	2	9	2	10	9	3	7	2	8	2	9
4834.5375	4834.5509	-0.0134	10	1	10	2	11	0	11	9	1	9	2	10	0	10
4834.5375	4834.5516	-0.0141	10	1	10	1	11	2	9	9	1	9	1	8	0	8
4834.5375	4834.5539	-0.0164	10	1	10	1	9	0	9	9	1	9	1	10	2	8
4834.5500	4834.5624	-0.0124	10	1	10	1	9	1	8	9	1	9	1	8	1	7
4834.5500	4834.5636	-0.0136	10	1	10	2	11	2	12	9	1	9	2	10	2	11
4834.5500	4834.5636	-0.0136	10	3	8	2	9	1	10	9	3	7	2	8	1	9
4834.5750	4834.5884	-0.0134	10	1	10	2	12	2	10	9	1	9	2	11	2	9
4834.5750	4834.5900	-0.0150	10	1	10	2	8	2	8	9	1	9	2	7	2	7
4834.5750	4834.5903	-0.0153	10	1	10	2	12	2	14	9	1	9	2	11	2	13
4834.5750	4834.5885	-0.0135	10	1	10	2	11	1	12	9	1	9	2	10	1	11
4834.5750	4834.5918	-0.0168	10	1	10	0	10	2	11	9	1	9	0	9	2	10
4834.5750	4834.5886	-0.0136	10	1	10	1	11	2	10	9	1	9	1	10	2	9
4834.6125	4834.6022	0.0103	10	1	10	2	11	2	10	9	1	9	2	10	2	9
4834.6125	4834.6044	0.0081	10	1	10	0	10	1	9	9	1	9	0	9	1	8
4834.6125	4834.6060	0.0065	10	1	10	1	10	1	9	9	1	9	1	9	1	8
4834.6125	4834.6060	0.0065	10	1	10	2	12	2	13	9	1	9	2	11	2	12
4834.6259	4834.6300	-0.0041	10	1	10	0	10	2	10	9	1	9	0	9	2	9
4834.6259	4834.6281	-0.0022	10	1	10	2	12	2	11	9	1	9	2	11	2	10
4834.6259	4834.6313	-0.0054	10	1	10	1	10	0	10	9	1	9	1	9	0	9
4942.7750	4942.7817	-0.0067	10	2	9	2	10	2	8	9	2	8	2	9	2	7
4942.7750	4942.7712	0.0038	10	2	9	2	10	1	10	9	2	8	2	9	1	9
4942.7750	4942.7656	0.0094	10	2	9	2	10	2	12	9	2	8	2	9	2	11
4942.7750	4942.7717	0.0033	10	2	9	2	10	0	10	9	2	8	2	9	0	9
4942.8125	4942.7941	0.0184	10	2	9	2	10	1	11	9	2	8	2	9	1	10
4942.8125	4942.8137	-0.0012	10	2	9	2	9	2	11	9	2	8	2	8	2	10
4942.8125	4942.7977	0.0148	10	2	9	2	10	2	11	9	2	8	2	9	2	10
4942.8125	4942.8124	0.0001	10	2	9	1	11	1	11	9	2	8	1	10	1	10

4942.8125	4942.8131	-0.0006	10	2	9	1	11	0	11	9	2	8	1	10	0	10
4942.8125	4942.8026	0.0099	10	2	9	2	11	2	13	9	2	8	2	10	2	12
4942.8125	4942.8053	0.0072	10	2	9	1	9	2	11	9	2	8	1	8	2	10
4942.8125	4942.8042	0.0083	10	2	9	2	11	1	11	9	2	8	2	10	1	10
4942.8125	4942.8090	0.0035	10	2	9	2	11	0	11	9	2	8	2	10	0	10
4942.8125	4942.8088	0.0037	10	2	9	1	11	2	13	9	2	8	1	10	2	12
4942.8375	4942.8377	-0.0002	10	2	9	1	11	2	12	9	2	8	1	10	2	11
4942.8375	4942.8454	-0.0079	10	2	9	2	12	2	14	9	2	8	2	11	2	13
4942.8375	4942.8408	-0.0033	10	2	9	1	11	1	12	9	2	8	1	10	1	11
4942.8375	4942.8298	0.0077	10	2	9	2	11	1	12	9	2	8	2	10	1	11
4942.8375	4942.8465	-0.0090	10	2	9	2	12	1	12	9	2	8	2	11	1	11
4942.8375	4942.8474	-0.0099	10	2	9	2	12	0	12	9	2	8	2	11	0	11
4942.8375	4942.8306	0.0069	10	2	9	2	11	2	12	9	2	8	2	10	2	11
4942.8375	4942.8504	-0.0129	10	2	9	1	10	2	12	9	2	8	1	9	2	11
4942.8375	4942.8509	-0.0134	10	2	9	0	10	2	12	9	2	8	0	9	2	11
4942.8750	4942.8705	0.0045	10	2	9	1	11	2	11	9	2	8	1	10	2	10
4942.8750	4942.8736	0.0014	10	2	9	1	10	2	11	9	2	8	1	9	2	10
4942.8750	4942.8707	0.0043	10	2	9	2	12	1	11	9	2	8	2	11	1	10
4942.8750	4942.8743	0.0007	10	2	9	0	10	2	11	9	2	8	0	9	2	10
4942.8750	4942.8757	-0.0007	10	2	9	2	12	2	11	9	2	8	2	11	2	10
4942.8750	4942.8818	-0.0068	10	2	9	0	10	1	11	9	2	8	0	9	1	10
4942.8750	4942.8556	0.0194	10	4	7	2	11	0	11	9	2	8	2	10	2	10
4942.8750	4942.8773	-0.0023	10	2	9	2	12	1	13	9	2	8	2	11	1	12
4942.8750	4942.8678	0.0072	10	2	9	2	12	2	13	9	2	8	2	11	2	12
4942.8750	4942.8815	-0.0065	10	2	9	1	10	1	11	9	2	8	1	9	1	10
4942.9125	4942.8854	0.0271	10	2	9	2	8	2	7	9	2	8	2	7	2	6
4942.9125	4942.8870	0.0255	10	2	9	2	8	1	9	9	2	8	2	7	1	8
4942.9125	4942.8879	0.0246	10	2	9	2	8	1	7	9	2	8	2	7	1	6
4942.9125	4942.8983	0.0142	10	2	9	2	12	2	12	9	2	8	2	11	2	11
4942.9125	4942.9044	0.0081	10	2	9	1	10	2	10	9	2	8	1	9	2	9
4942.9125	4942.9123	0.0002	10	2	9	2	8	2	8	9	2	8	2	7	2	7
4942.9125	4942.9053	0.0072	10	2	9	0	10	2	10	9	2	8	0	9	2	9
4981.4750	4981.4802	-0.0052	10	4	7	2	12	2	14	9	4	6	2	11	2	13
4981.4750	4981.4779	-0.0029	10	4	7	2	12	1	12	9	4	6	2	11	1	11
4981.4750	4981.4741	0.0009	10	4	7	1	10	1	10	9	4	6	1	9	1	9
4981.4750	4981.4741	0.0009	10	4	7	1	10	0	10	9	4	6	1	9	0	9
4981.4750	4981.4765	-0.0015	10	4	7	0	10	2	12	9	4	6	0	9	2	11
4981.4750	4981.4754	-0.0004	10	4	7	2	12	2	10	9	4	6	2	11	2	9
4981.4750	4981.4741	0.0009	10	4	7	0	10	1	10	9	4	6	0	9	1	9
4981.4750	4981.4740	0.0010	10	4	7	0	10	0	10	9	4	6	0	9	0	9
4981.4750	4981.4778	-0.0028	10	4	7	2	12	0	12	9	4	6	2	11	0	11
4981.4750	4981.4765	-0.0015	10	4	7	1	10	2	12	9	4	6	1	9	2	11
4981.5000	4981.5242	-0.0242	10	4	7	2	12	1	13	9	4	6	2	11	1	12
4981.5000	4981.5227	-0.0227	10	4	7	1	10	1	9	9	4	6	1	9	1	8
4981.5000	4981.5202	-0.0202	10	4	7	0	10	1	11	9	4	6	0	9	1	10
4981.5000	4981.5281	-0.0281	10	4	7	2	12	2	13	9	4	6	2	11	2	12
4981.5000	4981.5226	-0.0226	10	4	7	0	10	1	9	9	4	6	0	9	1	8
4981.5000	4981.5262	-0.0262	10	4	7	2	12	1	11	9	4	6	2	11	1	10
4981.5000	4981.5247	-0.0247	10	4	7	1	10	2	11	9	4	6	1	9	2	10
4981.5000	4981.5215	-0.0215	10	4	7	2	12	2	11	9	4	6	2	11	2	10
4981.5000	4981.5247	-0.0247	10	4	7	0	10	2	11	9	4	6	0	9	2	10
4981.5000	4981.5203	-0.0203	10	4	7	1	10	1	11	9	4	6	1	9	1	10
4981.5625	4981.5465	0.0160	10	4	7	1	11	0	11	9	4	6	1	10	0	10

4981.5625	4981.5567	0.0058	10	4	7	2	11	2	13	9	4	6	2	10	2	12
4981.5625	4981.5444	0.0181	10	4	7	1	11	2	9	9	4	6	1	10	2	8
4981.5625	4981.5492	0.0133	10	4	7	1	9	0	9	9	4	6	1	8	0	8
4981.5625	4981.5487	0.0138	10	4	7	1	11	2	13	9	4	6	1	10	2	12
4981.5625	4981.5464	0.0161	10	4	7	2	9	2	11	9	4	6	2	8	2	10
4981.5625	4981.5465	0.0160	10	4	7	1	11	1	11	9	4	6	1	10	1	10
4981.5625	4981.5516	0.0109	10	4	7	1	9	2	11	9	4	6	1	8	2	10
4981.5625	4981.5541	0.0084	10	6	5	2	11	0	11	9	4	6	2	10	0	10
4981.5625	4981.5541	0.0084	10	4	7	2	11	1	11	9	4	6	2	10	1	10
4981.6000	4981.5952	0.0048	10	4	7	1	9	1	10	9	4	6	1	8	1	9
4981.6000	4981.6003	-0.0003	10	4	7	1	9	2	10	9	4	6	1	8	2	9
4981.6000	4981.5948	0.0052	10	4	7	1	11	1	10	9	4	6	1	10	1	9
4981.6000	4981.5905	0.0095	10	4	7	1	11	2	10	9	4	6	1	10	2	9
4981.6000	4981.5975	0.0025	10	4	7	2	11	2	10	9	4	6	2	10	2	9
4981.6000	4981.5999	0.0001	10	4	7	2	11	1	12	9	4	6	2	10	1	11
4981.6000	4981.6046	-0.0046	10	4	7	2	11	2	12	9	4	6	2	10	2	11
4981.6000	4981.5925	0.0075	10	4	7	1	11	1	12	9	4	6	1	10	1	11
4981.6000	4981.5972	0.0028	10	4	7	1	11	2	12	9	4	6	1	10	2	11
4981.6000	4981.6020	-0.0020	10	4	7	2	11	1	10	9	4	6	2	10	1	9
4981.6750	4981.6439	0.0311	10	4	7	1	9	2	9	9	4	6	1	8	2	8
4981.6750	4981.6411	0.0339	10	4	7	1	11	2	11	9	4	6	1	10	2	10
4981.6750	4981.6477	0.0273	10	2	9	2	11	2	11	9	4	6	2	10	2	10
4981.6750	4981.6383	0.0367	10	4	7	2	9	2	9	9	4	6	2	8	2	8
4981.7000	4981.6672	0.0328	10	4	7	2	10	2	9	9	4	6	2	9	2	8
4981.7000	4981.6689	0.0311	10	4	7	2	10	1	11	9	4	6	2	9	1	10
4981.7000	4981.6745	0.0255	10	4	7	2	10	2	11	9	4	6	2	9	2	10
4981.7000	4981.6712	0.0288	10	4	7	2	10	1	9	9	4	6	2	9	1	8
4982.9219	4982.9296	-0.0077	10	4	6	0	10	2	12	9	4	5	0	9	2	11
4982.9219	4982.9314	-0.0095	10	4	6	2	12	0	12	9	4	5	2	11	0	11
4982.9219	4982.9274	-0.0055	10	4	6	1	10	0	10	9	4	5	1	9	0	9
4982.9219	4982.9293	-0.0074	10	2	8	2	12	2	10	9	4	5	2	11	2	9
4982.9219	4982.9295	-0.0076	10	4	6	1	10	2	12	9	4	5	1	9	2	11
4982.9219	4982.9274	-0.0055	10	4	6	0	10	1	10	9	4	5	0	9	1	9
4982.9219	4982.9336	-0.0117	10	4	6	2	12	2	14	9	4	5	2	11	2	13
4982.9219	4982.9274	-0.0055	10	6	4	0	10	0	10	9	4	5	0	9	0	9
4982.9219	4982.9274	-0.0055	10	4	6	1	10	1	10	9	4	5	1	9	1	9
4982.9219	4982.9315	-0.0096	10	4	6	2	12	1	12	9	4	5	2	11	1	11
4982.9748	4982.9759	-0.0011	10	4	6	0	10	2	11	9	4	5	0	9	2	10
4982.9748	4982.9713	0.0035	10	4	6	1	10	1	11	9	4	5	1	9	1	10
4982.9748	4982.9731	0.0017	10	4	6	2	12	2	11	9	4	5	2	11	2	10
4982.9748	4982.9753	-0.0005	10	4	6	2	12	1	13	9	4	5	2	11	1	12
4982.9748	4982.9758	-0.0010	10	4	6	1	10	2	11	9	4	5	1	9	2	10
4982.9748	4982.9735	0.0013	10	4	6	1	10	1	9	9	4	5	1	9	1	8
4982.9748	4982.9736	0.0012	10	4	6	0	10	1	9	9	4	5	0	9	1	8
4982.9748	4982.9774	-0.0026	10	4	6	2	12	1	11	9	4	5	2	11	1	10
4982.9748	4982.9713	0.0035	10	4	6	0	10	1	11	9	4	5	0	9	1	10
4982.9748	4982.9793	-0.0045	10	4	6	2	12	2	13	9	4	5	2	11	2	12
4983.0250	4983.0175	0.0075	10	4	6	0	10	2	10	9	4	5	0	9	2	9
4983.0250	4983.0085	0.0165	10	4	6	1	11	0	11	9	4	5	1	10	0	10
4983.0250	4983.0166	0.0084	10	6	4	2	11	0	11	9	4	5	2	10	0	10
4983.0250	4983.0189	0.0061	10	4	6	2	11	2	13	9	4	5	2	10	2	12
4983.0250	4983.0166	0.0084	10	4	6	2	11	1	11	9	4	5	2	10	1	10
4983.0250	4983.0145	0.0105	10	4	6	1	9	2	11	9	4	5	1	8	2	10

4983.0250	4983.0107	0.0143	10	4	6	1	11	2	13	9	4	5	1	10	2	12
4983.0250	4983.0065	0.0185	10	2	8	2	9	2	11	9	2	7	2	8	2	10
4983.0250	4983.0086	0.0164	10	4	6	1	11	1	11	9	4	5	1	10	1	10
4983.0250	4983.0210	0.0040	10	4	6	2	12	2	12	9	4	5	2	11	2	11
4983.0694	4983.0653	0.0041	10	4	6	2	11	2	12	9	4	5	2	10	2	11
4983.0694	4983.0546	0.0148	10	4	6	1	11	1	10	9	4	5	1	10	1	9
4983.0694	4983.0564	0.0130	10	4	6	1	9	1	10	9	4	5	1	8	1	9
4983.0694	4983.0567	0.0127	10	4	6	1	11	2	12	9	4	5	1	10	2	11
4983.0694	4983.0604	0.0090	10	4	6	2	11	1	12	9	4	5	2	10	1	11
4983.0694	4983.0499	0.0195	10	4	6	1	11	2	10	9	4	5	1	10	2	9
4983.0694	4983.0628	0.0066	10	4	6	2	11	1	10	9	4	5	2	10	1	9
4983.0694	4983.0581	0.0113	10	4	6	2	11	2	10	9	4	5	2	10	2	9
4983.0694	4983.0485	0.0209	10	4	6	2	9	1	10	9	4	5	2	8	1	9
4983.0694	4983.0525	0.0169	10	4	6	1	11	1	12	9	4	5	1	10	1	11
4983.1016	4983.0957	0.0059	10	4	6	2	10	2	12	9	4	5	2	9	2	11
4983.1016	4983.1061	-0.0045	10	2	8	2	11	2	11	9	4	5	2	10	2	10
4983.1016	4983.0937	0.0079	10	2	8	2	10	0	10	9	2	7	2	9	0	9
4983.1016	4983.0951	0.0065	10	6	4	2	9	2	9	9	6	3	2	8	2	8
4983.1016	4983.0916	0.0100	10	4	6	2	10	2	8	9	4	5	2	9	2	7
4983.1016	4983.0936	0.0080	10	4	6	2	10	1	10	9	4	5	2	9	1	9
4983.1016	4983.1025	-0.0009	10	4	6	1	9	2	9	9	4	5	1	8	2	8
4983.1016	4983.0985	0.0031	10	4	6	1	11	2	11	9	4	5	1	10	2	10
4983.1375	4983.1399	-0.0024	10	4	6	2	10	1	9	9	4	5	2	9	1	8
4983.1375	4983.1377	-0.0002	10	4	6	2	10	1	11	9	4	5	2	9	1	10
4983.1375	4983.1423	-0.0048	10	6	4	2	10	2	11	9	6	3	2	9	2	10
4983.1375	4983.1353	0.0022	10	2	8	2	10	2	9	9	2	7	2	9	2	8
4983.1664	4983.1838	-0.0174	10	2	8	2	10	2	10	9	4	5	2	9	2	9
5004.7531	5004.7650	-0.0119	10	3	7	2	12	1	12	9	3	6	2	11	1	11
5004.7531	5004.7653	-0.0122	10	3	7	2	12	0	12	9	3	6	2	11	0	11
5004.7531	5004.7587	-0.0056	10	3	7	1	10	2	12	9	3	6	1	9	2	11
5004.7531	5004.7705	-0.0174	10	3	7	2	12	2	13	9	3	6	2	11	2	12
5004.7531	5004.7777	-0.0246	10	3	7	2	12	2	12	9	3	6	2	11	2	11
5004.7531	5004.7706	-0.0175	10	3	7	2	12	1	11	9	3	6	2	11	1	10
5004.7531	5004.7683	-0.0152	10	3	7	0	10	1	11	9	3	6	0	9	1	10
5004.7531	5004.7594	-0.0063	10	3	7	0	10	2	12	9	3	6	0	9	2	11
5004.7531	5004.7633	-0.0102	10	3	7	2	12	2	14	9	3	6	2	11	2	13
5004.7531	5004.7709	-0.0178	10	3	7	2	12	1	13	9	3	6	2	11	1	12
5004.8809	5004.8815	-0.0006	10	3	7	1	11	0	11	9	3	6	1	10	0	10
5004.8809	5004.8831	-0.0022	10	3	7	1	9	2	11	9	3	6	1	8	2	10
5004.8809	5004.8925	-0.0116	10	3	7	1	11	2	11	9	3	6	1	10	2	10
5004.8809	5004.8995	-0.0186	10	3	7	2	11	1	12	9	3	6	2	10	1	11
5004.8809	5004.8988	-0.0179	10	3	7	2	11	2	12	9	3	6	2	10	2	11
5004.8809	5004.8813	-0.0004	10	3	7	1	11	1	11	9	3	6	1	10	1	10
5004.8809	5004.8803	0.0006	10	3	7	1	11	2	13	9	3	6	1	10	2	12
5004.8809	5004.8878	-0.0069	10	3	7	2	11	2	13	9	3	6	2	10	2	12
5004.8809	5004.8879	-0.0070	10	3	7	1	11	1	12	9	3	6	1	10	1	11
5004.8809	5004.8844	-0.0035	10	3	7	1	11	2	12	9	3	6	1	10	2	11
5057.9047	5057.9030	0.0017	10	2	8	2	12	1	12	9	2	7	2	11	1	11
5057.9047	5057.9032	0.0015	10	2	8	2	12	0	12	9	2	7	2	11	0	11
5057.9047	5057.9066	-0.0019	10	2	8	1	10	2	12	9	2	7	1	9	2	11
5057.9047	5057.9089	-0.0042	10	2	8	0	10	2	12	9	2	7	0	9	2	11
5057.9047	5057.9015	0.0032	10	2	8	2	12	2	14	9	2	7	2	11	2	13
5057.9047	5057.9024	0.0023	10	2	8	2	12	1	13	9	2	7	2	11	1	12

5057.9047	5057.8999	0.0048	10	2	8	2	12	2	13	9	2	7	2	11	2	12
5057.9047	5057.9009	0.0038	10	2	8	2	12	1	11	9	2	7	2	11	1	10
5057.9047	5057.9010	0.0037	10	2	8	2	12	2	12	9	2	7	2	11	2	11
5057.9047	5057.9086	-0.0039	10	2	8	1	10	1	11	9	2	7	1	9	1	10
5057.9625	5057.9620	0.0005	10	2	8	2	11	2	13	9	2	7	2	10	2	12
5057.9625	5057.9769	-0.0144	10	2	8	1	11	1	11	9	2	7	1	10	1	10
5057.9625	5057.9772	-0.0147	10	2	8	1	11	0	11	9	2	7	1	10	0	10
5057.9625	5057.9661	-0.0036	10	2	8	1	9	2	11	9	2	7	1	8	2	10
5057.9625	5057.9742	-0.0117	10	2	8	1	11	2	11	9	2	7	1	10	2	10
5057.9625	5057.9729	-0.0104	10	2	8	1	11	2	12	9	2	7	1	10	2	11
5057.9625	5057.9753	-0.0128	10	2	8	1	11	2	13	9	2	7	1	10	2	12
5057.9625	5057.9625	0.0000	10	2	8	2	11	2	12	9	2	7	2	10	2	11
5057.9625	5057.9763	-0.0138	10	2	8	1	11	1	12	9	2	7	1	10	1	11
5057.9625	5057.9651	-0.0026	10	2	8	2	11	1	12	9	2	7	2	10	1	11
5058.0419	5058.0374	0.0045	10	2	8	2	10	2	12	9	2	7	2	9	2	11
5058.0419	5058.0410	0.0009	10	2	8	2	10	1	11	9	2	7	2	9	1	10
5058.0419	5058.0431	-0.0012	10	2	8	2	10	2	9	9	0	9	2	9	2	8
5058.0419	5058.0417	0.0002	10	2	8	2	10	2	8	9	2	7	2	9	2	7
5058.0419	5058.0391	0.0028	10	2	8	2	10	1	10	9	2	7	2	9	1	9
5058.0419	5058.0408	0.0011	10	0	10	2	10	2	10	9	2	7	2	9	2	9
5058.0419	5058.0371	0.0048	10	4	6	2	10	2	11	9	2	7	2	9	2	10
5058.0419	5058.0395	0.0024	10	2	8	2	10	0	10	9	0	9	2	9	0	9
5058.0419	5058.0389	0.0030	10	2	8	2	10	1	9	9	2	7	2	9	1	8
5326.0988	5326.1067	-0.0079	11	0	11	2	11	2	13	10	0	10	2	10	2	12
5326.0988	5326.1113	-0.0125	11	0	11	2	11	0	11	10	0	10	2	10	0	10
5326.0988	5326.1157	-0.0169	11	0	11	2	11	2	9	10	0	10	2	10	2	8
5326.0988	5326.1102	-0.0114	11	0	11	2	11	1	11	10	0	10	2	10	1	10
5326.1250	5326.1604	-0.0354	11	0	11	2	10	2	12	10	2	8	2	10	2	11
5326.1250	5326.1522	-0.0272	11	0	11	2	11	1	12	10	0	10	2	10	1	11
5326.1250	5326.1651	-0.0401	11	0	11	2	10	1	10	10	0	10	2	10	1	9
5326.1989	5326.2251	-0.0262	11	2	9	2	10	2	11	10	2	8	2	9	2	10
5326.1989	5326.1986	0.0003	11	0	11	2	12	0	12	10	0	10	2	11	0	11
5326.1989	5326.1935	0.0054	11	0	11	2	12	1	12	10	0	10	2	11	1	11
5326.1989	5326.1850	0.0139	11	0	11	2	11	2	12	10	0	10	2	10	2	11
5326.1989	5326.2004	-0.0015	11	0	11	2	12	2	14	10	0	10	2	11	2	13
5326.1989	5326.2110	-0.0121	11	0	11	1	12	1	12	10	0	10	1	11	1	11
5326.1989	5326.2234	-0.0245	11	0	11	2	9	2	11	10	0	10	2	8	2	10
5326.1989	5326.2072	-0.0083	11	0	11	1	12	2	14	10	0	10	1	11	2	13
5326.1989	5326.2044	-0.0055	11	0	11	1	10	2	12	10	0	10	1	9	2	11
5326.1989	5326.2116	-0.0127	11	0	11	1	12	0	12	10	0	10	1	11	0	11
5326.2250	5326.2474	-0.0224	11	0	11	2	12	1	11	10	0	10	2	11	1	10
5326.2250	5326.2604	-0.0354	11	0	11	1	12	2	11	10	0	10	1	11	2	10
5326.2250	5326.2411	-0.0161	11	0	11	1	12	1	13	10	0	10	1	11	1	12
5326.2250	5326.2441	-0.0191	11	0	11	1	12	2	13	10	0	10	1	11	2	12
5326.2250	5326.2542	-0.0292	11	0	11	2	12	2	13	10	0	10	2	11	2	12
5326.2250	5326.2452	-0.0202	11	0	11	1	12	1	11	10	0	10	1	11	1	10
5326.2250	5326.2537	-0.0287	11	0	11	1	10	1	11	10	0	10	1	9	1	10
5326.2250	5326.2400	-0.0150	11	0	11	1	10	2	11	10	0	10	1	9	2	10
5326.2250	5326.2506	-0.0256	11	0	11	2	10	1	11	10	0	10	2	9	1	10
5326.2750	5326.3011	-0.0261	11	0	11	2	13	2	15	10	0	10	2	12	2	14
5326.2750	5326.2901	-0.0151	11	0	11	2	13	0	13	10	0	10	2	12	0	12
5326.2750	5326.2920	-0.0170	11	0	11	1	11	2	13	10	0	10	1	10	2	12
5326.2750	5326.2914	-0.0164	11	0	11	0	11	2	13	10	0	10	0	10	2	12

5326.2750	5326.2636	0.0114	11	0	11	2	13	1	13	10	0	10	2	11	1	12
5326.2750	5326.2767	-0.0017	11	0	11	2	12	1	13	10	0	10	2	12	1	12
5326.2750	5326.3055	-0.0305	11	0	11	0	11	2	12	10	0	10	0	10	2	11
5326.2750	5326.2940	-0.0190	11	0	11	1	11	2	12	10	0	10	1	11	2	11
5326.3250	5326.3324	-0.0074	11	0	11	1	11	1	10	10	0	10	1	10	1	9
5326.3250	5326.3359	-0.0109	11	0	11	0	11	1	12	10	0	10	0	10	1	11
5326.3250	5326.3350	-0.0100	11	0	11	2	13	1	14	10	0	10	2	12	1	13
5326.3250	5326.3386	-0.0136	11	0	11	2	9	1	10	10	0	10	2	8	1	9
5326.3250	5326.3040	0.0210	11	0	11	1	12	2	12	10	0	10	1	10	2	11
5326.3250	5326.3374	-0.0124	11	0	11	1	11	1	12	10	0	10	1	10	1	11
5326.3250	5326.3055	0.0195	11	0	11	2	12	2	12	10	0	10	2	9	2	11
5326.3250	5326.3243	0.0007	11	0	11	2	13	2	12	10	0	10	2	12	2	11
5326.3250	5326.3279	-0.0029	11	0	11	2	13	2	14	10	0	10	2	12	2	13
5326.3250	5326.3315	-0.0065	11	0	11	2	13	1	12	10	0	10	2	12	1	11
5326.3750	5326.3480	0.0270	11	0	11	2	9	2	8	10	0	10	2	8	2	7
5326.3750	5326.3673	0.0077	11	0	11	2	9	2	9	10	0	10	2	8	2	8
5326.3750	5326.3650	0.0100	11	0	11	0	11	2	11	10	0	10	0	10	2	10
5326.3750	5326.3630	0.0120	11	0	11	2	13	2	13	10	0	10	2	12	2	12
5326.3750	5326.3656	0.0094	11	0	11	1	11	2	11	10	0	10	1	10	2	10
5430.8875	5430.8700	0.0175	11	2	10	2	12	1	12	10	2	9	2	11	1	11
5430.8875	5430.8722	0.0153	11	2	10	1	10	2	12	10	2	9	1	9	2	11
5430.8875	5430.8791	0.0084	11	2	10	1	12	1	12	10	2	9	1	11	1	11
5430.8875	5430.8763	0.0112	11	2	10	1	12	2	14	10	2	9	1	11	2	13
5430.8875	5430.8689	0.0186	11	2	10	2	12	2	14	10	2	9	2	11	2	13
5430.8875	5430.8708	0.0167	11	2	10	2	12	0	12	10	2	9	2	11	0	11
5430.8875	5430.8797	0.0078	11	2	10	1	12	0	12	10	2	9	1	11	0	11
5430.9125	5430.8955	0.0170	11	2	10	2	12	1	13	10	2	9	2	11	1	12
5430.9125	5430.9037	0.0088	11	2	10	1	12	1	11	10	2	9	1	11	1	10
5430.9125	5430.9029	0.0096	11	2	10	1	12	2	13	10	2	9	1	11	2	12
5430.9125	5430.9058	0.0067	11	2	10	1	12	1	13	10	2	9	1	11	1	12
5430.9125	5430.9106	0.0019	11	2	10	2	10	1	11	10	2	9	2	9	1	10
5430.9125	5430.8991	0.0134	11	2	10	1	10	2	11	10	2	9	1	9	2	10
5430.9125	5430.9000	0.0125	11	2	10	1	10	1	11	10	2	9	1	9	1	10
5430.9125	5430.8836	0.0289	11	2	10	2	10	2	12	10	2	9	2	9	2	11
5430.9125	5430.8944	0.0181	11	2	10	2	12	2	13	10	2	9	2	11	2	12
5430.9250	5430.9204	0.0046	11	4	8	2	12	0	12	10	2	9	2	11	2	11
5430.9250	5430.9453	-0.0203	11	2	10	2	13	1	12	10	2	9	2	12	1	11
5430.9250	5430.9206	0.0044	11	2	10	2	13	2	15	10	2	9	2	12	2	14
5430.9250	5430.9428	-0.0178	11	2	10	2	13	2	14	10	2	9	2	12	2	13
5430.9250	5430.9253	-0.0003	11	2	10	1	11	2	13	10	2	9	1	10	2	12
5430.9250	5430.9223	0.0027	11	2	10	2	13	0	13	10	2	9	2	12	0	12
5430.9250	5430.9258	-0.0008	11	2	10	0	11	2	13	10	2	9	0	10	2	12
5430.9250	5430.9216	0.0034	11	2	10	2	13	1	13	10	2	9	2	12	1	12
5430.9250	5430.9331	-0.0081	11	2	10	1	12	2	12	10	2	9	1	11	2	11
5430.9250	5430.9310	-0.0060	11	2	10	2	10	2	11	10	2	9	2	9	2	10
5430.9500	5430.9498	0.0002	11	2	10	2	13	2	12	10	2	9	2	12	2	11
5430.9500	5430.9550	-0.0050	11	2	10	0	11	1	12	10	2	9	0	10	1	11
5430.9500	5430.9501	-0.0001	11	2	10	2	13	1	14	10	2	9	2	12	1	13
5430.9500	5430.9546	-0.0046	11	2	10	1	11	1	12	10	2	9	1	10	1	11
5430.9500	5430.9601	-0.0101	11	2	10	2	9	1	10	10	2	9	2	8	1	9
5430.9500	5430.9481	0.0019	11	2	10	1	11	2	12	10	2	9	1	10	2	11
5430.9500	5430.9487	0.0013	11	2	10	0	11	2	12	10	2	9	0	10	2	11
5430.9750	5430.9775	-0.0025	11	2	10	1	11	2	11	10	2	9	1	10	2	10

5430.9750	5430.9721	0.0029	11	2	10	2	13	2	13	10	2	9	2	12	2	12
5430.9750	5430.9600	0.0150	11	2	10	2	9	1	8	10	2	9	2	8	1	7
5430.9750	5430.9781	-0.0031	11	2	10	0	11	2	11	10	2	9	0	10	2	10
5430.9750	5430.9842	-0.0092	11	2	10	2	9	2	9	10	2	9	2	8	2	8
5430.9750	5430.9601	0.0149	11	2	10	2	9	2	8	10	2	9	2	8	2	7
5477.1580	5477.1510	0.0070	11	3	9	0	11	2	13	10	5	6	0	10	2	12
5477.1580	5477.1477	0.0103	11	5	7	1	11	1	11	10	5	6	1	10	1	10
5477.1580	5477.1475	0.0105	11	3	9	0	11	1	11	10	5	6	0	10	1	10
5477.1580	5477.1475	0.0105	11	5	7	1	11	0	11	10	5	6	1	10	0	10
5477.1580	5477.1473	0.0107	11	5	7	0	11	0	11	10	7	4	0	10	0	10
5477.1580	5477.1519	0.0061	11	5	7	2	13	0	13	10	5	6	2	12	0	12
5477.1580	5477.1484	0.0096	11	5	7	2	13	2	11	10	5	6	2	12	2	10
5477.1580	5477.1555	0.0025	11	5	7	2	13	2	15	10	5	6	2	12	2	14
5477.1580	5477.1521	0.0059	11	5	7	2	13	1	13	10	5	6	2	12	1	12
5477.1580	5477.1513	0.0067	11	5	7	1	11	2	13	10	5	6	1	10	2	12
5477.2500	5477.2541	-0.0041	11	5	6	2	13	1	13	10	5	5	2	12	1	12
5477.2500	5477.2077	0.0423	11	5	7	2	13	1	14	10	5	6	2	12	1	13
5477.2500	5477.2278	0.0222	11	5	7	1	12	2	14	10	5	6	1	11	2	13
5477.2500	5477.2349	0.0151	11	5	7	2	12	0	12	10	5	6	2	11	0	11
5477.2500	5477.2391	0.0109	11	5	7	2	12	2	14	10	5	6	2	11	2	13
5477.2500	5477.2102	0.0398	11	5	7	2	13	1	12	10	5	6	2	12	1	11
5477.2500	5477.2248	0.0252	11	5	7	1	12	0	12	10	5	6	1	11	0	11
5477.2500	5477.2249	0.0251	11	5	7	1	12	1	12	10	5	6	1	11	1	11
5477.2500	5477.2024	0.0476	11	5	7	0	11	1	12	10	5	6	0	10	1	11
5477.2500	5477.2532	-0.0032	11	5	6	1	11	2	13	10	5	5	1	10	2	12
5477.2500	5477.2529	-0.0029	11	5	6	0	11	2	13	10	5	5	0	10	2	12
5477.2500	5477.2575	-0.0075	11	5	6	2	13	2	15	10	5	5	2	12	2	14
5477.2500	5477.2539	-0.0039	11	5	6	2	13	0	13	10	5	5	2	12	0	12
5477.2500	5477.2352	0.0148	11	5	7	2	12	1	12	10	5	6	2	11	1	11
5477.2500	5477.2026	0.0474	11	5	7	1	11	1	12	10	5	6	1	10	1	11
5477.2500	5477.2338	0.0162	11	5	7	1	10	2	12	10	5	6	1	9	2	11
5477.2500	5477.2124	0.0376	11	5	7	2	13	2	14	10	5	6	2	12	2	13
5477.3000	5477.3120	-0.0120	11	5	6	2	13	1	12	10	5	5	2	12	1	11
5477.3000	5477.3095	-0.0095	11	5	6	2	13	1	14	10	5	5	2	12	1	13
5477.3000	5477.3142	-0.0142	11	5	6	2	13	2	14	10	5	5	2	12	2	13
5477.3000	5477.3275	-0.0275	11	5	6	1	12	1	12	10	5	5	1	11	1	11
5477.3000	5477.3042	-0.0042	11	5	6	0	11	1	12	10	5	5	0	10	1	11
5477.3000	5477.2837	0.0163	11	5	7	2	12	1	13	10	5	6	2	11	1	12
5477.3000	5477.2939	0.0061	11	5	7	2	12	2	13	10	5	6	2	11	2	12
5477.3000	5477.3124	-0.0124	11	7	5	2	11	2	13	10	5	6	2	10	2	12
5477.3000	5477.2869	0.0131	11	5	7	1	12	2	13	10	5	6	1	11	2	12
5477.3000	5477.3274	-0.0274	11	5	6	1	12	0	12	10	5	5	1	11	0	11
5477.3864	5477.3868	-0.0004	11	5	6	1	10	1	11	10	5	5	1	9	1	10
5477.3864	5477.3932	-0.0068	11	5	6	2	12	1	11	10	5	5	2	11	1	10
5477.3864	5477.3824	0.0040	11	5	6	1	12	1	13	10	5	5	1	11	1	12
5477.3864	5477.3917	-0.0053	11	5	6	1	12	1	11	10	5	5	1	11	1	10
5477.3864	5477.3966	-0.0102	11	5	6	2	12	2	13	10	5	5	2	11	2	12
5477.3864	5477.3699	0.0165	11	7	5	2	11	2	12	10	7	4	2	10	2	11
5477.3864	5477.3714	0.0150	11	5	6	2	13	2	13	10	5	5	2	12	2	12
5477.3864	5477.3894	-0.0030	11	5	6	1	12	2	13	10	5	5	1	11	2	12
5477.3864	5477.3912	-0.0048	11	5	6	2	12	1	13	10	5	5	2	11	1	12
5477.3864	5477.3801	0.0063	11	5	6	1	12	2	11	10	5	5	1	11	2	10
5481.8000	5481.8041	-0.0041	11	4	8	1	11	0	11	10	4	7	1	10	0	10

5481.8000	5481.8046	-0.0046	11	4	8	2	13	2	11	10	4	7	2	12	2	10
5481.8000	5481.8043	-0.0043	11	4	8	0	11	1	11	10	4	7	0	10	1	10
5481.8000	5481.8054	-0.0054	11	4	8	2	13	1	13	10	4	7	2	12	1	12
5481.8000	5481.8055	-0.0055	11	4	8	2	13	0	13	10	4	7	2	12	0	12
5481.8000	5481.8039	-0.0039	11	4	8	1	11	1	11	10	4	7	1	10	1	10
5481.8000	5481.8064	-0.0064	11	4	8	2	13	2	15	10	4	7	2	12	2	14
5481.8000	5481.8044	-0.0044	11	6	6	0	11	0	11	10	4	7	0	10	0	10
5481.8000	5481.8048	-0.0048	11	4	8	1	11	2	13	10	4	7	1	10	2	12
5481.8000	5481.8052	-0.0052	11	4	8	0	11	2	13	10	4	7	0	10	2	12
5481.8250	5481.8380	-0.0130	11	4	8	1	11	1	12	10	4	7	1	10	1	11
5481.8250	5481.8393	-0.0143	11	4	8	0	11	1	10	10	4	7	0	10	1	9
5481.8250	5481.8412	-0.0162	11	4	8	2	13	2	14	10	4	7	2	12	2	13
5481.8250	5481.8402	-0.0152	11	4	8	2	13	1	12	10	4	7	2	12	1	11
5481.8250	5481.8383	-0.0133	11	4	8	0	11	1	12	10	4	7	0	10	1	11
5481.8250	5481.8390	-0.0140	11	4	8	2	13	1	14	10	4	7	2	12	1	13
5481.8250	5481.8384	-0.0134	11	4	8	2	13	2	12	10	4	7	2	12	2	11
5481.8250	5481.8408	-0.0158	11	4	8	0	11	2	12	10	4	7	0	10	2	11
5481.8250	5481.8404	-0.0154	11	4	8	1	11	2	12	10	4	7	1	10	2	11
5481.8250	5481.8388	-0.0138	11	4	8	1	11	1	10	10	4	7	1	10	1	9
5481.8750	5481.8658	0.0092	11	6	6	2	12	0	12	10	4	7	2	11	0	11
5481.8750	5481.8630	0.0120	11	4	8	1	12	2	10	10	4	7	1	11	2	9
5481.8750	5481.8659	0.0091	11	4	8	1	10	0	10	10	4	7	1	9	0	9
5481.8750	5481.8662	0.0088	11	4	8	1	10	2	12	10	4	7	1	9	2	11
5481.8750	5481.8621	0.0129	11	4	8	2	10	2	12	10	4	7	2	9	2	11
5481.8750	5481.8658	0.0092	11	4	8	2	12	1	12	10	4	7	2	11	1	11
5481.8750	5481.8668	0.0082	11	4	8	2	12	2	14	10	4	7	2	11	2	13
5481.8750	5481.8650	0.0100	11	4	8	1	12	2	14	10	4	7	1	11	2	13
5481.8750	5481.8638	0.0112	11	4	8	1	12	0	12	10	4	7	1	11	0	11
5481.8750	5481.8638	0.0112	11	4	8	1	12	1	12	10	4	7	1	11	1	11
5481.9000	5481.9033	-0.0033	11	4	8	2	12	2	13	10	4	7	2	11	2	12
5481.9000	5481.9019	-0.0019	11	4	8	2	12	1	11	10	4	7	2	11	1	10
5481.9000	5481.8987	0.0013	11	4	8	1	12	1	11	10	4	7	1	11	1	10
5481.9000	5481.8997	0.0003	11	4	8	1	10	1	11	10	4	7	1	9	1	10
5481.9000	5481.8978	0.0022	11	4	8	1	12	1	13	10	4	7	1	11	1	12
5481.9000	5481.8996	0.0004	11	4	8	1	12	2	13	10	4	7	1	11	2	12
5481.9000	5481.9003	-0.0003	11	4	8	2	12	1	13	10	4	7	2	11	1	12
5481.9000	5481.8960	0.0040	11	4	8	1	12	2	11	10	4	7	1	11	2	10
5481.9000	5481.8991	0.0009	11	4	8	2	12	2	11	10	4	7	2	11	2	10
5481.9000	5481.9012	-0.0012	11	4	8	1	10	2	11	10	4	7	1	9	2	10
5481.9750	5481.9588	0.0162	11	4	8	2	11	1	12	10	6	5	0	10	1	11
5481.9750	5481.9604	0.0146	11	4	8	2	11	2	12	10	4	7	2	10	2	11
5481.9750	5481.9596	0.0154	11	4	8	2	11	1	10	10	4	7	2	10	1	9
5481.9750	5481.9569	0.0181	11	2	10	2	11	2	10	10	4	7	2	10	2	9
5484.6500	5484.6757	-0.0257	11	4	7	2	13	2	11	10	2	8	2	12	2	10
5484.6500	5484.6740	-0.0240	11	4	7	1	11	2	13	10	4	6	1	10	2	12
5484.6500	5484.6734	-0.0234	11	4	7	0	11	1	11	10	4	6	0	10	1	10
5484.6500	5484.6735	-0.0235	11	4	7	1	11	0	11	10	4	6	1	10	0	10
5484.6500	5484.6733	-0.0233	11	4	7	1	11	1	11	10	4	6	1	10	1	10
5484.6500	5484.6769	-0.0269	11	4	7	2	13	2	15	10	4	6	2	12	2	14
5484.6500	5484.6763	-0.0263	11	4	7	2	13	0	13	10	4	6	2	12	0	12
5484.6500	5484.6735	-0.0235	11	6	5	0	11	0	11	10	4	6	0	10	0	10
5484.6500	5484.6741	-0.0241	11	4	7	0	11	2	13	10	4	6	0	10	2	12
5484.6500	5484.6762	-0.0262	11	4	7	2	13	1	13	10	4	6	2	12	1	12

5484.6750	5484.7062	-0.0312	11	4	7	2	13	2	12	10	4	6	2	12	2	11
5484.6750	5484.7038	-0.0288	11	4	7	1	11	1	12	10	4	6	1	10	1	11
5484.6750	5484.7074	-0.0324	11	4	7	2	13	1	12	10	4	6	2	12	1	11
5484.6750	5484.7044	-0.0294	11	4	7	1	11	1	10	10	4	6	1	10	1	9
5484.6750	5484.7038	-0.0288	11	4	7	0	11	1	12	10	4	6	0	10	1	11
5484.6750	5484.7086	-0.0336	11	4	7	2	13	2	14	10	4	6	2	12	2	13
5484.6750	5484.7062	-0.0312	11	4	7	2	13	1	14	10	4	6	2	12	1	13
5484.6750	5484.7065	-0.0315	11	4	7	0	11	2	12	10	4	6	0	10	2	11
5484.6750	5484.7062	-0.0312	11	4	7	1	11	2	12	10	4	6	1	10	2	11
5484.6750	5484.7047	-0.0297	11	4	7	0	11	1	10	10	4	6	0	10	1	9
5484.7250	5484.7361	-0.0111	11	4	7	0	11	2	11	10	4	6	0	10	2	10
5484.7250	5484.7474	-0.0224	11	4	7	1	12	0	12	10	4	6	1	11	0	11
5484.7250	5484.7375	-0.0125	11	4	7	2	13	2	13	10	4	6	2	12	2	12
5484.7250	5484.7486	-0.0236	11	4	7	1	12	2	14	10	4	6	1	11	2	13
5484.7250	5484.7527	-0.0277	11	4	7	2	12	2	14	10	4	6	2	11	2	13
5484.7250	5484.7444	-0.0194	11	2	9	2	10	2	12	10	2	8	2	9	2	11
5484.7250	5484.7500	-0.0250	11	4	7	1	10	2	12	10	4	6	1	9	2	11
5484.7250	5484.7522	-0.0272	11	6	5	2	12	0	12	10	6	4	2	11	0	11
5484.7250	5484.7521	-0.0271	11	4	7	2	12	1	12	10	4	6	2	11	1	11
5484.7250	5484.7475	-0.0225	11	4	7	1	12	1	12	10	4	6	1	11	1	11
5484.7625	5484.7804	-0.0179	11	4	7	1	10	1	11	10	4	6	1	9	1	10
5484.7625	5484.7865	-0.0240	11	4	7	2	12	2	13	10	4	6	2	11	2	12
5484.7625	5484.7761	-0.0136	11	4	7	2	10	1	11	10	4	6	2	9	1	10
5484.7625	5484.7848	-0.0223	11	4	7	2	12	1	11	10	4	6	2	11	1	10
5484.7625	5484.7759	-0.0134	11	4	7	1	12	2	11	10	4	6	1	11	2	10
5484.7625	5484.7783	-0.0158	11	4	7	1	12	1	11	10	4	6	1	11	1	10
5484.7625	5484.7778	-0.0153	11	4	7	1	12	1	13	10	4	6	1	11	1	12
5484.7625	5484.7791	-0.0166	11	4	7	1	12	2	13	10	4	6	1	11	2	12
5484.7625	5484.7834	-0.0209	11	4	7	2	12	1	13	10	4	6	2	11	1	12
5484.7625	5484.7815	-0.0190	11	4	7	1	10	2	11	10	4	6	1	9	2	10
5484.8000	5484.8234	-0.0234	11	2	9	2	11	0	11	10	4	6	2	10	0	10
5484.8000	5484.8231	-0.0231	11	4	7	2	11	1	11	10	4	6	2	10	1	10
5484.8000	5484.8112	-0.0112	11	4	7	1	10	2	10	10	6	4	1	9	0	9
5484.8000	5484.8154	-0.0154	11	2	9	2	12	2	12	10	0	10	2	11	2	11
5484.8000	5484.8233	-0.0233	11	4	7	2	11	2	9	10	4	6	2	10	2	8
5484.8000	5484.8085	-0.0085	11	4	7	1	12	2	12	10	4	6	1	11	2	11
5484.8000	5484.8090	-0.0090	11	6	5	2	10	2	10	10	6	4	2	9	2	9
5484.8000	5484.8233	-0.0233	11	4	7	2	11	2	13	10	4	6	2	10	2	12
5484.8375	5484.8556	-0.0181	11	6	5	2	11	2	12	10	8	2	2	10	2	11
5484.8375	5484.8552	-0.0177	11	4	7	2	11	1	12	10	4	6	2	10	1	11
5484.8375	5484.8558	-0.0183	11	4	7	2	11	1	10	10	4	6	2	10	1	9
5484.8375	5484.8530	-0.0155	11	2	9	2	11	2	10	10	2	8	2	10	2	9
5484.8625	5484.8863	-0.0238	11	4	7	2	11	2	11	10	2	8	2	10	2	10
5515.6463	5515.6527	-0.0064	11	3	8	2	13	1	14	10	3	7	2	12	1	13
5515.6463	5515.6550	-0.0087	11	3	8	2	13	2	15	10	3	7	2	12	2	14
5515.6463	5515.6497	-0.0034	11	3	8	1	11	2	13	10	3	7	1	10	2	12
5515.6463	5515.6505	-0.0042	11	3	8	2	13	2	14	10	3	7	2	12	2	13
5515.6463	5515.6570	-0.0107	11	3	8	2	13	1	13	10	3	7	2	12	1	12
5515.6463	5515.6573	-0.0110	11	3	8	2	13	0	13	10	3	7	2	12	0	12
5515.6463	5515.6488	-0.0025	11	3	8	1	11	1	12	10	3	7	1	10	1	11
5515.6463	5515.6498	-0.0035	11	3	8	0	11	2	13	10	3	7	0	10	2	12
5515.6463	5515.6481	-0.0018	11	3	8	2	13	2	13	10	3	7	2	12	2	12
5515.6463	5515.6514	-0.0051	11	3	8	2	13	1	12	10	3	7	2	12	1	11

5515.7727	5515.7745	-0.0018	11	3	8	1	12	1	13	10	3	7	1	11	1	12
5515.7727	5515.7784	-0.0057	11	3	8	1	12	1	12	10	3	7	1	11	1	11
5515.7727	5515.7681	0.0046	11	3	8	1	12	2	12	10	3	7	1	11	2	11
5515.7727	5515.7830	-0.0103	11	3	8	2	12	1	13	10	3	7	2	11	1	12
5515.7727	5515.7801	-0.0074	11	3	8	2	12	2	13	10	3	7	2	11	2	12
5515.7727	5515.7819	-0.0092	11	3	8	2	12	2	14	10	3	7	2	11	2	13
5515.7727	5515.7767	-0.0040	11	3	8	1	12	2	14	10	3	7	1	11	2	13
5515.7727	5515.7698	0.0029	11	3	8	1	12	2	13	10	3	7	1	11	2	12
5515.7727	5515.7784	-0.0057	11	3	8	1	10	2	12	10	3	7	1	9	2	11
5515.7727	5515.7786	-0.0059	11	3	8	1	12	0	12	10	3	7	1	11	0	11
5515.9016	5515.9053	-0.0037	11	3	8	2	11	1	12	10	3	7	2	10	1	11
5515.9016	5515.8987	0.0029	11	3	8	2	11	2	12	10	3	7	2	10	2	11
5515.9016	5515.9093	-0.0077	11	3	8	2	11	2	9	10	3	7	2	10	2	8
5515.9016	5515.9058	-0.0042	11	3	8	2	11	1	11	10	3	7	2	10	1	10
5515.9016	5515.9033	-0.0017	11	3	8	2	11	2	13	10	3	7	2	10	2	12
5515.9016	5515.9062	-0.0046	11	3	8	2	11	0	11	10	3	7	2	10	0	10
5515.9016	5515.9002	0.0014	11	3	8	2	11	2	11	10	3	7	2	10	2	10
5515.9016	5515.9025	-0.0009	11	3	8	2	11	1	10	10	3	7	2	10	1	9
5515.9016	5515.9057	-0.0041	11	3	8	2	11	2	10	10	3	7	2	10	2	9
5567.3750	5567.3914	-0.0164	11	2	9	2	13	2	14	10	2	8	2	12	2	13
5567.3750	5567.4029	-0.0279	11	2	9	0	11	1	12	10	2	8	0	10	1	11
5567.3750	5567.3953	-0.0203	11	2	9	1	11	2	13	10	2	8	1	10	2	12
5567.3750	5567.3889	-0.0139	11	2	9	2	13	1	13	10	2	8	2	12	1	12
5567.3750	5567.3929	-0.0179	11	2	9	2	13	1	14	10	2	8	2	12	1	13
5567.3750	5567.4009	-0.0259	11	2	9	1	11	1	12	10	2	8	1	10	1	11
5567.3750	5567.3973	-0.0223	11	2	9	0	11	2	13	10	2	8	0	10	2	12
5567.3750	5567.3964	-0.0214	11	2	9	2	13	2	13	10	2	8	2	12	2	12
5567.3750	5567.3891	-0.0141	11	2	9	2	13	0	13	10	2	8	2	12	0	12
5567.3750	5567.3920	-0.0170	11	2	9	2	13	1	12	10	2	8	2	12	1	11
5567.4000	5567.4178	-0.0178	11	2	9	2	12	2	13	10	2	8	2	11	2	12
5567.4000	5567.4195	-0.0195	11	2	9	2	12	1	13	10	2	8	2	11	1	12
5567.4000	5567.4220	-0.0220	11	2	9	1	10	2	11	10	2	8	1	9	2	10
5567.4000	5567.4191	-0.0191	11	2	9	1	10	2	12	10	2	8	1	9	2	11
5567.4000	5567.4131	-0.0131	11	2	9	2	12	2	14	10	2	8	2	11	2	13
5567.4000	5567.4186	-0.0186	11	2	9	2	12	1	11	10	2	8	2	11	1	10
5567.4000	5567.4140	-0.0140	11	2	9	2	12	1	12	10	2	8	2	11	1	11
5567.4000	5567.4143	-0.0143	11	2	9	2	12	0	12	10	2	8	2	11	0	11
5567.4000	5567.4243	-0.0243	11	4	7	2	12	0	12	10	4	6	2	11	0	11
5567.4000	5567.4251	-0.0251	11	2	9	1	10	1	11	10	2	8	1	9	1	10
5567.4125	5567.4346	-0.0221	11	2	9	1	12	1	13	10	2	8	1	11	1	12
5567.4125	5567.4306	-0.0181	11	2	9	1	12	1	12	10	2	8	1	11	1	11
5567.4125	5567.4308	-0.0183	11	2	9	1	12	0	12	10	2	8	1	11	0	11
5567.4125	5567.4324	-0.0199	11	2	9	1	12	2	13	10	2	8	1	11	2	12
5567.4125	5567.4296	-0.0171	11	2	9	1	12	2	14	10	2	8	1	11	2	13
5567.4125	5567.4361	-0.0236	11	2	9	1	12	2	11	10	2	8	1	11	2	10
5567.4125	5567.4376	-0.0251	11	2	9	1	12	2	12	10	2	8	1	11	2	11
5567.4125	5567.4332	-0.0207	11	2	9	1	12	1	11	10	2	8	1	11	1	10
5567.4626	5567.4572	0.0054	11	2	9	2	11	1	11	10	2	8	2	10	1	10
5567.4626	5567.4546	0.0080	11	4	7	2	10	2	10	10	2	8	2	9	0	9
5567.4626	5567.4629	-0.0003	11	2	9	2	11	1	12	10	2	8	2	10	1	11
5567.4626	5567.4604	0.0022	11	4	7	2	11	2	12	10	6	4	2	10	2	11
5567.4626	5567.4492	0.0134	11	2	9	2	10	1	11	10	2	8	2	9	1	10
5567.4626	5567.4643	-0.0017	11	4	7	0	11	2	10	10	0	10	2	10	2	9

5567.4626	5567.4562	0.0064	11	2	9	2	11	2	13	10	2	8	2	10	2	12
5567.4626	5567.4673	-0.0047	11	2	9	2	11	2	11	10	0	10	2	10	2	10
5567.4626	5567.4575	0.0051	11	0	11	2	11	0	11	10	2	8	2	10	0	10
5567.4626	5567.4615	0.0011	11	2	9	2	11	1	10	10	2	8	2	10	1	9
5791.0375	5791.0378	-0.0003	12	1	12	0	12	1	12	11	1	11	2	11	1	11
5791.0375	5791.0337	0.0038	12	1	12	2	12	2	14	11	1	11	2	11	2	13
5791.0375	5791.0437	-0.0062	12	1	12	0	12	2	10	11	1	11	2	11	2	9
5791.0375	5791.0388	-0.0013	12	1	12	2	12	0	12	11	1	11	2	11	0	11
5791.0625	5791.0793	-0.0168	12	3	10	2	11	2	12	11	3	9	2	10	2	11
5791.0625	5791.0572	0.0053	12	1	12	2	12	1	13	11	1	11	2	11	1	12
5791.0625	5791.0785	-0.0160	12	1	12	2	13	1	13	11	1	11	2	12	1	12
5791.0625	5791.0514	0.0111	12	1	12	2	11	2	13	11	3	9	2	11	2	12
5791.1000	5791.0920	0.0080	12	1	12	1	13	1	14	11	1	11	1	12	1	13
5791.1000	5791.0866	0.0134	12	1	12	1	13	1	13	11	1	11	1	12	1	12
5791.1000	5791.1031	-0.0031	12	1	12	0	12	2	14	11	5	7	2	11	2	13
5791.1000	5791.0805	0.0195	12	1	12	2	13	2	15	11	1	11	2	12	2	14
5791.1000	5791.0956	0.0044	12	1	12	1	13	2	14	11	1	11	1	12	2	13
5791.1000	5791.0829	0.0171	12	1	12	2	13	0	13	11	1	11	2	12	0	12
5791.1000	5791.0767	0.0233	12	1	12	1	13	2	15	11	1	11	1	12	2	14
5791.1000	5791.0831	0.0169	12	1	12	1	11	2	13	11	1	11	1	10	2	12
5791.1000	5791.0754	0.0246	12	1	12	1	13	0	13	11	1	11	1	12	0	12
5791.1000	5791.0710	0.0290	12	1	12	2	12	2	13	11	1	11	2	11	2	12
5791.1292	5791.1236	0.0056	12	3	10	2	12	2	14	11	3	9	2	11	2	13
5791.1292	5791.1148	0.0144	12	1	12	2	13	1	14	11	1	11	2	12	1	13
5791.1292	5791.1132	0.0160	12	1	12	1	12	2	14	11	1	11	1	11	2	13
5791.1292	5791.1360	-0.0068	12	1	12	2	14	2	15	11	1	11	2	13	2	14
5791.1292	5791.1154	0.0138	12	1	12	1	13	2	13	11	1	11	1	12	2	12
5791.1292	5791.1156	0.0136	12	1	12	2	14	1	14	11	1	11	2	13	1	13
5791.1292	5791.1264	0.0028	12	1	12	2	14	2	13	11	1	11	2	10	2	12
5791.1292	5791.1205	0.0087	12	1	12	2	13	2	14	11	1	11	2	13	0	13
5791.1292	5791.1228	0.0064	12	1	12	2	14	2	16	11	1	11	2	13	2	15
5791.1625	5791.1439	0.0186	12	1	12	2	14	1	15	11	1	11	2	13	1	14
5791.1625	5791.1580	0.0045	12	1	12	2	14	0	14	11	1	11	2	13	2	13
5791.1625	5791.1398	0.0227	12	1	12	0	12	1	13	11	1	11	0	11	1	12
5791.1625	5791.1470	0.0155	12	1	12	2	13	2	13	11	1	11	2	13	2	12
5791.1625	5791.1474	0.0151	12	1	12	1	12	1	13	11	1	11	1	11	1	12
5791.1625	5791.1432	0.0193	12	1	12	2	14	1	13	11	1	11	2	13	1	12
5791.1625	5791.1471	0.0154	12	1	12	0	12	2	11	11	1	11	0	11	2	10
5791.1625	5791.1559	0.0066	12	1	12	1	12	0	12	11	1	11	1	11	0	11
5791.1625	5791.1551	0.0074	12	1	12	0	12	2	12	11	1	11	0	11	2	11
5791.1625	5791.1432	0.0193	12	1	12	2	10	1	11	11	1	11	2	9	1	10
5800.4343	5800.4474	-0.0131	12	0	12	2	12	2	10	11	0	11	2	11	2	9
5800.4343	5800.4433	-0.0090	12	0	12	2	12	0	12	11	0	11	2	11	0	11
5800.4343	5800.4390	-0.0047	12	0	12	0	12	2	14	11	0	11	2	11	2	13
5800.4343	5800.4424	-0.0081	12	0	12	0	12	1	12	11	0	11	2	11	1	11
5800.5125	5800.5397	-0.0272	12	2	10	2	11	2	12	11	2	9	2	10	2	11
5800.5125	5800.5227	-0.0102	12	0	12	1	11	1	11	11	0	11	1	10	1	10
5800.5125	5800.5093	0.0032	12	0	12	2	13	1	13	11	0	11	2	12	1	12
5800.5125	5800.5002	0.0123	12	0	12	2	12	2	13	11	0	11	2	11	2	12
5800.5125	5800.5151	-0.0026	12	0	12	2	13	0	13	11	0	11	2	12	0	12
5800.5125	5800.5174	-0.0049	12	0	12	2	13	2	15	11	0	11	2	12	2	14
5800.5125	5800.5225	-0.0100	12	0	12	1	13	1	13	11	0	11	1	12	1	12
5800.5125	5800.5190	-0.0065	12	0	12	1	13	2	15	11	0	11	1	12	2	14

5800.5125	5800.5192	-0.0067	12	0	12	1	11	2	13	11	0	11	1	10	2	12
5800.5125	5800.5229	-0.0104	12	0	12	1	13	0	13	11	0	11	1	12	0	12
5800.5750	5800.5878	-0.0128	12	0	12	2	12	2	14	11	0	11	0	11	2	13
5800.5750	5800.5851	-0.0101	12	0	12	1	12	2	14	11	0	11	1	11	2	13
5800.5750	5800.5855	-0.0105	12	0	12	2	14	0	14	11	0	11	2	13	0	13
5800.5750	5800.5968	-0.0218	12	0	12	1	12	2	13	11	0	11	1	11	2	12
5800.5750	5800.5733	0.0017	12	0	12	2	13	1	12	11	0	11	0	11	1	11
5800.5750	5800.5925	-0.0175	12	0	12	1	13	2	13	11	0	11	1	12	2	12
5800.5750	5800.5969	-0.0219	12	0	12	2	14	2	16	11	0	11	2	13	2	15
5800.5750	5800.5656	0.0094	12	0	12	2	13	2	14	11	0	11	2	12	2	13
5800.5750	5800.5727	0.0023	12	0	12	2	13	1	14	11	0	11	2	12	1	13
5800.5750	5800.5751	-0.0001	12	0	12	2	14	1	14	11	0	11	2	13	1	13
5800.6451	5800.6483	-0.0032	12	0	12	2	14	2	14	11	0	11	2	13	2	13
5800.6451	5800.6499	-0.0048	12	0	12	1	12	0	12	11	0	11	1	11	2	11
5800.6451	5800.6509	-0.0058	12	0	12	2	10	0	10	11	0	11	2	9	2	9
5800.6451	5800.6360	0.0091	12	0	12	2	10	2	9	11	0	11	2	9	2	8
5800.6451	5800.6497	-0.0046	12	0	12	0	12	2	12	11	0	11	0	11	2	11
5917.3750	5917.3683	0.0067	12	2	11	2	12	0	12	11	4	8	0	11	0	11
5917.3750	5917.3720	0.0030	12	2	11	2	12	2	10	11	2	10	2	11	2	9
5917.3750	5917.3652	0.0098	12	2	11	2	12	2	14	11	2	10	2	11	2	13
5917.3750	5917.3678	0.0072	12	2	11	2	12	1	12	11	2	10	2	11	1	11
5917.4000	5917.3898	0.0102	12	2	11	2	12	1	11	11	2	10	2	11	1	10
5917.4000	5917.3923	0.0077	12	2	11	2	12	2	13	11	2	10	2	11	2	12
5917.4000	5917.3997	0.0003	12	4	9	0	12	2	11	11	4	8	0	11	2	10
5917.4000	5917.3917	0.0083	12	2	11	2	12	1	13	11	2	10	2	11	1	12
5917.4375	5917.4189	0.0186	12	2	11	1	11	2	13	11	2	10	1	10	2	12
5917.4375	5917.4231	0.0144	12	2	11	1	13	2	15	11	2	10	1	12	2	14
5917.4375	5917.4153	0.0222	12	2	11	2	13	2	15	11	2	10	2	12	2	14
5917.4375	5917.4397	-0.0022	12	2	11	2	13	2	14	11	2	10	2	12	2	13
5917.4375	5917.4162	0.0213	12	2	11	2	13	1	13	11	2	10	2	12	1	12
5917.4375	5917.4168	0.0207	12	2	11	2	13	0	13	11	2	10	2	12	0	12
5917.4375	5917.4254	0.0121	12	2	11	1	13	1	13	11	2	10	1	12	1	12
5917.4375	5917.4414	-0.0039	12	2	11	2	13	1	14	11	2	10	2	12	1	13
5917.4375	5917.4292	0.0083	12	2	11	2	11	2	13	11	2	10	2	10	2	12
5917.4375	5917.4259	0.0116	12	2	11	1	13	0	13	11	2	10	1	12	0	12
5917.4625	5917.4441	0.0184	12	2	11	1	11	2	12	11	2	10	1	10	2	11
5917.4625	5917.4510	0.0115	12	2	11	1	13	1	14	11	2	10	1	12	1	13
5917.4625	5917.4562	0.0063	12	2	11	2	11	1	12	11	2	10	2	10	1	11
5917.4625	5917.4457	0.0168	12	2	11	1	11	1	12	11	2	10	1	10	1	11
5917.4625	5917.4492	0.0133	12	2	11	1	13	1	12	11	2	10	1	12	1	11
5917.4625	5917.4483	0.0142	12	2	11	1	13	2	14	11	2	10	1	12	2	13
5917.4625	5917.4560	0.0065	12	2	11	1	13	2	12	11	2	10	1	12	2	11
5917.4875	5917.4767	0.0108	12	2	11	1	13	2	13	11	2	10	1	12	2	12
5917.4875	5917.4749	0.0126	12	2	11	2	14	0	14	11	2	10	2	13	0	13
5917.4875	5917.4976	-0.0101	12	2	11	2	14	1	13	11	2	10	2	13	1	12
5917.4875	5917.4734	0.0141	12	2	11	2	14	2	16	11	2	10	2	13	2	15
5917.4875	5917.4779	0.0096	12	2	11	1	12	2	14	11	2	10	1	11	2	13
5917.4875	5917.4782	0.0093	12	2	11	0	12	2	14	11	2	10	0	11	2	13
5917.4875	5917.4744	0.0131	12	2	11	2	14	1	14	11	2	10	2	13	1	13
5917.4875	5917.4830	0.0045	12	2	11	2	11	2	12	11	2	10	2	10	2	11
5917.4875	5917.4956	-0.0081	12	2	11	2	14	2	15	11	2	10	2	13	2	14
5917.4875	5917.4659	0.0216	12	4	9	2	13	0	13	11	4	8	2	12	0	12
5917.5125	5917.5236	-0.0111	12	2	11	2	14	2	14	11	2	10	2	13	2	13

5917.5125	5917.5110	0.0015	12	2	11	2	10	1	11	11	2	10	2	9	1	10
5917.5125	5917.5016	0.0109	12	2	11	2	14	2	13	11	2	10	2	13	2	12
5917.5125	5917.5057	0.0068	12	2	11	1	12	1	13	11	2	10	1	11	1	12
5917.5125	5917.5061	0.0064	12	2	11	0	12	1	13	11	2	10	0	11	1	12
5917.5125	5917.5004	0.0121	12	2	11	1	12	2	13	11	2	10	1	11	2	12
5917.5125	5917.5008	0.0117	12	2	11	0	12	2	13	11	2	10	0	11	2	12
5917.5125	5917.5013	0.0112	12	2	11	2	14	1	15	11	2	10	2	13	1	14
5917.5375	5917.5285	0.0090	12	2	11	1	12	2	12	11	2	10	1	11	2	11
5917.5375	5917.5290	0.0085	12	2	11	0	12	2	12	11	2	10	0	11	2	11
5917.5375	5917.5119	0.0256	12	2	11	2	10	2	9	11	2	10	2	9	2	8
5917.5375	5917.5344	0.0031	12	2	11	2	10	2	10	11	2	10	2	9	2	9
5969.1988	5969.2063	-0.0075	12	8	5	2	14	2	16	11	8	4	2	13	2	15
5969.1988	5969.1966	0.0022	12	8	4	1	12	2	14	11	8	3	1	11	2	13
5969.1988	5969.1959	0.0029	12	8	4	0	12	2	14	11	8	3	0	11	2	13
5969.1988	5969.1955	0.0033	12	8	5	2	14	0	14	11	8	4	2	13	0	13
5969.1988	5969.1959	0.0029	12	8	5	0	12	2	14	11	8	4	0	11	2	13
5969.1988	5969.1968	0.0020	12	8	4	2	14	1	14	11	8	3	2	13	1	13
5969.1988	5969.1968	0.0020	12	8	5	2	14	1	14	11	8	4	2	13	1	13
5969.1988	5969.2063	-0.0075	12	8	4	2	14	2	16	11	8	3	2	13	2	15
5969.1988	5969.1966	0.0022	12	8	5	1	12	2	14	11	8	4	1	11	2	13
5969.1988	5969.1955	0.0033	12	8	4	2	14	0	14	11	8	3	2	13	0	13
5969.3238	5969.3193	0.0045	12	8	5	1	13	2	15	11	8	4	1	12	2	14
5969.3238	5969.3439	-0.0201	12	8	4	2	14	2	15	11	8	3	2	13	2	14
5969.3238	5969.3041	0.0197	12	8	5	1	12	1	13	11	8	4	1	11	1	12
5969.3238	5969.3112	0.0126	12	8	5	2	14	1	15	11	8	4	2	13	1	14
5969.3238	5969.3289	-0.0051	12	8	5	2	13	2	15	11	8	4	2	12	2	14
5969.3238	5969.3289	-0.0051	12	8	4	2	13	2	15	11	8	3	2	12	2	14
5969.3238	5969.3112	0.0126	12	8	4	2	14	1	15	11	8	3	2	13	1	14
5969.3238	5969.3193	0.0045	12	8	4	1	13	2	15	11	8	3	1	12	2	14
5969.3238	5969.3041	0.0197	12	8	4	1	12	1	13	11	8	3	1	11	1	12
5969.3238	5969.3439	-0.0201	12	8	5	2	14	2	15	11	8	4	2	13	2	14
5969.4419	5969.4554	-0.0135	12	8	4	2	14	2	14	11	8	3	2	13	2	13
5969.4419	5969.4554	-0.0135	12	8	5	2	14	2	14	11	8	4	2	13	2	13
5969.4419	5969.4437	-0.0018	12	8	4	2	12	2	14	11	8	3	2	11	2	13
5969.4419	5969.4445	-0.0026	12	8	4	2	13	1	14	11	8	3	2	12	1	13
5969.4419	5969.4445	-0.0026	12	8	5	2	13	1	14	11	8	4	2	12	1	13
5969.4419	5969.4241	0.0178	12	8	4	1	13	1	14	11	8	3	1	12	1	13
5969.4419	5969.4241	0.0178	12	8	5	1	13	1	14	11	8	4	1	12	1	13
5969.4419	5969.4437	-0.0018	12	8	5	2	12	2	14	11	8	4	2	11	2	13
5969.4419	5969.4441	-0.0022	12	8	4	1	13	2	14	11	8	3	1	12	2	13
5969.4419	5969.4441	-0.0022	12	8	5	1	13	2	14	11	8	4	1	12	2	13
5970.7525	5970.7474	0.0051	12	7	5	0	12	2	14	11	7	4	0	11	2	13
5970.7525	5970.7473	0.0052	12	7	6	0	12	2	14	11	5	7	0	11	2	13
5970.7525	5970.7478	0.0047	12	7	5	2	14	0	14	11	7	4	2	13	0	13
5970.7525	5970.7484	0.0041	12	7	6	2	14	1	14	11	7	5	2	13	1	13
5970.7525	5970.7484	0.0041	12	7	5	2	14	1	14	11	7	4	2	13	1	13
5970.7525	5970.7557	-0.0032	12	7	5	2	14	2	16	11	7	4	2	13	2	15
5970.7525	5970.7556	-0.0031	12	7	6	2	14	2	16	11	7	5	2	13	2	15
5970.7525	5970.7480	0.0045	12	7	6	1	12	2	14	11	7	5	1	11	2	13
5970.7525	5970.7481	0.0044	12	7	5	1	12	2	14	11	7	4	1	11	2	13
5970.7525	5970.7477	0.0048	12	7	6	2	14	0	14	11	7	5	2	13	0	13
5970.8472	5970.8625	-0.0153	12	7	6	2	13	2	15	11	7	5	2	12	2	14
5970.8472	5970.8399	0.0073	12	7	5	1	13	1	13	11	7	4	1	12	1	12

5970.8472	5970.8398	0.0074	12	7	6	1	13	1	13	11	7	5	1	12	1	12
5970.8472	5970.8470	0.0002	12	7	5	1	13	2	15	11	7	4	1	12	2	14
5970.8472	5970.8502	-0.0030	12	7	5	2	14	2	15	11	7	4	2	13	2	14
5970.8472	5970.8469	0.0003	12	7	6	1	13	2	15	11	7	5	1	12	2	14
5970.8472	5970.8361	0.0111	12	7	5	2	14	1	15	11	7	4	2	13	1	14
5970.8472	5970.8360	0.0112	12	7	6	2	14	1	15	11	7	5	2	13	1	14
5970.8472	5970.8501	-0.0029	12	7	6	2	14	2	15	11	7	5	2	13	2	14
5970.8472	5970.8626	-0.0154	12	7	5	2	13	2	15	11	7	4	2	12	2	14
5971.0554	5971.0364	0.0190	12	9	3	2	13	0	13	11	7	4	2	12	0	12
5971.0554	5971.0363	0.0191	12	9	4	2	13	0	13	11	7	5	2	12	0	12
5971.0554	5971.0481	0.0073	12	9	4	2	12	2	13	11	9	3	2	11	2	12
5971.0554	5971.0481	0.0073	12	7	5	2	12	2	13	11	7	4	2	11	2	12
5971.0554	5971.0297	0.0257	12	7	5	1	11	2	11	11	5	6	1	10	2	10
5971.0554	5971.0216	0.0338	12	7	5	1	13	2	13	11	7	4	1	12	2	12
5971.0554	5971.0331	0.0223	12	7	6	2	12	1	13	11	7	5	2	11	1	12
5971.0554	5971.0332	0.0222	12	7	5	2	12	1	13	11	7	4	2	11	1	12
5971.0554	5971.0215	0.0339	12	7	6	1	13	2	13	11	7	5	1	12	2	12
5971.0554	5971.0297	0.0257	12	7	6	1	11	2	11	11	5	7	1	10	2	10
5973.1482	5973.1449	0.0033	12	6	6	0	12	2	14	11	6	5	0	11	2	13
5973.1482	5973.1446	0.0036	12	6	6	2	14	0	14	11	6	5	2	13	0	13
5973.1482	5973.1392	0.0090	12	6	7	0	12	2	14	11	6	6	0	11	2	13
5973.1482	5973.1387	0.0095	12	6	7	2	14	0	14	11	6	6	2	13	0	13
5973.1482	5973.1431	0.0051	12	6	7	2	14	2	16	11	6	6	2	13	2	15
5973.1482	5973.1451	0.0031	12	6	6	1	12	2	14	11	6	5	1	11	2	13
5973.1482	5973.1409	0.0073	12	6	6	1	12	1	12	11	6	5	1	11	1	11
5973.1482	5973.1448	0.0034	12	6	6	2	14	1	14	11	6	5	2	13	1	13
5973.1482	5973.1390	0.0092	12	6	7	2	14	1	14	11	6	6	2	13	1	13
5973.1482	5973.1392	0.0090	12	6	7	1	12	2	14	11	6	6	1	11	2	13
5973.2425	5973.2132	0.0293	12	6	6	2	14	2	15	11	6	5	2	13	2	14
5973.2425	5973.2180	0.0245	12	6	7	1	13	2	15	11	6	6	1	12	2	14
5973.2425	5973.2075	0.0350	12	6	6	2	14	1	15	11	6	5	2	13	1	14
5973.2425	5973.2018	0.0407	12	6	7	2	14	1	15	11	6	6	2	13	1	14
5973.2425	5973.2143	0.0282	12	6	7	1	13	0	13	11	6	6	1	12	0	12
5973.2425	5973.2202	0.0223	12	6	6	1	13	1	13	11	6	5	1	12	1	12
5973.2425	5973.2106	0.0319	12	6	6	2	14	1	13	11	6	5	2	13	1	12
5973.2425	5973.2048	0.0377	12	6	7	2	14	1	13	11	6	6	2	13	1	12
5973.2425	5973.2074	0.0351	12	6	7	2	14	2	15	11	6	6	2	13	2	14
5973.2425	5973.2144	0.0281	12	6	7	1	13	1	13	11	6	6	1	12	1	12
5973.2875	5973.2911	-0.0036	12	6	6	1	13	2	14	11	6	5	1	12	2	13
5973.2875	5973.2827	0.0048	12	6	6	1	13	1	14	11	6	5	1	12	1	13
5973.2875	5973.2872	0.0003	12	6	6	1	13	1	12	11	6	5	1	12	1	11
5973.2875	5973.2828	0.0047	12	6	7	1	11	1	12	11	6	6	1	10	1	11
5973.2875	5973.2912	-0.0037	12	6	7	2	13	2	14	11	6	6	2	12	2	13
5973.2875	5973.2815	0.0060	12	6	7	1	13	1	12	11	6	6	1	12	1	11
5973.2875	5973.2769	0.0106	12	6	7	1	13	1	14	11	6	6	1	12	1	13
5973.2875	5973.2853	0.0022	12	6	7	1	13	2	14	11	6	6	1	12	2	13
5973.2875	5973.2904	-0.0029	12	6	6	2	13	1	14	11	6	5	2	12	1	13
5973.2875	5973.2846	0.0029	12	6	7	2	13	1	14	11	6	6	2	12	1	13
5973.3418	5973.3690	-0.0272	12	6	6	2	12	1	11	11	6	5	2	11	1	10
5973.3418	5973.3498	-0.0080	12	6	7	1	11	2	11	11	6	6	1	10	2	10
5973.3418	5973.3562	-0.0144	12	4	9	2	12	2	11	11	4	8	2	11	2	10
5973.3418	5973.3496	-0.0078	12	6	6	1	13	2	13	11	6	5	1	12	2	12
5973.3418	5973.3644	-0.0226	12	6	6	2	12	1	13	11	6	5	2	11	1	12

5973.3418	5973.3555	-0.0137	12	6	6	1	11	2	11	11	6	5	1	10	2	10
5973.3418	5973.3539	-0.0121	12	4	8	2	13	2	13	11	4	7	2	12	2	12
5973.3418	5973.3694	-0.0276	12	6	7	2	12	2	13	11	6	6	2	11	2	12
5973.3418	5973.3585	-0.0167	12	6	7	2	12	1	13	11	6	6	2	11	1	12
5973.3418	5973.3631	-0.0213	12	6	7	2	12	1	11	11	6	6	2	11	1	10
5976.0732	5976.0761	-0.0029	12	3	10	2	14	2	16	11	3	9	2	13	2	15
5976.0732	5976.0858	-0.0126	12	3	10	1	13	2	15	11	3	9	1	12	2	14
5976.0732	5976.0768	-0.0036	12	3	10	2	14	0	14	11	3	9	2	13	0	13
5976.0732	5976.0792	-0.0060	12	3	10	0	12	2	14	11	1	11	0	11	2	13
5976.0732	5976.0903	-0.0171	12	5	8	2	13	2	14	11	5	7	2	11	2	13
5976.0732	5976.0786	-0.0054	12	3	10	1	12	2	14	11	3	9	1	11	2	13
5976.0732	5976.0845	-0.0113	12	3	10	1	13	1	13	11	3	9	1	12	1	12
5976.0732	5976.0866	-0.0134	12	3	10	1	13	0	13	11	3	9	1	12	0	12
5976.0732	5976.0766	-0.0034	12	3	10	2	14	1	14	11	3	9	2	13	1	13
5976.0732	5976.0795	-0.0063	12	3	10	2	13	2	15	11	3	9	2	12	2	14
5976.1000	5976.1072	-0.0072	12	3	10	1	13	1	14	11	3	9	1	12	1	13
5976.1000	5976.1058	-0.0058	12	3	10	1	13	2	14	11	3	9	1	12	2	13
5976.1000	5976.0998	0.0002	12	3	10	2	13	2	14	11	3	9	2	12	2	13
5976.1000	5976.0967	0.0033	12	3	10	2	14	1	13	11	3	9	2	13	1	12
5976.1000	5976.1012	-0.0012	12	3	10	0	12	1	13	11	3	9	0	11	1	12
5976.1000	5976.1005	-0.0005	12	3	10	1	12	1	13	11	3	9	1	11	1	12
5976.1000	5976.0975	0.0025	12	3	10	2	14	1	15	11	3	9	2	13	1	14
5976.1000	5976.0961	0.0039	12	3	10	2	14	2	15	11	3	9	2	13	2	14
5976.1000	5976.1016	-0.0016	12	3	10	2	13	1	14	11	3	9	2	12	1	13
5976.1000	5976.1119	-0.0119	12	3	10	2	12	1	13	11	3	9	2	11	1	12
5976.1250	5976.1227	0.0023	12	3	10	2	13	2	13	11	1	11	2	12	2	12
5976.1250	5976.1212	0.0038	12	1	12	0	12	2	12	11	3	9	0	11	2	11
5976.1250	5976.1276	-0.0026	12	3	10	1	13	2	13	11	3	9	1	12	2	12
5976.1250	5976.1333	-0.0083	12	3	10	0	12	2	12	11	1	11	2	11	2	11
5976.1250	5976.1242	0.0008	12	3	10	1	11	2	11	11	5	7	1	10	0	10
5976.1250	5976.1218	0.0032	12	1	12	1	12	2	12	11	1	11	1	11	2	11
5976.1250	5976.1178	0.0072	12	1	12	2	14	2	14	11	3	9	2	13	2	13
5976.1250	5976.1349	-0.0099	12	5	8	2	11	2	11	11	5	7	2	10	2	10
5976.1250	5976.1269	-0.0019	12	3	10	2	11	2	10	11	1	11	2	10	2	9
5977.0830	5977.0799	0.0031	12	5	8	2	14	1	14	11	5	7	2	13	1	13
5977.0830	5977.0799	0.0031	12	5	8	2	14	0	14	11	5	7	2	13	0	13
5977.0830	5977.0770	0.0060	12	7	6	0	12	0	12	11	5	7	0	11	0	11
5977.0830	5977.0779	0.0051	12	5	8	2	14	2	12	11	5	7	2	13	2	11
5977.0830	5977.0818	0.0012	12	5	8	2	14	2	16	11	5	7	2	13	2	15
5977.0830	5977.0791	0.0039	12	5	8	1	12	2	14	11	5	7	1	11	2	13
5977.0830	5977.0790	0.0040	12	5	8	0	12	2	14	11	3	9	0	11	2	13
5977.0830	5977.0771	0.0059	12	5	8	1	12	1	12	11	5	7	1	11	1	11
5977.0830	5977.0770	0.0060	12	5	8	0	12	1	12	11	3	9	0	11	1	11
5977.0830	5977.0770	0.0060	12	5	8	1	12	0	12	11	5	7	1	11	0	11
5977.1045	5977.1207	-0.0162	12	5	8	0	12	1	11	11	5	7	0	11	1	10
5977.1045	5977.1223	-0.0178	12	5	8	1	12	2	13	11	5	7	1	11	2	12
5977.1045	5977.1218	-0.0173	12	5	8	2	14	1	15	11	5	7	2	13	1	14
5977.1045	5977.1207	-0.0162	12	5	8	1	12	1	11	11	5	7	1	11	1	10
5977.1045	5977.1234	-0.0189	12	5	8	2	14	1	13	11	5	7	2	13	1	12
5977.1045	5977.1187	-0.0142	12	5	8	0	12	1	13	11	5	7	0	11	1	12
5977.1045	5977.1187	-0.0142	12	5	8	1	12	1	13	11	5	7	1	11	1	12
5977.1045	5977.1194	-0.0149	12	5	8	2	14	2	13	11	5	7	2	13	2	12
5977.1045	5977.1222	-0.0177	12	5	8	0	12	2	13	11	5	7	0	11	2	12

5977.1045	5977.1249	-0.0204	12	5	8	2	14	2	15	11	5	7	2	13	2	14
5977.1625	5977.1419	0.0206	12	5	8	1	13	1	13	11	5	7	1	12	1	12
5977.1625	5977.1467	0.0158	12	5	8	1	11	2	13	11	5	7	1	10	2	12
5977.1625	5977.1478	0.0147	12	5	8	2	13	0	13	11	5	7	2	12	0	12
5977.1625	5977.1436	0.0189	12	5	8	1	13	2	15	11	5	7	1	12	2	14
5977.1625	5977.1478	0.0147	12	5	8	2	13	1	13	11	5	7	2	12	1	12
5977.1625	5977.1413	0.0212	12	3	10	2	11	2	13	11	5	7	2	10	2	12
5977.1625	5977.1415	0.0210	12	5	8	1	13	2	11	11	5	7	1	12	2	10
5977.1625	5977.1504	0.0121	12	5	8	1	11	0	11	11	7	5	1	10	0	10
5977.1625	5977.1500	0.0125	12	5	8	2	13	2	15	11	5	7	2	12	2	14
5977.1625	5977.1418	0.0207	12	5	8	1	13	0	13	11	5	7	1	12	0	12
5977.2000	5977.1927	0.0073	12	7	6	2	13	2	14	11	5	7	2	12	2	13
5977.2000	5977.1939	0.0061	12	5	8	2	13	1	14	11	5	7	2	12	1	13
5977.2000	5977.1835	0.0165	12	5	8	1	13	1	14	11	5	7	1	12	1	13
5977.2000	5977.1879	0.0121	12	5	8	1	13	1	12	11	5	7	1	12	1	11
5977.2000	5977.1862	0.0138	12	5	8	1	11	1	12	11	5	7	1	10	1	11
5977.2000	5977.1906	0.0094	12	7	6	1	11	2	12	11	7	5	1	10	2	11
5977.2000	5977.1818	0.0182	12	5	8	1	13	2	12	11	5	7	1	12	2	11
5977.2000	5977.1874	0.0126	12	5	8	1	13	2	14	11	5	7	1	12	2	13
5977.2000	5977.1803	0.0197	12	7	6	2	11	1	12	11	7	5	2	10	1	11
5977.2000	5977.1907	0.0093	12	5	8	2	13	1	12	11	5	7	2	12	1	11
5977.3000	5977.3113	-0.0113	12	5	7	2	14	0	14	11	5	6	2	13	0	13
5977.3000	5977.3083	-0.0083	12	5	7	0	12	0	12	11	5	6	0	11	0	11
5977.3000	5977.3114	-0.0114	12	5	7	2	14	1	14	11	5	6	2	13	1	13
5977.3000	5977.3084	-0.0084	12	5	7	1	12	0	12	11	5	6	1	11	0	11
5977.3000	5977.3084	-0.0084	12	5	7	1	12	1	12	11	5	6	1	11	1	11
5977.3000	5977.3133	-0.0133	12	5	7	2	14	2	16	11	5	6	2	13	2	15
5977.3000	5977.3093	-0.0093	12	5	7	2	14	2	12	11	5	6	2	13	2	11
5977.3000	5977.3104	-0.0104	12	5	7	1	12	2	14	11	5	6	1	11	2	13
5977.3000	5977.3084	-0.0084	12	5	7	0	12	1	12	11	5	6	0	11	1	11
5977.3000	5977.3104	-0.0104	12	5	7	0	12	2	14	11	5	6	0	11	2	13
5977.3375	5977.3464	-0.0089	12	5	7	1	12	1	11	11	5	6	1	11	1	10
5977.3375	5977.3458	-0.0083	12	5	7	0	12	1	11	11	5	6	0	11	1	10
5977.3375	5977.3533	-0.0158	12	5	7	0	12	2	13	11	5	6	0	11	2	12
5977.3375	5977.3497	-0.0122	12	5	7	0	12	1	13	11	5	6	0	11	1	12
5977.3375	5977.3533	-0.0158	12	5	7	1	12	2	13	11	5	6	1	11	2	12
5977.3375	5977.3505	-0.0130	12	5	7	2	14	2	13	11	5	6	2	13	2	12
5977.3375	5977.3529	-0.0154	12	5	7	2	14	1	15	11	5	6	2	13	1	14
5977.3375	5977.3560	-0.0185	12	5	7	2	14	2	15	11	5	6	2	13	2	14
5977.3375	5977.3545	-0.0170	12	5	7	2	14	1	13	11	5	6	2	13	1	12
5977.3375	5977.3498	-0.0123	12	5	7	1	12	1	13	11	5	6	1	11	1	12
5977.4000	5977.3763	0.0237	12	5	7	1	13	2	15	11	5	6	1	12	2	14
5977.4000	5977.3738	0.0262	12	5	7	2	11	2	13	11	5	6	2	10	2	12
5977.4000	5977.3828	0.0172	12	5	7	2	13	2	15	11	5	6	2	12	2	14
5977.4000	5977.3745	0.0255	12	5	7	1	13	0	13	11	5	6	1	12	0	12
5977.4000	5977.3745	0.0255	12	5	7	1	13	1	13	11	5	6	1	12	1	12
5977.4000	5977.3727	0.0273	12	5	7	1	13	2	11	11	5	6	1	12	2	10
5977.4000	5977.3807	0.0193	12	5	7	2	13	1	13	11	5	6	2	12	1	12
5977.4000	5977.3727	0.0273	12	5	7	1	11	0	11	11	7	4	1	10	0	10
5977.4000	5977.3795	0.0205	12	5	7	1	11	2	13	11	5	6	1	10	2	12
5977.4000	5977.3806	0.0194	12	5	7	2	13	0	13	11	5	6	2	12	0	12
5977.4375	5977.4187	0.0188	12	5	7	1	11	1	12	11	5	6	1	10	1	11
5977.4375	5977.4141	0.0234	12	5	7	1	13	2	12	11	5	6	1	12	2	11

5977.4375	5977.4159	0.0216	12	5	7	1	13	1	14	11	5	6	1	12	1	13
5977.4375	5977.4197	0.0178	12	5	7	1	13	2	14	11	5	6	1	12	2	13
5977.4375	5977.4216	0.0159	12	5	7	2	13	1	14	11	5	6	2	12	1	13
5977.4375	5977.4253	0.0122	12	5	7	2	13	2	14	11	5	6	2	12	2	13
5977.4375	5977.4122	0.0253	12	5	7	1	13	1	12	11	5	6	1	12	1	11
5977.4375	5977.4232	0.0143	12	5	7	2	13	1	12	11	5	6	2	12	1	11
5977.4375	5977.4126	0.0249	12	5	7	2	11	1	12	11	5	6	2	10	1	11
5977.4375	5977.4229	0.0146	12	5	7	1	11	2	12	11	5	6	1	10	2	11
5977.5253	5977.4866	0.0387	12	5	7	2	12	1	11	11	5	6	2	11	1	10
5977.5253	5977.4846	0.0407	12	5	7	2	12	1	13	11	5	6	2	11	1	12
5977.5253	5977.4893	0.0360	12	5	7	2	12	2	13	11	5	6	2	11	2	12
5977.5253	5977.4832	0.0421	12	5	7	2	12	2	11	11	5	6	2	11	2	10
5982.4621	5982.4900	-0.0279	12	4	9	2	14	2	12	11	4	8	2	13	2	11
5982.4621	5982.4889	-0.0268	12	4	9	0	12	0	12	11	6	6	0	11	0	11
5982.4621	5982.4890	-0.0269	12	4	9	0	12	2	14	11	4	8	0	11	2	13
5982.4621	5982.4889	-0.0268	12	4	9	1	12	2	14	11	4	8	1	11	2	13
5982.4621	5982.4904	-0.0283	12	4	9	2	14	2	16	11	4	8	2	13	2	15
5982.4621	5982.4901	-0.0280	12	4	9	2	14	1	14	11	4	8	2	13	1	13
5982.4621	5982.4889	-0.0268	12	4	9	1	12	0	12	11	4	8	1	11	0	11
5982.4621	5982.4902	-0.0281	12	4	9	2	14	0	14	11	4	8	2	13	0	13
5982.4621	5982.4887	-0.0266	12	4	9	1	12	1	12	11	4	8	1	11	1	11
5982.4621	5982.4888	-0.0267	12	4	9	0	12	1	12	11	4	8	0	11	1	11
5982.4875	5982.5157	-0.0282	12	4	9	1	12	2	13	11	4	8	1	11	2	12
5982.4875	5982.5157	-0.0282	12	4	9	2	14	2	13	11	4	8	2	13	2	12
5982.4875	5982.5145	-0.0270	12	4	9	1	12	1	11	11	4	8	1	11	1	10
5982.4875	5982.5146	-0.0271	12	4	9	0	12	1	11	11	4	8	0	11	1	10
5982.4875	5982.5166	-0.0291	12	4	9	2	14	2	15	11	4	8	2	13	2	14
5982.4875	5982.5159	-0.0284	12	4	9	2	14	1	13	11	4	8	2	13	1	12
5982.4875	5982.5145	-0.0270	12	4	9	0	12	1	13	11	4	8	0	11	1	12
5982.4875	5982.5153	-0.0278	12	4	9	2	14	1	15	11	4	8	2	13	1	14
5982.4875	5982.5145	-0.0270	12	4	9	1	12	1	13	11	4	8	1	11	1	12
5982.4875	5982.5158	-0.0283	12	4	9	0	12	2	13	11	4	8	0	11	2	12
5982.5000	5982.5393	-0.0393	12	6	7	2	13	0	13	11	6	6	2	12	0	12
5982.5000	5982.5392	-0.0392	12	4	9	2	13	1	13	11	4	8	2	12	1	12
5982.5000	5982.5390	-0.0390	12	4	9	1	11	2	13	11	4	8	1	10	2	12
5982.5000	5982.5396	-0.0396	12	4	9	1	13	1	13	11	4	8	1	12	1	12
5982.5000	5982.5396	-0.0396	12	4	9	1	13	0	13	11	4	8	1	12	0	12
5982.5000	5982.5414	-0.0414	12	4	9	0	12	2	12	11	4	8	0	11	2	11
5982.5000	5982.5413	-0.0413	12	4	9	2	14	2	14	11	4	8	2	13	2	13
5982.5000	5982.5402	-0.0402	12	4	9	1	13	2	15	11	4	8	1	12	2	14
5982.5000	5982.5367	-0.0367	12	4	9	2	11	2	13	11	4	8	2	10	2	12
5982.5000	5982.5394	-0.0394	12	4	9	2	13	2	15	11	4	8	2	12	2	14
5982.5500	5982.5639	-0.0139	12	4	9	1	13	2	12	11	4	8	1	12	2	11
5982.5500	5982.5645	-0.0145	12	4	9	1	11	2	12	11	4	8	1	10	2	11
5982.5500	5982.5641	-0.0141	12	4	9	2	11	1	12	11	4	8	2	10	1	11
5982.5500	5982.5651	-0.0151	12	4	9	1	13	1	12	11	4	8	1	12	1	11
5982.5500	5982.5649	-0.0149	12	4	9	1	11	1	12	11	4	8	1	10	1	11
5982.5500	5982.5667	-0.0167	12	4	9	2	13	1	12	11	4	8	2	12	1	11
5982.5500	5982.5653	-0.0153	12	4	9	1	13	2	14	11	4	8	1	12	2	13
5982.5500	5982.5656	-0.0156	12	4	9	2	13	1	14	11	4	8	2	12	1	13
5982.5500	5982.5674	-0.0174	12	4	9	2	13	2	14	11	4	8	2	12	2	13
5982.5500	5982.5651	-0.0151	12	4	9	1	13	1	14	11	4	8	1	12	1	13
5982.5875	5982.5887	-0.0012	12	4	9	2	12	2	14	11	4	8	2	11	2	13

5982.5875	5982.5905	-0.0030	12	4	9	1	13	2	13	11	4	8	1	12	2	12
5982.5875	5982.5893	-0.0018	12	4	9	2	12	2	10	11	4	8	2	11	2	9
5982.5875	5982.5918	-0.0043	12	4	9	2	11	2	11	11	4	8	2	10	2	10
5982.5875	5982.5890	-0.0015	12	4	9	2	12	0	12	11	2	10	2	11	0	11
5982.5875	5982.5888	-0.0013	12	4	9	2	12	1	12	11	4	8	2	11	1	11
5982.5875	5982.5922	-0.0047	12	2	11	2	13	2	13	11	2	10	2	12	2	12
5982.5875	5982.5903	-0.0028	12	4	9	1	11	2	11	11	4	8	1	10	2	10
5987.7500	5987.7696	-0.0196	12	4	8	1	12	2	14	11	4	7	1	11	2	13
5987.7500	5987.7727	-0.0227	12	4	8	2	14	1	14	11	4	7	2	13	1	13
5987.7500	5987.7700	-0.0200	12	4	8	1	12	0	12	11	4	7	1	11	0	11
5987.7500	5987.7728	-0.0228	12	4	8	2	14	0	14	11	4	7	2	13	0	13
5987.7500	5987.7726	-0.0226	12	4	8	2	14	2	16	11	4	7	2	13	2	15
5987.7500	5987.7698	-0.0198	12	4	8	1	12	1	12	11	4	7	1	11	1	11
5987.7500	5987.7696	-0.0196	12	4	8	0	12	2	14	11	4	7	0	11	2	13
5987.7500	5987.7699	-0.0199	12	4	8	0	12	0	12	11	6	5	0	11	0	11
5987.7500	5987.7697	-0.0197	12	4	8	0	12	1	12	11	4	7	0	11	1	11
5987.7500	5987.7731	-0.0231	12	4	8	2	14	2	12	11	4	7	2	13	2	11
5987.8000	5987.7896	0.0104	12	4	8	1	12	1	11	11	4	7	1	11	1	10
5987.8000	5987.7896	0.0104	12	4	8	0	12	1	11	11	4	7	0	11	1	10
5987.8000	5987.7898	0.0102	12	4	8	0	12	1	13	11	4	7	0	11	1	12
5987.8000	5987.7907	0.0093	12	4	8	1	12	2	13	11	4	7	1	11	2	12
5987.8000	5987.7921	0.0079	12	4	8	2	14	1	15	11	4	7	2	13	1	14
5987.8000	5987.7926	0.0074	12	4	8	2	14	1	13	11	4	7	2	13	1	12
5987.8000	5987.7908	0.0092	12	4	8	0	12	2	13	11	4	7	0	11	2	12
5987.8000	5987.7899	0.0101	12	4	8	1	12	1	13	11	4	7	1	11	1	12
5987.8000	5987.7932	0.0068	12	4	8	2	14	2	13	11	4	7	2	13	2	12
5987.8000	5987.7933	0.0067	12	4	8	2	14	2	15	11	4	7	2	13	2	14
5987.8250	5987.8431	-0.0181	12	4	8	1	13	2	15	11	4	7	1	12	2	14
5987.8250	5987.8445	-0.0195	12	4	8	2	13	2	15	11	4	7	2	12	2	14
5987.8250	5987.8428	-0.0178	12	4	8	1	13	1	13	11	4	7	1	12	1	12
5987.8250	5987.8450	-0.0200	12	6	6	2	13	0	13	11	6	5	2	12	0	12
5987.8250	5987.8448	-0.0198	12	4	8	2	13	1	13	11	4	7	2	12	1	12
5987.8250	5987.8427	-0.0177	12	4	8	1	13	0	13	11	4	7	1	12	0	12
5987.8250	5987.8430	-0.0180	12	4	8	1	11	2	13	11	4	7	1	10	2	12
5987.8250	5987.8444	-0.0194	12	4	8	1	11	0	11	11	4	7	1	10	0	10
5987.8250	5987.8371	-0.0121	12	2	10	2	11	2	13	11	2	9	2	10	2	12
5987.8250	5987.8428	-0.0178	12	4	8	1	13	2	11	11	4	7	1	12	2	10
5987.8875	5987.8673	0.0202	12	4	8	2	13	2	14	11	4	7	2	12	2	13
5987.8875	5987.8659	0.0216	12	4	8	2	13	1	14	11	4	7	2	12	1	13
5987.8875	5987.8626	0.0249	12	4	8	1	13	1	14	11	4	7	1	12	1	13
5987.8875	5987.8621	0.0254	12	4	8	1	13	2	14	11	4	7	1	12	2	13
5987.8875	5987.8640	0.0235	12	4	8	1	11	1	12	11	4	7	1	10	1	11
5987.8875	5987.8614	0.0261	12	4	8	1	13	2	12	11	4	7	1	12	2	11
5987.8875	5987.8620	0.0255	12	6	6	1	11	2	12	11	4	7	1	10	2	11
5987.8875	5987.8598	0.0277	12	6	6	2	11	1	12	11	4	7	2	10	1	11
5987.8875	5987.8675	0.0200	12	4	8	2	13	1	12	11	4	7	2	12	1	11
5987.8875	5987.8622	0.0253	12	4	8	1	13	1	12	11	4	7	1	12	1	11
5987.9486	5987.9151	0.0335	12	4	8	2	12	0	12	11	2	9	2	11	0	11
5987.9486	5987.9149	0.0337	12	2	10	2	12	1	12	11	4	7	2	11	1	11
5987.9486	5987.9142	0.0344	12	4	8	2	12	2	14	11	4	7	2	11	2	13
5987.9486	5987.9160	0.0326	12	4	8	2	12	2	10	11	4	7	2	11	2	9
5987.9625	5987.9364	0.0261	12	4	8	2	12	1	11	11	4	7	2	11	1	10
5987.9625	5987.9350	0.0275	12	0	12	2	12	2	11	11	2	9	2	11	2	10

5987.9625	5987.9369	0.0256	12	4	8	2	12	1	13	11	4	7	2	11	1	12
5987.9625	5987.9349	0.0276	12	6	6	2	12	2	13	11	6	5	2	11	2	12
6029.4233	6029.4259	-0.0026	12	3	9	0	12	2	14	11	3	8	0	11	2	13
6029.4233	6029.4322	-0.0089	12	3	9	2	14	1	14	11	3	8	2	13	1	13
6029.4233	6029.4256	-0.0023	12	3	9	1	12	2	14	11	3	8	1	11	2	13
6029.4233	6029.4091	0.0142	12	3	9	2	14	2	14	11	3	8	2	13	2	13
6029.4233	6029.4193	0.0040	12	3	9	2	14	1	13	11	3	8	2	13	1	12
6029.4233	6029.4325	-0.0092	12	3	9	2	14	0	14	11	3	8	2	13	0	13
6029.4233	6029.4180	0.0053	12	3	9	2	14	2	15	11	3	8	2	13	2	14
6029.4233	6029.4300	-0.0067	12	3	9	2	14	2	16	11	3	8	2	13	2	15
6029.4233	6029.4178	0.0055	12	3	9	1	12	1	13	11	3	8	1	11	1	12
6029.4233	6029.4211	0.0022	12	3	9	2	14	1	15	11	3	8	2	13	1	14
6029.5558	6029.5556	0.0002	12	3	9	1	13	0	13	11	3	8	1	12	0	12
6029.5558	6029.5553	0.0005	12	3	9	1	13	1	13	11	3	8	1	12	1	12
6029.5558	6029.5586	-0.0028	12	3	9	2	13	2	15	11	3	8	2	12	2	14
6029.5558	6029.5552	0.0006	12	3	9	1	11	2	13	11	3	8	1	10	2	12
6029.5558	6029.5533	0.0025	12	3	9	1	13	2	15	11	3	8	1	12	2	14
6029.5558	6029.5311	0.0247	12	3	9	1	13	2	13	11	3	8	1	12	2	12
6029.5558	6029.5445	0.0113	12	3	9	1	13	1	14	11	3	8	1	12	1	13
6029.5558	6029.5395	0.0163	12	3	9	1	13	2	14	11	3	8	1	12	2	13
6029.5558	6029.5524	0.0034	12	3	9	2	13	1	14	11	3	8	2	12	1	13
6029.5558	6029.5486	0.0072	12	3	9	2	13	2	14	11	3	8	2	12	2	13
6029.6886	6029.6639	0.0247	12	3	9	2	12	2	12	11	3	8	2	11	2	11
6029.6886	6029.6732	0.0154	12	3	9	2	12	1	11	11	3	8	2	11	1	10
6029.6886	6029.6876	0.0010	12	3	9	2	12	2	10	11	3	8	2	11	2	9
6029.6886	6029.6846	0.0040	12	3	9	2	12	0	12	11	3	8	2	11	0	11
6029.6886	6029.6842	0.0044	12	3	9	2	12	1	12	11	3	8	2	11	1	11
6029.6886	6029.6697	0.0189	12	3	9	2	12	2	13	11	3	8	2	11	2	12
6029.6886	6029.6775	0.0111	12	3	9	2	12	2	11	11	3	8	2	11	2	10
6029.6886	6029.6761	0.0125	12	3	9	2	12	1	13	11	3	8	2	11	1	12
6029.6886	6029.6816	0.0070	12	3	9	2	12	2	14	11	3	8	2	11	2	13
6074.1183	6074.1130	0.0053	12	2	10	1	13	2	15	11	2	9	1	12	2	14
6074.1183	6074.1111	0.0072	12	2	10	2	14	1	15	11	2	9	2	13	1	14
6074.1183	6074.1019	0.0164	12	2	10	2	14	1	14	11	2	9	2	13	1	13
6074.1183	6074.0945	0.0238	12	2	10	2	13	2	15	11	2	9	2	12	2	14
6074.1183	6074.1227	-0.0044	12	2	10	1	13	1	14	11	2	9	1	12	1	13
6074.1183	6074.1015	0.0168	12	2	10	2	14	2	16	11	2	9	2	13	2	15
6074.1183	6074.1020	0.0163	12	2	10	2	14	0	14	11	2	9	2	13	0	13
6074.1183	6074.1101	0.0082	12	2	10	1	12	2	14	11	2	9	1	11	2	13
6074.1183	6074.1104	0.0079	12	2	10	2	14	2	15	11	2	9	2	13	2	14
6074.1183	6074.1118	0.0065	12	2	10	0	12	2	14	11	2	9	0	11	2	13
6268.5250	6268.5462	-0.0212	13	1	13	1	14	1	14	12	1	12	1	13	1	13
6268.5250	6268.5525	-0.0275	13	1	13	1	12	2	14	12	1	12	1	13	0	13
6268.5250	6268.5281	-0.0031	13	1	13	2	13	2	14	12	1	12	2	12	2	13
6268.5250	6268.5308	-0.0058	13	1	13	2	13	1	14	12	1	12	2	12	1	13
6268.5250	6268.5621	-0.0371	13	1	13	1	14	2	15	12	1	12	1	13	2	14
6268.5250	6268.5024	0.0226	13	1	13	2	13	2	15	12	1	12	2	12	2	14
6268.5250	6268.5615	-0.0365	13	1	13	1	14	1	15	12	1	12	1	13	1	14
6268.5250	6268.5462	-0.0212	13	1	13	2	14	2	16	12	1	12	2	13	2	15
6268.5250	6268.5425	-0.0175	13	1	13	1	14	2	16	12	1	12	1	13	2	15
6268.5250	6268.5455	-0.0205	13	1	13	1	14	0	14	12	1	12	1	11	2	13
6268.5841	6268.5691	0.0150	13	1	13	0	13	2	15	12	1	12	0	12	2	14
6268.5841	6268.5810	0.0031	13	3	11	2	13	2	15	12	3	10	2	12	2	14

6268.5841	6268.5800	0.0041	13	1	13	2	15	1	15	12	1	12	2	14	1	14
6268.5841	6268.5744	0.0097	13	1	13	1	13	2	15	12	1	12	1	12	2	14
6268.6250	6268.6012	0.0238	13	1	13	0	13	1	14	12	1	12	0	12	1	13
6268.6250	6268.6011	0.0239	13	1	13	2	15	1	14	12	1	12	2	14	1	13
6268.6250	6268.5883	0.0367	13	1	13	0	13	2	14	12	1	12	0	12	2	13
6268.6250	6268.5856	0.0394	13	1	13	2	15	2	17	12	1	12	2	14	2	16
6268.6250	6268.5976	0.0274	13	1	13	2	15	2	16	12	1	12	2	14	2	15
6268.6250	6268.5919	0.0331	13	1	13	2	15	2	14	12	1	12	2	14	2	13
6268.6250	6268.6046	0.0204	13	1	13	2	15	1	16	12	1	12	2	14	1	15
6268.6250	6268.5820	0.0430	13	1	13	1	14	2	14	12	1	12	1	13	2	13
6268.6250	6268.6174	0.0076	13	1	13	2	15	0	15	12	1	12	2	14	0	14
6268.6250	6268.6025	0.0225	13	1	13	1	13	1	14	12	1	12	1	12	1	13
6275.1500	6275.1755	-0.0255	13	0	13	2	12	1	12	12	0	12	2	11	1	11
6275.1500	6275.1730	-0.0230	13	2	11	2	14	1	14	12	2	10	2	13	1	13
6275.1500	6275.1780	-0.0280	13	0	13	2	13	1	12	12	0	12	2	12	1	11
6275.1500	6275.1847	-0.0347	13	0	13	2	13	2	12	12	0	12	2	12	2	11
6275.1500	6275.1815	-0.0315	13	0	13	2	13	2	14	12	0	12	2	12	2	13
6275.1500	6275.1676	-0.0176	13	0	13	2	12	2	14	12	2	10	2	12	2	13
6275.1668	6275.1953	-0.0285	13	0	13	1	12	2	14	12	0	12	1	11	2	13
6275.1668	6275.1982	-0.0314	13	0	13	1	12	1	12	12	0	12	1	11	1	11
6275.1668	6275.1887	-0.0219	13	0	13	2	14	2	12	12	0	12	2	13	2	11
6275.1668	6275.2007	-0.0339	13	0	13	1	14	2	12	12	0	12	1	11	0	11
6275.1668	6275.1889	-0.0221	13	0	13	1	12	0	12	12	0	12	1	13	2	11
6275.1668	6275.1975	-0.0307	13	0	13	1	14	1	14	12	0	12	1	13	1	13
6275.1668	6275.1909	-0.0241	13	0	13	2	14	0	14	12	0	12	2	13	0	13
6275.1668	6275.1942	-0.0274	13	0	13	1	14	2	16	12	0	12	1	13	2	15
6275.1668	6275.1939	-0.0271	13	0	13	2	14	2	16	12	0	12	2	13	2	15
6275.1668	6275.1868	-0.0200	13	0	13	1	14	0	14	12	0	12	1	13	0	13
6275.2125	6275.2250	-0.0125	13	0	13	1	14	2	15	12	0	12	1	13	2	14
6275.2125	6275.2183	-0.0058	13	0	13	1	14	1	15	12	0	12	1	13	1	14
6275.2125	6275.2300	-0.0175	13	0	13	2	12	1	13	12	0	12	2	11	1	12
6275.2125	6275.2337	-0.0212	13	0	13	1	12	2	13	12	0	12	1	11	2	12
6275.2125	6275.2281	-0.0156	13	0	13	1	14	1	13	12	0	12	1	13	1	12
6275.2125	6275.2268	-0.0143	13	2	11	1	12	2	13	12	2	10	1	11	2	12
6275.2125	6275.2302	-0.0177	13	2	11	2	12	1	13	12	2	10	2	11	1	12
6275.2220	6275.2336	-0.0116	13	0	13	2	14	1	13	12	0	12	2	13	1	12
6275.2220	6275.2361	-0.0141	13	0	13	0	13	2	15	12	0	12	2	13	2	14
6275.2220	6275.2356	-0.0136	13	0	13	2	14	1	15	12	0	12	2	13	1	14
6275.2220	6275.2317	-0.0097	13	0	13	1	13	1	13	12	0	12	1	11	1	12
6275.2220	6275.2378	-0.0158	13	0	13	1	14	2	13	12	0	12	1	13	2	12
6275.2220	6275.2463	-0.0243	13	0	13	2	15	0	15	12	0	12	2	14	0	14
6275.2220	6275.2355	-0.0135	13	0	13	1	12	1	13	12	0	12	1	12	1	12
6275.2220	6275.2464	-0.0244	13	0	13	1	13	2	15	12	0	12	1	12	2	14
6275.2220	6275.2443	-0.0223	13	0	13	2	15	1	15	12	0	12	2	14	1	14
6275.2220	6275.2494	-0.0274	13	0	13	0	13	0	13	12	0	12	0	12	0	12
6275.2250	6275.2503	-0.0253	13	2	11	1	13	2	12	12	2	10	1	12	2	11
6275.2250	6275.2508	-0.0258	13	2	11	2	13	2	15	12	0	12	2	12	2	14
6275.2250	6275.2512	-0.0262	13	0	13	1	14	2	14	12	0	12	1	13	2	13
6275.2250	6275.2590	-0.0340	13	0	13	2	15	2	17	12	0	12	2	14	2	16
6275.2250	6275.2349	-0.0099	13	0	13	2	15	2	13	12	0	12	2	14	2	12
6275.2375	6275.2551	-0.0176	13	0	13	2	11	2	12	12	2	10	2	11	2	11
6275.2375	6275.2614	-0.0239	13	0	13	1	13	2	14	12	0	12	1	12	2	13
6275.2375	6275.2557	-0.0182	13	0	13	2	11	2	11	12	0	12	2	10	2	10

6275.2375	6275.2613	-0.0238	13	0	13	2	14	2	14	12	0	12	2	13	2	13
6275.2375	6275.2664	-0.0289	13	0	13	0	13	2	14	12	0	12	0	12	2	13
6275.2375	6275.2596	-0.0221	13	0	13	2	12	2	12	12	0	12	2	11	2	11
6275.2375	6275.2659	-0.0284	13	0	13	2	11	2	9	12	0	12	2	10	2	8
6275.2375	6275.2526	-0.0151	13	0	13	2	12	2	11	12	0	12	0	12	2	10
6275.2625	6275.2811	-0.0186	13	0	13	2	11	1	12	12	0	12	2	10	1	11
6275.2625	6275.2762	-0.0137	13	0	13	1	13	2	12	12	0	12	1	12	2	11
6275.2625	6275.2832	-0.0207	13	0	13	2	15	1	16	12	0	12	2	14	1	15
6275.2625	6275.2807	-0.0182	13	0	13	2	15	1	14	12	0	12	2	14	1	13
6275.2625	6275.2788	-0.0163	13	0	13	1	13	1	12	12	0	12	1	12	1	11
6275.2625	6275.2769	-0.0144	13	0	13	2	15	2	16	12	0	12	2	14	2	15
6275.2625	6275.2830	-0.0205	13	0	13	0	13	1	14	12	0	12	2	12	1	13
6275.2625	6275.2822	-0.0197	13	0	13	0	13	2	12	12	0	12	0	12	2	11
6275.2625	6275.2830	-0.0205	13	0	13	1	13	1	14	12	0	12	1	12	1	13
6275.2625	6275.2737	-0.0112	13	0	13	2	15	2	14	12	0	12	2	14	2	13
6275.2875	6275.3013	-0.0138	13	0	13	0	13	2	13	12	0	12	0	12	2	12
6275.2875	6275.3009	-0.0134	13	0	13	2	11	0	11	12	0	12	2	10	0	10
6275.2875	6275.3018	-0.0143	13	0	13	1	13	0	13	12	0	12	1	12	0	12
6275.2875	6275.3019	-0.0144	13	0	13	2	15	2	15	12	0	12	2	14	2	14
6402.4375	6402.3998	0.0377	13	2	12	2	13	2	15	12	2	11	2	12	2	14
6402.4375	6402.4018	0.0357	13	2	12	2	13	1	13	12	2	11	2	12	1	12
6402.4375	6402.4022	0.0353	13	2	12	2	13	0	13	12	2	11	2	12	0	12
6402.4375	6402.4049	0.0326	13	2	12	2	13	2	11	12	2	11	2	12	2	10
6402.4625	6402.4323	0.0302	13	4	10	0	13	2	12	12	4	9	0	12	2	11
6402.4625	6402.4256	0.0369	13	2	12	2	13	2	14	12	2	11	2	12	2	13
6402.4625	6402.4239	0.0386	13	2	12	2	13	1	12	12	2	11	2	12	1	11
6402.4625	6402.4256	0.0369	13	2	12	2	13	1	14	12	2	11	2	12	1	13
6402.4875	6402.4550	0.0325	13	2	12	2	14	1	14	12	2	11	2	13	1	13
6402.4875	6402.4543	0.0332	13	2	12	2	14	2	16	12	2	11	2	13	2	15
6402.4875	6402.4620	0.0255	13	2	12	1	14	2	16	12	2	11	1	13	2	15
6402.4875	6402.4555	0.0320	13	2	12	2	14	0	14	12	2	11	2	13	0	13
6402.4875	6402.4644	0.0231	13	2	12	1	14	0	14	12	2	11	1	13	0	13
6402.4875	6402.4578	0.0297	13	2	12	1	12	2	14	12	2	11	1	11	2	13
6402.4875	6402.4639	0.0236	13	2	12	1	14	1	14	12	2	11	1	13	1	13
6402.5086	6402.4819	0.0267	13	2	12	1	12	2	13	12	2	11	1	11	2	12
6402.5086	6402.4937	0.0149	13	2	12	2	12	1	13	12	2	11	2	11	1	12
6402.5086	6402.4839	0.0247	13	2	12	1	12	1	13	12	2	11	1	11	1	12
6402.5086	6402.4870	0.0216	13	2	12	1	14	1	13	12	2	11	1	13	1	12
6402.5086	6402.4862	0.0224	13	2	12	1	14	2	15	12	2	11	1	13	2	14
6402.5086	6402.4886	0.0200	13	2	12	1	14	1	15	12	2	11	1	13	1	14
6402.5086	6402.4799	0.0287	13	2	12	2	14	1	15	12	2	11	2	13	1	14
6402.5086	6402.4671	0.0415	13	2	12	2	12	2	14	12	2	11	2	11	2	13
6402.5086	6402.4779	0.0307	13	2	12	2	14	2	15	12	2	11	2	13	2	14
6402.5250	6402.5176	0.0074	13	2	12	2	15	1	15	12	2	11	2	14	1	14
6402.5250	6402.5211	0.0039	13	2	12	0	13	2	15	12	2	11	0	12	2	14
6402.5250	6402.5133	0.0117	13	2	12	1	14	2	14	12	2	11	1	13	2	13
6402.5250	6402.5254	-0.0004	13	2	12	2	11	2	13	12	2	11	2	11	2	12
6402.5250	6402.5167	0.0083	13	2	12	2	15	2	17	12	2	11	2	14	2	16
6402.5250	6402.5388	-0.0138	13	2	12	2	15	2	16	12	2	11	2	14	2	15
6402.5250	6402.5208	0.0042	13	2	12	1	13	2	15	12	2	11	1	12	2	14
6402.5250	6402.5039	0.0211	13	4	10	2	14	0	14	12	4	9	2	13	0	13
6402.5250	6402.5180	0.0070	13	2	12	2	15	0	15	12	2	11	2	14	0	14
6402.5250	6402.5405	-0.0155	13	2	12	2	15	1	14	12	2	11	2	14	1	13

6402.5500	6402.5479	0.0021	13	2	12	0	13	1	14	12	2	11	0	12	1	13
6402.5500	6402.5455	0.0045	13	2	12	1	13	1	12	12	2	11	1	12	1	11
6402.5500	6402.5475	0.0025	13	2	12	1	13	1	14	12	2	11	1	12	1	13
6402.5500	6402.5438	0.0062	13	2	12	2	15	2	14	12	2	11	2	14	2	13
6402.5500	6402.5524	-0.0024	13	2	12	2	11	1	12	12	2	11	2	10	1	11
6402.5500	6402.5433	0.0067	13	2	12	2	15	1	16	12	2	11	2	14	1	15
6402.5500	6402.5434	0.0066	13	2	12	0	13	2	14	12	2	11	0	12	2	13
6402.5500	6402.5430	0.0070	13	2	12	1	13	2	14	12	2	11	1	12	2	13
6402.5750	6402.5754	-0.0004	13	2	12	2	11	2	11	12	2	11	2	10	2	10
6402.5750	6402.5701	0.0049	13	2	12	1	13	2	13	12	2	11	1	12	2	12
6402.5750	6402.5705	0.0045	13	2	12	0	13	2	13	12	2	11	0	12	2	12
6402.5750	6402.5538	0.0212	13	2	12	2	11	2	10	12	2	11	2	10	2	9
6402.5750	6402.5657	0.0093	13	2	12	2	15	2	15	12	2	11	2	14	2	14
6463.8430	6463.8443	-0.0013	13	1	12	1	12	1	11	12	1	11	1	11	1	10
6463.8430	6463.8434	-0.0004	13	1	12	1	12	1	13	12	1	11	1	11	1	12
6463.8430	6463.8308	0.0122	13	1	12	2	14	1	15	12	1	11	2	13	1	14
6463.8430	6463.8934	-0.0504	13	1	12	1	12	2	12	12	3	9	1	11	0	11
6463.8430	6463.8627	-0.0197	13	1	12	1	14	2	15	12	1	11	1	13	2	14
6463.8430	6463.8536	-0.0106	13	1	12	2	15	2	16	12	1	11	2	13	2	15
6463.8430	6463.8604	-0.0174	13	1	12	1	14	1	15	12	1	11	1	13	1	14
6463.8430	6463.8444	-0.0014	13	1	12	1	12	2	11	12	1	11	1	11	2	10
6463.8430	6463.8621	-0.0191	13	1	12	1	14	1	13	12	1	11	1	13	1	12
6463.8430	6463.8817	-0.0387	13	1	12	2	12	1	11	12	1	11	2	11	1	10
6463.9772	6463.9763	0.0009	13	1	12	1	13	2	15	12	1	11	1	12	2	14
6463.9772	6463.9769	0.0003	13	1	12	0	13	1	13	12	1	11	0	12	1	12
6463.9772	6463.9755	0.0017	13	1	12	1	13	0	13	12	1	11	1	12	0	12
6463.9772	6463.9747	0.0025	13	1	12	1	13	2	11	12	1	11	1	12	2	10
6463.9772	6464.0087	-0.0315	13	1	12	2	15	1	16	12	1	11	2	14	1	15
6463.9772	6463.9604	0.0168	13	1	12	2	15	1	15	12	1	11	2	14	1	14
6463.9772	6463.9933	-0.0161	13	1	12	2	11	1	11	12	1	11	2	10	1	10
6463.9772	6463.9401	0.0371	13	1	12	2	14	2	16	12	1	11	2	14	2	15
6463.9772	6463.9753	0.0019	13	1	12	1	13	1	13	12	1	11	1	12	1	12
6463.9772	6463.9619	0.0153	13	1	12	2	15	2	17	12	1	11	2	14	2	16
6469.5467	6469.5324	0.0143	13	7	6	2	15	2	17	12	7	5	2	14	2	16
6469.5467	6469.5280	0.0187	13	7	6	2	15	1	15	12	7	5	2	14	1	14
6469.5467	6469.5277	0.0190	13	7	7	2	15	1	15	12	7	6	2	14	1	14
6469.5467	6469.5274	0.0193	13	7	7	2	15	0	15	12	7	6	2	14	0	14
6469.5467	6469.5321	0.0146	13	7	7	2	15	2	17	12	7	6	2	14	2	16
6469.5467	6469.5271	0.0196	13	7	6	0	13	2	15	12	7	5	0	12	2	14
6469.5467	6469.5272	0.0195	13	7	7	1	13	2	15	12	7	6	1	12	2	14
6469.5467	6469.5276	0.0191	13	7	6	2	15	0	15	12	7	5	2	14	0	14
6469.5467	6469.5275	0.0192	13	7	6	1	13	2	15	12	7	5	1	12	2	14
6469.5467	6469.5268	0.0199	13	7	7	0	13	2	15	12	7	6	0	12	2	14
6469.6487	6469.6205	0.0282	13	7	7	2	14	2	16	12	7	6	2	13	2	15
6469.6487	6469.6079	0.0408	13	7	6	1	14	2	16	12	7	5	1	13	2	15
6469.6487	6469.6076	0.0411	13	7	7	1	14	2	16	12	7	6	1	13	2	15
6469.6487	6469.5961	0.0526	13	7	6	2	15	1	16	12	7	5	2	14	1	15
6469.6487	6469.6035	0.0452	13	7	6	2	15	2	16	12	7	5	2	14	2	15
6469.6487	6469.6032	0.0455	13	7	7	2	15	2	16	12	7	6	2	14	2	15
6469.6487	6469.6208	0.0279	13	7	6	2	14	2	16	12	7	5	2	13	2	15
6469.6487	6469.6037	0.0450	13	7	6	1	14	1	14	12	7	5	1	13	1	13
6469.6487	6469.5958	0.0529	13	7	7	2	15	1	16	12	7	6	2	14	1	15
6469.6487	6469.6034	0.0453	13	7	7	1	14	1	14	12	7	6	1	13	1	13

6472.1724	6472.1531	0.0193	13	3	11	2	15	0	15	12	3	10	2	14	0	14
6472.1724	6472.1458	0.0266	13	3	11	2	14	2	16	12	3	10	2	13	2	15
6472.1724	6472.1712	0.0012	13	3	11	1	14	1	15	12	3	10	1	13	1	14
6472.1724	6472.1523	0.0201	13	3	11	2	15	2	17	12	3	10	2	14	2	16
6472.1724	6472.1721	0.0003	13	3	11	2	15	1	16	12	3	10	2	14	1	15
6472.1724	6472.1514	0.0210	13	3	11	1	14	2	16	12	3	10	1	13	2	15
6472.1724	6472.1694	0.0030	13	3	11	2	15	2	16	12	3	10	2	14	2	15
6472.1724	6472.1897	-0.0173	13	3	11	2	15	2	15	12	1	12	2	14	2	14
6472.1724	6472.1529	0.0195	13	3	11	2	15	1	15	12	3	10	2	14	1	14
6472.1724	6472.1553	0.0171	13	3	11	1	13	2	15	12	3	10	1	12	2	14
6472.5625	6472.5567	0.0058	13	6	8	2	15	1	15	12	6	7	2	14	1	14
6472.5625	6472.5593	0.0032	13	6	8	2	15	2	17	12	6	7	2	14	2	16
6472.5625	6472.5538	0.0087	13	6	8	2	15	2	13	12	6	7	2	14	2	12
6472.5625	6472.5533	0.0092	13	6	8	1	13	1	13	12	6	7	1	12	1	12
6472.5625	6472.5531	0.0094	13	6	8	0	13	1	13	12	6	7	0	12	1	12
6472.5625	6472.5531	0.0094	13	6	8	1	13	0	13	12	6	7	1	12	0	12
6472.5625	6472.5530	0.0095	13	6	8	0	13	0	13	12	6	7	0	12	0	12
6472.5625	6472.5560	0.0065	13	6	8	1	13	2	15	12	6	7	1	12	2	14
6472.5625	6472.5558	0.0067	13	6	8	0	13	2	15	12	6	7	0	12	2	14
6472.5625	6472.5565	0.0060	13	6	8	2	15	0	15	12	6	7	2	14	0	14
6472.5750	6472.5678	0.0072	13	6	7	1	13	1	13	12	6	6	1	12	1	12
6472.5750	6472.5676	0.0074	13	4	9	0	13	1	13	12	6	6	0	12	1	12
6472.5750	6472.5674	0.0076	13	6	7	0	13	0	13	12	6	6	0	12	0	12
6472.5750	6472.5676	0.0074	13	6	7	1	13	0	13	12	6	6	1	12	0	12
6472.5750	6472.5705	0.0045	13	6	7	1	13	2	15	12	6	6	1	12	2	14
6472.5750	6472.5683	0.0067	13	6	7	2	15	2	13	12	6	6	2	14	2	12
6472.5750	6472.5669	0.0081	13	6	7	2	11	2	13	12	6	6	2	10	2	12
6472.5750	6472.5712	0.0038	13	6	7	2	15	1	15	12	6	6	2	14	1	14
6472.5750	6472.5710	0.0040	13	6	7	2	15	0	15	12	6	6	2	14	0	14
6472.5750	6472.5703	0.0047	13	6	7	0	13	2	15	12	6	6	0	12	2	14
6472.6000	6472.6061	-0.0061	13	6	8	1	13	2	14	12	6	7	1	12	2	13
6472.6000	6472.6042	-0.0042	13	6	8	0	13	1	12	12	6	7	0	12	1	11
6472.6000	6472.6044	-0.0044	13	6	8	1	13	1	12	12	6	7	1	12	1	11
6472.6000	6472.6075	-0.0075	13	6	8	2	15	1	14	12	6	7	2	14	1	13
6472.6000	6472.6015	-0.0015	13	8	6	0	13	1	14	12	6	7	0	12	1	13
6472.6000	6472.6028	-0.0028	13	6	8	2	11	2	12	12	6	7	2	10	2	11
6472.6000	6472.6019	-0.0019	13	6	8	2	15	2	14	12	6	7	2	14	2	13
6472.6000	6472.6016	-0.0016	13	6	8	1	13	1	14	12	6	7	1	12	1	13
6472.6000	6472.6059	-0.0059	13	6	8	0	13	2	14	12	6	7	0	12	2	13
6472.6000	6472.6055	-0.0055	13	6	8	2	15	1	16	12	6	7	2	14	1	15
6472.6500	6472.6377	0.0123	13	6	7	1	12	1	12	12	6	6	1	11	1	11
6472.6500	6472.6389	0.0111	13	6	7	2	14	2	12	12	6	6	2	13	2	11
6472.6500	6472.6366	0.0134	13	6	7	1	14	2	16	12	6	6	1	13	2	15
6472.6500	6472.6375	0.0125	13	6	7	1	12	0	12	12	6	6	1	11	0	11
6472.6500	6472.6343	0.0157	13	6	7	1	14	0	14	12	6	6	1	13	0	13
6472.6500	6472.6322	0.0178	13	6	7	2	12	0	12	12	6	6	2	11	0	11
6472.6500	6472.6306	0.0194	13	6	8	2	14	2	16	12	6	7	2	13	2	15
6472.6500	6472.6344	0.0156	13	6	7	1	14	1	14	12	6	6	1	13	1	13
6472.6500	6472.6318	0.0182	13	6	7	1	14	2	12	12	6	6	1	13	2	11
6472.6500	6472.6345	0.0155	13	6	7	2	12	2	14	12	4	8	2	11	2	13
6472.6750	6472.6737	0.0013	13	6	8	1	14	2	15	12	6	7	1	13	2	14
6472.6750	6472.6749	0.0001	13	6	8	2	14	1	15	12	6	7	2	13	1	14
6472.6750	6472.6702	0.0048	13	6	7	2	15	2	15	12	6	6	2	14	2	14

6472.6750	6472.6720	0.0030	13	6	8	2	14	2	13	12	6	7	2	13	2	12
6472.6750	6472.6711	0.0039	13	6	8	1	14	1	13	12	6	7	1	13	1	12
6472.6750	6472.6683	0.0067	13	6	8	1	14	1	15	12	6	7	1	13	1	14
6472.6750	6472.6660	0.0090	13	4	9	1	13	2	13	12	4	8	1	12	2	12
6472.6750	6472.6715	0.0035	13	6	8	1	12	1	13	12	6	7	1	11	1	12
6472.6750	6472.6668	0.0082	13	6	8	1	14	2	13	12	6	7	1	13	2	12
6472.6750	6472.6659	0.0091	13	6	7	0	13	2	13	12	6	6	0	12	2	12
6472.7000	6472.6856	0.0144	13	6	7	1	14	1	13	12	6	6	1	13	1	12
6472.7000	6472.6939	0.0061	13	4	9	2	14	2	15	12	6	6	2	13	2	14
6472.7000	6472.6882	0.0118	13	6	7	1	14	2	15	12	6	6	1	13	2	14
6472.7000	6472.6912	0.0088	13	6	7	2	14	1	13	12	6	6	2	13	1	12
6472.7000	6472.6908	0.0092	13	6	8	2	13	0	13	12	6	7	2	12	0	12
6472.7000	6472.6940	0.0060	13	6	8	2	13	2	15	12	6	7	2	12	2	14
6472.7000	6472.6895	0.0105	13	6	7	2	14	1	15	12	6	6	2	13	1	14
6472.7000	6472.6910	0.0090	13	6	8	2	13	1	13	12	6	7	2	12	1	12
6472.7000	6472.6861	0.0139	13	6	7	1	12	1	13	12	6	6	1	11	1	12
6472.7000	6472.6924	0.0076	13	8	5	1	12	2	13	12	8	4	1	11	2	12
6477.9750	6477.9763	-0.0013	13	5	8	0	13	1	13	12	5	7	0	12	1	12
6477.9750	6477.9763	-0.0013	13	5	8	1	13	0	13	12	5	7	1	12	0	12
6477.9750	6477.9763	-0.0013	13	5	8	1	13	1	13	12	5	7	1	12	1	12
6477.9750	6477.9783	-0.0033	13	5	8	2	15	0	15	12	5	7	2	14	0	14
6477.9750	6477.9773	-0.0023	13	5	8	1	13	2	15	12	5	7	1	12	2	14
6477.9750	6477.9793	-0.0043	13	5	8	2	15	2	17	12	5	7	2	14	2	16
6477.9750	6477.9783	-0.0033	13	5	8	2	15	1	15	12	5	7	2	14	1	14
6477.9750	6477.9774	-0.0024	13	5	8	2	15	2	13	12	5	7	2	14	2	12
6477.9750	6477.9773	-0.0023	13	5	8	0	13	2	15	12	5	7	0	12	2	14
6477.9750	6477.9764	-0.0014	13	3	10	0	13	0	13	12	5	7	0	12	0	12
6478.0000	6478.0087	-0.0087	13	5	8	0	13	1	12	12	5	7	0	12	1	11
6478.0000	6478.0077	-0.0077	13	5	8	1	13	1	14	12	5	7	1	12	1	13
6478.0000	6478.0100	-0.0100	13	5	8	0	13	2	14	12	5	7	0	12	2	13
6478.0000	6478.0099	-0.0099	13	5	8	1	13	2	14	12	5	7	1	12	2	13
6478.0000	6478.0095	-0.0095	13	5	8	2	15	1	16	12	5	7	2	14	1	15
6478.0000	6478.0106	-0.0106	13	5	8	2	15	1	14	12	5	7	2	14	1	13
6478.0000	6478.0077	-0.0077	13	5	8	0	13	1	14	12	7	5	0	12	1	13
6478.0000	6478.0086	-0.0086	13	5	8	1	13	1	12	12	5	7	1	12	1	11
6478.0000	6478.0117	-0.0117	13	5	8	2	15	2	16	12	5	7	2	14	2	15
6478.0000	6478.0087	-0.0087	13	5	8	2	15	2	14	12	5	7	2	14	2	13
6478.0500	6478.0335	0.0165	13	5	8	2	12	2	14	12	5	7	2	11	2	13
6478.0500	6478.0348	0.0152	13	5	8	1	14	1	14	12	5	7	1	13	1	13
6478.0500	6478.0374	0.0126	13	5	8	1	12	2	14	12	5	7	1	11	2	13
6478.0500	6478.0403	0.0097	13	5	8	0	13	2	13	12	5	7	0	12	2	12
6478.0500	6478.0384	0.0116	13	5	8	2	14	1	14	12	5	7	2	13	1	13
6478.0500	6478.0348	0.0152	13	5	8	1	14	0	14	12	5	7	1	13	0	13
6478.0500	6478.0394	0.0106	13	5	8	2	14	2	16	12	5	7	2	13	2	15
6478.0500	6478.0418	0.0082	13	5	8	2	15	2	15	12	5	7	2	14	2	14
6478.0500	6478.0359	0.0141	13	5	8	1	14	2	16	12	5	7	1	13	2	15
6478.0500	6478.0384	0.0116	13	5	8	2	14	0	14	12	5	7	2	13	0	13
6478.0685	6478.0680	0.0005	13	5	8	1	14	2	15	12	5	7	1	13	2	14
6478.0685	6478.0713	-0.0028	13	5	8	2	14	1	13	12	5	7	2	13	1	12
6478.0685	6478.0661	0.0024	13	5	8	1	14	1	15	12	5	7	1	13	1	14
6478.0685	6478.0679	0.0006	13	5	8	1	12	1	13	12	5	7	1	11	1	12
6478.0685	6478.0670	0.0015	13	5	8	1	14	1	13	12	5	7	1	13	1	12
6478.0685	6478.0648	0.0037	13	5	8	1	14	2	13	12	5	7	1	13	2	12

6478.0685	6478.0644	0.0041	13	5	8	2	12	1	13	12	5	7	2	11	1	12
6478.0685	6478.0697	-0.0012	13	5	8	1	12	2	13	12	5	7	1	11	2	12
6478.0685	6478.0725	-0.0040	13	5	8	2	14	2	15	12	5	7	2	13	2	14
6478.0685	6478.0699	-0.0014	13	5	8	2	14	1	15	12	5	7	2	13	1	14
6478.0898	6478.0983	-0.0085	13	5	8	1	14	2	14	12	5	7	1	13	2	13
6478.0898	6478.1024	-0.0126	13	7	6	2	14	0	14	12	5	7	2	13	2	13
6478.0898	6478.0956	-0.0058	13	5	8	2	13	2	15	12	5	7	2	12	2	14
6478.0898	6478.0941	-0.0043	13	5	8	2	13	2	11	12	5	7	2	12	2	10
6478.0898	6478.0973	-0.0075	13	5	8	2	12	2	12	12	5	7	2	11	2	11
6478.0898	6478.0949	-0.0051	13	5	8	2	13	0	13	12	5	7	2	12	0	12
6478.0898	6478.0948	-0.0050	13	5	8	2	13	1	13	12	5	7	2	12	1	12
6478.0898	6478.1002	-0.0104	13	5	8	1	12	2	12	12	3	9	1	11	2	11
6478.1125	6478.1275	-0.0150	13	5	8	2	13	1	12	12	5	7	2	12	1	11
6478.1125	6478.1266	-0.0141	13	5	8	2	13	1	14	12	3	9	2	12	1	13
6478.1125	6478.1284	-0.0159	13	5	8	2	13	2	14	12	5	7	2	12	2	13
6478.1125	6478.1252	-0.0127	13	5	8	2	13	2	12	12	5	7	2	12	2	11
6492.5625	6492.5728	-0.0103	13	2	11	0	13	1	13	12	4	8	0	12	1	12
6492.5625	6492.5726	-0.0101	13	4	9	1	13	0	13	12	4	8	1	12	0	12
6492.5625	6492.5723	-0.0098	13	4	9	1	13	1	13	12	4	8	1	12	1	12
6492.5625	6492.5746	-0.0121	13	4	9	2	15	2	13	12	4	8	2	14	2	12
6492.5625	6492.5735	-0.0110	13	4	9	2	15	1	15	12	4	8	2	14	1	14
6492.5625	6492.5714	-0.0089	13	4	9	1	13	2	15	12	4	8	1	12	2	14
6492.5625	6492.5728	-0.0103	13	4	9	2	15	2	17	12	4	8	2	14	2	16
6492.5625	6492.5731	-0.0106	13	4	9	0	13	0	13	12	4	8	0	12	0	12
6492.5625	6492.5737	-0.0112	13	4	9	2	15	0	15	12	4	8	2	14	0	14
6492.5625	6492.5720	-0.0095	13	4	9	0	13	2	15	12	4	8	0	12	2	14
6492.5876	6492.5843	0.0033	13	4	9	2	15	1	16	12	4	8	2	14	1	15
6492.5876	6492.5835	0.0041	13	4	9	0	13	1	12	12	4	8	0	12	1	11
6492.5876	6492.5841	0.0035	13	4	9	0	13	2	14	12	4	8	0	12	2	13
6492.5876	6492.5841	0.0035	13	4	9	0	13	1	14	12	2	10	0	12	1	13
6492.5876	6492.5837	0.0039	13	4	9	1	13	1	14	12	4	8	1	12	1	13
6492.5876	6492.5829	0.0047	13	4	9	1	13	1	12	12	4	8	1	12	1	11
6492.5876	6492.5845	0.0031	13	4	9	2	15	1	14	12	4	8	2	14	1	13
6492.5876	6492.5865	0.0011	13	4	9	2	15	2	14	12	4	8	2	14	2	13
6492.5876	6492.5834	0.0042	13	4	9	1	13	2	14	12	4	8	1	12	2	13
6492.5876	6492.5848	0.0028	13	4	9	2	15	2	16	12	4	8	2	14	2	15
6492.6375	6492.6556	-0.0181	13	4	9	1	14	2	15	12	4	8	1	13	2	14
6492.6375	6492.6460	-0.0085	13	4	9	1	14	1	14	12	4	8	1	13	1	13
6492.6375	6492.6571	-0.0196	13	4	9	1	14	1	15	12	4	8	1	13	1	14
6492.6375	6492.6508	-0.0133	13	6	7	2	14	0	14	12	6	6	2	13	0	13
6492.6375	6492.6505	-0.0130	13	6	7	2	14	1	14	12	6	6	2	13	1	13
6492.6375	6492.6471	-0.0096	13	4	9	1	12	2	14	12	4	8	1	11	2	13
6492.6375	6492.6460	-0.0085	13	4	9	1	14	0	14	12	4	8	1	13	0	13
6492.6375	6492.6454	-0.0079	13	4	9	2	12	2	14	12	2	10	2	11	2	13
6492.6375	6492.6493	-0.0118	13	4	9	2	14	2	16	12	4	8	2	13	2	15
6492.6375	6492.6456	-0.0081	13	4	9	1	14	2	16	12	4	8	1	13	2	15
6492.6625	6492.6603	0.0022	13	4	9	1	12	1	13	12	4	8	1	11	1	12
6492.6625	6492.6636	-0.0011	13	2	11	2	14	2	15	12	4	8	2	13	2	14
6492.6625	6492.6562	0.0063	13	4	9	1	14	1	13	12	4	8	1	13	1	12
6492.6625	6492.6634	-0.0009	13	4	9	2	14	1	15	12	4	8	2	13	1	14
6492.6625	6492.6674	-0.0049	13	4	9	1	14	2	14	12	4	8	1	13	2	13
6492.7375	6492.7241	0.0134	13	4	9	2	13	2	11	12	4	8	2	12	2	10
6492.7375	6492.7209	0.0166	13	6	7	2	13	2	15	12	4	8	2	12	2	14

6492.7375	6492.7222	0.0153	13	4	9	2	13	1	13	12	2	10	2	12	1	12
6492.7375	6492.7226	0.0149	13	4	9	2	13	0	13	12	4	8	2	12	0	12
6492.7625	6492.7351	0.0274	13	4	9	2	13	1	12	12	4	8	2	12	1	11
6492.7625	6492.7329	0.0296	13	4	9	2	13	2	14	12	6	6	2	12	2	13
6492.7625	6492.7360	0.0265	13	6	7	0	13	1	14	12	4	8	2	12	1	13
6492.7625	6492.7352	0.0273	13	2	11	2	13	2	12	12	2	10	2	12	2	11
6545.4807	6545.4840	-0.0033	13	3	10	2	15	2	17	12	3	9	2	14	2	16
6545.4807	6545.4864	-0.0057	13	3	10	2	15	0	15	12	3	9	2	14	0	14
6545.4807	6545.4717	0.0090	13	3	10	2	15	1	16	12	3	9	2	14	1	15
6545.4807	6545.4862	-0.0055	13	3	10	2	15	1	15	12	3	9	2	14	1	14
6545.4807	6545.4683	0.0124	13	3	10	2	15	2	16	12	3	9	2	14	2	15
6545.4807	6545.4811	-0.0004	13	3	10	0	13	2	15	12	3	9	0	12	2	14
6545.4807	6545.4560	0.0247	13	3	10	2	15	2	15	12	3	9	2	14	2	14
6545.4807	6545.4698	0.0109	13	3	10	2	15	1	14	12	3	9	2	14	1	13
6545.4807	6545.4807	-0.0000	13	3	10	1	13	2	15	12	3	9	1	12	2	14
6545.4807	6545.4692	0.0115	13	3	10	1	13	1	14	12	3	9	1	12	1	13
6545.6055	6545.6068	-0.0013	13	3	10	2	14	2	16	12	3	9	2	13	2	15
6545.6055	6545.5905	0.0150	13	3	10	1	14	1	15	12	3	9	1	13	1	14
6545.6055	6545.5858	0.0197	13	3	10	1	14	2	15	12	3	9	1	13	2	14
6545.6055	6545.6028	0.0027	13	3	10	1	14	2	16	12	3	9	1	13	2	15
6545.6055	6545.5926	0.0129	13	3	10	2	14	2	15	12	3	9	2	13	2	14
6545.6055	6545.6049	0.0006	13	3	10	1	14	1	14	12	3	9	1	13	1	13
6545.6055	6545.6051	0.0004	13	3	10	1	14	0	14	12	3	9	1	13	0	13
6545.6055	6545.5966	0.0089	13	3	10	2	14	1	15	12	3	9	2	13	1	14
6545.6055	6545.5738	0.0317	13	3	10	1	14	2	14	12	3	9	1	13	2	13
6545.6055	6545.6042	0.0013	13	3	10	1	12	2	14	12	3	9	1	11	2	13
6545.7338	6545.7310	0.0028	13	3	10	2	13	2	11	12	3	9	2	12	2	10
6545.7338	6545.7176	0.0162	13	3	10	2	13	2	12	12	3	9	2	12	2	11
6545.7338	6545.7255	0.0083	13	3	10	2	13	2	15	12	3	9	2	12	2	14
6545.7338	6545.7001	0.0337	13	3	10	2	13	2	13	12	3	9	2	12	2	12
6545.7338	6545.7130	0.0208	13	3	10	2	13	1	12	12	3	9	2	12	1	11
6545.7338	6545.7099	0.0239	13	3	10	2	13	2	14	12	3	9	2	12	2	13
6545.7338	6545.7283	0.0055	13	3	10	2	13	0	13	12	3	9	2	12	0	12
6545.7338	6545.7279	0.0059	13	3	10	2	13	1	13	12	3	9	2	12	1	12
6545.7338	6545.7157	0.0181	13	3	10	2	13	1	14	12	3	9	2	12	1	13
6577.4250	6577.4627	-0.0377	13	2	11	1	14	2	15	12	2	10	1	13	2	14
6577.4250	6577.4437	-0.0187	13	4	9	2	14	2	15	12	2	10	2	13	2	14
6577.4250	6577.4136	0.0114	13	2	11	2	13	2	15	12	2	10	2	12	2	14
6577.4250	6577.4285	-0.0035	13	2	11	2	14	2	16	12	2	10	2	13	2	15
6577.4250	6577.4437	-0.0187	13	2	11	2	14	1	15	12	2	10	2	13	1	14
6577.4750	6577.4645	0.0105	13	0	13	2	15	0	15	12	2	10	2	14	0	14
6577.4750	6577.4483	0.0267	13	2	11	1	14	2	16	12	2	10	1	13	2	15
6577.4750	6577.4629	0.0121	13	2	11	1	14	1	15	12	2	10	1	13	1	14
6577.4750	6577.4644	0.0106	13	2	11	2	15	1	15	12	2	10	2	14	1	14
6577.4750	6577.4644	0.0106	13	2	11	2	15	2	17	12	2	10	2	14	2	16
6577.5125	6577.4774	0.0351	13	2	11	1	14	2	14	12	2	10	1	13	2	13
6577.5125	6577.4754	0.0371	13	2	11	0	13	2	15	12	2	10	0	12	2	14
6577.5125	6577.4936	0.0189	13	2	11	2	15	2	15	12	2	10	2	14	2	14
6577.5125	6577.4888	0.0237	13	2	11	1	13	1	14	12	2	10	1	12	1	13
6577.5125	6577.4903	0.0222	13	2	11	0	13	1	14	12	2	10	0	12	1	13
6577.5125	6577.4790	0.0335	13	2	11	2	15	1	16	12	2	10	2	14	1	15
6577.5125	6577.4739	0.0386	13	2	11	1	13	2	15	12	2	10	1	12	2	14
6577.5125	6577.4789	0.0336	13	2	11	2	15	1	14	12	2	10	2	14	1	13

6577.5125	6577.4793	0.0332	13	2	11	2	15	2	14	12	2	10	2	14	2	13
6577.5125	6577.4790	0.0335	13	2	11	2	15	2	16	12	2	10	2	14	2	15
6745.6125	6745.5922	0.0203	14	1	14	2	14	2	16	13	1	13	2	13	2	15
6745.6125	6745.6000	0.0125	14	1	14	2	14	2	12	13	1	13	0	13	2	11
6745.6125	6745.5955	0.0170	14	1	14	2	14	1	14	13	1	13	0	13	1	13
6745.6125	6745.5961	0.0164	14	1	14	2	14	0	14	13	1	13	2	13	0	13
6745.6250	6745.6158	0.0092	14	1	14	2	14	1	13	13	1	13	2	13	1	12
6745.6250	6745.6178	0.0072	14	1	14	2	13	1	13	13	1	13	2	12	1	12
6745.6250	6745.6215	0.0035	14	1	14	2	14	2	15	13	1	13	2	13	2	14
6745.6250	6745.6199	0.0051	14	3	12	2	15	1	15	13	3	11	2	14	1	14
6745.6250	6745.6224	0.0026	14	1	14	2	13	0	13	13	1	13	2	12	0	12
6745.6375	6745.6325	0.0050	14	1	14	1	15	1	15	13	1	13	1	14	1	14
6745.6375	6745.6352	0.0023	14	1	14	1	13	1	13	13	1	13	1	12	1	12
6745.6375	6745.6317	0.0058	14	1	14	2	15	0	15	13	1	13	2	14	0	14
6745.6375	6745.6327	0.0048	14	1	14	2	15	2	17	13	1	13	2	14	2	16
6745.6375	6745.6268	0.0107	14	1	14	2	15	1	15	13	1	13	2	14	1	14
6745.6375	6745.6291	0.0084	14	1	14	1	15	2	17	13	1	13	1	14	2	16
6745.6375	6745.6355	0.0020	14	5	10	2	13	2	14	13	5	9	2	12	2	13
6745.6375	6745.6273	0.0102	14	1	14	2	15	2	13	13	1	13	2	14	2	12
6745.6375	6745.6321	0.0054	14	1	14	1	15	0	15	13	1	13	1	12	2	14
6745.6375	6745.6323	0.0052	14	1	14	1	13	2	15	13	1	13	1	14	0	14
6745.6750	6745.6553	0.0197	14	1	14	0	14	2	16	13	1	13	0	13	2	15
6745.6750	6745.6566	0.0184	14	1	14	1	15	2	16	13	1	13	1	13	2	15
6745.6750	6745.6691	0.0059	14	1	14	2	16	2	18	13	1	13	2	15	2	17
6745.6750	6745.6649	0.0101	14	1	14	2	16	1	16	13	1	13	2	15	1	15
6745.6750	6745.6587	0.0163	14	1	14	2	15	1	16	13	1	13	2	14	1	15
6745.6750	6745.6651	0.0099	14	1	14	2	16	0	16	13	1	13	2	15	2	15
6745.6750	6745.6704	0.0046	14	1	14	1	14	2	15	13	1	13	1	13	2	14
6745.6750	6745.6521	0.0229	14	1	14	1	15	1	14	13	1	13	1	14	1	13
6745.6750	6745.6628	0.0122	14	1	14	2	15	2	16	13	1	13	2	14	2	15
6745.6750	6745.6641	0.0109	14	1	14	1	15	2	15	13	1	13	1	14	2	14
6745.7000	6745.6801	0.0199	14	1	14	2	12	1	13	13	1	13	2	11	1	12
6745.7000	6745.6846	0.0154	14	1	14	2	15	2	15	13	1	13	2	14	2	14
6745.7000	6745.6800	0.0200	14	1	14	2	16	2	17	13	1	13	2	15	2	16
6745.7000	6745.6800	0.0200	14	1	14	1	14	1	13	13	1	13	1	13	1	12
6745.7000	6745.6804	0.0196	14	1	14	0	14	1	13	13	3	11	2	13	1	12
6745.7000	6745.6752	0.0248	14	1	14	2	16	2	15	13	1	13	2	15	2	14
6745.7000	6745.6863	0.0137	14	1	14	2	16	1	17	13	1	13	2	15	1	16
6745.7000	6745.6830	0.0170	14	1	14	2	16	1	15	13	1	13	2	15	1	14
6745.7000	6745.6832	0.0168	14	1	14	0	14	1	15	13	1	13	0	13	1	14
6745.7000	6745.6842	0.0158	14	1	14	1	14	1	15	13	1	13	1	13	1	14
6750.1704	6750.1816	-0.0112	14	0	14	0	14	2	12	13	0	13	2	13	2	11
6750.1704	6750.1743	-0.0039	14	0	14	2	14	2	16	13	0	13	2	13	2	15
6750.1704	6750.1774	-0.0070	14	0	14	0	14	1	14	13	0	13	2	13	1	13
6750.1704	6750.1780	-0.0076	14	0	14	2	14	0	14	13	0	13	2	13	0	13
6750.2125	6750.2032	0.0093	14	0	14	2	14	1	15	13	0	13	2	13	1	14
6750.2125	6750.2068	0.0057	14	0	14	2	13	2	15	13	0	13	2	12	2	14
6750.2370	6750.2432	-0.0062	14	2	12	2	13	2	14	13	0	13	2	12	2	13
6750.2370	6750.2279	0.0091	14	0	14	1	15	2	17	13	0	13	1	14	2	16
6750.2370	6750.2299	0.0071	14	0	14	1	15	0	15	13	0	13	1	12	2	14
6750.2370	6750.2410	-0.0040	14	0	14	1	13	2	15	13	0	13	1	14	0	14
6750.2370	6750.2099	0.0271	14	0	14	2	14	2	15	13	0	13	2	13	2	14
6750.2370	6750.2253	0.0117	14	0	14	2	15	0	15	13	0	13	2	14	0	14

6750.2370	6750.2291	0.0079	14	0	14	2	15	2	17	13	0	13	2	14	2	16
6750.2370	6750.2309	0.0061	14	0	14	1	15	1	15	13	0	13	1	14	1	14
6750.2370	6750.2188	0.0182	14	0	14	2	15	1	15	13	0	13	2	14	1	14
6750.2625	6750.2734	-0.0109	14	0	14	1	15	2	15	13	0	13	1	14	2	14
6750.2625	6750.2617	0.0008	14	0	14	2	15	1	16	13	0	13	2	14	1	15
6750.2625	6750.2759	-0.0134	14	0	14	2	16	0	16	13	2	11	2	14	2	15
6750.2625	6750.2485	0.0140	14	0	14	1	15	1	16	13	0	13	1	14	1	15
6750.2625	6750.2744	-0.0119	14	0	14	2	16	1	16	13	0	13	2	15	1	15
6750.2625	6750.2666	-0.0041	14	0	14	1	14	2	16	13	0	13	1	13	2	15
6750.2625	6750.2564	0.0061	14	0	14	1	15	2	16	13	0	13	1	14	2	15
6750.2625	6750.2654	-0.0029	14	0	14	0	14	2	16	13	0	13	0	13	2	15
6750.2625	6750.2696	-0.0071	14	0	14	2	15	2	16	13	0	13	2	14	2	15
6750.3000	6750.3001	-0.0001	14	0	14	2	16	1	15	13	0	13	2	15	1	14
6750.3000	6750.2971	0.0029	14	0	14	2	16	2	17	13	0	13	2	15	2	16
6750.3000	6750.2823	0.0177	14	0	14	2	16	2	18	13	0	13	2	15	2	17
6750.3000	6750.3021	-0.0021	14	0	14	1	14	1	15	13	0	13	1	13	1	14
6750.3000	6750.3010	-0.0010	14	0	14	0	14	1	15	13	0	13	0	13	1	14
6750.3000	6750.2887	0.0113	14	0	14	0	14	2	15	13	0	13	0	13	2	14
6750.3000	6750.2873	0.0127	14	0	14	2	16	2	15	13	0	13	2	14	2	14
6750.3000	6750.2941	0.0059	14	0	14	2	15	2	15	13	0	13	2	15	2	14
6750.3000	6750.3029	-0.0029	14	0	14	2	16	1	17	13	0	13	2	15	1	16
6750.3000	6750.2857	0.0143	14	0	14	1	14	2	15	13	0	13	1	13	2	14
6938.6490	6938.6599	-0.0109	14	1	13	2	13	2	12	13	1	12	2	13	2	11
6938.6490	6938.6414	0.0076	14	1	13	1	13	2	14	13	1	12	1	12	2	13
6938.6490	6938.6519	-0.0029	14	1	13	1	13	2	11	13	1	12	1	12	2	10
6938.6734	6938.6720	0.0014	14	1	13	1	13	2	15	13	1	12	1	12	2	14
6938.6734	6938.6717	0.0017	14	1	13	2	15	2	17	13	1	12	2	14	2	16
6938.6734	6938.6693	0.0041	14	1	13	1	13	1	13	13	1	12	1	12	1	12
6938.6734	6938.6747	-0.0013	14	1	13	1	15	2	17	13	1	12	1	14	2	16
6938.6734	6938.6643	0.0091	14	1	13	2	15	1	14	13	1	12	2	14	1	13
6938.6875	6938.6885	-0.0010	14	1	13	2	15	2	14	13	1	12	2	14	2	13
6938.6875	6938.6867	0.0008	14	1	13	1	15	1	15	13	1	12	1	14	1	14
6938.6875	6938.6884	-0.0009	14	1	13	2	13	2	15	13	1	12	2	13	2	14
6938.6875	6938.6787	0.0088	14	1	13	2	15	1	16	13	1	12	2	14	1	15
6938.6875	6938.6838	0.0037	14	1	13	1	15	0	15	13	1	12	1	14	0	14
6938.6875	6938.6967	-0.0092	14	1	13	1	13	2	12	13	1	12	1	12	2	11
6938.6875	6938.6973	-0.0098	14	1	13	1	13	1	14	13	1	12	1	12	1	13
6938.6875	6938.6893	-0.0018	14	1	13	2	15	2	15	13	3	10	2	14	2	14
6938.6875	6938.6960	-0.0085	14	1	13	1	13	1	12	13	1	12	1	12	1	11
6938.7375	6938.7162	0.0213	14	1	13	2	13	2	13	13	3	10	2	13	2	12
6938.7375	6938.7204	0.0171	14	1	13	1	15	1	16	13	1	12	1	14	1	15
6938.7375	6938.7214	0.0161	14	1	13	1	15	2	16	13	1	12	1	14	2	15
6938.7375	6938.7361	0.0014	14	1	13	1	15	2	14	13	1	12	1	14	2	13
6938.7375	6938.7203	0.0172	14	1	13	1	15	1	14	13	1	12	1	14	1	13
6938.7375	6938.7014	0.0361	14	1	13	2	13	0	13	13	1	12	2	12	0	12
6938.7375	6938.7123	0.0252	14	1	13	2	13	2	14	13	1	12	0	13	0	13
6938.7375	6938.7186	0.0189	14	1	13	2	13	2	11	13	1	12	2	12	2	10
6938.7375	6938.7179	0.0196	14	1	13	2	13	1	12	13	1	12	2	12	1	11
6938.7375	6938.7031	0.0344	14	1	13	2	13	1	13	13	1	12	2	12	1	12
6938.7500	6938.7397	0.0103	14	1	13	1	13	2	13	13	3	10	1	12	2	12
6938.7500	6938.7359	0.0141	14	1	13	1	13	2	14	13	1	12	1	13	2	13
6938.7500	6938.7289	0.0211	14	1	13	2	15	2	16	13	1	12	2	15	2	15
6938.8339	6938.8197	0.0142	14	1	13	2	16	1	16	13	1	12	2	15	1	15

6938.8339	6938.8279	0.0060	14	1	13	0	14	2	16	13	1	12	0	13	2	15
6938.8339	6938.8215	0.0124	14	1	13	2	16	2	18	13	1	12	2	15	2	17
6938.8339	6938.8367	-0.0028	14	1	13	2	12	2	14	13	3	10	2	11	2	13
6938.8339	6938.8292	0.0047	14	1	13	1	14	2	16	13	1	12	1	13	2	15
6938.8339	6938.8322	0.0017	14	1	13	2	16	2	17	13	1	12	2	15	2	16
6938.8339	6938.8208	0.0131	14	1	13	2	16	0	16	13	1	12	2	15	0	15
6938.8339	6938.8279	0.0060	14	1	13	0	14	1	14	13	1	12	0	13	1	13
6938.8339	6938.8281	0.0058	14	1	13	1	14	1	14	13	1	12	1	13	1	13
6938.8339	6938.8282	0.0057	14	1	13	1	14	0	14	13	1	12	1	13	0	13
6938.8625	6938.8672	-0.0047	14	1	13	2	16	1	17	13	1	12	2	15	1	16
6938.8625	6938.8652	-0.0027	14	1	13	0	14	1	13	13	1	12	0	13	1	12
6938.8625	6938.8637	-0.0012	14	1	13	2	12	2	13	13	1	12	2	12	2	12
6938.8625	6938.8608	0.0017	14	1	13	1	14	1	15	13	1	12	1	13	1	14
6938.8625	6938.8585	0.0040	14	1	13	1	14	1	13	13	1	12	1	13	1	12
6938.8625	6938.8633	-0.0008	14	1	13	2	12	1	13	13	1	12	2	11	1	12
6938.8625	6938.8544	0.0081	14	1	13	0	14	1	15	13	1	12	0	13	1	14
6938.8625	6938.8678	-0.0053	14	1	13	0	14	2	14	13	1	12	0	13	2	13
6938.8625	6938.8662	-0.0037	14	1	13	1	14	2	14	13	1	12	1	12	2	13
6966.1542	6966.1576	-0.0034	14	8	6	2	16	2	18	13	8	5	2	15	2	17
6966.1542	6966.1499	0.0043	14	8	7	0	14	2	16	13	8	6	0	13	2	15
6966.1542	6966.1507	0.0035	14	8	7	1	14	2	16	13	8	6	1	13	2	15
6966.1542	6966.1507	0.0035	14	8	6	1	14	2	16	13	8	5	1	13	2	15
6966.1542	6966.1499	0.0043	14	8	6	0	14	2	16	13	8	5	0	13	2	15
6966.1542	6966.1575	-0.0033	14	8	7	2	16	2	18	13	8	6	2	15	2	17
6966.1542	6966.1500	0.0042	14	8	6	2	16	0	16	13	8	5	2	15	0	15
6966.1542	6966.1500	0.0042	14	8	7	2	16	0	16	13	8	6	2	15	0	15
6966.1542	6966.1495	0.0047	14	8	7	2	16	1	16	13	8	6	2	15	1	15
6966.1542	6966.1495	0.0047	14	8	6	2	16	1	16	13	8	5	2	15	1	15
6966.2333	6966.2247	0.0086	14	8	6	2	16	1	17	13	8	5	2	15	1	16
6966.2333	6966.2339	-0.0006	14	8	7	2	16	2	17	13	8	6	2	15	2	16
6966.2333	6966.2192	0.0141	14	8	6	1	14	1	15	13	8	5	1	13	1	14
6966.2333	6966.2191	0.0142	14	8	7	1	14	1	15	13	8	6	1	13	1	14
6966.2333	6966.2247	0.0086	14	8	7	2	16	1	17	13	8	6	2	15	1	16
6966.2333	6966.2345	-0.0012	14	8	6	1	15	2	17	13	8	5	1	14	2	16
6966.2333	6966.2345	-0.0012	14	8	7	1	15	2	17	13	8	6	1	14	2	16
6966.2333	6966.2297	0.0036	14	8	6	1	15	1	15	13	8	5	1	14	1	14
6966.2333	6966.2297	0.0036	14	8	7	1	15	1	15	13	8	6	1	14	1	14
6966.2333	6966.2339	-0.0006	14	8	6	2	16	2	17	13	8	5	2	15	2	16
6966.2929	6966.3174	-0.0245	14	8	6	2	14	0	14	13	8	5	2	13	0	13
6966.2929	6966.3133	-0.0204	14	8	7	1	15	2	16	13	8	6	1	14	2	15
6966.2929	6966.3133	-0.0204	14	8	6	1	15	2	16	13	8	5	1	14	2	15
6966.2929	6966.2948	-0.0019	14	8	7	0	14	2	14	13	8	6	0	13	2	13
6966.2929	6966.3017	-0.0088	14	8	6	1	15	1	16	13	8	5	1	14	1	15
6966.2929	6966.3017	-0.0088	14	8	7	1	15	1	16	13	8	6	1	14	1	15
6966.2929	6966.3007	-0.0078	14	8	7	2	16	2	16	13	8	6	2	15	2	15
6966.2929	6966.3007	-0.0078	14	8	6	2	16	2	16	13	6	7	2	15	0	15
6966.2929	6966.2948	-0.0019	14	8	6	0	14	2	14	13	8	5	0	13	2	13
6966.2929	6966.3174	-0.0245	14	8	7	2	14	0	14	13	8	6	2	13	0	13
6966.9725	6966.9592	0.0133	14	3	12	2	16	2	18	13	3	11	2	15	2	17
6966.9725	6966.9781	-0.0056	14	3	12	2	16	1	17	13	3	11	2	15	1	16
6966.9725	6966.9624	0.0101	14	3	12	1	14	2	16	13	3	11	1	13	2	15
6966.9725	6966.9600	0.0125	14	3	12	2	16	0	16	13	3	11	2	15	0	15
6966.9725	6966.9628	0.0097	14	3	12	0	14	2	16	13	3	11	0	13	2	15

6966.9725	6966.9661	0.0064	14	3	12	1	15	2	16	13	3	11	1	14	2	15
6966.9725	6966.9606	0.0119	14	3	12	2	15	1	16	13	3	11	2	14	1	15
6966.9725	6966.9597	0.0128	14	3	12	2	16	1	16	13	3	11	2	15	1	15
6966.9725	6966.9674	0.0051	14	3	12	1	15	1	16	13	3	11	1	14	1	15
6966.9725	6966.9733	-0.0008	14	3	12	2	16	2	17	13	3	11	2	15	2	16
6968.5980	6968.5842	0.0138	14	7	8	2	16	0	16	13	7	7	2	15	0	15
6968.5980	6968.5852	0.0128	14	7	7	0	14	2	16	13	7	6	0	13	2	15
6968.5980	6968.5851	0.0129	14	7	7	2	16	1	16	13	7	6	2	15	1	15
6968.5980	6968.5850	0.0130	14	7	7	2	16	0	16	13	7	6	2	15	0	15
6968.5980	6968.5844	0.0136	14	7	8	2	16	1	16	13	7	7	2	15	1	15
6968.5980	6968.5874	0.0106	14	7	8	2	16	2	18	13	7	7	2	15	2	17
6968.5980	6968.5845	0.0135	14	7	8	1	14	2	16	13	7	7	1	13	2	15
6968.5980	6968.5845	0.0135	14	7	8	0	14	2	16	13	7	7	0	13	2	15
6968.5980	6968.5881	0.0099	14	7	7	2	16	2	18	13	7	6	2	15	2	17
6968.5980	6968.5853	0.0127	14	7	7	1	14	2	16	13	7	6	1	13	2	15
6968.6750	6968.6522	0.0228	14	7	8	1	15	2	17	13	7	7	1	14	2	16
6968.6750	6968.6408	0.0342	14	7	8	2	16	1	15	13	7	7	2	15	1	14
6968.6750	6968.6602	0.0148	14	7	8	2	15	2	17	13	7	7	2	14	2	16
6968.6750	6968.6609	0.0141	14	7	7	2	15	2	17	13	7	6	2	14	2	16
6968.6750	6968.6392	0.0358	14	7	7	2	16	1	17	13	7	6	2	15	1	16
6968.6750	6968.6529	0.0221	14	7	7	1	15	2	17	13	7	6	1	14	2	16
6968.6750	6968.6416	0.0334	14	7	7	2	16	1	15	13	7	6	2	15	1	14
6968.6750	6968.6384	0.0366	14	7	8	2	16	1	17	13	7	7	2	15	1	16
6968.6750	6968.6436	0.0314	14	7	7	2	16	2	17	13	7	6	2	15	2	16
6968.6750	6968.6428	0.0322	14	7	8	2	16	2	17	13	7	7	2	15	2	16
6968.7146	6968.6947	0.0199	14	7	7	2	16	2	16	13	7	6	2	15	2	15
6968.7146	6968.7104	0.0042	14	7	7	1	15	2	16	13	7	6	1	14	2	15
6968.7146	6968.6940	0.0206	14	7	8	2	16	2	16	13	7	7	2	15	2	15
6968.7146	6968.7256	-0.0110	14	7	7	2	14	2	16	13	7	6	2	13	2	15
6968.7146	6968.7096	0.0050	14	7	8	1	15	2	16	13	7	7	1	14	2	15
6968.7146	6968.7098	0.0048	14	7	7	2	15	1	16	13	7	6	2	14	1	15
6968.7146	6968.7248	-0.0102	14	7	8	2	14	2	16	13	7	7	2	13	2	15
6968.7146	6968.7041	0.0105	14	7	7	1	15	1	16	13	7	6	1	14	1	15
6968.7146	6968.7091	0.0055	14	7	8	2	15	1	16	13	7	7	2	14	1	15
6968.7146	6968.7034	0.0112	14	7	8	1	15	1	16	13	7	7	1	14	1	15
6968.7515	6968.7647	-0.0132	14	7	7	2	15	2	15	13	11	2	2	14	0	14
6968.7515	6968.7639	-0.0124	14	7	8	2	15	2	15	13	11	3	2	14	0	14
6968.7515	6968.7740	-0.0225	14	7	7	2	14	1	15	13	7	6	2	13	1	14
6968.7515	6968.7613	-0.0098	14	7	7	1	15	2	15	13	7	6	1	14	2	14
6968.7515	6968.7812	-0.0297	14	9	6	2	14	2	15	13	9	5	2	13	2	14
6968.7515	6968.7819	-0.0304	14	7	7	2	14	2	15	13	7	6	2	13	2	14
6968.7515	6968.7606	-0.0091	14	7	8	1	15	2	15	13	7	7	1	14	2	14
6968.7515	6968.7733	-0.0218	14	5	10	2	14	1	15	13	5	9	2	13	1	14
6972.3500	6972.3611	-0.0111	14	6	9	0	14	2	16	13	6	8	0	13	2	15
6972.3500	6972.3600	-0.0100	14	6	9	2	16	2	14	13	6	8	2	15	2	13
6972.3500	6972.3634	-0.0134	14	6	9	2	16	2	18	13	6	8	2	15	2	17
6972.3500	6972.3594	-0.0094	14	6	9	1	14	0	14	13	6	8	1	13	0	13
6972.3500	6972.3594	-0.0094	14	6	9	0	14	0	14	13	6	8	0	13	0	13
6972.3500	6972.3595	-0.0095	14	6	9	1	14	1	14	13	6	8	1	13	1	13
6972.3500	6972.3594	-0.0094	14	6	9	0	14	1	14	13	6	8	0	13	1	13
6972.3500	6972.3612	-0.0112	14	6	9	1	14	2	16	13	6	8	1	13	2	15
6972.3500	6972.3618	-0.0118	14	6	9	2	16	1	16	13	6	8	2	15	1	15
6972.3500	6972.3617	-0.0117	14	6	9	2	16	0	16	13	6	8	2	15	0	15

6972.3750	6972.3976	-0.0226	14	6	9	0	14	1	15	13	6	8	0	13	1	14
6972.3750	6972.4015	-0.0265	14	6	9	2	16	1	15	13	6	8	2	15	1	14
6972.3750	6972.3953	-0.0203	14	6	8	1	14	2	16	13	6	7	1	13	2	15
6972.3750	6972.3953	-0.0203	14	6	8	0	14	2	16	13	6	7	0	13	2	15
6972.3750	6972.3976	-0.0226	14	6	8	2	16	2	18	13	6	7	2	15	2	17
6972.3750	6972.3976	-0.0226	14	6	9	1	14	1	15	13	6	8	1	13	1	14
6972.3750	6972.4027	-0.0277	14	6	9	2	16	2	17	13	6	8	2	15	2	16
6972.3750	6972.3960	-0.0210	14	6	8	2	16	1	16	13	6	7	2	15	1	15
6972.3750	6972.3959	-0.0209	14	6	8	2	16	0	16	13	4	9	2	15	0	15
6972.3750	6972.4001	-0.0251	14	6	9	2	16	1	17	13	6	8	2	15	1	16
6972.4250	6972.4317	-0.0067	14	6	8	0	14	1	15	13	6	7	0	13	1	14
6972.4250	6972.4318	-0.0068	14	6	8	1	14	1	15	13	6	7	1	13	1	14
6972.4250	6972.4167	0.0083	14	6	9	1	15	0	15	13	6	8	1	14	0	14
6972.4250	6972.4395	-0.0145	14	6	9	2	16	2	16	13	6	8	2	15	2	15
6972.4250	6972.4167	0.0083	14	6	9	1	15	1	15	13	6	8	1	14	1	14
6972.4250	6972.4343	-0.0093	14	6	8	2	16	1	17	13	6	7	2	15	1	16
6972.4250	6972.4182	0.0068	14	6	9	1	15	2	17	13	6	8	1	14	2	16
6972.4250	6972.4369	-0.0119	14	6	8	2	16	2	17	13	6	7	2	15	2	16
6972.4250	6972.4235	0.0015	14	6	9	2	15	2	17	13	6	8	2	14	2	16
6972.4250	6972.4356	-0.0106	14	6	8	2	16	1	15	13	6	7	2	15	1	14
6972.4500	6972.4525	-0.0025	14	6	8	1	15	2	17	13	6	7	1	14	2	16
6972.4500	6972.4578	-0.0078	14	6	8	2	15	2	17	13	6	7	2	14	2	16
6972.4500	6972.4508	-0.0008	14	6	8	1	15	0	15	13	6	7	1	14	0	14
6972.4500	6972.4736	-0.0236	14	6	8	2	16	2	16	13	6	7	2	15	2	15
6972.4500	6972.4582	-0.0082	14	6	9	1	15	2	16	13	6	8	1	14	2	15
6972.4500	6972.4594	-0.0094	14	6	9	2	15	1	16	13	6	8	2	14	1	15
6972.4500	6972.4624	-0.0124	14	6	9	2	15	2	16	13	6	8	2	14	2	15
6972.4500	6972.4549	-0.0049	14	6	9	1	15	1	16	13	6	8	1	14	1	15
6972.5000	6972.4985	0.0015	14	4	11	2	15	2	15	13	4	10	2	14	2	14
6972.5000	6972.4784	0.0216	14	6	9	2	14	2	16	13	6	8	2	13	2	15
6972.5000	6972.4967	0.0033	14	6	8	2	15	2	16	13	6	7	2	14	2	15
6972.5000	6972.4949	0.0051	14	6	9	1	15	2	15	13	6	8	1	14	2	14
6972.5000	6972.4892	0.0108	14	6	8	1	15	1	16	13	6	7	1	14	1	15
6972.5000	6972.4925	0.0075	14	6	8	1	15	2	16	13	6	7	1	14	2	15
6972.5000	6972.4937	0.0063	14	6	8	2	15	1	16	13	6	7	2	14	1	15
6972.5000	6972.4950	0.0050	14	6	8	2	15	1	14	13	6	7	2	14	1	13
6972.5000	6972.4909	0.0091	14	6	8	1	15	1	14	13	6	7	1	14	1	13
6972.5000	6972.4914	0.0086	14	6	8	1	13	1	14	13	6	7	1	12	1	13
6972.5250	6972.5112	0.0138	14	4	10	2	14	1	14	13	6	7	2	13	1	13
6972.5250	6972.5111	0.0139	14	6	8	2	14	0	14	13	6	7	2	13	0	13
6972.5250	6972.5328	-0.0078	14	4	10	2	15	2	15	13	4	9	2	14	2	14
6972.5250	6972.5159	0.0091	14	6	9	2	14	1	13	13	6	8	2	13	1	12
6972.5250	6972.5182	0.0068	14	6	9	2	14	2	15	13	6	8	2	13	2	14
6972.5250	6972.5141	0.0109	14	6	9	2	14	1	15	13	6	8	2	13	1	14
6972.5250	6972.5130	0.0120	14	6	8	2	14	2	16	13	8	5	2	13	2	15
6972.5250	6972.5291	-0.0041	14	6	8	1	15	2	15	13	6	7	1	14	2	14
6978.3625	6978.3907	-0.0282	14	5	10	1	14	2	16	13	5	9	1	13	2	15
6978.3625	6978.3866	-0.0241	14	5	10	2	16	0	16	13	5	9	2	15	0	15
6978.3625	6978.4068	-0.0443	14	5	10	2	16	1	15	13	5	9	2	15	1	14
6978.3625	6978.3928	-0.0303	14	5	10	0	14	2	16	13	5	9	0	13	2	15
6978.3625	6978.4061	-0.0436	14	5	10	2	16	2	17	13	5	9	2	15	2	16
6978.3625	6978.4070	-0.0445	14	5	10	2	16	1	17	13	5	9	2	15	1	16
6978.3625	6978.4064	-0.0439	14	5	10	2	16	2	15	13	5	9	2	15	2	14

6978.3625	6978.3864	-0.0239	14	5	10	2	16	1	16	13	5	9	2	15	1	15
6978.3625	6978.3971	-0.0346	14	3	12	2	12	2	14	13	5	9	2	11	2	13
6978.3625	6978.3866	-0.0241	14	5	10	2	16	2	18	13	5	9	2	15	2	17
6978.3875	6978.4130	-0.0255	14	5	10	1	14	2	15	13	5	9	1	13	2	14
6978.3875	6978.4131	-0.0256	14	5	10	1	14	1	15	13	5	9	1	13	1	14
6978.3875	6978.4240	-0.0365	14	5	10	2	16	2	16	13	5	9	2	15	2	15
6978.3875	6978.4156	-0.0281	14	5	10	0	14	2	15	13	5	9	0	13	2	14
6978.3875	6978.4160	-0.0285	14	5	10	0	14	1	15	13	5	9	0	13	1	14
6978.3875	6978.4160	-0.0285	14	5	10	0	14	1	13	13	5	9	0	13	1	12
6978.4125	6978.4491	-0.0366	14	5	10	1	13	2	15	13	5	9	1	12	2	14
6978.4125	6978.4352	-0.0227	14	7	8	2	15	1	15	13	7	7	2	14	1	14
6978.4125	6978.4433	-0.0308	14	5	10	1	15	0	15	13	5	9	1	14	0	14
6978.4125	6978.4351	-0.0226	14	5	10	2	15	0	15	13	5	9	2	14	0	14
6978.4125	6978.4365	-0.0240	14	3	12	2	13	2	15	13	3	11	2	12	2	14
6978.4125	6978.4384	-0.0259	14	5	10	0	14	2	14	13	5	9	0	13	2	13
6978.4125	6978.4437	-0.0312	14	5	10	1	15	2	17	13	5	9	1	14	2	16
6978.4125	6978.4358	-0.0233	14	5	10	2	15	2	17	13	5	9	2	14	2	16
6978.4125	6978.4433	-0.0308	14	5	10	1	15	1	15	13	5	9	1	14	1	14
6979.3375	6979.3625	-0.0250	14	5	9	2	16	0	16	13	5	8	2	15	0	15
6979.3375	6979.3624	-0.0249	14	5	9	2	16	1	16	13	5	8	2	15	1	15
6979.3375	6979.3610	-0.0235	14	5	9	1	14	0	14	13	5	8	1	13	0	13
6979.3375	6979.3612	-0.0237	14	5	9	1	14	2	16	13	5	8	1	13	2	15
6979.3375	6979.3613	-0.0238	14	5	9	0	14	2	16	13	5	8	0	13	2	15
6979.3375	6979.3622	-0.0247	14	5	9	2	16	2	14	13	5	8	2	15	2	13
6979.3375	6979.3610	-0.0235	14	5	9	0	14	0	14	13	5	8	0	13	0	13
6979.3375	6979.3609	-0.0234	14	5	9	0	14	1	14	13	5	8	0	13	1	13
6979.3375	6979.3628	-0.0253	14	5	9	2	16	2	18	13	5	8	2	15	2	17
6979.3375	6979.3609	-0.0234	14	5	9	1	14	1	14	13	5	8	1	13	1	13
6979.3625	6979.3849	-0.0224	14	5	9	0	14	1	13	13	5	8	0	13	1	12
6979.3625	6979.3858	-0.0233	14	5	9	2	16	1	17	13	5	8	2	15	1	16
6979.3625	6979.3859	-0.0234	14	5	9	0	14	2	15	13	5	8	0	13	2	14
6979.3625	6979.3845	-0.0220	14	5	9	0	14	1	15	13	5	8	0	13	1	14
6979.3625	6979.3864	-0.0239	14	5	9	2	16	1	15	13	5	8	2	15	1	14
6979.3625	6979.3857	-0.0232	14	5	9	1	14	2	15	13	5	8	1	13	2	14
6979.3625	6979.3848	-0.0223	14	5	9	1	14	1	13	13	5	8	1	13	1	12
6979.3625	6979.3845	-0.0220	14	5	9	1	14	1	15	13	5	8	1	13	1	14
6979.3625	6979.3859	-0.0234	14	5	9	2	16	2	15	13	5	8	2	15	2	14
6979.3625	6979.3871	-0.0246	14	5	9	2	16	2	17	13	5	8	2	15	2	16
6979.4000	6979.4130	-0.0130	14	5	9	2	13	2	15	13	5	8	2	12	2	14
6979.4000	6979.4154	-0.0154	14	5	9	1	15	2	17	13	5	8	1	14	2	16
6979.4000	6979.4171	-0.0171	14	5	9	2	15	2	17	13	5	8	2	14	2	16
6979.4000	6979.4159	-0.0159	14	5	9	1	13	2	15	13	5	8	1	12	2	14
6979.4000	6979.4149	-0.0149	14	5	9	1	15	0	15	13	5	8	1	14	0	14
6979.4000	6979.4091	-0.0091	14	5	9	0	14	2	14	13	5	8	0	13	2	13
6979.4000	6979.4169	-0.0169	14	5	9	2	15	0	15	13	5	8	2	14	0	14
6979.4000	6979.4169	-0.0169	14	5	9	2	15	1	15	13	5	8	2	14	1	14
6979.4000	6979.4149	-0.0149	14	5	9	1	15	1	15	13	5	8	1	14	1	14
6979.4000	6979.4099	-0.0099	14	5	9	2	16	2	16	13	5	8	2	15	2	15
6979.4125	6979.4373	-0.0248	14	5	9	2	13	1	14	13	5	8	2	12	1	13
6979.4125	6979.4396	-0.0271	14	5	9	1	13	1	14	13	5	8	1	12	1	13
6979.4125	6979.4419	-0.0294	14	5	9	2	15	1	14	13	5	8	2	14	1	13
6979.4125	6979.4425	-0.0300	14	5	9	2	15	2	16	13	5	8	2	14	2	15
6979.4125	6979.4387	-0.0262	14	5	9	1	15	1	14	13	5	8	1	14	1	13

6979.4125	6979.4390	-0.0265	14	5	9	1	15	2	16	13	5	8	1	14	2	15
6979.4125	6979.4410	-0.0285	14	5	9	2	15	1	16	13	5	8	2	14	1	15
6979.4125	6979.4398	-0.0273	14	5	9	1	13	2	14	13	5	8	1	12	2	13
6979.4125	6979.4384	-0.0259	14	5	9	1	15	1	16	13	5	8	1	14	1	15
6979.4125	6979.4375	-0.0250	14	5	9	1	15	2	14	13	5	8	1	14	2	13
6979.4625	6979.4693	-0.0068	14	5	9	2	14	2	16	13	5	8	2	13	2	15
6979.4625	6979.4655	-0.0030	14	5	9	2	15	2	15	13	7	6	2	14	0	14
6979.4625	6979.4694	-0.0069	14	5	9	2	14	0	14	13	5	8	2	13	0	13
6979.4625	6979.4692	-0.0067	14	5	9	2	14	1	14	13	5	8	2	13	1	13
6979.4625	6979.4621	0.0004	14	5	9	1	15	2	15	13	5	8	1	14	2	14
6979.4625	6979.4622	0.0003	14	5	9	2	13	2	13	13	5	8	2	12	2	12
6979.4625	6979.4693	-0.0068	14	5	9	2	14	2	12	13	5	8	2	13	2	11
6979.4625	6979.4633	-0.0008	14	5	9	1	13	2	13	13	7	6	1	12	2	12
6979.4875	6979.4926	-0.0051	14	5	9	2	14	2	13	13	7	6	0	13	2	12
6979.4875	6979.4940	-0.0065	14	5	9	2	14	1	13	13	5	8	2	13	1	12
6979.4875	6979.4938	-0.0063	14	5	9	2	14	2	15	13	5	8	2	13	2	14
6979.4875	6979.4938	-0.0063	14	5	9	2	14	1	15	13	5	8	2	13	1	14
6984.3966	6984.4034	-0.0068	14	4	11	2	16	0	16	13	4	10	2	15	0	15
6984.3966	6984.4187	-0.0221	14	4	11	2	16	2	17	13	4	10	2	15	2	16
6984.3966	6984.4033	-0.0067	14	4	11	2	16	1	16	13	4	10	2	15	1	15
6984.3966	6984.4195	-0.0229	14	4	11	0	14	1	15	13	4	10	0	13	1	14
6984.3966	6984.4033	-0.0067	14	4	11	0	14	2	16	13	4	10	0	13	2	15
6984.3966	6984.4186	-0.0220	14	4	11	2	16	1	15	13	4	10	2	15	1	14
6984.3966	6984.4031	-0.0065	14	4	11	1	14	2	16	13	4	10	1	13	2	15
6984.3966	6984.4186	-0.0220	14	4	11	2	16	1	17	13	4	10	2	15	1	16
6984.3966	6984.4029	-0.0063	14	4	11	2	16	2	18	13	4	10	2	15	2	17
6984.3966	6984.4193	-0.0227	14	4	11	1	14	1	15	13	4	10	1	13	1	14
6984.4625	6984.4535	0.0090	14	4	11	2	15	1	16	13	4	10	2	14	1	15
6984.4625	6984.4545	0.0080	14	4	11	1	15	1	16	13	4	10	1	14	1	15
6984.4625	6984.4342	0.0283	14	4	11	2	16	2	16	13	4	10	2	15	2	15
6984.4625	6984.4390	0.0235	14	4	11	1	15	1	15	13	4	10	1	14	1	14
6984.4625	6984.4390	0.0235	14	4	11	1	15	0	15	13	4	10	1	14	0	14
6984.4625	6984.4537	0.0088	14	4	11	2	15	2	16	13	4	10	2	14	2	15
6984.4625	6984.4535	0.0090	14	4	11	1	15	2	16	13	4	10	1	14	2	15
6984.4625	6984.4388	0.0237	14	4	11	1	15	2	17	13	4	10	1	14	2	16
6984.4625	6984.4364	0.0261	14	4	11	2	15	2	17	13	4	10	2	14	2	16
6984.4625	6984.4370	0.0255	14	4	11	1	13	2	15	13	4	10	1	12	2	14
6999.4897	6999.4816	0.0081	14	4	10	0	14	2	16	13	4	9	0	13	2	15
6999.4897	6999.4881	0.0016	14	4	10	2	16	1	15	13	4	9	2	15	1	14
6999.4897	6999.4839	0.0058	14	4	10	2	16	2	18	13	4	9	2	15	2	17
6999.4897	6999.4886	0.0011	14	4	10	2	16	1	17	13	4	9	2	15	1	16
6999.4897	6999.4924	-0.0027	14	4	10	2	16	2	16	13	4	9	2	15	2	15
6999.4897	6999.4851	0.0046	14	4	10	2	16	0	16	13	2	11	2	15	0	15
6999.4897	6999.4879	0.0018	14	4	10	2	16	2	17	13	4	9	2	15	2	16
6999.4897	6999.4850	0.0047	14	4	10	2	16	1	16	13	4	9	2	15	1	15
6999.4897	6999.4815	0.0082	14	4	10	1	14	2	16	13	4	9	1	13	2	15
6999.4897	6999.4869	0.0028	14	4	10	0	14	1	15	13	4	9	0	13	1	14
6999.5719	6999.5626	0.0093	14	4	10	1	15	0	15	13	4	9	1	14	0	14
6999.5719	6999.5624	0.0095	14	4	10	1	13	2	15	13	4	9	1	12	2	14
6999.5719	6999.5690	0.0029	14	4	10	1	15	2	15	13	4	9	1	14	2	14
6999.5719	6999.5664	0.0055	14	4	10	1	15	1	16	13	4	9	1	14	1	15
6999.5719	6999.5618	0.0101	14	4	10	1	15	2	17	13	4	9	1	14	2	16
6999.5719	6999.5641	0.0078	14	4	10	1	15	2	16	13	4	9	1	14	2	15

6999.5719	6999.5706	0.0013	14	4	10	2	15	1	16	13	4	9	2	14	1	15
6999.5719	6999.5696	0.0023	14	4	10	2	15	2	16	13	6	7	2	14	2	15
6999.5719	6999.5625	0.0094	14	4	10	1	15	1	15	13	4	9	1	14	1	14
6999.5719	6999.5640	0.0079	14	4	10	2	15	2	17	13	4	9	2	14	2	16
6999.6528	6999.6474	0.0054	14	4	10	2	14	1	13	13	4	9	2	13	1	12
6999.6528	6999.6449	0.0079	14	4	10	2	14	2	15	13	2	11	2	13	2	14
6999.6528	6999.6413	0.0115	14	4	10	2	14	2	16	13	4	9	2	13	2	15
6999.6528	6999.6487	0.0041	14	4	10	2	14	1	15	13	4	9	2	13	1	14
6999.6528	6999.6483	0.0045	14	4	10	2	14	2	13	13	4	9	2	13	2	12
6999.6528	6999.6518	0.0010	14	2	12	2	14	2	14	13	2	11	2	13	2	13
6999.6528	6999.6446	0.0082	14	2	12	2	14	2	12	13	4	9	2	13	2	11
6999.6528	6999.6427	0.0101	14	4	10	2	14	1	14	13	4	9	2	13	1	13
6999.6528	6999.6430	0.0098	14	4	10	2	14	0	14	13	4	9	2	13	0	13
7062.7396	7062.7458	-0.0062	14	3	11	2	16	0	16	13	3	10	2	15	0	15
7062.7396	7062.7456	-0.0060	14	3	11	2	16	1	16	13	3	10	2	15	1	15
7062.7396	7062.7296	0.0100	14	3	11	1	14	1	15	13	3	10	1	13	1	14
7062.7396	7062.7300	0.0096	14	3	11	0	14	1	15	13	3	10	0	13	1	14
7062.7396	7062.7421	-0.0025	14	3	11	0	14	2	16	13	3	10	0	13	2	15
7062.7396	7062.7291	0.0105	14	3	11	2	16	1	15	13	3	10	2	15	1	14
7062.7396	7062.7437	-0.0041	14	3	11	2	16	2	18	13	3	10	2	15	2	17
7062.7396	7062.7277	0.0119	14	3	11	2	16	2	17	13	3	10	2	15	2	16
7062.7396	7062.7417	-0.0021	14	3	11	1	14	2	16	13	3	10	1	13	2	15
7062.7396	7062.7309	0.0087	14	3	11	2	16	1	17	13	3	10	2	15	1	16
7062.8516	7062.8526	-0.0010	14	3	11	2	15	2	17	13	3	10	2	14	2	16
7062.8516	7062.8378	0.0138	14	3	11	1	15	1	16	13	3	10	1	14	1	15
7062.8516	7062.8506	0.0010	14	3	11	1	15	2	17	13	3	10	1	14	2	16
7062.8516	7062.8527	-0.0011	14	3	11	1	15	0	15	13	3	10	1	14	0	14
7062.8516	7062.8338	0.0178	14	3	11	1	15	2	16	13	3	10	1	14	2	15
7062.8516	7062.8546	-0.0030	14	3	11	2	15	1	15	13	3	10	2	14	1	14
7062.8516	7062.8510	0.0006	14	3	11	1	13	2	15	13	3	10	1	12	2	14
7062.8516	7062.8525	-0.0009	14	3	11	1	15	1	15	13	3	10	1	14	1	14
7062.8516	7062.8414	0.0102	14	3	11	2	15	1	16	13	3	10	2	14	1	15
7062.8516	7062.8377	0.0139	14	3	11	2	15	2	16	13	3	10	2	14	2	15
7062.9716	7062.9619	0.0097	14	3	11	2	14	0	14	13	3	10	2	13	0	13
7062.9716	7062.9437	0.0279	14	3	11	2	14	2	15	13	3	10	2	13	2	14
7062.9716	7062.9595	0.0121	14	3	11	2	14	2	16	13	3	10	2	13	2	15
7062.9716	7062.9616	0.0100	14	3	11	2	14	1	14	13	3	10	2	13	1	13
7062.9716	7062.9462	0.0254	14	3	11	2	14	1	13	13	3	10	2	13	1	12
7062.9716	7062.9505	0.0211	14	3	11	2	14	2	13	13	3	10	2	13	2	12
7062.9716	7062.9486	0.0230	14	3	11	2	14	1	15	13	3	10	2	13	1	14
7062.9716	7062.9642	0.0074	14	3	11	2	14	2	12	13	3	10	2	13	2	11
7076.9000	7076.9355	-0.0355	14	2	12	2	13	2	15	13	2	11	2	12	2	14
7076.9000	7076.9213	-0.0213	14	2	12	2	15	1	16	13	2	11	2	14	1	15
7076.9000	7076.9219	-0.0219	14	2	12	2	15	2	16	13	4	9	2	14	2	15
7076.9000	7076.9419	-0.0419	14	2	12	2	15	2	15	13	2	11	2	14	2	14
7076.9000	7076.9101	-0.0101	14	2	12	1	13	2	15	13	2	11	1	12	2	14
7076.9000	7076.9215	-0.0215	14	2	12	1	15	1	15	13	2	11	1	14	1	14
7076.9000	7076.9218	-0.0218	14	2	12	1	15	2	17	13	2	11	1	14	2	16
7076.9000	7076.9216	-0.0216	14	2	12	1	15	0	15	13	2	11	1	14	0	14
7076.9000	7076.9419	-0.0419	14	2	12	1	15	2	16	13	2	11	1	14	2	15
7076.9000	7076.9414	-0.0414	14	2	12	1	15	1	16	13	2	11	1	14	1	15
7076.9824	7076.9948	-0.0124	14	2	12	0	14	1	15	13	2	11	0	13	1	14
7076.9824	7076.9952	-0.0128	14	2	12	0	14	2	15	13	2	11	0	13	2	14

7076.9824	7076.9935	-0.0111	14	2	12	1	14	1	15	13	2	11	1	13	1	14
7076.9824	7076.9938	-0.0114	14	2	12	1	14	2	15	13	2	11	1	13	2	14
7076.9824	7076.9750	0.0074	14	2	12	0	14	2	16	13	2	11	0	13	2	15
7076.9824	7076.9831	-0.0007	14	2	12	2	16	2	15	13	2	11	2	15	2	14
7076.9824	7076.9835	-0.0011	14	2	12	2	16	1	15	13	2	11	2	15	1	14
7076.9824	7076.9833	-0.0009	14	2	12	2	16	1	17	13	2	11	2	15	1	16
7076.9824	7076.9737	0.0087	14	2	12	1	14	2	16	13	2	11	1	13	2	15
7076.9824	7076.9838	-0.0014	14	2	12	2	16	2	17	13	2	11	2	15	2	16
7222.3750	7222.4022	-0.0272	15	1	15	2	15	0	15	14	1	14	2	14	0	14
7222.3750	7222.4057	-0.0307	15	1	15	2	15	2	13	14	1	14	2	14	2	12
7222.3750	7222.4016	-0.0266	15	1	15	2	15	1	15	14	1	14	2	14	1	14
7222.3750	7222.3986	-0.0236	15	1	15	2	15	2	17	14	1	14	2	14	2	16
7222.4250	7222.4324	-0.0074	15	1	15	1	16	2	18	14	1	14	1	15	2	17
7222.4250	7222.4353	-0.0103	15	1	15	1	16	0	16	14	1	14	1	15	0	15
7222.4250	7222.4286	-0.0036	15	1	15	2	16	1	16	14	1	14	2	15	1	15
7222.4250	7222.4335	-0.0085	15	1	15	2	16	0	16	14	1	14	2	15	0	15
7222.4250	7222.4360	-0.0110	15	1	15	2	16	2	18	14	1	14	2	15	2	17
7222.4250	7222.4479	-0.0229	15	1	15	1	16	1	17	14	1	14	1	15	1	16
7222.4250	7222.4354	-0.0104	15	1	15	1	16	1	16	14	1	14	1	15	1	15
7222.4250	7222.4258	-0.0008	15	1	15	2	15	2	16	14	1	14	2	14	2	15
7222.4250	7222.4256	-0.0006	15	3	13	2	16	1	16	14	3	12	2	15	1	15
7222.4250	7222.4352	-0.0102	15	1	15	1	14	2	16	14	1	14	1	13	2	15
7222.4875	7222.4660	0.0215	15	1	15	2	17	0	17	14	1	14	2	16	0	16
7222.4875	7222.4617	0.0258	15	1	15	2	16	2	17	14	1	14	2	15	2	16
7222.4875	7222.4634	0.0241	15	1	15	1	16	2	16	14	1	14	1	15	2	15
7222.4875	7222.4577	0.0298	15	1	15	0	15	2	17	14	1	14	0	14	2	16
7222.4875	7222.4586	0.0289	15	1	15	2	16	1	17	14	1	14	2	15	1	16
7222.4875	7222.4693	0.0182	15	1	15	2	17	2	19	14	1	14	2	16	2	18
7222.4875	7222.4662	0.0213	15	1	15	2	17	1	17	14	1	14	2	16	1	16
7222.4875	7222.4558	0.0317	15	1	15	1	16	2	17	14	1	14	1	15	2	16
7222.4875	7222.4527	0.0348	15	1	15	1	15	2	17	14	1	14	1	14	2	16
7222.5000	7222.4721	0.0279	15	1	15	0	15	2	16	14	1	14	0	14	2	15
7222.5000	7222.4952	0.0048	15	1	15	2	17	2	17	14	1	14	2	16	2	16
7222.5000	7222.4828	0.0172	15	1	15	1	15	1	16	14	1	14	1	14	1	15
7222.5000	7222.4754	0.0246	15	1	15	2	17	2	16	14	1	14	2	16	2	15
7222.5000	7222.4818	0.0182	15	1	15	2	17	1	16	14	1	14	2	16	1	15
7222.5000	7222.4820	0.0180	15	1	15	0	15	1	16	14	1	14	0	14	1	15
7222.5000	7222.4848	0.0152	15	1	15	2	17	1	18	14	1	14	2	16	1	17
7222.5000	7222.4791	0.0209	15	1	15	2	17	2	18	14	1	14	2	16	2	17
7225.5500	7225.5265	0.0235	15	0	15	2	15	2	13	14	0	14	2	14	2	12
7225.5500	7225.5226	0.0274	15	0	15	2	15	1	15	14	0	14	2	14	1	14
7225.5500	7225.5232	0.0268	15	0	15	2	15	0	15	14	0	14	2	14	0	14
7225.5500	7225.5198	0.0302	15	0	15	2	15	2	17	14	0	14	2	14	2	16
7225.5735	7225.5665	0.0070	15	0	15	2	16	2	18	14	0	14	2	15	2	17
7225.5735	7225.5556	0.0179	15	0	15	2	16	1	16	14	0	14	2	15	1	15
7225.5735	7225.5783	-0.0048	15	4	11	2	14	2	15	14	4	10	2	13	2	14
7225.5735	7225.5666	0.0069	15	0	15	1	14	2	16	14	0	14	1	13	2	15
7225.5735	7225.5618	0.0117	15	0	15	2	16	0	16	14	0	14	2	15	0	15
7225.5735	7225.5523	0.0212	15	2	13	2	16	1	16	14	2	12	2	15	1	15
7225.5735	7225.5668	0.0067	15	0	15	1	16	0	16	14	0	14	1	15	0	15
7225.5735	7225.5672	0.0063	15	0	15	1	16	1	16	14	0	14	1	15	1	15
7225.5735	7225.5537	0.0198	15	0	15	2	15	2	16	14	0	14	2	14	2	15
7225.5735	7225.5643	0.0092	15	0	15	1	16	2	18	14	0	14	1	15	2	17

7225.6000	7225.5821	0.0179	15	0	15	1	16	1	17	14	0	14	1	15	1	16
7225.6000	7225.5977	0.0023	15	0	15	0	15	2	17	14	0	14	0	14	2	16
7225.6000	7225.5929	0.0071	15	0	15	2	16	1	17	14	0	14	2	15	1	16
7225.6000	7225.5908	0.0092	15	0	15	1	14	1	15	14	0	14	1	13	1	14
7225.6000	7225.6058	-0.0058	15	0	15	2	17	0	17	14	0	14	2	16	0	16
7225.6000	7225.5909	0.0091	15	0	15	1	15	2	17	14	0	14	1	14	2	16
7225.6000	7225.6058	-0.0058	15	0	15	2	17	1	17	14	0	14	2	16	1	16
7225.6000	7225.5983	0.0017	15	0	15	2	16	2	17	14	0	14	2	15	2	16
7225.6000	7225.5943	0.0057	15	0	15	1	16	2	17	14	0	14	1	15	2	16
7225.6000	7225.6017	-0.0017	15	0	15	1	16	2	16	14	0	14	1	15	2	15
7225.6250	7225.6257	-0.0007	15	0	15	2	17	1	16	14	0	14	2	16	1	15
7225.6250	7225.6231	0.0019	15	0	15	2	17	2	18	14	0	14	2	16	2	17
7225.6250	7225.6106	0.0144	15	0	15	2	17	2	19	14	0	14	2	16	2	18
7225.6250	7225.6160	0.0090	15	0	15	0	15	2	16	14	0	14	0	14	2	15
7225.6250	7225.6141	0.0109	15	0	15	1	15	2	16	14	0	14	1	14	2	15
7225.6250	7225.6284	-0.0034	15	0	15	2	17	1	18	14	0	14	2	16	1	17
7225.6250	7225.6163	0.0087	15	0	15	2	17	2	16	14	0	14	2	16	2	15
7225.6250	7225.6264	-0.0014	15	0	15	0	15	1	16	14	0	14	0	14	1	15
7225.6250	7225.6272	-0.0022	15	0	15	1	15	1	16	14	0	14	1	14	1	15
7225.6250	7225.6202	0.0048	15	0	15	2	16	2	16	14	0	14	2	15	2	15
7225.6375	7225.6397	-0.0022	15	0	15	0	15	2	15	14	0	14	0	14	2	14
7225.6375	7225.6381	-0.0006	15	0	15	2	13	2	13	14	0	14	2	12	2	12
7225.6375	7225.6402	-0.0027	15	0	15	1	15	2	15	14	0	14	1	14	2	14
7225.6375	7225.6306	0.0069	15	0	15	2	13	2	12	14	0	14	2	12	2	11
7225.6375	7225.6414	-0.0039	15	0	15	2	17	2	17	14	0	14	2	16	2	16
7368.2125	7368.2235	-0.0110	15	2	14	2	15	2	13	14	2	13	2	14	2	12
7368.2125	7368.2202	-0.0077	15	2	14	2	15	2	17	14	2	13	2	14	2	16
7368.2125	7368.2215	-0.0090	15	2	14	2	15	1	15	14	2	13	2	14	1	14
7368.2125	7368.2218	-0.0093	15	2	14	2	15	0	15	14	2	13	2	14	0	14
7368.2500	7368.2343	0.0157	15	2	14	2	15	2	16	14	2	13	2	14	2	15
7368.2500	7368.2432	0.0068	15	2	14	2	15	1	14	14	2	13	2	14	1	13
7368.2500	7368.2490	0.0010	15	2	14	0	15	2	14	14	2	13	0	14	2	13
7368.2500	7368.2446	0.0054	15	2	14	2	15	1	16	14	2	13	2	14	1	15
7368.2875	7368.2861	0.0014	15	2	14	1	16	2	18	14	2	13	1	15	2	17
7368.2875	7368.2793	0.0082	15	2	14	2	16	2	18	14	2	13	2	15	2	17
7368.2875	7368.3018	-0.0143	15	2	14	2	16	2	17	14	2	13	2	15	2	16
7368.2875	7368.2825	0.0050	15	2	14	1	14	2	16	14	2	13	1	13	2	15
7368.2875	7368.2896	-0.0021	15	2	14	2	14	2	16	14	2	13	2	13	2	15
7368.2875	7368.2799	0.0076	15	2	14	2	16	1	16	14	2	13	2	15	1	15
7368.2875	7368.2802	0.0073	15	2	14	2	16	0	16	14	2	13	2	15	0	15
7368.2875	7368.3037	-0.0162	15	2	14	2	16	1	17	14	2	13	2	15	1	16
7368.2875	7368.2875	0.0000	15	2	14	1	16	1	16	14	2	13	1	15	1	15
7368.2875	7368.2878	-0.0003	15	2	14	1	16	0	16	14	2	13	1	15	0	15
7368.3250	7368.3106	0.0144	15	2	14	1	16	1	17	14	2	13	1	15	1	16
7368.3250	7368.3093	0.0157	15	2	14	1	16	1	15	14	2	13	1	15	1	14
7368.3250	7368.3086	0.0164	15	2	14	1	16	2	17	14	2	13	1	15	2	16
7368.3250	7368.3266	-0.0016	15	4	12	2	16	0	16	14	4	11	2	15	0	15
7368.3250	7368.3069	0.0181	15	2	14	1	14	1	15	14	2	13	1	13	1	14
7368.3250	7368.3048	0.0202	15	2	14	1	14	2	15	14	2	13	1	13	2	14
7368.3250	7368.3149	0.0101	15	2	14	2	14	1	15	14	2	13	2	13	1	14
7368.3500	7368.3461	0.0039	15	2	14	2	17	1	17	14	2	13	2	16	1	16
7368.3500	7368.3680	-0.0180	15	2	14	2	17	1	16	14	2	13	2	16	1	15
7368.3500	7368.3488	0.0012	15	2	14	0	15	2	17	14	2	13	0	14	2	16

7368.3500	7368.3700	-0.0200	15	2	14	1	15	2	16	14	2	13	1	14	2	15
7368.3500	7368.3464	0.0036	15	2	14	2	17	0	17	14	2	13	2	16	0	16
7368.3500	7368.3454	0.0046	15	2	14	2	17	2	19	14	2	13	2	16	2	18
7368.3500	7368.3335	0.0165	15	2	14	1	16	2	16	14	2	13	1	15	2	15
7368.3500	7368.3668	-0.0168	15	2	14	2	17	2	18	14	2	13	2	16	2	17
7368.3500	7368.3486	0.0014	15	2	14	1	15	2	17	14	2	13	1	14	2	16
7368.3500	7368.3699	-0.0199	15	2	14	2	17	1	18	14	2	13	2	16	1	17
7368.3750	7368.3775	-0.0025	15	2	14	2	13	1	14	14	2	13	2	12	1	13
7368.3750	7368.3740	0.0010	15	2	14	0	15	1	16	14	2	13	0	14	1	15
7368.3750	7368.3705	0.0045	15	2	14	2	17	2	16	14	2	13	2	16	2	15
7368.3750	7368.3830	-0.0080	15	2	14	1	15	1	16	14	2	13	1	14	1	15
7368.3750	7368.3702	0.0048	15	2	14	0	15	2	16	14	2	13	0	14	2	15
7368.3750	7368.3522	0.0228	15	2	14	2	14	2	15	14	2	13	2	13	2	14
7368.4000	7368.3916	0.0084	15	2	14	2	17	2	17	14	2	13	2	16	2	16
7368.4000	7368.3952	0.0048	15	2	14	1	15	2	15	14	2	13	1	14	2	14
7368.4000	7368.3994	0.0006	15	2	14	2	13	2	13	14	2	13	2	12	2	12
7368.4000	7368.3791	0.0209	15	2	14	2	13	2	12	14	2	13	2	12	2	11
7368.4000	7368.3955	0.0045	15	2	14	0	15	2	15	14	2	13	0	14	2	14
7464.9792	7464.9664	0.0128	15	8	8	2	17	2	19	14	8	7	2	16	2	18
7464.9792	7464.9628	0.0164	15	8	7	1	15	2	17	14	8	6	1	14	2	16
7464.9792	7464.9626	0.0166	15	8	7	0	15	2	17	14	8	6	0	14	2	16
7464.9792	7464.9664	0.0128	15	8	7	2	17	2	19	14	8	6	2	16	2	18
7464.9792	7464.9629	0.0163	15	8	8	2	17	0	17	14	8	7	2	16	0	16
7464.9792	7464.9630	0.0162	15	8	7	2	17	0	17	14	8	6	2	16	0	16
7464.9792	7464.9625	0.0167	15	8	8	0	15	2	17	14	8	7	0	14	2	16
7464.9792	7464.9632	0.0160	15	8	7	2	17	1	17	14	8	6	2	16	1	16
7464.9792	7464.9631	0.0161	15	8	8	2	17	1	17	14	8	7	2	16	1	16
7464.9792	7464.9628	0.0164	15	8	8	1	15	2	17	14	8	7	1	14	2	16
7465.0346	7465.0318	0.0028	15	8	7	1	16	2	18	14	8	6	1	15	2	17
7465.0346	7465.0268	0.0078	15	8	7	2	17	2	18	14	8	6	2	16	2	17
7465.0346	7465.0318	0.0028	15	8	8	1	16	2	18	14	8	7	1	15	2	17
7465.0346	7465.0213	0.0133	15	8	7	2	17	1	18	14	8	6	2	16	1	17
7465.0346	7465.0212	0.0134	15	8	8	2	17	1	18	14	8	7	2	16	1	17
7465.0346	7465.0287	0.0059	15	8	7	1	16	1	16	14	8	6	1	15	1	15
7465.0346	7465.0287	0.0059	15	8	8	1	16	1	16	14	8	7	1	15	1	15
7465.0346	7465.0268	0.0078	15	8	8	2	17	2	18	14	8	7	2	16	2	17
7465.0346	7465.0411	-0.0065	15	8	7	2	16	2	18	14	8	6	2	15	2	17
7465.0346	7465.0411	-0.0065	15	8	8	2	16	2	18	14	8	7	2	15	2	17
7465.0875	7465.0934	-0.0059	15	8	8	1	16	2	17	14	8	7	1	15	2	16
7465.0875	7465.0941	-0.0066	15	8	7	2	16	1	17	14	8	6	2	15	1	16
7465.0875	7465.0941	-0.0066	15	8	8	2	16	1	17	14	8	7	2	15	1	16
7465.0875	7465.0935	-0.0060	15	8	7	1	16	2	17	14	8	6	1	15	2	16
7465.0875	7465.1066	-0.0191	15	8	7	2	15	2	17	14	8	6	2	14	2	16
7465.0875	7465.1066	-0.0191	15	8	8	2	15	2	17	14	8	7	2	14	2	16
7465.0875	7465.0816	0.0059	15	8	7	2	17	2	17	14	8	6	2	16	2	16
7465.0875	7465.0816	0.0059	15	8	8	2	17	2	17	14	8	7	2	16	2	16
7465.0875	7465.0866	0.0009	15	8	7	1	16	1	17	14	8	6	1	15	1	16
7465.0875	7465.0866	0.0009	15	8	8	1	16	1	17	14	8	7	1	15	1	16
7465.1587	7465.1673	-0.0086	15	8	8	2	15	2	16	14	8	7	2	14	2	15
7465.1587	7465.1630	-0.0043	15	8	7	2	15	1	14	14	8	6	2	14	1	13
7465.1587	7465.1593	-0.0006	15	8	8	2	15	1	16	14	8	7	2	14	1	15
7465.1587	7465.1536	0.0051	15	6	9	2	16	2	16	14	12	2	2	15	0	15
7465.1587	7465.1593	-0.0006	15	6	9	2	15	1	16	14	6	8	2	14	1	15

7465.1587	7465.1536	0.0051	15	6	10	2	16	2	16	14	12	3	2	15	0	15
7465.1587	7465.1673	-0.0086	15	10	5	2	15	2	16	14	10	4	2	14	2	15
7465.1587	7465.1480	0.0107	15	8	8	1	16	2	16	14	8	7	1	15	2	15
7465.1587	7465.1630	-0.0043	15	8	8	2	15	1	14	14	8	7	2	14	1	13
7465.1587	7465.1480	0.0107	15	8	7	1	16	2	16	14	8	6	1	15	2	15
7467.9608	7467.9496	0.0112	15	7	9	1	15	2	17	14	7	8	1	14	2	16
7467.9608	7467.9514	0.0094	15	7	8	0	15	2	17	14	7	7	0	14	2	16
7467.9608	7467.9502	0.0106	15	7	9	2	17	0	17	14	7	8	2	16	0	16
7467.9608	7467.9503	0.0105	15	7	9	2	17	1	17	14	7	8	2	16	1	16
7467.9608	7467.9494	0.0114	15	7	9	0	15	2	17	14	7	8	0	14	2	16
7467.9608	7467.9523	0.0085	15	7	8	2	17	1	17	14	7	7	2	16	1	16
7467.9608	7467.9516	0.0092	15	7	8	1	15	2	17	14	7	7	1	14	2	16
7467.9608	7467.9544	0.0064	15	7	8	2	17	2	19	14	7	7	2	16	2	18
7467.9608	7467.9524	0.0084	15	7	9	2	17	2	19	14	7	8	2	16	2	18
7467.9608	7467.9522	0.0086	15	7	8	2	17	0	17	14	7	7	2	16	0	16
7468.0000	7468.0406	-0.0406	15	7	8	2	17	2	17	14	7	7	2	16	2	16
7468.0000	7468.0146	-0.0146	15	7	9	2	16	2	18	14	7	8	2	15	2	17
7468.0000	7467.9958	0.0042	15	7	8	2	17	1	18	14	7	7	2	16	1	17
7468.0000	7468.0386	-0.0386	15	7	9	2	17	2	17	14	7	8	2	16	2	16
7468.0000	7468.0096	-0.0096	15	7	8	1	16	2	18	14	7	7	1	15	2	17
7468.0000	7468.0076	-0.0076	15	7	9	1	16	2	18	14	7	8	1	15	2	17
7468.0000	7467.9990	0.0010	15	7	8	2	17	2	18	14	7	7	2	16	2	17
7468.0000	7467.9938	0.0062	15	7	9	2	17	1	18	14	7	8	2	16	1	17
7468.0000	7468.0166	-0.0166	15	7	8	2	16	2	18	14	7	7	2	15	2	17
7468.0000	7467.9970	0.0030	15	7	9	2	17	2	18	14	7	8	2	16	2	17
7468.0375	7468.0490	-0.0115	15	7	9	1	16	1	17	14	7	8	1	15	1	16
7468.0375	7468.0547	-0.0172	15	7	9	2	16	1	17	14	7	8	2	15	1	16
7468.0375	7468.0510	-0.0135	15	7	8	1	16	1	17	14	7	7	1	15	1	16
7468.0375	7468.0567	-0.0192	15	7	8	2	16	1	17	14	7	7	2	15	1	16
7468.0375	7468.0553	-0.0178	15	7	8	1	16	2	17	14	7	7	1	15	2	16
7468.0375	7468.0533	-0.0158	15	7	9	1	16	2	17	14	7	8	1	15	2	16
7468.0750	7468.0966	-0.0216	15	7	8	1	16	2	16	14	7	7	1	15	2	15
7468.0750	7468.0725	0.0025	15	7	8	2	15	2	17	14	7	7	2	14	2	16
7468.0750	7468.0946	-0.0196	15	7	9	1	16	2	16	14	7	8	1	15	2	15
7468.0750	7468.1101	-0.0351	15	3	13	2	15	1	16	14	5	10	2	14	1	15
7468.0750	7468.0704	0.0046	15	7	9	2	15	2	17	14	7	8	2	14	2	16
7468.0750	7468.1121	-0.0371	15	5	10	2	15	1	16	14	7	7	2	14	1	15
7468.0750	7468.1154	-0.0404	15	11	5	2	15	2	16	14	9	6	2	14	2	15
7468.0750	7468.1175	-0.0425	15	7	8	2	15	2	16	14	7	7	2	14	2	15
7468.0750	7468.1010	-0.0260	15	3	12	2	16	2	16	14	7	7	2	15	2	15
7468.0750	7468.0990	-0.0240	15	5	11	2	16	2	16	14	7	8	2	15	2	15
7479.7750	7479.8016	-0.0266	15	5	11	2	17	1	17	14	5	10	2	16	1	16
7479.7750	7479.8014	-0.0264	15	5	11	1	15	2	17	14	5	10	1	14	2	16
7479.7750	7479.8017	-0.0267	15	5	11	2	17	2	15	14	5	10	2	16	2	14
7479.7750	7479.8017	-0.0267	15	5	11	2	17	0	17	14	5	10	2	16	0	16
7479.7750	7479.8015	-0.0265	15	5	11	1	15	1	15	14	5	10	1	14	1	14
7479.7750	7479.8018	-0.0268	15	5	11	0	15	2	17	14	5	10	0	14	2	16
7479.7750	7479.8016	-0.0266	15	5	11	2	17	2	19	14	5	10	2	16	2	18
7479.7750	7479.8019	-0.0269	15	3	13	0	15	0	15	14	5	10	0	14	0	14
7479.7750	7479.8016	-0.0266	15	5	11	1	15	0	15	14	5	10	1	14	0	14
7479.7750	7479.8018	-0.0268	15	5	11	0	15	1	15	14	5	10	0	14	1	14
7479.8000	7479.8203	-0.0203	15	5	11	2	17	1	18	14	5	10	2	16	1	17
7479.8250	7479.8468	-0.0218	15	5	11	1	14	2	16	14	5	10	1	13	2	15

7479.8250	7479.8483	-0.0233	15	5	11	2	16	0	16	14	5	10	2	15	0	15
7479.8250	7479.8398	-0.0148	15	5	11	2	17	2	17	14	5	10	2	16	2	16
7479.8250	7479.8460	-0.0210	15	5	11	1	16	0	16	14	5	10	1	15	0	15
7479.8250	7479.8473	-0.0223	15	3	13	2	14	2	16	14	3	12	2	13	2	15
7479.8250	7479.8462	-0.0212	15	5	11	1	16	2	18	14	5	10	1	15	2	17
7479.8250	7479.8479	-0.0229	15	5	11	2	16	2	18	14	5	10	2	15	2	17
7479.8250	7479.8460	-0.0210	15	5	11	1	16	1	16	14	5	10	1	15	1	15
7479.8250	7479.8481	-0.0231	15	7	9	2	16	1	16	14	7	8	2	15	1	15
7479.8250	7479.8405	-0.0155	15	5	11	0	15	2	15	14	5	10	0	14	2	14
7479.8500	7479.8678	-0.0178	15	5	11	2	16	1	17	14	5	10	2	15	1	16
7479.8500	7479.8688	-0.0188	15	5	11	2	16	2	17	14	5	10	2	15	2	16
7479.8500	7479.8649	-0.0149	15	5	11	1	16	2	17	14	5	10	1	15	2	16
7479.8500	7479.8646	-0.0146	15	5	11	1	16	2	15	14	5	10	1	15	2	14
7479.8500	7479.8648	-0.0148	15	5	11	1	16	1	15	14	5	10	1	15	1	14
7479.8500	7479.8649	-0.0149	15	5	11	1	16	1	17	14	5	10	1	15	1	16
7479.8500	7479.8663	-0.0163	15	5	11	1	14	1	15	14	5	10	1	13	1	14
7479.8500	7479.8686	-0.0186	15	5	11	2	16	1	15	14	5	10	2	15	1	14
7479.8500	7479.8675	-0.0175	15	5	11	2	14	1	15	14	5	10	2	13	1	14
7479.8500	7479.8658	-0.0158	15	5	11	1	14	2	15	14	5	10	1	13	2	14
7479.8750	7479.8922	-0.0172	15	5	11	2	15	1	15	14	5	10	2	14	1	14
7479.8750	7479.8924	-0.0174	15	5	11	2	15	0	15	14	5	10	2	14	0	14
7479.8750	7479.8838	-0.0088	15	5	11	1	16	2	16	14	5	10	1	15	2	15
7479.8750	7479.8853	-0.0103	15	7	9	1	14	0	14	14	3	12	1	13	2	13
7479.8750	7479.8879	-0.0129	15	7	9	2	16	0	16	14	5	10	2	15	2	15
7479.8750	7479.8918	-0.0168	15	5	11	2	15	2	17	14	5	10	2	14	2	16
7479.8750	7479.8874	-0.0124	15	9	7	2	14	2	14	14	7	8	2	13	2	13
7479.8750	7479.8928	-0.0178	15	5	11	2	15	2	13	14	5	10	2	14	2	12
7479.8875	7479.9123	-0.0248	15	7	9	0	15	1	16	14	3	12	2	14	1	15
7479.8875	7479.9123	-0.0248	15	5	11	2	15	1	14	14	5	10	2	14	1	13
7479.8875	7479.9117	-0.0242	15	7	9	0	15	2	14	14	5	10	2	14	2	13
7479.8875	7479.9116	-0.0241	15	9	7	2	15	2	16	14	7	8	2	14	2	15
7481.6046	7481.6018	0.0028	15	5	10	1	15	2	17	14	5	9	1	14	2	16
7481.6046	7481.6023	0.0023	15	5	10	2	17	2	19	14	5	9	2	16	2	18
7481.6046	7481.6195	-0.0149	15	5	10	2	17	1	18	14	5	9	2	16	1	17
7481.6046	7481.6204	-0.0158	15	5	10	2	17	2	18	14	5	9	2	16	2	17
7481.6046	7481.6024	0.0022	15	5	10	2	17	1	17	14	5	9	2	16	1	16
7481.6046	7481.6025	0.0021	15	5	10	2	17	0	17	14	5	9	2	16	0	16
7481.6046	7481.6193	-0.0147	15	5	10	1	15	1	16	14	5	9	1	14	1	15
7481.6046	7481.6021	0.0025	15	5	10	0	15	2	17	14	5	9	0	14	2	16
7481.6046	7481.6199	-0.0153	15	5	10	2	17	1	16	14	5	9	2	16	1	15
7481.6046	7481.6196	-0.0150	15	5	10	0	15	1	16	14	5	9	0	14	1	15
7481.6607	7481.6554	0.0053	15	5	10	2	16	2	18	14	5	9	2	15	2	17
7481.6607	7481.6747	-0.0140	15	5	10	2	16	2	17	14	5	9	2	15	2	16
7481.6607	7481.6530	0.0077	15	5	10	1	16	1	16	14	5	9	1	15	1	15
7481.6607	7481.6530	0.0077	15	5	10	1	16	0	16	14	5	9	1	15	0	15
7481.6607	7481.6540	0.0067	15	5	10	1	14	2	16	14	5	9	1	13	2	15
7481.6607	7481.6373	0.0234	15	5	10	2	17	2	17	14	5	9	2	16	2	16
7481.6607	7481.6701	-0.0094	15	5	10	1	16	2	17	14	5	9	1	15	2	16
7481.6607	7481.6739	-0.0132	15	5	10	2	16	1	17	14	5	9	2	15	1	16
7481.6607	7481.6702	-0.0095	15	5	10	1	16	1	17	14	5	9	1	15	1	16
7481.6607	7481.6531	0.0076	15	5	10	1	16	2	18	14	5	9	1	15	2	17
7508.9349	7508.9256	0.0093	15	4	11	2	17	2	19	14	4	10	2	16	2	18
7508.9349	7508.9241	0.0108	15	4	11	2	17	1	18	14	4	10	2	16	1	17

7508.9349	7508.9228	0.0121	15	4	11	2	17	2	18	14	4	10	2	16	2	17
7508.9349	7508.9233	0.0116	15	4	11	2	17	1	16	14	4	10	2	16	1	15
7508.9349	7508.9222	0.0127	15	4	11	1	15	1	16	14	4	10	1	14	1	15
7508.9349	7508.9231	0.0118	15	4	11	1	15	2	17	14	4	10	1	14	2	16
7508.9349	7508.9232	0.0117	15	4	11	0	15	2	17	14	4	10	0	14	2	16
7508.9349	7508.9269	0.0080	15	4	11	2	17	1	17	14	4	10	2	16	1	16
7508.9349	7508.9270	0.0079	15	4	11	2	17	0	17	14	4	10	2	16	0	16
7508.9349	7508.9212	0.0137	15	4	11	2	17	2	17	14	4	10	2	16	2	16
7509.0213	7509.0104	0.0109	15	4	11	1	16	2	18	14	4	10	1	15	2	17
7509.0213	7509.0133	0.0080	15	4	11	2	16	2	18	14	4	10	2	15	2	17
7509.0213	7509.0117	0.0096	15	4	11	2	16	2	17	14	4	10	2	15	2	16
7509.0213	7509.0089	0.0124	15	4	11	1	16	1	17	14	4	10	1	15	1	16
7509.0213	7509.0061	0.0152	15	4	11	1	16	2	17	14	4	10	1	15	2	16
7509.0213	7509.0136	0.0077	15	4	11	2	16	1	17	14	4	10	2	15	1	16
7509.0213	7509.0048	0.0165	15	4	11	1	16	2	16	14	4	10	1	15	2	15
7509.0213	7509.0114	0.0099	15	4	11	1	16	1	16	14	4	10	1	15	1	15
7509.0213	7509.0115	0.0098	15	4	11	1	16	0	16	14	4	10	1	15	0	15
7509.0213	7509.0115	0.0098	15	4	11	1	14	2	16	14	4	10	1	13	2	15
7509.1144	7509.0977	0.0167	15	4	11	2	15	2	17	14	4	10	2	14	2	16
7509.1144	7509.0993	0.0151	15	4	11	2	15	1	15	14	4	10	2	14	1	14
7509.1144	7509.0986	0.0158	15	4	11	2	15	1	16	14	4	10	2	14	1	15
7509.1144	7509.0945	0.0199	15	4	11	2	15	2	16	14	4	10	2	14	2	15
7509.1144	7509.0995	0.0149	15	4	11	2	15	0	15	14	4	10	2	14	0	14
7509.1144	7509.1013	0.0131	15	4	11	2	15	2	13	14	2	12	2	14	2	12
7509.1144	7509.0950	0.0194	15	4	11	2	15	2	15	14	2	12	2	14	2	14
7509.1144	7509.0969	0.0175	15	4	11	2	15	1	14	14	4	10	2	14	1	13
7509.1144	7509.0988	0.0156	15	4	11	2	15	2	14	14	4	10	2	14	2	13
7572.0059	7571.9666	0.0393	15	2	13	2	15	2	13	14	2	12	2	14	2	12
7572.0059	7571.9929	0.0130	15	2	13	2	15	1	14	14	2	12	2	14	1	13
7572.0059	7571.9923	0.0136	15	4	11	0	15	2	14	14	4	10	0	14	2	13
7572.0059	7571.9676	0.0383	15	2	13	2	15	2	17	14	2	12	2	14	2	16
7572.0059	7571.9924	0.0135	15	0	15	2	15	1	16	14	0	14	2	14	1	15
7572.0059	7571.9671	0.0388	15	2	13	2	15	0	15	14	2	12	2	14	0	14
7572.0059	7571.9671	0.0388	15	2	13	2	15	1	15	14	2	12	2	14	1	14
7572.0059	7572.0185	-0.0126	15	0	15	2	15	2	15	14	0	14	2	14	2	14
7572.0625	7572.0528	0.0097	15	2	13	1	16	0	16	14	2	12	1	15	0	15
7572.0625	7572.0417	0.0208	15	2	13	1	14	2	16	14	2	12	1	13	2	15
7572.0625	7572.0532	0.0093	15	2	13	1	16	2	18	14	2	12	1	15	2	17
7572.0625	7572.0527	0.0098	15	2	13	1	16	1	16	14	2	12	1	15	1	15
7572.0625	7572.0325	0.0300	15	2	13	2	16	2	18	14	2	12	2	15	2	17
7572.0625	7572.0779	-0.0154	15	2	13	1	16	1	17	14	2	12	1	15	1	16
7572.0625	7572.0790	-0.0165	15	2	13	1	16	2	17	14	2	12	1	15	2	16
7572.0625	7572.0318	0.0307	15	4	11	2	16	1	16	14	4	10	2	15	1	15
7572.0625	7572.0583	0.0042	15	2	13	2	16	2	17	14	2	12	2	15	2	16
7572.0625	7572.0572	0.0053	15	2	13	2	16	1	17	14	2	12	2	15	1	16
7572.1351	7572.1442	-0.0091	15	2	13	2	17	1	16	14	2	12	2	16	1	15
7572.1351	7572.1541	-0.0190	15	2	13	1	15	1	16	14	2	12	1	14	1	15
7572.1351	7572.1694	-0.0343	15	2	13	2	17	2	17	14	2	12	2	16	2	16
7572.1351	7572.1306	0.0045	15	2	13	0	15	2	17	14	2	12	0	14	2	16
7572.1351	7572.1447	-0.0096	15	2	13	2	17	2	18	14	2	12	2	16	2	17
7572.1351	7572.1185	0.0166	15	2	13	2	17	1	17	14	2	12	2	16	1	16
7572.1351	7572.1191	0.0160	15	2	13	2	17	2	19	14	2	12	2	16	2	18
7572.1351	7572.1438	-0.0087	15	2	13	2	17	1	18	14	2	12	2	16	1	17

7572.1351	7572.1186	0.0165	15	2	13	2	17	0	17	14	2	12	2	16	0	16
7572.1351	7572.1294	0.0057	15	2	13	1	15	2	17	14	2	12	1	14	2	16
7579.8601	7579.8521	0.0080	15	3	12	2	17	1	18	14	3	11	2	16	1	17
7579.8601	7579.8494	0.0107	15	3	12	2	17	2	18	14	3	11	2	16	2	17
7579.8601	7579.8628	-0.0027	15	3	12	2	17	2	19	14	3	11	2	16	2	18
7579.8601	7579.8624	-0.0023	15	3	12	1	15	2	17	14	3	11	1	14	2	16
7579.8601	7579.8628	-0.0027	15	3	12	0	15	2	17	14	3	11	0	14	2	16
7579.8601	7579.8643	-0.0042	15	3	12	2	17	1	17	14	3	11	2	16	1	16
7579.8601	7579.8645	-0.0044	15	3	12	2	17	0	17	14	3	11	2	16	0	16
7579.8601	7579.8521	0.0080	15	3	12	1	15	1	16	14	3	11	1	14	1	15
7579.8601	7579.8387	0.0214	15	3	12	2	17	2	17	14	3	11	2	16	2	16
7579.8601	7579.8506	0.0095	15	3	12	2	17	1	16	14	3	11	2	16	1	15
7579.9523	7579.9508	0.0015	15	3	12	2	16	2	18	14	3	11	2	15	2	17
7579.9523	7579.9513	0.0010	15	3	12	1	16	2	18	14	3	11	1	15	2	17
7579.9523	7579.9268	0.0255	15	3	12	1	16	2	16	14	3	11	1	15	2	15
7579.9523	7579.9412	0.0111	15	3	12	2	16	1	17	14	3	11	2	15	1	16
7579.9523	7579.9382	0.0141	15	3	12	2	16	2	17	14	3	11	2	15	2	16
7579.9523	7579.9529	-0.0006	15	3	12	1	16	1	16	14	3	11	1	15	1	15
7579.9523	7579.9530	-0.0007	15	3	12	1	16	0	16	14	3	11	1	15	0	15
7579.9523	7579.9505	0.0018	15	3	12	1	14	2	16	14	3	11	1	13	2	15
7579.9523	7579.9406	0.0117	15	3	12	1	16	1	17	14	3	11	1	15	1	16
7579.9523	7579.9373	0.0150	15	3	12	1	16	2	17	14	3	11	1	15	2	16
7580.0375	7580.0317	0.0058	15	3	12	2	15	2	14	14	3	11	2	14	2	13
7580.0375	7580.0432	-0.0057	15	3	12	2	15	2	13	14	3	11	2	14	2	12
7580.0375	7580.0169	0.0206	15	3	12	2	15	2	15	14	3	11	2	14	2	14
7580.0375	7580.0281	0.0094	15	3	12	2	15	1	14	14	3	11	2	14	1	13
7580.0375	7580.0394	-0.0019	15	3	12	2	15	2	17	14	3	11	2	14	2	16
7580.0375	7580.0300	0.0075	15	3	12	2	15	1	16	14	3	11	2	14	1	15
7580.0375	7580.0262	0.0113	15	3	12	2	15	2	16	14	3	11	2	14	2	15
7580.0375	7580.0413	-0.0038	15	3	12	2	15	0	15	14	3	11	2	14	0	14
7580.0375	7580.0411	-0.0036	15	3	12	2	15	1	15	14	3	11	2	14	1	14
7701.1092	7701.1018	0.0074	16	0	16	2	16	0	16	15	0	15	2	15	0	15
7701.1092	7701.1189	-0.0097	16	0	16	2	16	1	15	15	0	15	2	15	1	14
7701.1092	7701.1048	0.0044	16	0	16	2	16	2	14	15	0	15	2	15	2	13
7701.1092	7701.0987	0.0105	16	0	16	2	16	2	18	15	0	15	2	15	2	17
7701.1092	7701.1013	0.0079	16	0	16	2	16	1	16	15	0	15	2	15	1	15
7701.1317	7701.1363	-0.0046	16	0	16	1	17	2	19	15	0	15	1	16	2	18
7701.1317	7701.1390	-0.0073	16	0	16	2	17	2	19	15	0	15	2	16	2	18
7701.1317	7701.1389	-0.0072	16	0	16	1	17	0	17	15	0	15	1	16	0	16
7701.1317	7701.1389	-0.0072	16	0	16	1	17	1	17	15	0	15	1	16	1	16
7701.1317	7701.1384	-0.0067	16	0	16	1	15	2	17	15	0	15	1	14	2	16
7701.1317	7701.1288	0.0029	16	2	14	2	17	1	17	15	2	13	2	16	1	16
7701.1317	7701.1220	0.0097	16	2	14	2	16	2	17	15	2	13	2	15	2	16
7701.1317	7701.1284	0.0033	16	0	16	2	16	2	17	15	0	15	2	15	2	16
7701.1623	7701.1731	-0.0108	16	0	16	2	18	1	18	15	0	15	2	17	1	17
7701.1623	7701.1762	-0.0139	16	0	16	2	18	2	20	15	0	15	2	17	2	19
7701.1623	7701.1609	0.0014	16	0	16	1	16	2	18	15	0	15	1	15	2	17
7701.1623	7701.1518	0.0105	16	0	16	1	17	1	18	15	0	15	1	16	1	17
7701.1623	7701.1678	-0.0055	16	0	16	1	17	2	17	15	0	15	1	16	2	16
7701.1623	7701.1727	-0.0104	16	0	16	2	18	0	18	15	0	15	2	17	0	17
7701.1623	7701.1598	0.0025	16	0	16	1	17	2	18	15	0	15	1	16	2	17
7701.1623	7701.1611	0.0012	16	0	16	2	17	1	18	15	0	15	2	16	1	17
7701.1623	7701.1645	-0.0022	16	0	16	2	17	2	18	15	0	15	2	16	2	17

7701.1623	7701.1659	-0.0036	16	0	16	0	16	2	18	15	0	15	0	15	2	17
7701.1785	7701.2028	-0.0243	16	0	16	2	18	2	18	15	0	15	2	17	2	17
7701.1785	7701.1918	-0.0133	16	0	16	2	18	1	19	15	0	15	2	17	1	18
7701.1785	7701.1897	-0.0112	16	0	16	0	16	1	17	15	0	15	0	15	1	16
7701.1785	7701.1823	-0.0038	16	0	16	2	18	2	17	15	0	15	2	17	2	16
7701.1785	7701.1903	-0.0118	16	0	16	1	16	1	17	15	0	15	1	15	1	16
7701.1785	7701.1891	-0.0106	16	0	16	2	18	1	17	15	0	15	2	17	1	16
7701.1785	7701.1868	-0.0083	16	0	16	2	18	2	19	15	0	15	2	17	2	18
7849.2475	7849.2364	0.0111	16	2	15	2	16	0	16	15	2	14	2	15	0	15
7849.2475	7849.2349	0.0126	16	2	15	2	16	2	18	15	2	14	2	15	2	17
7849.2475	7849.2361	0.0114	16	2	15	2	16	1	16	15	2	14	2	15	1	15
7849.2475	7849.2378	0.0097	16	2	15	2	16	2	14	15	2	14	2	15	2	13
7849.2750	7849.2573	0.0177	16	2	15	2	16	1	15	15	2	14	2	15	1	14
7849.2750	7849.2623	0.0127	16	2	15	0	16	2	15	15	2	14	0	15	2	14
7849.2750	7849.2585	0.0165	16	2	15	2	16	1	17	15	2	14	2	15	1	16
7849.2750	7849.2578	0.0172	16	2	15	2	16	2	17	15	2	14	2	15	2	16
7849.3000	7849.3007	-0.0007	16	2	15	1	17	2	19	15	2	14	1	16	2	18
7849.3000	7849.2953	0.0047	16	2	15	2	17	0	17	15	2	14	2	16	0	16
7849.3000	7849.2974	0.0026	16	2	15	1	15	2	17	15	2	14	1	14	2	16
7849.3000	7849.3019	-0.0019	16	2	15	1	17	1	17	15	2	14	1	16	1	16
7849.3000	7849.3022	-0.0022	16	2	15	1	17	0	17	15	2	14	1	16	0	16
7849.3000	7849.2950	0.0050	16	2	15	2	17	1	17	15	2	14	2	16	1	16
7849.3000	7849.2945	0.0055	16	2	15	2	17	2	19	15	2	14	2	16	2	18
7849.3366	7849.3210	0.0156	16	2	15	1	15	1	16	15	2	14	1	14	1	15
7849.3366	7849.3230	0.0136	16	2	15	1	17	1	16	15	2	14	1	16	1	15
7849.3366	7849.3280	0.0086	16	2	15	2	15	1	16	15	2	14	2	14	1	15
7849.3366	7849.3404	-0.0038	16	4	13	2	17	0	17	15	4	12	2	16	0	16
7849.3366	7849.3164	0.0202	16	2	15	2	17	2	18	15	2	14	2	16	2	17
7849.3366	7849.3242	0.0124	16	2	15	1	17	1	18	15	2	14	1	16	1	17
7849.3366	7849.3181	0.0185	16	2	15	2	17	1	18	15	2	14	2	16	1	17
7849.3366	7849.3224	0.0142	16	2	15	1	17	2	18	15	2	14	1	16	2	17
7849.3366	7849.3035	0.0331	16	2	15	2	15	2	17	15	2	14	2	14	2	16
7849.3366	7849.3189	0.0177	16	2	15	1	15	2	16	15	2	14	1	14	2	15
7849.3625	7849.3613	0.0012	16	2	15	2	18	0	18	15	2	14	2	17	0	17
7849.3625	7849.3462	0.0163	16	2	15	1	17	2	17	15	2	14	1	16	2	16
7849.3625	7849.3840	-0.0215	16	2	15	1	16	2	17	15	2	14	1	15	2	16
7849.3625	7849.3634	-0.0009	16	2	15	0	16	2	18	15	2	14	0	15	2	17
7849.3625	7849.3611	0.0014	16	2	15	2	18	1	18	15	2	14	2	17	1	17
7849.3625	7849.3823	-0.0198	16	2	15	2	18	1	17	15	2	14	2	17	1	16
7849.3625	7849.3605	0.0020	16	2	15	2	18	2	20	15	2	14	2	17	2	19
7849.3625	7849.3840	-0.0215	16	2	15	2	18	1	19	15	2	14	2	17	1	18
7849.3625	7849.3813	-0.0188	16	2	15	2	18	2	19	15	2	14	2	17	2	18
7849.3625	7849.3633	-0.0008	16	2	15	1	16	2	18	15	2	14	1	15	2	17
7849.3875	7849.3845	0.0030	16	2	15	2	18	2	17	15	2	14	2	17	2	16
7849.3875	7849.3663	0.0212	16	2	15	2	15	2	16	15	2	14	2	14	2	15
7849.3875	7849.3873	0.0002	16	2	15	0	16	1	17	15	2	14	0	15	1	16
7849.3875	7849.3842	0.0033	16	2	15	0	16	2	17	15	2	14	0	15	2	16
7849.3875	7849.3872	0.0003	16	2	15	1	16	1	17	15	2	14	1	15	1	16
7849.3875	7849.4050	-0.0175	16	2	15	2	18	2	18	15	2	14	2	17	2	17
7883.4463	7883.4817	-0.0354	16	1	15	2	16	2	14	15	1	14	2	15	2	13
7883.4463	7883.4785	-0.0322	16	1	15	2	16	1	16	15	1	14	2	15	1	15
7883.4463	7883.4778	-0.0315	16	1	15	2	16	2	18	15	1	14	2	15	2	17
7883.4463	7883.4786	-0.0323	16	1	15	2	16	0	16	15	1	14	2	15	0	15

7883.5910	7883.6087	-0.0177	16	1	15	1	15	1	15	15	1	14	1	14	1	14
7883.5910	7883.6018	-0.0108	16	1	15	2	17	0	17	15	1	14	2	16	0	16
7883.5910	7883.6170	-0.0260	16	1	15	1	17	2	19	15	1	14	1	16	2	18
7883.5910	7883.6266	-0.0356	16	1	15	2	15	2	17	15	1	14	2	14	2	16
7883.5910	7883.6017	-0.0107	16	1	15	2	17	1	17	15	1	14	2	16	1	16
7883.5910	7883.6089	-0.0179	16	1	15	1	15	0	15	15	1	14	1	14	0	14
7883.5910	7883.6028	-0.0118	16	1	15	2	17	2	19	15	1	14	2	16	2	18
7883.5910	7883.6095	-0.0185	16	1	15	1	15	2	17	15	1	14	1	14	2	16
7883.5910	7883.6176	-0.0266	16	1	15	1	17	1	17	15	1	14	1	16	1	16
7883.5910	7883.6178	-0.0268	16	1	15	1	17	0	17	15	1	14	1	16	0	16
7883.6437	7883.6381	0.0056	16	1	15	2	17	1	16	15	1	14	2	16	1	15
7883.6437	7883.6583	-0.0146	16	1	15	1	17	2	16	15	1	14	1	16	2	15
7883.6437	7883.6647	-0.0210	16	1	15	2	15	1	16	15	1	14	2	14	1	15
7883.6437	7883.6492	-0.0055	16	1	15	1	15	2	16	15	1	14	1	14	2	15
7883.6437	7883.6569	-0.0132	16	1	15	1	17	2	18	15	1	14	1	16	2	17
7883.6437	7883.6560	-0.0123	16	1	15	1	17	1	18	15	1	14	1	16	1	17
7883.6437	7883.6399	0.0038	16	1	15	2	17	1	18	15	1	14	2	16	1	17
7883.6437	7883.6563	-0.0126	16	1	15	1	17	1	16	15	1	14	1	16	1	15
7883.6437	7883.6415	0.0022	16	1	15	2	17	2	18	15	1	14	2	16	2	17
7883.6437	7883.6474	-0.0037	16	1	15	1	15	1	16	15	1	14	1	14	1	15
7883.7366	7883.7501	-0.0135	16	1	15	1	16	2	18	15	1	14	1	15	2	17
7883.7366	7883.7425	-0.0059	16	1	15	2	18	2	20	15	1	14	2	17	2	19
7883.7366	7883.7507	-0.0141	16	1	15	0	16	0	16	15	1	14	0	15	0	15
7883.7366	7883.7418	-0.0052	16	1	15	2	18	0	18	15	1	14	2	17	0	17
7883.7366	7883.7416	-0.0050	16	1	15	2	18	1	18	15	1	14	2	17	1	17
7883.7366	7883.7506	-0.0140	16	1	15	0	16	2	18	15	1	14	0	15	2	17
7883.7366	7883.7587	-0.0221	16	1	15	2	15	2	16	15	1	14	2	14	2	15
7883.7366	7883.7499	-0.0133	16	1	15	1	16	1	16	15	1	14	1	15	1	15
7883.7366	7883.7501	-0.0135	16	1	15	1	16	0	16	15	1	14	1	15	0	15
7883.7366	7883.7505	-0.0139	16	1	15	0	16	1	16	15	1	14	0	15	1	15
7883.7645	7883.7892	-0.0247	16	1	15	1	16	1	17	15	1	14	1	15	1	16
7883.7645	7883.7789	-0.0144	16	1	15	2	18	2	19	15	1	14	2	17	2	18
7883.7645	7883.7870	-0.0225	16	1	15	1	16	2	17	15	1	14	1	15	2	16
7883.7645	7883.7897	-0.0252	16	1	15	0	16	1	17	15	1	14	0	15	1	16
7883.7645	7883.7790	-0.0145	16	1	15	2	18	1	17	15	1	14	2	17	1	16
7883.7645	7883.7815	-0.0170	16	1	15	2	18	1	19	15	1	14	2	17	1	18
7883.7645	7883.7890	-0.0245	16	1	15	1	16	1	15	15	1	14	1	15	1	14
7883.7645	7883.7876	-0.0231	16	1	15	0	16	2	17	15	1	14	0	15	2	16
7883.7645	7883.7786	-0.0141	16	1	15	2	18	2	17	15	1	14	2	17	2	16
7883.7645	7883.7979	-0.0334	16	1	15	2	14	1	15	15	1	14	2	13	1	14
7883.8154	7883.8355	-0.0201	16	1	15	2	14	2	14	15	1	14	2	13	2	13
7883.8154	7883.8267	-0.0113	16	1	15	0	16	2	16	15	1	14	0	15	2	15
7883.8154	7883.8261	-0.0107	16	1	15	1	16	2	16	15	1	14	1	15	2	15
7883.8154	7883.8178	-0.0024	16	1	15	2	18	2	18	15	1	14	2	17	2	17
7952.0979	7952.0613	0.0366	16	1	16	2	16	2	16	15	3	13	2	15	2	15
7952.0979	7952.0523	0.0456	16	3	14	2	17	1	16	15	3	13	2	15	1	15
7952.0979	7952.0575	0.0404	16	5	12	2	17	2	17	15	7	9	2	15	2	16
7952.0979	7952.0589	0.0390	16	3	14	2	17	2	18	15	3	13	2	16	2	17
7952.0979	7952.0537	0.0442	16	3	14	2	16	1	16	15	3	13	2	16	1	15
7952.0979	7952.0621	0.0358	16	5	12	2	16	2	15	15	3	13	2	15	2	14
7952.0979	7952.0609	0.0370	16	3	14	1	17	2	19	15	3	13	1	16	2	18
7952.1084	7952.0676	0.0408	16	5	12	2	17	1	17	15	5	11	2	16	1	16
7952.1084	7952.0690	0.0394	16	5	12	2	16	2	17	15	3	13	2	16	0	16

7952.1084	7952.0778	0.0306	16	3	14	1	15	2	16	15	3	13	1	14	2	15
7952.1084	7952.0773	0.0311	16	3	14	1	17	1	18	15	3	13	1	16	1	17
7952.1084	7952.0719	0.0365	16	3	14	1	16	2	17	15	3	13	1	14	2	16
7952.1084	7952.0730	0.0354	16	3	14	2	17	2	17	15	3	13	2	16	2	16
7952.1084	7952.0704	0.0380	16	3	14	2	17	1	18	15	3	13	2	16	1	17
7952.1084	7952.0754	0.0330	16	3	14	2	15	1	16	15	3	13	2	14	1	15
7952.1084	7952.0682	0.0402	16	3	14	2	17	2	19	15	3	13	2	16	2	18
7952.1084	7952.0760	0.0324	16	3	14	1	17	2	18	15	3	13	1	16	2	17
7952.1250	7952.0862	0.0388	16	3	14	2	18	2	20	15	3	13	2	17	2	19
7952.1250	7952.0942	0.0308	16	3	14	2	18	1	17	15	3	13	2	17	1	16
7952.1250	7952.0866	0.0384	16	3	14	2	18	2	19	15	3	13	2	17	2	18
7952.1250	7952.0904	0.0346	16	3	14	1	16	0	16	15	3	13	1	15	0	15
7952.1250	7952.0906	0.0344	16	3	14	0	16	1	16	15	3	13	0	15	1	15
7952.1250	7952.0866	0.0384	16	3	14	2	18	1	18	15	3	13	2	17	1	17
7952.1250	7952.0895	0.0355	16	3	14	1	16	2	18	15	3	13	1	15	2	17
7952.1250	7952.0923	0.0327	16	3	14	1	17	2	17	15	3	13	1	16	2	16
7952.1250	7952.1008	0.0242	16	3	14	0	16	1	17	15	3	13	0	15	1	16
7952.1250	7952.0902	0.0348	16	3	14	1	16	1	16	15	3	13	1	15	1	15
7964.0459	7964.0232	0.0227	16	8	8	2	18	1	18	15	8	7	2	17	1	17
7964.0459	7964.0231	0.0228	16	8	9	2	18	1	18	15	8	8	2	17	1	17
7964.0459	7964.0256	0.0203	16	8	8	2	18	2	20	15	8	7	2	17	2	19
7964.0459	7964.0255	0.0204	16	8	9	2	18	2	20	15	8	8	2	17	2	19
7964.0459	7964.0212	0.0247	16	8	9	0	16	2	18	15	8	8	0	15	2	17
7964.0459	7964.0217	0.0242	16	8	9	1	16	2	18	15	8	8	1	15	2	17
7964.0459	7964.0213	0.0246	16	8	8	0	16	2	18	15	8	7	0	15	2	17
7964.0459	7964.0217	0.0242	16	8	8	1	16	2	18	15	8	7	1	15	2	17
7964.0459	7964.0230	0.0229	16	8	8	2	18	0	18	15	8	7	2	17	0	17
7964.0459	7964.0230	0.0229	16	8	9	2	18	0	18	15	8	8	2	17	0	17
7964.0682	7964.0729	-0.0047	16	8	8	2	18	1	17	15	8	7	2	17	1	16
7964.0682	7964.0808	-0.0126	16	8	9	1	17	2	19	15	8	8	1	16	2	18
7964.0682	7964.0728	-0.0046	16	8	9	2	18	1	17	15	8	8	2	17	1	16
7964.0682	7964.0749	-0.0067	16	8	8	2	18	2	19	15	8	7	2	17	2	18
7964.0682	7964.0709	-0.0027	16	8	8	2	18	1	19	15	8	7	2	17	1	18
7964.0682	7964.0809	-0.0127	16	8	8	1	17	2	19	15	8	7	1	16	2	18
7964.0682	7964.0748	-0.0066	16	8	9	2	18	2	19	15	8	8	2	17	2	18
7964.0682	7964.0708	-0.0026	16	8	9	2	18	1	19	15	8	8	2	17	1	18
7964.1478	7964.1308	0.0170	16	8	9	1	17	2	18	15	8	8	1	16	2	17
7964.1478	7964.1202	0.0276	16	8	9	2	18	2	18	15	8	8	2	17	2	17
7964.1478	7964.1341	0.0137	16	8	8	2	17	1	18	15	8	7	2	16	1	17
7964.1478	7964.1309	0.0169	16	8	8	1	17	2	18	15	8	7	1	16	2	17
7964.1478	7964.0902	0.0576	16	8	8	2	17	2	19	15	8	7	2	16	2	18
7964.1478	7964.1203	0.0275	16	8	8	2	18	2	18	15	8	7	2	17	2	17
7964.1478	7964.0902	0.0576	16	8	9	2	17	2	19	15	8	8	2	16	2	18
7964.1478	7964.1340	0.0138	16	8	9	2	17	1	18	15	8	8	2	16	1	17
7964.1478	7964.1259	0.0219	16	8	8	1	17	1	18	15	8	7	1	16	1	17
7964.1478	7964.1258	0.0220	16	8	9	1	17	1	18	15	8	8	1	16	1	17
7964.1778	7964.1899	-0.0121	16	8	9	2	16	1	17	15	8	8	2	15	1	16
7964.1778	7964.1900	-0.0122	16	6	10	2	16	1	17	15	6	9	2	15	1	16
7964.1778	7964.1759	0.0019	16	8	8	1	17	2	17	15	8	7	1	16	2	16
7964.1778	7964.1758	0.0020	16	8	9	1	17	2	17	15	8	8	1	16	2	16
7964.1778	7964.1828	-0.0050	16	6	10	2	17	2	17	15	6	9	2	16	2	16
7964.1778	7964.1828	-0.0050	16	6	11	2	17	2	17	15	6	10	2	16	2	16
7964.1778	7964.1960	-0.0182	16	8	9	2	16	2	17	15	8	8	2	15	2	16

7964.1778	7964.1960	-0.0182	16	10	6	2	16	2	17	15	10	5	2	15	2	16
7964.1778	7964.1926	-0.0148	16	8	8	2	16	1	15	15	8	7	2	15	1	14
7964.1778	7964.1925	-0.0147	16	8	9	2	16	1	15	15	8	8	2	15	1	14
7967.6483	7967.6457	0.0026	16	7	10	2	18	2	20	15	7	9	2	17	2	19
7967.6483	7967.6491	-0.0008	16	7	9	2	18	1	18	15	7	8	2	17	1	17
7967.6483	7967.6443	0.0040	16	7	10	2	18	1	18	15	7	9	2	17	1	17
7967.6483	7967.6490	-0.0007	16	7	9	2	18	0	18	15	7	8	2	17	0	17
7967.6483	7967.6442	0.0041	16	7	10	2	18	0	18	15	7	9	2	17	0	17
7967.6483	7967.6486	-0.0003	16	7	9	1	16	2	18	15	7	8	1	15	2	17
7967.6483	7967.6438	0.0045	16	7	10	1	16	2	18	15	7	9	1	15	2	17
7967.6483	7967.6486	-0.0003	16	7	9	0	16	2	18	15	7	8	0	15	2	17
7967.6483	7967.6437	0.0046	16	7	10	0	16	2	18	15	7	9	0	15	2	17
7967.6483	7967.6505	-0.0022	16	7	9	2	18	2	20	15	7	8	2	17	2	19
7967.6816	7967.6818	-0.0002	16	7	10	2	18	2	19	15	7	9	2	17	2	18
7967.6816	7967.6844	-0.0028	16	7	9	2	18	1	19	15	7	8	2	17	1	18
7967.6816	7967.7205	-0.0389	16	7	9	2	18	2	18	15	7	8	2	17	2	17
7967.6816	7967.7157	-0.0341	16	7	10	2	18	2	18	15	7	9	2	17	2	17
7967.6816	7967.6795	0.0021	16	7	10	2	18	1	19	15	7	9	2	17	1	18
7967.6816	7967.6995	-0.0179	16	7	9	1	17	2	19	15	7	8	1	16	2	18
7967.6816	7967.6947	-0.0131	16	7	10	1	17	2	19	15	7	9	1	16	2	18
7967.6816	7967.6866	-0.0050	16	7	9	2	18	2	19	15	7	8	2	17	2	18
7967.6816	7967.7039	-0.0223	16	7	9	2	17	2	19	15	7	8	2	16	2	18
7967.6816	7967.6991	-0.0175	16	7	10	2	17	2	19	15	7	9	2	16	2	18
7967.7250	7967.7532	-0.0282	16	7	9	2	16	2	18	15	7	8	2	15	2	17
7967.7250	7967.7483	-0.0233	16	7	10	2	16	2	18	15	7	9	2	15	2	17
7967.7250	7967.7314	-0.0064	16	7	10	1	17	2	18	15	7	9	1	16	2	17
7967.7250	7967.7334	-0.0084	16	7	9	1	17	1	18	15	7	8	1	16	1	17
7967.7250	7967.7286	-0.0036	16	7	10	1	17	1	18	15	7	9	1	16	1	17
7967.7250	7967.7362	-0.0112	16	7	9	1	17	2	18	15	7	8	1	16	2	17
7967.7250	7967.7371	-0.0121	16	7	9	2	17	1	18	15	7	8	2	16	1	17
7967.7250	7967.7323	-0.0073	16	7	10	2	17	1	18	15	7	9	2	16	1	17
7967.7625	7967.7862	-0.0237	16	7	9	2	16	1	17	15	7	8	2	15	1	16
7967.7625	7967.7847	-0.0222	16	9	8	2	16	2	17	15	11	5	2	15	2	16
7967.7625	7967.7652	-0.0027	16	7	10	1	17	2	17	15	7	9	1	16	2	16
7967.7625	7967.7701	-0.0076	16	7	9	1	17	2	17	15	7	8	1	16	2	16
7967.7625	7967.7731	-0.0106	16	5	11	2	17	2	17	15	3	12	2	16	2	16
7967.7625	7967.7683	-0.0058	16	7	10	2	17	2	17	15	5	11	2	16	2	16
7967.7625	7967.7896	-0.0271	16	7	9	2	16	2	17	15	7	8	2	15	2	16
7967.7625	7967.7813	-0.0188	16	5	12	2	16	1	17	15	5	11	2	15	1	16
7973.2645	7973.2661	-0.0016	16	6	11	2	18	1	18	15	6	10	2	17	1	17
7973.2645	7973.2661	-0.0016	16	6	11	2	18	0	18	15	6	10	2	17	0	17
7973.2645	7973.2654	-0.0009	16	6	11	1	16	2	18	15	6	10	1	15	2	17
7973.2645	7973.3110	-0.0465	16	6	11	1	17	2	19	15	6	10	1	16	2	18
7973.2645	7973.3128	-0.0483	16	6	11	2	17	2	19	15	6	10	2	16	2	18
7973.2645	7973.3346	-0.0701	16	6	11	1	17	1	18	15	6	10	1	16	1	17
7973.2645	7973.2654	-0.0009	16	6	11	0	16	2	18	15	6	10	0	15	2	17
7973.2645	7973.2914	-0.0269	16	6	11	2	18	2	19	15	6	10	2	17	2	18
7973.2645	7973.2666	-0.0021	16	6	11	2	18	2	20	15	6	10	2	17	2	19
7973.2645	7973.2902	-0.0257	16	6	11	2	18	1	19	15	6	10	2	17	1	18
7973.4280	7973.4685	-0.0405	16	6	10	1	17	2	19	15	6	9	1	16	2	18
7973.4280	7973.4482	-0.0202	16	6	10	2	18	2	19	15	6	9	2	17	2	18
7973.4280	7973.4469	-0.0189	16	6	10	2	18	1	19	15	6	9	2	17	1	18
7973.4280	7973.4715	-0.0435	16	6	10	2	18	2	18	15	6	9	2	17	2	17

7973.4280	7973.4930	-0.0650	16	6	10	1	17	2	18	15	6	9	1	16	2	17
7973.4280	7973.4704	-0.0424	16	6	10	2	17	2	19	15	6	9	2	16	2	18
7973.4280	7973.4920	-0.0640	16	6	10	1	17	1	18	15	6	9	1	16	1	17
7973.4280	7973.4479	-0.0199	16	6	10	2	18	1	17	15	6	9	2	17	1	16
7973.4280	7973.4941	-0.0661	16	6	10	2	17	1	18	15	6	9	2	16	1	17
7973.4280	7973.4955	-0.0675	16	6	10	2	17	2	18	15	6	9	2	16	2	17
7981.6730	7981.6668	0.0062	16	5	12	2	18	2	20	15	5	11	2	17	2	19
7981.6730	7981.6810	-0.0080	16	5	12	0	16	1	17	15	5	11	0	15	1	16
7981.6730	7981.6669	0.0061	16	5	12	2	18	1	18	15	5	11	2	17	1	17
7981.6730	7981.6810	-0.0080	16	5	12	1	16	1	17	15	5	11	1	15	1	16
7981.6730	7981.6815	-0.0085	16	5	12	2	18	1	19	15	5	11	2	17	1	18
7981.6730	7981.6817	-0.0087	16	5	12	2	18	2	19	15	5	11	2	17	2	18
7981.6730	7981.6815	-0.0085	16	5	12	2	18	1	17	15	5	11	2	17	1	16
7981.6730	7981.6670	0.0060	16	5	12	2	18	0	18	15	5	11	2	17	0	17
7981.6730	7981.6659	0.0071	16	5	12	1	16	2	18	15	5	11	1	15	2	17
7981.6730	7981.6660	0.0070	16	5	12	0	16	2	18	15	5	11	0	15	2	17
7981.7000	7981.7078	-0.0078	16	5	12	1	17	2	19	15	5	11	1	16	2	18
7981.7000	7981.7071	-0.0071	16	5	12	1	15	2	17	15	5	11	1	14	2	16
7981.7000	7981.7071	-0.0071	16	5	12	2	17	2	19	15	5	11	2	16	2	18
7981.7000	7981.6963	0.0037	16	5	12	2	18	2	18	15	5	11	2	17	2	17
7981.7000	7981.7220	-0.0220	16	5	12	1	17	2	18	15	5	11	1	16	2	17
7981.7000	7981.7227	-0.0227	16	5	12	2	17	1	18	15	5	11	2	16	1	17
7981.7000	7981.7229	-0.0229	16	5	12	2	17	2	18	15	5	11	2	16	2	17
7981.7000	7981.7078	-0.0078	16	5	12	1	17	1	17	15	5	11	1	16	1	16
7981.7000	7981.7078	-0.0078	16	5	12	1	17	0	17	15	5	11	1	16	0	16
7981.7000	7981.7225	-0.0225	16	5	12	1	17	1	18	15	5	11	1	16	1	17
7981.7375	7981.7483	-0.0108	16	5	12	2	16	1	16	15	5	11	2	15	1	15
7981.7375	7981.7637	-0.0262	16	5	12	2	16	1	15	15	5	11	2	15	1	14
7981.7375	7981.7489	-0.0114	16	5	12	2	16	2	14	15	5	11	2	15	2	13
7981.7375	7981.7379	-0.0004	16	7	10	2	17	0	17	15	7	9	2	16	0	16
7981.7375	7981.7628	-0.0253	16	7	10	2	16	2	17	15	9	7	2	15	2	16
7981.7375	7981.7479	-0.0104	16	5	12	2	16	2	18	15	5	11	2	15	2	17
7981.7375	7981.7640	-0.0265	16	3	14	2	16	1	17	15	3	13	2	15	1	16
7981.7375	7981.7484	-0.0109	16	5	12	2	16	0	16	15	5	11	2	15	0	15
7981.7375	7981.7634	-0.0259	16	5	12	0	16	2	15	15	7	9	0	15	2	14
7984.9000	7984.8904	0.0096	16	5	11	2	18	1	17	15	5	10	2	17	1	16
7984.9000	7984.8894	0.0106	16	5	11	0	16	1	17	15	5	10	0	15	1	16
7984.9000	7984.9026	-0.0026	16	5	11	2	18	2	18	15	5	10	2	17	2	17
7984.9000	7984.8782	0.0218	16	5	11	2	18	2	20	15	5	10	2	17	2	19
7984.9000	7984.8904	0.0096	16	5	11	2	18	1	19	15	5	10	2	17	1	18
7984.9000	7984.8768	0.0232	16	5	11	1	16	2	18	15	5	10	1	15	2	17
7984.9000	7984.8786	0.0214	16	5	11	2	18	0	18	15	5	10	2	17	0	17
7984.9000	7984.8768	0.0232	16	5	11	0	16	2	18	15	5	10	0	15	2	17
7984.9000	7984.8905	0.0095	16	5	11	2	18	2	19	15	5	10	2	17	2	18
7984.9000	7984.8785	0.0215	16	5	11	2	18	1	18	15	5	10	2	17	1	17
7984.9390	7984.9402	-0.0012	16	5	11	1	17	2	18	15	5	10	1	16	2	17
7984.9390	7984.9423	-0.0033	16	5	11	2	17	1	18	15	5	10	2	16	1	17
7984.9390	7984.9424	-0.0034	16	5	11	2	17	2	18	15	5	10	2	16	2	17
7984.9390	7984.9290	0.0100	16	5	11	1	17	1	17	15	5	10	1	16	1	16
7984.9390	7984.9411	-0.0021	16	5	11	1	17	1	18	15	5	10	1	16	1	17
7984.9390	7984.9290	0.0100	16	5	11	1	17	0	17	15	5	10	1	16	0	16
7984.9390	7984.9289	0.0101	16	5	11	1	17	2	19	15	5	10	1	16	2	18
7984.9390	7984.9290	0.0100	16	5	11	2	17	2	19	15	5	10	2	16	2	18

7984.9390	7984.9526	-0.0136	16	5	11	1	17	2	17	15	5	10	1	16	2	16
7984.9390	7984.9286	0.0104	16	5	11	1	15	2	17	15	5	10	1	14	2	16
7984.9847	7985.0050	-0.0203	16	5	11	2	16	2	16	15	5	10	2	15	2	15
7984.9847	7984.9808	0.0039	16	5	11	2	16	2	14	15	5	10	2	15	2	13
7984.9847	7984.9925	-0.0078	16	3	13	2	16	2	15	15	1	14	2	15	2	14
7984.9847	7984.9794	0.0053	16	5	11	2	16	2	18	15	5	10	2	15	2	17
7984.9847	7984.9933	-0.0086	16	5	11	2	16	1	17	15	5	10	2	15	1	16
7984.9847	7984.9915	-0.0068	16	5	11	2	16	2	17	15	5	10	2	15	2	16
7984.9847	7984.9801	0.0046	16	5	11	2	16	0	16	15	5	10	2	15	0	15
7984.9847	7984.9800	0.0047	16	5	11	2	16	1	16	15	5	10	2	15	1	15
7984.9847	7984.9927	-0.0080	16	5	11	2	16	1	15	15	5	10	2	15	1	14
7985.7860	7985.7601	0.0259	16	4	13	0	16	2	18	15	4	12	0	15	2	17
7985.7860	7985.7589	0.0271	16	4	13	2	18	2	20	15	4	12	2	17	2	19
7985.7860	7985.7705	0.0155	16	4	13	2	18	1	19	15	4	12	2	17	1	18
7985.7860	7985.7812	0.0048	16	4	13	1	17	2	19	15	4	12	1	16	2	18
7985.7860	7985.7701	0.0159	16	4	13	2	18	2	19	15	4	12	2	17	2	18
7985.7860	7985.7779	0.0081	16	4	13	2	17	2	19	15	4	12	2	16	2	18
7985.7860	7985.7928	-0.0068	16	4	13	1	17	1	18	15	4	12	1	16	1	17
7985.7860	7985.7594	0.0266	16	4	13	2	18	1	18	15	4	12	2	17	1	17
7985.7860	7985.7595	0.0265	16	4	13	2	18	0	18	15	4	12	2	17	0	17
7985.7860	7985.7599	0.0261	16	4	13	1	16	2	18	15	4	12	1	15	2	17

References

[1] F. Neese, "The ORCA program system," *Wiley Interdisciplinary Reviews: Computational Molecular Science*, vol. 2, no. 1, pp. 73–78, 2012.

[2] F. Neese, "Software update: The ORCA program system—Version 5.0," *Wiley Interdisciplinary Reviews: Computational Molecular Science*, vol. 12, no. 5, p. e1606, 2022.

[3] A. D. Becke, "Density-functional thermochemistry. III. The role of exact exchange," *The Journal of chemical physics*, vol. 98, no. 7, pp. 5648–5652, 1993.

[4] C. Lee, W. Yang, and R. G. Parr, "Development of the Colle-Salvetti correlation-energy formula into a functional of the electron density," *Physical review B*, vol. 37, no. 2, p. 785, 1988.

[5] S. H. Vosko, L. Wilk, and M. Nusair, "Accurate spin-dependent electron liquid correlation energies for local spin density calculations: a critical analysis," *Canadian Journal of physics*, vol. 58, no. 8, pp. 1200–1211, 1980.

[6] P. J. Stephens, F. J. Devlin, C. F. Chabalowski, and M. J. Frisch, "Ab initio calculation of vibrational absorption and circular dichroism spectra using density functional force fields," *The Journal of physical chemistry*, vol. 98, no. 45, pp. 11623–11627, 1994.

[7] J. K. G. Watson, "Determination of Centrifugal Distortion Coefficients of Asymmetric-Top Molecules," *The Journal of Chemical Physics*, vol. 46, pp. 1935–1949, 03 1967.

[8] C. Western, "PGOPHER, a Program for Simulating Rotational, Vibrational and Electronic Structure," *Journal of Quantitative Spectroscopy & Radiative Transfer*, vol. 186, pp. 221–242, 2016.

[9] D. Schmitz, V. A. Shubert, T. Betz, and M. Schnell, "Multi-resonance effects within a single chirp in broadband rotational spectroscopy: The rapid adiabatic passage regime for benzonitrile," *Journal of Molecular Spectroscopy*, vol. 280, pp. 77–84, 2012.

[10] V. L. Heywood, T. P. Alford, J. J. Roeleveld, S. J. L. Deprez, A. Verhoofstad, J. I. van der Vlugt, S. R. Domingos, M. Schnell, A. P. Davis, and T. J. Mooibroek, "Observations of tetrel bonding between sp^3-carbon and THF," *Chemical science*, vol. 11, no. 20, pp. 5289–5293, 2020.

[11] E. R. Johnson, S. Keinan, P. Mori-Sánchez, J. Contreras-García, A. J. Cohen, and W. Yang, "Revealing noncovalent interactions," *Journal of the American Chemical Society*, vol. 132, no. 18, pp. 6498–6506, 2010.

[12] T. Lu and Q. Chen, “Visualization analysis of weak interactions in chemical systems,” *Comprehensive Computational Chemistry*, vol. 2, pp. 240–264, 2024.

[13] R. Boto, F. Peccati, R. Laplaza, A. Carbone, J.-P. Piquemal, Y. Maday, and J. Contreras-García, “NCIPLOT4: A new step towards a fast quantification of noncovalent interactions,” 2020.

[14] J. Contreras-García, E. R. Johnson, S. Keinan, R. Chaudret, J.-P. Piquemal, D. N. Beratan, and W. Yang, “NCIPLOT: a program for plotting noncovalent interaction regions,” *Journal of chemical theory and computation*, vol. 7, no. 3, pp. 625–632, 2011.

[15] E. F. Pettersen, T. D. Goddard, C. C. Huang, G. S. Couch, D. M. Greenblatt, E. C. Meng, and T. E. Ferrin, “UCSF Chimera—a visualization system for exploratory research and analysis,” *Journal of computational chemistry*, vol. 25, no. 13, pp. 1605–1612, 2004.
